# Supplementary material for: Patient experience and perceived acceptability of whole-body magnetic resonance imaging for staging colorectal and lung cancer compared with current staging scans: a qualitative study
Source: BMJ Open. 2017 Sep 6;7(9):e016391. doi: 10.1136/bmjopen-2017-016391 (PMC5588966; doi:10.1136/bmjopen-2017-016391)
Supplement: Author's manuscript [file bmjopen-2017-016391.draft_revisions.pdf]

Patient acceptability of whole body magnetic resonance imaging for staging colorectal and lung cancer: a qualitative study

Journal:	*BMJ Open*
Manuscript ID	bmjopen-2017-016391
Article Type:	Research
Date Submitted by the Author:	10-Feb-2017
Complete List of Authors:	Evans, Ruth; Birkbeck University of London, Psychological Sciences<br>Taylor, Stuart; University College London Hospitals NHS Foundation Trust, Centre for Medical Imaging<br>Janes, Sam; University College London, Respiratory Medicine<br>Halligan, Steve; University College London, Centre for Medical Imaging<br>Morton, Alison; Patient Representative<br>Navani, Neal; University College London, Respiratory medicine<br>Oliver, Alf; Patient Representative<br>Rockall, Andrea; Royal Marsden NHS Foundation Trust, Radiology<br>Teague, Jonathan; Cancer Research UK and UCL Clinical Trials Centre<br>Miles, Anne; Birkbeck, University of London,
**Primary Subject Heading**:	Radiology and imaging
Secondary Subject Heading:	Oncology, Patient-centred medicine, Qualitative research
Keywords:	Magnetic resonance imaging < RADIOLOGY & IMAGING, Respiratory tract tumours < ONCOLOGY, Gastrointestinal tumours < ONCOLOGY, QUALITATIVE RESEARCH

SCHOLARONE™
Manuscripts

Title: Patient acceptability of whole body magnetic resonance imaging for staging colorectal and lung cancer: a qualitative study.

Authors: REC Evans[a], SA Taylor[b], S Janes[c], S Halligan[b], A Morton[d], N Navani[c], A Oliver[d], A Rockall[e], J Teague[f] and A Miles[a]

[a] Psychological Sciences, Birkbeck, University of London, Malet Street, London, WC1E 7HX

[b] Centre for Medical Imaging, Division of Medicine, University College London, Gower Street, London, WC1E 6BT

[c] Lungs for Living Research Centre, Division of Medicine, University College London, Gower Street, London, WC1E 6BT

[d] Patient Representative, no institutional address available

[e] Department of Surgery and Cancer, Imperial College London, Kensington, London SW7 2AZ

[f] CR UK &UCL Clinical Trials Centre, 90 Tottenham Court Road, London, W1T 4TJ

Corresponding author: Dr Anne Miles, Psychological Science, Birkbeck, University of London, Malet Street, London, WC1E 7HX, ae.miles@bbk.ac.uk, Tel: +44 20 7631 6488

Funding statement:

This project was funded by the National Institute of Health Research health technology assessment NIHR HTA programme (project number 10/68/01) and will be published in full in Health Technology Assessment. The project is supported by researchers at the National Institute for Health Research University College London Hospitals Biomedical Research Centre.

For peer review only - http://bmjopen.bmj.com/site/about/guidelines.xhtml

Department of Health disclaimer:

This report presents independent research commissioned by the National Institute for Health Research (NIHR). The views and opinions expressed by authors in this publication are those of the authors and do not necessarily reflect those of the NHS, the NIHR, NETSCC or the HTA programme or the Department of Health. The views and opinions expressed by the interviewees in this publication are those of the interviewees and do not necessarily reflect those of the authors, those of the NHS, the NIHR, MRC, CCF, NETSCC or the HTA programme or the Department of Health.

Competing interests

We have read and understood BMJ policy on declaration of interests and declare that we have no competing interests.

Authors' contribution

SA Taylor, S Janes, S Halligan, A Morton, N Navani, A Oliver, A Rockall and A Miles designed the clinical trial protocol which includes this study. J Teague assisted in patient recruitment. REC Evans and A Miles interviewed patients, analysed and interpreted the data. REC Evans wrote the first draft of the manuscript. All authors reviewed the manuscript and approved the final draft.

Acknowledgments:

The authors would like to acknowledge the contribution of the following individuals to the clinical trial which provided the data presented in this paper: A Aboagye, L Agoramoorthy, S Ahmed, A Amadi, G Anand, G Atkin, A Austria, S Ball, F Bazari, R Beable, S Beare, H Beedham, T Beeston, N Bharwani, G Bhatnagar, A Bhowmik, L Blakeway, D Blunt, P

For peer review only - http://bmjopen.bmj.com/site/about/guidelines.xhtml

Boavida, D Boisfer, D Breen, J Bridgewater, S Burke, R Butawan, Y Campbell, E Chang, D Chao, S Chukundah, B Collins, C Collins, V Conteh, J Couture, J Crosbie, H Curtis, A Daniel, L Davis, K Desai, M Duggan, S Ellis, C Elton, A Engledow, C Everitt, S Ferdous, A Frow, M Furneaux, N Gibbons, R Glynne-Jones, A Gogbashian, V Goh, S Gourtsoyianni, A Green, Laura Green, Liz Green, A Groves, A Guthrie, E Hadley, A Hameeduddin, G Hanid, S Hans, B Hans, A Higginson, L Honeyfield, H Hughes, J Hughes, L Hurl, E Isaac, M Jackson, A Jalloh, R Jannapureddy, A Jayme, A Johnson, E Johnson, P Julka, J Kalasthry, E Karapanagiotou, S Karp, C Kay, J Kellaway, S Khan, D Koh, T Light, P Limbu, S Lock, I Locke, T Loke, A Lowe, N Lucas, S Maheswaran, S Mallett, E Marwood, J McGowan, F Mckirdy, T Mills-Baldock, T Moon, V Morgan, S Morris, S Nasseri, P Nichols, C Norman, E Ntala, A Nunes, A Obichere, J O'Donohue, I Olaleye, A Onajobi, T O'Shaughnessy, A Padhani, H Pardoe, W Partridge, U Patel, K Perry, W Piga, D Prezzi, K Prior, S Punwani, J Pyers, H Rafiee, F Rahman, I Rajanpandian, S Ramesh, S Raouf, K Reczko, A Reinhardt, D Robinson, P Russell, K Sargus, E Scurr, K Shahabuddin, A Sharp, B Shepherd, H Sidhu, I Simcock, C Simeon, A Smith, D Smith, D Snell, J Spence, R Srirajaskanthan, V Stachini, S Stegner, J Stirling, N Strickland, K Tarver, M Thaha, M Train, S Tulmuntaha, N Tunariu, K van Ree, A Verjee, C Wanstall, S Weir, S Wijeyekoon, J Wilson, S Wilson, T Win, L Woodrow, D Yu.

Data sharing statement: Extra data from anonymised transcripts are available by emailing ae.miles@bbk.ac.uk

For peer review only

ABSTRACT:

Objective: To determine the acceptability of whole body magnetic resonance imaging (WB-MRI) staging among patients with highly suspected or known colorectal or lung cancer.

Design: Qualitative study using one-to-one interviews with thematic analysis.

Setting: Patients recruited from 10 hospitals in London, East and South East England between March 2013 and July 2014.

Participants:51 patients (31 male, age range 40 to 89 years), with varying levels of social deprivation were recruited consecutively from two parallel clinical trials comparing the diagnostic accuracy and cost-effectiveness of WB-MRI with standard scans for staging colorectal and lung cancer ('XX- C' and 'XX-L').

Results: In general WB-MRI presented a greater challenge than standard scans, although all but 4 patients completed the WB-MRI. Key challenges were: enclosed space, noise and scan duration; reduced patient tolerance was associated with claustrophobia, pulmonary symptoms and existing co-morbidities. Coping strategies facilitated scan tolerance and were grouped into 1) those intended to help with physical and emotional challenges, and 2) those focused on motivation to complete the scan e.g. focusing on health benefit. Positive staff interaction bolstered patients' ability to cope with WB-MRI, while negative staff interactions had the opposite effect.

Conclusions: Although WB-MRI was perceived as more challenging than standard scans, it was sufficiently acceptable and tolerated by most patients to potentially replace them if appropriate.

Trial Registration: ISRCTN43958015 and ISRCTN50436483

Keywords: Cancer Oncology Neoplasms Magnetic Resonance Imaging Patient Satisfaction

Word Count: 3292

For peer review only - http://bmjopen.bmj.com/site/about/guidelines.xhtml

ARTICLE SUMMARY

Strengths and limitations of this study

- This study is the first to qualitatively assess acceptability of whole body MRI in patients with highly suspected lung or colorectal cancer.
- Qualitative methodology, and large numbers recruited (with wide age range and deprivation levels), enabled us to observe that there is a range of intensity of difficulties experienced during the whole body MRI.
- Assessment within the context of a research trial, with some patient declining participation due to claustrophobia, might have led to an over estimation of acceptability.

For peer review only

BACKGROUND

Current NICE recommended staging pathways for lung and colorectal cancer are often complex and time consuming, involving several different imaging tests and potentially adding to the physical and psychological burden of patients with suspected cancer (1,2). More accurate and streamlined staging could improve patient outcomes both by triaging to optimal therapy and decreasing the time between diagnosis and treatment (3). Whole body magnetic resonance imaging (WB-MRI) has been advocated as an accurate and efficient "one stop shop" staging investigation that could potentially replace current strategies (4) and two parallel, multi-centre, prospective cohort studies (ref removed) investigating the diagnostic accuracy and cost-effectiveness of WB-MRI compared to standard pathways for newly diagnosed lung ('XX-L') and colon ('XX-C') cancer have recently completed. WB-MRI however can be stressful: its duration, 45 to 90 minutes, is longer than standard tests - CT takes a few seconds. MRI scanners are noisy and require full body and head immersion into a relatively narrow tube, often necessitating coils wrapped around the patient that restrict movement. Somewhere between 5-30% of patients experience distress associated with the anticipated and actual experience of undergoing MRI (5-9). Anxiety relates to the scan experience itself as well as the result (5). Severe claustrophobia can lead to premature scan termination in 1-15% of attempts (10,11), and distress is associated with elevated post scan anxiety (6), which can engender MRI fear or phobia (12-14), especially problematic in patients needing future MRI studies.

Increased physical and psychological vulnerability of patients with suspected cancer may reduce their ability to cope with a WB-MRI (2,15). Cancer diagnosis and subsequent treatment are still much feared despite recognition of better treatment outcomes (16,17); and patients may be experiencing shock, anxiety and worries about the future (15,18). While two small qualitative studies have investigated general patient experience of MRI

For peer review only - http://bmjopen.bmj.com/site/about/guidelines.xhtml

(19,20) there have been few descriptions of patients undergoing WB- MRI, particularly in those diagnosed with or highly suspected of having cancer.

Patient acceptability is central to the successful adoption of any new technology. Poor acceptability reduces adherence which in turn considerably blunts the impact of the technology, even if diagnostically superior to existing tests.

The aim of this study was to compare patient experience and acceptability of WB-MRI with standard staging tests, in the context of lung and colon cancer using patient interviews.

METHODS

There is little existing research describing patients' experiences of undergoing WB-MRI and we adopted a qualitative design as this provides the flexibility to capture rich descriptions of experiences without a priori knowledge of potential responses. One-to-one interviews were completed face to face (at home or at hospital) or via telephone. Individual interviews were conducted to facilitate expression of emotions and negative experiences that may be inhibited in a group setting. For two interviews a relative was present, to support translation where English was not the patient's first language. Patients were interviewed only once.

Trial and interview study recruitment

Patients were recruited to the 'XX' trials from 10 English NHS hospitals and consented to undergo WB-MRI in addition to standard staging investigations at one of five MRI scan hospital hubs. During trial consent, patients could opt into the interview study and were contacted, by psychology researchers by phone, as soon as possible after all imaging had been completed. Accelerated treatment pathways meant some interviews were undertaken

For peer review only - http://bmjopen.bmj.com/site/about/guidelines.xhtml

after treatment had commenced. Trial inclusion/ exclusion criteria are listed in the study protocol (reference removed).

The a priori total sample size was a minimum of 50, stratified by cancer site (25 lung and 25 colorectal patients). There was flexibility to recruit beyond this number to achieve saturation, i.e. the point beyond which further interviews contribute little new information, although this was not needed. The first 123 trial patients were approached and 91 (74%) agreed to be interviewed. Ultimately 51 patients (56%) participated as an additional patient contacted the trial team requesting to share her views. Reasons patients were not interviewed included: consent not initially offered or later retracted (n=40); participation in a related questionnaire study (n=14); withdrawal from the main trial (n=12); and interview quota reached before completion of all imaging tests (n=6).

Conducting the interview

Interviews were conducted from March 2013 to July 2014, by two female psychology researchers (PhD qualified), independent of the clinical trial team, with prior training and experience of conducting in-depth interviews with cancer patients (refs removed). The researchers introduced themselves as working with the doctors at the patient's hospital and explained that the interview was an opportunity to provide feedback about the WB-MRI and other tests the patients had had. A topic guide was developed (Box 1) to encourage discussion of key aspects of the staging experience including comparisons between WB-MRI and standard investigations. This guide was reviewed after completion of the first two interviews to assess whether it needed to be modified. Topic ordering and emphasis varied depending on relevance to each patient. Open ended questions were followed by verbal probing to elicit further clarification. Interviews lasted between 12 and 86 minutes (mean 48 minutes), and were recorded digitally. Participating patients were paid £20 plus travel expenses.

For peer review only - http://bmjopen.bmj.com/site/about/guidelines.xhtml

Additional measures

Age, gender and postcode data were collated. Postcode was used to calculate an area based deprivation score using the 2010 IMD scale (21) which was then categorised into quintiles (quintile 1 highest and quintile 5 lowest deprivation).

Data capture, coding and analysis

Interviews were transcribed verbatim. NVIVO, version 10, was used to manage data (tagging and labelling) while completing thematic analysis (22). A coding structure was developed by (psychology researcher name removed), using transcript review combined with a reflective log maintained during interviews, this was reviewed and agreed by both psychology researchers. This framework identified themes related to scan events, beliefs, attitudes, and emotional responses as well as coping strategies. Themes identified were influenced by prior research as well as emergent from the data. This process was iterative, with constant data comparisons to identify similarities and differences within and across individual interviews. Matrix tables were created with themes as columns and relevant data summarised into separate rows from each transcript. Matrix tables provided an overview of all 51 interviews, and ensured that the full range of experiences were represented in the final description. Patients were not asked to verify the thematic analysis so as not to increase participation burden.

Statistical analysis

Data were entered into SPSS, version 20, and Analysis Of Variance and Chi Square used to test continuous and categorical data respectively.

Ethical review

For peer review only - http://bmjopen.bmj.com/site/about/guidelines.xhtml

The trial protocol was reviewed and ethical permission granted by Camden and Islington NRES committee on 03/10/2012 (project number removed).

RESULTS

Table 1 gives full demographics of the interviewed cohort. All but four patients completed the WB-MRI. There were no significant differences in age, gender, or area-based deprivation between those who volunteered for interviews (n=91), and those who declined (n=32); mean age: 65 vs. 64, $F_{(1,121)}$ <1, p=0.527; % male: 64% vs. 56%, X^2 (df=1, n=123) =0.562, p=0.453; % in deprivation categories (Fisher's Exact Test chi-square: 1.834, p=0.788). Similarly there were no significant demographic differences between patients who were (n=51) or were not (n=40) interviewed; mean age 65 vs. 64, F $_{(1,89)}$ <1, p=0.579; gender % male: 53.4% vs. 46.6%, X^2 (df=1, n=91)=0.437, p=0.508, % in deprivation categories (Fisher's Exact chi-square: 5.247, p=0.254)

For peer review only

Table 1: Demographic characteristics of interview participants, by trial stream

		'XX- L'* % (n=25)	'XX-C'* %(n=26)	Group differences
Gender				
	Male	60 (15)	61.5 (16)	X^2=0.013, df=1,
	Female	40 (10)	38.5 (10)	p=0.910
Mean age (years)		65	64	t=0.261, df=49;
		(Sd=11 years)	(sd=9 years)	p=0.795

Area Deprivation Score (quintile)			
Highest 1	52.0 (13)	30.8 (8)	X^2=5.459
2	32.0 (8)	23.1 (6)	df=2,
Mid to low 3-5	16.0 (4)	46.2 (12)	p=0.065
I/v method			
Face to Face	20.0 (5)	26.9 (7)	X^2=0.339, df=1;
Phone	80.0 (20)	73.1 (19)	p=0.560
Median Interval between interview and WB-MRI	15 days (6-43 days)	20 days (1-63 days)	U=270.00, n=51, p=0.299

*XX-L' -lung cancer. 'XX-C' –colorectal cancer

Was the WB-MRI scan a challenge? Diversity of experience.

For patients recruited to 'XX-C', standard investigation included CT of the chest, abdomen and pelvis, with some undergoing an additional abdominal/ pelvic MRI. Patients recruited to 'XX-L' underwent at least a CT and PET-CT with a proportion undergoing bronchoscopy for tissue sampling.

WB-MRI was perceived to be more challenging than standard scans by many: *"I would say the most challenging of the MRI scans was the body scan"*s56 (60-69, male, Colorectal -'C'), like *"torture - medieval torture"*s12 (50-59,male,C) while CT was described as *"a walk in the park"*s42 (60-69, male, C), and *"very simple"*s35 (50-59, female, Lung – 'L'). PET-CT was described by some as *"so much easier"*s51 (70-79, female, L) and *"wasn't so unpleasant"* s12 (50-59, male, C). However there was variability in patient experience of

For peer review only - http://bmjopen.bmj.com/site/about/guidelines.xhtml

WB-MRI (see below), with some patients even enjoying it. The CT scan was reported as being a comparatively greater challenge by one patient because of the intravenous contrast *"you feel a bit, you know, woozy, funny, …and it did make me feel slightly off"* s63 (<50, male, C); another patient described the bronchoscopy as more unpleasant *"I'd rather have them scans again than have that tube down my throat again, that was the worst thing, and I hated it"* s73 (60-69, male, L).

What was difficult about the WB-MRI?

Factors identified as difficult were: claustrophobia, physical discomfort, noise, scan duration, as well as the challenge of coping with emotions elicited during the scan such as fear /panic and isolation. There was a range of reaction intensity, with some reporting positive experiences (Table 2).

Table 2: Problems and the diversity of challenge experienced related to WB-MRI

Problem	No challenge/ enjoyable	Low challenge	Medium/ High	Did not complete
Enclosed space	*"I'm not claustrophobic …that [enclosed space] doesn't frighten me" s36 (50-59, male,L)*	*"Maybe in the back of my mind you're worried about it [enclosed space]" s5(60-69, female, C)*	*"Coffin came to mind…initially, you feel like 'Oh god, it's so close in here'" s26(60-69, female,L)*	**"***I had this sense of being in a sarcophagus" s17(60-69, male, L)*

For peer review only - http://bmjopen.bmj.com/site/about/guidelines.xhtml

Noise	*"I thought it like music really…and it relaxed me"* s64(80+, male,C)	*"the noise was a bit…disconcerting because it was quite loud and quite sort of clanking and banging… …and it took some time getting used to it"* s63(<50, male,C)	*"that's one of the worst ones that I've had to go through with the noise… I felt like something was going to fall off and hit me."* s54(<50, female,L)	
Scan duration/ discomfort	*"It's actually quite comfortable in there"* s87(60-69, male,L)	*"It was not excruciatingly painful. It just ached a bit, that is all."* s18(60-69, male,C)	*"My back was uncomfortable. My, the back of my neck was uncomfortable…" "my brain was saying, "You can't go much longer…* s56(60-69, male,C)	
Negative Emotions (e.g. anxiety, panic, shock, distress)	*"I mean, there wasn't, you know, anything in particular to*	*"when I first went in there, I was quite shocked …. …I wondered*	*"when I first laid down in the scan I was frightened" s79(60-69,*	*I don't know whether the people doing the scan knew I*

For peer review only - http://bmjopen.bmj.com/site/about/guidelines.xhtml

	be frightening [sic]" s76(<50, male,C)	*what the hell was going on… after a while I quite enjoyed it"* s18(60-69, male,C)	*female,C)* and *"I'm lying there and all of a sudden, they put all these things on top of me… And I'm thinking, 'Oh, my God! …this is threatening'"*s53 (60-69, male,C)	*had lung cancer, I didn't feel any empathy or any sort of friendly attitude. I walked out of there feeling quite upset actually"* s30 (50-59,male,L)

Claustrophobia: patients described feeling trapped or buried in the WB-MRI "*it was the feeling of being sort of trapped because you can feel the machine all around your body"* s12 (50-59, male, C) and "*I had this sense of being in this sarcophagus"* s17 (60-69, male, L). Only one participant asked to terminate the scan early because of claustrophobia (s17).

Noise: Noise emmited from the MRI during data acquisition was aversive for some "*horrendous. It's horrible! "*s55 (60-69, female, L), but not all, *"it was quite good.… I would say, 'What's coming next? What's the next noise?' So, I enjoyed that"* s18 (60-69, male, C).

Scan duration and physical discomfort**:** The scan length differentiated WB-MRI from other tests "*the CT scan is very quick…you're in and out. Whereas, the MRI scan was very long"* s13 (60-69, female, C). Duration was linked to comfort: "*the back of my neck was uncomfortable, [the WB-MRI] maybe 15 minutes too long to be comfortable"* s56 (60-69,

For peer review only - http://bmjopen.bmj.com/site/about/guidelines.xhtml

male, C) In contrast a patient without discomfort viewed the long duration acceptable, *"I think it's over one and a half hour or something like that, so it was alright for me… is comfortable"* s19 (60-69, male, C).

Emotions elicited by WB-MRI: WB-MRI induced strong emotions in some:*"I was going to have a sort of panic attack and have to be taken out of the machine…this claustrophobia, the feeling of being...trapped, really" s12*(50-59, male, C). Worry and fear were also prompted by the novelty of the experience "*the oppression comes from the unknown"*s45 (70-79, male, C) and the implications of the WB-MRI scan result *"whether they'd find … it has gone everywhere and you know nothing you can do, I suppose that was the main worry"* s60 (60-69, female, L). However some suggested that it was preferable to know if their cancer had spread "*but that is a good thing, it will be dealt with"* s79 (60-69, female, C).

Explaining differences in experiences

Cancer type: Physical symptoms reported more frequently by those recruited to 'XX-L' were associated with discomfort, "*I found lying flat on my back, yes, very uncomfortable... I was coping okay for about 20, 25 minutes and then that's when this pressure or aching or uncomfortable feeling on my chest began to kick in"*s30 (50-59, male, L) and difficulty with breath holding "*they had to make me do it a few times because I couldn't hold it for the length of time they wanted me to … I've got COPD, whether that made it worse, I don't know"* s58 (60-69, male, L).

Existing musculo-skeletal problems: Sometimes these made the WB-MRI more uncomfortable, but not for all: *"I do suffer a bit from back pain… My back started to sort of*

For peer review only - http://bmjopen.bmj.com/site/about/guidelines.xhtml

be pretty uncomfortable" s42 (60-69, male, C), and *"I was worried about my neck being uncomfortable … because of arthritis but they managed to sort me out and then I managed to last the hour*"s18 (60-69, male, C).

Mental health co-morbidities: One patient with prior anxiety found the scan difficult, describing their anxiety as causing feelings of claustrophobia "*I'm not claustrophobic. I just felt that way because of the anxiety. When I get anxious, it feels like everything's just closing in on me"* s54 (<50, female, L).

Prior experiences: One patient terminated the scan because he didn't like being constrained and his aversion came as a surprise, *"a sense of being constrained and being strapped down which is a new one for me… I haven't encountered that situation before"*s17 (60-69, male, L). In contrast, another participant (s8: 60-69, male, C) who knew he suffered from severe claustrophobia requested a sedative and completed the scan with relative ease. Work experiences meant some patients were used to confined spaces and/or loud noises, *"it doesn't bother me. I've worked in pipes and tunnels and all sorts of places"*s91 (60-69, male, L) and "*if I wasn't in the trade I suppose that that could really freak you out because it's quite loud…"* s76 (<50, male, C). Vicarious experiences were sources of knowledge for patients without prior personal experience, and influenced expectations, sometimes reassuring "*I have a few friends and relatives… they have already done MRI, so I had something from them. this [is not] painful or anything like that. "* s19 (60-69, male, C); sometimes contrary to their own eventual experience, *"it was not as intimidating as I thought… I had heard people talking about going in to an MRI scanner, and they were telling me how nervous they were, and how they hated it…. And when I saw it, I thought, 'That's okay'"* s18 (60-69, male, C).

For peer review only - http://bmjopen.bmj.com/site/about/guidelines.xhtml

Staff contact: Some patients spoke of a sense of isolation when in the scanner and hearing a voice confirmed they were not alone *"just made me feel a bit confident that you wasn't on your own, you know?"* s58 (60-69, female, L), and acted as distraction *"the person speaking keeps my mind occupied*" s26 (60-69, female, L). Whether staff were perceived to be reassuring was also important for some and a change in staff mid-way through the scan was detrimental: "*she was constantly talking to me, I was fine. But then … another lady took over and she didn't talk as much … …when I panicked"* s58 (60-69, female, L) and *"People I've been speaking with beforehand… I've actually built up the initial … trust… Then, suddenly, a complete stranger is now telling me to breathe in and out… then said, ' I should be coming down now to inject you with dye'…* "*In my mind, I thought the absolutely bizarre"* s80 (60-69, male, C).

Coping: Patients adopted various coping strategies, categorised into two main groups: 1) strategies for coping with distressing thoughts, emotional responses, or physical sensations, e.g. mental distraction and relaxation. *"Because I love Cyprus I was thinking of Cyprus the whole time. And it sort of takes your mind off of what's going"*s55 (60-69, female, L) and *"breathing, breathing…And you know trying to sort of just to calm…Because you can't help but get anxious"*s60 (60-69, female, L); and 2) Strategies related to motivation to complete the scan, such as focusing on benefit beliefs: "*I was so fed up with pain that I would have done…you know, any investigation was better… it was [WB-MRI] something to get me better"*s14 (60-69, female, C). *"I was laying there and I was thinking, 'Well, it's for my own benefit so I can live with that'"*s8 (60-69, male, C).

Staff supported patients' coping with verbal reassurance, information provision, as well as making them physically comfortable.

For peer review only

Willingness to have another WB-MRI Scan

All patients stated they would undergo another WB-MRI scan if the doctor recommended it, including the four patients who had requested scan termination. However this agreement was offered with varying enthusiasm; *"well if I have to….I don't like it one bit but if it has to be done"* s73 (60-69, male, L) compared with *"yes without hesitation"* s63(<50,male,C).

CONCLUSIONS

We aimed to determine the acceptability of WB- MRI as a potential replacement for the modalities currently used for staging lung and colorectal cancer. Patients recognised that WB-MRI was different from other scans although the extent of the challenge varied considerably. The majority completed WB-MRI with just four patients requesting premature termination; all were prepared to attempt future WB-MRI. Patients adopted a variety of coping strategies. These centred on the physical and emotional responses experienced during scanning, as well as focusing on beliefs that bolstered motivation to complete the scan. The role of staff was vital to support vulnerable patients; positive interactions bolstered coping while negative interactions had the opposite effect.

To our knowledge, there are only two other comparable qualitative studies of patients' experiences of MRI (19,20). In common with our study the enclosed space, noise, physical discomfort and duration were indentified as challenging, and the need for staff support was also highlighted. The importance of a "trustful dialogue" between radiographer and patients, to facilitate coping strategies (19), resonate with comments made by patients in our study.

Noise and confinement are the most frequently cited negative aspects of MRI in quantitative work published over the last 20 years (6,12). Other studies identify additional factors

For peer review only - http://bmjopen.bmj.com/site/about/guidelines.xhtml

increasing the likelihood of premature scan termination, including acquisition position (head first, and/or prone); age and gender (middle age or female); prior experience (first scan or prior negative experience) and comorbidity (pain, anxiety) (6,13,23,24). Our results concur with some of these findings; we found evidence that co-morbidity (e.g. anxiety and physical symptoms) influenced experience although, unlike others (e.g. (12,14)) prior experience (positive or negative) generally increased coping ability.

Some patients feared that WB-MRI may reveal cancer dissemination and likened the magnet to a coffin or sarcophagus. It is possible that these feelings were more salient because the patients were contemplating a life threatening diagnosis. Carlsson and Carlsson (19) reported greater fear when MRI was used to confirm serious illness. WB-MRI for cancer staging comes at a point when patients are likely to be emotionally vulnerable. However, while the implications of scanning patients with co-morbidities and emotional vulnerability should be acknowledged, it is important to note that most completed the WB-MRI.

Our study suggests that good staff communication could reduce anxiety and boost coping strategies by acting as a source of distraction, motivation and emotional reassurance. The varied experiences encountered underline the need for staff interaction to be tailored. Advanced staff training to build rapport can reduce MRI non-completion rates and increase patient satisfaction (25,26). A recent UK survey suggests that whilst strategies are in place to help reduce MRI related anxiety (commonly written leaflets, verbal pre-scan information, music during scanning, dedicated staff support), these are not optimised yet, with resource restrictions a potential barrier to implementation (27).

Study limitations

For peer review only - http://bmjopen.bmj.com/site/about/guidelines.xhtml

The generalisability of our results may be limited. WB-MRI was performed within the context of a research trial and some patients declined participation citing claustrophobia. Acceptability of WB-MRI may be lower than our study suggests. However the patients were from a wide age range and deprivation levels, and were interviewed from a number of different hospitals. As a result, a diverse spread of experiences were documented and included some who ultimately were unable to tolerate scanning.

Clinical implications

Our study confirms that WB-MRI can be a challenging experience and that staff support is important in modifying scan related stress. Our study also highlights that patient experience is varied and for the most part, the scan was tolerated, suggesting WB-MRI could potentially replace existing modalities, assuming adequate diagnostic accuracy and cost-effectiveness. Experience of WB-MRI reflects the success of coping strategies adopted by patients and the quality of support they receive. Further research would facilitate understanding of the interrelationship between patients' experiences, the effectiveness of their coping strategies, and of the support received from staff such that patients and clinicians can benefit maximally from the diagnostic potential of WB-MRI.

For peer review only - http://bmjopen.bmj.com/site/about/guidelines.xhtml

For peer review only

Reference List

(1) Brocken P, Prins JB, Dekhuijzen PN et al.The faster the better?-A systematic review on distress in the diagnostic phase of suspected cancer, and the influence of rapid diagnostic pathways. *Psychooncology* 2012 Jan;21(1):1-10.

(2) Brocken P, van der Heijden EH, Oud KT et al.Distress in suspected lung cancer patients following rapid and standard diagnostic programs: a prospective observational study. *Psychooncology* 2015 Apr;24(4):433-41.

(3) Risberg T, Sorbye SW, Norum J et al.Diagnostic delay causes more psychological distress in female than in male cancer patients. *Anticancer Res* 1996 Mar;16(2):995-9.

(4) Ciliberto M, Maggi F, Treglia G, et al. Comparison between whole-body MRI and Fluorine-18-Fluorodeoxyglucose PET or PET/CT in oncology: a systematic review. *Radiol Oncol* 2013 Jul 30;47(3):206-18.

(5) Katz RC, Wilson L, Frazer N. Anxiety and its determinants in patients undergoing magnetic resonance imaging. *J Behav Ther Exp Psychiatry* 1994 Jun;25(2):131-4.

(6) MacKenzie R, Sims C, Owens RG et al. Patients' perceptions of magnetic resonance imaging. *Clin Radiol* 1995 Mar;50(3):137-43.

(7) McIsaac HK, Thordarson DS, Shafran R et al.Claustrophobia and the magnetic resonance imaging procedure. *J Behav Med* 1998;21(3):255-68.

(8) Melendez JC, McCrank E. Anxiety-related reactions associated with magnetic resonance imaging examinations. *JAMA* 1993 Aug 11;270(6):745-7.

(9) van MD, Klaming L, Weda H. Pinpointing moments of high anxiety during an MRI examination. *Int J Behav Med* 2014 Jun;21(3):487-95.

(10) Dewey M, Schink T, Dewey CF. Claustrophobia during magnetic resonance imaging: cohort study in 55,000 patients. *J Magn Reson Imaging* 2007;38(3):722-5.

(11) Eshed I, Althoff CE, Hamm B et al. Claustrophobia and premature termination of magnetic resonance imaging examinations. *J Magn Reson Imaging* 2007 Aug;26(2):401-4.

(12) Harris LM, Cumming SR, Menzies RG. Predicting anxiety in magnetic resonance imaging scans. *Int J Behav Med* 2004;11(1):1-7.

(13) Kilborn LC, Labbe EE. Magnetic resonance imaging scanning procedures: development of phobic response during scan and at one-month follow-up. *J Behav Med* 1990 Aug;13(4):391-401.

(14) Lukins R, Davan IG, Drummond PD. A cognitive behavioural approach to preventing anxiety during magnetic resonance imaging. *J Behav Ther Exp Psychiatry* 1997 Jun;28(2):97-104.

For peer review only - http://bmjopen.bmj.com/site/about/guidelines.xhtml

(15) Wiljer D, Walton T, Gilbert J et al.Understanding the needs of colorectal cancer patients during the pre-diagnosis phase. *J Cancer Educ* 2013;28(3):402-7.

(16) Lehto RH. Identifying Primary Concerns in Patients Newly Diagnosed With Lung Cancer. *Oncology Nursing Forum* 2011;38(4):440-7.

(17) Robb KA, Simon AE, Miles A et al.Public perceptions of cancer: a qualitative study of the balance of positive and negative beliefs. *BMJ Open* 2014;4(7):e005434.

(18) Martin MY., Fouad MN, Oster RA et al.What do cancer patients worry about when making decisions about treatment? Variation across racial/ethnic groups. *Support Care Cancer* 2014;22(1):233-44.

(19) Carlsson S, Carlsson E. 'The situation and the uncertainty about the coming result scared mebut interaction with the radiographers helped me through': a qualitative study on patients' experiences of magnetic resonance imaging examinations. *Journal of Clinical Nursing* 2013;22:3225-34.

(20) Tornqvist E, Mansson A, Larsson EM et al. It's like being in another world – patients' lived experience of magnetic resonance imaging. *Issues in Clinical Nursing* 2006;954-61.

(21) McLennan D, Barnes H, Noble M et al. The English Indices of Deprivation 2010. London: Department for Communities and Local Government; 2011.

(22) Braun V, Clarke V. Using thematic analysis in psychology. Qualitative Research in Psychology 2006; 3(2):77-101

(23) Mohlman J, Eldreth DA, Price RB et al. Predictors of unsuccessful magnetic resonance imaging scanning in older generalized anxiety disorder patients and controls. *J Behav Med* 2012;35:19-26.

(24) Thorpe S, Salkovskis PM, Dittner A. Claustrophobia in MRI: the role of cognitions. *Magnetic Resonance Imaging* 2008;26:1081-8.

(25) Elvira V.Lang. A Better Patient Experience Through Better Communication. *J Radiol Nurs* 2012;31((4)):114-9.

(26) Lang EV1, Ward C, Laser E. Effect of team training on patients' ability to complete MRI examinations**.** *Acad Radiol* 2010;17(1):18-23.

(27) Tischler V, Calton T, Williams M et al.Patient anxiety in magnetic resonance imaging centres: Is further intervention needed? *Radiography* 2008;14:265-6.

For peer review only - http://bmjopen.bmj.com/site/about/guidelines.xhtml

Box 1: Key interview topics

- *I understand that you have been having a number of scans to investigate your symptoms, can you tell me what's been happening?*
- *Can you describe what it feels like physically to have the scan?*
- *How did you feel during/after/about the test?*
- *What were the staff like?*
- *What did you do to cope with [problem identified by patient e.g. noise, keeping still, etc]*
- *Would you have another WB-MRI if the doctor recommended it?*

For peer review only

Streamline C

Streamlining Staging of Colorectal Cancer with Whole Body MRI

Trial Sponsor: University College London (UCL)
Trial Sponsor reference: UCL/11/0097

Trial funder: NIHR HTA
Funder reference: 10/68/01
ISRCTN no: 43958015

Protocol version no: 6.0
Protocol version date: 06/09/2016

This project was funded by the National Institute for Health Research (NIHR) Health Technology Assessment (HTA) programme *(project number 10/68/01)*

For peer review only

Protocol version 6.0, 06/09/2016 Authorisation signatures

Name & Role:	Signature:	Date authorised:
**Chief Investigator:** Professor Stuart Taylor Professor of Medical Imaging, UCL	[illegible]	14/10/16
**For the Sponsor:** Professor Jonathan Ledermann Director, UCL CTC	[illegible]	14 Oct 2016
Sandy Beare Tumour Group Lead, UCL CTC	[illegible]	14/10/2016

Please note: This trial protocol must not be applied to patients treated outside the Streamline C trial. UCL CTC can only ensure that approved trial investigators are provided with amendments to the protocol.

For peer review only - http://bmjopen.bmj.com/site/about/guidelines.xhtml

Coordinating Centre:

For general queries, supply of trial documentation and central data management please contact:

Streamline C Trial Coordinator
Cancer Research UK & UCL Cancer Trials Centre (UCL CTC)
90 Tottenham Court Road
London
W1T 4TJ

Tel: +44 (0) 20 7679 9878
Fax: +44 (0) 20 7679 9871
09:00 to 17:00 Monday to Friday

Email: ctc.streamlineC@ucl.ac.uk

Other trial contacts:

Chief Investigator: Professor Stuart Taylor
Address: Dept Specialist X Ray, Level 2 Podium,
University College London Hospital (UCLH)
235 Euston Road
London
NW1 2BU

Trial Management Group (TMG):

Prof Stuart Taylor	Professor of Medical Imaging	UCLH
Dr Dow-Mu Koh	Consultant Radiologist	The Royal Marsden
Prof Vicky Goh	Professor of Clinical Cancer Imaging	Guys and St Thomas
Prof Anwar Padhani	Clinical Lead in MRI	Mount Vernon
Dr Shonit Punwani	Senior Lecturer in Medical Imaging	UCLH
Prof Andrea Rockall	Professor of Cancer Imaging	Imperial College London
Prof Steve Halligan	Professor of Radiology	UCL
Dr Neal Navani	Consultant in Thoracic Medicine	UCLH
Prof Sam Janes	Wellcome Senior Fellow	UCLH
Dr John Bridgewater	Consultant Oncologist	UCLH
Alison Morton	Patient Representative	
Alf Oliver	Patient Representative	
Dr Anne Miles	Senior Lecturer in Health Psychology	Birkbeck College
Prof Steve Morris	Professor of Health Economics	UCL
Dr Susan Mallett	Senior Medical Statistician	University of Birmingham
Krystyna Reczko	Trial Coordinator	UCL CTC
Katie Prior	Data Manager	UCL CTC
Marian Duggan (maternity leave)	Senior Trial Coordinator	UCL CTC
Abby Sharp (maternity cover)	Senior Trial Coordinator	UCL CTC
Dr Sandy Beare	Tumour Group Lead	UCL CTC

For peer review only - http://bmjopen.bmj.com/site/about/guidelines.xhtml

Table of Contents

1. PROTOCOL SUMMARY 7
1.1. SUMMARY OF TRIAL DESIGN 7
1.2. TRIAL SCHEMA 9
2. INTRODUCTION 10
2.1. BACKGROUND 10
3. TRIAL OVERVIEW 12
3.1. PRIMARY OBJECTIVE 12
3.2. SECONDARY OBJECTIVES 12
3.3. TRIAL ACTIVATION 13
4. SELECTION OF SITES/SITE INVESTIGATORS 14
4.1. SITE SELECTION 14
4.1.1. Selection of Principal Investigator and other investigators at sites 14
4.1.2. Training requirements for site staff 14
4.2. SITE INITIATION AND ACTIVATION 15
4.2.1. Site initiation 15
4.2.2. Required documentation 15
4.2.3. Site activation letter 15
5. INFORMED CONSENT 16
6. SELECTION OF PATIENTS 18
6.1. PRE-REGISTRATION EVALUATION 18
6.2. SCREENING LOG 18
6.3. MULTI DISCIPLINARY TEAM (MDT) MEETING LOG 18
6.4. PATIENT ELIGIBILITY 18
6.4.1. Patient Inclusion Criteria 18
6.4.2. Exclusion Criteria 19
6.4.3. Pregnancy and Birth Control 19
7. REGISTRATION PROCEDURES 20
7.1. REGISTRATION 20
7.2. REGISTRATION PACKS 20
7.3. BOOKING WHOLE BODY MRI 20
8. PATIENT MANAGEMENT AND TRIAL PROCEDURES 22
8.1. SUMMARY 22
8.1.1. Staging and Treatment Decisions 22
8.1.2. Patient experience Interviews and Questionnaires (Optional) 23
8.1.3. Quality of Life Questionnaires and Patient Diaries 23
8.2. DETAILED DESCRIPTION 25
8.2.1. Standard care 25
8.2.2. Trial Imaging 25
8.2.3. Release of Conventional and trial WB-MRI findings 27
8.2.4. Final treatment plan 28
8.2.5. Early release of WB-MRI findings 29
8.2.6. Time to full staging 29
8.2.7. Patient follow up 30
8.2.8. Patient diaries and EQ-5D Quality of Life questionnaires 30
8.2.9. Final reference standard for tumour stage (Consensus Meetings) 30
8.2.10. Health psychology assessment 32

For peer review only - http://bmjopen.bmj.com/site/about/guidelines.xhtml

8.2.11. *Cost Effectiveness Assessment* ... *35*
8.2.12. *Nested substudies* ... *37*

9. DATA MANAGEMENT GUIDELINES ... 39

9.1. Completing Case Report Forms ... 39
9.2. Missing Data ... 39
9.3. Timelines for data return ... 39
9.4. Data Queries ... 40

10. SAFETY REPORTING ... 41

11. INCIDENT REPORTING ... 42

12. TRIAL MONITORING AND OVERSIGHT ... 43

12.1. Central Monitoring ... 43
12.2. 'For Cause' On-Site Monitoring ... 43
12.3. Oversight Committees ... 44
12.3.1. *Trial Management Group (TMG)* ... *44*
12.3.2. *Trial Steering Committee (TSC)* ... *44*
12.3.3. *Independent Data Monitoring Committee (IDMC)* ... *44*
12.3.4. *Role of UCL CTC* ... *44*

13. WITHDRAWAL OF PATIENTS ... 45

14. TRIAL CLOSURE ... 46

14.1. End of Trial ... 46
14.2. Archiving of Trial Documentation ... 46
14.3. Early discontinuation of trial ... 46
14.4. Withdrawal from trial participation by a site ... 46

15. QUALITY ASSURANCE ... 47

16. STATISTICS ... 48

16.1. Sample size calculation ... 48
16.2. Population for analysis ... 49
16.3. Analysis of the primary endpoint ... 49
16.4. Analysis of secondary endpoints ... 50
16.4.1. *Efficacy (secondary)* ... *50*
16.4.2. *Safety* ... *52*
16.4.3. *Economic evaluation* ... *52*
16.4.4. *Health psychology assessment* ... *53*
16.4.5. *Handling missing data* ... *54*
16.5. Interim analyses ... 54

17. ETHICAL APPROVALS ... 55

17.1. Ethical Approval ... 55
17.2. Site Approvals ... 55
17.3. Protocol Amendments ... 55
17.4. Patient Confidentiality & Data Protection ... 56

18. SPONSORSHIP AND INDEMNITY ... 57

18.1. Sponsor Details ... 57
18.2. Indemnity ... 57

19. FUNDING ... 58

20. PUBLICATION POLICY ... 59

For peer review only - http://bmjopen.bmj.com/site/about/guidelines.xhtml

Streamline C

21. REFERENCES 60
APPENDIX 1: ABBREVIATIONS 63
APPENDIX 2: CONSENT PROCESS 65
APPENDIX 3: MINIMUM WB-MRI PROTOCOL DATASET 67
APPENDIX 4: EXPLORATORY ANALYSIS 69
APPENDIX 5: PROTOCOL VERSION HISTORY 71

For peer review only

For peer review only - http://bmjopen.bmj.com/site/about/guidelines.xhtml

1. Protocol Summary

1.1. Summary of Trial Design

**Title:**	Streamlining Staging of Colorectal Cancer with Whole Body MRI
**Short Title/acronym:**	Streamline C
**Sponsor name & reference:**	UCL/11/0097
**Funder name & reference:**	NIHR HTA
**ISRCTN no:**	43958015
**Design:**	Multicentre comparison
**Overall aim:**	To evaluate whether early whole body magnetic resonance Imaging (WB-MRI) increases per patient sensitivity for metastasis in colorectal cancer compared to standard NICE-approved diagnostic pathways.
**Primary endpoint:**	Per patient sensitivity for metastasis detection by whole body MRI (WB-MRI) compared to standard staging pathways in newly diagnosed colorectal cancer
**Secondary endpoints:**	1. The time and test number taken to reach, and the nature of, the first major treatment decision based on WB-MRI in comparison to standard staging pathways.<br>2. Diagnostic accuracy of WB-MRI and conventional staging pathways for local tumour staging and detection of metastasis in comparison to an expert derived consensus reference standard.<br>3. Lifetime incremental cost and cost-effectiveness of staging using WB-MRI compared to standard diagnostic pathways.<br>4. Patient experience of staging using WB-MRI in comparison to standard diagnostic pathways and priorities placed by patients on differing attributes related to competing staging pathways.<br>5. Inter-observer variability in WB-MRI analysis and effect of diagnostic confidence on staging accuracy.<br>6. Diagnostic accuracy of limited T1 and diffusion weighted sequences compared to full multi-sequence WB-MRI protocols.
**Target accrual:**	360
**Inclusion & exclusion criteria:**	**Inclusion criteria:**<br>• Adult patients (18 or over) with histologically

For peer review only - http://bmjopen.bmj.com/site/about/guidelines.xhtml

	proven or suspected colorectal cancer referred for staging.<br>• Suspicion of colorectal cancer defined as: Presence of a mass highly suspicious for colorectal cancer on endoscopy, barium enema, CT colonography or other imaging which triggers staging investigations.<br>• Patient must have given written informed consent and be willing to comply with the protocol intervention and follow up.<br>**Exclusion criteria:**<br>• Any psychiatric or other disorder likely to impact on informed consent<br>• Evidence of severe or uncontrolled systemic disease which make it undesirable for the patient to participate in the trial<br>• Pregnancy<br>• Contraindications to MRI (e.g. cardiac pacemaker, severe claustrophobia, inability to lie flat)<br>• Polyp cancer (because metastatic disease in these patients is vanishingly rare)
**Planned number of sites:**	14 (including 7 Imaging hubs)
**Target Country**	UK
**Trial Procedure:**	All patients will undergo a whole body MRI protocol in addition to the standard staging protocol employed at their institution.
**Anticipated duration of recruitment:**	4 years
**Duration of patient follow up:**	12 months
**Definition of end of trial:**	12 months after enrolment of the final patient.
**Other related research:**	Sub-study of WB-MRI generalisability

For peer review only

For peer review only - http://bmjopen.bmj.com/site/about/guidelines.xhtml

1.2. Trial Schema

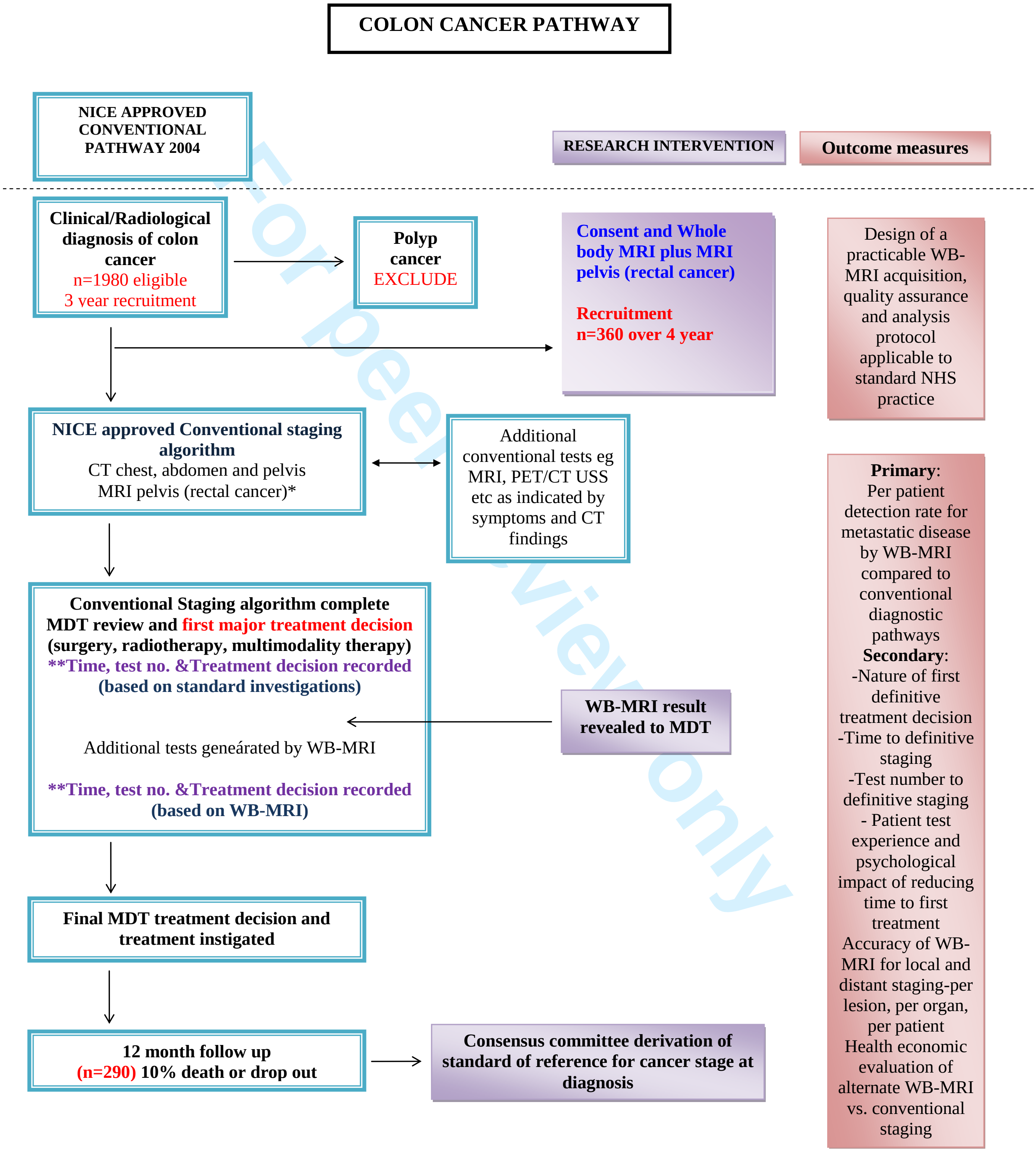

For peer review only - http://bmjopen.bmj.com/site/about/guidelines.xhtml

2. Introduction

2.1. Background

Colorectal cancer is the third most common malignancy in the UK and accounts for 13% of all new cancer diagnosis; approximately 40,000 patients are diagnosed per year [1]. Treatment decisions for colorectal cancer are critically dependent upon rapid and accurate tumour staging at diagnosis. In particular detection of metastatic disease (i.e. disease which has disseminated beyond the primary tumour into distant organ sites such as bone, liver and brain) is vital to optimised therapeutic triage. More aggressive approaches to treating metastatic colorectal cancer using combined chemotherapy and surgical resection regimens are widely adopted in routine National Health Service (NHS) practice [2].

Success rates however depend on the accuracy of initial disease staging so all disease sites are appropriately identified and treated [3]. Currently around 80% of patients with colorectal cancer undergo primary surgery with curative intent, although up to 50% subsequently relapse with metastatic disease, often within 12 months [4], in part reflecting sub-optimal disease staging. Accurate local staging of the tumour and spread to lymph nodes is also of importance. Imaging derived local staging in rectal cancer dictates the use of pre-operative (neo-adjuvant) chemotherapy. Furthermore, trials investigating neoadjuvant chemotherapy for non-rectal colonic tumours are currently recruiting in the NHS (FOXTROT).

Staging of colorectal cancer within the NHS is reliant on high technology imaging platforms such as computerised tomography (CT), positron emission tomography (PET) and magnetic resonance imaging (MRI), together with standard scintigraphy, plain X-rays and ultrasound. Staging pathways are complex (see flowchart, section 1.2), and governed by National Institute for Clinical Excellence (NICE) approved guidelines [5] which delineate an integrated step by step deployment of the various imaging modalities before tumour staging is deemed complete and the first treatment decision is made.

The complexity of current staging algorithms reflects the differing diagnostic attributes of the various imaging modalities across tumour types and body organs. CT for example is superior to ultrasound for detecting liver metastasis [6], but inferior to scintigraphy in diagnosing malignant spread to the bone. PET scanning is in general superior to scintigraphy for detecting bone metastases [7], but not as effective as MRI in identifying spread to the brain [8]. It is not unusual for a patient with colorectal cancer to undergo CT chest abdomen and pelvis, together with pelvic MRI in the case of rectal cancer. However additional tests such as ultrasound, MRI and PET-CT are increasingly used in cases of known or suspected metastatic disease. Such a stepwise multi-modality approach is time and resource intensive and onerous for patients at a very difficult time following their cancer diagnosis. Furthermore patients receive significant doses of diagnostic ionising radiation during the staging process; a single PET-CT imparts 6-10 times normal annual background radiation and even small doses of radiation can increase an individual’s risk of subsequent malignancy [9].

Recent technological advances mean an MRI scan of the whole body can be acquired in less than one hour; sensitivity for metastatic cancer spread is high [10]. MRI does

For peer review only - http://bmjopen.bmj.com/site/about/guidelines.xhtml

not impart ionising radiation and is advocated as a safe and effective alternative to the current multi-modality approach. There is no available secondary research evidence concerning the accuracy of whole body MRI (WB-MRI) in cancer staging. Indeed meta-analysis would be challenging because literature is limited to relatively small cohort studies comparing WB-MRI with conventional imaging tests, and in a wide variety of cancers. These do however suggest potential as an efficient and accurate “one stop shop” alternative for cancer staging [8, 11, 12]. For example, in 123 patients with non-small cell cancer, Yi found no difference between PET-CT and WB-MRI, with both achieving 86% accuracy [12]. Notably, the vast majority of available studies are single site, using one MRI platform, with interpretation by one or two experienced radiologists.

The literature is deficient regarding generalisability of WB-MRI. For example, variability amongst interpreting radiologists is unknown and the effect (if any) of different manufacturers’ platforms is uncertain. It is also unclear how WB-MRI can be best integrated into existing NHS pathways, which frequently differ between institutions, or how effective it would be in different clinical settings. By undertaking a multicentre trial of colorectal cancer using a variety of MRI platforms and interpreting radiologists, the current proposal will address these unknown variables.

Health psychology assessment

WB-MRI staging of colorectal cancer could reduce the physical and psychological burden associated with current pathways. By reducing time from diagnosis to treatment, WB-MRI may reduce patient anxiety, minimising concerns that treatment delay might adversely affect prognosis. Delay in diagnosis is known to be distressing, particularly for women [13]. In addition, staging scans can be uncomfortable and embarrassing [14]. MRI for example, while avoiding radiation, is noisy and claustrophobic. The potential of WB-MRI to generate additional tests is also an important consideration, although recent data suggest test sensitivity is the key determinant of patient choice and influences preference more than discomfort and complications [15]. While rapid staging means patients without metastasis are in receipt of good news more quickly, it also means those with metastasis will also receive bad news earlier, which may impact adversely on psychological wellbeing: It is known that patients are more depressed following a speedier diagnosis of breast cancer (at a one-stop clinic) when compared to those waiting a week [16].

Cost Effectiveness Analysis

Although WB-MRI is a relatively expensive imaging technology, other standard tests in the staging pathways such as PET-CT are also resource intensive. A detailed analysis of the cost and the cost-effectiveness of WB-MRI versus standard NICE-approved staging algorithms conforming to accepted economic evaluation methods [17] is essential to any trial investigating the role of WB-MRI in cancer staging.

For peer review only - http://bmjopen.bmj.com/site/about/guidelines.xhtml

3. Trial Overview

This trial will be an independent multi-centre prospective cohort trial comparing the staging accuracy of early WB-MRI with standard pathways in newly diagnosed colorectal cancer. We will follow the recommendations of the STAndards for the Reporting of Diagnostic accuracy studies (STARD) initiative [18]. A key element of our design is the investigation of early WB-MRI as a replacement test for standard pathways [19], and data regarding the therapeutic impact of WB-MRI will be collated, as well as addressing the practicalities of general NHS implementation. The trial design, which compares two different staging strategies in the same patients ensures that clean, comparable data are collected for both pathways while simultaneously increasing trial power to meet trial endpoints.

In addition, both qualitative and quantitative assessments will be employed to determine the psychological burden and acceptability of WB-MRI versus standard pathways, and to identify those attributes which most strongly influence patient preference. Health-related quality of life data will be collected which will be used to inform cost-effectiveness modelling, and determine whether patients believe more rapid staging would have helped them cope better with their cancer diagnosis.

3.1. Primary objective

To evaluate whether early whole body magnetic resonance Imaging (WB-MRI) increases per patient sensitivity for metastasis in colorectal cancer compared to standard NICE-approved diagnostic pathways.

3.2. Secondary objectives

1) To determine how WB-MRI influences time to and nature of first major treatment decision following definitive staging compared to standard investigations and to determine whether early WB-MRI could reduce or replace standard investigations.
2) To assess the accuracy of WB-MRI and standard diagnostic pathways for local and distant staging by comparison with an expert derived consensus reference standard using 1-year patient follow up data.
3) To evaluate the lifetime incremental cost and cost-effectiveness of staging using WB-MRI compared to standard diagnostic pathways.
4) To evaluate patients' experiences of staging using WB-MRI and to determine the priorities placed by patients on differing attributes related to competing staging pathways, including impact of reducing time to first treatment.
5) To determine the effect of radiologist confidence on the diagnostic accuracy of WB-MRI for metastatic disease.
6) To evaluate the diagnostic performance of limited WB-MRI protocols based on diffusion and T1 weighted imaging only, and to assess the incremental benefit of intravenous gadolinium contrast enhancement.

Additional objectives which will be met by the trial will be the definition of an efficient WB-MRI acquisition and analysis protocol, and quality assurance (QA) program

For peer review only - http://bmjopen.bmj.com/site/about/guidelines.xhtml

applicable to standard NHS practice and collation of a database of WB-MRI datasets for NHS staff training and service development.

3.3. Trial activation

UCL CTC will ensure that all trial documentation has been reviewed and approved by all relevant bodies and that the following have been obtained prior to activating the trial:

- Research Ethics Committee approval
- ‘Adoption’ into NIHR portfolio
- NHS permissions
- Adequate funding for central coordination
- Confirmation of sponsorship
- Adequate insurance provision

For peer review only

For peer review only - http://bmjopen.bmj.com/site/about/guidelines.xhtml

4. Selection of Sites/Site Investigators

4.1. Site selection

In this protocol a 'recruitment site' refers to a hospital where certain trial-related activities are conducted.

Recruitment sites must be able to comply with:

- Patient recruitment, follow up schedules and all requirements of the trial protocol
- Requirements of the Research Governance Framework
- Data collection requirements
- **No** trial imaging, recruitment sites will refer patients to designated imaging hub (see below)

In this protocol an 'imaging hub' refers to a hospital where certain trial-related activities are conducted.

Imaging hubs must be able to comply with:

- Patient recruitment (where applicable), follow up schedules (where applicable) and all requirements of the trial protocol
- Requirements of the Research Governance Framework
- Data collection requirements
- Trial imaging

4.1.1. Selection of Principal Investigator and other investigators at sites

Sites must have an appropriate Principal Investigator (PI), i.e. a health care professional authorised by the site and ethics committee, to lead and coordinate the work of the trial on behalf of the site. Other investigators at site wishing to participate in the trial must be trained and approved by the PI. All investigators must be medical doctors and have experience of diagnosing, staging or treating colorectal cancer.

4.1.2. Training requirements for site staff

All site staff must be appropriately qualified by education, training and experience to perform the trial related duties allocated to them, which must be recorded on the site delegation log.

CVs for all staff must be kept up to date, with signed and dated and copies held in the Investigator Site File (ISF). An up to date, signed copy of the CV for the PI must be forwarded to UCL CTC upon request.

All staff involved in the trial must receive GCP training which is relevant to their role and responsibilities within the trial. The frequency of repeat training may be dictated by the requirements of their employing institution, or 2 yearly where the institution has no policy, and more frequently when there have been updates to the legal or regulatory requirements for the conduct of clinical trials.

For peer review only - http://bmjopen.bmj.com/site/about/guidelines.xhtml

4.2. Site initiation and activation

4.2.1. Site initiation

Before a site is activated, the UCL CTC trial team will arrange a site initiation with the site which the PI and site research team must attend. The site will be trained in the day to day management of the trial, and essential documentation required for the trial will be checked.

Site initiation will be performed for each site by site visit or teleconference.

4.2.2. Required documentation

The following documentation must be submitted by the site to UCL CTC prior to a site being activated by UCL CTC trial team:

- Trial specific Declaration of Participation/Site Registration Form (identifying relevant local staff)
- All relevant institutional approvals (e.g. local NHS permission)
- A completed site delegation log that is initialled and dated by the PI
- A copy of the PI's current CV that is signed and dated

In addition, the following agreements must be in place:

- A signed Clinical Trial Site Agreement (CTSA) between the Sponsor and the relevant institution (usually a NHS Trust)

4.2.3. Site activation letter

Once the UCL CTC trial team has received all required documentation and the site has been initiated, a site activation letter will be issued to the PI. **Sites may not begin to approach patients until the site activation letter has been issued.**

Once the site has been activated by UCL CTC, the PI is responsible for ensuring:

- adherence to the most recent version of the protocol;
- all relevant site staff are trained in the protocol requirements;
- appropriate recruitment and medical care of patients in the trial;
- timely completion and return of CRFs (including assessment of all adverse events).

For peer review only

For peer review only - http://bmjopen.bmj.com/site/about/guidelines.xhtml

Streamline C

5. Informed consent

Sites are responsible for assessing a patient's capacity to give informed consent.

Sites must ensure that all patients have been given the current approved version of the patient information sheet, are fully informed about the trial and have confirmed their willingness to take part in the trial by signing the current approved consent form.

Sites must assess a patient's ability to understand verbal and written information in English and whether or not an interpreter would be required to ensure fully informed consent. If a patient requires an interpreter and none is available, the patient should not be considered for the trial.

The PI, or, where delegated by the PI, other appropriately trained site staff, are required to provide a full explanation of the trial to each patient prior to trial entry. During these discussions, the current approved patient information sheet for the trial should be discussed with the patient. A minimum of twenty four hours before the WB-MRI must be allowed for the patient to consider and discuss participation in the trial.

The consent process has been left deliberately flexible in order to accommodate the needs of individual patients and variations in Site requirements. The flowchart below describes three different options for obtaining written informed consent from patients, each of which ensures that patients have at least 24 hours to consider their participation in the trial before any trial related activities take place.

Streamline Recruitment Pathways/Consent Process

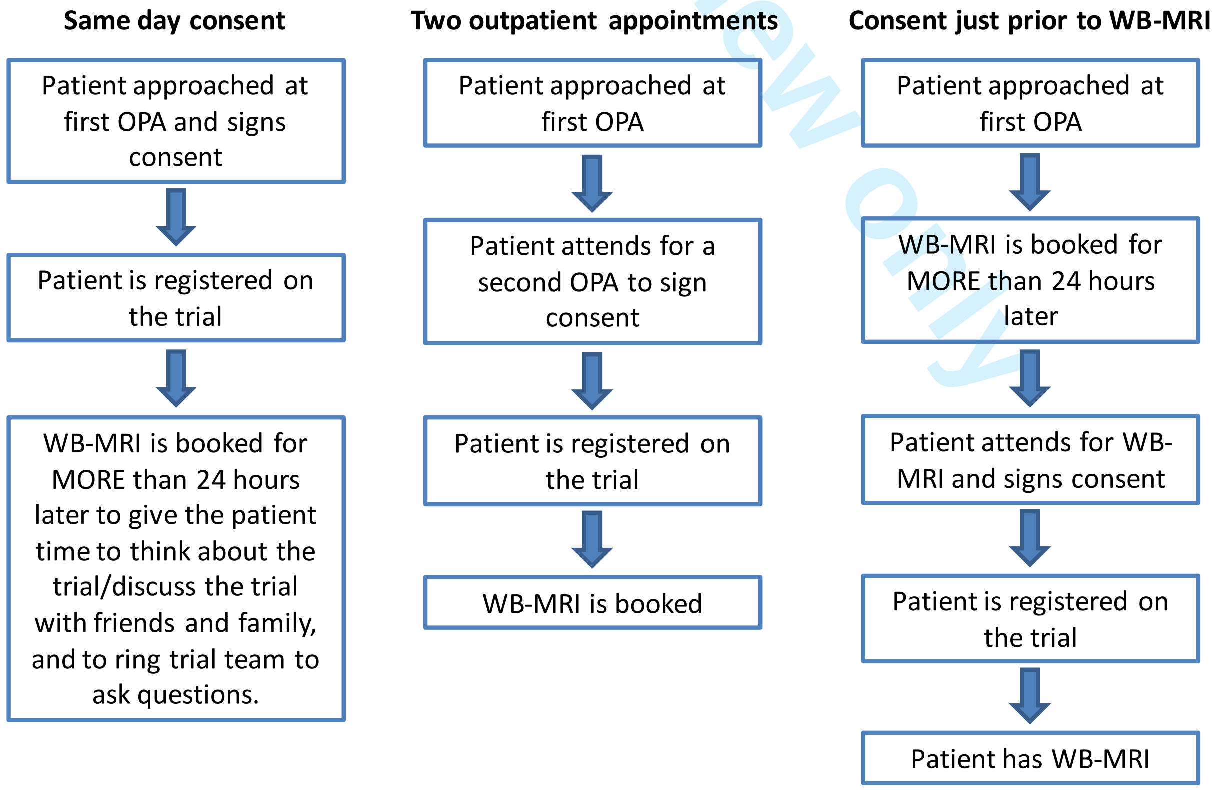

For peer review only - http://bmjopen.bmj.com/site/about/guidelines.xhtml

See Appendix 2 for a more detailed example of the consent process.

Written informed consent on the current approved version of the consent form for the trial must be obtained before any trial-specific procedures are conducted. The discussion and consent process must be documented in the patient notes.

Site staff are responsible for:

- checking that the correct (current approved) version of the patient information sheet and consent form are used;
- checking that information on the consent form is complete and legible;
- checking that the patient has completed/initialled all relevant sections and signed and dated the form;
- checking that an appropriate member of staff has countersigned and dated the consent form to confirm that they provided information to the patient;
- checking that an appropriate member of staff has made dated entries in the patient's medical notes relating to the informed consent process (i.e. information given, consent signed etc.);
- Following registration:
    - adding the patient trial number to all copies of the consent form, which should be filed in the patient's medical notes and investigator site file.
    - giving the patient a copy of their signed consent form and patient information sheet.

The right of the patient to refuse to participate in the trial without giving reasons must be respected. All patients are free to withdraw at any time. Also refer to section 13.0 (Withdrawal of patients).

For peer review only - http://bmjopen.bmj.com/site/about/guidelines.xhtml

6. Selection of Patients

6.1. Pre-registration Evaluation

The following assessments or procedures are required to evaluate the suitability of patients for the trial:

- Suspicion of colorectal cancer defined as: Presence of a mass highly suspicious for colorectal cancer on endoscopy, barium enema, CT colonography or other imaging which triggers staging investigations
- No contraindications to MRI (e.g. cardiac pacemaker, severe claustrophobia, inability to lie flat)

6.2. Screening Log

A screening log must be maintained by the site and kept in the Investigator Site File. This must record all potentially eligible patients approached about the trial and the reasons why they were not registered in the trial if this is the case. The log must be sent to UCL CTC in line with the monitoring plan and as requested, with patient identifiers removed prior to sending.

6.3. Multi Disciplinary Team (MDT) Meeting Log

An anonymous record of the number of patients with a new diagnosis of colorectal cancer discussed in the MDT meeting, with their attributed cancer stage (TNM or Dukes) will be submitted annually to UCL CTC. This will be used to assess whether the group of patients recruited to the trial is representative of the general population of newly diagnosed colorectal cancer patients seen at Sites.

6.4. Patient Eligibility

There will be no exception to the eligibility requirements at the time of registration. Queries in relation to the eligibility criteria must be addressed prior to calling/faxing for registration. Patients are eligible for the trial if all the inclusion criteria are met and none of the exclusion criteria applies.

6.4.1. Patient Inclusion Criteria

- Adult patients (18 or over) with histologically proven or suspected colorectal cancer referred for staging.
- Suspicion of colorectal cancer defined as: Presence of a mass highly suspicious for colorectal cancer on endoscopy, barium enema, CT colonography or other imaging which triggers staging investigations.
- Patient must have given written informed consent and be willing to comply with the protocol intervention and follow up.

For peer review only

For peer review only - http://bmjopen.bmj.com/site/about/guidelines.xhtml

6.4.2. Exclusion Criteria

- Any psychiatric or other disorder likely to impact on informed consent.
- Evidence of severe or uncontrolled systemic disease which make it undesirable for the patient to participate in the trial.
- Pregnancy.
- Contraindications to MRI (e.g. cardiac pacemaker, severe claustrophobia, inability to lie flat).
- Polyp cancer (because metastatic disease in these patients is vanishingly rare).

6.4.3. Pregnancy and Birth Control

MRI poses a theoretical risk to the foetus, particularly in the first trimester due to local acoustic and heating effects. However the risk is generally deemed very small and significantly less than the risk of ionising radiation imparted by PET-CT and CT (standard imaging investigations).

A woman of childbearing potential (WCBP) is a sexually mature woman (i.e. any female who has ever experienced menstrual bleeding) and who has not undergone a hysterectomy or who has not been postmenopausal for 24 consecutive months (i.e. who has had menses at any time in the preceding 24 consecutive months). Patients will be excluded if they are pregnant.

There is no requirement for additional contraceptive advice to patients over and above that routinely given as part of their routine clinical care given their diagnoses of colorectal cancer.

The need to perform a pregnancy test in WCBP will be decided as part of the patient's routine clinical care given the risk to pregnancy from standard staging investigations and subsequent treatment. There is no requirement to perform a pregnancy test purely because of recruitment to the trial if this would not have been performed as part of standard clinical care.

For peer review only - http://bmjopen.bmj.com/site/about/guidelines.xhtml

7. Registration Procedures

7.1. Registration

Patient registration will be undertaken centrally at UCL CTC and this must be performed prior to commencement of any trial intervention. Registration can be performed via telephone or fax.

Following pre-registration evaluations (as detailed in section 6.1), confirmation of eligibility and consent of a patient at a site, the registration form must be fully completed prior to faxing or telephoning UCL CTC. The eligibility criteria will be reviewed during the registration telephone call using the same form at UCL CTC.

A trial number will be assigned for the patient during the call and must be recorded at site by the caller. If desired, the site may fax a copy of the completed registration form to UCL CTC and the faxed registration from will be used it confirm patient eligibility at UCL CTC.

UCL CTC will email confirmation of the patient's inclusion in the trial and their trial number to the main contact, PI and Imaging Hub contact. If requested, a fax confirmation may also be sent. Case report forms will be emailed to the main contact at site.

Registration telephone number:	+44 (0)20 7679 9880
Registration fax number:	+44 (0)20 7679 9871
UCL CTC Office hours:	09:00 to 17:00 Monday to Friday

7.2. Registration Packs

Once a patient has consented to take part in the trial they should be provided with a registration pack to include the following:

- A copy of their signed consent form and patient information sheet
- An expenses claim form (to claim travel expenses to and from WB-MRI appointment)
- Pre-paid return envelope

7.3. Booking Whole Body MRI

Once a patient has been registered it is the responsibility of the recruiting site to contact their designated imaging hub to make arrangements for the WB-MRI. Each recruitment site will be provided with a contact number at their designated imaging hub. Recruitment sites will approach consecutive potentially eligible patients until the number of recruited patients fills the MRI capacity of the imaging hub.

For peer review only - http://bmjopen.bmj.com/site/about/guidelines.xhtml

It is possible recruitment potential at recruitment sites will be greater than the WB-MRI capacity at each central imaging hub. If a patient is approached and registered for the trial but MRI capacity is such that the scan could not take place in a timely fashion, a decision to withdraw the patient will be made by the recruitment site and the patient will then be informed. This possibility is explained to patients in the patient information sheet.

Because of the need to perform WB-MRI in a timely fashion, it is anticipated recruitment sites will be requested to pause recruitment if WB-MRI waiting time exceeds 2 weeks (if WB-MRI is performed after conventional imaging is complete) or 3 weeks (if WB-MRI is to be performed concurrently with standard tests). There will be flexibility in these timings depending on the circumstances of individual patients and their anticipated time to complete standard staging.

For peer review only

For peer review only - http://bmjopen.bmj.com/site/about/guidelines.xhtml

8. Patient Management and Trial Procedures

8.1. Summary

8.1.1. Staging and Treatment Decisions

The following information is required for the trial to document staging and treatment decisions:

- Stage and treatment decision based on conventional imaging only (and the number, timing nature and findings of these investigations)
- Stage and theoretical treatment decision based on WB-MRI only (and any additional tests generated by the WB-MRI) only (and the number, timing nature and findings of additional tests generated)
- A final treatment decision incorporating all available tests.

Conventional Imaging

Trial patients will undergo the standard staging protocol employed at their institution as per usual clinical care pathways. See section 8.2.1 for further detail.

Whole Body MRI Imaging and MDT Reveal

Recruited patients will undergo a WB-MRI at one of the designated imaging hubs. Recruiting sites should contact their designated imaging hub to request a WB-MRI slot (see section 7.3). The WB-MRI should be performed either concurrently with the standard staging investigations, or no later than 3 weeks after the final standard staging investigation. Images will be uploaded at each hub to a secure central imaging server called 3Dnet™. If a language interpreter was required for a patient during the consent process, then an interpreter should be made available to the patient during the WB-MRI. The WB-MRI will be reported by designated radiologist(s) at each imaging hub who are blinded to the conventional imaging tests and other clinical information (other than the cancer diagnosis and location). A copy of the report will also be uploaded to 3Dnet™. See section 8.2.2 for further detail.

Once the MDT has made a treatment decision based on the conventional imaging, the MDT coordinator, research nurse or other designated individual will reveal the WB-MRI images and report on 3Dnet™ via an internet-enabled PC. The MDT will review the WB-MRI results and decide whether or not any additional tests are required or would have theoretically been required (if in fact the test was already performed as part of conventional staging) in addition to the WB-MRI. If any tests are required that have not already been performed as part of the standard staging, they should be performed and the patient reviewed again at the next MDT when the results are available. If no further tests are needed then the stage and theoretical treatment decision based on the WB-MRI (and any additional tests generated from the WB-MRI) must then be made by the MDT. The MDT must then decide a final treatment decision for the patient based on all imaging available. If referral to the liver MDT is indicated, the final treatment decision based on WB-MRI will be that made by the liver MDT after review of the WB-MRI and generated tests. See section 8.2.3 for further detail.

For peer review only - http://bmjopen.bmj.com/site/about/guidelines.xhtml

If a patient is due to start treatment before the next formal MDT meeting, an ad hoc mini MDT meeting may be scheduled outside of the standard MDT meeting. This should include the relevant multidisciplinary clinical team members who are appropriate to make the final treatment decision based on the specific imaging finding, according to usual standards of clinical care. A record will be kept of who is present during the ad hoc mini MDTs on the CRFs.

8.1.2. Patient experience Interviews and Questionnaires (Optional)

Interviews (Study 1)

A total of 25 patients have undergone interviews by a Health Psychologist pertaining to their experience of the cancer staging process. **Target accrual has now been reached for the interview part of the trial and recruitment to this is now closed.**

Questionnaires (Study 2)

Patient experience questionnaires from baseline and post-staging have been received for approximately 50 patients to assess patient experience and acceptability of WB-MRI and standard tests. **Target accrual has now been reached for the questionnaire part of the trial and recruitment to this is now closed.**

Discrete choice experiment (Study 3)

A discrete choice experiment (DCE) questionnaire will be administered to a subset of approximately 50 patients to elicit their preference for various attributes associated with conventional and WB-MRI staging pathways. The DCE questionnaire is optional and patients will be asked to consent to this on trial entry. Once a patient consents to the DCE they will be allocated a DCE questionnaire (out of a possible 2 DCE questionnaires) which will be posted by UCL CTC along with their Quality of Life questionnaire, patient diary and pre-paid envelope immediately after registration (baseline). UCL CTC will supply the details of any recruited patients who consented to complete DCE questionnaire to the Health psychologists who may contact them directly.

8.1.3. Quality of Life Questionnaires and Patient Diaries

Upon consent the recruiting site must inform the patient that they will receive an EQ-5D Quality of Life Questionnaire and Patient Diary by post from UCL CTC and they are to complete these and return them using the pre-paid envelope provided. The patient diary will cover a 3 month period and will collect information regarding the patient's Primary Health Care contacts, other health care contacts and also the medication they are currently taking. Patient diaries and EQ-5D questionnaires will be posted to all patients at consent and then at 3 monthly intervals for 12 months.

For peer review only - http://bmjopen.bmj.com/site/about/guidelines.xhtml

Streamline C

Table 1: Summary of patient management and trial activities

Activity	Protocol section	Timing							Responsible organisation/person
		Prior to registration	Immediately after registration	During staging	After staging (months after registration)				
					1	3	6	9	
Pre-registration evaluation & assessment of eligibility	6.1	X							Recruiting site
Consent	5.0	X							Recruiting site
Register patient	7.1	X							Recruiting site
Give patient registration pack (copy of PIS and consent, expenses claim form and pre-paid return envelope)	7.2		X						Recruiting site
Book WB-MRI scan & inform patient of appointment date	7.3		X						Recruiting site
WB-MRI	8.2.2			X					Imaging Hub
Upload WB-MRI scan & report	8.2.2			X					Imaging Hub
Post EQ-5D questionnaires	8.2.8		X			X	X	X	UCL CTC
Post patient resource diary	8.2.8		X			X	X	X	UCL CTC
Discrete choice experiment Questionnaire	8.2.10		X						UCL CTC

For peer review only

For peer review only - http://bmjopen.bmj.com/site/about/guidelines.xhtml

8.2. Detailed description

8.2.1. Standard care

Trial patients will undergo the conventional staging investigations employed at their recruiting institution according to local protocols and the requirements of their clinical care team.

All conventional investigations will be performed and interpreted by the usual radiologists and clinicians employed at the site of the investigations. Standard clinical reports will be made and all investigations (and their results) will be freely available on hospital Picture Archiving and Communications System (PACS), Radiology Information System (RIS) and Clinical Data Repository (CDR) systems as per usual clinical practice.

The type and date of investigations (e.g. CT scan, PET-CT, organ specific MRI, biopsy etc) will be recorded on CRFs, along with the presence and location of metastatic disease based on the radiological report.

8.2.2. Trial Imaging

Whole Body MRI (WB-MRI) protocol

Recruited patients will undergo a WB-MRI at their designated imaging hub site. The choice of MRI platform (i.e. manufacturer and Tesla (T) strength) will be decided by the local hub radiologist according to scanner availability and their usual practice. It is anticipated most MRIs will be performed at 1.5T. Exact imaging parameters will vary according to MRI platform but a minimum dataset of sequences will be acquired (full details given in appendix 3).

Patients with rectal cancer would normally undergo staging pelvic MRI, and the WB-MRI will be additional to this. Where possible (and according to site and patient preference), the WB-MRI and rectal staging MRI would be performed on the same day to reduce patient travel.

Timing

The WB-MRI will be performed either concurrently with the standard staging investigations, or no later than 3 weeks after their final standard staging investigation.

Blinding

To ensure the integrity of the trial, the WB-MRI must be reported by a radiologist blinded to the conventional imaging tests and other clinical information (other than the cancer diagnosis and site). Conversely the WB-MRI images and reports must not be available to either those radiologists reporting the standard staging investigations, nor those involved in direct patient care before the WB-MRI is revealed in the MDT meeting.

The un-anonymised WB-MRI images will therefore not be immediately sent to the Picture Archive and Communication System (PACS) at either the imaging hub or recruitment sites. Instead images will be uploaded at each hub to a secure central

For peer review only - http://bmjopen.bmj.com/site/about/guidelines.xhtml

imaging server (3Dnet™) provided by Biotronics3D. This solution allows easy upload of MRI datasets via standard internet connection. A PC based internet gateway will be installed in each imaging hub to facilitate automated transfer of WB-MRI from the scanner/workstation to 3Dnet™), and thereafter back to PACS to the appropriate time point after MDT revelation (see below).

Interpretation and reporting

The WB-MRI will be interpreted by designated radiologist(s) at each imaging hub who are expert in interpretation based on previous experience of reporting WB-MRI in cancer staging. As noted above, the radiologist will be blinded to the standard staging investigations performed on recruited patients.

Interpretation can be performed using the 3Dnet™ software, or stand alone workstation according to the preference of the radiologist.

Images will be analysed in the following order:

1) Diffusion and non contrast enhanced T1 images
2) Diffusion, non contrast enhanced T1 and T2 images
3) Diffusion, non contrast enhanced T1, T2 and contrast enhanced T1 images.

After viewing each sequence set (and before reviewing the next set), the radiologist will complete WB-MRI Imaging Booklet CRFs documenting the presence, location and size of metastatic disease, together with their diagnostic confidence on a scale of 1 to 6. Reporting time for each sequence set analysis (defined as the time required to interrogate the sequences to reach a diagnostic conclusion) will be recorded along with the technical quality of the MRI dataset.

The reporting radiologist will then produce a free text clinical report as per their usual clinical practice (using all available sequences and based on the TMN 7 staging guidance) for subsequent release to the clinical team. This report will contain information relating to the local T and N stage of the tumour, together with the presence, location, number and size of metastatic deposits, as well as important "incidental" findings, for example aortic aneurysm. The radiologist may express their level of confidence in reported findings as they would in normal clinical practice but a formal numerical score of confidence will not be provided as this would not mirror how radiological examinations are reported in standard clinical care. If the radiologist would usually recommend additional tests for equivocal findings, this will be included in the report so as to also mirror routine clinical practice. The definitions of T, N M stage will be based on conventional MRI criteria adopted by the radiologist in their usual clinical practice.

The free text report will be uploaded onto the 3Dnet™ software, and a copy stored as part of the patient's medical record.

For peer review only - http://bmjopen.bmj.com/site/about/guidelines.xhtml

8.2.3. Release of Conventional and trial WB-MRI findings

MDT discussion of the first major treatment decision based on conventional imaging

WB-MRI images and reports will be withheld initially from the clinical care team and radiologists reporting standard imaging (to avoid bias) until patients have completed all conventional investigations and have been definitively staged such that a first major treatment decision has been made by the MDT based on these conventional tests. The first major treatment decision based on conventional imaging will be defined as:

- Referral for surgical excision of either the primary tumour and/or a metastatic site
- Instigation of definitive treatment using chemotherapy, radiotherapy or a combination of the two
- Decision to offer palliative/supportive care only
- Request for a highly invasive surgical staging procedure such as surgical mediastinal lymph node sampling (mediastinoscopy), video-assisted thoracoscopic surgery (VATS), or laparoscopy

In some instances the first major treatment decision will not be made by the colorectal MDT, for example the patient being referred to a liver MDT. In this case the treatment decisions of the liver MDT made according to the protocols described below will be used for the purposes of the trial.

If additional conventional tests are required to complete staging, the WB-MRI result will be withheld until they are completed, and the patient re-discussed with the findings of the additional test(s).

The nature, date and findings of all conventional investigations will be recorded by the MDT, and the TNM 7 stage recorded. The MDT/appropriate MDT personnel will define the nature of the first treatment decision based on conventional investigations in the MDT record and on the CRF.

Release of WB-MRI images and report

Once a stage and treatment decision has been made based on conventional imaging, the WB-MRI images (supplemented where possible with "screen shots" and annotations of relevant findings) and radiologist's report will be revealed to the MDT using the 3Dnet™ software on an internet enabled PC. If technical issues preclude use of 3Dnet™ in the MDT, presentation of a written copy of the WB-MRI report is permissible.

When the WB-MRI images and report are released, the MDT will discuss the images and decide:

1) *If the patient can be adequately staged based on WB-MRI alone:*

    In this case the MDT should state the patient's stage and theoretical treatment decision based on the WB-MRI alone.

Or:

For peer review only - http://bmjopen.bmj.com/site/about/guidelines.xhtml

For peer review only

2) *If any additional tests are required based on the WB-MRI alone for adequate staging* (for example for equivocal WB-MRI findings):
    a. For additional tests that would have theoretically been requested based on the WB-MRI alone but have already been performed as part of the standard imaging, the results can be used by the MDT. The MDT personnel will state the patient's stage and theoretical treatment decision based on the WB-MRI and those additional tests that would have theoretically been generated.
    b. If the additional tests generated by WB-MRI have not already been performed as part of standard care and are deemed necessary in the opinion of the MDT these will be undertaken according to standard clinical care. After completion of the additional test(s), the patient will be re-discussed by the MDT and a final treatment decision made based on the WB-MRI and the additional test(s) undertaken.

If, following revelation of the findings of the WB-MRI (and any additional tests it generates), the colorectal MDT request review by the liver MDT, (which had not been the case based on standard imaging), for the purposes of the trial the final treatment decision based on WB-MRI will be that made by the liver MDT after review of the WB-MRI and generated tests. The fact that following WB-MRI a referral to the liver MDT is made will be recorded.

8.2.4. Final treatment plan

Once a stage and treatment decision has been made based on conventional imaging alone and also a stage and theoretical treatment decision made based on the WB-MRI (and any additional tests it generates) alone a final treatment plan can be made by the MDT. The final treatment plan for the patient will be based on all available information (including the WB-MRI). The MDT radiologist(s) will be at liberty to review the WB-MRI data after the WB-MRI has been revealed in order to review its findings and feedback to the clinical care team.

The completed MDT CRF will thus list:

- Stage and treatment decision based on conventional investigations (and the number, timing nature and findings of these investigations).
- Stage and theoretical treatment decision based on WB-MRI (and the number, timing nature and findings of additional tests generated).
- Final treatment decision incorporating all available tests.

Once the WB-MRI images and report have been revealed to the MDT according to trial protocol, they can be released onto the relevant hospital PACS server using the 3Dnet™ gateway described above or other IT solution (e.g. image exchange portal) and be made freely available to those providing subsequent patient care, as per usual clinical practice.

For peer review only

For peer review only - http://bmjopen.bmj.com/site/about/guidelines.xhtml

8.2.5. Early release of WB-MRI findings

Any requests for early WB-MRI release must be made to the site PI, who will discuss the request with the lead clinician who is responsible for the patient's clinical care (if the request did not originate from the lead clinician). After discussion if it is decided by the PI, that release of the WB-MRI images and report is necessary, the PI (or a designated member of the trial team) will inform UCL CTC. The WB-MRI result should only be released early in the event of an emergency situation in which the patient is unfit to undergo additional investigations (including MRI) which would have normally been performed were the patient not in the Streamline C trial, and the WB-MRI may have a direct effect on immediate patient care. In the event of early release, the patient will be replaced, although the findings of the WB-MRI and conventional staging (performed up to the time of early MRI release) will be collated by UCL CTC on the standard trial CRFs for subsequent reporting in the trial publications.

Early release for important clinical findings

The time between the patient's diagnosis and the WB-MRI reveal is typically less than 4 weeks at the proposed recruitment sites. The majority of findings on WB-MRI will have no direct impact on patient care during this time. However should WB-MRI reveal a serious finding which could have an immediate impact on direct patient care before the MDT, the reporting radiologist will contact the patient's clinician to discuss the finding and a decision will be made as to whether the results should be revealed early to all members of the clinical team (based on review of standard tests already performed which may also have detected the finding). Specific findings which will trigger this review are:

- Impending spinal cord compression
- Deep vein thrombosis or pulmonary embolism
- Brain metastasis with significant mass effect requiring immediate treatment

It is unlikely the patient would be asymptomatic in any of these scenarios, and it is probable additional imaging tests would have already been requested as part of usual clinical care.

Early release for urgent patient management

Although unlikely, it is possible there may be a need to access the WB-MRI report urgently before its release to the MDT, for example if a recruited patient presents to hospital with collapse and knowledge of the WB-MRI findings would potentially change patient management. As noted above, imaging hubs will keep a copy of the free text report of the WB-MRI and this will be made available if requested by clinical teams in this scenario.

8.2.6. Time to full staging

Standard pathway

The total time required to fully stage the patient using standard imaging pathways will be calculated. The start of the staging process will be defined as the date of request of the first staging investigation following a proven or assumed diagnosis of colorectal

For peer review only - http://bmjopen.bmj.com/site/about/guidelines.xhtml

cancer (for example date of requested for CT chest, abdomen and pelvis after colonoscopic diagnosis of likely malignant tumour). The completion of staging will be defined as the date of the MDT/appropriate MDT personnel made the first major treatment decision based on the standard imaging.

WB-MRI

Because revelation of the result of WB-MRI is deferred until after standard staging is complete, actual measurement of the time to full staging using WB-MRI will not be possible. The theoretical time to complete staging using WB-MRI will be thus modelled taking into consideration the time from recruitment date to the date of the WB-MRI, plus the number and type of additional staging investigations generated by WB-MRI.

8.2.7. Patient follow up

Patients will be followed for a period of 12 months from the date of recruitment. Follow up will not require any trial specific patient visits to the recruiting site, and follow up CRFs can be completed using hospital databases or patient notes.

For all recruited patients the final TN tumour stage, according to the 7th classification, will be recorded based on histopathological analysis of surgical specimens (if surgery is performed). The results of any biopsy procedure undertaken over the 12 months will also be recorded. The date, nature and findings of follow up imaging investigations will be recorded for each recruited patient, in particular the presence or absence of metastasis.

The date and cause of patient death and post mortem findings (if performed) will also be recorded.

MDT records and hospital data repositories will be used to collate this data, and data collection will be coordinated by designated individuals at each recruitment site, aided by UCL CTC.

8.2.8. Patient diaries and EQ-5D Quality of Life questionnaires

All patients will receive a Diary and EQ-5D Quality of Life questionnaire every 3 months which will be posted by UCL CTC. Upon consent the recruiting site must inform the patient that they will receive an EQ-5D questionnaire and Diary through the post from UCL CTC and they are to complete these and return them using the pre-paid envelope provided. The patient diary will cover a 3 month period and will collect information regarding the patient's Primary Health Care contacts, other health care contacts and also the medication they are currently taking. Patient diaries and EQ-5D questionnaires will be posted to all patients at consent and then at 3 monthly intervals for 12 months.

8.2.9. Final reference standard for tumour stage (Consensus Meetings)

Multi-disciplinary consensus panel review is standard methodology for diagnostic test accuracy studies where an independent reference standard is impossible because of incorporation bias. Consensus panels will convene annually to derive the reference standard for tumour stage at diagnosis for recruited patients completing the first 12 month follow up (i.e. a panel at the end of year 2 will consider patients recruited in

For peer review only - http://bmjopen.bmj.com/site/about/guidelines.xhtml

year 1, a panel at the end of year 3 will consider patient recruited in year 2 etc.). The panels will consider all available clinical information including the results of all original staging investigations, WB-MRI, histopathology (surgical resection and biopsies), follow up imaging and post-mortem reports (where available) and MDT records. UCL CTC will coordinate collation of these data via submitted CRFs over the preceding 12 months for presentation to the panel. Each imaging hub or recruitment site will host a consensus panel to consider patients undergoing WB-MRI (or recruited) at its site (so all imaging studies are available for review on local PACS systems if required). Each panel will consist of at least an oncologist, and/or a colorectal surgeon, and 3 radiologists, 1 external to the imaging hub and 2 internal: 1 with specific expertise in WB-MRI and 1 with expertise in PET (a single radiologist is acceptable if they have expertise in WB-MRI and PET). The panel will have access to a histopathologist if required. Each panel will adjudicate on the TMN stage of the cancer at diagnosis, including the organ specific sites and burden of metastatic spread.

The definition of the presence or absence of metastasis will be assigned for each organ on a designated CRF. The designation will be made in consensus by all on the panel. Consideration will be made to histology in all biopsied lesions. In the absence of histological proof of metastasis, metastatic disease will be assumed if new lesions appear during the 12 month follow up with imaging characteristics compatible with metastasis and no alternative explanation, or if lesions with characteristics compatible with metastasis which either grow or shrink (on therapy). Lesions identified which remain stable over the 12 month follow up period will assumed not to be metastatic unless there are specific circumstances considered by the panel that indicate malignancy (e.g. change in lesion morphology with treatment).

All "new" sites of metastatic disease diagnosed in the 12 month follow up will be assessed by the panel to see if they were visible in retrospect at diagnosis. Although the primary analysis by the original reporting radiologist will be that used to define test accuracy, this retrospective review will allow definition of the rates of perceptual error.

For patients in whom the primary tumour has been completely removed within 3 months of diagnosis, all new metastatic sites will be assumed to have been present at diagnosis for the purposes of calculating test sensitivity.

If the primary tumour is left in situ for more than 3 months (or there is incomplete removal), any new diagnosed sites of metastatic disease will be assumed to have been present at diagnosis if identified within 6 months of diagnosis of the primary tumour. If they are diagnosed beyond 6 months of diagnosis, and there is no evidence of their presence on retrospective review of all staging investigations, they will be assumed to be new disease not present at diagnosis.

If patients with tumours left in situ do not undergo any imaging capable of detecting metastatic disease within 6 months of diagnosis of the primary tumour (other than initial staging tests) and imaging beyond this identifies metastatic disease not visible in retrospect on any trial imaging, the consensus panel will opine if the disease was likely present at diagnosis, based on its location, size and imaging characteristics.

If a patient dies before the 12 month follow up, the panel will review all available imaging, histopathology and clinical course prior to death and in consensus state if a confident diagnosis of the presence or absence of metastatic disease can be made (for example imaging characteristics compatible with metastasis and no alternative

For peer review only - http://bmjopen.bmj.com/site/about/guidelines.xhtml

explanation, or if lesions with characteristics compatible with metastasis which either grow or shrink (on therapy). If this judgement cannot be made with confidence (for example if the patient has equivocal lesions on staging investigations and no further follow up), these patients will not be excluded but multiple imputation will be used to account for missing data. A sensitivity analysis for the primary outcome, where only patients with complete data are analysed, will exclude these patients.

8.2.10. Health psychology assessment

Study 1: Patient interviews (approximate n=25)

Target accrual has now been reached for the patient experience interview part of the trial and so recruitment to this part is now closed. Individual in-depth interviews were conducted by a health psychologist pertaining to their experience of the cancer staging process. The aim of the interviews is to determine patients' experience of WB-MRI, standard tests and those tests generated by WB-MRI. We collected simple demographic data from participants in relation to gender, age and educational level as such factors may influence experience and preferences, and this data will enable us to determine whether the sample of people who are interviewed are representative of the sample of people who also participate in studies 2 and 3 (see below). The interviews assessed which aspects of testing caused patients physical or psychological stress (e.g. number of tests/hospital visits, test attributes (i.e. physical experiences of the tests such as claustrophobia, need to lie still, scanner noise etc.), total length of time taken, additional tests generated by initial scan findings) and elicited any factors patients felt would have made staging easier for them.

The interviews provided in-depth qualitative data about the experience of cancer staging and in particular under-going WB-MRI, and has informed the modification of a questionnaire (study 2) designed to assess patient acceptability and experience of WB-MRI compared to standard tests, and also has informed the design of a discrete choice experiment (study 3).

Study 2: Patient questionnaires (approximate n=50)

Target accrual has now been reached for the patient experience questionnaire part of the trial and so recruitment to this part is now closed. Follow up questionnaires will continue to be posted until the last patient's month 6 time point occurs. A questionnaire was developed and sent to consenting patients to assess their experience and acceptability of WB-MRI and standard tests (e.g. PET-CT). Final design was informed by analysis of the qualitative data collected in study 1 (See above). Patient Experience Questionnaire content is outlined in Table 3 and includes patients' views about test preparation (e.g. fasting), and experience of the scan itself (e.g. discomfort, claustrophobia, fear etc.). Patients were also asked to rate the importance of various attributes associated with competing staging pathways (e.g. waiting time from diagnosis to treatment, total test number, test accuracy etc.). This rating identified those attributes considered most important by patients, which has been included in the discrete choice experiment (study 3).

Patients were given the opportunity to opt in to completing the questionnaires when they were recruited into the trial. Questionnaires, together with the positive and negative affect schedule (PANAS) and the General Health Questionnaire (GHQ-12) were administered during and after staging pathways, and at 3 and 6 months later to

For peer review only - http://bmjopen.bmj.com/site/about/guidelines.xhtml

examine patient test preferences once they are aware of their cancer stage and knew which of the tests they received were more accurate as well as the burden placed on them by any extra tests they needed as a result of WB-MRI.

Patients were posted the baseline questionnaire along with pre-paid reply envelope at the time they were recruited into the trial. The questionnaire was worded to take into account the fact that some patients had not yet had any staging investigations. Patients were posted the post-staging and 3 and 6 month follow up questionnaires along with pre-paid envelopes at the relevant time points.

Study 3: Discrete choice experiment (approximate n=50)

Discrete choice experiments (DCE) can elicit preferences for different types of health care provision [18] by estimating the relative importance of different attributes, and the trade-offs between them [19]. WB-MRI and conventional staging pathways differ in associated attributes, not only related to physical experience but also rate of adverse events, time to diagnosis, overall accuracy etc. The most important attributes have been identified in studies 1 and 2 and appropriate levels assigned to each based on accumulating data from the trial, together with appropriate literature review. A DCE questionnaire has been developed whereby patients state their preference between two choices, with each choice containing different levels of the identified attributes.

The DCE questionnaire is optional and patients will be asked to consent to complete the questionnaire on trial entry. Once a patient consents to the DCE they will be allocated a DCE questionnaire (out of a possible 2 DCE questionnaires) which will be posted by UCL CTC immediately after registration (baseline) along with their QoL questionnaire, patient diary and pre-paid envelope. UCL CTC will supply the details of any recruited patients which consented to complete the DCE questionnaire to the Health psychologists who may contact them directly.

For peer review only - http://bmjopen.bmj.com/site/about/guidelines.xhtml

Streamline C

Table 3: Questionnaire schedule. Questionnaires will be posted to patients by UCL CTC.

	Baseline (peri-staging)	Months after registration		
		1	3	6
Demographic measures	✓			
Co-morbidities	✓			
Self-rated health	✓			
Symptoms of bowel cancer	✓			
Concern for future consequences	✓			
Which staging tests have had	✓	✓		
Perceived importance of test attributes	✓	✓		
Perceived acceptability of different tests	✓	✓		
Least acceptable aspects of different tests	✓	✓		
Agreement to having tests again (short and long-term)	✓	✓		
Single test choice	✓	✓		
Test recovery time	✓	✓		
Patient experience of WB-MRI and standard tests	✓	✓		
Perceived efficacy of WB-MRI and standard tests		✓		
Difficulties with completing scan (WB-MRI and standard tests)		✓		
Ranked attribute preferences	✓	✓		
Feelings about number of tests			✓	✓
EQ-5D	✓		✓	✓
PANAS	✓	✓	✓	✓
GHQ-12	✓	✓	✓	✓

For peer review only

For peer review only - http://bmjopen.bmj.com/site/about/guidelines.xhtml

8.2.11. Cost Effectiveness Assessment

Overview

We will undertake a detailed analysis of the cost and the cost-effectiveness of WB-MRI versus standard NICE-approved staging algorithms. The analysis will conform to accepted economic evaluation methods [17]. All costs will be assessed from the perspective of the NHS and personal social services (PSS).

Making comparisons between WB-MRI versus standard staging algorithms

The care pathway for colorectal cancer patients can both be divided into two stages, the *treatment decision pathway* and the *subsequent disease pathway*. The former includes the time from diagnosis to treatment decision by the MDT; the latter includes the time period following the treatment decision.

The treatment decision pathway will be different between WB-MRI and standard staging algorithms, yielding different costs and potentially different treatment decisions. In patients for whom the treatment decision with WB-MRI is the same as that with conventional staging algorithms, the subsequent disease pathways will be the same. Where the treatment decision with WB-MRI is different, the disease pathway will be different, yielding potentially different costs and health outcomes.

If in the patients studied the concordance between the treatment decisions associated with WB-MRI and conventional staging algorithms is high, then the economic analysis can focus on the cost of the treatment decision pathways only because the disease pathways will be no different. In this case the cost-effectiveness of WB-MRI versus conventional staging algorithms depends only on the incremental cost (positive or negative) of WB-MRI versus standard staging algorithms in the treatment decision pathway.

Conversely, if the concordance between the treatment decisions is low, then the economic analysis ought to focus on both the treatment decision pathways and the subsequent disease pathways because both of these will vary between WB-MRI and conventional staging algorithms. In this case the cost-effectiveness of WB-MRI depends on the incremental cost of the WB-MRI versus standard staging algorithms in the treatment decision pathway plus the incremental costs and health benefits of the disease pathway.

The precise nature of the economic analysis will therefore depend on the degree of concordance between treatment decisions provoked by WB-MRI versus conventional staging.

Discordance of major treatment decisions

Discordance will be defined when the first major treatment decision differs between WB-MRI and standard investigations in greater than 10% of patients. Concordance will be defined as the absence of discordance. For colorectal patients a 4x4 table will be used to summarise major differences between 4 main treatment decisions (surgery for the primary but no chemotherapy, surgery for the primary and chemotherapy (and/or radiotherapy), chemotherapy (and/or radiotherapy) without surgery, and surgical metastectomy with or without chemotherapy) for colorectal cancer).

For peer review only - http://bmjopen.bmj.com/site/about/guidelines.xhtml

Streamline C

Scenario 1: there is concordance between the treatment decisions associated with WB-MRI and standard staging

In this case, the cost components included in the analysis will be:

- Conventional tests for staging of disease among diagnosed patients (e.g., CT chest, pelvis and abdomen and MRI pelvis for colon cancer; plus additional tests as indicated by the conventional staging algorithm);
- Costs of treating adverse events associated with staging tests;
- WB-MRI, plus additional tests generated by WB-MRI;
- MDT meetings.

The volume of resource use for each cost component will be measured directly in the trial from treatment decisions recorded in MDT reports, based first on conventional staging alone and then based on WB-MRI alone, and on patient records included in the trial. Unit costs will be taken from standard published sources. Since the two algorithms yield the same treatment decisions cost-effectiveness depends on the incremental cost of WB-MRI versus conventional staging algorithms in the treatment decision pathway (i.e., formally this is a cost-minimisation analysis).

Scenario 2: there is discordance between the treatment decisions associated with WB-MRI and standard staging

In this case, cost-effectiveness depends on the incremental cost (positive or negative) of the treatment decision pathway and disease pathway associated with WB-MRI versus conventional staging and the incremental health benefits (positive or negative). We will calculate cost-effectiveness in terms of the incremental cost per quality-adjusted life year (QALY) gained using one year and lifetime time horizons.

For the analysis based on the one-year time horizon, the trial will provide information on the treatment decisions arising from WB-MRI and standard staging and follow up data for the first year. We will collect these data from three sources.

Firstly, for all patients in the trial we will collect the resource use data for the main drivers of hospital costs using a trial specific CRF. This will collect resource use data on the following cost components for each patient:

- Imaging investigations
- Chemotherapy
- Radiotherapy
- Surgery and biopsies
- Outpatient visits
- Inpatient stays
- Day cases

These data will be recorded prospectively based mainly on MDT consensus meeting recommendations and on patient records (for major changes in treatment). Unit costs will be taken from standard published sources and applied to the resource use data, allowing us to cost the care received by each patient.

In addition, from this source we will collect data on whether or not the patient died during the follow up period, and if they died, the date of death.

For peer review only - http://bmjopen.bmj.com/site/about/guidelines.xhtml

Secondly, for all patients in the trial we will prospectively collect resource use data using patient diaries. This will supplement the above data which may not provide a complete picture of hospital resource use, plus it will allow us to collect data on primary and community care contacts. These will record resource use data on the following cost components for each patient:

- Medications taken
- Day cases
- GP contacts
- Practice and community nurse contacts
- A&E Visits
- Any other primary care or community care contacts related to cancer

The diaries will be posted out by UCL CTC at baseline, 3, 6, and 9 months and patients will be asked to complete them for the following 3 month period.

In addition we will also collect data on health-related quality of life score, measured according to the EQ-5D (www.euroqol.org), which will be measured at baseline and at 3, 6, 9 and 12 months for all surviving patients.

UCL CTC will contact sites just prior to sending out any post registration diaries or questionnaires to check the status of the patient to ensure none are sent inappropriately to deceased patients.

Patients who are found not to have cancer or are withdrawn for other reasons (see section 13), will not be sent diaries, quality of life or patient experience questionnaires to complete. Thirdly, the two sources of resource use data described above will be supplemented by retrospective review of resource use (including imaging, outpatient visits, inpatient stays and medication use) for the 12 months post diagnosis of up to 150 patients diagnosed with colorectal cancer at UCLH. This retrospective review will be carried out by a member of the clinical team and according to guidance provided by the UCLH Caldicott guardian. All collated retrospective data will be fully anonymised before sharing with other trial researchers outside the clinical care team.

8.2.12. Nested substudies

1) Diagnostic performance of limited WB-MRI protocols based on diffusion and T1 weighted imaging only, and to assess the incremental benefit of intravenous gadolinium contrast enhancement.

   As noted above, WB-MRI will be analysed using a locked sequential viewing paradigm such that the diagnostic accuracy of Diffusion and non contrast enhanced T1 images, Diffusion, non contrast enhanced T1 and T2 images, and Diffusion, non contrast enhanced T1, T2 and contrast enhanced T1 images can be calculated.

2) Effect of radiologists' diagnostic confidence on the accuracy of WB-MRI.

   Radiologists will indicate their level of confidence for the presence or absence of metastasis on a 1-6 scale on the CRF recording their interpretation of the WB-MRI.

3) Inter-observer variation in WB-MRI interpretation.

For peer review only - http://bmjopen.bmj.com/site/about/guidelines.xhtml

Streamline C

Each hub radiologist (n=6) will interpret a sample of 25 WB-MRI datasets selected at random from the other imaging hubs to define inter-observer variation in the reported presence absence of metastatic disease.

4) The collated WB-MRI datasets and 12 month patient follow up data collated as part of the trial protocol provides opportunities to retrospectively mine the data for additional diagnostic indices and prognostic markers (See appendix 4). The following or similar may be quantified in all or a subset of whole body MRI datasets:

- Body fat (organ, visceral and whole body),
- MRI signal from the primary tumour and metastatic sites, notably diffusion weighted signal, apparent diffusion coefficient, T1 (including fat and water components), T2 signal, Contrast enhancement, and textural analysis (for heterogeneity) of the primary and metastatic deposits on the various sequences.

For peer review only

For peer review only - http://bmjopen.bmj.com/site/about/guidelines.xhtml

9. Data Management Guidelines

Data will be collected from sites on version controlled case report forms (CRFs) designed for the trial and supplied by UCL CTC. Data entered onto CRFs must reflect source data at site. Some data will be recorded directly on the CRFs (i.e. no prior written or electronic record of data), and it will be considered to be source data. This will be made clear in the CRF guidance.

Where supporting documentation (e.g. autopsy reports, pathology reports, CT scan images etc.) is being submitted to UCL CTC, the patient's trial number must be clearly indicated on all material and any patient identifiers removed/blacked out prior to sending to maintain confidentiality.

9.1. Completing Case Report Forms

All CRFs must be completed and signed by staff who are listed on the site staff delegation log and authorised by the PI to perform this duty. The PI is responsible for the accuracy of all data reported in the CRF.

All entries must be clear, legible and written in ball point pen. The use of abbreviations and acronyms must be avoided.

Any corrections made to a CRF at site must be made by drawing a single line through the incorrect item ensuring that the previous entry is not obscured. Each correction must be dated and initialled. Correction fluid must not be used.

The use of abbreviations and acronyms must be avoided.

Once completed the original CRFs must be sent to UCL CTC and a copy kept at site.

9.2. Missing Data

To avoid the need for unnecessary data queries CRFs must be checked at site to ensure there are no blank fields before sending to UCL CTC. When data are unavailable because a measure has not been taken or test not performed, enter "ND" for not done. If an item was not required at the particular time the form relates to, enter "NA" for not applicable. When data are unknown enter the value "NK" (only use if every effort has been made to obtain the data).

9.3. Timelines for data return

CRFs must be completed at site and returned to UCL CTC as soon as possible and within 4 weeks of scheduled time point. For further details, see CRF instructions.

Sites who persistently do not return data within the required timelines may be suspended from recruiting further patients into the trial by UCL CTC and subjected to a 'for cause' monitoring visit. See section 12.2 ('For cause' on-site monitoring) for details.

For peer review only - http://bmjopen.bmj.com/site/about/guidelines.xhtml

9.4. Data Queries

Data arriving at UCL CTC will be checked for legibility, completeness, accuracy and consistency, including checks for missing or unusual values. Query reports will be sent to the data contact at site. Further guidance on how data contacts should respond to Data Queries can be found on the Query Reports.

For peer review only

For peer review only - http://bmjopen.bmj.com/site/about/guidelines.xhtml

10. Safety Reporting

Clinical Review

The MRI Imaging Hub and/or Recruiting site will record any complications attributable to the WB-MRI or any additional test performed as a result of the WB-MRI, notably contrast reactions, biopsy complications (infection, bleeding or hospital admission), and treatment target breaches on the Staging Complications CRF which will be submitted to UCL CTC.

UCL CTC will provide trial data to the CI on a periodic basis for review. If it is declared necessary to revise the conduct of the trial due to safety concerns, UCL CTC will inform the REC as appropriate.

For peer review only - http://bmjopen.bmj.com/site/about/guidelines.xhtml

11. Incident Reporting

Organisations must notify UCL CTC of all deviations from the protocol or GCP immediately. UCL CTC may require a report on the incident(s) and a form will be provided if the organisation does not have an appropriate document (e.g. Trust Incident Form).

If site staff are unsure whether a certain occurrence constitutes a deviation from the protocol or GCP, the UCL CTC trial team can be contacted immediately to discuss.

UCL CTC will use an organisation's history of non-compliance to make decisions on future collaborations.

For peer review only

For peer review only - http://bmjopen.bmj.com/site/about/guidelines.xhtml

12. Trial Monitoring and Oversight

Participating sites and PIs must agree to allow trial-related on site monitoring, Sponsor audits and regulatory inspections by providing direct access to source data/documents as required. Patients are informed of this in the patient information sheet and are asked to consent to their medical notes being reviewed by appropriate individuals on the consent form.

UCL CTC will determine the appropriate level and nature of monitoring required for the trial. Risk will be assessed on an ongoing basis and adjustments made accordingly.

12.1. Central Monitoring

Sites will be requested to submit relevant logs to UCL CTC at the frequency detailed in the trial monitoring plan or on request and these will be checked for consistency and completeness. Also refer to sections 4.2.2 (Required Documentation) and 6.2 (Screening Logs).

Ensuring patient eligibility is the responsibility of the PI or other delegated Investigator(s). Checks of the criteria listed on the registration form will be undertaken by an appropriately trained UCL CTC staff member prior to registration. Also refer to section 7.1 (Registration).

Details relating to the informed consent process will be collected on the registration form and are subject to review by UCL CTC as part of patient eligibility.

Data received at UCL CTC will be subject to review in accordance with section 9.4 (Data Queries).

Where central monitoring of data and/or documentation submitted by sites indicates that a patient may have been placed at risk, the matter will be raised urgently with site staff and escalated as appropriate (refer to section 11 (Incident Reporting) and 12.3 ('For cause' on site monitoring) for further details).

12.2. 'For Cause' On-Site Monitoring

On site monitoring visits may be scheduled at a site where there is evidence or suspicion of non-compliance with important aspect(s) of the trial protocol/GCP requirements. Sites will be sent a letter in advance outlining the reason(s) for the visit. The letter will include a list of the documents that are to be reviewed, interviews that will be conducted, planned inspections of the facilities, who will be performing the visit and when the visit is likely to occur.

Following a monitoring visit, the Trial Monitor/Trial Coordinator will provide a report to the site, which will summarise the documents reviewed and a statement of findings, deviations, deficiencies, conclusions, actions taken and actions required. The PI at each site will be responsible for ensuring that monitoring findings are addressed in a timely manner, and by the deadline specified.

For peer review only - http://bmjopen.bmj.com/site/about/guidelines.xhtml

UCL CTC will assess whether it is appropriate for the site to continue participation in the trial. Refer to section 11 (Incident Reporting) for details.

12.3. Oversight Committees

12.3.1. Trial Management Group (TMG)

The TMG will include the Chief Investigator, clinicians and experts from relevant specialities and Streamline C trial staff from UCL CTC (see page 3). The TMG will be responsible for overseeing the trial. The group will meet regularly at least once a year and will send updates to PIs (via newsletters or at Investigator meetings) and to the NCRI Colorectal Clinical Studies Group.

The TMG will review substantial amendments to the protocol prior to submission to the REC. All PIs will be kept informed of substantial amendments through their nominated responsible individuals.

TMG members must sign a charter confirming acceptance of their responsibilities.

12.3.2. Trial Steering Committee (TSC)

The role of the TSC is to provide overall supervision of the trial. The TSC will review the recommendations of the Independent Data Monitoring Committee and, on consideration of this information, recommend any appropriate amendments/actions for the trial as necessary. The TSC acts on behalf of the funder and Sponsor.

TSC members must sign a charter confirming acceptance of their responsibilities and declaring any potential conflicts of interest.

12.3.3. Independent Data Monitoring Committee (IDMC)

The role of the IDMC is to provide independent advice on data and safety aspects of the trial. Meetings of the Committee will be held at least once a year or as necessary to address any issues. The IDMC is advisory to the TSC and can recommend premature closure of the trial to the TSC.

IDMC members must sign a charter confirming acceptance of their responsibilities and declaring any potential conflicts of interest.

12.3.4. Role of UCL CTC

UCL CTC will be responsible for the day to day coordination and management of the trial and will act as custodian of the data generated in the trial (on behalf of UCL). UCL CTC is responsible for all duties relating to safety reporting which are conducted in accordance with section 10 (Safety Reporting).

For peer review only - http://bmjopen.bmj.com/site/about/guidelines.xhtml

13. Withdrawal of Patients

In consenting to the trial, patients are consenting to the WB-MRI, and also specifically consenting to completing questionnaires and diaries.

Losses to follow up

If a patient moves from the area, the site should make every effort to ensure the patient is followed up at another participating trial site and for this new site to take over the responsibility for the patient, or for follow up via GP. Details of participating trial sites can be obtained from the UCL CTC trial team who must be informed of the transfer of care and follow up arrangements.

Patients should be considered lost to follow up only once documented efforts on the part of the site have failed to produce any response or information from the patient or GP over the course of one year.

If a patient is lost to follow up at a site every effort should be made to contact the patient's GP to obtain information on the patient's status. If the patient cannot be contacted this should be recorded on the Change of Status CRF.

Patient Withdrawing Consent

If a patient expresses their wish to withdraw from the trial, sites should explain the importance of allowing routine follow up data to be used for trial purposes and for allowing existing collected data to be used. If the patient gives a reason for their withdrawal, this should be recorded (see below).

Withdrawal of Consent to Future Data Collection and use of past data

If a patient explicitly states they do not wish to contribute further data to the trial or allow the use of past data, their decision must be respected and recorded on the Change of Status CRF. In this event details should be recorded in the patient's hospital records, no further CRFs must be completed and no further data sent to UCL CTC.

Withdrawal of Consent to QoL, Resource use diary and/or Patient experience questionnaires

If a patient withdraws consent from completing the QoL, Resource use diary and/or the Patient experience questionnaires their decision must be recorded on the Change of Status CRF. UCL CTC will not post any further documents to the patient. However, the patient may continue to participate in the trial, and trial follow up completed as per protocol.

For peer review only - http://bmjopen.bmj.com/site/about/guidelines.xhtml

14. Trial Closure

14.1. End of Trial

For regulatory purposes the end of the trial will be 1 year after recruitment of the final patient at which point the 'declaration of end of trial' form will be submitted to ethical committees, as required.

Following this, UCL CTC will advise sites on the procedure for closing the trial at the site.

14.2. Archiving of Trial Documentation

At the end of the trial, UCL CTC will archive securely all centrally held trial related documentation for a minimum of 5 years. Arrangements for confidential destruction will then be made. It is the responsibility of PIs to ensure data and all essential documents relating to the trial held at site are retained for a minimum of 5 years after the end of the trial, in accordance with national legislation and for the maximum period of time permitted by the site.

Essential documents are those which enable both the conduct of the trial and the quality of the data produced to be evaluated and show whether the site complied with the principles of GCP and all applicable regulatory requirements.

UCL CTC will notify sites when trial documentation held at sites may be archived. All archived documents must continue to be available for inspection by appropriate authorities upon request.

Health Psychology data collected as part of this research will be retained for 10 years in line with Birkbeck, University of London's records management policy. At the end of this time period the documents will be shredded.

14.3. Early discontinuation of trial

The trial may be stopped before completion as an Urgent Safety Measure on the recommendation of the TSC or IDMC (see section 12.3.2 TSC and 12.3.3 IDMC). Sites will be informed in writing by UCL CTC of reasons for early closure and the actions to be taken with regards the treatment and follow up of patients.

14.4. Withdrawal from trial participation by a site

Should a site choose to close to recruitment the PI must inform UCL CTC in writing. Follow up as per protocol must continue for all patients recruited into the trial at that site and other responsibilities continue as per Clinical Trial Site Agreement (CTSA).

For peer review only - http://bmjopen.bmj.com/site/about/guidelines.xhtml

15. Quality Assurance

The technical quality of at least 10% of the WB-MRI from each imaging hub will be assessed by the trial radiographer at UCLH. Using the 3Dnet™ software, the radiographer will access performed scans on a weekly basis and enter an audit score of quality according to the definitions below. A report of the quality will be provided to the CI every 2 weeks and to the TMG at each meeting

Technical quality - general

1 = More than one sequence with substantial degradation of images severely limiting interpretation of those sequences, and not repeated

2 = One sequence with substantial degradation of images severely limiting interpretation of that sequence, and not repeated

3 = More than one sequence has minor artefact, but all remain fully diagnostic and repeat although optimal, not necessary OR all sequences initially technically inadequate (score 1 or 2) correctly repeated

4 = One sequence a has minor artefact, but remains fully diagnostic and repeat, although optimal, not necessary

5 = All sequences technically optimal with no artefact or degradation

Technical quality - anatomical coverage

1 = Wrong examination performed

2 = More than one sequence does not adequately cover the body (skull to mid thigh), or designated organ(s) coverage

3 = One sequence does not optimally cover the body or designated organ(s) coverage but examination remains fully diagnostic

4 = All sequences optimally cover the body and designated organ(s) coverage

For peer review only

For peer review only - http://bmjopen.bmj.com/site/about/guidelines.xhtml

16. Statistics

16.1. Sample size calculation

SAMPLE SIZE: Studies of diagnostic test accuracy are based on a cohort design where all patients receive all main diagnostic tests (CT or WB-MRI), although additional tests ordered in each staging pathway differ between patients, based on clinical appropriateness. Existing pathways and therapeutic decision making is complex for both cancers (see flow charts). This NIHR HTA trial focuses on first definitive staging and we are powered at this point in the patient pathway, informed by a detailed literature review.

Multiple therapeutic options are contingent on diagnosis of metastatic disease.

To power on any one of these (e.g. liver resection rates) would be beyond pragmatic recruitment and available funding.

We therefore power on a change in sensitivity for metastases detection with a WB-MRI pathway as a replacement to the standard NICE staging pathway.

Sample size calculation

- Paired trial design – comparing two diagnostic pathways where both are used in all patients.
- Sample size method for difference in paired proportions [22].
- Power trial to show difference in sensitivity between two diagnostic pathways, as fewer patients are with metastasis than without. Trials powered like this should also be suitable for difference in specificity.
- 80% power type II error, type I error 5% ($p<0.05$)
- Ratio of marginal cells in 2 x 2 table: $s/t=7$ (table 4)
- Sum of marginal cells in 2 x 2 table: $s+t/N = 0.14$
- A sample size of 116 patients with metastasis is needed, from a population of 40% prevalence of metastasis. Thus a total sample size of 290 (116 times 2.5) is required.

Table 4: Calculations comparing WB-MRI to PET

		MDT results for standard tests: patients with metastases detected		
	Result	+	-	Total
MDT results for WB-MRI plus additional tests: patients with metastases detected	+	85	14	99
	-	2	15	17
	Total	87	29	116

For peer review only - http://bmjopen.bmj.com/site/about/guidelines.xhtml

290 patients would be required to detect a difference of 10% in sensitivity of WB-MRI for metastatic disease (85%) compared to conventional staging (75%) given the disease prevalence as described above.

The sample size above must be adjusted to account for “withdrawals”. Patients are classified as withdrawn if they are recruited but are not evaluable for any of the following reasons (i) they do not have an WB-MRI scan, the main component of the primary outcome (ii) they do not have cancer (iii) they are lost to follow up before one year and do not have sufficient information for the expert panel to evaluate whether metastases were present.

The withdrawal rate was expected to be 10%, giving a target recruitment of 290 patients. However, the actual withdrawal rate has been 19% and the target recruitment has therefore been revised to 360 patients.

16.2. Population for analysis

The analysis population will be all patients, equivalent to the RCT population intention to treat (ITT) population, with multiple imputation used to impute missing data. For the primary outcome, complete case analysis (all available patient data with no imputation) will be reported alongside as a sensitivity analysis to the ITT analysis.

16.3. Analysis of the primary endpoint

Primary Outcome- Per patient sensitivity for metastasis detection by whole body MRI (WB-MRI) compared to standard staging pathways in newly diagnosed colorectal cancer against an expert derived consensus reference standard.

The primary objective is to evaluate whether early whole body magnetic resonance Imaging (WB-MRI) increases per patient sensitivity for metastasis in colorectal cancer compared to standard NICE-approved diagnostic pathways.

- Comparison to detect metastases between early WB-MRI alone and standard NICE diagnostic strategy.
- Difference in per patient sensitivity.
- Paired comparison of proportions of each strategy against the reference standard of full clinical diagnosis and 12 month follow up. [23]
- Imputation will be used to account for missing data and imperfect reference data. [24].

Definition of positive test result from standard tests (conventional imaging)

- Conventional imaging tests include CT and may include PET, USS, organ specific MRI, bone scans, lymph node sampling.
- Report from the MDT will express presence of metastatic disease for each patient categorised as yes, no and equivocal.

For peer review only - http://bmjopen.bmj.com/site/about/guidelines.xhtml

Streamline C

- Equivocal results will be grouped with positive test results as these results require additional follow up investigations compared to negative results.
- The radiology imaging report supplied to the MDT will be used for sensitivity analysis using free text report (possible and probable metastasis counted as positive).

Definition of positive test result from WB-MRI and additional tests requested after WB-MRI (WB-MRI as replacement test)

- WB-MRI imaging CRF report from the WB-MRI radiologist will express presence of metastatic disease for each patient categorised as yes, no and equivocal.
- Equivocal results will be grouped with positive test results as these results require additional follow up investigations compared to negative results.

Definition of positive test result from reference test: 12 month expert consensus panel

Expert panel results will be classified as positive or negative for metastasis by expert consensus. No equivocal category will be allowed.

Multiple imputation will be used for missing data using chained equations in STATA [25].

Sensitivity analysis: A sensitivity analysis will investigate the impact of the equivocal results on the detection rate by WB-MRI. Equivocal results will be grouped with negative test results [26].

A planned subgroup analysis will include: patients with rectal and non-rectal colorectal cancer.

16.4. Analysis of secondary endpoints

16.4.1. Efficacy (secondary)

Secondary outcome- The time and test number taken to reach, and the nature of, the first major treatment decision based on WB-MRI in comparison to standard staging pathways

The objective is to determine how WB-MRI influences time to and nature of first major treatment decision following definitive staging compared to standard investigations and to determine whether early WB-MRI could reduce or replace standard investigations.

In this secondary outcome, the following components will be measured up to the time of the first major treatment decision

- Nature of first major treatment decision and number of patients with changed management decision
- Time taken for diagnostic pathways
- Number of tests: average per patient, number of patients with fewer tests in pathway

These will be compared for WB-MRI pathway in comparison to standard staging pathways.

For peer review only - http://bmjopen.bmj.com/site/about/guidelines.xhtml

This information will be reported and discussed as a basis for whether early WB-MRI could replace or reduce standard investigations.

No absolute pre-specified definition of the combination of these outcomes likely to be considered clinically significant is attempted although it is noted that:

- An average decrease in diagnosis time of 7 days is likely to be considered clinically significant if accuracy were similar.

Secondary outcome- Diagnosis accuracy of WB-MRI and conventional staging pathways for local tumour staging and detection of metastasis in comparison to an expert derived consensus reference standard

The objective is to assess the accuracy of WB-MRI and standard diagnostic pathways for local and distant staging by comparison with an expert derived consensus reference standard using 1 year patient follow up data.

- All comparisons except difference in sensitivity per patient compare WB-MRI as both replacement and additional test compared to standard NICE staging pathway

The following outcomes will be assessed as secondary outcome #2:

- Difference in sensitivity per patient: WB-MRI as additional test compared to standard NICE staging pathway
- Difference in sensitivity and specificity per organ site. Count each site once per patient, regardless of the number of metastases per organ.
- Difference in sensitivity and specificity per metastasis.

Secondary outcome- Inter-observer variability in WB-MRI analysis and effect of diagnostic confidence on staging accuracy.

The objective is to determine the effect of radiologist confidence on the diagnostic accuracy of WB-MRI for metastatic disease.

A substudy will be conducted to look at inter-observer variability between radiologists interpreting WB-MRI images and the effect of diagnostic confidence on staging accuracy.

- Each hub radiologist (n=5) will interpret a sample of 25 WB-MRI datasets selected at random from the other imaging hubs to define inter-observer variation in the reported presence absence of metastatic disease.
- These images will be read after patient management decisions are taken and so will not affect patient diagnosis or treatment.

Secondary outcome – Diagnostic accuracy of limited T1 and diffusion weighted sequences compared to full multi-sequence WB-MRI protocols

The objective is to evaluate the diagnostic performance of limited WB-MRI protocols based on diffusion and T1 weighted imaging only, and to assess the incremental benefit of intravenous gadolinium contrast enhancement.

Comparison of diagnostic accuracy of WB-MRI alone (i) block 1 sequence (ii) block 2 (iii) block 3 sequences.

- Reference standard: WB-MRI plus standard tests with 12 month follow up.

For peer review only - http://bmjopen.bmj.com/site/about/guidelines.xhtml

- Methods and tables: as per primary outcome and secondary outcome #3.
- Analysed for units of analysis as (i) per patient (ii) per organ site (iii) per metastasis.

16.4.2. Safety

We will provide information on the following:

- Additional tests ordered as a result of WB-MRI but not ordered for the patient in the standard NICE staging pathway. Note this might include tests due to abnormalities outside colon.
- Extra days in hospital due to extra tests ordered through WB-MRI.

16.4.3. Economic evaluation

Patient-specific utility profiles will then be constructed assuming a straight line relation between each of the patients EQ-5D scores at each follow up point. The quality-adjusted life-years (QALYs) experienced by each patient up to one year will be calculated as the area underneath this profile.

We will calculate for each individual patient in the trial their costs and outcomes up to one year. We will also have data from the retrospective review of resource use in UCLH patients previously diagnosed with colorectal cancer.

Individual patients will then be grouped according to the specific disease path depending on the treatment decision (e.g., surgery for the primary but no chemotherapy, surgery for the primary and chemotherapy (and/or radiotherapy), chemotherapy (and/or radiotherapy) without surgery, and surgical metastectomy with or without chemotherapy for colorectal cancer) and the accuracy of the staging result. We will calculate the mean costs and QALYs for each group. Mean costs and QALYs for WB-MRI versus standard staging algorithms will then be calculated based on the proportion in each group using each algorithm, which will be different since in this scenario there is discordance in treatment decision.

Cost-effectiveness will be calculated as the mean cost difference between WB-MRI versus standard staging algorithms divided by the mean difference in outcomes (QALYs) to give the incremental cost-effectiveness ratio (ICER). Non-parametric methods for calculating confidence intervals around the ICER based on bootstrapped estimates of the mean cost and QALY differences will be used [27]. The bootstrap replications will also be used to construct a cost-effectiveness acceptability curve, which will show the probability that WB-MRI is cost-effective at one year for different values of the NHS' willingness to pay for an additional QALY. We will also subject the results to extensive deterministic (one-, two- and multi-way) sensitivity analysis.

For the analysis based on the lifetime time horizon we will use the 1 year data described above. To extrapolate beyond the end of the 1 year follow up period we will develop a de novo cost-effectiveness model for the disease pathway, which will be populated via available evidence. These will model patient movements between long-term health states. The models will be similar in design to a recent NIHR HTA-funded trial of PET and MRI for detection of metastasis in breast cancer [28]. Following decisions about model structure, a list of parameter estimates required for the model will be developed. The specific details of the data to be used to populate the model will

For peer review only - http://bmjopen.bmj.com/site/about/guidelines.xhtml

be determined following the development of the structure and the systematic searches of the literature to identify existing models.

The upshot of this analysis is that we will calculate for each individual patient in the trial their lifetime costs and QALYs. Individual patients will then be grouped according to the specific disease path and the accuracy of the staging result, as before, and we will calculate the mean costs and QALYs for each group. Mean costs and QALYs for WB-MRI versus standard staging algorithms will then be calculated based on the proportion in each group using each algorithm, again as before. We will undertake deterministic (one-, two- and multi-way) and probabilistic sensitivity analysis, the latter assuming appropriate distributions and parameter values [29].

16.4.4. Health psychology assessment

Study 1: Patient interviews (approximate n=25)

No formal statistical analysis will be carried out on the information from the patient interviews. The interviews will provide in-depth qualitative data about the experience of cancer staging and in particular under-going WB-MRI, and will inform the modification of a questionnaire (study 2) designed to assess patient acceptability and experience of WB-MRI compared to standard tests, and also inform the design of a discrete choice experiment (study 3).

Study 2: Patient questionnaires (approximate n=50)

Comparative patient experience between WB-MRI and standard staging investigations, identification of important staging pathway attributes, comparative anxiety, expectations and attribute importance before and following the staging process will be analysed using within subjects ANOVA or the Wilcoxon matched-pair sign-test depending on whether the data are normally distributed or not.

Study 3: Discrete choice experiment (approximate n=50)

Sample size should be greater than (500*c)/(t*a) where t = the number of sets of choices, a = the number of scenarios to choose between in each choice, and c = the largest number of levels for any one attribute [30]. Assuming each person will undertake 15 sets of choices, there are two scenarios in each choice, and the largest number of levels for any one attribute is 3 then the required sample size is (500*3)/(15*2) = 50. We will administer this questionnaire to approximately 50 colon patients after staging has been completed, sampling across more than one of the imaging hubs. The questionnaire will be posted to patients with a pre-paid reply envelope. A reminder letter will be sent if no questionnaire has been returned within approximately 4 weeks. Analysis will conform to the checklist for conjoint analysis applications in health developed by the International Society for Pharmaceuticals and Outcome Research (ISPOR). We will use a random effects probit model for statistical analysis, which is appropriate given the data structure. Outcomes: The average relative importance weighting of attributes ascribed to standard versus WB-MRI staging pathways by patients diagnosed with colonic cancer.

For peer review only - http://bmjopen.bmj.com/site/about/guidelines.xhtml

16.4.5. Handling missing data

Multiple imputation will be used for missing data using chained equations in STATA [25].

For the primary outcome, complete case analysis (all available patient data with no imputation) will be reported alongside as a sensitivity analysis to the ITT analysis.

16.5. Interim analyses

Interim safety data will be supplied to the IDMC on a periodic basis from UCL CTC on:

- Accumulating information relating to recruitment and data quality (e.g. data return rates).
- Safety data:
  - Additional tests performed as a result of the WB-MRI that would not have otherwise been performed as part of the patient's standard diagnostic pathway. Note this might include tests due to abnormalities outside colon or lung.
  - Potential harms to patients (e.g. staging complications resulting from MRI).
  - Whole Body MRIs that were not revealed or revealed too early/late.
- Percentage missing data for primary outcome.
- Prevalence of metastasis within recruited patients (key sample size assumption).

There will be no interim analysis based on the trial outcomes of diagnostic accuracy as all diagnostic test results are made available for the MDT meeting which decides first management treatment decision.

For peer review only

For peer review only - http://bmjopen.bmj.com/site/about/guidelines.xhtml

17. Ethical Approvals

In conducting the trial, the Sponsor, UCL CTC and sites shall comply with all laws and statutes, as amended from time to time, applicable to the performance of clinical trials including, but not limited to:

- the principles of ICH Harmonised Tripartite Guideline for Good Clinical Practice
- the Human Rights Act 1998
- the Data Protection Act 1998
- the Freedom of Information Act 2000
- the Research Governance Framework for Health and Social Care, issued by the UK Department of Health (Second Edition 2005) or the Scottish Health Department Research Governance Framework for Health and Community Care (Second Edition 2006)

17.1. Ethical Approval

The trial will be conducted in accordance with the World Medical Association Declaration of Helsinki entitled 'Ethical Principles for Medical Research Involving Human Subjects' (1996 version) and in accordance with the terms and conditions of the ethical approval given to the trial.

The trial has received a favourable opinion from the London – Camden & Islington Research Ethics Committee.

UCL CTC will submit Annual Progress Reports to the REC, which will commence one year from the date of ethical approval for the trial.

17.2. Site Approvals

The Lead Comprehensive Local Research Network (CLRN), Central and East London CLRN, has given NHS permission following global governance checks. Local governance checks will be undertaken by local CLRNs associated with individual trial sites.

Evidence of approval from the Trust R&D for a trial site must be provided to UCL CTC. Sites will only be activated when all necessary local approvals for the trial have been obtained.

17.3. Protocol Amendments

UCL CTC will be responsible for gaining ethical approval, for amendments made to the protocol and other trial-related documents. Once approved, UCL CTC will ensure that all amended documents are distributed to sites and CLRNs as appropriate.

Site staff will be responsible for acknowledging receipt of documents and for implementing all amendments.

For peer review only - http://bmjopen.bmj.com/site/about/guidelines.xhtml

17.4. Patient Confidentiality & Data Protection

Patient identifiable data, including full name, address, date of birth, gender and NHS number will be required for the registration process and will be provided to UCL CTC. UCL CTC will preserve patient confidentiality and will not disclose or reproduce any information by which patients could be identified. Data will be stored in a secure manner and UCL CTC trials are registered in accordance with the Data Protection Act 1998 with the Data Protection Officer at UCL. Patient identifiable data will be passed onto Birkbeck, University of London. This is described in the patient information sheet and the patients will consent to it.

For peer review only

For peer review only - http://bmjopen.bmj.com/site/about/guidelines.xhtml

18. Sponsorship and Indemnity

18.1. Sponsor Details

Sponsor Name: University College London

Address: Joint Research Office
Gower Street
London
WC1E 6BT

Contact: Director of Research Support

Tel: 020 3447 9995/2178 (unit admin)

Fax: 020 3447 9937

18.2. Indemnity

University College London holds insurance against claims from participants for injury caused by their participation in the clinical trial. Participants may be able to claim compensation if they can prove that UCL has been negligent. However, if this clinical trial is being carried out in a hospital, the hospital continues to have a duty of care to the participant of the clinical trial. University College London does not accept liability for any breach in the hospital's duty of care, or any negligence on the part of hospital employees. This applies whether the hospital is an NHS Trust or otherwise.

Hospitals selected to participate in this clinical trial shall provide clinical negligence insurance cover for harm caused by their employees and a copy of the relevant insurance policy or summary shall be provided to University College London, upon request.

For peer review only - http://bmjopen.bmj.com/site/about/guidelines.xhtml

19. Funding

This trial is funded by the National Institute for Health Research Health Technology Assessment (NIHR HTA).

For peer review only

For peer review only - http://bmjopen.bmj.com/site/about/guidelines.xhtml

20. Publication Policy

The TMG will oversee the publication and presentation of the data to peer reviewed journals and scientific meetings. All members of the TMG will approve publications. The writing committee will be led by Professor Stuart Taylor and include all TMG members. All TMG members, Trial Coordinator and Trial Statistician will be authors on the publications and named individually.

For peer review only

For peer review only - http://bmjopen.bmj.com/site/about/guidelines.xhtml

21. References

1. *Cancer Stats from Cancer Research UK Website*. Available from: http://info.cancerresearchuk.org.cancerstats/.
2. Garden, O.J., et al., *Guidelines for resection of colorectal cancer liver metastases.* Gut, 2006. **55 Suppl 3**: p. iii1-8.
3. Abdalla, E.K., et al., *Improving resectability of hepatic colorectal metastases: expert consensus statement.* Ann Surg Oncol, 2006. **13**(10): p. 1271-80.
4. Buyse, M., et al., *Progression-free survival is a surrogate for survival in advanced colorectal cancer.* J Clin Oncol, 2007. **25**(33): p. 5218-24.
5. *NICE Guidance: Improving Outcomes in Colorectal Cancers 2004*. Available from: http://www.nice.org.uk/nicemedia/pdf/CSGCCfullguidance.pdf.
6. Floriani, I., et al., *Performance of imaging modalities in diagnosis of liver metastases from colorectal cancer: a systematic review and meta-analysis.* J Magn Reson Imaging, 2010. **31**(1): p. 19-31.
7. Qu, X., et al., *A meta-analysis of (1)(8)FDG-PET-CT, (1)(8)FDG-PET, MRI and bone scintigraphy for diagnosis of bone metastases in patients with lung cancer.* Eur J Radiol, 2012. **81**(5): p. 1007-15.
8. Ohno, Y., et al., *Non-small cell lung cancer: whole-body MR examination for M-stage assessment--utility for whole-body diffusion-weighted imaging compared with integrated FDG PET/CT.* Radiology, 2008. **248**(2): p. 643-54.
9. Pierce, D.A. and D.L. Preston, *Radiation-related cancer risks at low doses among atomic bomb survivors.* Radiat Res, 2000. **154**(2): p. 178-86.
10. Schmidt, G.P., M.F. Reiser, and A. Baur-Melnyk, *Whole-body MRI for the staging and follow-up of patients with metastasis.* Eur J Radiol, 2009. **70**(3): p. 393-400.
11. Squillaci, E., et al., *Staging of colon cancer: whole-body MRI vs. whole-body PET-CT--initial clinical experience.* Abdom Imaging, 2008. **33**(6): p. 676-88.
12. Yi, C.A., et al., *Non-small cell lung cancer staging: efficacy comparison of integrated PET/CT versus 3.0-T whole-body MR imaging.* Radiology, 2008. **248**(2): p. 632-42.
13. Risberg, T., et al., *Diagnostic delay causes more psychological distress in female than in male cancer patients.* Anticancer Res, 1996. **16**(2): p. 995-9.
14. Bastiaannet, E., et al., *Perception of burden experienced during diagnostic tests by melanoma patients with lymph node metastases.* Melanoma Res, 2009. **19**(1): p. 36-41.
15. von Wagner, C., et al., *Choosing between CT colonography and colonoscopy in the diagnostic context: a qualitative study of influences on patient preferences.* Health Expect, 2009. **12**(1): p. 18-26.
16. Harcourt, D., N. Rumsey, and N. Ambler, *Same-day diagnosis of symptomatic breast problems: Psychological impact and coping strategies.* Psychology, Health & Medicine 1999. **4**(1): p. 5.
17. *NICE Guide to the methods of technology appraisal 2008*. Available from: http://www.nice.org.uk/media/B52/A7/TAMethodsGuideUpdatedJune2008.pdf.
18. Bossuyt, P.M., et al., *Towards complete and accurate reporting of studies of diagnostic accuracy: The STARD Initiative.* Radiology, 2003. **226**(1): p. 24-8.
19. Bossuyt, P.M., et al., *Comparative accuracy: assessing new tests against existing diagnostic pathways.* BMJ, 2006. **332**(7549): p. 1089-92.

For peer review only - http://bmjopen.bmj.com/site/about/guidelines.xhtml

20. Ryan, M., et al., *Use of discrete choice experiments to elicit preferences.* Qual Health Care, 2001. **10 Suppl 1**: p. i55-60.
21. Ryan, M. and S. Farrar, *Using conjoint analysis to elicit preferences for health care.* BMJ, 2000. **320**(7248): p. 1530-3.
22. Julious, S.A., M.J. Campbell, and D.G. Altman, *Estimating sample sizes for continuous, binary, and ordinal outcomes in paired comparisons: practical hints.* J Biopharm Stat, 1999. **9**(2): p. 241-51.
23. Newcombe, R.G., *Improved confidence intervals for the difference between binomial proportions based on paired data.* Stat Med, 1998. **17**(22): p. 2635-50.
24. Reitsma, J.B., et al., *A review of solutions for diagnostic accuracy studies with an imperfect or missing reference standard.* J Clin Epidemiol, 2009. **62**(8): p. 797-806.
25. White, I.R., P. Royston, and A.M. Wood, *Multiple imputation using chained equations: Issues and guidance for practice.* Stat Med, 2011. **30**(4): p. 377-99.
26. Begg, C.B., R.A. Greenes, and B. Iglewicz, *The influence of uninterpretability on the assessment of diagnostic tests.* J Chronic Dis, 1986. **39**(8): p. 575-84.
27. Briggs, A.H., D.E. Wonderling, and C.Z. Mooney, *Pulling cost-effectiveness analysis up by its bootstraps: a non-parametric approach to confidence interval estimation.* Health Econ, 1997. **6**(4): p. 327-40.
28. Cooper, K.L., et al., *Positron emission tomography (PET) and magnetic resonance imaging (MRI) for the assessment of axillary lymph node metastases in early breast cancer: systematic review and economic evaluation.* Health Technol Assess, 2011. **15**(4): p. iii-iv, 1-134.
29. Briggs, A.H., K. Claxton, and M.J. Sculpher, *Decision modelling for health economic evaluation*. Oxford handbooks in health economic evaluation. 2006, Oxford: Oxford University Press. x, 237 p.
30. Johnson, R., *Conjoint Analysis Software*, I. Sawtooth Software, Editor.: Sequim, WA
31. Azvolinsky, A., *Cancer prognosis: role of BMI and fat tissue.* J Natl Cancer Inst, 2014. **106**(6): p. dju177.
32. Yip, C., et al., *Assessment of sarcopenia and changes in body composition after neoadjuvant chemotherapy and associations with clinical outcomes in oesophageal cancer.* Eur Radiol, 2014. **24**(5): p. 998-1005.
33. Attaran, S., et al., *A propensity-matched comparison of survival after lung resection in patients with a high versus low body mass index.* Eur J Cardiothorac Surg, 2012. **42**(4): p. 653-8.
34. Curvo-Semedo, L., et al., *Diffusion-weighted MRI in rectal cancer: apparent diffusion coefficient as a potential noninvasive marker of tumor aggressiveness.* J Magn Reson Imaging, 2012. **35**(6): p. 1365-71.
35. De Felice, C., et al., *Apparent diffusion coefficient on 3.0 Tesla magnetic resonance imaging and prognostic factors in breast cancer.* Eur J Gynaecol Oncol, 2014. **35**(4): p. 408-14.
36. Ohno, Y., et al., *Diffusion-weighted MRI versus 18F-FDG PET/CT: performance as predictors of tumor treatment response and patient survival in patients with non-small cell lung cancer receiving chemoradiotherapy.* AJR Am J Roentgenol, 2012. **198**(1): p. 75-82.

For peer review only - http://bmjopen.bmj.com/site/about/guidelines.xhtml

37. Zakaria, R., et al., *Diffusion-weighted MRI characteristics of the cerebral metastasis to brain boundary predicts patient outcomes.* BMC Med Imaging, 2014. **14**: p. 26.
38. Parikh, J., et al., *Changes in primary breast cancer heterogeneity may augment midtreatment MR imaging assessment of response to neoadjuvant chemotherapy.* Radiology, 2014. **272**(1): p. 100-12.

For peer review only

For peer review only - http://bmjopen.bmj.com/site/about/guidelines.xhtml

Appendix 1: Abbreviations

**CDR**	Clinical Data Repository
**CI**	Chief Investigator
**CLRN**	Comprehensive Local Research Network
**CRF**	Case Report Form
**CSP**	Coordinated System for gaining NHS Permission
**CT**	Computerised Tomography
**CTSA**	Clinical Trial Site Agreement
**DCE**	Direct Choice Experiment
**DPA**	Data Protection Act
**GCP**	Good Clinical Practice
**GHQ**	General Health Questionnaire
**ICER**	Incremental Cost-Effectiveness Ratio
**ICH GCP**	International Conference of Harmonisation-Good Clinical Practice
**IDMC**	Independent Data Monitoring Committee
**IRAS**	Integrated Research Application System
**ISF**	Investigator Site File
**ISRCTN**	International Standard Randomised Controlled Trial Number
**ITT**	Intention To Treat
**MDT**	Multi-Disciplinary Team
**MRI**	Magnetic Resonance Imaging
**NCRI**	National Cancer Research Institute
**NCRN**	National Cancer Research Network
**NHS**	National Health Service
**NICE**	National Institute for Clinical Excellence
**NIHR**	National Institute for Health Research
**NIHR HTA**	National Institute for Health Research Health Technology Assessment
**NRES**	National Research Ethics Service
**OPD**	Out Patients Department
**PACS**	Picture Archiving and Communications System
**PANAS**	Positive and Negative Affect
**PET**	Positron Emission Tomography
**PET-CT**	Positron Emission Tomography- Computerised Tomography
**PI**	Principal Investigator
**PSS**	Personal Social Services
**QA**	Quality Assurance
**QALY**	Quality Adjusted Life Year
**R&D**	Research & Development
**RCT**	Randomised Controlled Trial
**REC**	Research Ethics Committee
**RIS**	Radiology Information System
**SSI**	Site Specific Information
**STARD**	STAndards for the Reporting of Diagnostic accuracy studies
**T**	Tesla
**TMF**	Trial Master File

For peer review only - http://bmjopen.bmj.com/site/about/guidelines.xhtml
For peer review only

Streamline C

**TMG**	Trial Management Group
**TSC**	Trial Steering Committee
**UCL**	University College London
**UCLH**	University College London Hospital
**UCL CTC**	Cancer Research UK & UCL Cancer Trials Centre
**USS**	Ultrasound Scan
**VATS**	Video-Assisted Thoracoscopic Surgery
**WB-MRI**	Whole Body Magnetic Resonance Imaging
**WCBP**	Woman of Childbearing Potential

For peer review only

For peer review only - http://bmjopen.bmj.com/site/about/guidelines.xhtml

Appendix 2: Consent process

Patients will be recruited at several hospital sites, and for many, the actual WB-MRI will take place at a different hospital (one of the central imaging hubs). It is vital that recruited patients undergo informed consent, have a minimum of 24 hours to consider participation and are subsequently free to withdraw at any time.
The consent process must be sensitive to the needs of patients which may differ from individual to individual. It is important therefore to provide patients with options as to how they may provide their consent.

The proposed consent process for this trial is based on the experience of other similar trials. It was found that patients and recruitment sites found it more efficient if the designated recruiting individual at the peripheral hospital knows that after fully explaining the trial, the patient in principle is willing to take part. For all recruitment sites, patients are seen by a clinical practitioner after a suspected diagnosis of colon cancer based on an abnormal endoscopy, CT scan or barium enema. At this discussion the diagnosis is explained and the need for a series of staging tests (such as CT scanning, PET CT, and MRI etc.) is also explained. Because of the need to stage the patient quickly so treatment may start, these tests are often done within a very short time period. There is no scheduled return by the patient to an OPD during this staging process. Instead patients are next seen face to face after the MDT has made a decision about their treatment.

If consent in principle is given, the recruiter can then ring the central hub and there and then arrange the time of the MRI and thus whenever possible inform the patient immediately of the time and date so they can plan their attendance for the scan. Patients expressing an interest in participating in the trial will therefore be asked if they prefer to sign the consent form when first seen when the trial is explained (and this will be later filed in the medical notes and the conversation documented). It will be made perfectly clear that they are free to withdraw at any time and that the scan will be at least 24 hours later (usually more than this). This will save them having to re-attend the hospital simply to sign a consent form, incurring time and expense at a very busy and difficult time for them. Before they go home, where possible patients will be given the date of the WB-MRI and a copy of their signed consent form. The recruitment site can then register the patient with UCL CTC. The date of the WB-MRI will be at least 24 hours later and in reality likely several days later. The patient will take the full patient information sheet home to review at their leisure. They will be provided with a telephone number at the recruitment site to ring if on reflection, they want to opt out of the trial. If they opt out, this will be recorded in the medical notes and screening log. Patients will also be asked permission to be called by the recruitment site or imaging hub to remind them of the date of scan and confirm their attendance. Patients will retain the right to opt out at any time by cancelling or not attending their WB-MRI appointment.

As noted, a major advantage of this approach is that the patient does not have to make a special trip back to the hospital just to sign the consent form. The signed consent

For peer review only - http://bmjopen.bmj.com/site/about/guidelines.xhtml

form does not leave the recruitment hospital and can be more efficiently filed in the patient notes and a copy filed in the Investigator site file: an important aspect of trial governance and patient clinical care.

Note this method of consent has been previously deemed ethically sound and efficient in the NIHR HTA funded Siggar 1 trial (CT colonography, colonoscopy or barium enema for diagnosis or colorectal cancer in older symptomatic patients), Northern and Yorkshire MREC, MREC/3/3/075, which ran from 2004 to 2009 and recruited over 5000 patients to undergo CT colonography in a similar trial design to the current proposal. The trial team did pilot a scheme where patients returned a copy of their signed consent form after 24 hours if they still wanted to take part, but patients found this very cumbersome and confusing and much preferred the process proposed, using telephone confirmation. The procedure is supported by the patient representatives in the trial and has also worked successfully in the BOOST trial which randomised patients to standard care or endobronchial biopsy at exactly the same point in the patient pathway. The BOOST trial ran at many of the intended recruitment sites for Streamline C.

Patients will be given the option to come back and sign the consent form if they or their recruiter feel this is preferable.

Occasionally it may be more convenient for the patient to obtain written consent when the patient attends for the WB-MRI or before a planned conventional staging test. For example the patient may wish to take part in the trial, not wish to sign the consent form on the same day but also would prefer not to attend a hospital appointment just to sign the consent form. This is acceptable although it is the least desirable option. After the patient has signed consent they will need to be registered before proceeding with the WB-MRI and a copy of the consent form will need to be returned to the initial recruitment site.

For peer review only

For peer review only - http://bmjopen.bmj.com/site/about/guidelines.xhtml

Appendix 3: Minimum WB-MRI protocol dataset

MRI Protocol
Whole body is head to thigh

The aim is to complete the whole protocol in 60 minutes or less. Depending on the MRI technology available, it may be possible to use the 5mm slices for all axial imaging, although up to 7mm is acceptable if time constraints are problematic.

1. These specifications are a set of minimum requirements – where higher field strengths/scanner software permits the resolution/quality of sequences should be optimised (whilst keeping the maximum imaging time limited to 1 hour).

2. Scanning maybe performed at either 1.5T or 3T.

3. Whole-body coverage is defined as head to mid-thigh optimised for detection of metastases.

Imaging Procedure

a) A standard safety questionnaire should be completed.
b) For patients undergoing contrast enhancement: set up IV line in a vein in the antecubital fossa, connected to an automated injector with two syringes (contrast and saline flush).
c) Unless contra-indicated administer 20mg buscopan or 1mg glucagon iv. to be given just before the start of the scan.

1. Whole-body diffusion weighted imaging:
Axial: STIR-EPI (or other fat sat technique) diffusion weighted imaging. Fixed slice thickness of 5mm to 7mm (to match T2 and t1 weighted axials as below) two b-values (b50 and b900). A minimum acquisition matrix of 128 x 128 (or an interpolated equivalent) (rectangular FOV should be used if available and appropriate for the patient), as a reference a minimum SNR of 6 on b50 images (for liver) should be maintained if possible by increasing the number of averages. All imaging should be performed in gentle respiration (recommended as 4 stations of 50 slices beginning from the vertex to mid thighs). Diffusion imaging through the brain is optional.

2. Whole-body T2 weighted imaging:
Axial*:* Axial T2 weighted (without fat-suppression) imaging should be performed from vertex to mid-thigh. A 5 to 7 mm slice thickness should be used for all scanners. *Where possible (within the 60 minute imaging time)* respiratory and ECG triggering should be used for the chest, respiratory triggering alone for the upper abdomen. The head, neck, pelvis and legs should be scanned without any triggering. The number of stacks should be adjusted to cover the imaging volume.

For peer review only - http://bmjopen.bmj.com/site/about/guidelines.xhtml

4. **Pre-contrast T1 weighted imaging:**

DIXON Technique to be applied if available.

a. Axial: Whole-body T1 GRE (e.g. Flash 2D) non-contrast enhanced non fat sat. Image resolution and slice thickness should be ideally matched to T2 weighted imaging.

OR

b. Coronal: T1 fat saturated volume interpolated gradient echo imaging (e.g. 3D) pre contrast.

5. **Post-contrast T1 weighted imaging (if gadolinium not contraindicated or refused):**

Minimum data set
Axial liver (60-70 sec)
Axial lung (equilibrium phase)
SFOV axial head

Optional
Coronal (organ specific or whole body)

a. Axial: post contrast e.g. T1 fat saturated volume interpolated gradient echo imaging (3D) breath hold of the *liver (60-70 seconds delay) and lungs*. Multiple breath-holds employed to provide full volume coverage if required. A minimum of a 256x256 (rectangular FOV acquisition if possible and appropriate for the patient) acquisition matrix should be employed. 5-7 mm slice thickness.

b. Coronal: post contrast whole body; e.g.T1 fat saturated volume interpolated gradient echo imaging (3D) and post contrast. Slice thickness 5mm. Breath Hold.

c. **Axial:** fat saturated T1 weighted imaging of the brain (SFOV). An acquisition matrix of 256 x 256 should be employed.

For peer review only - http://bmjopen.bmj.com/site/about/guidelines.xhtml

Appendix 4: Exploratory Analysis

The collated WB-MRI datasets and 12 months patient follow up data collated as part of the trial protocol provides opportunities to retrospectively mine the data for additional diagnostic indices and prognostic markers. All analysis will be done by members of the research team under supervision of the Chief Investigator.

There is for example, increasing data linking total body fat with prognosis in malignancy [31, 32]. In lung cancer, a higher body mass index has been associated with a better prognosis [33]. From the WB-MRI datasets acquired as part of the trial, it is possible to extract quantitative data on total body fat using simple segmentation algorithms which can be linked with patients' outcomes such as metastatic status and 12 month survival.

In addition, quantification of the MRI signal returned from sites of disease may be able to predict histological characteristics and also may be linked to prognosis. For example diffusion weighted imaging of the primary tumour has been shown to differentiate between grades of dysplasia in rectal cancer [34] and may predict prognosis in lung, brain and breast cancer [35-37]. The heterogeneity of signal within tumours can be measured and has been linked with overall prognosis and treatment response. For example in breast cancer, increased homogeneity of the signal measured using regions of interest placed in metastatic deposits in response to treatment is associated with a better prognosis [38]. The various sequences performed as part of the collected WB-MRI protocol will allow testing of such associations in recruited patients.

The following or similar may be quantified in all or a subset of WB-MRI datasets:

1. Body fat (organ, visceral and whole body).
2. MRI signal from the primary tumour and metastatic sites, notably diffusion weighted signal, apparent diffusion coefficient, T1 (including fat and water components), T2 signal, Contrast enhancement, and textural analysis (for heterogeneity) of the primary and metastatic deposits on the various sequences.

We will look for correlations between MRI variables and clinical data collated as part of the trial (for example basic histology, metastatic status, patient outcome and findings of conventional staging tests) or collected as part of the patients' usual care, for example, more detailed histology of biopsies or excised tumour (including tumours markers if collected as part of usual clinical care). Associations will be sought between the MRI variables and the clinical variables using appropriate conventional parametric and non-parametric statistics, for example:

- Differences in body fat composition between different cancer histological subtypes, patients with and without metastatic disease, and according to 12 month survival
- Heterogeneity of MRI signal from a region of interest drawn within the primary tumours and metastatic deposits, comparing between lesions in the same

For peer review only - http://bmjopen.bmj.com/site/about/guidelines.xhtml

patient, between different patients and according to histological parameters and 12 month survival.

In addition comparisons will be made between MRI signal within normal body tissues (such as the liver, pancreas and muscle) between patients scanned on the different MRI scanners comparing with local MRI phantom data as appropriate to see how homogenous the MRI data is between different imaging hubs.

In some instances use of external commercial software may be required (e.g. fat quantification). MRI datasets will be anonymised and no personal identifiable information will be shared outside the research team at any time.

For peer review only

For peer review only - http://bmjopen.bmj.com/site/about/guidelines.xhtml

Appendix 5: Protocol Version History

**Protocol:**		**Amendments:**		
**Version no.**	**Date**	**Amendment no.**	**Protocol Section (no./title)**	**Summary of main changes from previous version.**
1.0				
1.1	14/09/12	N/A	Front cover & Section 1.1<br>Section 5<br>8.2.10<br>Section 14.2<br><br>Appendix 2	ISRCTN number and new CR UK logo added<br><br>Flowchart and text re consent process inserted<br>Text inserted re QoLs<br>Text inserted re destruction of interview recordings<br>Text inserted re consent process
2.0	15/03/13	1.0	Throughout<br>Section 4<br><br>Section 5<br><br><br>Section 7<br><br>Section 8<br><br><br><br><br><br>Section 9<br><br>Section 12<br>Section 13<br>Appendix 3	Typos and clarifications<br>Clarification about GCP training and documents required for activation updated<br>Details added about interpreters for informed consent and removal of informed consent log information<br>Removal of text regarding slot availability updates<br>Addition of ad hoc mini MDT, reduce the amount of patient experience questionnaires, change the content of these questionnaires and alter who is sending them to patients.<br>Clarifications of timing of WB-MRI and the process of early release of findings<br>Update data management guidelines in line with changes at UCL CTC<br>Details about informed consent log removed<br>Addition of details re patient withdrawal<br>Clarifications to Whole body MRI protocol
3.0	24/04/14	3.0	Section 5<br><br><br><br>Section 7<br><br>Section 8	Removal of information about QoL and Patient diaries given out by site at consent<br>Removal of patient diaries and QoLs from registration packs.<br>Updating information to state that the Interview section of the trial is now closed to recruitment.<br>Updating information that UCL CTC is now posting out QoL and patient diaries at baseline.

For peer review only - http://bmjopen.bmj.com/site/about/guidelines.xhtml

Streamline C

4.0	22/12/14	5.0	Section 8<br><br><br><br><br><br><br><br>8.2.12<br><br><br>Section 10<br><br><br>Appendix 4<br><br>Appendix 5	Rewording and clarification of Conventional Imaging, WB-MRI reveal and MDT discussion, Final Treatment decision and follow up sections. Updating information to state that the Interview section of the trial is now closed to recruitment. Updating information that UCL CTC is now posting out QoL and patient diaries at baseline.<br>Information added regarding exploratory analysis for additional diagnostic indices and prognostic markers.<br>Safety Reporting section updated to provide more information on staging complications and how these are reported and reviewed by the CI.<br>Exploratory Analysis added to include the additional analysis of WB-MRI scans<br>Protocol Version History has become Appendix 5.
5.0	27/08/15	5.0	Section 8<br><br><br><br><br><br>Section 10<br><br><br><br><br>Section 13<br><br><br>Section 16	Changing the target recruitment for the patient experience questionnaires to 50 patients and closing this part of the trial. Updating and clarifying information regarding the Discrete Choice Experiment and confirming the time point these will be sent to patients.<br>Updating the Safety Section to confirm that only staging complications related to the WB-MRI or additional tests performed due to WB-MRI are to be recorded on the Staging Complications CRF.<br>The withdrawal process was updated and clarified to better match the new Change of Status CRF.<br>The type of Interim safety data that will be supplied to IDMC was updated
6.0	06/09/16	7.0	Section 1<br><br><br><br>Section 13<br><br>Section 16	Recruitment target increased from 322 to 360 in sections 1.1 and 1.2.<br>Anticipated duration of recruitment increased from 3 to 4 years in sections 1.1 and 1.2.<br>Deletion of section on early release of WB MRI and patient failure to undergo WB MRI<br>Rationale for revised target accrual added to 16.1.<br>Grammatical and typographical errors corrected throughout

For peer review only - http://bmjopen.bmj.com/site/about/guidelines.xhtml

Streamline L

Streamlining Staging of Lung Cancer with Whole Body MRI

Trial Sponsor:	University College London (UCL)
Trial Sponsor reference:	UCL/12/0156
Trial funder:	NIHR HTA
Funder reference:	10/68/01
ISRCTN no:	50436483
Protocol version no:	6.0
Protocol version date:	06/09/2016

This project was funded by the National Institute for Health Research (NIHR) Health Technology Assessment (HTA) programme *(project number 10/68/01)*

For peer review only

Protocol version 6.0, 06/09/2016 Authorisation signatures

Name & Role:	Signature:	Date authorised:
**Chief Investigator:**<br>Professor Stuart Taylor<br>Professor of Medical Imaging, UCL	[illegible]	14/10/16
**For the Sponsor:**<br>Professor Jonathan Ledermann<br>Director, UCL CTC	[illegible]	14OCT2016
Sandy Beare<br>Tumour Group Lead, UCL CTC	[illegible]	14/10/2016

Please note: This trial protocol must not be applied to patients treated outside the Streamline L trial. UCL CTC can only ensure that approved trial investigators are provided with amendments to the protocol.

For peer review only - http://bmjopen.bmj.com/site/about/guidelines.xhtml

Coordinating Centre:

For general queries, supply of trial documentation and central data management please contact:

Streamline L Trial Coordinator
Cancer Research UK & UCL Cancer Trials Centre (UCL CTC)
90 Tottenham Court Road
London
W1T 4TJ

Tel: +44 (0) 20 7679 9878
Fax: +44 (0) 20 7679 9871
09:00 to 17:00 Monday to Friday

Email: ctc.streamlineL@ucl.ac.uk

Other trial contacts:

Chief Investigator: Professor Stuart Taylor
Address: Dept Specialist X Ray, Level 2 Podium,
University College London Hospital (UCLH)
235 Euston Road
London
NW1 2BU

Trial Management Group (TMG):

Prof Stuart Taylor	Professor of Medical Imaging	UCLH
Dr Dow-Mu Koh	Consultant Radiologist	The Royal Marsden
Prof Vicky Goh	Professor of Clinical Cancer Imaging	Guy's and St Thomas'
Prof Anwar Padhani	Clinical Lead in MRI	Mount Vernon
Dr Shonit Punwani	Senior Lecturer in Medical Imaging	UCLH
Prof Andrea Rockall	Professor of Cancer Imaging	Imperial College London
Prof Steve Halligan	Professor of Radiology	UCL
Dr Neal Navani	Consultant in Thoracic Medicine	UCLH
Prof Sam Janes	Wellcome Senior Fellow	UCLH
Dr John Bridgewater	Consultant Oncologist	UCLH
Alison Morton	Patient Representative	
Alf Oliver	Patient Representative	
Dr Anne Miles	Senior Lecturer in Health Psychology	Birkbeck College
Prof Steve Morris	Professor of Health Economics	UCL
Dr Susan Mallett	Senior Medical Statistician	University of Birmingham
Krystyna Reczko	Trial Coordinator	UCL CTC
Katie Prior	Data Manager	UCL CTC
Marian Duggan (maternity leave)	Senior Trial Coordinator	UCL CTC
Abby Sharp (maternity cover)	Senior Trial Coordinator	UCL CTC
Dr Sandy Beare	Tumour Group Lead	UCL CTC

For peer review only - http://bmjopen.bmj.com/site/about/guidelines.xhtml

Table of Contents

1. PROTOCOL SUMMARY ... 7
1.1. SUMMARY OF TRIAL DESIGN ... 7
1.2. TRIAL SCHEMA ... 9
2. INTRODUCTION ... 10
2.1. BACKGROUND ... 10
3. TRIAL OVERVIEW ... 12
3.1. PRIMARY OBJECTIVE ... 12
3.2. SECONDARY OBJECTIVES ... 12
3.3. TRIAL ACTIVATION ... 13
4. SELECTION OF SITES/SITE INVESTIGATORS ... 14
4.1. SITE SELECTION ... 14
4.1.1. Selection of Principal Investigator and other investigators at sites ... 14
4.1.2. Training requirements for site staff ... 14
4.2. SITE INITIATION AND ACTIVATION ... 15
4.2.1. Site initiation ... 15
4.2.2. Required documentation ... 15
4.2.3. Site activation letter ... 15
5. INFORMED CONSENT ... 16
6. SELECTION OF PATIENTS ... 18
6.1. PRE-REGISTRATION EVALUATION ... 18
6.2. SCREENING LOG ... 18
6.3. MULTI DISCIPLINARY (MDT) MEETING LOG ... 18
6.4. PATIENT ELIGIBILITY ... 18
6.4.1. Patient Inclusion Criteria ... 18
6.4.2. Exclusion Criteria ... 19
6.4.3. Pregnancy and Birth Control ... 19
7. REGISTRATION PROCEDURES ... 20
7.1. REGISTRATION ... 20
7.2. REGISTRATION PACKS ... 20
7.3. BOOKING WHOLE BODY MRI ... 20
8. PATIENT MANAGEMENT AND TRIAL PROCEDURES ... 22
8.1. SUMMARY ... 22
8.1.1. Staging and Treatment Decisions ... 22
8.1.2. Patient experience Interviews and Questionnaires (Optional) ... 23
8.1.3. Quality of Life Questionnaires and Patient Diaries ... 23
8.2. DETAILED DESCRIPTION ... 25
8.2.1. Standard care ... 25
8.2.2. Trial Imaging ... 25
8.2.3. Release of Conventional and trial WB-MRI findings ... 26
8.2.4. Final treatment plan ... 28
8.2.5. Early release of WB-MRI findings ... 28
8.2.6. Time to full staging ... 29
8.2.7. Patient follow up ... 29
8.2.8. Patient diaries and EQ-5D Quality of Life questionnaires ... 30
8.2.9. Final reference standard for tumour stage (Consensus Meetings) ... 30

For peer review only - http://bmjopen.bmj.com/site/about/guidelines.xhtml

8.2.10. Health psychology assessment ... 31
8.2.11. Cost Effectiveness Assessment ... 34
8.2.12. Nested substudies ... 36

9. DATA MANAGEMENT GUIDELINES ... 38

9.1. COMPLETING CASE REPORT FORMS ... 38
9.2. MISSING DATA ... 38
9.3. TIMELINES FOR DATA RETURN ... 38
9.4. DATA QUERIES ... 39

10. SAFETY REPORTING ... 40

11. INCIDENT REPORTING ... 41

12. TRIAL MONITORING AND OVERSIGHT ... 42

12.1. CENTRAL MONITORING ... 42
12.2. 'FOR CAUSE' ON SITE MONITORING ... 42
12.3. OVERSIGHT COMMITTEES ... 43
12.3.1. Trial Management Group (TMG) ... 43
12.3.2. Trial Steering Committee (TSC) ... 43
12.3.3. Independent Data Monitoring Committee (IDMC) ... 43
12.3.4. Role of UCL CTC ... 43

13. WITHDRAWAL OF PATIENTS ... 44

14. TRIAL CLOSURE ... 45

14.1. END OF TRIAL ... 45
14.2. ARCHIVING OF TRIAL DOCUMENTATION ... 45
14.3. EARLY DISCONTINUATION OF TRIAL ... 45
14.4. WITHDRAWAL FROM TRIAL PARTICIPATION BY A SITE ... 45

15. QUALITY ASSURANCE ... 46

16. STATISTICS ... 47

16.1. SAMPLE SIZE CALCULATION ... 47
16.2. POPULATION FOR ANALYSIS ... 48
16.3. ANALYSIS OF THE PRIMARY ENDPOINT ... 49
16.4. ANALYSIS OF SECONDARY ENDPOINTS ... 50
16.4.1. Efficacy (secondary) ... 50
16.4.2. Safety ... 51
16.4.3. Economic evaluation ... 51
16.4.4. Health psychology assessment ... 52
16.4.5. Handling missing data ... 53
16.5. INTERIM ANALYSES ... 53

17. ETHICAL APPROVALS ... 55

17.1. ETHICAL APPROVAL ... 55
17.2. SITE APPROVALS ... 55
17.3. PROTOCOL AMENDMENTS ... 55
17.4. PATIENT CONFIDENTIALITY & DATA PROTECTION ... 56

18. SPONSORSHIP AND INDEMNITY ... 57

18.1. SPONSOR DETAILS ... 57
18.2. INDEMNITY ... 57

19. FUNDING ... 58

20. PUBLICATION POLICY ... 59

For peer review only - http://bmjopen.bmj.com/site/about/guidelines.xhtml

Streamline L

21. REFERENCES 60
APPENDIX 1: ABBREVIATIONS 63
APPENDIX 2: CONSENT PROCESS 65
APPENDIX 3: MINIMUM WB-MRI PROTOCOL DATASET 67
APPENDIX 4: EXPLORATORY ANALYSIS 69
APPENDIX 5: PROTOCOL VERSION HISTORY 71

For peer review only

For peer review only - http://bmjopen.bmj.com/site/about/guidelines.xhtml

1. Protocol Summary

1.1. Summary of Trial Design

**Title:**	Streamlining Staging of Lung Cancer with Whole Body MRI
**Short Title/acronym:**	Streamline L
**Sponsor name & reference:**	UCL/12/0156
**Funder name & reference:**	NIHR HTA
**ISRCTN no:**	50436483
**Design:**	Multicentre comparison
**Overall aim:**	To evaluate whether early whole body magnetic resonance Imaging (WB-MRI) increases per patient sensitivity for metastasis in non-small cell lung cancer compared to standard NICE-approved diagnostic pathways.
**Primary endpoint:**	Per patient sensitivity for metastasis detection by whole body MRI (WB-MRI) compared to standard staging pathways in newly diagnosed non-small cell lung cancer
**Secondary endpoints:**	1. The time and test number taken to reach, and the nature of, the first major treatment decision based on WB-MRI in comparison to standard staging pathways.<br>2. Diagnostic accuracy of WB-MRI and conventional staging pathways for local tumour staging and detection of metastasis in comparison to an expert derived consensus reference standard.<br>3. Lifetime incremental cost and cost-effectiveness of staging using WB-MRI compared to standard diagnostic pathways.<br>4. Patient experience of staging using WB-MRI in comparison to standard diagnostic pathways and priorities placed by patients on differing attributes related to competing staging pathways.<br>5. Inter-observer variability in WB-MRI analysis and effect of diagnostic confidence on staging accuracy.<br>6. Diagnostic accuracy of limited T1 and diffusion weighted sequences compared to full multi-sequence WB-MRI protocols.
**Target accrual:**	353

For peer review only - http://bmjopen.bmj.com/site/about/guidelines.xhtml

**Inclusion & exclusion criteria:**	**Inclusion criteria:**<br>• Adult patients (18 or over) with suspected primary non-small cell lung cancer on chest CT with sufficient confidence to trigger staging investigations/biopsy OR with already histologically proven primary non-small cell lung cancer<br>• Disease is potentially radically treatable as defined as stage IIIb or less on diagnostic CT (i.e. T1-4, N0-2, M0)<br>• Performance status 0-2 (fit to undergo radical treatment if indicated)<br>• Patient must have given written informed consent and be willing to comply with the protocol intervention and follow up.<br>**Exclusion criteria:**<br>• Any psychiatric or other disorder likely to impact on informed consent<br>• Evidence of severe or uncontrolled systemic disease which makes it undesirable for the patient to participate in the trial<br>• Pregnancy<br>• Contraindications to MRI (e.g. cardiac pacemaker, severe claustrophobia, inability to lie flat)<br>• Unequivocal metastatic or N3 disease on diagnostic CT chest and abdomen (including M1a disease; malignant pleural effusion)<br>• Further staging work up not indicated in the opinion of the MDT due to poor performance status or patient choice<br>• Histologies other than non-small cell lung cancer.
**Planned number of sites:**	17 (including 7 Imaging hubs)
**Target Country**	UK
**Trial Procedure:**	All patients will undergo a whole body MRI protocol in addition to the standard staging protocol employed at their institution.
**Anticipated duration of recruitment:**	4 years
**Duration of patient follow up:**	12 months
**Definition of end of trial:**	12 months after enrolment of the final patient.
**Other related research:**	Sub-study of WB-MRI generalisability

For peer review only - http://bmjopen.bmj.com/site/about/guidelines.xhtml

1.2. Trial Schema

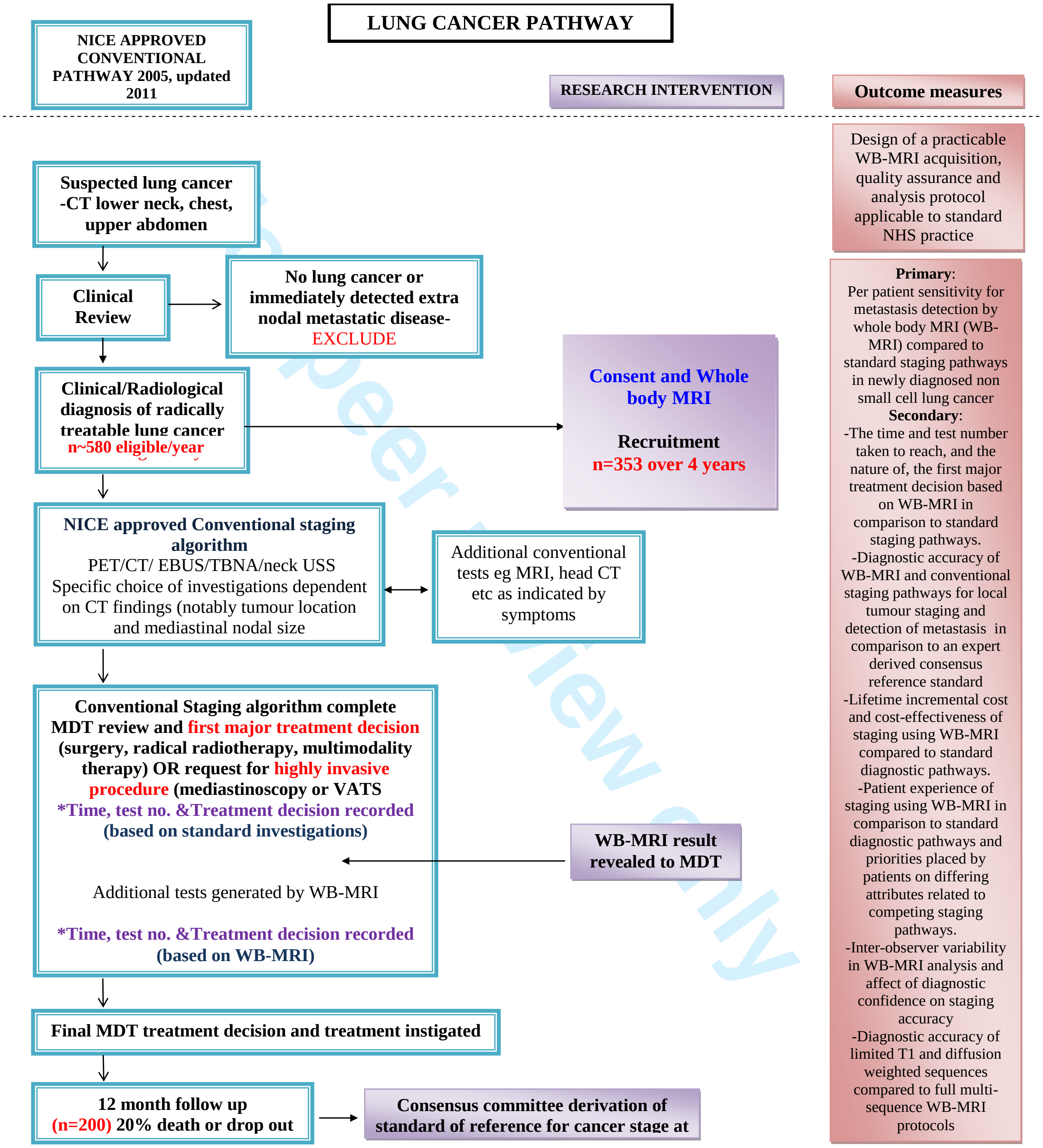

Key: EBUS= Endobronchial Ultrasound. TBNA= transbronchial needle aspiration. USS=ultrasound VATS= video-assisted thoracoscopic surgery

For peer review only - http://bmjopen.bmj.com/site/about/guidelines.xhtml

2. Introduction

2.1. Background

Lung cancer is the second most common malignancy in the UK and accounts for 13% of all new cancer diagnosis; approximately 40,000 patients are diagnosed per year [1]. Treatment decisions for lung cancer are critically dependent upon rapid and accurate tumour staging at diagnosis. In particular detection of metastatic disease (i.e. disease which has disseminated beyond the primary tumour into distant organ sites such as bone, liver and brain) is vital to optimised therapeutic triage. Although 1 year lung cancer survival in the UK is just 33% [2], correctly staged and treated early stage disease (i.e. without metastatic spread) achieves between 54 and 80% 5 year survival. Conversely, currently over 20% of patients undergoing "curative" thoracotomy for presumed early stage disease rapidly relapse with metastatic spread undetected by conventional staging tests - so called "futile thoracotomy" [3]. Accurate local staging of the tumour and spread to lymph nodes is also of importance. For example in patients with non-small cell lung cancer, spread to contra lateral mediastinal lymph nodes precludes surgery with curative intent.

Staging of lung cancer within the NHS is reliant on high technology imaging platforms such as computerised tomography (CT), positron emission tomography (PET) and magnetic resonance imaging (MRI), together with standard scintigraphy, plain X-Rays and ultrasound. Minimally invasive tissue sampling techniques such as endobronchial ultrasound (EBUS) are also increasingly used. Staging pathways are complex (see flowchart, section 1.2), and governed by NICE approved guidelines [2] which delineate an integrated step by step deployment of the various imaging modalities before tumour staging is deemed complete and the first treatment decision is made. The complexity of current staging algorithms reflects the differing diagnostic attributes of the various imaging modalities across tumour types and body organs. CT for example is superior to ultrasound for detecting liver metastasis [4], but inferior to scintigraphy in diagnosing malignant spread to the bone. PET scanning is in general superior to scintigraphy for detecting bone metastases [5], but not as effective as MRI in identifying spread to the brain [6]. It is not unusual for a patient newly diagnosed with lung cancer to undergo standard chest and abdominal CT, whole body PET, brain CT, and invasive mediastinal nodal sampling before the first definitive treatment is decided. Such a stepwise multi-modality approach is time and resource intensive and onerous for patients at a very difficult time following their cancer diagnosis. Furthermore patients receive significant doses of diagnostic ionising radiation during the staging process - a single PET-CT imparts 6-10 times normal annual background radiation and even small doses of radiation can increase an individual's risk of subsequent malignancy [7].

Recent technological advances mean an MRI scan of the whole body can be acquired in less than one hour; sensitivity for metastatic cancer spread is high [8]. MRI does not impart ionising radiation and is advocated as a safe and effective alternative to the current multi-modality approach. There is no available secondary research evidence concerning the accuracy of WB-MRI in cancer staging. Indeed meta-analysis would be challenging because literature is limited to relatively small cohort studies comparing WB-MRI with conventional imaging tests, and in a wide variety of cancers. These do however suggest potential as an efficient and accurate "one stop shop" alternative for

For peer review only - http://bmjopen.bmj.com/site/about/guidelines.xhtml

cancer staging [6, 9, 10]. For example, in 123 patients with non-small cell cancer, Yi found no difference between PET-CT and WB-MRI, both achieving 86% accuracy [10]. Notably, the vast majority of available studies are single site, using one MRI platform, with interpretation by one or two experienced radiologists. The literature is deficient regarding generalisability of WB-MRI. For example, variability amongst interpreting radiologists is unknown and the effect (if any) of different manufacturers' platforms is uncertain. It is also unclear how WB-MRI can be best integrated into existing NHS pathways, which frequently differ between institutions, or how effective it would be in different clinical settings. By undertaking a multi–centre trial of lung cancer using a variety of MRI platforms and interpreting radiologists, the current proposal will address these unknown variables.

Health psychology assessment

WB-MRI staging of lung cancer could reduce the physical and psychological burden associated with current pathways. By reducing time from diagnosis to treatment, WB-MRI may reduce patient anxiety, minimising concerns that treatment delay might adversely affect prognosis. Delay in diagnosis is known to be distressing, particularly for women [11]. In addition, staging scans can be uncomfortable and embarrassing [12]. MRI for example, while avoiding radiation, is noisy and claustrophobic. The potential of WB-MRI to generate additional tests is also an important consideration, although recent data suggest test sensitivity is the key determinant of patient choice and influences preference more than discomfort and complications [13]. While rapid staging means patients without metastasis are in receipt of good news more quickly, it also means those with metastasis will also receive bad news earlier, which may impact adversely on psychological wellbeing: It is known that patients are more depressed following a speedier diagnosis of breast cancer (at a one-stop clinic) when compared to those waiting a week [14].

Cost Effectiveness Analysis

Although WB-MRI is a relatively expensive imaging technology, other standard tests in the staging pathways such as PET-CT are also resource intensive. A detailed analysis of the cost and the cost-effectiveness of WB-MRI versus standard NICE-approved staging algorithms conforming to accepted economic evaluation methods [15] is essential to any trial investigating the role of WB-MRI in cancer staging.

For peer review only - http://bmjopen.bmj.com/site/about/guidelines.xhtml

3. Trial Overview

This trial will be an independent multi-centre prospective cohort trial comparing the staging accuracy of early WB-MRI with standard pathways in newly diagnosed non-small cell lung cancer. We will follow the recommendations of the STAndards for the Reporting of Diagnostic accuracy studies (STARD) initiative [16]. A key element of our design is the investigation of early WB-MRI as a replacement test for standard pathways [17], and data regarding the therapeutic impact of WB-MRI will be collated, as well as addressing the practicalities of general NHS implementation. The trial design, which compares two different staging strategies in the same patients ensures that clean, comparable data are collected for both pathways while simultaneously increasing trial power to meet trial endpoints.

In addition, both qualitative and quantitative assessments will be employed to determine the psychological burden and acceptability of WB-MRI versus standard pathways, and to identify those attributes which most strongly influence patient preference. Health-related quality of life data will be collected which will be used to inform cost-effectiveness modelling, and determine whether patients believe more rapid staging would have helped them cope better with their cancer diagnosis.

3.1. Primary objective

To evaluate whether early whole body magnetic resonance Imaging (WB-MRI) increases per patient sensitivity for metastasis in non-small cell lung cancer compared to standard NICE-approved diagnostic pathways.

3.2. Secondary objectives

1) To determine how WB-MRI influences time to and nature of first major treatment decision following definitive staging compared to standard investigations and to determine whether early WB-MRI could reduce or replace standard investigations.
2) To assess the accuracy of WB-MRI and standard diagnostic pathways for local and distant staging by comparison with an expert derived consensus reference standard using 1-year patient follow up data.
3) To evaluate the lifetime incremental cost and cost-effectiveness of staging using WB-MRI compared to standard diagnostic pathways.
4) To evaluate patients' experiences of staging using WB-MRI and to determine the priorities placed by patients on differing attributes related to competing staging pathways, including impact of reducing time to first treatment.
5) To determine the effect of radiologist confidence on the diagnostic accuracy of WB-MRI for metastatic disease.
6) To evaluate the diagnostic performance of limited WB-MRI protocols based on diffusion and T1 weighted imaging only, and to assess the incremental benefit of intravenous gadolinium contrast enhancement.

For peer review only - http://bmjopen.bmj.com/site/about/guidelines.xhtml

Additional objectives which will be met by the trial will be the definition of an efficient WB-MRI acquisition and analysis protocol, and quality assurance (QA) program applicable to standard NHS practice and collation of a database of WB-MRI datasets for NHS staff training and service development.

3.3. Trial activation

UCL CTC will ensure that all trial documentation has been reviewed and approved by all relevant bodies and that the following have been obtained prior to activating the trial:

- Research Ethics Committee approval
- 'Adoption' into NIHR portfolio
- NHS permissions
- Adequate funding for central coordination
- Confirmation of sponsorship
- Adequate insurance provision

For peer review only - http://bmjopen.bmj.com/site/about/guidelines.xhtml

4. Selection of Sites/Site Investigators

4.1. Site selection

In this protocol a ‘recruitment site’ refers to a hospital where certain trial-related activities are conducted.

Recruitment sites must be able to comply with:

- Patient recruitment, follow up schedules and all requirements of the trial protocol
- Requirements of the Research Governance Framework
- Data collection requirements
- **No** trial imaging, recruitment sites will refer patients to designated imaging hub (see below)

In this protocol an ‘imaging hub’ refers to a hospital where certain trial-related activities are conducted.

Imaging hubs must be able to comply with:

- Patient recruitment (where applicable), follow up schedules (where applicable) and all requirements of the trial protocol
- Requirements of the Research Governance Framework
- Data collection requirements
- Trial imaging

4.1.1. Selection of Principal Investigator and other investigators at sites

Sites must have an appropriate Principal Investigator (PI), i.e. a health care professional authorised by the site and ethics committee, to lead and coordinate the work of the trial on behalf of the site. Other investigators at site wishing to participate in the trial must be trained and approved by the PI. All investigators must be medical doctors and have experience of diagnosing, staging or treating lung cancer.

4.1.2. Training requirements for site staff

All site staff must be appropriately qualified by education, training and experience to perform the trial related duties allocated to them, which must be recorded on the site delegation log.

CVs for all staff must be kept up to date, with signed and dated and copies held in the Investigator Site File (ISF). An up to date, signed copy of the CV for the PI must be forwarded to UCL CTC upon request.

All staff involved in the trial must receive GCP training which is relevant to their role and responsibilities within the trial. The frequency of repeat training may be dictated by the requirements of their employing institution, or 2 yearly where the institution has no policy, and more frequently when there have been updates to the legal or regulatory requirements for the conduct of clinical trials.

For peer review only - http://bmjopen.bmj.com/site/about/guidelines.xhtml
For peer review only

4.2. Site initiation and activation

4.2.1. Site initiation

Before a site is activated, the UCL CTC trial team will arrange a site initiation with the site which the PI and site research team must attend. The site will be trained in the day to day management of the trial, and essential documentation required for the trial will be checked.

Site initiation will be performed for each site by site visit or teleconference.

4.2.2. Required documentation

The following documentation must be submitted by the site to UCL CTC prior to a site being activated by UCL CTC trial team:

- Trial specific Declaration of Participation/Site Registration Form (identifying relevant local staff)
- All relevant institutional approvals (e.g. local NHS permission)
- A completed site delegation log that is initialled and dated by the PI
- A copy of the PI's current CV that is signed and dated

In addition, the following agreement must be in place:

- A signed Clinical Trial Site Agreement (CTSA) between the Sponsor and the relevant institution (usually a NHS Trust)

4.2.3. Site activation letter

Once the UCL CTC trial team has received all required documentation and the site has been initiated, a site activation letter will be issued to the PI. **Sites may not begin to approach patients until the site activation letter has been issued.**

Once the site has been activated by UCL CTC, the PI is responsible for ensuring:

- adherence to the most recent version of the protocol;
- all relevant site staff are trained in the protocol requirements;
- appropriate recruitment and medical care of patients in the trial;
- timely completion and return of CRFs (including assessment of all adverse events).

For peer review only - http://bmjopen.bmj.com/site/about/guidelines.xhtml

5. Informed consent

Sites are responsible for assessing a patient's capacity to give informed consent.

Sites must ensure that all patients have been given the current approved version of the patient information sheet, are fully informed about the trial and have confirmed their willingness to take part in the trial by signing the current approved consent form.

Sites must assess a patient's ability to understand verbal and written information in English and whether or not an interpreter would be required to ensure fully informed consent. If a patient requires an interpreter and none is available, the patient should not be considered for the trial.

The PI, or, where delegated by the PI, other appropriately trained site staff, are required to provide a full explanation of the trial to each patient prior to trial entry. During these discussions, the current approved patient information sheet for the trial should be discussed with the patient. A minimum of 24 hours before the WB-MRI must be allowed for the patient to consider and discuss participation in the trial.

The consent process has been left deliberately flexible in order to accommodate the needs of individual patients and variations in Site requirements. The flowchart below describes three different options for obtaining written informed consent from patients, each of which ensures that patients have at least 24 hours to consider their participation in the trial before any trial-related activities take place.

Streamline Recruitment Pathways/Consent Process

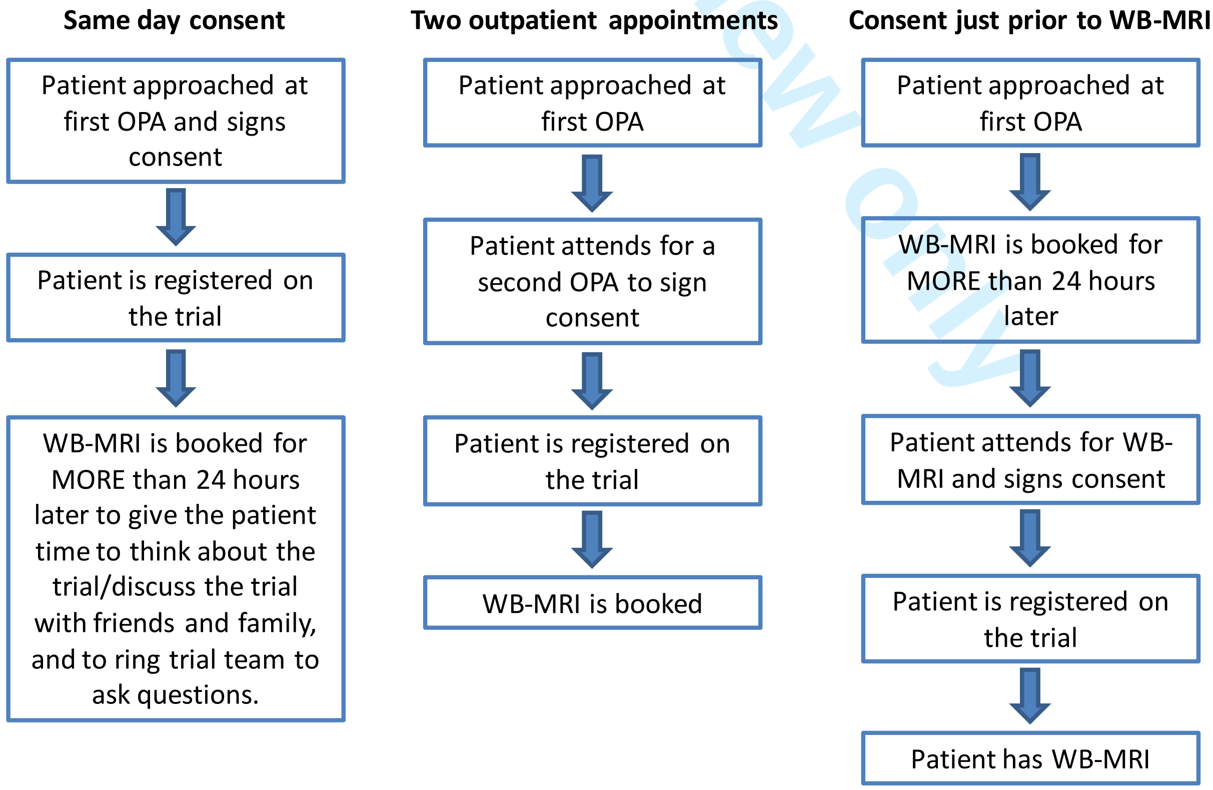

For peer review only - http://bmjopen.bmj.com/site/about/guidelines.xhtml

See Appendix 2 for a more detailed example of the consent process.

Written informed consent on the current approved version of the consent form for the trial must be obtained before any trial-specific procedures are conducted. The discussion and consent process must be documented in the patient notes.

Site staff are responsible for:

- checking that the correct (current approved) version of the patient information sheet and consent form are used;
- checking that information on the consent form is complete and legible;
- checking that the patient has completed/initialled all relevant sections and signed and dated the form;
- checking that an appropriate member of staff has countersigned and dated the consent form to confirm that they provided information to the patient;
- checking that an appropriate member of staff has made dated entries in the patient's medical notes relating to the informed consent process (i.e. information given, consent signed etc.);
- Following registration:
    - adding the patient trial number to all copies of the consent form, which should be filed in the patient's medical notes and investigator site file,
    - giving the patient a copy of their signed consent form and patient information sheet.

The right of the patient to refuse to participate in the trial without giving reasons must be respected. All patients are free to withdraw at any time. Also refer to section 13.0 (Withdrawal of patients).

For peer review only - http://bmjopen.bmj.com/site/about/guidelines.xhtml

6. Selection of Patients

6.1. Pre-registration Evaluation

The following assessments or procedures are required to evaluate the suitability of patients for the trial:

- Histologically proven or clinically diagnosed (defined as radiological diagnosis of lung cancer on chest CT with sufficient confidence to trigger staging investigations) primary non-small cell lung cancer with potentially radically treatable disease
- No contraindications to MRI (e.g. cardiac pacemaker, severe claustrophobia, inability to lie flat)

6.2. Screening Log

A screening log must be maintained by the site and kept in the Investigator Site File. This must record all potentially eligible patients approached about the trial and the reasons why they were not registered in the trial if this is the case. The log must be sent to UCL CTC in line with the monitoring plan and as requested, with patient identifiers removed prior to sending.

6.3. Multi Disciplinary (MDT) Meeting Log

An anonymous record of the number of patients with a new diagnosis of non-small cell lung cancer discussed in the MDT meeting, with their attributed cancer stage will be submitted annually to UCL CTC. This will be used to assess whether the group of patients recruited to the trial is representative of the general population of newly diagnosed lung cancer patients seen at Sites.

6.4. Patient Eligibility

There will be no exception to the eligibility requirements at the time of registration. Queries in relation to the eligibility criteria must be addressed prior to calling/faxing for registration. Patients are eligible for the trial if all the inclusion criteria are met and none of the exclusion criteria applies.

6.4.1. Patient Inclusion Criteria

- Adult patients (18 or over) with suspected primary non-small cell lung cancer on chest CT with sufficient confidence to trigger staging investigations/biopsy OR with already histologically proven primary non-small cell lung cancer
- Disease is potentially radically treatable as defined as stage IIIb or less on diagnostic CT (i.e. T1-4, N0-2, M0)
- Performance status 0-2 (fit to undergo radical treatment if indicated)
- Patient must have given written informed consent and be willing to comply with the protocol intervention and follow up.

For peer review only - http://bmjopen.bmj.com/site/about/guidelines.xhtml
For peer review only

6.4.2. Exclusion Criteria

- Unequivocal metastatic or N3 disease on diagnostic CT chest and abdomen (including M1a disease; malignant pleural effusion)
- Further staging work up not indicated in the opinion of the MDT or clinician due to poor performance status or patient choice.
- Histologies other than non-small cell lung cancer
- Any psychiatric or other disorder likely to impact on informed consent
- Evidence of severe or uncontrolled systemic disease which makes it undesirable for the patient to participate in the trial
- Pregnancy
- Contraindications to MRI (e.g. cardiac pacemaker, severe claustrophobia, inability to lie flat)

6.4.3. Pregnancy and Birth Control

MRI poses a theoretical risk to the foetus, particularly in the first trimester due to local acoustic and heating effects. However the risk is generally deemed very small and significantly less than the risk of ionising radiation imparted by PET-CT and CT (standard imaging investigations).

A woman of childbearing potential (WCBP) is a sexually mature woman (i.e. any female who has ever experienced menstrual bleeding) and who has not undergone a hysterectomy or who has not been postmenopausal for 24 consecutive months (i.e. who has had menses at any time in the preceding 24 consecutive months). Patients will be excluded if they are pregnant.

There is no requirement for additional contraceptive advice to patients over and above that routinely given as part of their routine clinical care given their diagnoses of lung cancer.

The need to perform a pregnancy test in WCBP will be decided as part of the patient's routine clinical care given the risk to pregnancy from standard staging investigations and subsequent treatment. There is no requirement to perform a pregnancy test purely because of recruitment to the trial if this would not have been performed as part of standard clinical care.

For peer review only - http://bmjopen.bmj.com/site/about/guidelines.xhtml

7. Registration Procedures

7.1. Registration

Patient registration will be undertaken centrally at UCL CTC and this must be performed prior to commencement of any trial intervention. Registration can be performed via telephone or fax.

Following pre-registration evaluations (as detailed in section 6.1), confirmation of eligibility and consent of a patient at a site, the registration form must be fully completed prior to faxing or telephoning UCL CTC. The eligibility criteria will be reviewed during the registration telephone call using the same form at UCL CTC.

A trial number will be assigned for the patient during the call and must be recorded at site by the caller. If desired, the site may fax a copy of the completed registration form to UCL CTC and the faxed registration from will be used to confirm patient eligibility at UCL CTC.

UCL CTC will email confirmation of the patient's inclusion in the trial and their trial number to the main contact, PI and Imaging Hub contact. If requested, a fax confirmation may also be sent. Case report forms will be emailed to the main contact at site.

Registration telephone number:	+44 (0)20 7679 9880
Registration fax number:	+44 (0)20 7679 9871
UCL CTC Office hours:	09:00 to 17:00 Monday to Friday

7.2. Registration Packs

Once a patient has consented to take part in the trial they should be provided with a registration pack to include the following:

- A copy of their signed consent form and patient information sheet
- An expenses claim form (to claim travel expenses to and from WB-MRI appointment)
- Pre-paid return envelope

7.3. Booking Whole Body MRI

Once a patient has been registered it is the responsibility of the recruiting site to contact their designated imaging hub to make arrangements for the WB-MRI. Each recruitment site will be provided with a contact number for their designated imaging hub. Recruitment sites will approach consecutive potentially eligible patients until the number of recruited patients fills the MRI capacity of the imaging hub.

For peer review only - http://bmjopen.bmj.com/site/about/guidelines.xhtml

It is possible recruitment potential at recruitment sites will be greater than the WB-MRI capacity at each central imaging hub. If a patient is approached and registered for the trial but MRI capacity is such that the scan could not take place in a timely fashion, a decision to withdraw the patient will be made by the recruitment site and the patient will then be informed. This possibility is explained to patients in the patient information sheet.

Because of the need to perform WB-MRI in a timely fashion, it is anticipated recruitment sites will be requested to pause recruitment if WB-MRI waiting time exceeds 2 weeks (if WB-MRI is performed after conventional imaging is complete) or 3 weeks (if WB-MRI is to be performed concurrently with standard tests). There will be flexibility in these timings depending on the circumstances of individual patients and their anticipated time to complete standard staging.

For peer review only

For peer review only - http://bmjopen.bmj.com/site/about/guidelines.xhtml

8. Patient Management and Trial Procedures

8.1. Summary

8.1.1. Staging and Treatment Decisions

The following information is required for the trial to document staging and treatment decisions:

- Stage and treatment decision based on conventional imaging only (and the number, timing, nature and findings of these investigations)
- Stage and theoretical treatment decision based on WB-MRI (and any additional tests generated by the WB-MRI) only (and the number, timing, nature and findings of additional tests generated)
- A final treatment decision incorporating all available tests.

Conventional Imaging

Trial patients will undergo the conventional staging protocol employed at their institution as per usual clinical care pathways. See section 8.2.1 for further detail.

Whole Body MRI Imaging and MDT Reveal

Recruited patients will undergo a WB-MRI at one of the designated imaging hubs. Recruiting sites should contact their designated imaging hub to request a WB-MRI slot (see section 7.3). The WB-MRI should be performed either concurrently with the standard staging investigations, or no later than 3 weeks after the final standard staging investigation. Images will be uploaded at each hub to a secure central imaging server called 3Dnet™. If a language interpreter was required for a patient during the consent process, then an interpreter should be made available to the patient during the WB-MRI. The WB-MRI will be reported by designated radiologist(s) at each imaging hub who are blinded to the conventional imaging tests and other clinical information (other than the cancer diagnosis and location). A copy of the report will also be uploaded to 3Dnet™. See section 8.2.2 for further detail.

Once the MDT has made a treatment decision based on the conventional imaging the MDT coordinator, research nurse or other designated individual will reveal the WB-MRI images and report on 3Dnet™ via an internet-enabled PC. The MDT will review the WB-MRI results and decide whether or not any additional tests are required or theoretically would have been required (if in fact the test was already performed as part of conventional staging) in addition to the WB-MRI. If any tests are required that have not already been performed as part of the standard staging, they should be requested and the patient reviewed again at the next MDT when the results are available. If no further tests are needed then the stage and theoretical treatment decision based on the WB-MRI (and the results of any actual or theoretical additional tests generated from the WB-MRI) must then be made by the MDT. The MDT must then decide a final treatment decision for the patient based on all imaging available.

If a patient is due to start treatment before the next formal MDT meeting, an ad hoc mini MDT meeting may be scheduled outside of the standard MDT meeting. This should include the relevant multidisciplinary clinical team members who are appropriate

For peer review only - http://bmjopen.bmj.com/site/about/guidelines.xhtml

to make the final treatment decision based on the specific imaging finding, according to usual standards of clinical care. A record will be kept of who is present during the ad hoc mini MDTs on the CRFs.

8.1.2. Patient experience Interviews and Questionnaires (Optional)

Interviews (Study 1)

A total of 25 patients have undergone interviews by the Health Psychologist pertaining to their experience of the cancer staging process. **Target accrual has now been reached for the interview part of the trial and recruitment to this is now closed**.

Questionnaires (Study 2)

Patient experience questionnaires from baseline and post-staging have been received for at approximately 50 patients to assess patient experience and acceptability of WB-MRI and standard tests. **Target accrual has now been reached for the questionnaire part of the trial and recruitment to this is now closed.**

Discrete choice experiment (Study 3)

A discrete choice experiment (DCE) questionnaire will be administered to a subset of approximately 50 patients to elicit their preference for various attributes associated with conventional and WB-MRI staging pathways. The DCE questionnaire is optional and patients will be asked to consent to this on trial entry. Once a patient consents to the DCE they will be allocated a DCE questionnaire (out of a possible 2 DCE questionnaires) which will be posted by UCL CTC along with their Quality of Life questionnaire, patient diary and pre-paid envelope immediately after registration (baseline). UCL CTC will supply the details of any recruited patients who consented to complete DCE questionnaire to the Health psychologists who may contact them directly.

8.1.3. Quality of Life Questionnaires and Patient Diaries

Upon consent the recruiting site must inform the patient that they will receive an EQ-5D Quality of Life Questionnaire and Patient Diary through the post from UCL CTC and they are to complete these and return them using the pre-paid envelope provided. The patient diary will cover a 3 month period and will collect information regarding the patient's Primary Health Care contacts, other health care contacts and also the medication they are currently taking. Patient diaries and EQ-5D questionnaires will be posted to all patients at consent and then at 3 monthly intervals for 12 months.

For peer review only - http://bmjopen.bmj.com/site/about/guidelines.xhtml

Streamline L

Table 1: Summary of patient management and trial activities

Activity	Protocol section	Timing							Responsible organisation/person
		Prior to registration	Immediately after registration	During staging	After staging (months after registration)				
					1	3	6	9	
Pre-registration evaluation & assessment of eligibility	6.1	X							Recruiting site
Consent	5.0	X							Recruiting site
Register patient	7.1	X							Recruiting site
Give patient registration pack (copy of PIS and consent, expenses claim form and pre-paid return envelope)	7.2		X						Recruiting site
Book WB-MRI scan & inform patient of appointment date	7.3		X						Recruiting site
WB-MRI	8.2.2			X					Imaging Hub
Upload WB-MRI scan & report	8.2.2			X					Imaging Hub
Post EQ-5D questionnaires	8.2.7		X			X	X	X	UCL CTC
Post patient resource diary	8.2.7		X			X	X	X	UCL CTC
Discrete choice experiment Questionnaire	8.2.10		X						UCL CTC

For peer review only

For peer review only - http://bmjopen.bmj.com/site/about/guidelines.xhtml

8.2. Detailed description

8.2.1. Standard care

Trial patients will undergo the conventional staging investigations employed at their recruiting institution according to local protocols and the requirements of their clinical care team.

All conventional investigations will be performed and interpreted by the usual radiologists and clinicians employed at the site of the investigations. Standard clinical reports will be made and all investigations (and their results) will be freely available on hospital Picture Archiving and Communications System (PACS), Radiology Information System (RIS) and Clinical Data Repository (CDR) systems as per usual clinical practice.

The type and date of investigations (e.g. CT scan, PET-CT, organ specific MRI, biopsy etc.) will be recorded on CRFs, along with the presence and location of metastatic disease based on the radiological report.

8.2.2. Trial Imaging

Whole Body MRI (WB MRI) protocol

Recruited patients will undergo a WB-MRI at their designated imaging hub site. The choice of MRI platform (i.e. manufacturer and Tesla (T) strength) will be decided by the local hub radiologist according to scanner availability and their usual practice. It is anticipated most MRIs will be performed at 1.5T. Exact imaging parameters will vary according to MRI platform but a minimum dataset of sequences will be acquired (full details given in appendix 3).

Timing

The WB-MRI will be performed either concurrently with the standard staging investigations, or no later than 3 weeks after their final standard staging investigation.

Blinding

To ensure the integrity of the trial, the WB-MRI must be reported by a radiologist blinded to the conventional imaging tests and other clinical information (other than the cancer diagnosis and site). Conversely the WB-MRI images and reports must be blinded to radiologists reporting the standard staging investigations, and those involved in direct patient care before the WB-MRI is revealed in the MDT.

The un-anonymised WB-MRI images will therefore not be immediately sent to the Picture Archive and Communication System (PACS) at either the imaging hub or recruitment sites. Instead images will be uploaded to a secure central imaging server (3Dnet™) provided by Biotronics3D. This solution allows easy upload of MRI datasets via standard internet connection. A PC based internet gateway will be installed in each imaging hub to facilitate automated transfer of WB-MRI from the scanner/workstation to 3Dnet™), and thereafter automatically back to PACS to the appropriate time point after MDT revelation (see below).

For peer review only - http://bmjopen.bmj.com/site/about/guidelines.xhtml

Interpretation and reporting

The WB-MRI will be interpreted by designated radiologist(s) at each imaging hub who are expert in interpretation based on previous experience of reporting WB-MRI in cancer staging. As noted above, the radiologist will be blinded to the standard staging investigations performed on recruited patients.

Interpretation can be performed using the 3Dnet™ software, or stand alone workstation according to the preference of the radiologist.

Images will be analysed in the following order:

1) Diffusion and non contrast enhanced T1 images
2) Diffusion, non contrast enhanced T1 and T2 images
3) Diffusion, non contrast enhanced T1, T2 and contrast enhanced T1 images.

After viewing each sequence set (and before reviewing the next set), the radiologist will complete WB-MRI Imaging Booklet CRFs documenting the presence, location and size of metastatic disease, together with their diagnostic confidence on a scale of 1 to 6. Reporting time for each sequence set analysis (defined as the time required to interrogate the sequences to reach a diagnostic conclusion) will be recorded along with the technical quality of the MRI dataset.

The reporting radiologist will then produce a free text clinical report as per their usual clinical practice (using all available sequences and based on the TMN 7 staging guidance) for subsequent release to the clinical team. This report will contain information relating to the local T and N stage of the tumour, together with the presence, location, number and size of metastatic deposits, as well as important "incidental" findings, for example aortic aneurysm. The radiologist may express their level of confidence in reported findings as they would in normal clinical practice but a formal numerical score of confidence will not be provided as this would not mirror how radiological examinations are reported in standard clinical care. If the radiologist would usually recommend additional tests for equivocal findings, this will be included in the report so as to also mirror routine clinical practice. The definitions of T, N M stage will be based on conventional MRI criteria adopted by the radiologist in their usual clinical practice.

The free text report will be uploaded onto the 3Dnet™ software, and a copy stored as part of the patient's medical record.

8.2.3. Release of Conventional and trial WB-MRI findings

MDT discussion of the first major treatment decision based on conventional imaging

WB-MRI images and reports will be withheld initially from the clinical care team and radiologists reporting standard imaging (to avoid bias) until patients have completed all conventional investigations and have been definitively staged such that a first major treatment decision has been made by the MDT based on these conventional tests. The first major treatment decision based on conventional imaging will be defined as:

- Referral for surgical excision of either the primary tumour and/or a metastatic site

For peer review only - http://bmjopen.bmj.com/site/about/guidelines.xhtml

- Instigation of definitive treatment using chemotherapy, radiotherapy or a combination of the two
- Decision to offer palliative/supportive care only
- Request for a highly invasive surgical staging procedure such as surgical mediastinal lymph node sampling (mediastinoscopy), video-assisted thoracoscopic surgery (VATS), or laparoscopy

If additional conventional tests are required to complete staging, the WB-MRI result will be withheld until they are completed, and the patient re-discussed with the findings of the additional test(s).

The nature, date and findings of all standard investigations will be recorded by the MDT, and the TNM 7 stage recorded. The MDT will define the nature of the first treatment decision based on conventional investigations in the MDT record and on the CRF.

Release of WB-MRI images and report

Once a stage and treatment decision has been made based on conventional imaging, the WB-MRI images (supplemented where possible with "screen shots" and annotations of relevant findings) and radiologist's report will be revealed to the MDT using the 3Dnet™ software on an internet enabled PC. If technical issues preclude use of 3Dnet™ in the MDT, presentation of a written copy of the WB-MRI report is permissible.

When the WB-MRI images and report are released, the MDT will discuss the images and decide:

1) *If the patient can be adequately staged based on WB-MRI alone:*

   In this case the MDT should state the patient's stage and theoretical treatment decision based on the WB-MRI alone.

Or:

2) *If any additional tests are required based on the WB-MRI alone for adequate staging* (for example for equivocal WB-MRI findings):
   a. For additional tests that would have theoretically been requested based on the WB-MRI alone but have already been performed as part of the standard imaging, the results can be used by the MDT. The MDT personnel will state the patient's stage and theoretical treatment decision based on the WB-MRI and those additional tests that would have theoretically been generated.
   b. If the additional tests generated by WB-MRI have not already been performed as part of standard care and are deemed necessary in the opinion of the MDT these will be undertaken according to standard clinical care. After completion of the additional test(s), the patient will be re-discussed by the MDT and a final treatment decision made based on the WB-MRI and the additional test(s) undertaken.

For peer review only - http://bmjopen.bmj.com/site/about/guidelines.xhtml

8.2.4. Final treatment plan

Once a stage and treatment decision has been made based on conventional imaging and also a stage and theoretical treatment decision made based on the WB-MRI alone (and any additional tests) then a final treatment plan can be made by the MDT. The final treatment plan for the patient will be based on all available information including the WB-MRI. The MDT radiologist(s) will be at liberty to review the WB-MRI data after the WB-MRI has been revealed in order to review its findings and feedback to the clinical care team.

The completed MDT CRF will thus list:

- Stage and treatment decision based on conventional investigations (and the number, timing, nature and findings of these investigations).
- Stage and theoretical treatment decision based on WB-MRI (and any additional tests generated by the WB-MRI) alone (and the number, timing, nature and findings of additional tests generated).
- A Final treatment decision incorporating all available tests.

Once the WB-MRI images and report have been revealed to the MDT according to trial protocol, they can be released onto the relevant hospital PACS server using the 3Dnet™ gateway described above or other IT solution (e.g. image exchange portal) and be made freely available to those providing subsequent patient care, as per usual clinical practice.

8.2.5. Early release of WB-MRI findings

Any requests for early WB-MRI release must be made to the site PI, who will discuss the request with the lead clinician who is responsible for the patient's clinical care (if the request did not originate from the lead clinician). After discussion, if it is decided by the PI that release of the WB-MRI images and report is necessary, the PI (or a designated member of the trial team) will inform UCL CTC. The WB-MRI result should only be released early in the event of an emergency situation in which the patient is unfit to undergo additional investigations (including MRI) which would have normally been performed were the patient not in the Streamline L trial, and the WB-MRI may have a direct effect on immediate patient care. In the event of early release, the patient will be replaced, although the findings of the WB-MRI and conventional staging (performed up to the time of early MRI release) will be collated by UCL CTC on the standard trial CRFs for subsequent reporting in the trial publications.

Early release for important clinical findings

The time between the patient's diagnosis and the WB-MRI reveal is typically less than 4 weeks at the proposed recruitment sites. The majority of findings on WB-MRI will have no direct impact on patient care during this time. However should WB-MRI reveal a serious finding which could have an immediate impact on direct patient care before the MDT, the reporting radiologist will contact the patient's clinician to discuss the finding and a decision will be made as to whether the results should be revealed early to all members of the clinical team (based on review of standard tests already performed which may also have detected the finding). Specific findings which will trigger this review are:

- Impending spinal cord compression

For peer review only - http://bmjopen.bmj.com/site/about/guidelines.xhtml

- Deep vein thrombosis or pulmonary embolism
- Brain metastasis with significant mass effect requiring immediate treatment

It is unlikely the patient would be asymptomatic in any of these scenarios, and it is probable additional imaging tests would have already been requested as part of usual clinical care.

Early release for urgent patient management

Although unlikely, it is possible there may be a need to access the WB-MRI report urgently before its release to the MDT, for example if a recruited patient presents to hospital with collapse and knowledge of the WB-MRI findings would potentially change patient management. As noted above, imaging hubs will keep a copy of the free text report of the WB-MRI and this will be made available if requested by clinical teams in this scenario.

8.2.6. Time to full staging

Standard pathway

The total time required to fully stage the patient using standard imaging pathways will be calculated. The start of the staging process will be defined as the date of request of the first staging investigation following a proven or assumed diagnosis of lung cancer (for example date of requested PET/CT after CT or biopsy diagnosis of likely lung tumour). The completion of staging will be defined as the date when the MDT/ appropriate MDT personnel made the first major treatment decision based on the standard imaging.

WB-MRI

Because revelation of the result of WB-MRI is deferred until after standard staging is complete, actual measurement of the time to full staging using WB-MRI will not be possible. The theoretical time to complete staging using WB-MRI will be thus modelled taking into consideration the time from recruitment date to the date of the WB-MRI, plus the number and type of additional staging investigations generated by WB-MRI.

8.2.7. Patient follow up

Patients will be followed for a period of 12 months from the date of recruitment. Follow up will not require any trial specific patient visits to the recruiting site, and follow up CRFs can be completed using hospital databases or patient notes.

For all recruited patients the final TN tumour stage according to the 7th classification will be recorded based on histopathological analysis of surgical specimens (if surgery is performed). The results of any biopsy procedure undertaken over the 12 months will also be recorded. The date, nature and findings of follow up imaging investigations will be recorded for each recruited patient, in particular the presence or absence of metastasis.

The date and cause of patient death and post mortem findings (if performed) will also be recorded.

For peer review only - http://bmjopen.bmj.com/site/about/guidelines.xhtml

Use will be made of MDT records and hospital data repositories to collate this data, and data collection will be coordinated by designated individuals at each recruitment site, aided by UCL CTC.

8.2.8. Patient diaries and EQ-5D Quality of Life questionnaires

All patients will receive a Diary and EQ-5D Quality of Life questionnaire every 3 months which will be posted by UCL CTC. Upon consent the recruiting site must inform the patient that they will receive an EQ-5D questionnaire and Diary through the post from UCL CTC and they are to complete these and return them using the pre-paid envelope provided. The patient diary will cover a 3 month period and will collect information regarding the patient's Primary Health Care contacts, other health care contacts and also the medication they are currently taking. Patient diaries and EQ-5D questionnaires will be posted to all patients at consent and then at 3 monthly intervals for 12 months.

8.2.9. Final reference standard for tumour stage (Consensus Meetings)

Multi-disciplinary consensus panel review is standard methodology for diagnostic test accuracy studies where an independent reference standard is impossible because of incorporation bias. Consensus panels will convene annually to derive the reference standard for tumour stage at diagnosis for recruited patients completing the first 12 month follow up (i.e. a panel at the end of year 2 will consider patients recruited in year 1, a panel at the end of year 3 will consider patient recruited in year 2 etc.). The panels will consider all available clinical information including the results of all original staging investigations, WB-MRI, histopathology (surgical resection and biopsies), follow up imaging and post-mortem reports (where available) and MDT records. UCL CTC will coordinate collation of these data via submitted CRFs over the preceding 12 months for presentation to the panel. Each imaging hub or recruitment site will host a consensus panel to consider patients undergoing WB-MRI (or recruited) at its site (so all imaging studies are available for review on local PACS systems if required). Each panel will consist of at least an oncologist, and/or chest physician, and 3 radiologists, 1 external to the imaging hub and 2 internal: 1 with specific expertise in WB-MRI and 1 with expertise in PET (a single radiologist is acceptable if they have expertise in WB-MRI and PET). The panel will have access to a histopathologist if required. Each panel will adjudicate on the TMN stage of the cancer at diagnosis, including the organ specific sites and burden of metastatic spread.

The definition of the presence or absence of metastasis will be assigned for each organ on a designated CRF. The designation will be made in consensus by all on the panel. Consideration will be made to histology in all biopsied lesions. In the absence of histological proof of metastasis, metastatic disease will be assumed if new lesions appear during the 12 month follow up with imaging characteristics compatible with metastasis and no alternative explanation, or if lesions with characteristics compatible with metastasis which either grow or shrink (on therapy). Lesions identified which remain stable over the 12 month follow up period will assumed not to be metastatic unless there are specific circumstances considered by the panel that indicate malignancy (e.g. change in lesion morphology with treatment).

For peer review only - http://bmjopen.bmj.com/site/about/guidelines.xhtml

All “new” sites of metastatic disease diagnosed in the 12 month follow up will be assessed by the panel to see if they were visible in retrospect at diagnosis. Although the primary analysis by the original reporting radiologist will be that used to define test accuracy, this retrospective review will allow definition of the rates of perceptual error.

For patients in whom the primary tumour has been completely removed within 3 months of diagnosis, all new metastatic sites will be assumed to have been present at diagnosis for the purposes of calculating test sensitivity.

If the primary tumour is left in situ for more than 3 months (or there is incomplete removal), any new diagnosed sites of metastatic disease will be assumed to have been present at diagnosis if identified within 6 months of diagnosis of the primary tumour. If they are diagnosed beyond 6 months of diagnosis, and there is no evidence of their presence on retrospective review of all staging investigations, they will be assumed to be new disease not present at diagnosis.

If patients with tumours left in situ do not undergo any imaging capable of detecting metastatic disease within 6 months of diagnosis of the primary tumour (other than initial staging tests) and imaging beyond this identifies metastatic disease not visible in retrospect on any trial imaging, the consensus panel will opine if the disease was likely present at diagnosis, based on its location, size and imaging characteristics.

If a patient dies before the 12 month follow up, the panel will review all available imaging, histopathology and clinical course prior to death and in consensus state if a confident diagnosis of the presence or absence of metastatic disease can be made (for example imaging characteristics compatible with metastasis and no alternative explanation, or if lesions with characteristics compatible with metastasis which either grow or shrink (on therapy). If this judgement cannot be made with confidence (for example if the patient has equivocal lesions on staging investigations and no further follow up), these patients will not be excluded but multiple imputation will be used to account for missing data. A sensitivity analysis for the primary outcome, where only patients with complete data are analysed, will exclude these patients.

8.2.10. Health psychology assessment

Study 1: Patient interviews (approximate n=25)

Target accrual has now been reached for the patient experience interview part of the trial and so recruitment to this part is now closed. Individual in-depth interviews were conducted by a health psychologist pertaining to their experience of the cancer staging process. The aim of the interviews is to determine patients’ experience of WB-MRI, standard tests and those tests generated by WB-MRI. We collected simple demographic data from participants in relation to gender, age and educational level as such factors may influence experience and preferences, and this data will enable us to determine whether the sample of people who are interviewed are representative of the sample of people who also participate in studies 2 and 3 (see below). The interviews assessed which aspects of testing caused patients physical or psychological stress (e.g. number of tests/hospital visits, test attributes (i.e. physical experiences of the tests such as claustrophobia, need to lie still, scanner noise etc.), total length of time taken, additional tests generated by initial scan findings) and elicited any factors patients felt would have made staging easier for them.

For peer review only - http://bmjopen.bmj.com/site/about/guidelines.xhtml

The interviews provided in-depth qualitative data about the experience of cancer staging and in particular undergoing WB-MRI, and has informed the modification of a questionnaire (study 2) designed to assess patient acceptability and experience of WB-MRI compared to standard tests, and also has informed the design of a discrete choice experiment (study 3).

Study 2: Patient questionnaires (approximate n=50)

Target accrual has now been reached for the patient experience questionnaire part of the trial and so recruitment to this part is now closed. Follow up questionnaires will continue to be posted until the last patient's month 6 time point occurs. A questionnaire was developed and sent to consenting patients to assess their experience and acceptability of WB-MRI and standard tests (e.g. PET-CT). Final design was informed by analysis of the qualitative data collected in study 1 (See above). Patient Experience Questionnaire content is outlined in Table 3 and includes patients' views about test preparation (e.g. fasting), and experience of the scan itself (e.g. discomfort, claustrophobia, fear etc.). Patients were also asked to rate the importance of various attributes associated with competing staging pathways (e.g. waiting time from diagnosis to treatment, total test number, test accuracy etc.). This rating identified those attributes considered most important by patients, which has been included in the discrete choice experiment (study 3).

Patients were given the opportunity to opt in to completing the questionnaires when they were recruited into the trial. Questionnaires, together with the positive and negative affect schedule (PANAS) and the General Health Questionnaire (GHQ-12) were administered during and after staging pathways, and at 3 and 6 months later to examine patient test preferences once they are aware of their cancer stage and knew which of the tests they received were more accurate as well as the burden placed on them by any extra tests they needed as a result of WB-MRI.

Patients were posted the baseline questionnaire along with pre-paid reply envelope at the time they were recruited into the trial. The questionnaire was worded to take into account the fact that some patients had not yet had any staging investigations. Patients were posted the post-staging and 3 and 6 month follow up questionnaires along with pre-paid envelopes at the relevant time points.

Study 3: Discrete choice experiment (approximate n=50)

Discrete choice experiments (DCE) can elicit preferences for different types of health care provision [18] by estimating the relative importance of different attributes, and the trade-offs between them [19]. WB-MRI and conventional staging pathways differ in associated attributes, not only related to physical experience but also rate of adverse events, time to diagnosis, overall accuracy etc. The most important attributes have been identified in studies 1 and 2 and appropriate levels assigned to each based on accumulating data from the trial, together with appropriate literature review. A DCE questionnaire has been developed whereby patients state their preference between two choices, with each choice containing different levels of the identified attributes.

The DCE questionnaire is optional and patients will be asked to consent to complete the questionnaire on trial entry. Once a patient consents to the DCE they will be allocated a DCE questionnaire (out of a possible 2 DCE questionnaires) which will be posted by UCL CTC immediately after registration (baseline) along with their QoL

For peer review only - http://bmjopen.bmj.com/site/about/guidelines.xhtml

questionnaire, patient diary and pre-paid envelope. UCL CTC will supply the details of any recruited patients who consented to complete the DCE questionnaire to the Health psychologists who may contact them directly.

Table 2: Questionnaire schedule.

Questionnaires will be posted to patients by UCL CTC.

	Baseline (peri-staging)	Months after registration		
		1	3	6
Demographic measures	✔			
Co-morbidities	✔			
Self-rated health	✔			
Symptoms of lung cancer	✔			
Concern for future consequences	✔			
Which staging tests have had	✔	✔		
Perceived importance of test attributes	✔	✔		
Perceived acceptability of different tests	✔	✔		
Least acceptable aspects of different tests	✔	✔		
Agreement to having tests again (short and long-term)	✔	✔		
Single test choice	✔	✔		
Test recovery time	✔	✔		
Patient experience of WB-MRI and standard tests	✔	✔		
Perceived efficacy of WB-MRI and standard tests		✔		
Difficulties with completing scan (WB-MRI and standard tests)		✔		
Ranked attribute preferences	✔	✔		
Feelings about number of tests			✔	✔
EQ-5D	✔		✔	✔
PANAS	✔	✔	✔	✔
GHQ-12	✔	✔	✔	✔

For peer review only - http://bmjopen.bmj.com/site/about/guidelines.xhtml

8.2.11. Cost Effectiveness Assessment

Overview

We will undertake a detailed analysis of the cost and the cost-effectiveness of WB-MRI versus standard NICE-approved staging algorithms. The analysis will conform to accepted economic evaluation methods [15]. All costs will be assessed from the perspective of the NHS and personal social services (PSS).

Making comparisons between WB-MRI versus standard staging algorithms

The care pathway for lung cancer patients can both be divided into two stages, the *treatment decision pathway* and the *subsequent disease pathway*. The former includes the time from diagnosis to treatment decision by the MDT; the latter includes the time period following the treatment decision.

The treatment decision pathway will be different between WB-MRI and standard staging algorithms, yielding different costs and potentially different treatment decisions. In patients for whom the treatment decision with WB-MRI is the same as that with conventional staging algorithms, the subsequent disease pathways will be the same. Where the treatment decision with WB-MRI is different, the disease pathway will be different, yielding potentially different costs and health outcomes.

If in the patients studied the concordance between the treatment decisions associated with WB-MRI and conventional staging algorithms is high, then the economic analysis can focus on the cost of the treatment decision pathways only because the disease pathways will be no different. In this case the cost-effectiveness of WB-MRI versus conventional staging algorithms depends only on the incremental cost (positive or negative) of WB-MRI versus standard staging algorithms in the treatment decision pathway.

Conversely, if the concordance between the treatment decisions is low, then the economic analysis ought to focus on both the treatment decision pathways and the subsequent disease pathways because both of these will vary between WB-MRI and conventional staging algorithms. In this case the cost-effectiveness of WB-MRI depends on the incremental cost of the WB-MRI versus standard staging algorithms in the treatment decision pathway plus the incremental costs and health benefits of the disease pathway.

The precise nature of the economic analysis will therefore depend on the degree of concordance between treatment decisions provoked by WB-MRI versus conventional staging.

Discordance of major treatment decisions

Discordance will be defined when the first major treatment decision differs between WB-MRI and standard investigations in greater than 10% of patients. Concordance will be defined as the absence of discordance. For lung cancer we define a major difference in treatment decision as occurring when only one pathway suggests the patient is a surgical candidate or suitable for radical radiotherapy. A 3x2 table will be constructed according to decision to perform surgery, use radical radiotherapy or not based on WB-MRI vs. standard diagnostic pathways.

For peer review only - http://bmjopen.bmj.com/site/about/guidelines.xhtml

Scenario 1: there is concordance between the treatment decisions associated with WB-MRI and standard staging

In this case, the cost components included in the analysis will be:

- Conventional tests for staging of disease among diagnosed patients (e.g., PET/CT/EBUS/TBNA/USS neck for lung cancer, plus additional tests as indicated by the conventional staging algorithm);
- Costs of treating adverse events associated with staging tests;
- WB-MRI, plus additional tests generated by WB-MRI;
- MDT meetings.

The volume of resource use for each cost component will be measured directly in the trial from treatment decisions recorded in MDT reports, based first on conventional staging alone and then based on WB-MRI alone, and on patient records included in the trial. Unit costs will be taken from standard published sources. Since the two algorithms yield the same treatment decisions cost-effectiveness depends on the incremental cost of WB-MRI versus conventional staging algorithms in the treatment decision pathway (i.e., formally this is a cost-minimisation analysis).

Scenario 2: there is discordance between the treatment decisions associated with WB-MRI and standard staging

In this case, cost-effectiveness depends on the incremental cost (positive or negative) of the treatment decision pathway and disease pathway associated with WB-MRI versus conventional staging and the incremental health benefits (positive or negative). We will calculate cost-effectiveness in terms of the incremental cost per quality-adjusted life year (QALY) gained using one year and lifetime time horizons.

For the analysis based on the one-year time horizon, the trial will provide information on the treatment decisions arising from WB-MRI and standard staging and follow up data for the first year. We will collect these data from three sources.

Firstly for all patients in the trial we will collect the resource use data for the main drivers of hospital costs using a trial specific CRF. This will collect resource use data on the following cost components for each patient:

- Imaging investigations
- Chemotherapy
- Radiotherapy
- Surgery and biopsies
- Outpatient visits
- Inpatient stays
- Day cases

These data will be recorded prospectively based mainly on MDT consensus meeting recommendations and on patient records (for major changes in treatment). Unit costs will be taken from standard published sources and applied to the resource use data, allowing us to cost the care received by each patient.

For peer review only - http://bmjopen.bmj.com/site/about/guidelines.xhtml

In addition, from this source we will collect data on whether or not the patient died during the follow up period, and if they died, the date of death.

Secondly for all patients in the trial we will prospectively collect resource use data using patient diaries. This will supplement the above data which may not provide a complete picture of hospital resource use, plus it will allow us to collect data on primary and community care contacts. These will record resource use data on the following cost components for each patient:

- Medications taken
- Day cases
- GP contacts
- Practice and community nurse contacts
- A&E Visits
- Any other primary care or community care contacts related to cancer

The diaries will be posted out by UCL CTC at baseline, 3, 6, and 9 months and patients will be asked to complete them for the following 3 month period.

In addition we will also collect data on health-related quality of life score, measured according to the EQ-5D (www.euroqol.org) which will be measured at baseline and at 3, 6, 9 and 12 months for all surviving patients.

UCL CTC will contact sites just prior to sending out any post registration diaries or questionnaires to check the status of the patient to ensure none are sent inappropriately to deceased patients.

Patients who are found not to have cancer or are withdrawn for other reasons (see section 13), will not be sent diaries, quality of life or patient experience questionnaires to complete.

Thirdly the two sources of resource use data described above will be supplemented with a review of resource data use collected in the recently completed Lung-BOOST trial. This may be supplemented by retrospective review of resource use (including imaging, outpatient visits, inpatients stays and medication use) for the 12 months post diagnosis of up to 150 patients diagnosed with lung cancer at UCLH. This retrospective review will be carried out by a member of the clinical team and according to guidance provided by the UCLH Caldicott guardian. All collated retrospective data will be fully anonymised before sharing with other trial researchers outside the clinical care team.

8.2.12. Nested substudies

1) Diagnostic performance of limited WB-MRI protocols based on diffusion and T1 weighted imaging only, and to assess the incremental benefit of intravenous gadolinium contrast enhancement.

   As noted above, WB-MRI will be analysed using a locked sequential viewing paradigm such that the diagnostic accuracy of Diffusion and non contrast enhanced T1 images, Diffusion, non contrast enhanced T1 and T2 images, and Diffusion, non contrast enhanced T1, T2 and contrast enhanced T1 images can be calculated.

For peer review only - http://bmjopen.bmj.com/site/about/guidelines.xhtml

2) Effect of radiologists' diagnostic confidence on the accuracy of WB-MRI.

   Radiologists will indicate their level of confidence for the presence or absence of metastasis on a 1-6 scale on the CRF recording their interpretation of the WB-MRI.

3) Inter-observer variation in WB-MRI interpretation.

   Each hub radiologist (n=6) will interpret a sample of 25 WB-MRI datasets selected at random from the other imaging hubs to define inter-observer variation in the reported presence absence of metastatic disease.

4) The collated WB-MRI datasets and 12 month patient follow up data collated as part of the trial protocol provides opportunities to retrospectively mine the data for additional diagnostic indices and prognostic markers (See appendix 4). The following or similar may be quantified in all or a subset of whole body MRI datasets:

   - Body fat (organ, visceral and whole body)
   - MRI signal from the primary tumour and metastatic sites, notably diffusion weighted signal, apparent diffusion coefficient, T1 (including fat and water components), T2 signal, Contrast enhancement, and textural analysis (for heterogeneity) of the primary and metastatic deposits on the various sequences.

For peer review only - http://bmjopen.bmj.com/site/about/guidelines.xhtml

9. Data Management Guidelines

Data will be collected from sites on version controlled case report forms (CRFs) designed for the trial and supplied by UCL CTC. Data entered onto CRFs must reflect source data at site. Some data will be recorded directly on the CRFs (i.e. no prior written or electronic record of data), and it will be considered to be source data. This will be made clear in the CRF guidance.

Where supporting documentation (e.g. autopsy reports, pathology reports, CT scan images etc.) is being submitted to UCL CTC, the patient's trial number must be clearly indicated on all material and any patient identifiers removed/blacked out prior to sending to maintain confidentiality.

9.1. Completing Case Report Forms

All CRFs must be completed and signed by staff who are listed on the site staff delegation log and authorised by the PI to perform this duty. The PI is responsible for the accuracy of all data reported in the CRF.

All entries must be clear, legible and written in ball point pen. The use of abbreviations and acronyms must be avoided.

Any corrections made to a CRF at site must be made by drawing a single line through the incorrect item ensuring that the previous entry is not obscured. Each correction must be dated and initialled. Correction fluid must not be used.

The use of abbreviations and acronyms must be avoided.

Once completed the original CRFs must be sent to UCL CTC and a copy kept at site.

9.2. Missing Data

To avoid the need for unnecessary data queries CRFs must be checked at site to ensure there are no blank fields before sending to UCL CTC. When data are unavailable because a measure has not been taken or test not performed, enter "ND" for not done. If an item was not required at the particular time the form relates to, enter "NA" for not applicable. When data are unknown enter the value "NK" (only use if every effort has been made to obtain the data).

9.3. Timelines for data return

CRFs must be completed at site and returned to UCL CTC as soon as possible and within 4 weeks of scheduled time point. For further details, see CRF instructions.

Sites who persistently do not return data within the required timelines may be suspended from recruiting further patients into the trial by UCL CTC and subjected to a 'for cause' monitoring visit. See section 12.2 ('For cause' on-site monitoring) for details.

For peer review only - http://bmjopen.bmj.com/site/about/guidelines.xhtml

9.4. Data Queries

Data arriving at UCL CTC will be checked for legibility, completeness, accuracy and consistency, including checks for missing or unusual values. Query reports will be sent to the data contact at site. Further guidance on how data contacts should respond to Data Queries can be found on the Query Reports.

For peer review only

For peer review only - http://bmjopen.bmj.com/site/about/guidelines.xhtml

10. Safety Reporting

Clinical Review

The MRI Imaging Hub and/or Recruiting site will record any complications attributable to the WB-MRI or any additional test performed as a result of the WB-MRI, notably contrast reactions, biopsy complications (infection, bleeding or hospital admission), and treatment target breaches on the Staging Complications CRF which will be submitted to UCL CTC.

UCL CTC will provide trial data to the CI on a periodic basis for review. If it is declared necessary to revise the conduct of the trial due to safety concerns, UCL CTC will inform the REC as appropriate.

For peer review only

For peer review only - http://bmjopen.bmj.com/site/about/guidelines.xhtml

11. Incident Reporting

Organisations must notify UCL CTC of all deviations from the protocol or GCP immediately. UCL CTC may require a report on the incident(s) and a form will be provided if the organisation does not have an appropriate document (e.g. Trust Incident Form).

If site staff are unsure whether a certain occurrence constitutes a deviation from the protocol or GCP, the UCL CTC trial team can be contacted immediately to discuss.

UCL CTC will use an organisation's history of non-compliance to make decisions on future collaborations.

For peer review only

For peer review only - http://bmjopen.bmj.com/site/about/guidelines.xhtml

12. Trial Monitoring and Oversight

Participating sites and PIs must agree to allow trial-related on site monitoring, Sponsor audits and regulatory inspections by providing direct access to source data/documents as required. Patients are informed of this in the patient information sheet and are asked to consent to their medical notes being reviewed by appropriate individuals on the consent form.

UCL CTC will determine the appropriate level and nature of monitoring required for the trial. Risk will be assessed on an ongoing basis and adjustments made accordingly.

12.1. Central Monitoring

Sites will be requested to submit relevant logs to UCL CTC at the frequency detailed in the trial monitoring plan or on request and these will be checked for consistency and completeness. Also refer to sections 4.2.2 (Required Documentation) and 6.2 (Screening Logs).

Ensuring patient eligibility is the responsibility of the PI or other delegated Investigator(s). Checks of the criteria listed on the registration form will be undertaken by an appropriately trained UCL CTC staff member prior to registration. Also refer to section 7.1 (Registration).

Details relating to the informed consent process will be collected on the registration form and are subject to review by UCL CTC as part of patient eligibility.

Data received at UCL CTC will be subject to review in accordance with section 9.4 (Data Queries).

Where central monitoring of data and/or documentation submitted by sites indicates that a patient may have been placed at risk, the matter will be raised urgently with site staff and escalated as appropriate (refer to section 11 (Incident Reporting) and 12.2 ('For cause' on site monitoring) for further details).

12.2. 'For Cause' On Site Monitoring

On site monitoring visits may be scheduled at a site where there is evidence or suspicion of non-compliance with important aspect(s) of the trial protocol/GCP requirements. Sites will be sent a letter in advance outlining the reason(s) for the visit. The letter will include a list of the documents that are to be reviewed, interviews that will be conducted, planned inspections of the facilities, who will be performing the visit and when the visit is likely to occur.

Following a monitoring visit, the Trial Monitor/Trial Coordinator will provide a report to the site, which will summarise the documents reviewed and a statement of findings, deviations, deficiencies, conclusions, actions taken and actions required. The PI at each site will be responsible for ensuring that monitoring findings are addressed in a timely manner, and by the deadline specified.

For peer review only - http://bmjopen.bmj.com/site/about/guidelines.xhtml

UCL CTC will assess whether it is appropriate for the site to continue participation in the trial. Refer to section 11 (Incident Reporting) for details.

12.3. Oversight Committees

12.3.1. Trial Management Group (TMG)

The TMG will include the Chief Investigator, clinicians and experts from relevant specialities and Streamline L trial staff from UCL CTC (see page 3). The TMG will be responsible for overseeing the trial. The group will meet regularly at least once a year and will send updates to PIs (via newsletters or at Investigator meetings) and to the NCRI Lung Clinical Studies Group.

The TMG will review substantial amendments to the protocol prior to submission to the REC. All PIs will be kept informed of substantial amendments through their nominated responsible individuals.

TMG members must sign a charter confirming acceptance of their responsibilities.

12.3.2. Trial Steering Committee (TSC)

The role of the TSC is to provide overall supervision of the trial. The TSC will review the recommendations of the Independent Data Monitoring Committee and, on consideration of this information, recommend any appropriate amendments/actions for the trial as necessary. The TSC acts on behalf of the funder and Sponsor.

TSC members must sign a charter confirming acceptance of their responsibilities and declaring any potential conflicts of interest.

12.3.3. Independent Data Monitoring Committee (IDMC)

The role of the IDMC is to provide independent advice on data and safety aspects of the trial. Meetings of the Committee will be held at least once a year or as necessary to address any issues. The IDMC is advisory to the TSC and can recommend premature closure of the trial to the TSC.

IDMC members must sign a charter confirming acceptance of their responsibilities and declaring any potential conflicts of interest.

12.3.4. Role of UCL CTC

UCL CTC will be responsible for the day to day coordination and management of the trial and will act as custodian of the data generated in the trial (on behalf of UCL). UCL CTC is responsible for all duties relating to safety reporting which are conducted in accordance with section 10 (Safety Reporting).

For peer review only - http://bmjopen.bmj.com/site/about/guidelines.xhtml

13. Withdrawal of Patients

In consenting to the trial, patients are consenting to the WB-MRI, and also specifically consenting to completing questionnaires and diaries.

<u>Losses to follow up</u>

If a patient moves from the area, the site should make every effort to ensure the patient is followed up at another participating trial site and for this new site to take over the responsibility for the patient, or for follow up via GP. Details of participating trial sites can be obtained from the UCL CTC trial team who must be informed of the transfer of care and follow up arrangements.

Patients should be considered lost to follow up only once documented efforts on the part of the site have failed to produce any response or information from the patient or GP over the course of one year.

If a patient is lost to follow up at a site every effort should be made to contact the patient's GP to obtain information on the patient's status. If the patient cannot be contacted this should be recorded on the Change of Status CRF.

<u>Patients Withdrawing Consent</u>

If a patient expresses their wish to withdraw from the trial, sites should explain the importance of allowing routine follow up data to be used for trial purposes and for allowing existing collected data to be used. If the patient gives a reason for their withdrawal, this should be recorded (see below).

Withdrawal of Consent to Future Data Collection and use of past data

If a patient explicitly states they do not wish to contribute further data to the trial or allow the use of past data, their decision must be respected and recorded on the Change of Status CRF. In this event details should be recorded in the patient's hospital records, no further CRFs must be completed and no further data sent to UCL CTC.

Withdrawal of Consent to QoL, Resource use diary and/or Patient experience questionnaires

If a patient withdraws consent from completing the QoL, Resource use diary and/or the Patient experience questionnaires their decision must be recorded on the Change of Status CRF. UCL CTC will not post any further documents to the patient. However, the patient may continue to participate in the trial, and trial follow up completed as per protocol.

For peer review only - http://bmjopen.bmj.com/site/about/guidelines.xhtml

14. Trial Closure

14.1. End of Trial

For regulatory purposes the end of the trial will be 1 year after recruitment of the final patient at which point the 'declaration of end of trial' form will be submitted to ethical committees, as required.

Following this, UCL CTC will advise sites on the procedure for closing the trial at the site.

14.2. Archiving of Trial Documentation

At the end of the trial, UCL CTC will archive securely all centrally held trial related documentation for a minimum of 5 years. Arrangements for confidential destruction will then be made. It is the responsibility of PIs to ensure data and all essential documents relating to the trial held at site are retained for a minimum of 5 years after the end of the trial, in accordance with national legislation and for the maximum period of time permitted by the site.

Essential documents are those which enable both the conduct of the trial and the quality of the data produced to be evaluated and show whether the site complied with the principles of GCP and all applicable regulatory requirements.

UCL CTC will notify sites when trial documentation held at sites may be archived. All archived documents must continue to be available for inspection by appropriate authorities upon request.

Health Psychology data collected as part of this research will be retained for 10 years in line with Birkbeck, University of London's records management policy. At the end of this time period the documents will be shredded. The recordings of the interviews will be destroyed after they have been transcribed and verified.

14.3. Early discontinuation of trial

The trial may be stopped before completion as an Urgent Safety Measure on the recommendation of the TSC or IDMC (see section 12.3.2 TSC and 12.3.3 IDMC). Sites will be informed in writing by UCL CTC of reasons for early closure and the actions to be taken with regards the treatment and follow up of patients.

14.4. Withdrawal from trial participation by a site

Should a site choose to close to recruitment the PI must inform UCL CTC in writing. Follow up as per protocol must continue for all patients recruited into the trial at that site and other responsibilities continue as per Clinical Trial Site Agreement (CTSA).

For peer review only - http://bmjopen.bmj.com/site/about/guidelines.xhtml

15. Quality Assurance

The technical quality of at least 10% the WB-MRIs from each imaging hub will be assessed by the trial radiographer at UCLH. Using the 3Dnet™ software, the radiographer will access performed scans on a weekly basis and enter an audit score of quality according to the definitions below. A report of the quality will be provided to the CI every 2 weeks and to the TMG at each meeting.

Technical quality – general

1 = More than one sequence with substantial degradation of images severely limiting interpretation of those sequences, and not repeated

2 = One sequence with substantial degradation of images severely limiting interpretation of that sequence, and not repeated

3 = More than one sequence has minor artefact, but all remain fully diagnostic and repeat although optimal, not necessary OR all sequences initially technically inadequate (score 1 or 2) correctly repeated

4 = One sequence a has minor artefact, but remains fully diagnostic and repeat, although optimal, not necessary

5 = All sequences technically optimal with no artefact or degradation

Technical quality – anatomical coverage

1 = Wrong examination performed

2 = More than one sequence does not adequately cover the body (skull to mid thigh), or designated organ(s) coverage

3 = One sequence does not optimally cover the body or designated organ(s) coverage but examination remains fully diagnostic

4 = All sequences optimally cover the body and designated organ(s) coverage

For peer review only

For peer review only - http://bmjopen.bmj.com/site/about/guidelines.xhtml

16. Statistics

16.1. Sample size calculation

SAMPLE SIZE: Studies of diagnostic test accuracy are based on a cohort design where all patients receive all main diagnostic tests (PET/CT or WB-MRI), although additional tests ordered in each staging pathway differ between patients, based on clinical appropriateness. Existing pathways and therapeutic decision making is complex for lung cancers (see flow chart). This NIHR HTA trial focuses on first definitive staging and we are powered at this point in the patient pathway, informed by a detailed literature review.

Multiple therapeutic options are contingent on diagnosis of metastatic disease.

We therefore power on a change in sensitivity for metastases detection with a WB-MRI pathway as a replacement to the standard NICE staging pathway.

The premise of the trial power is to detect a higher sensitivity for extra thoracic disease (specifically brain and bone metastasis) with WB-MRI in comparison to standard imaging paradigms. To power on equivalence or non-inferiority would be well beyond pragmatic recruitment and funding.

Based on recent meta-analysis data [10] considering 1874 patients, the overall prevalence of brain metastasis at presentation in NSCLC is 13% (6% to 32%) and bone 20% (8-34%). Around 20% have metastasis at multiple sites [1, 4, 17, 20].

Prevalence assumptions

Conservative estimate of the prevalence of brain metastasis at presentation is 10%.

20% of these patients are already identified as having metastatic disease via detection of co-existing extra cranial disease on pre-diagnosis CT (e.g. liver, adrenal).

A further 10% of patients will have disease at more than one site on CT (and therefore more likely detected by PET CT than if isolated to the brain).

Overall 12% (n=40) of the hypothetical 325 patients with potentially operable disease will have isolated brain metastasis and 5% (n=16) have bone metastasis. The remaining 8% (of the total 25% with undiagnosed extra-thoracic metastatic disease) will have metastasis at other organ sites (e.g. lung, liver adrenal).

Sample size calculation

Comparison of WB-MRI to PET & PET/CT accuracy - both against reference standard

- Paired trial design – comparing two diagnostic pathways where both are used in all patients.
- Sample size method for difference in paired proportions [21].
- Power trial to show difference in sensitivity, as fewer patients are with metastasis than without. Trials powered like this should also be suitable for difference in specificity.
- 80% power type II error, type I error 5% ($p<0.05$).

For peer review only - http://bmjopen.bmj.com/site/about/guidelines.xhtml

Streamline L

- Assume ratio of marginal cells of 2 x 2 table comparing WB-MRI to PET: s/t = 9 to 11 (tables 3 and 4)
- Assume sum of marginal cells as proportion of total with disease: s+t/N=0.24
- A sample size of 50 patients with occult metastasis is needed, from a population of 25% prevalence of metastasis. Thus a total sample size of 200 (50 times 4) is required.

200 patients with no metastasis at lung cancer diagnosis and potential surgical candidates would be required to detect a difference of 24% in sensitivity of WB-MRI for metastatic disease (79%) compared to conventional staging (55%) given site specific disease prevalence as described above.

The sample size above must be adjusted to account for “withdrawals”. Patients are classified as withdrawn if they are recruited but are not evaluable for any of the following reasons (i) they do not have an WB-MRI scan, the main component of the primary outcome (ii) they do not have cancer (iii) they are lost to follow up before 1 year and do not have sufficient information for the expert panel to evaluate whether metastases were present.

The withdrawal rate was expected to be 20%, giving a target recruitment of 250 patients. However, the actual withdrawal rate has been 43% and the target recruitment has therefore been revised to 353 patients.

Table 3: Calculations comparing WB-MRI to PET

		PET & PET/CT		
	Result	+	-	**Total**
**WB-MRI**	+	26	11	37
	-	1	12	13
	**Total**	27	23	**50**

Table 4: Calculations broken down by tumour site

		PET & PET/CT		
	Result	+	-	**Total**
**WB-MRI**	+	brain-7<br>bone-7<br>other 12	brain-10<br>bone-1<br>other 0	37
	-	brain-0<br>bone-0<br>other-1	brain-8<br>bone-1<br>other-3	13
	**Total**	27	23	**50**

16.2. Population for analysis

The analysis population will be all patients, equivalent to the RCT population intention to treat (ITT) population, with multiple imputation used to impute missing data. For the primary outcome, complete case analysis (all available patient data with no imputation) will be reported alongside as a sensitivity analysis to the ITT analysis.

For peer review only
For peer review only - http://bmjopen.bmj.com/site/about/guidelines.xhtml

16.3. Analysis of the primary endpoint

Primary Outcome- Per patient sensitivity for metastasis detection by whole body MRI (WB-MRI) compared to standard staging pathways in newly diagnosed non-small cell lung cancer against an expert derived consensus reference standard.

The primary objective is to evaluate whether early whole body magnetic resonance Imaging (WB-MRI) increases per patient sensitivity for metastasis in non-small cell lung cancer compared to standard NICE-approved diagnostic pathways.

- Comparison to detect metastases between early WB-MRI alone and standard NICE diagnostic strategy.
- Difference in per patient sensitivity.
- Paired comparison of proportions of each strategy against the reference standard of full clinical diagnosis and 12 month follow up. [22]
- Imputation will be used to account for missing data and imperfect reference data. [23]

Definition of positive test result from standard tests (conventional imaging)

- Conventional imaging tests include CT and may include PET, USS, organ specific MRI, bone scans, lymph node sampling.
- Report from the MDT will express presence of metastatic disease for each patient categorised as yes, no and equivocal.
- Equivocal results will be grouped with positive test results as these results require additional follow up investigations compared to negative results.
- The radiology imaging report supplied to the MDT will be used for sensitivity analysis using free text report (possible and probable metastasis counted as positive).

Definition of positive test result from WB-MRI and additional tests requested after WB-MRI (WB-MRI as replacement test)

- WB-MRI imaging CRF report from the WB-MRI radiologist will express presence of metastatic disease for each patient categorised as yes, no and equivocal.
- Equivocal results will be grouped with positive test results as these results require additional follow up investigations compared to negative results.

Definition of positive test result from reference test: 12 month expert consensus panel

Expert panel results will be classified as positive or negative for metastasis by expert consensus. No equivocal category will be allowed.

Multiple imputation will be used for missing data using chained equations in STATA [24].

Sensitivity analysis: A sensitivity analysis will investigate the impact of the equivocal results on the detection rate by WB-MRI. Equivocal results will be grouped with negative test results [25].

For peer review only - http://bmjopen.bmj.com/site/about/guidelines.xhtml

16.4. Analysis of secondary endpoints

16.4.1. Efficacy (secondary)

Secondary outcome- The time and test number taken to reach, and the nature of, the first major treatment decision based on WB-MRI in comparison to standard staging pathways.

The objective is to determine how WB-MRI influences time to and nature of first major treatment decision following definitive staging compared to standard investigations and to determine whether early WB-MRI could reduce or replace standard investigations.

For this secondary outcome, the following components will be measured up to the time of the first major treatment decision

- Nature of first major treatment decision and number of patients with changed management decision
- Time taken for diagnostic pathways
- Number of tests: average per patient, number of patients with fewer tests in pathway

These will be compared for WB-MRI pathway in comparison to standard staging pathways.

This information will be reported and discussed as a basis for whether early WB-MRI could replace or reduce standard investigations.

No absolute pre-specified definition of the combination of these outcomes likely to be considered clinically significant is attempted although it is noted that:

- An average decrease in diagnosis time of 7 days is likely to be considered clinically significant if accuracy were similar.

Secondary outcome- Diagnosis accuracy of WB-MRI and conventional staging pathways for local tumour staging and detection of metastasis in comparison to an expert derived consensus reference standard.

The objective is to assess the accuracy of WB-MRI and standard diagnostic pathways for local and distant staging by comparison with an expert derived consensus reference standard using 1 year patient follow up data.

- All comparisons except difference in sensitivity per patient compare WB-MRI as both replacement and additional test compared to standard NICE staging pathway

The following outcomes will be assessed for this secondary outcome:

- Difference in sensitivity per patient: WB-MRI as additional test compared to standard NICE staging pathway
- Difference in sensitivity and specificity per organ site. Count each site once per patient, regardless of the number of metastases per organ.
- Difference in sensitivity and specificity per metastasis.

For peer review only - http://bmjopen.bmj.com/site/about/guidelines.xhtml

Secondary outcome- Inter-observer variability in WB-MRI analysis and effect of diagnostic confidence on staging accuracy.

The objective is to determine the effect of radiologist confidence on the diagnostic accuracy of WB-MRI for metastatic disease.

A substudy will be conducted to look at inter-observer variability between radiologists interpreting WB-MRI images and the effect of diagnostic confidence on staging accuracy.

- Each hub radiologist (n=6) will interpret a sample of 25 WB-MRI datasets selected at random from the other imaging hubs to define inter-observer variation in the reported presence absence of metastatic disease.
- These images will be read after patient management decisions are taken and so will not affect patient diagnosis or treatment.

Secondary outcome – Diagnostic accuracy of limited T1 and diffusion weighted sequences compared to full multi-sequence WB-MRI protocols.

To evaluate the diagnostic performance of limited WB-MRI protocols based on diffusion and T1 weighted imaging only, and to assess the incremental benefit of intravenous gadolinium contrast enhancement.

Comparison of diagnostic accuracy of WB-MRI alone (i) block 1 sequence (ii) block 2 (iii) block 3 sequences.

- Reference standard: WB-MRI plus standard tests with 12 month follow up.
- Methods and tables: as per primary outcome and secondary outcome #3.
- Analysed for units of analysis as (i) per patient (ii) per organ site (iii) per metastasis.

16.4.2. Safety

We will provide information on the following:

- Additional tests ordered as a result of WB-MRI but not ordered for the patient in the standard NICE staging pathway. Note this might include tests due to abnormalities outside lung.
- Extra days in hospital due to extra tests ordered through WB-MRI.

16.4.3. Economic evaluation

Patient-specific utility profiles will then be constructed assuming a straight line relation between each of the patients EQ-5D scores at each follow up point. The quality-adjusted life-years (QALYs) experienced by each patient up to one year will be calculated as the area underneath this profile.

We will calculate for each individual patient in the trial their costs and outcomes up to one year. We will also have data from the Lung-BOOST trial and retrospective data collection for patients previously diagnosed with lung cancer at UCLH on the cost of similar patients up to one year.

For peer review only - http://bmjopen.bmj.com/site/about/guidelines.xhtml

Individual patients will then be grouped according to the specific disease path depending on the treatment decision (e.g., surgery, radical radiotherapy or chemotherapy/non radical radiotherapy for lung cancer) and the accuracy of the staging result. We will calculate the mean costs and QALYs for each group. Mean costs and QALYs for WB-MRI versus standard staging algorithms will then be calculated based on the proportion in each group using each algorithm, which will be different since in this scenario there is discordance in treatment decision.

Cost-effectiveness will be calculated as the mean cost difference between WB-MRI versus standard staging algorithms divided by the mean difference in outcomes (QALYs) to give the incremental cost-effectiveness ratio (ICER). Non-parametric methods for calculating confidence intervals around the ICER based on bootstrapped estimates of the mean cost and QALY differences will be used [26]. The bootstrap replications will also be used to construct a cost-effectiveness acceptability curve, which will show the probability that WB-MRI is cost-effective at one year for different values of the NHS' willingness to pay for an additional QALY. We will also subject the results to extensive deterministic (one-, two- and multi-way) sensitivity analysis.

For the analysis based on the lifetime time horizon we will use the 1 year data described above. To extrapolate beyond the end of the 1 year follow up period we will develop a de novo cost-effectiveness model for the disease pathway, which will be populated via available evidence. These will model patient movements between long-term health states. The models will be similar in design to a recent NIHR HTA-funded trial of PET and MRI for detection of metastasis in breast cancer [27]. Following decisions about model structure, a list of parameter estimates required for the model will be developed. The specific details of the data to be used to populate the model will be determined following the development of the structure and the systematic searches of the literature to identify existing models.

The upshot of this analysis is that we will calculate for each individual patient in the trial their lifetime costs and QALYs. Individual patients will then be grouped according to the specific disease path and the accuracy of the staging result, as before, and we will calculate the mean costs and QALYs for each group. Mean costs and QALYs for WB-MRI versus standard staging algorithms will then be calculated based on the proportion in each group using each algorithm, again as before. We will undertake deterministic (one-, two- and multi-way) and probabilistic sensitivity analysis, the latter assuming appropriate distributions and parameter values [28].

16.4.4. Health psychology assessment

Study 1: Patient interviews (approximate n=25)

No formal statistical analysis will be carried out on the information from the patient interviews. The interviews will provide in-depth qualitative data about the experience of cancer staging and in particular undergoing WB-MRI, and will inform the modification of a questionnaire (study 2) designed to assess patient acceptability and experience of WB-MRI compared to standard tests, and also inform the design of a discrete choice experiment (study 3).

For peer review only - http://bmjopen.bmj.com/site/about/guidelines.xhtml

Study 2: Patient questionnaires (approximate n=50)

Comparative patient experience between WB-MRI and standard staging investigations, identification of important staging pathway attributes, comparative anxiety, expectations and attribute importance before and following the staging process will be analysed using within subjects ANOVA or the Wilcoxon matched-pair sign-test depending on whether the data are normally distributed or not.

Study 3: Discrete choice experiment (approximate n=50)

Sample size should be greater than (500*c)/(t*a) where t = the number of sets of choices, a = the number of scenarios to choose between in each choice, and c = the largest number of levels for any one attribute [29]. Assuming each person will undertake 15 sets of choices, there are two scenarios in each choice, and the largest number of levels for any one attribute is 3 then the required sample size is (500*3)/(15*2) = 50. We will administer this questionnaire to approximately 50 lung patients after staging has been completed, sampling across more than one of the imaging hubs. The questionnaire will be posted to patients with a pre-paid reply envelope. A reminder letter will be sent if no questionnaire has been returned within approximately 4 weeks. Analysis will conform to the checklist for conjoint analysis applications in health developed by the International Society for Pharmaceuticals and Outcome Research (ISPOR). We will use a random effects probit model for statistical analysis, which is appropriate given the data structure. Outcomes: The average relative importance weighting of attributes ascribed to standard versus WB-MRI staging pathways by patients diagnosed with lung cancer.

16.4.5. Handling missing data

Multiple imputation will be used for missing data using chained equations in STATA [24].

For the primary outcome, complete case analysis (all available patient data with no imputation) will be reported alongside as a sensitivity analysis to the ITT analysis.

16.5. Interim analyses

Interim safety data will be supplied to the IDMC on a periodic basis from UCL CTC on:

- Accumulating information relating to recruitment and data quality (e.g. data return rates).
- Safety data:
  - Additional tests performed as a result of the WB-MRI that would not have otherwise been performed as part of the patient's standard diagnostic pathway. Note this might include tests due to abnormalities outside colon or lung.
  - Potential harms to patients (e.g. staging complications resulting from MRI).
  - Whole Body MRIs that were not revealed or revealed too early/late.
- Percentage missing data for primary outcome.
- Prevalence of metastasis within recruited patients (key sample size assumption).

For peer review only - http://bmjopen.bmj.com/site/about/guidelines.xhtml

Streamline L

There will be no interim analysis based on the trial outcomes of diagnostic accuracy as all diagnostic test results are made available for the MDT meeting which decides first management treatment decision.

For peer review only

For peer review only - http://bmjopen.bmj.com/site/about/guidelines.xhtml

17. Ethical Approvals

In conducting the trial, the Sponsor, UCL CTC and sites shall comply with all laws and statutes, as amended from time to time, applicable to the performance of clinical trials including, but not limited to:

- the principles of ICH Harmonised Tripartite Guideline for Good Clinical Practice
- the Human Rights Act 1998
- the Data Protection Act 1998
- the Freedom of Information Act 2000
- the Research Governance Framework for Health and Social Care, issued by the UK Department of Health (Second Edition 2005) or the Scottish Health Department Research Governance Framework for Health and Community Care (Second Edition 2006)

17.1. Ethical Approval

The trial will be conducted in accordance with the World Medical Association Declaration of Helsinki entitled 'Ethical Principles for Medical Research Involving Human Subjects' (1996 version) and in accordance with the terms and conditions of the ethical approval given to the trial.

The trial has received a favourable opinion from the London – Camden & Islington Research Ethics Committee.

UCL CTC will submit Annual Progress Reports to the REC, which will commence one year from the date of ethical approval for the trial.

17.2. Site Approvals

The Lead Comprehensive Local Research Network (CLRN), Central and East London CLRN, has given NHS permission following global governance checks. Local governance checks will be undertaken by local CLRNs associated with individual trial sites.

Evidence of approval from the Trust R&D for a trial site must be provided to UCL CTC. Sites will only be activated when all necessary local approvals for the trial have been obtained.

17.3. Protocol Amendments

UCL CTC will be responsible for gaining ethical approval, for amendments made to the protocol and other trial-related documents. Once approved, UCL CTC will ensure that all amended documents are distributed to sites and CLRNs as appropriate.

Site staff will be responsible for acknowledging receipt of documents and for implementing all amendments.

For peer review only - http://bmjopen.bmj.com/site/about/guidelines.xhtml

17.4. Patient Confidentiality & Data Protection

Patient identifiable data, including full name, address, date of birth, gender and NHS number will be required for the registration process and will be provided to UCL CTC. UCL CTC will preserve patient confidentiality and will not disclose or reproduce any information by which patients could be identified. Data will be stored in a secure manner and UCL CTC trials are registered in accordance with the Data Protection Act 1998 with the Data Protection Officer at UCL. Patient identifiable data will be passed on to Birkbeck, University of London. This is described in the patient information sheet and the patients will consent to it.

For peer review only

For peer review only - http://bmjopen.bmj.com/site/about/guidelines.xhtml

18. Sponsorship and Indemnity

18.1. Sponsor Details

Sponsor Name: University College London

Address: Joint Research Office
Gower Street
London
WC1E 6BT

Contact: Director of Research Support

Tel 020 3447 9995/2178 (unit admin)

Fax 020 3447 9937

18.2. Indemnity

University College London holds insurance against claims from participants for injury caused by their participation in the clinical trial. Participants may be able to claim compensation if they can prove that UCL has been negligent. However, if this clinical trial is being carried out in a hospital, the hospital continues to have a duty of care to the participant of the clinical trial. University College London does not accept liability for any breach in the hospital's duty of care, or any negligence on the part of hospital employees. This applies whether the hospital is an NHS Trust or otherwise.

Hospitals selected to participate in this clinical trial shall provide clinical negligence insurance cover for harm caused by their employees and a copy of the relevant insurance policy or summary shall be provided to University College London, upon request.

For peer review only - http://bmjopen.bmj.com/site/about/guidelines.xhtml

19. Funding

This trial is funded by the National Institute for Health Research Health Technology Assessment (NIHR HTA).

For peer review only

For peer review only - http://bmjopen.bmj.com/site/about/guidelines.xhtml

20. Publication Policy

The TMG will oversee the publication and presentation of the data to peer reviewed journals and scientific meetings. All members of the TMG will approve publications. The writing committee will be led by Professor Stuart Taylor and include all TMG members. All TMG members, Trial Coordinator and Trial Statistician will be authors on the publications and named individually.

For peer review only

For peer review only - http://bmjopen.bmj.com/site/about/guidelines.xhtml

21. References

1. *Cancer Stats from Cancer Research UK Website*. Available from: http://info.cancerresearchuk.org.cancerstats/.
2. *NICE Lung Cancer Guidance 2011.*
3. Gilligan, D., et al., *Preoperative chemotherapy in patients with resectable non-small cell lung cancer: results of the MRC LU22/NVALT 2/EORTC 08012 multicentre randomised trial and update of systematic review.* Lancet, 2007. **369**(9577): p. 1929-37.
4. Floriani, I., et al., *Performance of imaging modalities in diagnosis of liver metastases from colorectal cancer: a systematic review and meta-analysis.* J Magn Reson Imaging, 2010. **31**(1): p. 19-31.
5. Qu, X., et al., *A meta-analysis of (1)(8)FDG-PET-CT, (1)(8)FDG-PET, MRI and bone scintigraphy for diagnosis of bone metastases in patients with lung cancer.* Eur J Radiol, 2012. **81**(5): p. 1007-15.
6. Ohno, Y., et al., *Non-small cell lung cancer: whole-body MR examination for M-stage assessment--utility for whole-body diffusion-weighted imaging compared with integrated FDG PET/CT.* Radiology, 2008. **248**(2): p. 643-54.
7. Pierce, D.A. and D.L. Preston, *Radiation-related cancer risks at low doses among atomic bomb survivors.* Radiat Res, 2000. **154**(2): p. 178-86.
8. Schmidt, G.P., M.F. Reiser, and A. Baur-Melnyk, *Whole-body MRI for the staging and follow-up of patients with metastasis.* Eur J Radiol, 2009. **70**(3): p. 393-400.
9. Squillaci, E., et al., *Staging of colon cancer: whole-body MRI vs. whole-body PET-CT--initial clinical experience.* Abdom Imaging, 2008. **33**(6): p. 676-88.
10. Yi, C.A., et al., *Non-small cell lung cancer staging: efficacy comparison of integrated PET/CT versus 3.0-T whole-body MR imaging.* Radiology, 2008. **248**(2): p. 632-42.
11. Risberg, T., et al., *Diagnostic delay causes more psychological distress in female than in male cancer patients.* Anticancer Res, 1996. **16**(2): p. 995-9.
12. Bastiaannet, E., et al., *Perception of burden experienced during diagnostic tests by melanoma patients with lymph node metastases.* Melanoma Res, 2009. **19**(1): p. 36-41.
13. von Wagner, C., et al., *Choosing between CT colonography and colonoscopy in the diagnostic context: a qualitative study of influences on patient preferences.* Health Expect, 2009. **12**(1): p. 18-26.
14. Harcourt, D., N. Rumsey, and N. Ambler, *Same-day diagnosis of symptomatic breast problems: Psychological impact and coping strategies.* Psychology, Health & Medicine 1999. **4**(1): p. 5.
15. *NICE Guide to the methods of technology appraisal 2008*. Available from: http://www.nice.org.uk/media/B52/A7/TAMethodsGuideUpdatedJune2008.pdf.
16. Bossuyt, P.M., et al., *Towards complete and accurate reporting of studies of diagnostic accuracy: The STARD Initiative.* Radiology, 2003. **226**(1): p. 24-8.
17. Bossuyt, P.M., et al., *Comparative accuracy: assessing new tests against existing diagnostic pathways.* BMJ, 2006. **332**(7549): p. 1089-92.
18. Ryan, M., et al., *Use of discrete choice experiments to elicit preferences.* Qual Health Care, 2001. **10 Suppl 1**: p. i55-60.

For peer review only - http://bmjopen.bmj.com/site/about/guidelines.xhtml

19. Ryan, M. and S. Farrar, *Using conjoint analysis to elicit preferences for health care.* BMJ, 2000. **320**(7248): p. 1530-3.
20. Stephens, R.J. and D.H. Johnson, *Treatment and outcomes for elderly patients with small cell lung cancer.* Drugs Aging, 2000. **17**(3): p. 229-47.
21. Julious, S.A., M.J. Campbell, and D.G. Altman, *Estimating sample sizes for continuous, binary, and ordinal outcomes in paired comparisons: practical hints.* J Biopharm Stat, 1999. **9**(2): p. 241-51.
22. Newcombe, R.G., *Improved confidence intervals for the difference between binomial proportions based on paired data.* Stat Med, 1998. **17**(22): p. 2635-50.
23. Reitsma, J.B., et al., *A review of solutions for diagnostic accuracy studies with an imperfect or missing reference standard.* J Clin Epidemiol, 2009. **62**(8): p. 797-806.
24. White, I.R., P. Royston, and A.M. Wood, *Multiple imputation using chained equations: Issues and guidance for practice.* Stat Med, 2011. **30**(4): p. 377-99.
25. Begg, C.B., R.A. Greenes, and B. Iglewicz, *The influence of uninterpretability on the assessment of diagnostic tests.* J Chronic Dis, 1986. **39**(8): p. 575-84.
26. Briggs, A.H., D.E. Wonderling, and C.Z. Mooney, *Pulling cost-effectiveness analysis up by its bootstraps: a non-parametric approach to confidence interval estimation.* Health Econ, 1997. **6**(4): p. 327-40.
27. Cooper, K.L., et al., *Positron emission tomography (PET) and magnetic resonance imaging (MRI) for the assessment of axillary lymph node metastases in early breast cancer: systematic review and economic evaluation.* Health Technol Assess, 2011. **15**(4): p. iii-iv, 1-134.
28. Briggs, A.H., K. Claxton, and M.J. Sculpher, *Decision modelling for health economic evaluation*. Oxford handbooks in health economic evaluation. 2006, Oxford: Oxford University Press. x, 237 p.
29. Johnson, R., *Conjoint Analysis Software*, I. Sawtooth Software, Editor.: Sequim, WA
30. Azvolinsky, A., *Cancer prognosis: role of BMI and fat tissue.* J Natl Cancer Inst, 2014. **106**(6): p. dju177.
31. Yip, C., et al., *Assessment of sarcopenia and changes in body composition after neoadjuvant chemotherapy and associations with clinical outcomes in oesophageal cancer.* Eur Radiol, 2014. **24**(5): p. 998-1005.
32. Attaran, S., et al., *A propensity-matched comparison of survival after lung resection in patients with a high versus low body mass index.* Eur J Cardiothorac Surg, 2012. **42**(4): p. 653-8.
33. Curvo-Semedo, L., et al., *Diffusion-weighted MRI in rectal cancer: apparent diffusion coefficient as a potential noninvasive marker of tumor aggressiveness.* J Magn Reson Imaging, 2012. **35**(6): p. 1365-71.
34. De Felice, C., et al., *Apparent diffusion coefficient on 3.0 Tesla magnetic resonance imaging and prognostic factors in breast cancer.* Eur J Gynaecol Oncol, 2014. **35**(4): p. 408-14.
35. Ohno, Y., et al., *Diffusion-weighted MRI versus 18F-FDG PET/CT: performance as predictors of tumor treatment response and patient survival in patients with non-small cell lung cancer receiving chemoradiotherapy.* AJR Am J Roentgenol, 2012. **198**(1): p. 75-82.

For peer review only - http://bmjopen.bmj.com/site/about/guidelines.xhtml

36. Zakaria, R., et al., *Diffusion-weighted MRI characteristics of the cerebral metastasis to brain boundary predicts patient outcomes.* BMC Med Imaging, 2014. **14**: p. 26.
37. Parikh, J., et al., *Changes in primary breast cancer heterogeneity may augment midtreatment MR imaging assessment of response to neoadjuvant chemotherapy.* Radiology, 2014. **272**(1): p. 100-12.

For peer review only

For peer review only - http://bmjopen.bmj.com/site/about/guidelines.xhtml

Appendix 1: Abbreviations

**CDR**	Clinical Data Repository
**CI**	Chief Investigator
**CLRN**	Comprehensive Local Research Network
**CRF**	Case Report Form
**CSP**	Coordinated System for gaining NHS Permission
**CT**	Computerised Tomography
**CTSA**	Clinical Trial Site Agreement
**DCE**	Direct Choice Experiment
**DPA**	Data Protection Act
**GCP**	Good Clinical Practice
**GHQ**	General Health Questionnaire
**ICER**	Incremental Cost-Effectiveness Ratio
**ICH GCP**	International Conference of Harmonisation-Good Clinical Practice
**IDMC**	Independent Data Monitoring Committee
**IRAS**	Integrated Research Application System
**ISF**	Investigator Site File
**ISRCTN**	International Standard Randomised Controlled Trial Number
**ITT**	Intention To Treat
**MDT**	Multi-Disciplinary Team
**MRI**	Magnetic Resonance Imaging
**NCRI**	National Cancer Research Institute
**NCRN**	National Cancer Research Network
**NHS**	National Health Service
**NICE**	National Institute for Clinical Excellence
**NIHR**	National Institute for Health Research
**NIHR HTA**	National Institute for Health Research Health Technology Assessment
**NRES**	National Research Ethics Service
**OPD**	Out Patients Department
**PACS**	Picture Archiving and Communications System
**PANAS**	Positive and Negative Affect
**PET**	Positron Emission Tomography
**PET-CT**	Positron Emission Tomography- Computerised Tomography
**PI**	Principal Investigator
**PSS**	Personal Social Services
**QA**	Quality Assurance
**QALY**	Quality Adjusted Life Year
**R&D**	Research & Development
**RCT**	Randomised Controlled Trial
**REC**	Research Ethics Committee
**RIS**	Radiology Information System
**SSI**	Site Specific Information
**STARD**	STAndards for the Reporting of Diagnostic accuracy studies
**T**	Tesla

For peer review only - http://bmjopen.bmj.com/site/about/guidelines.xhtml

**TMF**	Trial Master File
**TMG**	Trial Management Group
**TSC**	Trial Steering Committee
**UCL**	University College London
**UCL CTC**	Cancer Research UK & UCL Cancer Trials Centre
**UCLH**	University College London Hospital
**USS**	Ultrasound Scan
**VATS**	Video-Assisted Thoracoscopic Surgery
**WB-MRI**	Whole Body Magnetic Resonance Imaging
**WCBP**	Woman of Childbearing Potential

For peer review only

For peer review only - http://bmjopen.bmj.com/site/about/guidelines.xhtml

Appendix 2: Consent Process

Patients will be recruited at several hospital sites, and for many, the actual WB-MRI will take place at a different hospital (one of the central imaging hubs). It is vital that recruited patients undergo informed consent, have a minimum of 24 hours to consider participation and are subsequently free to withdraw at any time.
The consent process must be sensitive to the needs of patients which may differ from individual to individual. It is important therefore to provide patients with options as to how they may provide their consent.

The proposed consent process for this trial is based on the experience of other similar trials. It was found that patients and recruitment sites found it more efficient if the designated recruiting individual at the peripheral hospital knows that after fully explaining the trial, the patient in principle is willing to take part. For all recruitment sites, patients are seen by a clinical practitioner after a suspected diagnosis of lung cancer based on an abnormal chest X-Ray and CT scan. At this discussion the diagnosis is explained and the need for a series of staging tests (such as PET CT, bronchoscopy, biopsy etc.) is also explained. Because of the need to stage the patient quickly so treatment may start, these tests are often done within a very short time period. There is no scheduled return by the patient to an OPD during this staging process. Instead patients are next seen face to face after the MDT has made a decision about their treatment.

If consent in principle is given, the recruiter can then ring the central hub and there and then arrange the time of the MRI and thus whenever possible inform the patient immediately of the time and date so they can plan their attendance for the scan. Patients expressing an interest in participating in the trial will therefore be asked if they prefer to sign the consent form when first seen when the trial is explained (and this will be later filed in the medical notes and the conversation documented). It will be made perfectly clear that they are free to withdraw at any time and that the scan will be at least 24 hours later (usually more than this). This will save them having to re-attend the hospital simply to sign a consent form, incurring time and expense at a very busy and difficult time for them. Before they go home, where possible patients will be given the date of the WB-MRI and a copy of their signed consent form. The recruitment site can then register the patient with UCL CTC. The date of the WB-MRI will be at least 24 hours later and in reality likely several days later. The patient will take the full patient information sheet home to review at their leisure. They will be provided with a telephone number at the recruitment site to ring if on reflection, they want to opt out of the trial. If they opt out, this will be recorded in the medical notes and screening log. Patients will also be asked permission to be called by the recruitment site or imaging hub to remind them of the date of scan and confirm their attendance. Patients will retain the right to opt out at any time by cancelling or not attending their WB-MRI appointment.

As noted, a major advantage of this approach is that the patient does not have to make a special trip back to the hospital just to sign the consent form. The signed consent

For peer review only - http://bmjopen.bmj.com/site/about/guidelines.xhtml

form does not leave the recruitment hospital and can be more efficiently filed in the patient notes and a copy filed in the Investigator site file: an important aspect of trial governance and patient clinical care.

Note this method of consent has been previously deemed ethically sound and efficient in the NIHR HTA funded Siggar 1 trial (CT colonography, colonoscopy or barium enema for diagnosis or colorectal cancer in older symptomatic patients), Northern and Yorkshire MREC, MREC/3/3/075, which ran from 2004 to 2009 and recruited over 5000 patients to undergo CT colonography in a similar trial design to the current proposal. The trial team did pilot a scheme where patients returned a copy of their signed consent form after 24 hours if they still wanted to take part, but patients found this very cumbersome and confusing and much preferred the process proposed, using telephone confirmation. The procedure is supported by the patient representatives in the trial and has also worked successfully in the BOOST trial which randomised patients to standard care or endobronchial biopsy at exactly the same point in the patient pathway. The BOOST trial ran at many of the intended recruitment sites for Streamline L.

Patients will be given the option to come back and sign the consent form if they or their recruiter feel this is preferable.

Occasionally it may be more convenient for the patient to obtain written consent when the patient attends for the WB-MRI or before a planned conventional staging test. For example the patient may wish to take part in the trial, not wish to sign the consent form on the same day but also would prefer not to attend a hospital appointment just to sign the consent form. This is acceptable although it is the least desirable option. After the patient has signed consent they will need to be registered before proceeding with the WB-MRI and a copy of the consent form will need to be returned to the initial recruitment site.

For peer review only - http://bmjopen.bmj.com/site/about/guidelines.xhtml

Appendix 3: Minimum WB-MRI protocol dataset

MRI Protocol
Whole body is head to thigh

The aim is to complete the whole protocol in 60 minutes or less. Depending on the MRI technology available, it may be possible to use the 5mm slices for all axial imaging, although up to 7mm is acceptable if time constraints are problematic.

1. These specifications are a set of minimum requirements – where higher field strengths/scanner software permits the resolution/quality of sequences should be optimised (whilst keeping the maximum imaging time limited to 1 hour).

2. Scanning maybe performed at either 1.5T or 3T.

3. Whole-body coverage is defined as head to mid-thigh optimised for detection of metastases.

Imaging Procedure

a) A standard safety questionnaire should be completed.
b) For patients undergoing contrast enhancement: set up IV line in a vein in the antecubital fossa, connected to an automated injector with two syringes (contrast and saline flush).
c) Unless contra-indicated administer 20mg buscopan or 1mg glucagon iv. to be given just before the start of the scan.

1. Whole-body diffusion weighted imaging:
Axial: STIR-EPI (or other fat sat technique) diffusion weighted imaging. Fixed slice thickness of 5mm to 7mm (to match T2 and t1 weighted axials as below) two b-values (b50 and b900). A minimum acquisition matrix of 128 x 128 (or an interpolated equivalent) (rectangular FOV should be used if available and appropriate for the patient), as a reference a minimum SNR of 6 on b50 images (for liver) should be maintained if possible by increasing the number of averages. All imaging should be performed in gentle respiration (recommended as 4 stations of 50 slices beginning from the vertex to mid thighs). Diffusion imaging through the brain is optional.

2. Whole-body T2 weighted imaging:
Axial*:* Axial T2 weighted (without fat-suppression) imaging should be performed from vertex to mid-thigh. A 5 to 7 mm slice thickness should be used for all scanners. *Where possible (within the 60 minute imaging time)* respiratory and ECG triggering should be used for the chest, respiratory triggering alone for the upper abdomen. The head, neck, pelvis and legs should be scanned without any triggering. The number of stacks should be adjusted to cover the imaging volume.

For peer review only - http://bmjopen.bmj.com/site/about/guidelines.xhtml

4. **Pre-contrast T1 weighted imaging:**

DIXON Technique to be applied if available.

a. Axial: Whole-body T1 GRE (e.g. Flash 2D) non-contrast enhanced non fat sat. Image resolution and slice thickness should be ideally matched to T2 weighted imaging.

OR

b. Coronal: T1 fat saturated volume interpolated gradient echo imaging (e.g. 3D) pre contrast.

5. **Post-contrast T1 weighted imaging (if gadolinium not contraindicated or refused):**

Minimum data set
Axial liver (60-70 sec)
Axial lung (equilibrium phase)
SFOV axial head

Optional
Coronal (organ specific or whole body)

a. Axial: post contrast e.g. T1 fat saturated volume interpolated gradient echo imaging (3D) breath hold of the *liver (60-70 seconds delay) and lungs*. Multiple breath-holds employed to provide full volume coverage if required. A minimum of a 256x256 (rectangular FOV acquisition if possible and appropriate for the patient) acquisition matrix should be employed. 5-7 mm slice thickness.
b. Coronal: post contrast whole body; e.g. T1 fat saturated volume interpolated gradient echo imaging (3D) and post contrast. Slice thickness 5mm. Breath Hold.
c. **Axial:** fat saturated T1 weighted imaging of the brain (SFOV). An acquisition matrix of 256 x 256 should be employed.

For peer review only - http://bmjopen.bmj.com/site/about/guidelines.xhtml

Appendix 4: Exploratory Analysis

The collated WB-MRI datasets and 12 months patient follow up data collated as part of the trial protocol provides opportunities to retrospectively mine the data for additional diagnostic indices and prognostic markers. All analysis will be done by members of the research team under supervision of the Chief Investigator.

There is for example, increasing data linking total body fat with prognosis in malignancy [30, 31]. In lung cancer, a higher body mass index has been associated with a better prognosis [32]. From the WB-MRI datasets acquired as part of the trial, it is possible to extract quantitative data on total body fat using simple segmentation algorithms which can be linked with patients' outcomes such as metastatic status and 12 month survival.

In addition, quantification of the MRI signal returned from sites of disease may be able to predict histological characteristics and also may be linked to prognosis. For example diffusion weighted imaging of the primary tumour has been shown to differentiate between grades of dysplasia in rectal cancer [33] and may predict prognosis in lung, brain and breast cancer [34-36]. The heterogeneity of signal within tumours can be measured and has been linked with overall prognosis and treatment response. For example in breast cancer, increased homogeneity of the signal measured using regions of interest placed in metastatic deposits in response to treatment is associated with a better prognosis [37]. The various sequences performed as part of the collected WB-MRI protocol will allow testing of such associations in recruited patients.

The following or similar may be quantified in all or a subset of WB-MRI datasets:

1) Body fat (organ, visceral and whole body).
2) MRI signal from the primary tumour and metastatic sites, notably diffusion weighted signal, apparent diffusion coefficient, T1 (including fat and water components), T2 signal, Contrast enhancement, and textural analysis (for heterogeneity) of the primary and metastatic deposits on the various sequences.

We will look for correlations between MRI variables and clinical data collated as part of the trial (for example basic histology, metastatic status, patient outcome and findings of conventional staging tests) or collected as part of the patients' usual care, for example, more detailed histology of biopsies or excised tumour (including tumours markers if collected as part of usual clinical care). Associations will be sought between the MRI variables and the clinical variables using appropriate conventional parametric and non-parametric statistics, for example:

- Differences in body fat composition between different cancer histological subtypes, patients with and without metastatic disease, and according to 12 month survival.
- Heterogeneity of MRI signal from a region of interest drawn within the primary tumours and metastatic deposits, comparing between lesions in the same

For peer review only - http://bmjopen.bmj.com/site/about/guidelines.xhtml

patient, between different patients and according to histological parameters and 12 month survival.

In addition comparisons will be made between MRI signal within normal body tissues (such as the liver, pancreas and muscle) between patients scanned on the different MRI scanners comparing with local MRI phantom data as appropriate to see how homogenous the MRI data is between different imaging hubs.

In some instances use of external commercial software may be required (e.g. fat quantification). MRI datasets will be anonymised and no personal identifiable information will be shared outside the research team at any time.

For peer review only

For peer review only - http://bmjopen.bmj.com/site/about/guidelines.xhtml

Appendix 5: Protocol Version History

**Protocol:**		**Amendments:**		
**Version no.**	**Date**	**Amendment no.**	**Protocol Section (no./title)**	**Summary of main changes from previous version.**
1.0				
1.1	14/09/12	N/A	Front cover & Section 1.1<br>Section 5<br>8.2.10<br>Section 14.2<br>Appendix 2	ISRCTN number added and new CR UK logo added<br>Flowchart and inserted re consent process<br>Text inserted re QoLs<br>Text inserted re destruction of interview recordings<br>Text inserted re consent process
2.0	15/03/13	1.0	Throughout<br>Section 4<br>Section 5<br>Section 7<br>Section 8<br>Section 9<br>Section 12<br>Section 13<br>Appendix 3	Typos and clarifications<br>Clarification about GCP training and documents required for activation updated<br>Details added about interpreters for informed consent and removal of informed consent log information<br>Removal of text regarding slot availability updates<br>Addition of ad hoc mini MDT, reduce the amount of patient experience questionnaires, change the content of these questionnaires and alter who is sending them to patients. Clarifications of timing of WB-MRI and the process of early release of findings<br>Update data management guidelines in line with changes at UCL CTC<br>Details removed about informed consent log<br>Addition of details re patient withdrawal<br>Clarifications to Whole body MRI protocol
3.0	24/04/14	3.0	Section 1<br>Section 5<br>Section 6<br>Section 7<br>Section 8	Rewording of the inclusion criteria<br>Removal of information about QoL and Patient diaries given out by site at consent<br>Rewording of the MDT log information and inclusion criteria<br>Removal of patient diaries and QoLs from registration packs.<br>Rewording and clarification of Conventional Imaging, WB-MRI reveal and MDT discussion, Final Treatment decision and follow up sections. Updating information to state that the Interview section of the trial is now closed to recruitment. Updating information that UCL CTC is now posting out QoL and patient diaries at baseline.

For peer review only - http://bmjopen.bmj.com/site/about/guidelines.xhtml

4.0	22/12/14	4.0	Section 8.2.12<br><br>Section 10<br><br>Appendix 4<br><br>Appendix 5	Information added regarding exploratory analysis for additional diagnostic indices and prognostic markers.<br>Safety Reporting section updated to provide more information on staging complications and how these are reported and reviewed by the CI.<br>Exploratory Analysis added to include the additional analysis of WB-MRI scans<br>Protocol Version History has become Appendix 5.
5.0	11/05/15	5.0	Section 8<br><br>Section 10<br><br>Section 13<br><br>Section 16	Changing target recruitment for patient experience questionnaires to 50 patients and closing this part of the trial. Updating and clarifying information regarding the Discrete Choice Experiment and confirming the time point these will be sent to patients.<br>Updating the Safety Section to confirm that only staging complications related to the WB-MRI or additional tests performed due to WB-MRI are to be recorded on the Staging Complications CRF.<br>The withdrawal process was updated and clarified to better match the new Change of Status CRF.<br>The type of Interim safety data that will be supplied to IDMC was updated
6.0	06/09/16	6.0	Sections 1.1 & 1.2<br><br>Section 13<br><br>Section 16.1	Recruitment target increased from 250 to 353<br>Anticipated duration of recruitment increased from 3 to 4 years.<br>Deletion of section on early release of WB MRI and patient failure to undergo WB MRI<br>Rationale for revised target accrual added<br>Grammatical and typographical errors corrected throughout

For peer review only

For peer review only - http://bmjopen.bmj.com/site/about/guidelines.xhtml

COREQ (COnsolidated criteria for REporting Qualitative research) Checklist

A checklist of items that should be included in reports of qualitative research. You must report the page number in your manuscript where you consider each of the items listed in this checklist. If you have not included this information, either revise your manuscript accordingly before submitting or note N/A.

Topic	Item No.	Guide Questions/Description	Reported on Page No.
**Domain 1: Research team and reflexivity**			
Personal characteristics			
Interviewer/facilitator	1	Which author/s conducted the interview or focus group?	Page 8
Credentials	2	What were the researcher's credentials? E.g. PhD, MD	Page 8
Occupation	3	What was their occupation at the time of the study?	Page 8
Gender	4	Was the researcher male or female?	Page 8
Experience and training	5	What experience or training did the researcher have?	Page 8
Relationship with participants			
Relationship established	6	Was a relationship established prior to study commencement?	Page 7
Participant knowledge of the interviewer	7	What did the participants know about the researcher? e.g. personal goals, reasons for doing the research	Page 8
Interviewer characteristics	8	What characteristics were reported about the inter viewer/facilitator? e.g. Bias, assumptions, reasons and interests in the research topic	Page 8
**Domain 2: Study design**			
Theoretical framework			
Methodological orientation and Theory	9	What methodological orientation was stated to underpin the study? e.g. grounded theory, discourse analysis, ethnography, phenomenology, content analysis	Page 9
Participant selection			
Sampling	10	How were participants selected? e.g. purposive, convenience, consecutive, snowball	Page 9
Method of approach	11	How were participants approached? e.g. face-to-face, telephone, mail, email	Page 7
Sample size	12	How many participants were in the study?	Page 8
Non-participation	13	How many people refused to participate or dropped out? Reasons?	Page 8
Setting			
Setting of data collection	14	Where was the data collected? e.g. home, clinic, workplace	Page 7
Presence of non-participants	15	Was anyone else present besides the participants and researchers?	Page 7
Description of sample	16	What are the important characteristics of the sample? e.g. demographic data, date	Pages 10& 11
Data collection			
Interview guide	17	Were questions, prompts, guides provided by the authors? Was it pilot tested?	Box 1 and Page 8
Repeat interviews	18	Were repeat inter views carried out? If yes, how many?	Page 7
Audio/visual recording	19	Did the research use audio or visual recording to collect the data?	Page 8
Field notes	20	Were field notes made during and/or after the inter view or focus group?	Page 9
Duration	21	What was the duration of the inter views or focus group?	Page 8
Data saturation	22	Was data saturation discussed?	Page 8
Transcripts returned	23	Were transcripts returned to participants for comment and/or	Page 9

For peer review only

Topic	Item No.	Guide Questions/Description	Reported on Page No.
		correction?	
**Domain 3: analysis and findings** *Data analysis*			
Number of data coders	24	How many data coders coded the data?	Page 9
Description of the coding tree	25	Did authors provide a description of the coding tree?	Page 9
Derivation of themes	26	Were themes identified in advance or derived from the data?	Page 9
Software	27	What software, if applicable, was used to manage the data?	Page 9
Participant checking	28	Did participants provide feedback on the findings?	Page 9
Reporting			
Quotations presented	29	Were participant quotations presented to illustrate the themes/findings? Was each quotation identified? e.g. participant number	Pages 11-18
Data and findings consistent	30	Was there consistency between the data presented and the findings?	Pages 11-18
Clarity of major themes	31	Were major themes clearly presented in the findings?	Pages 11-18
Clarity of minor themes	32	Is there a description of diverse cases or discussion of minor themes?	Pages 11-18

Developed from: Tong A, Sainsbury P, Craig J. Consolidated criteria for reporting qualitative research (COREQ): a 32-item checklist for interviews and focus groups. *International Journal for Quality in Health Care*. 2007. Volume 19, Number 6: pp. 349 – 357

Once you have completed this checklist, please save a copy and upload it as part of your submission. DO NOT include this checklist as part of the main manuscript document. It must be uploaded as a separate file.

For peer review only

Patient experience and perceived acceptability of whole body magnetic resonance imaging for staging colorectal and lung cancer compared with current staging scans: a qualitative study

Journal:	*BMJ Open*
Manuscript ID	bmjopen-2017-016391.R1
Article Type:	Research
Date Submitted by the Author:	02-May-2017
Complete List of Authors:	Evans, Ruth; Birkbeck University of London, Psychological Sciences<br>Taylor, Stuart; University College London Hospitals NHS Foundation Trust, Centre for Medical Imaging<br>Janes, Sam; University College London, Respiratory Medicine<br>Halligan, Steve; University College London, Centre for Medical Imaging<br>Morton, Alison; Patient Representative<br>Navani, Neal; University College London, Respiratory medicine<br>Oliver, Alf; Patient Representative<br>Rockall, Andrea; Royal Marsden NHS Foundation Trust, Radiology<br>Teague, Jonathan; Cancer Research UK and UCL Clinical Trials Centre<br>Miles, Anne; Birkbeck, University of London,
**Primary Subject Heading**:	Radiology and imaging
Secondary Subject Heading:	Oncology, Patient-centred medicine, Qualitative research
Keywords:	Magnetic resonance imaging < RADIOLOGY & IMAGING, Respiratory tract tumours < ONCOLOGY, Gastrointestinal tumours < ONCOLOGY, QUALITATIVE RESEARCH

Title: Patient experience and perceived acceptability of whole body magnetic resonance imaging for staging colorectal and lung cancer compared with current staging scans: a qualitative study.

Authors: REC Evans[a], SA Taylor[b], S Janes[c], S Halligan[b], A Morton[d], N Navani[c], A Oliver[d], A Rockall[e], J Teague[f] and A Miles[a]

[a] Psychological Sciences, Birkbeck, University of London, Malet Street, London, WC1E 7HX

[b] Centre for Medical Imaging, Division of Medicine, University College London, Gower Street, London, WC1E 6BT

[c] Lungs for Living Research Centre, Division of Medicine, University College London, Gower Street, London, WC1E 6BT

[d] Patient Representative, no institutional address available

[e] Department of Surgery and Cancer, Imperial College London, Kensington, London SW7 2AZ

[f] CR UK &UCL Clinical Trials Centre, 90 Tottenham Court Road, London, W1T 4TJ

Corresponding author: Dr Anne Miles, Psychological Science, Birkbeck, University of London, Malet Street, London, WC1E 7HX, ae.miles@bbk.ac.uk, Tel: +44 20 7631 6488

Funding statement:

This project was funded by the National Institute of Health Research health technology assessment NIHR HTA programme (project number 10/68/01) and will be published in full in Health Technology Assessment. The project is supported by researchers at the National

For peer review only - http://bmjopen.bmj.com/site/about/guidelines.xhtml

For peer review only

Institute for Health Research University College London Hospitals Biomedical Research Centre.

Department of Health disclaimer:

This report presents independent research commissioned by the National Institute for Health Research (NIHR). The views and opinions expressed by authors in this publication are those of the authors and do not necessarily reflect those of the NHS, the NIHR, NETSCC or the HTA programme or the Department of Health. The views and opinions expressed by the interviewees in this publication are those of the interviewees and do not necessarily reflect those of the authors, those of the NHS, the NIHR, MRC, CCF, NETSCC or the HTA programme or the Department of Health.

Competing interests

We have read and understood BMJ policy on declaration of interests and declare that we have no competing interests.

Authors' contribution

SA Taylor, S Janes, S Halligan, A Morton, N Navani, A Oliver, A Rockall and A Miles designed the clinical trial protocol which includes this study. J Teague assisted in patient recruitment. REC Evans and A Miles interviewed patients, analysed and interpreted the data. REC Evans wrote the first draft of the manuscript. All authors reviewed the manuscript and approved the final draft.

Acknowledgments:

The authors would like to acknowledge the contribution of the following individuals to the clinical trial which provided the data presented in this paper: A Aboagye, L Agoramoorthy,

For peer review only - http://bmjopen.bmj.com/site/about/guidelines.xhtml

S Ahmed, A Amadi, G Anand, G Atkin, A Austria, S Ball, F Bazari, R Beable, S Beare, H Beedham, T Beeston, N Bharwani, G Bhatnagar, A Bhowmik, L Blakeway, D Blunt, P Boavida, D Boisfer, D Breen, J Bridgewater, S Burke, R Butawan, Y Campbell, E Chang, D Chao, S Chukundah, B Collins, C Collins, V Conteh, J Couture, J Crosbie, H Curtis, A Daniel, L Davis, K Desai, M Duggan, S Ellis, C Elton, A Engledow, C Everitt, S Ferdous, A Frow, M Furneaux, N Gibbons, R Glynne-Jones, A Gogbashian, V Goh, S Gourtsoyianni, A Green, Laura Green, Liz Green, A Groves, A Guthrie, E Hadley, A Hameeduddin, G Hanid, S Hans, B Hans, A Higginson, L Honeyfield, H Hughes, J Hughes, L Hurl, E Isaac, M Jackson, A Jalloh, R Jannapureddy, A Jayme, A Johnson, E Johnson, P Julka, J Kalasthry, E Karapanagiotou, S Karp, C Kay, J Kellaway, S Khan, D Koh, T Light, P Limbu, S Lock, I Locke, T Loke, A Lowe, N Lucas, S Maheswaran, S Mallett, E Marwood, J McGowan, F Mckirdy, T Mills-Baldock, T Moon, V Morgan, S Morris, S Nasseri, P Nichols, C Norman, E Ntala, A Nunes, A Obichere, J O'Donohue, I Olaleye, A Onajobi, T O'Shaughnessy, A Padhani, H Pardoe, W Partridge, U Patel, K Perry, W Piga, D Prezzi, K Prior, S Punwani, J Pyers, H Rafiee, F Rahman, I Rajanpandian, S Ramesh, S Raouf, K Reczko, A Reinhardt, D Robinson, P Russell, K Sargus, E Scurr, K Shahabuddin, A Sharp, B Shepherd, H Sidhu, I Simcock, C Simeon, A Smith, D Smith, D Snell, J Spence, R Srirajaskanthan, V Stachini, S Stegner, J Stirling, N Strickland, K Tarver, M Thaha, M Train, S Tulmuntaha, N Tunariu, K van Ree, A Verjee, C Wanstall, S Weir, S Wijeyekoon, J Wilson, S Wilson, T Win, L Woodrow, D Yu.

Data sharing statement: Extra data from anonymised transcripts are available by emailing ae.miles@bbk.ac.uk

For peer review only

ABSTRACT:

Objective: To describe the experience and acceptability of whole body magnetic resonance imaging (WB-MRI) staging compared with standard scans among patients with highly suspected or known colorectal or lung cancer.

Design: Qualitative study using one-to-one interviews with thematic analysis.

Setting: Patients recruited from 10 hospitals in London, East and South East England between March 2013 and July 2014.

Participants:51 patients (31 male, age range 40 to 89 years), with varying levels of social deprivation were recruited consecutively from two parallel clinical trials comparing the diagnostic accuracy and cost-effectiveness of WB-MRI with standard scans for staging colorectal and lung cancer (‘XX- C’ and ‘XX-L’).

Results: In general WB-MRI presented a greater challenge than standard scans, although all but 4 patients completed the WB-MRI. Key challenges were: enclosed space, noise and scan duration; reduced patient tolerance was associated with claustrophobia, pulmonary symptoms and existing co-morbidities. Coping strategies facilitated scan tolerance and were grouped into 1) those intended to help with physical and emotional challenges, and 2) those focused on motivation to complete the scan e.g. focusing on health benefit. Positive staff interaction bolstered patients’ ability to cope with WB-MRI, while negative staff interactions had the opposite effect.

Conclusions: Although WB-MRI was perceived as more challenging than standard scans, it was sufficiently acceptable and tolerated by most patients to potentially replace them if appropriate.

Trial Registration: ISRCTN43958015 and ISRCTN50436483

Keywords: Cancer Oncology Neoplasms Magnetic Resonance Imaging Patient Satisfaction

For peer review only - http://bmjopen.bmj.com/site/about/guidelines.xhtml

ARTICLE SUMMARY

Strengths and limitations of this study

- This study is the first to describe the experience and acceptability of having a whole body MRI in patients with highly suspected lung or colorectal cancer compared with standard scans.
- Qualitative methodology, and large numbers recruited (with wide age range and deprivation levels), enabled us to observe that there is a range of intensity of difficulties experienced during the whole body MRI.
- Assessment within the context of a research trial, with some patient declining participation due to claustrophobia, might have led to an under-reporting of WB-MRI challenge.

For peer review only

For peer review only - http://bmjopen.bmj.com/site/about/guidelines.xhtml

BACKGROUND

Current NICE recommended staging pathways for lung and colorectal cancer are often complex and time consuming, involving several different imaging tests utlilising ionising radiation, and potentially adding to the physical and psychological burden of patients with suspected cancer (1,2). For example it is not unusual for a patient with suspected lung cancer to undergo diagnostic CT, followed by staging PET-CT, dedicated brain imaging and invasive mediastinal nodal sampling prior to the first major treatment decision. More accurate and streamlined staging could improve patient outcomes both by triaging to optimal therapy and decreasing the time between diagnosis and treatment (3). Whole body magnetic resonance imaging (WB-MRI) has been advocated as a safe accurate and efficient “one stop shop” investigation that could potentially replace current complex multi-modality staging strategies (4). A single WB-MRI scan could not only accelerate staging but would simultaneously reduce exposure to ionising radiation, in theory reducing the risk of subsequent radiation induced malignancies, particularly in those diagnosed at a younger age. WB-MRI however can be stressful: its duration, 45 to 90 minutes, is longer than standard tests - CT takes a few seconds. MRI scanners are noisy and require full body and head immersion into a relatively narrow tube, often necessitating coils wrapped around the patient that restrict movement. Somewhere between 5-30% of patients experience distress associated with the anticipated and actual experience of undergoing MRI (5-9). Anxiety relates to the scan experience itself as well as the result (5). Severe claustrophobia can lead to premature scan termination in 1-15% of attempts (10,11), and distress is associated with elevated post scan anxiety (6), which can engender MRI fear or phobia (12-14), especially problematic in patients needing future MRI studies.

Increased physical and psychological vulnerability of patients with suspected cancer may reduce their ability to cope with a WB-MRI (2,15). Cancer diagnosis and subsequent

For peer review only - http://bmjopen.bmj.com/site/about/guidelines.xhtml

treatment are still much feared despite recognition of better treatment outcomes (16,17); and patients may be experiencing shock, anxiety and worries about the future (15,18). While two small qualitative studies have investigated general patient experience of MRI (19,20) there have been few descriptions of patients undergoing WB- MRI, particularly in those diagnosed with or highly suspected of having cancer.

Patient acceptability is central to the successful adoption of any new technology. Poor acceptability could reduce adherence to WB-MRI, which in turn considerably blunts the impact of the technology, even if diagnostically superior to existing tests.

Two parallel, multi-centre, prospective cohort studies (ref removed) investigating the diagnostic accuracy and cost-effectiveness of WB-MRI compared to standard pathways for newly diagnosed lung ('XX-L') and colon ('XX-C') cancer have recently completed. The aim of this study was to describe patient experience and acceptability of WB-MRI and compare with standard staging tests, in the context of lung and colorectal cancer, using interviews from patients recruited to the XX trials.

METHODS

There is little existing research describing patients' experiences of undergoing WB-MRI and we adopted a qualitative design as this provides the flexibility to capture rich descriptions of experiences without a priori knowledge of potential responses. One-to-one interviews were completed face to face (at home or at hospital) or via telephone. Individual interviews were conducted to facilitate expression of emotions and negative experiences that may be inhibited in a group setting. For two interviews a relative was present, to support translation where English was not the patient's first language. Patients were interviewed only once.

For peer review only - http://bmjopen.bmj.com/site/about/guidelines.xhtml

Trial and interview study recruitment

Patients were recruited to the 'XX' trials from 10 English NHS hospitals and consented to undergo WB-MRI in addition to standard staging investigations at one of five MRI scan hospital hubs. During trial consent, patients could opt into the interview study and were contacted, by psychology researchers by phone, as soon as possible after all imaging had been completed. Accelerated treatment pathways meant some interviews (n=13) were undertaken after treatment had commenced. Trial inclusion/ exclusion criteria are listed in the study protocol (reference removed).

The a priori total sample size was a minimum of 50, stratified by cancer site (25 lung and 25 colorectal patients). There was flexibility to recruit beyond this number to achieve saturation, i.e. the point beyond which further interviews contribute little new information, although this was not needed. The first 123 trial patients were approached and 91 (74%) agreed to be interviewed. Of the 91 who agreed, 51 patients (56%) participated; we believed we had reached saturation by 50 patients and ceased interviews, however one additional interview was subsequently completed as a patient (from the original 91 consenting patients) expressed a strong wish to share their views.. Reasons patients were not interviewed included: consent retracted (n=8); participation in a related questionnaire study (n=14); withdrawal from the main trial (n=12); and interview quota reached before completion of all staging imaging tests (n=6).

Conducting the interview

Interviews were conducted from March 2013 to July 2014, by two female psychology researchers (PhD qualified), independent of the clinical trial team, with prior training and experience of conducting in-depth interviews with cancer patients (refs removed). The researchers introduced themselves as working with the doctors at the patient's hospital and

For peer review only - http://bmjopen.bmj.com/site/about/guidelines.xhtml

explained that the interview was an opportunity to provide feedback about the WB-MRI and other tests the patients had had. A topic guide was developed (Box 1) to encourage discussion of key aspects of the staging experience including comparisons between WB-MRI and standard investigations. This guide was reviewed after completion of the first two interviews to assess whether it needed to be modified. Topic ordering and emphasis varied depending on relevance to each patient. Open ended questions were followed by verbal probing to elicit further clarification. Interviews lasted between 12 and 86 minutes (mean 48 minutes), and were recorded digitally. Participating patients were paid £20 plus travel expenses.

Box 1: Key interview topics

- *I understand that you have been having a number of scans to investigate your symptoms, can you tell me what's been happening?*
- *Can you describe what it feels like physically to have the scan?*
- *How did you feel during/after/about the test?*
- *What were the staff like?*
- *What did you do to cope with [problem identified by patient e.g. noise, keeping still, etc]*
- *Would you have another WB-MRI if the doctor recommended it?*

Additional measures

Age, gender and postcode data were collated. Postcode was used to calculate an area based deprivation score using the 2010 IMD scale (21) which was then categorised into quintiles (quintile 1 highest and quintile 5 lowest deprivation).

Data capture, coding and analysis

For peer review only - http://bmjopen.bmj.com/site/about/guidelines.xhtml

Interviews were transcribed verbatim. NVIVO, version 10, was used to manage data (tagging and labelling) while completing thematic analysis (22). A coding structure was developed by (psychology researcher name removed), using transcript review combined with a reflective log maintained during interviews, this was reviewed and agreed by both psychology researchers. This framework identified themes related to scan events, beliefs, attitudes, and emotional responses as well as coping strategies. Themes identified were influenced by prior research as well as emergent from the data. This process was iterative, with constant data comparisons to identify similarities and differences within and across individual interviews. Matrix tables were created with themes as columns and relevant data summarised into separate rows from each transcript. Matrix tables provided an overview of all 51 interviews, and ensured that the full range of experiences were represented in the final description. Patients were not asked to verify the thematic analysis so as not to increase participation burden.

Statistical analysis

Data were entered into SPSS, version 20, and Analysis Of Variance and Chi Square used to test continuous and categorical data respectively.

Ethical review

The trial protocol was reviewed and ethical permission granted by Camden and Islington NRES committee on 03/10/2012 (project number removed).

RESULTS

Table 1 gives full demographics of the interviewed cohort. All but four patients completed the WB-MRI. There were no significant differences in age, gender, or area-based deprivation between those who volunteered for interviews (n=91), and those who declined (n=32); mean

For peer review only - http://bmjopen.bmj.com/site/about/guidelines.xhtml

age: 65 vs. 64, $F_{(1,121)}$ <1, p=0.527; % male: 64% vs. 56%, X^2 (df=1, n=123) =0.562, p=0.453; % in deprivation categories (Fisher's Exact Test chi-square: 1.834, p=0.788). Similarly there were no significant demographic differences between patients who were (n=51) or were not (n=40) interviewed; mean age 65 vs. 64, $F_{(1,89)}$ <1, p=0.579; gender % male: 53.4% vs. 46.6%, X^2 (df=1, n=91)=0.437, p=0.508, % in deprivation categories (Fisher's Exact chi-square: 5.247, p=0.254). The majority of patients were interviewed over the phone (76%, n=39) and the median interval between WB-MRI and interview was 15 days (1-63 days).

Table 1: Demographic characteristics of interview participants, by trial stream

	'XX- L'* % (n=25)	'XX-C'* %(n=26)	Group differences
Gender			
Male	60 (15)	61.5 (16)	X^2=0.013, df=1,
Female	40 (10)	38.5 (10)	p=0.910
Mean age (years)	65 (Sd=11 years)	64 (sd=9 years)	t=0.261, df=49; p=0.795
Area Deprivation Score (quintile)			
Highest 1	52.0 (13)	30.8 (8)	X^2=5.459
2	32.0 (8)	23.1 (6)	df=2,
Mid to low 3-5	16.0 (4)	46.2 (12)	p=0.065

For peer review only - http://bmjopen.bmj.com/site/about/guidelines.xhtml

I/v method			
Face to Face	20.0 (5)	26.9 (7)	X^2=0.339, df=1;
Phone	80.0 (20)	73.1 (19)	p=0.560
Median Interval between interview and WB-MRI	15 days (6-43 days)	20 days (1-63 days)	U=270.00, n=51, p=0.299

*XX-L' -lung cancer. 'XX-C' –colorectal cancer

Was the WB-MRI scan a challenge? Diversity of experience and comparison with other scans and tests.

As part of the XX trials, all patients had WB-MRI as an additional 'trial' investigation alongside standard staging investigations. For patients recruited to 'XX-C', standard investigation included CT of the chest, abdomen and pelvis, with some undergoing an additional abdominal/ pelvic MRI. Patients recruited to 'XX-L' underwent at least a CT and PET-CT with a proportion undergoing bronchoscopy for tissue sampling.

WB-MRI was perceived to be more challenging than standard scans by many: *"I would say the most challenging of the MRI scans was the body scan"*s56 (60-69, male, Colorectal -'C'), like *"torture - medieval torture"*s12 (50-59,male,C) while CT was described as *"a walk in the park"*s42 (60-69, male, C), and *"very simple"*s35 (50-59, female, Lung – 'L'). PET-CT was described by some as *"so much easier"*s51 (70-79, female, L) and *"wasn't so unpleasant"* s12 (50-59, male, C). However the CT and PET_CT were not without challenges: for example some commented on the intravenous contrast administered during the CT scan*"you feel a bit, you know, woozy, funny, …and it did make me feel slightly off"*

For peer review only - http://bmjopen.bmj.com/site/about/guidelines.xhtml

s63 (<50, male, C) and the perceived 'invasiveness' of the radiation exposure associated with CT and PET-CT: "*all that radiation dye in my system and everything…And it just disturbed me. Because I couldn't go near my niece and she's only six"* s54 (<50, female, L). Another patient described the bronchoscopy as more unpleasant *"I'd rather have them scans again than have that tube down my throat again, that was the worst thing, and I hated it"* s73 (60-69, male, L).

For peer review only

What was difficult about the WB-MRI?

Factors identified as difficult were: claustrophobia, physical discomfort, noise, scan duration, as well as the challenge of coping with emotions elicited during the scan such as fear /panic and isolation. There was a range of reaction intensity, with some reporting positive experiences (Table 2).

Table 2: Problems and the diversity of challenge experienced related to WB-MRI

Problem	No challenge/ enjoyable	Low challenge	Medium/ High	Did not complete
Enclosed space	*"I'm not claustrophobic …that [enclosed space] doesn't frighten me" s36 (50-59, male,L)*	*"Maybe in the back of my mind you're worried about it [enclosed space]" s5(60-69, female, C)*	*"Coffin came to mind…initially, you feel like 'Oh god, it's so close in here'" s26(60-69, female,L)*	**"***I had this sense of being in a sarcophagus" s17(60-69, male, L)*

Noise	*"I thought it like music really…and it relaxed me"* s64(80+, male,C)	*"the noise was a bit…disconcerting because it was quite loud and quite sort of clanking and banging… …and it took some time getting used to it"* s63(<50, male,C)	*"that's one of the worst ones that I've had to go through with the noise… I felt like something was going to fall off and hit me."* s54(<50, female,L)	
Scan duration/ discomfort	*"It's actually quite comfortable in there"* s87(60-69, male,L)	*"It was not excruciatingly painful. It just ached a bit, that is all."* s18(60-69, male,C)	*"My back was uncomfortable. My, the back of my neck was uncomfortable…" "my brain was saying, "You can't go much longer…* s56(60-69, male,C)	
Negative Emotions (e.g. anxiety, panic, shock, distress)	*"I mean, there wasn't, you know, anything in particular to*	*"when I first went in there, I was quite shocked …. …I wondered*	*"when I first laid down in the scan I was frightened" s79(60-69,*	*I don't know whether the people doing the scan knew I*

For peer review only - http://bmjopen.bmj.com/site/about/guidelines.xhtml

	be frightening [sic]" s76(<50, male,C)	*what the hell was going on… after a while I quite enjoyed it"* s18(60-69, male,C)	*female,C)* and *"I'm lying there and all of a sudden, they put all these things on top of me… And I'm thinking, 'Oh, my God! …this is threatening'"*s53 (60-69, male,C)	*had lung cancer, I didn't feel any empathy or any sort of friendly attitude. I walked out of there feeling quite upset actually"* s30 (50-59,male,L)

For peer review only

Claustrophobia: patients described feeling trapped or buried in the WB-MRI "*it was the feeling of being sort of trapped because you can feel the machine all around your body"* s12 (50-59, male, C) and "*I had this sense of being in this sarcophagus"* s17 (60-69, male, L). Only one participant asked to terminate the scan early because of claustrophobia (s17). In comparison the CT scan was described as being like "*going through the big polo mint. And, therefore, it's not as much as like entombment"* s80 (60-69, male, C) and the PET-CT was described as *"not as claustrophobic. That one [MRI] is close to your face. And the other one [PET-CT] is a bit higher. So that, you know, that's not a big problem, really"* s73 (60-69, male, L).

Noise: Noise emmited from the MRI during data acquisition was aversive for some "*horrendous. It's horrible! "*s55 (60-69, female, L), but not all, *"it was quite good.… I would say, 'What's coming next? What's the next noise?' So, I enjoyed that"* s18 (60-69,

male, C). The absence of noise was a notable feature of other scans e.g. "*PET scan isn't noisy*" s91 (60-69, male, L) and "*there wasn't all the noise. That's the only difference really*" s41 (60-69, male, L).

Scan duration and physical discomfort**:** The scan length differentiated WB-MRI from other tests "*the CT scan is very quick…you're in and out. Whereas, the MRI scan was very long"* s13 (60-69, female, C). However one patient saw the PET-CT scan duration times as equivalent "*if you took the PET scan, and take the waiting time [for radioactive glucose to circulate], and deduct that, timewise, they're much the same"* s49 (>80, male, L). Duration was linked to comfort: "*the back of my neck was uncomfortable, [the WB-MRI] maybe 15 minutes too long to be comfortable"* s56 (60-69, male, C) In contrast a patient without discomfort viewed the long duration acceptable, *"I think it's over one and a half hour or something like that, so it was alright for me… is comfortable"* s19 (60-69, male, C). Emotions elicited by WB-MRI: WB-MRI induced strong emotions in some:*"I was going to have a sort of panic attack and have to be taken out of the machine…this claustrophobia, the feeling of being...trapped, really" s12*(50-59, male, C). Worry and fear were also prompted by the novelty of the experience "*the oppression comes from the unknown"*s45 (70-79, male, C). Dealing with feelings of panic and fear was a challenge in itself and needed effort to overcome "*when I first laid down in the scan I was frightened…I was trying to put it [fear] out of my head and think positively*" s79 (60-69, female, C). In comparison "*they [CT and PET-CT] were slightly more easier to relax in that environment, not that I didn't relax in the MRI scan too, it's just that it took a bit more sort of power, more energy if you like, to relax*" s41, (60-69, male, L).

Cancer context: The WB-MRI was perceived to have the potential to either suggest a cancer diagnosis or reveal additional findings associated with a poorer prognosis and treatment

For peer review only - http://bmjopen.bmj.com/site/about/guidelines.xhtml

implications *"I was lying in there and I was thinking, what if they say it's cancer? Then, what?"* s54 (<50, female, L) and *"whether they'd find … it has gone everywhere and you know nothing you can do, I suppose that was the main worry"* s60 (60-69, female, L). One patient had a dilemma whether to go ahead with the scan because of the possibility that its findings might mean he wasn't offered treatment "*I was of two minds about the trial scan because I didn't want the trial scan to say the cancer's spread and they wouldn't operate."* s30 (50-59, male, L). However another patient reported relief when the scan confirmed that initial tumours were localised "*my biggest fear, I know I've got the cancer in the bowels. I've seen it myself on the colonoscopy. And I was worried that they might, it might just disguise I've got other cancers. So when he said straight away, there was nothing else and I feel relieved*" s45 (70-79, male, C). There was also appreciation of having the WB-MRI because it did reveal spread "*The MRI actually showed up even more of what's going on with me and I think if I hadn't had it, they would…we still wouldn't know. Because they found traces in five other places I feel a lot better now having the MRI scan, knowing that they've picked up on all of it" and "without that, they would…I would just be having the chemo and not the full treatment that I need*" s25 (<50, female, L). The existential threat posed by a cancer diagnosis may have influenced how physical characteristics of the scan where interpreted, one patient described the enclosed experience as being *"It's like being in a coffin. You know, I did think that. It's like being in a coffin. But I wasn't. You just have to think that… Because if you've got something really, really bad, it would be like being in a coffin. Just like thinking, 'my God. This is going to be me'. You know. And I would have thought that could be quite scary*" s16 (60-69, male, C), at this point the patient, recently diagnosed with colorectal cancer became tearful. Another patient interpreted MRI sounds to indicate the presence of pathology *" And then you'll hear all these noises coming through you, so you wonder…, I thought, if the machine is clicking on, may be something is wrong there with me"* s13 (60-69, female, C).

For peer review only - http://bmjopen.bmj.com/site/about/guidelines.xhtml

Explaining differences in experiences (see Figure One)

Cancer type: Physical symptoms reported more frequently by those recruited to 'XX-L' were associated with discomfort, "*I found lying flat on my back, yes, very uncomfortable... I was coping okay for about 20, 25 minutes and then that's when this pressure or aching or uncomfortable feeling on my chest began to kick in"*s30 (50-59, male, L) and difficulty with breath holding "*they had to make me do it a few times because I couldn't hold it for the length of time they wanted me to … I've got COPD, whether that made it worse, I don't know"* s58 (60-69, male, L).

Existing musculo-skeletal problems: Sometimes these made the WB-MRI more uncomfortable, but not for all: *"I do suffer a bit from back pain… My back started to sort of be pretty uncomfortable*" s42 (60-69, male, C), and *"I was worried about my neck being uncomfortable … because of arthritis but they managed to sort me out and then I managed to last the hour*"s18 (60-69, male, C).

Mental health co-morbidities: One patient with prior anxiety found the scan difficult, describing their anxiety as causing feelings of claustrophobia "*I'm not claustrophobic. I just felt that way because of the anxiety. When I get anxious, it feels like everything's just closing in on me"* s54 (<50, female, L).

Prior experiences: One patient terminated the scan because he didn't like being constrained and his aversion came as a surprise, *"a sense of being constrained and being strapped down which is a new one for me… I haven't encountered that situation before"*s17 (60-69, male, L). In contrast, another participant (s8: 60-69, male, C) who knew he suffered from severe claustrophobia requested a sedative and completed the scan with relative ease. Work experiences meant some patients were used to confined spaces and/or loud noises, *"it doesn't*

For peer review only

*bother me. I've worked in pipes and tunnels and all sorts of places"*s91 (60-69, male, L) and "*if I wasn't in the trade I suppose that that could really freak you out because it's quite loud…"* s76 (<50, male, C). Vicarious experiences were sources of knowledge for patients without prior personal experience, and influenced expectations, sometimes reassuring "*I have a few friends and relatives… they have already done MRI, so I had something from them. this [is not] painful or anything like that. "* s19 (60-69, male, C); sometimes contrary to their own eventual experience, *"it was not as intimidating as I thought… I had heard people talking about going in to an MRI scanner, and they were telling me how nervous they were, and how they hated it…. And when I saw it, I thought, 'That's okay'"* s18 (60-69, male, C).

Staff contact: Staff supported patients with verbal reassurance, information provision, as well as making them physically comfortable. For example, "*they told me all about it so I didn't get anxious. I knew what to expect"* s26 (60-69, female, L), and *"they managed to find something to put under my neck…they did put it in the right place under my neck and I was okay"* s18 (60-69, male, C). Staff communication acted as a distraction *"the person speaking keeps my mind occupied"* s26 (60-69, female, L). Some patients spoke of a sense of isolation when in the scanner and hearing a voice confirmed they were not alone *"just made me feel a bit confident that you wasn't on your own, you know?"* s58 (60-69, female, L). Patients varied in whether they felt they received enough information *"they told me everything I needed to know"* s76 (<50, male, C) versus *"there wasn't any [information]…that was the lacking part*" s35 (50-59, female, L). Whether staff were perceived to be reassuring was also important for some and a change in staff mid-way through the scan was detrimental: "*she was constantly talking to me, I was fine. But then … another lady took over and she didn't talk as much … …when I panicked"* s58 (60-69, female, L) and *"People I've been speaking with beforehand… I've actually built up the initial … trust… Then, suddenly, a complete stranger is now telling me to breathe in and out… then said, ' I should be coming down now to inject*

For peer review only - http://bmjopen.bmj.com/site/about/guidelines.xhtml

you with dye'… "In my mind, I thought the absolutely bizarre" s80 (60-69, male, C). Failure to heed a patient preference influenced their decision to terminate the scan *"…they told me that they were going to inject dye. And once again I said 'don't put that cannula in my left arm' but she did and the vein collapsed. And I said to them 'I can't do any more now… I've had enough'"* s30 (50-59, Male, L).

Coping: Patients adopted various coping strategies, categorised into two main groups: 1) strategies for coping with distressing thoughts, emotional responses, or physical sensations, e.g. mental distraction and relaxation. *"Because I love Cyprus I was thinking of Cyprus the whole time. And it sort of takes your mind off of what's going"*s55 (60-69, female, L) and *"breathing, breathing…And you know trying to sort of just to calm…Because you can't help but get anxious"*s60 (60-69, female, L); and 2) Strategies related to motivation to complete the scan, such as focusing on benefit beliefs: "*I was so fed up with pain that I would have done…you know, any investigation was better… it was [WB-MRI] something to get me better"*s14 (60-69, female, C). *"I was laying there and I was thinking, 'Well, it's for my own benefit so I can live with that'"*s8 (60-69, male, C) and "*knowing I had cancer, you know, that's probably the most frightening thing that probably anyone could be told… So, this [WB_MRI]was all a culmination of things that's going to help me get better….The bed rocking a little bit, these loud noises really palled into insignificance because in my body now, I've got a nasty little house guest, which has now stayed, not welcome. I'm going to rid. And this is part of the mechanism to get rid…I was totally focused on the cancer. And these are the pictures that would help me get that done"* s80 (60-69, male, C).

Patients undergoing the WB-MRI scan were not only faced with the challenge of a potentially new technology, but were also coping with a possible or recent cancer diagnosis. For some, this influenced the challenge posed by the WB-MRI; "*it's been you know really anxious time.*

For peer review only - http://bmjopen.bmj.com/site/about/guidelines.xhtml

It's not just going to have an MRI. You obviously know something is wrong with and you're hoping that there's only going to be one thing wrong with you and then you get two things [tests prior to WB-MRI found a spine tumour in addition to lung cancer]. And all of a sudden, it's a completely different ballgame you know for me. About that stage, I had realised that I probably wasn't going to be able have the operation and just carry on with my life as normal. .... which is why I was finding it so hard to relax. It wasn't the MRI, it was just me. ...you're stressed out" s60 (60-69, female, L).

Willingness to have another WB-MRI Scan

All patients stated they would undergo another WB-MRI scan if the doctor recommended it, including the four patients who had requested scan termination. However this agreement was offered with varying enthusiasm; *"well if I have to....I don't like it one bit but if it has to be done"* s73 (60-69, male, L) compared with *"yes without hesitation"* s63(<50,male,C).

CONCLUSIONS

We aimed to describe patient experience and potential acceptability of WB- MRI as a potential replacement for the modalities currently used for staging lung and colorectal cancer. Patients recognised that WB-MRI was different from other scans although the extent of the challenge varied considerably. The majority completed WB-MRI with just four patients requesting premature termination; all were prepared to attempt future WB-MRI. Patients adopted a variety of coping strategies. These centred on the physical and emotional responses experienced during scanning, as well as focusing on beliefs that bolstered motivation to complete the scan. The role of staff was vital to support vulnerable patients; positive interactions bolstered coping while negative interactions had the opposite effect.

For peer review only - http://bmjopen.bmj.com/site/about/guidelines.xhtml

To our knowledge, there are only two other comparable qualitative studies of patients' experiences of MRI (19,20). In common with our study the enclosed space, noise, physical discomfort and duration were indentified as challenging, and the need for staff support was also highlighted. The importance of a "trustful dialogue" between radiographer and patients, to facilitate coping strategies (19), resonate with comments made by patients in our study.

Noise and confinement are the most frequently cited negative aspects of MRI in quantitative work published over the last 20 years (6,12). Other studies identify additional factors increasing the likelihood of premature scan termination, including acquisition position (head first, and/or prone); age and gender (middle age or female); prior experience (first scan or prior negative experience) and comorbidity (pain, anxiety) (6,13,23,24). Our results concur with some of these findings; we found evidence that co-morbidity (e.g. anxiety and physical symptoms) influenced experience although, unlike others (e.g. (12,14)) prior experience (positive or negative) generally increased coping ability.

Our study however adds to the existing literature. Our patient cohort were highly suspected or just diagnosed with cancer, and we were able to elicit influences of this on their experience of MRI. Furthermore the specific scan type under investigation (WB MRI) differs from simpler MRI scans such as knees and spine, based on its longer duration and need for receiver coils to cover the whole body. Patients described how the WB-MRI had the potential to reveal a cancer diagnosis or additional metastatic disease, with treatment implications. This caused anxiety and a dilemma as to whether to have the scan. However some patients also described relief and gratitude for having the scan either when it found no additional cancer or indeed when it diagnosed additional metastatic disease. The perceived length of the scan, together with its title of "whole body" may have added to patients perceptions about its ability to influence their treatment and prognosis. The WB-MRI scanner was likened to a coffin or

For peer review only - http://bmjopen.bmj.com/site/about/guidelines.xhtml

sarcophagus and it is possible that these observations were more emotive because the patients were contemplating a life threatening diagnosis. Carlsson and Carlsson (19) reported greater fear when MRI was used to confirm serious illness. WB-MRI for cancer staging comes at a point when patients are likely to be emotionally vulnerable, stretching coping resources for challenging medical procedures. However, while the implications of scanning patients with co-morbidities and emotional vulnerability should be acknowledged, it is important to note that most completed the WB-MRI.

Our study suggests that good staff communication could reduce anxiety and boost coping strategies by acting as a source of distraction, motivation and emotional reassurance. The varied experiences encountered underline the need for staff interaction to be tailored. Advanced staff training to build rapport can reduce MRI non-completion rates and increase patient satisfaction (25,26). A recent UK survey suggests that whilst strategies are in place to help reduce MRI related anxiety (commonly written leaflets, verbal pre-scan information, music during scanning, dedicated staff support), these are not optimised yet, with resource restrictions a potential barrier to implementation (27).

Study limitations

The generalisability of our results may be limited. WB-MRI was performed within the context of a research trial and some patients declined participation citing claustrophobia. Acceptability of WB-MRI may be lower than our study suggests. However the patients were from a wide age range and deprivation levels, and were interviewed from a number of different hospitals. As a result, a diverse spread of experiences were documented and included some who ultimately were unable to tolerate scanning.

Clinical implications

For peer review only - http://bmjopen.bmj.com/site/about/guidelines.xhtml

Our study confirms that WB-MRI can be a challenging experience and that staff support is important in modifying scan related stress. Our study also highlights that patient experience is varied and for the most part, the scan was tolerated, suggesting WB-MRI could potentially replace existing modalities, assuming adequate diagnostic accuracy and cost-effectiveness. Experience of WB-MRI reflects the success of coping strategies adopted by patients and the quality of support they receive. Further research would facilitate understanding of the interrelationship between patients' experiences, the effectiveness of their coping strategies, and of the support received from staff such that patients and clinicians can benefit maximally from the diagnostic potential of WB-MRI.

Figure One: A model illustrating the factors that influence a patient's WB-MRI experience.

For peer review only

Reference List

(1) Brocken P, Prins JB, Dekhuijzen PN et al.The faster the better?-A systematic review on distress in the diagnostic phase of suspected cancer, and the influence of rapid diagnostic pathways. *Psychooncology* 2012 Jan;21(1):1-10.

(2) Brocken P, van der Heijden EH, Oud KT et al.Distress in suspected lung cancer patients following rapid and standard diagnostic programs: a prospective observational study. *Psychooncology* 2015 Apr;24(4):433-41.

(3) Risberg T, Sorbye SW, Norum J et al.Diagnostic delay causes more psychological distress in female than in male cancer patients. *Anticancer Res* 1996 Mar;16(2):995-9.

(4) Ciliberto M, Maggi F, Treglia G, et al. Comparison between whole-body MRI and Fluorine-18-Fluorodeoxyglucose PET or PET/CT in oncology: a systematic review. *Radiol Oncol* 2013 Jul 30;47(3):206-18.

(5) Katz RC, Wilson L, Frazer N. Anxiety and its determinants in patients undergoing magnetic resonance imaging. *J Behav Ther Exp Psychiatry* 1994 Jun;25(2):131-4.

(6) MacKenzie R, Sims C, Owens RG et al. Patients' perceptions of magnetic resonance imaging. *Clin Radiol* 1995 Mar;50(3):137-43.

(7) McIsaac HK, Thordarson DS, Shafran R et al.Claustrophobia and the magnetic resonance imaging procedure. *J Behav Med* 1998;21(3):255-68.

(8) Melendez JC, McCrank E. Anxiety-related reactions associated with magnetic resonance imaging examinations. *JAMA* 1993 Aug 11;270(6):745-7.

(9) van MD, Klaming L, Weda H. Pinpointing moments of high anxiety during an MRI examination. *Int J Behav Med* 2014 Jun;21(3):487-95.

(10) Dewey M, Schink T, Dewey CF. Claustrophobia during magnetic resonance imaging: cohort study in 55,000 patients. *J Magn Reson Imaging* 2007;38(3):722-5.

(11) Eshed I, Althoff CE, Hamm B et al. Claustrophobia and premature termination of magnetic resonance imaging examinations. *J Magn Reson Imaging* 2007 Aug;26(2):401-4.

(12) Harris LM, Cumming SR, Menzies RG. Predicting anxiety in magnetic resonance imaging scans. *Int J Behav Med* 2004;11(1):1-7.

(13) Kilborn LC, Labbe EE. Magnetic resonance imaging scanning procedures: development of phobic response during scan and at one-month follow-up. *J Behav Med* 1990 Aug;13(4):391-401.

(14) Lukins R, Davan IG, Drummond PD. A cognitive behavioural approach to preventing anxiety during magnetic resonance imaging. *J Behav Ther Exp Psychiatry* 1997 Jun;28(2):97-104.

For peer review only - http://bmjopen.bmj.com/site/about/guidelines.xhtml

(15) Wiljer D, Walton T, Gilbert J et al.Understanding the needs of colorectal cancer patients during the pre-diagnosis phase. *J Cancer Educ* 2013;28(3):402-7.

(16) Lehto RH. Identifying Primary Concerns in Patients Newly Diagnosed With Lung Cancer. *Oncology Nursing Forum* 2011;38(4):440-7.

(17) Robb KA, Simon AE, Miles A et al.Public perceptions of cancer: a qualitative study of the balance of positive and negative beliefs. *BMJ Open* 2014;4(7):e005434.

(18) Martin MY., Fouad MN, Oster RA et al.What do cancer patients worry about when making decisions about treatment? Variation across racial/ethnic groups. *Support Care Cancer* 2014;22(1):233-44.

(19) Carlsson S, Carlsson E. 'The situation and the uncertainty about the coming result scared mebut interaction with the radiographers helped me through': a qualitative study on patients' experiences of magnetic resonance imaging examinations. *Journal of Clinical Nursing* 2013;22:3225-34.

(20) Tornqvist E, Mansson A, Larsson EM et al. It's like being in another world – patients' lived experience of magnetic resonance imaging. *Issues in Clinical Nursing* 2006;954-61.

(21) McLennan D, Barnes H, Noble M et al. The English Indices of Deprivation 2010. London: Department for Communities and Local Government; 2011.

(22) Braun V, Clarke V. Using thematic analysis in psychology. Qualitative Research in Psychology 2006; 3(2):77-101

(23) Mohlman J, Eldreth DA, Price RB et al. Predictors of unsuccessful magnetic resonance imaging scanning in older generalized anxiety disorder patients and controls. *J Behav Med* 2012;35:19-26.

(24) Thorpe S, Salkovskis PM, Dittner A. Claustrophobia in MRI: the role of cognitions. *Magnetic Resonance Imaging* 2008;26:1081-8.

(25) Elvira V.Lang. A Better Patient Experience Through Better Communication. *J Radiol Nurs* 2012;31((4)):114-9.

(26) Lang EV1, Ward C, Laser E. Effect of team training on patients' ability to complete MRI examinations. *Acad Radiol* 2010;17(1):18-23.

(27) Tischler V, Calton T, Williams M et al.Patient anxiety in magnetic resonance imaging centres: Is further intervention needed? *Radiography* 2008;14:265-6.

For peer review only - http://bmjopen.bmj.com/site/about/guidelines.xhtml

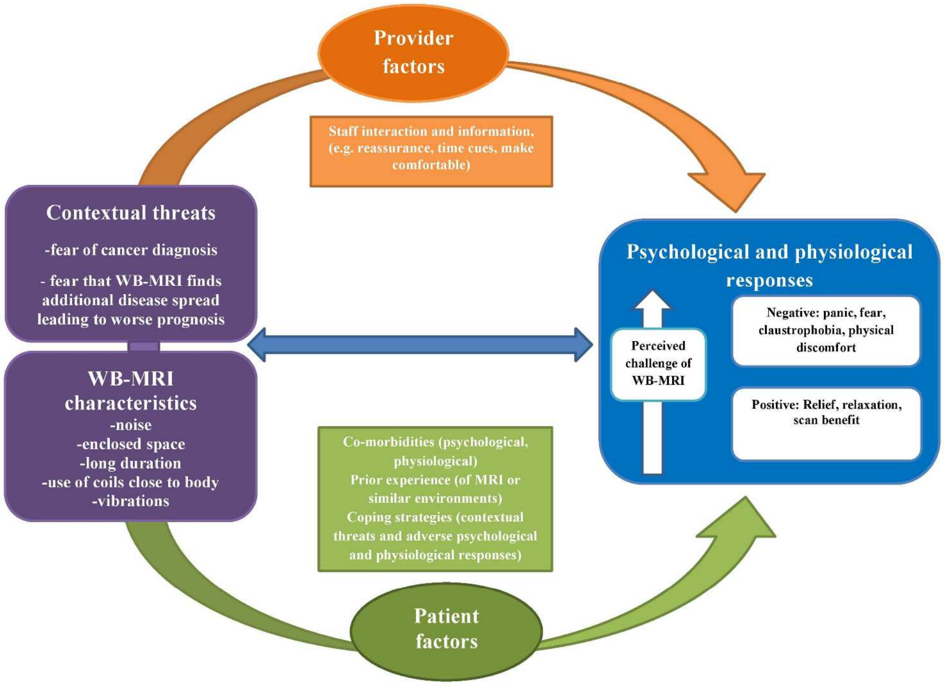

Figure 1: A model illustrating the factors that influence a patient's WB-MRI experience

297x210mm (300 x 300 DPI)

For peer review only

Streamline L

For peer review only

Streamlining Staging of Lung Cancer with Whole Body MRI

Trial Sponsor:	University College London (UCL)
Trial Sponsor reference:	UCL/12/0156
Trial funder:	NIHR HTA
Funder reference:	10/68/01
ISRCTN no:	50436483
Protocol version no:	6.0
Protocol version date:	06/09/2016

This project was funded by the National Institute for Health Research (NIHR) Health Technology Assessment (HTA) programme *(project number 10/68/01)*

Protocol version 6.0, 06/09/2016 Authorisation signatures

Name & Role:	Signature:	Date authorised:
**Chief Investigator:**<br>Professor Stuart Taylor<br>Professor of Medical Imaging, UCL		14/10/16
**For the Sponsor:**<br>Professor Jonathan Ledermann<br>Director, UCL CTC		14OCT2016
Sandy Beare<br>Tumour Group Lead, UCL CTC		14/10/2016

Please note: This trial protocol must not be applied to patients treated outside the Streamline L trial. UCL CTC can only ensure that approved trial investigators are provided with amendments to the protocol.

For peer review only - http://bmjopen.bmj.com/site/about/guidelines.xhtml

Coordinating Centre:

For general queries, supply of trial documentation and central data management please contact:

Streamline L Trial Coordinator
Cancer Research UK & UCL Cancer Trials Centre (UCL CTC)
90 Tottenham Court Road
London
W1T 4TJ

Tel: +44 (0) 20 7679 9878
Fax: +44 (0) 20 7679 9871
09:00 to 17:00 Monday to Friday

Email: ctc.streamlineL@ucl.ac.uk

Other trial contacts:

Chief Investigator: Professor Stuart Taylor
Address: Dept Specialist X Ray, Level 2 Podium,
University College London Hospital (UCLH)
235 Euston Road
London
NW1 2BU

Trial Management Group (TMG):

Prof Stuart Taylor	Professor of Medical Imaging	UCLH
Dr Dow-Mu Koh	Consultant Radiologist	The Royal Marsden
Prof Vicky Goh	Professor of Clinical Cancer Imaging	Guy's and St Thomas'
Prof Anwar Padhani	Clinical Lead in MRI	Mount Vernon
Dr Shonit Punwani	Senior Lecturer in Medical Imaging	UCLH
Prof Andrea Rockall	Professor of Cancer Imaging	Imperial College London
Prof Steve Halligan	Professor of Radiology	UCL
Dr Neal Navani	Consultant in Thoracic Medicine	UCLH
Prof Sam Janes	Wellcome Senior Fellow	UCLH
Dr John Bridgewater	Consultant Oncologist	UCLH
Alison Morton	Patient Representative	
Alf Oliver	Patient Representative	
Dr Anne Miles	Senior Lecturer in Health Psychology	Birkbeck College
Prof Steve Morris	Professor of Health Economics	UCL
Dr Susan Mallett	Senior Medical Statistician	University of Birmingham
Krystyna Reczko	Trial Coordinator	UCL CTC
Katie Prior	Data Manager	UCL CTC
Marian Duggan (maternity leave)	Senior Trial Coordinator	UCL CTC
Abby Sharp (maternity cover)	Senior Trial Coordinator	UCL CTC
Dr Sandy Beare	Tumour Group Lead	UCL CTC

For peer review only - http://bmjopen.bmj.com/site/about/guidelines.xhtml

Table of Contents

1. PROTOCOL SUMMARY 7
1.1. SUMMARY OF TRIAL DESIGN 7
1.2. TRIAL SCHEMA 9
2. INTRODUCTION 10
2.1. BACKGROUND 10
3. TRIAL OVERVIEW 12
3.1. PRIMARY OBJECTIVE 12
3.2. SECONDARY OBJECTIVES 12
3.3. TRIAL ACTIVATION 13
4. SELECTION OF SITES/SITE INVESTIGATORS 14
4.1. SITE SELECTION 14
4.1.1. Selection of Principal Investigator and other investigators at sites 14
4.1.2. Training requirements for site staff 14
4.2. SITE INITIATION AND ACTIVATION 15
4.2.1. Site initiation 15
4.2.2. Required documentation 15
4.2.3. Site activation letter 15
5. INFORMED CONSENT 16
6. SELECTION OF PATIENTS 18
6.1. PRE-REGISTRATION EVALUATION 18
6.2. SCREENING LOG 18
6.3. MULTI DISCIPLINARY (MDT) MEETING LOG 18
6.4. PATIENT ELIGIBILITY 18
6.4.1. Patient Inclusion Criteria 18
6.4.2. Exclusion Criteria 19
6.4.3. Pregnancy and Birth Control 19
7. REGISTRATION PROCEDURES 20
7.1. REGISTRATION 20
7.2. REGISTRATION PACKS 20
7.3. BOOKING WHOLE BODY MRI 20
8. PATIENT MANAGEMENT AND TRIAL PROCEDURES 22
8.1. SUMMARY 22
8.1.1. Staging and Treatment Decisions 22
8.1.2. Patient experience Interviews and Questionnaires (Optional) 23
8.1.3. Quality of Life Questionnaires and Patient Diaries 23
8.2. DETAILED DESCRIPTION 25
8.2.1. Standard care 25
8.2.2. Trial Imaging 25
8.2.3. Release of Conventional and trial WB-MRI findings 26
8.2.4. Final treatment plan 28
8.2.5. Early release of WB-MRI findings 28
8.2.6. Time to full staging 29
8.2.7. Patient follow up 29
8.2.8. Patient diaries and EQ-5D Quality of Life questionnaires 30
8.2.9. Final reference standard for tumour stage (Consensus Meetings) 30

For peer review only - http://bmjopen.bmj.com/site/about/guidelines.xhtml
For peer review only

8.2.10. Health psychology assessment ... 31
8.2.11. Cost Effectiveness Assessment ... 34
8.2.12. Nested substudies ... 36

9. DATA MANAGEMENT GUIDELINES ... 38

9.1. COMPLETING CASE REPORT FORMS ... 38
9.2. MISSING DATA ... 38
9.3. TIMELINES FOR DATA RETURN ... 38
9.4. DATA QUERIES ... 39

10. SAFETY REPORTING ... 40

11. INCIDENT REPORTING ... 41

12. TRIAL MONITORING AND OVERSIGHT ... 42

12.1. CENTRAL MONITORING ... 42
12.2. 'FOR CAUSE' ON SITE MONITORING ... 42
12.3. OVERSIGHT COMMITTEES ... 43
12.3.1. Trial Management Group (TMG) ... 43
12.3.2. Trial Steering Committee (TSC) ... 43
12.3.3. Independent Data Monitoring Committee (IDMC) ... 43
12.3.4. Role of UCL CTC ... 43

13. WITHDRAWAL OF PATIENTS ... 44

14. TRIAL CLOSURE ... 45

14.1. END OF TRIAL ... 45
14.2. ARCHIVING OF TRIAL DOCUMENTATION ... 45
14.3. EARLY DISCONTINUATION OF TRIAL ... 45
14.4. WITHDRAWAL FROM TRIAL PARTICIPATION BY A SITE ... 45

15. QUALITY ASSURANCE ... 46

16. STATISTICS ... 47

16.1. SAMPLE SIZE CALCULATION ... 47
16.2. POPULATION FOR ANALYSIS ... 48
16.3. ANALYSIS OF THE PRIMARY ENDPOINT ... 49
16.4. ANALYSIS OF SECONDARY ENDPOINTS ... 50
16.4.1. Efficacy (secondary) ... 50
16.4.2. Safety ... 51
16.4.3. Economic evaluation ... 51
16.4.4. Health psychology assessment ... 52
16.4.5. Handling missing data ... 53
16.5. INTERIM ANALYSES ... 53

17. ETHICAL APPROVALS ... 55

17.1. ETHICAL APPROVAL ... 55
17.2. SITE APPROVALS ... 55
17.3. PROTOCOL AMENDMENTS ... 55
17.4. PATIENT CONFIDENTIALITY & DATA PROTECTION ... 56

18. SPONSORSHIP AND INDEMNITY ... 57

18.1. SPONSOR DETAILS ... 57
18.2. INDEMNITY ... 57

19. FUNDING ... 58

20. PUBLICATION POLICY ... 59

For peer review only - http://bmjopen.bmj.com/site/about/guidelines.xhtml

Streamline L

21. REFERENCES 60
APPENDIX 1: ABBREVIATIONS 63
APPENDIX 2: CONSENT PROCESS 65
APPENDIX 3: MINIMUM WB-MRI PROTOCOL DATASET 67
APPENDIX 4: EXPLORATORY ANALYSIS 69
APPENDIX 5: PROTOCOL VERSION HISTORY 71

For peer review only

For peer review only - http://bmjopen.bmj.com/site/about/guidelines.xhtml

1. Protocol Summary

1.1. Summary of Trial Design

**Title:**	Streamlining Staging of Lung Cancer with Whole Body MRI
**Short Title/acronym:**	Streamline L
**Sponsor name & reference:**	UCL/12/0156
**Funder name & reference:**	NIHR HTA
**ISRCTN no:**	50436483
**Design:**	Multicentre comparison
**Overall aim:**	To evaluate whether early whole body magnetic resonance Imaging (WB-MRI) increases per patient sensitivity for metastasis in non-small cell lung cancer compared to standard NICE-approved diagnostic pathways.
**Primary endpoint:**	Per patient sensitivity for metastasis detection by whole body MRI (WB-MRI) compared to standard staging pathways in newly diagnosed non-small cell lung cancer
**Secondary endpoints:**	1. The time and test number taken to reach, and the nature of, the first major treatment decision based on WB-MRI in comparison to standard staging pathways.<br>2. Diagnostic accuracy of WB-MRI and conventional staging pathways for local tumour staging and detection of metastasis in comparison to an expert derived consensus reference standard.<br>3. Lifetime incremental cost and cost-effectiveness of staging using WB-MRI compared to standard diagnostic pathways.<br>4. Patient experience of staging using WB-MRI in comparison to standard diagnostic pathways and priorities placed by patients on differing attributes related to competing staging pathways.<br>5. Inter-observer variability in WB-MRI analysis and effect of diagnostic confidence on staging accuracy.<br>6. Diagnostic accuracy of limited T1 and diffusion weighted sequences compared to full multi-sequence WB-MRI protocols.
**Target accrual:**	353

For peer review only - http://bmjopen.bmj.com/site/about/guidelines.xhtml

**Inclusion & exclusion criteria:**	**Inclusion criteria:**<br>• Adult patients (18 or over) with suspected primary non-small cell lung cancer on chest CT with sufficient confidence to trigger staging investigations/biopsy OR with already histologically proven primary non-small cell lung cancer<br>• Disease is potentially radically treatable as defined as stage IIIb or less on diagnostic CT (i.e. T1-4, N0-2, M0)<br>• Performance status 0-2 (fit to undergo radical treatment if indicated)<br>• Patient must have given written informed consent and be willing to comply with the protocol intervention and follow up.<br>**Exclusion criteria:**<br>• Any psychiatric or other disorder likely to impact on informed consent<br>• Evidence of severe or uncontrolled systemic disease which makes it undesirable for the patient to participate in the trial<br>• Pregnancy<br>• Contraindications to MRI (e.g. cardiac pacemaker, severe claustrophobia, inability to lie flat)<br>• Unequivocal metastatic or N3 disease on diagnostic CT chest and abdomen (including M1a disease; malignant pleural effusion)<br>• Further staging work up not indicated in the opinion of the MDT due to poor performance status or patient choice<br>• Histologies other than non-small cell lung cancer.
**Planned number of sites:**	17 (including 7 Imaging hubs)
**Target Country**	UK
**Trial Procedure:**	All patients will undergo a whole body MRI protocol in addition to the standard staging protocol employed at their institution.
**Anticipated duration of recruitment:**	4 years
**Duration of patient follow up:**	12 months
**Definition of end of trial:**	12 months after enrolment of the final patient.
**Other related research:**	Sub-study of WB-MRI generalisability

For peer review only - http://bmjopen.bmj.com/site/about/guidelines.xhtml

For peer review only

1.2. Trial Schema

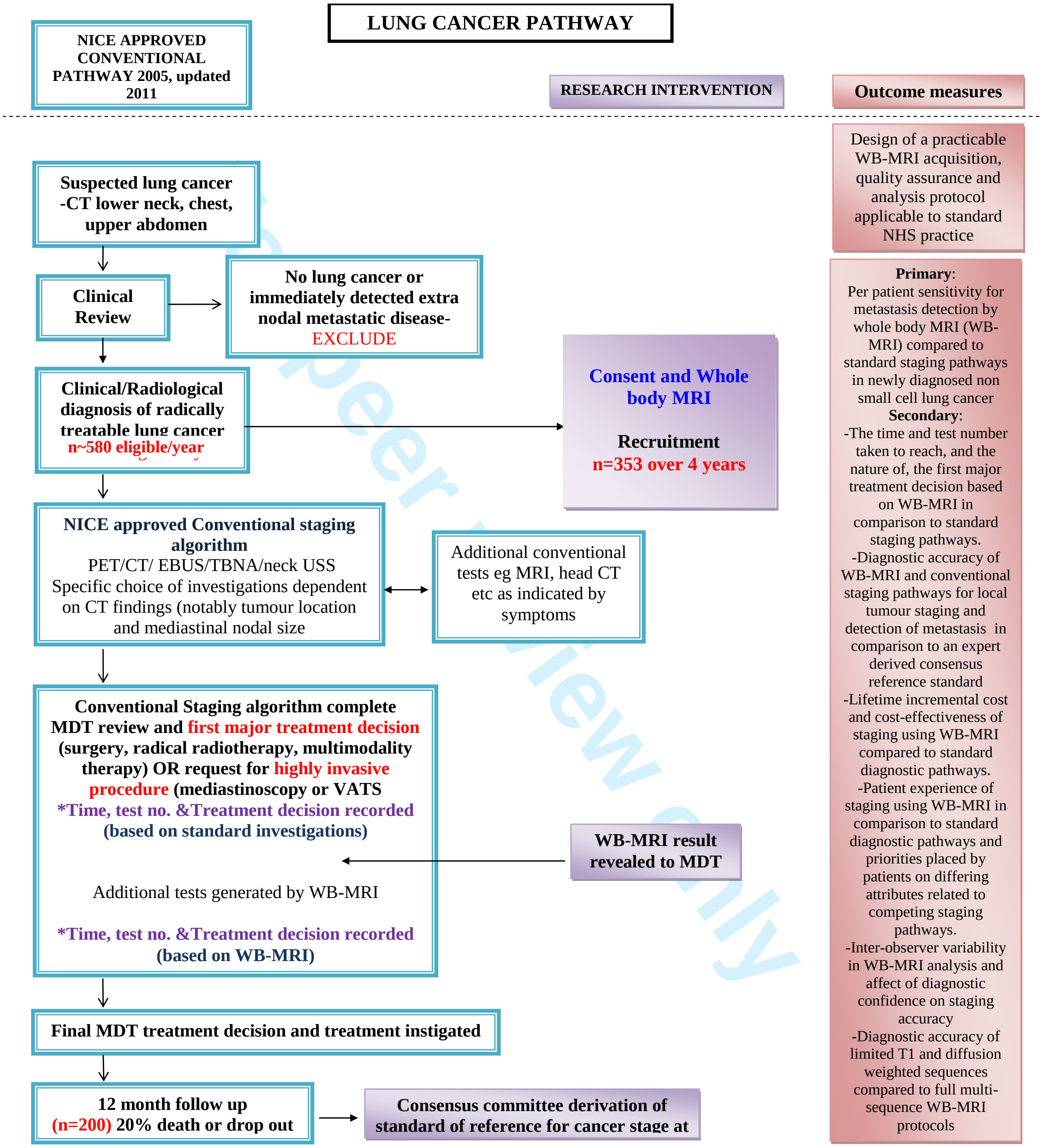

Key: EBUS= Endobronchial Ultrasound. TBNA= transbronchial needle aspiration. USS=ultrasound VATS= video-assisted thoracoscopic surgery

For peer review only - http://bmjopen.bmj.com/site/about/guidelines.xhtml

2. Introduction

2.1. Background

Lung cancer is the second most common malignancy in the UK and accounts for 13% of all new cancer diagnosis; approximately 40,000 patients are diagnosed per year [1]. Treatment decisions for lung cancer are critically dependent upon rapid and accurate tumour staging at diagnosis. In particular detection of metastatic disease (i.e. disease which has disseminated beyond the primary tumour into distant organ sites such as bone, liver and brain) is vital to optimised therapeutic triage. Although 1 year lung cancer survival in the UK is just 33% [2], correctly staged and treated early stage disease (i.e. without metastatic spread) achieves between 54 and 80% 5 year survival. Conversely, currently over 20% of patients undergoing “curative” thoracotomy for presumed early stage disease rapidly relapse with metastatic spread undetected by conventional staging tests - so called “futile thoracotomy” [3]. Accurate local staging of the tumour and spread to lymph nodes is also of importance. For example in patients with non-small cell lung cancer, spread to contra lateral mediastinal lymph nodes precludes surgery with curative intent.

Staging of lung cancer within the NHS is reliant on high technology imaging platforms such as computerised tomography (CT), positron emission tomography (PET) and magnetic resonance imaging (MRI), together with standard scintigraphy, plain X-Rays and ultrasound. Minimally invasive tissue sampling techniques such as endobronchial ultrasound (EBUS) are also increasingly used. Staging pathways are complex (see flowchart, section 1.2), and governed by NICE approved guidelines [2] which delineate an integrated step by step deployment of the various imaging modalities before tumour staging is deemed complete and the first treatment decision is made. The complexity of current staging algorithms reflects the differing diagnostic attributes of the various imaging modalities across tumour types and body organs. CT for example is superior to ultrasound for detecting liver metastasis [4], but inferior to scintigraphy in diagnosing malignant spread to the bone. PET scanning is in general superior to scintigraphy for detecting bone metastases [5], but not as effective as MRI in identifying spread to the brain [6]. It is not unusual for a patient newly diagnosed with lung cancer to undergo standard chest and abdominal CT, whole body PET, brain CT, and invasive mediastinal nodal sampling before the first definitive treatment is decided. Such a stepwise multi-modality approach is time and resource intensive and onerous for patients at a very difficult time following their cancer diagnosis. Furthermore patients receive significant doses of diagnostic ionising radiation during the staging process - a single PET-CT imparts 6-10 times normal annual background radiation and even small doses of radiation can increase an individual’s risk of subsequent malignancy [7].

Recent technological advances mean an MRI scan of the whole body can be acquired in less than one hour; sensitivity for metastatic cancer spread is high [8]. MRI does not impart ionising radiation and is advocated as a safe and effective alternative to the current multi-modality approach. There is no available secondary research evidence concerning the accuracy of WB-MRI in cancer staging. Indeed meta-analysis would be challenging because literature is limited to relatively small cohort studies comparing WB-MRI with conventional imaging tests, and in a wide variety of cancers. These do however suggest potential as an efficient and accurate “one stop shop” alternative for

For peer review only - http://bmjopen.bmj.com/site/about/guidelines.xhtml

cancer staging [6, 9, 10]. For example, in 123 patients with non-small cell cancer, Yi found no difference between PET-CT and WB-MRI, both achieving 86% accuracy [10]. Notably, the vast majority of available studies are single site, using one MRI platform, with interpretation by one or two experienced radiologists. The literature is deficient regarding generalisability of WB-MRI. For example, variability amongst interpreting radiologists is unknown and the effect (if any) of different manufacturers' platforms is uncertain. It is also unclear how WB-MRI can be best integrated into existing NHS pathways, which frequently differ between institutions, or how effective it would be in different clinical settings. By undertaking a multi–centre trial of lung cancer using a variety of MRI platforms and interpreting radiologists, the current proposal will address these unknown variables.

Health psychology assessment

WB-MRI staging of lung cancer could reduce the physical and psychological burden associated with current pathways. By reducing time from diagnosis to treatment, WB-MRI may reduce patient anxiety, minimising concerns that treatment delay might adversely affect prognosis. Delay in diagnosis is known to be distressing, particularly for women [11]. In addition, staging scans can be uncomfortable and embarrassing [12]. MRI for example, while avoiding radiation, is noisy and claustrophobic. The potential of WB-MRI to generate additional tests is also an important consideration, although recent data suggest test sensitivity is the key determinant of patient choice and influences preference more than discomfort and complications [13]. While rapid staging means patients without metastasis are in receipt of good news more quickly, it also means those with metastasis will also receive bad news earlier, which may impact adversely on psychological wellbeing: It is known that patients are more depressed following a speedier diagnosis of breast cancer (at a one-stop clinic) when compared to those waiting a week [14].

Cost Effectiveness Analysis

Although WB-MRI is a relatively expensive imaging technology, other standard tests in the staging pathways such as PET-CT are also resource intensive. A detailed analysis of the cost and the cost-effectiveness of WB-MRI versus standard NICE-approved staging algorithms conforming to accepted economic evaluation methods [15] is essential to any trial investigating the role of WB-MRI in cancer staging.

For peer review only - http://bmjopen.bmj.com/site/about/guidelines.xhtml

3. Trial Overview

This trial will be an independent multi-centre prospective cohort trial comparing the staging accuracy of early WB-MRI with standard pathways in newly diagnosed non-small cell lung cancer. We will follow the recommendations of the STAndards for the Reporting of Diagnostic accuracy studies (STARD) initiative [16]. A key element of our design is the investigation of early WB-MRI as a replacement test for standard pathways [17], and data regarding the therapeutic impact of WB-MRI will be collated, as well as addressing the practicalities of general NHS implementation. The trial design, which compares two different staging strategies in the same patients ensures that clean, comparable data are collected for both pathways while simultaneously increasing trial power to meet trial endpoints.

In addition, both qualitative and quantitative assessments will be employed to determine the psychological burden and acceptability of WB-MRI versus standard pathways, and to identify those attributes which most strongly influence patient preference. Health-related quality of life data will be collected which will be used to inform cost-effectiveness modelling, and determine whether patients believe more rapid staging would have helped them cope better with their cancer diagnosis.

3.1. Primary objective

To evaluate whether early whole body magnetic resonance Imaging (WB-MRI) increases per patient sensitivity for metastasis in non-small cell lung cancer compared to standard NICE-approved diagnostic pathways.

3.2. Secondary objectives

1) To determine how WB-MRI influences time to and nature of first major treatment decision following definitive staging compared to standard investigations and to determine whether early WB-MRI could reduce or replace standard investigations.
2) To assess the accuracy of WB-MRI and standard diagnostic pathways for local and distant staging by comparison with an expert derived consensus reference standard using 1-year patient follow up data.
3) To evaluate the lifetime incremental cost and cost-effectiveness of staging using WB-MRI compared to standard diagnostic pathways.
4) To evaluate patients' experiences of staging using WB-MRI and to determine the priorities placed by patients on differing attributes related to competing staging pathways, including impact of reducing time to first treatment.
5) To determine the effect of radiologist confidence on the diagnostic accuracy of WB-MRI for metastatic disease.
6) To evaluate the diagnostic performance of limited WB-MRI protocols based on diffusion and T1 weighted imaging only, and to assess the incremental benefit of intravenous gadolinium contrast enhancement.

For peer review only - http://bmjopen.bmj.com/site/about/guidelines.xhtml

Additional objectives which will be met by the trial will be the definition of an efficient WB-MRI acquisition and analysis protocol, and quality assurance (QA) program applicable to standard NHS practice and collation of a database of WB-MRI datasets for NHS staff training and service development.

3.3. Trial activation

UCL CTC will ensure that all trial documentation has been reviewed and approved by all relevant bodies and that the following have been obtained prior to activating the trial:

- Research Ethics Committee approval
- 'Adoption' into NIHR portfolio
- NHS permissions
- Adequate funding for central coordination
- Confirmation of sponsorship
- Adequate insurance provision

For peer review only

For peer review only - http://bmjopen.bmj.com/site/about/guidelines.xhtml

4. Selection of Sites/Site Investigators

4.1. Site selection

In this protocol a 'recruitment site' refers to a hospital where certain trial-related activities are conducted.

Recruitment sites must be able to comply with:

- Patient recruitment, follow up schedules and all requirements of the trial protocol
- Requirements of the Research Governance Framework
- Data collection requirements
- **No** trial imaging, recruitment sites will refer patients to designated imaging hub (see below)

In this protocol an 'imaging hub' refers to a hospital where certain trial-related activities are conducted.

Imaging hubs must be able to comply with:

- Patient recruitment (where applicable), follow up schedules (where applicable) and all requirements of the trial protocol
- Requirements of the Research Governance Framework
- Data collection requirements
- Trial imaging

4.1.1. Selection of Principal Investigator and other investigators at sites

Sites must have an appropriate Principal Investigator (PI), i.e. a health care professional authorised by the site and ethics committee, to lead and coordinate the work of the trial on behalf of the site. Other investigators at site wishing to participate in the trial must be trained and approved by the PI. All investigators must be medical doctors and have experience of diagnosing, staging or treating lung cancer.

4.1.2. Training requirements for site staff

All site staff must be appropriately qualified by education, training and experience to perform the trial related duties allocated to them, which must be recorded on the site delegation log.

CVs for all staff must be kept up to date, with signed and dated and copies held in the Investigator Site File (ISF). An up to date, signed copy of the CV for the PI must be forwarded to UCL CTC upon request.

All staff involved in the trial must receive GCP training which is relevant to their role and responsibilities within the trial. The frequency of repeat training may be dictated by the requirements of their employing institution, or 2 yearly where the institution has no policy, and more frequently when there have been updates to the legal or regulatory requirements for the conduct of clinical trials.

For peer review only - http://bmjopen.bmj.com/site/about/guidelines.xhtml

4.2. Site initiation and activation

4.2.1. Site initiation

Before a site is activated, the UCL CTC trial team will arrange a site initiation with the site which the PI and site research team must attend. The site will be trained in the day to day management of the trial, and essential documentation required for the trial will be checked.

Site initiation will be performed for each site by site visit or teleconference.

4.2.2. Required documentation

The following documentation must be submitted by the site to UCL CTC prior to a site being activated by UCL CTC trial team:

- Trial specific Declaration of Participation/Site Registration Form (identifying relevant local staff)
- All relevant institutional approvals (e.g. local NHS permission)
- A completed site delegation log that is initialled and dated by the PI
- A copy of the PI's current CV that is signed and dated

In addition, the following agreement must be in place:

- A signed Clinical Trial Site Agreement (CTSA) between the Sponsor and the relevant institution (usually a NHS Trust)

4.2.3. Site activation letter

Once the UCL CTC trial team has received all required documentation and the site has been initiated, a site activation letter will be issued to the PI. **Sites may not begin to approach patients until the site activation letter has been issued.**

Once the site has been activated by UCL CTC, the PI is responsible for ensuring:

- adherence to the most recent version of the protocol;
- all relevant site staff are trained in the protocol requirements;
- appropriate recruitment and medical care of patients in the trial;
- timely completion and return of CRFs (including assessment of all adverse events).

For peer review only - http://bmjopen.bmj.com/site/about/guidelines.xhtml

5. Informed consent

Sites are responsible for assessing a patient's capacity to give informed consent.

Sites must ensure that all patients have been given the current approved version of the patient information sheet, are fully informed about the trial and have confirmed their willingness to take part in the trial by signing the current approved consent form.

Sites must assess a patient's ability to understand verbal and written information in English and whether or not an interpreter would be required to ensure fully informed consent. If a patient requires an interpreter and none is available, the patient should not be considered for the trial.

The PI, or, where delegated by the PI, other appropriately trained site staff, are required to provide a full explanation of the trial to each patient prior to trial entry. During these discussions, the current approved patient information sheet for the trial should be discussed with the patient. A minimum of 24 hours before the WB-MRI must be allowed for the patient to consider and discuss participation in the trial.

The consent process has been left deliberately flexible in order to accommodate the needs of individual patients and variations in Site requirements. The flowchart below describes three different options for obtaining written informed consent from patients, each of which ensures that patients have at least 24 hours to consider their participation in the trial before any trial-related activities take place.

Streamline Recruitment Pathways/Consent Process

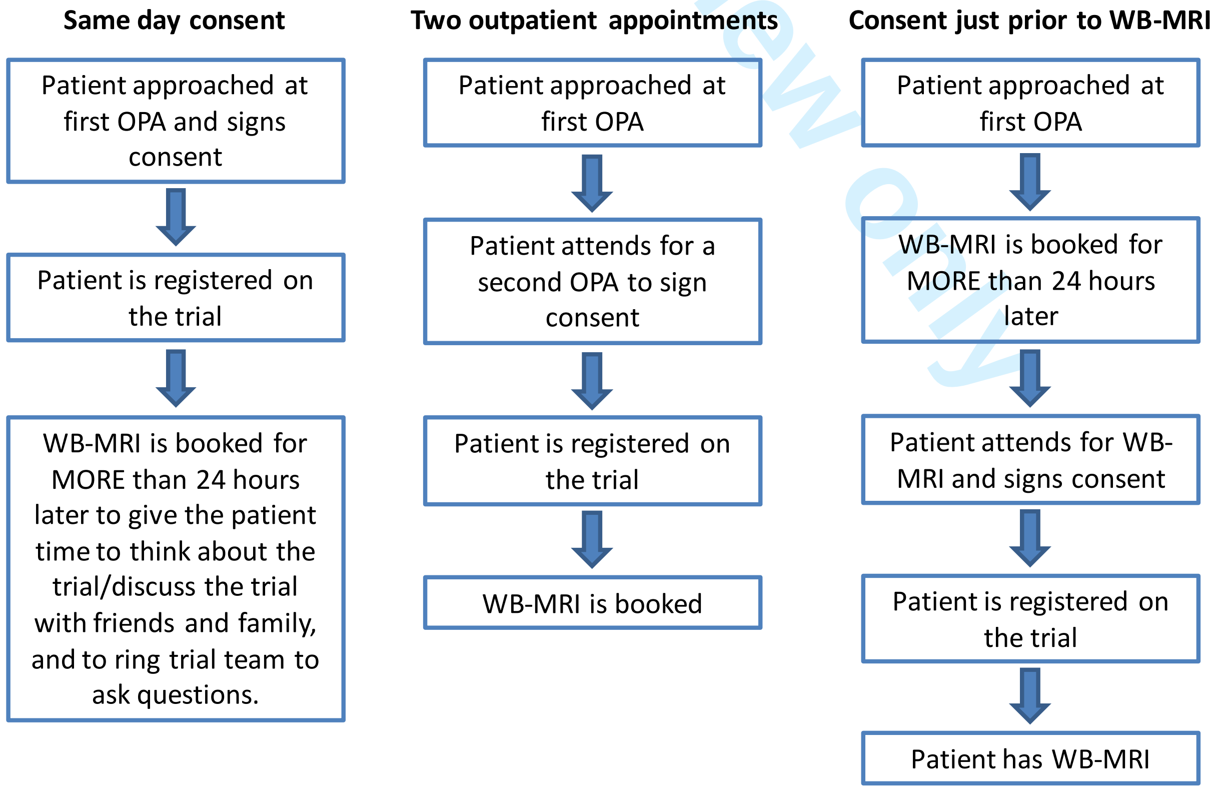

For peer review only - http://bmjopen.bmj.com/site/about/guidelines.xhtml

See Appendix 2 for a more detailed example of the consent process.

Written informed consent on the current approved version of the consent form for the trial must be obtained before any trial-specific procedures are conducted. The discussion and consent process must be documented in the patient notes.

Site staff are responsible for:

- checking that the correct (current approved) version of the patient information sheet and consent form are used;
- checking that information on the consent form is complete and legible;
- checking that the patient has completed/initialled all relevant sections and signed and dated the form;
- checking that an appropriate member of staff has countersigned and dated the consent form to confirm that they provided information to the patient;
- checking that an appropriate member of staff has made dated entries in the patient's medical notes relating to the informed consent process (i.e. information given, consent signed etc.);
- Following registration:
    - adding the patient trial number to all copies of the consent form, which should be filed in the patient's medical notes and investigator site file,
    - giving the patient a copy of their signed consent form and patient information sheet.

The right of the patient to refuse to participate in the trial without giving reasons must be respected. All patients are free to withdraw at any time. Also refer to section 13.0 (Withdrawal of patients).

For peer review only - http://bmjopen.bmj.com/site/about/guidelines.xhtml

6. Selection of Patients

6.1. Pre-registration Evaluation

The following assessments or procedures are required to evaluate the suitability of patients for the trial:

- Histologically proven or clinically diagnosed (defined as radiological diagnosis of lung cancer on chest CT with sufficient confidence to trigger staging investigations) primary non-small cell lung cancer with potentially radically treatable disease
- No contraindications to MRI (e.g. cardiac pacemaker, severe claustrophobia, inability to lie flat)

6.2. Screening Log

A screening log must be maintained by the site and kept in the Investigator Site File. This must record all potentially eligible patients approached about the trial and the reasons why they were not registered in the trial if this is the case. The log must be sent to UCL CTC in line with the monitoring plan and as requested, with patient identifiers removed prior to sending.

6.3. Multi Disciplinary (MDT) Meeting Log

An anonymous record of the number of patients with a new diagnosis of non-small cell lung cancer discussed in the MDT meeting, with their attributed cancer stage will be submitted annually to UCL CTC. This will be used to assess whether the group of patients recruited to the trial is representative of the general population of newly diagnosed lung cancer patients seen at Sites.

6.4. Patient Eligibility

There will be no exception to the eligibility requirements at the time of registration. Queries in relation to the eligibility criteria must be addressed prior to calling/faxing for registration. Patients are eligible for the trial if all the inclusion criteria are met and none of the exclusion criteria applies.

6.4.1. Patient Inclusion Criteria

- Adult patients (18 or over) with suspected primary non-small cell lung cancer on chest CT with sufficient confidence to trigger staging investigations/biopsy OR with already histologically proven primary non-small cell lung cancer
- Disease is potentially radically treatable as defined as stage IIIb or less on diagnostic CT (i.e. T1-4, N0-2, M0)
- Performance status 0-2 (fit to undergo radical treatment if indicated)
- Patient must have given written informed consent and be willing to comply with the protocol intervention and follow up.

For peer review only - http://bmjopen.bmj.com/site/about/guidelines.xhtml

6.4.2. Exclusion Criteria

- Unequivocal metastatic or N3 disease on diagnostic CT chest and abdomen (including M1a disease; malignant pleural effusion)
- Further staging work up not indicated in the opinion of the MDT or clinician due to poor performance status or patient choice.
- Histologies other than non-small cell lung cancer
- Any psychiatric or other disorder likely to impact on informed consent
- Evidence of severe or uncontrolled systemic disease which makes it undesirable for the patient to participate in the trial
- Pregnancy
- Contraindications to MRI (e.g. cardiac pacemaker, severe claustrophobia, inability to lie flat)

6.4.3. Pregnancy and Birth Control

MRI poses a theoretical risk to the foetus, particularly in the first trimester due to local acoustic and heating effects. However the risk is generally deemed very small and significantly less than the risk of ionising radiation imparted by PET-CT and CT (standard imaging investigations).

A woman of childbearing potential (WCBP) is a sexually mature woman (i.e. any female who has ever experienced menstrual bleeding) and who has not undergone a hysterectomy or who has not been postmenopausal for 24 consecutive months (i.e. who has had menses at any time in the preceding 24 consecutive months). Patients will be excluded if they are pregnant.

There is no requirement for additional contraceptive advice to patients over and above that routinely given as part of their routine clinical care given their diagnoses of lung cancer.

The need to perform a pregnancy test in WCBP will be decided as part of the patient's routine clinical care given the risk to pregnancy from standard staging investigations and subsequent treatment. There is no requirement to perform a pregnancy test purely because of recruitment to the trial if this would not have been performed as part of standard clinical care.

For peer review only - http://bmjopen.bmj.com/site/about/guidelines.xhtml

7. Registration Procedures

7.1. Registration

Patient registration will be undertaken centrally at UCL CTC and this must be performed prior to commencement of any trial intervention. Registration can be performed via telephone or fax.

Following pre-registration evaluations (as detailed in section 6.1), confirmation of eligibility and consent of a patient at a site, the registration form must be fully completed prior to faxing or telephoning UCL CTC. The eligibility criteria will be reviewed during the registration telephone call using the same form at UCL CTC.

A trial number will be assigned for the patient during the call and must be recorded at site by the caller. If desired, the site may fax a copy of the completed registration form to UCL CTC and the faxed registration from will be used to confirm patient eligibility at UCL CTC.

UCL CTC will email confirmation of the patient's inclusion in the trial and their trial number to the main contact, PI and Imaging Hub contact. If requested, a fax confirmation may also be sent. Case report forms will be emailed to the main contact at site.

Registration telephone number:	+44 (0)20 7679 9880
Registration fax number:	+44 (0)20 7679 9871
UCL CTC Office hours:	09:00 to 17:00 Monday to Friday

7.2. Registration Packs

Once a patient has consented to take part in the trial they should be provided with a registration pack to include the following:

- A copy of their signed consent form and patient information sheet
- An expenses claim form (to claim travel expenses to and from WB-MRI appointment)
- Pre-paid return envelope

7.3. Booking Whole Body MRI

Once a patient has been registered it is the responsibility of the recruiting site to contact their designated imaging hub to make arrangements for the WB-MRI. Each recruitment site will be provided with a contact number for their designated imaging hub. Recruitment sites will approach consecutive potentially eligible patients until the number of recruited patients fills the MRI capacity of the imaging hub.

For peer review only - http://bmjopen.bmj.com/site/about/guidelines.xhtml

It is possible recruitment potential at recruitment sites will be greater than the WB-MRI capacity at each central imaging hub. If a patient is approached and registered for the trial but MRI capacity is such that the scan could not take place in a timely fashion, a decision to withdraw the patient will be made by the recruitment site and the patient will then be informed. This possibility is explained to patients in the patient information sheet.

Because of the need to perform WB-MRI in a timely fashion, it is anticipated recruitment sites will be requested to pause recruitment if WB-MRI waiting time exceeds 2 weeks (if WB-MRI is performed after conventional imaging is complete) or 3 weeks (if WB-MRI is to be performed concurrently with standard tests). There will be flexibility in these timings depending on the circumstances of individual patients and their anticipated time to complete standard staging.

For peer review only

For peer review only - http://bmjopen.bmj.com/site/about/guidelines.xhtml

8. Patient Management and Trial Procedures

8.1. Summary

8.1.1. Staging and Treatment Decisions

The following information is required for the trial to document staging and treatment decisions:

- Stage and treatment decision based on conventional imaging only (and the number, timing, nature and findings of these investigations)
- Stage and theoretical treatment decision based on WB-MRI (and any additional tests generated by the WB-MRI) only (and the number, timing, nature and findings of additional tests generated)
- A final treatment decision incorporating all available tests.

<u>Conventional Imaging</u>

Trial patients will undergo the conventional staging protocol employed at their institution as per usual clinical care pathways. See section 8.2.1 for further detail.

<u>Whole Body MRI Imaging and MDT Reveal</u>

Recruited patients will undergo a WB-MRI at one of the designated imaging hubs. Recruiting sites should contact their designated imaging hub to request a WB-MRI slot (see section 7.3). The WB-MRI should be performed either concurrently with the standard staging investigations, or no later than 3 weeks after the final standard staging investigation. Images will be uploaded at each hub to a secure central imaging server called 3Dnet™. If a language interpreter was required for a patient during the consent process, then an interpreter should be made available to the patient during the WB-MRI. The WB-MRI will be reported by designated radiologist(s) at each imaging hub who are blinded to the conventional imaging tests and other clinical information (other than the cancer diagnosis and location). A copy of the report will also be uploaded to 3Dnet™. See section 8.2.2 for further detail.

Once the MDT has made a treatment decision based on the conventional imaging the MDT coordinator, research nurse or other designated individual will reveal the WB-MRI images and report on 3Dnet™ via an internet-enabled PC. The MDT will review the WB-MRI results and decide whether or not any additional tests are required or theoretically would have been required (if in fact the test was already performed as part of conventional staging) in addition to the WB-MRI. If any tests are required that have not already been performed as part of the standard staging, they should be requested and the patient reviewed again at the next MDT when the results are available. If no further tests are needed then the stage and theoretical treatment decision based on the WB-MRI (and the results of any actual or theoretical additional tests generated from the WB-MRI) must then be made by the MDT. The MDT must then decide a final treatment decision for the patient based on all imaging available.

If a patient is due to start treatment before the next formal MDT meeting, an ad hoc mini MDT meeting may be scheduled outside of the standard MDT meeting. This should include the relevant multidisciplinary clinical team members who are appropriate

For peer review only - http://bmjopen.bmj.com/site/about/guidelines.xhtml

to make the final treatment decision based on the specific imaging finding, according to usual standards of clinical care. A record will be kept of who is present during the ad hoc mini MDTs on the CRFs.

8.1.2. Patient experience Interviews and Questionnaires (Optional)

Interviews (Study 1)

A total of 25 patients have undergone interviews by the Health Psychologist pertaining to their experience of the cancer staging process. **Target accrual has now been reached for the interview part of the trial and recruitment to this is now closed**.

Questionnaires (Study 2)

Patient experience questionnaires from baseline and post-staging have been received for at approximately 50 patients to assess patient experience and acceptability of WB-MRI and standard tests. **Target accrual has now been reached for the questionnaire part of the trial and recruitment to this is now closed.**

Discrete choice experiment (Study 3)

A discrete choice experiment (DCE) questionnaire will be administered to a subset of approximately 50 patients to elicit their preference for various attributes associated with conventional and WB-MRI staging pathways. The DCE questionnaire is optional and patients will be asked to consent to this on trial entry. Once a patient consents to the DCE they will be allocated a DCE questionnaire (out of a possible 2 DCE questionnaires) which will be posted by UCL CTC along with their Quality of Life questionnaire, patient diary and pre-paid envelope immediately after registration (baseline). UCL CTC will supply the details of any recruited patients who consented to complete DCE questionnaire to the Health psychologists who may contact them directly.

8.1.3. Quality of Life Questionnaires and Patient Diaries

Upon consent the recruiting site must inform the patient that they will receive an EQ-5D Quality of Life Questionnaire and Patient Diary through the post from UCL CTC and they are to complete these and return them using the pre-paid envelope provided. The patient diary will cover a 3 month period and will collect information regarding the patient's Primary Health Care contacts, other health care contacts and also the medication they are currently taking. Patient diaries and EQ-5D questionnaires will be posted to all patients at consent and then at 3 monthly intervals for 12 months.

For peer review only - http://bmjopen.bmj.com/site/about/guidelines.xhtml

Table 1: Summary of patient management and trial activities

Activity	Protocol section	Timing: Prior to registration	Timing: Immediately after registration	Timing: During staging	Timing: After staging (months after registration) 1	3	6	9	Responsible organisation/person
Pre-registration evaluation & assessment of eligibility	6.1	X							Recruiting site
Consent	5.0	X							Recruiting site
Register patient	7.1	X							Recruiting site
Give patient registration pack (copy of PIS and consent, expenses claim form and pre-paid return envelope)	7.2		X						Recruiting site
Book WB-MRI scan & inform patient of appointment date	7.3		X						Recruiting site
WB-MRI	8.2.2			X					Imaging Hub
Upload WB-MRI scan & report	8.2.2			X					Imaging Hub
Post EQ-5D questionnaires	8.2.7		X			X	X	X	UCL CTC
Post patient resource diary	8.2.7		X			X	X	X	UCL CTC
Discrete choice experiment Questionnaire	8.2.10		X						UCL CTC

For peer review only

For peer review only - http://bmjopen.bmj.com/site/about/guidelines.xhtml

8.2. Detailed description

8.2.1. Standard care

Trial patients will undergo the conventional staging investigations employed at their recruiting institution according to local protocols and the requirements of their clinical care team.

All conventional investigations will be performed and interpreted by the usual radiologists and clinicians employed at the site of the investigations. Standard clinical reports will be made and all investigations (and their results) will be freely available on hospital Picture Archiving and Communications System (PACS), Radiology Information System (RIS) and Clinical Data Repository (CDR) systems as per usual clinical practice.

The type and date of investigations (e.g. CT scan, PET-CT, organ specific MRI, biopsy etc.) will be recorded on CRFs, along with the presence and location of metastatic disease based on the radiological report.

8.2.2. Trial Imaging

Whole Body MRI (WB MRI) protocol

Recruited patients will undergo a WB-MRI at their designated imaging hub site. The choice of MRI platform (i.e. manufacturer and Tesla (T) strength) will be decided by the local hub radiologist according to scanner availability and their usual practice. It is anticipated most MRIs will be performed at 1.5T. Exact imaging parameters will vary according to MRI platform but a minimum dataset of sequences will be acquired (full details given in appendix 3).

Timing

The WB-MRI will be performed either concurrently with the standard staging investigations, or no later than 3 weeks after their final standard staging investigation.

Blinding

To ensure the integrity of the trial, the WB-MRI must be reported by a radiologist blinded to the conventional imaging tests and other clinical information (other than the cancer diagnosis and site). Conversely the WB-MRI images and reports must be blinded to radiologists reporting the standard staging investigations, and those involved in direct patient care before the WB-MRI is revealed in the MDT.

The un-anonymised WB-MRI images will therefore not be immediately sent to the Picture Archive and Communication System (PACS) at either the imaging hub or recruitment sites. Instead images will be uploaded to a secure central imaging server (3Dnet™) provided by Biotronics3D. This solution allows easy upload of MRI datasets via standard internet connection. A PC based internet gateway will be installed in each imaging hub to facilitate automated transfer of WB-MRI from the scanner/workstation to 3Dnet™), and thereafter automatically back to PACS to the appropriate time point after MDT revelation (see below).

For peer review only - http://bmjopen.bmj.com/site/about/guidelines.xhtml

Interpretation and reporting

The WB-MRI will be interpreted by designated radiologist(s) at each imaging hub who are expert in interpretation based on previous experience of reporting WB-MRI in cancer staging. As noted above, the radiologist will be blinded to the standard staging investigations performed on recruited patients.

Interpretation can be performed using the 3Dnet™ software, or stand alone workstation according to the preference of the radiologist.

Images will be analysed in the following order:

1) Diffusion and non contrast enhanced T1 images
2) Diffusion, non contrast enhanced T1 and T2 images
3) Diffusion, non contrast enhanced T1, T2 and contrast enhanced T1 images.

After viewing each sequence set (and before reviewing the next set), the radiologist will complete WB-MRI Imaging Booklet CRFs documenting the presence, location and size of metastatic disease, together with their diagnostic confidence on a scale of 1 to 6. Reporting time for each sequence set analysis (defined as the time required to interrogate the sequences to reach a diagnostic conclusion) will be recorded along with the technical quality of the MRI dataset.

The reporting radiologist will then produce a free text clinical report as per their usual clinical practice (using all available sequences and based on the TMN 7 staging guidance) for subsequent release to the clinical team. This report will contain information relating to the local T and N stage of the tumour, together with the presence, location, number and size of metastatic deposits, as well as important “incidental” findings, for example aortic aneurysm. The radiologist may express their level of confidence in reported findings as they would in normal clinical practice but a formal numerical score of confidence will not be provided as this would not mirror how radiological examinations are reported in standard clinical care. If the radiologist would usually recommend additional tests for equivocal findings, this will be included in the report so as to also mirror routine clinical practice. The definitions of T, N M stage will be based on conventional MRI criteria adopted by the radiologist in their usual clinical practice.

The free text report will be uploaded onto the 3Dnet™ software, and a copy stored as part of the patient’s medical record.

For peer review only

8.2.3. Release of Conventional and trial WB-MRI findings

MDT discussion of the first major treatment decision based on conventional imaging

WB-MRI images and reports will be withheld initially from the clinical care team and radiologists reporting standard imaging (to avoid bias) until patients have completed all conventional investigations and have been definitively staged such that a first major treatment decision has been made by the MDT based on these conventional tests. The first major treatment decision based on conventional imaging will be defined as:

- Referral for surgical excision of either the primary tumour and/or a metastatic site

For peer review only - http://bmjopen.bmj.com/site/about/guidelines.xhtml

- Instigation of definitive treatment using chemotherapy, radiotherapy or a combination of the two
- Decision to offer palliative/supportive care only
- Request for a highly invasive surgical staging procedure such as surgical mediastinal lymph node sampling (mediastinoscopy), video-assisted thoracoscopic surgery (VATS), or laparoscopy

If additional conventional tests are required to complete staging, the WB-MRI result will be withheld until they are completed, and the patient re-discussed with the findings of the additional test(s).

The nature, date and findings of all standard investigations will be recorded by the MDT, and the TNM 7 stage recorded. The MDT will define the nature of the first treatment decision based on conventional investigations in the MDT record and on the CRF.

Release of WB-MRI images and report

Once a stage and treatment decision has been made based on conventional imaging, the WB-MRI images (supplemented where possible with "screen shots" and annotations of relevant findings) and radiologist's report will be revealed to the MDT using the 3Dnet™ software on an internet enabled PC. If technical issues preclude use of 3Dnet™ in the MDT, presentation of a written copy of the WB-MRI report is permissible.

When the WB-MRI images and report are released, the MDT will discuss the images and decide:

1) *If the patient can be adequately staged based on WB-MRI alone:*

   In this case the MDT should state the patient's stage and theoretical treatment decision based on the WB-MRI alone.

Or:

2) *If any additional tests are required based on the WB-MRI alone for adequate staging* (for example for equivocal WB-MRI findings):
   a. For additional tests that would have theoretically been requested based on the WB-MRI alone but have already been performed as part of the standard imaging, the results can be used by the MDT. The MDT personnel will state the patient's stage and theoretical treatment decision based on the WB-MRI and those additional tests that would have theoretically been generated.
   b. If the additional tests generated by WB-MRI have not already been performed as part of standard care and are deemed necessary in the opinion of the MDT these will be undertaken according to standard clinical care. After completion of the additional test(s), the patient will be re-discussed by the MDT and a final treatment decision made based on the WB-MRI and the additional test(s) undertaken.

For peer review only - http://bmjopen.bmj.com/site/about/guidelines.xhtml

8.2.4. Final treatment plan

Once a stage and treatment decision has been made based on conventional imaging and also a stage and theoretical treatment decision made based on the WB-MRI alone (and any additional tests) then a final treatment plan can be made by the MDT. The final treatment plan for the patient will be based on all available information including the WB-MRI. The MDT radiologist(s) will be at liberty to review the WB-MRI data after the WB-MRI has been revealed in order to review its findings and feedback to the clinical care team.

The completed MDT CRF will thus list:

- Stage and treatment decision based on conventional investigations (and the number, timing, nature and findings of these investigations).
- Stage and theoretical treatment decision based on WB-MRI (and any additional tests generated by the WB-MRI) alone (and the number, timing, nature and findings of additional tests generated).
- A Final treatment decision incorporating all available tests.

Once the WB-MRI images and report have been revealed to the MDT according to trial protocol, they can be released onto the relevant hospital PACS server using the 3Dnet™ gateway described above or other IT solution (e.g. image exchange portal) and be made freely available to those providing subsequent patient care, as per usual clinical practice.

8.2.5. Early release of WB-MRI findings

Any requests for early WB-MRI release must be made to the site PI, who will discuss the request with the lead clinician who is responsible for the patient's clinical care (if the request did not originate from the lead clinician). After discussion, if it is decided by the PI that release of the WB-MRI images and report is necessary, the PI (or a designated member of the trial team) will inform UCL CTC. The WB-MRI result should only be released early in the event of an emergency situation in which the patient is unfit to undergo additional investigations (including MRI) which would have normally been performed were the patient not in the Streamline L trial, and the WB-MRI may have a direct effect on immediate patient care. In the event of early release, the patient will be replaced, although the findings of the WB-MRI and conventional staging (performed up to the time of early MRI release) will be collated by UCL CTC on the standard trial CRFs for subsequent reporting in the trial publications.

Early release for important clinical findings

The time between the patient's diagnosis and the WB-MRI reveal is typically less than 4 weeks at the proposed recruitment sites. The majority of findings on WB-MRI will have no direct impact on patient care during this time. However should WB-MRI reveal a serious finding which could have an immediate impact on direct patient care before the MDT, the reporting radiologist will contact the patient's clinician to discuss the finding and a decision will be made as to whether the results should be revealed early to all members of the clinical team (based on review of standard tests already performed which may also have detected the finding). Specific findings which will trigger this review are:

- Impending spinal cord compression

For peer review only - http://bmjopen.bmj.com/site/about/guidelines.xhtml

- Deep vein thrombosis or pulmonary embolism
- Brain metastasis with significant mass effect requiring immediate treatment

It is unlikely the patient would be asymptomatic in any of these scenarios, and it is probable additional imaging tests would have already been requested as part of usual clinical care.

Early release for urgent patient management

Although unlikely, it is possible there may be a need to access the WB-MRI report urgently before its release to the MDT, for example if a recruited patient presents to hospital with collapse and knowledge of the WB-MRI findings would potentially change patient management. As noted above, imaging hubs will keep a copy of the free text report of the WB-MRI and this will be made available if requested by clinical teams in this scenario.

8.2.6. Time to full staging

Standard pathway

The total time required to fully stage the patient using standard imaging pathways will be calculated. The start of the staging process will be defined as the date of request of the first staging investigation following a proven or assumed diagnosis of lung cancer (for example date of requested PET/CT after CT or biopsy diagnosis of likely lung tumour). The completion of staging will be defined as the date when the MDT/ appropriate MDT personnel made the first major treatment decision based on the standard imaging.

WB-MRI

Because revelation of the result of WB-MRI is deferred until after standard staging is complete, actual measurement of the time to full staging using WB-MRI will not be possible. The theoretical time to complete staging using WB-MRI will be thus modelled taking into consideration the time from recruitment date to the date of the WB-MRI, plus the number and type of additional staging investigations generated by WB-MRI.

8.2.7. Patient follow up

Patients will be followed for a period of 12 months from the date of recruitment. Follow up will not require any trial specific patient visits to the recruiting site, and follow up CRFs can be completed using hospital databases or patient notes.

For all recruited patients the final TN tumour stage according to the 7th classification will be recorded based on histopathological analysis of surgical specimens (if surgery is performed). The results of any biopsy procedure undertaken over the 12 months will also be recorded. The date, nature and findings of follow up imaging investigations will be recorded for each recruited patient, in particular the presence or absence of metastasis.

The date and cause of patient death and post mortem findings (if performed) will also be recorded.

For peer review only - http://bmjopen.bmj.com/site/about/guidelines.xhtml

Use will be made of MDT records and hospital data repositories to collate this data, and data collection will be coordinated by designated individuals at each recruitment site, aided by UCL CTC.

8.2.8. Patient diaries and EQ-5D Quality of Life questionnaires

All patients will receive a Diary and EQ-5D Quality of Life questionnaire every 3 months which will be posted by UCL CTC. Upon consent the recruiting site must inform the patient that they will receive an EQ-5D questionnaire and Diary through the post from UCL CTC and they are to complete these and return them using the pre-paid envelope provided. The patient diary will cover a 3 month period and will collect information regarding the patient's Primary Health Care contacts, other health care contacts and also the medication they are currently taking. Patient diaries and EQ-5D questionnaires will be posted to all patients at consent and then at 3 monthly intervals for 12 months.

8.2.9. Final reference standard for tumour stage (Consensus Meetings)

Multi-disciplinary consensus panel review is standard methodology for diagnostic test accuracy studies where an independent reference standard is impossible because of incorporation bias. Consensus panels will convene annually to derive the reference standard for tumour stage at diagnosis for recruited patients completing the first 12 month follow up (i.e. a panel at the end of year 2 will consider patients recruited in year 1, a panel at the end of year 3 will consider patient recruited in year 2 etc.). The panels will consider all available clinical information including the results of all original staging investigations, WB-MRI, histopathology (surgical resection and biopsies), follow up imaging and post-mortem reports (where available) and MDT records. UCL CTC will coordinate collation of these data via submitted CRFs over the preceding 12 months for presentation to the panel. Each imaging hub or recruitment site will host a consensus panel to consider patients undergoing WB-MRI (or recruited) at its site (so all imaging studies are available for review on local PACS systems if required). Each panel will consist of at least an oncologist, and/or chest physician, and 3 radiologists, 1 external to the imaging hub and 2 internal: 1 with specific expertise in WB-MRI and 1 with expertise in PET (a single radiologist is acceptable if they have expertise in WB-MRI and PET). The panel will have access to a histopathologist if required. Each panel will adjudicate on the TMN stage of the cancer at diagnosis, including the organ specific sites and burden of metastatic spread.

The definition of the presence or absence of metastasis will be assigned for each organ on a designated CRF. The designation will be made in consensus by all on the panel. Consideration will be made to histology in all biopsied lesions. In the absence of histological proof of metastasis, metastatic disease will be assumed if new lesions appear during the 12 month follow up with imaging characteristics compatible with metastasis and no alternative explanation, or if lesions with characteristics compatible with metastasis which either grow or shrink (on therapy). Lesions identified which remain stable over the 12 month follow up period will assumed not to be metastatic unless there are specific circumstances considered by the panel that indicate malignancy (e.g. change in lesion morphology with treatment).

For peer review only - http://bmjopen.bmj.com/site/about/guidelines.xhtml

All “new” sites of metastatic disease diagnosed in the 12 month follow up will be assessed by the panel to see if they were visible in retrospect at diagnosis. Although the primary analysis by the original reporting radiologist will be that used to define test accuracy, this retrospective review will allow definition of the rates of perceptual error.

For patients in whom the primary tumour has been completely removed within 3 months of diagnosis, all new metastatic sites will be assumed to have been present at diagnosis for the purposes of calculating test sensitivity.

If the primary tumour is left in situ for more than 3 months (or there is incomplete removal), any new diagnosed sites of metastatic disease will be assumed to have been present at diagnosis if identified within 6 months of diagnosis of the primary tumour. If they are diagnosed beyond 6 months of diagnosis, and there is no evidence of their presence on retrospective review of all staging investigations, they will be assumed to be new disease not present at diagnosis.

If patients with tumours left in situ do not undergo any imaging capable of detecting metastatic disease within 6 months of diagnosis of the primary tumour (other than initial staging tests) and imaging beyond this identifies metastatic disease not visible in retrospect on any trial imaging, the consensus panel will opine if the disease was likely present at diagnosis, based on its location, size and imaging characteristics.

If a patient dies before the 12 month follow up, the panel will review all available imaging, histopathology and clinical course prior to death and in consensus state if a confident diagnosis of the presence or absence of metastatic disease can be made (for example imaging characteristics compatible with metastasis and no alternative explanation, or if lesions with characteristics compatible with metastasis which either grow or shrink (on therapy). If this judgement cannot be made with confidence (for example if the patient has equivocal lesions on staging investigations and no further follow up), these patients will not be excluded but multiple imputation will be used to account for missing data. A sensitivity analysis for the primary outcome, where only patients with complete data are analysed, will exclude these patients.

8.2.10. Health psychology assessment

Study 1: Patient interviews (approximate n=25)

Target accrual has now been reached for the patient experience interview part of the trial and so recruitment to this part is now closed. Individual in-depth interviews were conducted by a health psychologist pertaining to their experience of the cancer staging process. The aim of the interviews is to determine patients’ experience of WB-MRI, standard tests and those tests generated by WB-MRI. We collected simple demographic data from participants in relation to gender, age and educational level as such factors may influence experience and preferences, and this data will enable us to determine whether the sample of people who are interviewed are representative of the sample of people who also participate in studies 2 and 3 (see below). The interviews assessed which aspects of testing caused patients physical or psychological stress (e.g. number of tests/hospital visits, test attributes (i.e. physical experiences of the tests such as claustrophobia, need to lie still, scanner noise etc.), total length of time taken, additional tests generated by initial scan findings) and elicited any factors patients felt would have made staging easier for them.

For peer review only - http://bmjopen.bmj.com/site/about/guidelines.xhtml

The interviews provided in-depth qualitative data about the experience of cancer staging and in particular undergoing WB-MRI, and has informed the modification of a questionnaire (study 2) designed to assess patient acceptability and experience of WB-MRI compared to standard tests, and also has informed the design of a discrete choice experiment (study 3).

Study 2: Patient questionnaires (approximate n=50)

Target accrual has now been reached for the patient experience questionnaire part of the trial and so recruitment to this part is now closed. Follow up questionnaires will continue to be posted until the last patient's month 6 time point occurs. A questionnaire was developed and sent to consenting patients to assess their experience and acceptability of WB-MRI and standard tests (e.g. PET-CT). Final design was informed by analysis of the qualitative data collected in study 1 (See above). Patient Experience Questionnaire content is outlined in Table 3 and includes patients' views about test preparation (e.g. fasting), and experience of the scan itself (e.g. discomfort, claustrophobia, fear etc.). Patients were also asked to rate the importance of various attributes associated with competing staging pathways (e.g. waiting time from diagnosis to treatment, total test number, test accuracy etc.). This rating identified those attributes considered most important by patients, which has been included in the discrete choice experiment (study 3).

Patients were given the opportunity to opt in to completing the questionnaires when they were recruited into the trial. Questionnaires, together with the positive and negative affect schedule (PANAS) and the General Health Questionnaire (GHQ-12) were administered during and after staging pathways, and at 3 and 6 months later to examine patient test preferences once they are aware of their cancer stage and knew which of the tests they received were more accurate as well as the burden placed on them by any extra tests they needed as a result of WB-MRI.

Patients were posted the baseline questionnaire along with pre-paid reply envelope at the time they were recruited into the trial. The questionnaire was worded to take into account the fact that some patients had not yet had any staging investigations. Patients were posted the post-staging and 3 and 6 month follow up questionnaires along with pre-paid envelopes at the relevant time points.

Study 3: Discrete choice experiment (approximate n=50)

Discrete choice experiments (DCE) can elicit preferences for different types of health care provision [18] by estimating the relative importance of different attributes, and the trade-offs between them [19]. WB-MRI and conventional staging pathways differ in associated attributes, not only related to physical experience but also rate of adverse events, time to diagnosis, overall accuracy etc. The most important attributes have been identified in studies 1 and 2 and appropriate levels assigned to each based on accumulating data from the trial, together with appropriate literature review. A DCE questionnaire has been developed whereby patients state their preference between two choices, with each choice containing different levels of the identified attributes.

The DCE questionnaire is optional and patients will be asked to consent to complete the questionnaire on trial entry. Once a patient consents to the DCE they will be allocated a DCE questionnaire (out of a possible 2 DCE questionnaires) which will be posted by UCL CTC immediately after registration (baseline) along with their QoL

For peer review only - http://bmjopen.bmj.com/site/about/guidelines.xhtml

questionnaire, patient diary and pre-paid envelope. UCL CTC will supply the details of any recruited patients who consented to complete the DCE questionnaire to the Health psychologists who may contact them directly.

Table 2: Questionnaire schedule.

Questionnaires will be posted to patients by UCL CTC.

	Baseline (peri-staging)	Months after registration		
		1	3	6
Demographic measures	✓			
Co-morbidities	✓			
Self-rated health	✓			
Symptoms of lung cancer	✓			
Concern for future consequences	✓			
Which staging tests have had	✓	✓		
Perceived importance of test attributes	✓	✓		
Perceived acceptability of different tests	✓	✓		
Least acceptable aspects of different tests	✓	✓		
Agreement to having tests again (short and long-term)	✓	✓		
Single test choice	✓	✓		
Test recovery time	✓	✓		
Patient experience of WB-MRI and standard tests	✓	✓		
Perceived efficacy of WB-MRI and standard tests		✓		
Difficulties with completing scan (WB-MRI and standard tests)		✓		
Ranked attribute preferences	✓	✓		
Feelings about number of tests			✓	✓
EQ-5D	✓		✓	✓
PANAS	✓	✓	✓	✓
GHQ-12	✓	✓	✓	✓

For peer review only - http://bmjopen.bmj.com/site/about/guidelines.xhtml

8.2.11. Cost Effectiveness Assessment

Overview

We will undertake a detailed analysis of the cost and the cost-effectiveness of WB-MRI versus standard NICE-approved staging algorithms. The analysis will conform to accepted economic evaluation methods [15]. All costs will be assessed from the perspective of the NHS and personal social services (PSS).

Making comparisons between WB-MRI versus standard staging algorithms

The care pathway for lung cancer patients can both be divided into two stages, the *treatment decision pathway* and the *subsequent disease pathway*. The former includes the time from diagnosis to treatment decision by the MDT; the latter includes the time period following the treatment decision.

The treatment decision pathway will be different between WB-MRI and standard staging algorithms, yielding different costs and potentially different treatment decisions. In patients for whom the treatment decision with WB-MRI is the same as that with conventional staging algorithms, the subsequent disease pathways will be the same. Where the treatment decision with WB-MRI is different, the disease pathway will be different, yielding potentially different costs and health outcomes.

If in the patients studied the concordance between the treatment decisions associated with WB-MRI and conventional staging algorithms is high, then the economic analysis can focus on the cost of the treatment decision pathways only because the disease pathways will be no different. In this case the cost-effectiveness of WB-MRI versus conventional staging algorithms depends only on the incremental cost (positive or negative) of WB-MRI versus standard staging algorithms in the treatment decision pathway.

Conversely, if the concordance between the treatment decisions is low, then the economic analysis ought to focus on both the treatment decision pathways and the subsequent disease pathways because both of these will vary between WB-MRI and conventional staging algorithms. In this case the cost-effectiveness of WB-MRI depends on the incremental cost of the WB-MRI versus standard staging algorithms in the treatment decision pathway plus the incremental costs and health benefits of the disease pathway.

The precise nature of the economic analysis will therefore depend on the degree of concordance between treatment decisions provoked by WB-MRI versus conventional staging.

Discordance of major treatment decisions

Discordance will be defined when the first major treatment decision differs between WB-MRI and standard investigations in greater than 10% of patients. Concordance will be defined as the absence of discordance. For lung cancer we define a major difference in treatment decision as occurring when only one pathway suggests the patient is a surgical candidate or suitable for radical radiotherapy. A 3x2 table will be constructed according to decision to perform surgery, use radical radiotherapy or not based on WB-MRI vs. standard diagnostic pathways.

For peer review only - http://bmjopen.bmj.com/site/about/guidelines.xhtml

Scenario 1: there is concordance between the treatment decisions associated with WB-MRI and standard staging

In this case, the cost components included in the analysis will be:

- Conventional tests for staging of disease among diagnosed patients (e.g., PET/CT/EBUS/TBNA/USS neck for lung cancer, plus additional tests as indicated by the conventional staging algorithm);
- Costs of treating adverse events associated with staging tests;
- WB-MRI, plus additional tests generated by WB-MRI;
- MDT meetings.

The volume of resource use for each cost component will be measured directly in the trial from treatment decisions recorded in MDT reports, based first on conventional staging alone and then based on WB-MRI alone, and on patient records included in the trial. Unit costs will be taken from standard published sources. Since the two algorithms yield the same treatment decisions cost-effectiveness depends on the incremental cost of WB-MRI versus conventional staging algorithms in the treatment decision pathway (i.e., formally this is a cost-minimisation analysis).

Scenario 2: there is discordance between the treatment decisions associated with WB-MRI and standard staging

In this case, cost-effectiveness depends on the incremental cost (positive or negative) of the treatment decision pathway and disease pathway associated with WB-MRI versus conventional staging and the incremental health benefits (positive or negative). We will calculate cost-effectiveness in terms of the incremental cost per quality-adjusted life year (QALY) gained using one year and lifetime time horizons.

For the analysis based on the one-year time horizon, the trial will provide information on the treatment decisions arising from WB-MRI and standard staging and follow up data for the first year. We will collect these data from three sources.

Firstly for all patients in the trial we will collect the resource use data for the main drivers of hospital costs using a trial specific CRF. This will collect resource use data on the following cost components for each patient:

- Imaging investigations
- Chemotherapy
- Radiotherapy
- Surgery and biopsies
- Outpatient visits
- Inpatient stays
- Day cases

These data will be recorded prospectively based mainly on MDT consensus meeting recommendations and on patient records (for major changes in treatment). Unit costs will be taken from standard published sources and applied to the resource use data, allowing us to cost the care received by each patient.

For peer review only - http://bmjopen.bmj.com/site/about/guidelines.xhtml

In addition, from this source we will collect data on whether or not the patient died during the follow up period, and if they died, the date of death.

Secondly for all patients in the trial we will prospectively collect resource use data using patient diaries. This will supplement the above data which may not provide a complete picture of hospital resource use, plus it will allow us to collect data on primary and community care contacts. These will record resource use data on the following cost components for each patient:

- Medications taken
- Day cases
- GP contacts
- Practice and community nurse contacts
- A&E Visits
- Any other primary care or community care contacts related to cancer

The diaries will be posted out by UCL CTC at baseline, 3, 6, and 9 months and patients will be asked to complete them for the following 3 month period.

In addition we will also collect data on health-related quality of life score, measured according to the EQ-5D (www.euroqol.org) which will be measured at baseline and at 3, 6, 9 and 12 months for all surviving patients.

UCL CTC will contact sites just prior to sending out any post registration diaries or questionnaires to check the status of the patient to ensure none are sent inappropriately to deceased patients.

Patients who are found not to have cancer or are withdrawn for other reasons (see section 13), will not be sent diaries, quality of life or patient experience questionnaires to complete.

Thirdly the two sources of resource use data described above will be supplemented with a review of resource data use collected in the recently completed Lung-BOOST trial. This may be supplemented by retrospective review of resource use (including imaging, outpatient visits, inpatients stays and medication use) for the 12 months post diagnosis of up to 150 patients diagnosed with lung cancer at UCLH. This retrospective review will be carried out by a member of the clinical team and according to guidance provided by the UCLH Caldicott guardian. All collated retrospective data will be fully anonymised before sharing with other trial researchers outside the clinical care team.

8.2.12. Nested substudies

1) Diagnostic performance of limited WB-MRI protocols based on diffusion and T1 weighted imaging only, and to assess the incremental benefit of intravenous gadolinium contrast enhancement.

   As noted above, WB-MRI will be analysed using a locked sequential viewing paradigm such that the diagnostic accuracy of Diffusion and non contrast enhanced T1 images, Diffusion, non contrast enhanced T1 and T2 images, and Diffusion, non contrast enhanced T1, T2 and contrast enhanced T1 images can be calculated.

For peer review only - http://bmjopen.bmj.com/site/about/guidelines.xhtml

2) Effect of radiologists' diagnostic confidence on the accuracy of WB-MRI.

Radiologists will indicate their level of confidence for the presence or absence of metastasis on a 1-6 scale on the CRF recording their interpretation of the WB-MRI.

3) Inter-observer variation in WB-MRI interpretation.

Each hub radiologist (n=6) will interpret a sample of 25 WB-MRI datasets selected at random from the other imaging hubs to define inter-observer variation in the reported presence absence of metastatic disease.

4) The collated WB-MRI datasets and 12 month patient follow up data collated as part of the trial protocol provides opportunities to retrospectively mine the data for additional diagnostic indices and prognostic markers (See appendix 4). The following or similar may be quantified in all or a subset of whole body MRI datasets:

- Body fat (organ, visceral and whole body)
- MRI signal from the primary tumour and metastatic sites, notably diffusion weighted signal, apparent diffusion coefficient, T1 (including fat and water components), T2 signal, Contrast enhancement, and textural analysis (for heterogeneity) of the primary and metastatic deposits on the various sequences.

For peer review only - http://bmjopen.bmj.com/site/about/guidelines.xhtml

9. Data Management Guidelines

Data will be collected from sites on version controlled case report forms (CRFs) designed for the trial and supplied by UCL CTC. Data entered onto CRFs must reflect source data at site. Some data will be recorded directly on the CRFs (i.e. no prior written or electronic record of data), and it will be considered to be source data. This will be made clear in the CRF guidance.

Where supporting documentation (e.g. autopsy reports, pathology reports, CT scan images etc.) is being submitted to UCL CTC, the patient's trial number must be clearly indicated on all material and any patient identifiers removed/blacked out prior to sending to maintain confidentiality.

9.1. Completing Case Report Forms

All CRFs must be completed and signed by staff who are listed on the site staff delegation log and authorised by the PI to perform this duty. The PI is responsible for the accuracy of all data reported in the CRF.

All entries must be clear, legible and written in ball point pen. The use of abbreviations and acronyms must be avoided.

Any corrections made to a CRF at site must be made by drawing a single line through the incorrect item ensuring that the previous entry is not obscured. Each correction must be dated and initialled. Correction fluid must not be used.

The use of abbreviations and acronyms must be avoided.

Once completed the original CRFs must be sent to UCL CTC and a copy kept at site.

9.2. Missing Data

To avoid the need for unnecessary data queries CRFs must be checked at site to ensure there are no blank fields before sending to UCL CTC. When data are unavailable because a measure has not been taken or test not performed, enter "ND" for not done. If an item was not required at the particular time the form relates to, enter "NA" for not applicable. When data are unknown enter the value "NK" (only use if every effort has been made to obtain the data).

9.3. Timelines for data return

CRFs must be completed at site and returned to UCL CTC as soon as possible and within 4 weeks of scheduled time point. For further details, see CRF instructions.

Sites who persistently do not return data within the required timelines may be suspended from recruiting further patients into the trial by UCL CTC and subjected to a 'for cause' monitoring visit. See section 12.2 ('For cause' on-site monitoring) for details.

For peer review only - http://bmjopen.bmj.com/site/about/guidelines.xhtml

9.4. Data Queries

Data arriving at UCL CTC will be checked for legibility, completeness, accuracy and consistency, including checks for missing or unusual values. Query reports will be sent to the data contact at site. Further guidance on how data contacts should respond to Data Queries can be found on the Query Reports.

For peer review only

For peer review only - http://bmjopen.bmj.com/site/about/guidelines.xhtml

10. Safety Reporting

Clinical Review

The MRI Imaging Hub and/or Recruiting site will record any complications attributable to the WB-MRI or any additional test performed as a result of the WB-MRI, notably contrast reactions, biopsy complications (infection, bleeding or hospital admission), and treatment target breaches on the Staging Complications CRF which will be submitted to UCL CTC.

UCL CTC will provide trial data to the CI on a periodic basis for review. If it is declared necessary to revise the conduct of the trial due to safety concerns, UCL CTC will inform the REC as appropriate.

For peer review only

For peer review only - http://bmjopen.bmj.com/site/about/guidelines.xhtml

11. Incident Reporting

Organisations must notify UCL CTC of all deviations from the protocol or GCP immediately. UCL CTC may require a report on the incident(s) and a form will be provided if the organisation does not have an appropriate document (e.g. Trust Incident Form).

If site staff are unsure whether a certain occurrence constitutes a deviation from the protocol or GCP, the UCL CTC trial team can be contacted immediately to discuss.

UCL CTC will use an organisation's history of non-compliance to make decisions on future collaborations.

For peer review only

For peer review only - http://bmjopen.bmj.com/site/about/guidelines.xhtml

12. Trial Monitoring and Oversight

Participating sites and PIs must agree to allow trial-related on site monitoring, Sponsor audits and regulatory inspections by providing direct access to source data/documents as required. Patients are informed of this in the patient information sheet and are asked to consent to their medical notes being reviewed by appropriate individuals on the consent form.

UCL CTC will determine the appropriate level and nature of monitoring required for the trial. Risk will be assessed on an ongoing basis and adjustments made accordingly.

12.1. Central Monitoring

Sites will be requested to submit relevant logs to UCL CTC at the frequency detailed in the trial monitoring plan or on request and these will be checked for consistency and completeness. Also refer to sections 4.2.2 (Required Documentation) and 6.2 (Screening Logs).

Ensuring patient eligibility is the responsibility of the PI or other delegated Investigator(s). Checks of the criteria listed on the registration form will be undertaken by an appropriately trained UCL CTC staff member prior to registration. Also refer to section 7.1 (Registration).

Details relating to the informed consent process will be collected on the registration form and are subject to review by UCL CTC as part of patient eligibility.

Data received at UCL CTC will be subject to review in accordance with section 9.4 (Data Queries).

Where central monitoring of data and/or documentation submitted by sites indicates that a patient may have been placed at risk, the matter will be raised urgently with site staff and escalated as appropriate (refer to section 11 (Incident Reporting) and 12.2 ('For cause' on site monitoring) for further details).

12.2. 'For Cause' On Site Monitoring

On site monitoring visits may be scheduled at a site where there is evidence or suspicion of non-compliance with important aspect(s) of the trial protocol/GCP requirements. Sites will be sent a letter in advance outlining the reason(s) for the visit. The letter will include a list of the documents that are to be reviewed, interviews that will be conducted, planned inspections of the facilities, who will be performing the visit and when the visit is likely to occur.

Following a monitoring visit, the Trial Monitor/Trial Coordinator will provide a report to the site, which will summarise the documents reviewed and a statement of findings, deviations, deficiencies, conclusions, actions taken and actions required. The PI at each site will be responsible for ensuring that monitoring findings are addressed in a timely manner, and by the deadline specified.

For peer review only - http://bmjopen.bmj.com/site/about/guidelines.xhtml

UCL CTC will assess whether it is appropriate for the site to continue participation in the trial. Refer to section 11 (Incident Reporting) for details.

12.3. Oversight Committees

12.3.1. Trial Management Group (TMG)

The TMG will include the Chief Investigator, clinicians and experts from relevant specialities and Streamline L trial staff from UCL CTC (see page 3). The TMG will be responsible for overseeing the trial. The group will meet regularly at least once a year and will send updates to PIs (via newsletters or at Investigator meetings) and to the NCRI Lung Clinical Studies Group.

The TMG will review substantial amendments to the protocol prior to submission to the REC. All PIs will be kept informed of substantial amendments through their nominated responsible individuals.

TMG members must sign a charter confirming acceptance of their responsibilities.

12.3.2. Trial Steering Committee (TSC)

The role of the TSC is to provide overall supervision of the trial. The TSC will review the recommendations of the Independent Data Monitoring Committee and, on consideration of this information, recommend any appropriate amendments/actions for the trial as necessary. The TSC acts on behalf of the funder and Sponsor.

TSC members must sign a charter confirming acceptance of their responsibilities and declaring any potential conflicts of interest.

12.3.3. Independent Data Monitoring Committee (IDMC)

The role of the IDMC is to provide independent advice on data and safety aspects of the trial. Meetings of the Committee will be held at least once a year or as necessary to address any issues. The IDMC is advisory to the TSC and can recommend premature closure of the trial to the TSC.

IDMC members must sign a charter confirming acceptance of their responsibilities and declaring any potential conflicts of interest.

12.3.4. Role of UCL CTC

UCL CTC will be responsible for the day to day coordination and management of the trial and will act as custodian of the data generated in the trial (on behalf of UCL). UCL CTC is responsible for all duties relating to safety reporting which are conducted in accordance with section 10 (Safety Reporting).

For peer review only - http://bmjopen.bmj.com/site/about/guidelines.xhtml

13. Withdrawal of Patients

In consenting to the trial, patients are consenting to the WB-MRI, and also specifically consenting to completing questionnaires and diaries.

Losses to follow up

If a patient moves from the area, the site should make every effort to ensure the patient is followed up at another participating trial site and for this new site to take over the responsibility for the patient, or for follow up via GP. Details of participating trial sites can be obtained from the UCL CTC trial team who must be informed of the transfer of care and follow up arrangements.

Patients should be considered lost to follow up only once documented efforts on the part of the site have failed to produce any response or information from the patient or GP over the course of one year.

If a patient is lost to follow up at a site every effort should be made to contact the patient's GP to obtain information on the patient's status. If the patient cannot be contacted this should be recorded on the Change of Status CRF.

Patients Withdrawing Consent

If a patient expresses their wish to withdraw from the trial, sites should explain the importance of allowing routine follow up data to be used for trial purposes and for allowing existing collected data to be used. If the patient gives a reason for their withdrawal, this should be recorded (see below).

Withdrawal of Consent to Future Data Collection and use of past data

If a patient explicitly states they do not wish to contribute further data to the trial or allow the use of past data, their decision must be respected and recorded on the Change of Status CRF. In this event details should be recorded in the patient's hospital records, no further CRFs must be completed and no further data sent to UCL CTC.

Withdrawal of Consent to QoL, Resource use diary and/or Patient experience questionnaires

If a patient withdraws consent from completing the QoL, Resource use diary and/or the Patient experience questionnaires their decision must be recorded on the Change of Status CRF. UCL CTC will not post any further documents to the patient. However, the patient may continue to participate in the trial, and trial follow up completed as per protocol.

For peer review only - http://bmjopen.bmj.com/site/about/guidelines.xhtml

14. Trial Closure

14.1. End of Trial

For regulatory purposes the end of the trial will be 1 year after recruitment of the final patient at which point the 'declaration of end of trial' form will be submitted to ethical committees, as required.

Following this, UCL CTC will advise sites on the procedure for closing the trial at the site.

14.2. Archiving of Trial Documentation

At the end of the trial, UCL CTC will archive securely all centrally held trial related documentation for a minimum of 5 years. Arrangements for confidential destruction will then be made. It is the responsibility of PIs to ensure data and all essential documents relating to the trial held at site are retained for a minimum of 5 years after the end of the trial, in accordance with national legislation and for the maximum period of time permitted by the site.

Essential documents are those which enable both the conduct of the trial and the quality of the data produced to be evaluated and show whether the site complied with the principles of GCP and all applicable regulatory requirements.

UCL CTC will notify sites when trial documentation held at sites may be archived. All archived documents must continue to be available for inspection by appropriate authorities upon request.

Health Psychology data collected as part of this research will be retained for 10 years in line with Birkbeck, University of London's records management policy. At the end of this time period the documents will be shredded. The recordings of the interviews will be destroyed after they have been transcribed and verified.

14.3. Early discontinuation of trial

The trial may be stopped before completion as an Urgent Safety Measure on the recommendation of the TSC or IDMC (see section 12.3.2 TSC and 12.3.3 IDMC). Sites will be informed in writing by UCL CTC of reasons for early closure and the actions to be taken with regards the treatment and follow up of patients.

14.4. Withdrawal from trial participation by a site

Should a site choose to close to recruitment the PI must inform UCL CTC in writing. Follow up as per protocol must continue for all patients recruited into the trial at that site and other responsibilities continue as per Clinical Trial Site Agreement (CTSA).

For peer review only - http://bmjopen.bmj.com/site/about/guidelines.xhtml

15. Quality Assurance

The technical quality of at least 10% the WB-MRIs from each imaging hub will be assessed by the trial radiographer at UCLH. Using the 3Dnet™ software, the radiographer will access performed scans on a weekly basis and enter an audit score of quality according to the definitions below. A report of the quality will be provided to the CI every 2 weeks and to the TMG at each meeting.

Technical quality – general

1 = More than one sequence with substantial degradation of images severely limiting interpretation of those sequences, and not repeated

2 = One sequence with substantial degradation of images severely limiting interpretation of that sequence, and not repeated

3 = More than one sequence has minor artefact, but all remain fully diagnostic and repeat although optimal, not necessary OR all sequences initially technically inadequate (score 1 or 2) correctly repeated

4 = One sequence a has minor artefact, but remains fully diagnostic and repeat, although optimal, not necessary

5 = All sequences technically optimal with no artefact or degradation

Technical quality – anatomical coverage

1 = Wrong examination performed

2 = More than one sequence does not adequately cover the body (skull to mid thigh), or designated organ(s) coverage

3 = One sequence does not optimally cover the body or designated organ(s) coverage but examination remains fully diagnostic

4 = All sequences optimally cover the body and designated organ(s) coverage

For peer review only - http://bmjopen.bmj.com/site/about/guidelines.xhtml

16. Statistics

16.1. Sample size calculation

SAMPLE SIZE: Studies of diagnostic test accuracy are based on a cohort design where all patients receive all main diagnostic tests (PET/CT or WB-MRI), although additional tests ordered in each staging pathway differ between patients, based on clinical appropriateness. Existing pathways and therapeutic decision making is complex for lung cancers (see flow chart). This NIHR HTA trial focuses on first definitive staging and we are powered at this point in the patient pathway, informed by a detailed literature review.

Multiple therapeutic options are contingent on diagnosis of metastatic disease.

We therefore power on a change in sensitivity for metastases detection with a WB-MRI pathway as a replacement to the standard NICE staging pathway.

The premise of the trial power is to detect a higher sensitivity for extra thoracic disease (specifically brain and bone metastasis) with WB-MRI in comparison to standard imaging paradigms. To power on equivalence or non-inferiority would be well beyond pragmatic recruitment and funding.

Based on recent meta-analysis data [10] considering 1874 patients, the overall prevalence of brain metastasis at presentation in NSCLC is 13% (6% to 32%) and bone 20% (8-34%). Around 20% have metastasis at multiple sites [1, 4, 17, 20].

Prevalence assumptions

Conservative estimate of the prevalence of brain metastasis at presentation is 10%.

20% of these patients are already identified as having metastatic disease via detection of co-existing extra cranial disease on pre-diagnosis CT (e.g. liver, adrenal).

A further 10% of patients will have disease at more than one site on CT (and therefore more likely detected by PET CT than if isolated to the brain).

Overall 12% (n=40) of the hypothetical 325 patients with potentially operable disease will have isolated brain metastasis and 5% (n=16) have bone metastasis. The remaining 8% (of the total 25% with undiagnosed extra-thoracic metastatic disease) will have metastasis at other organ sites (e.g. lung, liver adrenal).

Sample size calculation

Comparison of WB-MRI to PET & PET/CT accuracy - both against reference standard

- Paired trial design – comparing two diagnostic pathways where both are used in all patients.
- Sample size method for difference in paired proportions [21].
- Power trial to show difference in sensitivity, as fewer patients are with metastasis than without. Trials powered like this should also be suitable for difference in specificity.
- 80% power type II error, type I error 5% ($p<0.05$).

For peer review only - http://bmjopen.bmj.com/site/about/guidelines.xhtml

Streamline L

- Assume ratio of marginal cells of 2 x 2 table comparing WB-MRI to PET: s/t = 9 to 11 (tables 3 and 4)
- Assume sum of marginal cells as proportion of total with disease: s+t/N=0.24
- A sample size of 50 patients with occult metastasis is needed, from a population of 25% prevalence of metastasis. Thus a total sample size of 200 (50 times 4) is required.

200 patients with no metastasis at lung cancer diagnosis and potential surgical candidates would be required to detect a difference of 24% in sensitivity of WB-MRI for metastatic disease (79%) compared to conventional staging (55%) given site specific disease prevalence as described above.

The sample size above must be adjusted to account for “withdrawals”. Patients are classified as withdrawn if they are recruited but are not evaluable for any of the following reasons (i) they do not have an WB-MRI scan, the main component of the primary outcome (ii) they do not have cancer (iii) they are lost to follow up before 1 year and do not have sufficient information for the expert panel to evaluate whether metastases were present.

The withdrawal rate was expected to be 20%, giving a target recruitment of 250 patients. However, the actual withdrawal rate has been 43% and the target recruitment has therefore been revised to 353 patients.

Table 3: Calculations comparing WB-MRI to PET

		PET & PET/CT		
	Result	+	-	**Total**
**WB-MRI**	+	26	11	37
	-	1	12	13
	**Total**	27	23	**50**

Table 4: Calculations broken down by tumour site

		PET & PET/CT		
	Result	+	-	**Total**
**WB-MRI**	+	brain-7<br>bone-7<br>other 12	brain-10<br>bone-1<br>other 0	37
	-	brain-0<br>bone-0<br>other-1	brain-8<br>bone-1<br>other-3	13
	**Total**	27	23	**50**

16.2. Population for analysis

The analysis population will be all patients, equivalent to the RCT population intention to treat (ITT) population, with multiple imputation used to impute missing data. For the primary outcome, complete case analysis (all available patient data with no imputation) will be reported alongside as a sensitivity analysis to the ITT analysis.

For peer review only - http://bmjopen.bmj.com/site/about/guidelines.xhtml

16.3. Analysis of the primary endpoint

Primary Outcome- Per patient sensitivity for metastasis detection by whole body MRI (WB-MRI) compared to standard staging pathways in newly diagnosed non-small cell lung cancer against an expert derived consensus reference standard.

The primary objective is to evaluate whether early whole body magnetic resonance Imaging (WB-MRI) increases per patient sensitivity for metastasis in non-small cell lung cancer compared to standard NICE-approved diagnostic pathways.

- Comparison to detect metastases between early WB-MRI alone and standard NICE diagnostic strategy.
- Difference in per patient sensitivity.
- Paired comparison of proportions of each strategy against the reference standard of full clinical diagnosis and 12 month follow up. [22]
- Imputation will be used to account for missing data and imperfect reference data. [23]

Definition of positive test result from standard tests (conventional imaging)

- Conventional imaging tests include CT and may include PET, USS, organ specific MRI, bone scans, lymph node sampling.
- Report from the MDT will express presence of metastatic disease for each patient categorised as yes, no and equivocal.
- Equivocal results will be grouped with positive test results as these results require additional follow up investigations compared to negative results.
- The radiology imaging report supplied to the MDT will be used for sensitivity analysis using free text report (possible and probable metastasis counted as positive).

Definition of positive test result from WB-MRI and additional tests requested after WB-MRI (WB-MRI as replacement test)

- WB-MRI imaging CRF report from the WB-MRI radiologist will express presence of metastatic disease for each patient categorised as yes, no and equivocal.
- Equivocal results will be grouped with positive test results as these results require additional follow up investigations compared to negative results.

Definition of positive test result from reference test: 12 month expert consensus panel

Expert panel results will be classified as positive or negative for metastasis by expert consensus. No equivocal category will be allowed.

Multiple imputation will be used for missing data using chained equations in STATA [24].

Sensitivity analysis: A sensitivity analysis will investigate the impact of the equivocal results on the detection rate by WB-MRI. Equivocal results will be grouped with negative test results [25].

For peer review only - http://bmjopen.bmj.com/site/about/guidelines.xhtml

16.4. Analysis of secondary endpoints

16.4.1. Efficacy (secondary)

Secondary outcome- The time and test number taken to reach, and the nature of, the first major treatment decision based on WB-MRI in comparison to standard staging pathways.

The objective is to determine how WB-MRI influences time to and nature of first major treatment decision following definitive staging compared to standard investigations and to determine whether early WB-MRI could reduce or replace standard investigations.

For this secondary outcome, the following components will be measured up to the time of the first major treatment decision

- Nature of first major treatment decision and number of patients with changed management decision
- Time taken for diagnostic pathways
- Number of tests: average per patient, number of patients with fewer tests in pathway

These will be compared for WB-MRI pathway in comparison to standard staging pathways.

This information will be reported and discussed as a basis for whether early WB-MRI could replace or reduce standard investigations.

No absolute pre-specified definition of the combination of these outcomes likely to be considered clinically significant is attempted although it is noted that:

- An average decrease in diagnosis time of 7 days is likely to be considered clinically significant if accuracy were similar.

Secondary outcome- Diagnosis accuracy of WB-MRI and conventional staging pathways for local tumour staging and detection of metastasis in comparison to an expert derived consensus reference standard.

The objective is to assess the accuracy of WB-MRI and standard diagnostic pathways for local and distant staging by comparison with an expert derived consensus reference standard using 1 year patient follow up data.

- All comparisons except difference in sensitivity per patient compare WB-MRI as both replacement and additional test compared to standard NICE staging pathway

The following outcomes will be assessed for this secondary outcome:

- Difference in sensitivity per patient: WB-MRI as additional test compared to standard NICE staging pathway
- Difference in sensitivity and specificity per organ site. Count each site once per patient, regardless of the number of metastases per organ.
- Difference in sensitivity and specificity per metastasis.

For peer review only - http://bmjopen.bmj.com/site/about/guidelines.xhtml

Secondary outcome- Inter-observer variability in WB-MRI analysis and effect of diagnostic confidence on staging accuracy.

The objective is to determine the effect of radiologist confidence on the diagnostic accuracy of WB-MRI for metastatic disease.

A substudy will be conducted to look at inter-observer variability between radiologists interpreting WB-MRI images and the effect of diagnostic confidence on staging accuracy.

- Each hub radiologist (n=6) will interpret a sample of 25 WB-MRI datasets selected at random from the other imaging hubs to define inter-observer variation in the reported presence absence of metastatic disease.
- These images will be read after patient management decisions are taken and so will not affect patient diagnosis or treatment.

Secondary outcome – Diagnostic accuracy of limited T1 and diffusion weighted sequences compared to full multi-sequence WB-MRI protocols.

To evaluate the diagnostic performance of limited WB-MRI protocols based on diffusion and T1 weighted imaging only, and to assess the incremental benefit of intravenous gadolinium contrast enhancement.

Comparison of diagnostic accuracy of WB-MRI alone (i) block 1 sequence (ii) block 2 (iii) block 3 sequences.

- Reference standard: WB-MRI plus standard tests with 12 month follow up.
- Methods and tables: as per primary outcome and secondary outcome #3.
- Analysed for units of analysis as (i) per patient (ii) per organ site (iii) per metastasis.

16.4.2. Safety

We will provide information on the following:

- Additional tests ordered as a result of WB-MRI but not ordered for the patient in the standard NICE staging pathway. Note this might include tests due to abnormalities outside lung.
- Extra days in hospital due to extra tests ordered through WB-MRI.

16.4.3. Economic evaluation

Patient-specific utility profiles will then be constructed assuming a straight line relation between each of the patients EQ-5D scores at each follow up point. The quality-adjusted life-years (QALYs) experienced by each patient up to one year will be calculated as the area underneath this profile.

We will calculate for each individual patient in the trial their costs and outcomes up to one year. We will also have data from the Lung-BOOST trial and retrospective data collection for patients previously diagnosed with lung cancer at UCLH on the cost of similar patients up to one year.

For peer review only - http://bmjopen.bmj.com/site/about/guidelines.xhtml

Individual patients will then be grouped according to the specific disease path depending on the treatment decision (e.g., surgery, radical radiotherapy or chemotherapy/non radical radiotherapy for lung cancer) and the accuracy of the staging result. We will calculate the mean costs and QALYs for each group. Mean costs and QALYs for WB-MRI versus standard staging algorithms will then be calculated based on the proportion in each group using each algorithm, which will be different since in this scenario there is discordance in treatment decision.

Cost-effectiveness will be calculated as the mean cost difference between WB-MRI versus standard staging algorithms divided by the mean difference in outcomes (QALYs) to give the incremental cost-effectiveness ratio (ICER). Non-parametric methods for calculating confidence intervals around the ICER based on bootstrapped estimates of the mean cost and QALY differences will be used [26]. The bootstrap replications will also be used to construct a cost-effectiveness acceptability curve, which will show the probability that WB-MRI is cost-effective at one year for different values of the NHS' willingness to pay for an additional QALY. We will also subject the results to extensive deterministic (one-, two- and multi-way) sensitivity analysis.

For the analysis based on the lifetime time horizon we will use the 1 year data described above. To extrapolate beyond the end of the 1 year follow up period we will develop a de novo cost-effectiveness model for the disease pathway, which will be populated via available evidence. These will model patient movements between long-term health states. The models will be similar in design to a recent NIHR HTA-funded trial of PET and MRI for detection of metastasis in breast cancer [27]. Following decisions about model structure, a list of parameter estimates required for the model will be developed. The specific details of the data to be used to populate the model will be determined following the development of the structure and the systematic searches of the literature to identify existing models.

The upshot of this analysis is that we will calculate for each individual patient in the trial their lifetime costs and QALYs. Individual patients will then be grouped according to the specific disease path and the accuracy of the staging result, as before, and we will calculate the mean costs and QALYs for each group. Mean costs and QALYs for WB-MRI versus standard staging algorithms will then be calculated based on the proportion in each group using each algorithm, again as before. We will undertake deterministic (one-, two- and multi-way) and probabilistic sensitivity analysis, the latter assuming appropriate distributions and parameter values [28].

16.4.4. Health psychology assessment

Study 1: Patient interviews (approximate n=25)

No formal statistical analysis will be carried out on the information from the patient interviews. The interviews will provide in-depth qualitative data about the experience of cancer staging and in particular undergoing WB-MRI, and will inform the modification of a questionnaire (study 2) designed to assess patient acceptability and experience of WB-MRI compared to standard tests, and also inform the design of a discrete choice experiment (study 3).

For peer review only - http://bmjopen.bmj.com/site/about/guidelines.xhtml

Study 2: Patient questionnaires (approximate n=50)

Comparative patient experience between WB-MRI and standard staging investigations, identification of important staging pathway attributes, comparative anxiety, expectations and attribute importance before and following the staging process will be analysed using within subjects ANOVA or the Wilcoxon matched-pair sign-test depending on whether the data are normally distributed or not.

Study 3: Discrete choice experiment (approximate n=50)

Sample size should be greater than (500*c)/(t*a) where t = the number of sets of choices, a = the number of scenarios to choose between in each choice, and c = the largest number of levels for any one attribute [29]. Assuming each person will undertake 15 sets of choices, there are two scenarios in each choice, and the largest number of levels for any one attribute is 3 then the required sample size is (500*3)/(15*2) = 50. We will administer this questionnaire to approximately 50 lung patients after staging has been completed, sampling across more than one of the imaging hubs. The questionnaire will be posted to patients with a pre-paid reply envelope. A reminder letter will be sent if no questionnaire has been returned within approximately 4 weeks. Analysis will conform to the checklist for conjoint analysis applications in health developed by the International Society for Pharmaceuticals and Outcome Research (ISPOR). We will use a random effects probit model for statistical analysis, which is appropriate given the data structure. Outcomes: The average relative importance weighting of attributes ascribed to standard versus WB-MRI staging pathways by patients diagnosed with lung cancer.

16.4.5. Handling missing data

Multiple imputation will be used for missing data using chained equations in STATA [24].

For the primary outcome, complete case analysis (all available patient data with no imputation) will be reported alongside as a sensitivity analysis to the ITT analysis.

16.5. Interim analyses

Interim safety data will be supplied to the IDMC on a periodic basis from UCL CTC on:

- Accumulating information relating to recruitment and data quality (e.g. data return rates).
- Safety data:
    - Additional tests performed as a result of the WB-MRI that would not have otherwise been performed as part of the patient's standard diagnostic pathway. Note this might include tests due to abnormalities outside colon or lung.
    - Potential harms to patients (e.g. staging complications resulting from MRI).
    - Whole Body MRIs that were not revealed or revealed too early/late.
- Percentage missing data for primary outcome.
- Prevalence of metastasis within recruited patients (key sample size assumption).

For peer review only - http://bmjopen.bmj.com/site/about/guidelines.xhtml

Streamline L

There will be no interim analysis based on the trial outcomes of diagnostic accuracy as all diagnostic test results are made available for the MDT meeting which decides first management treatment decision.

For peer review only

For peer review only - http://bmjopen.bmj.com/site/about/guidelines.xhtml

17. Ethical Approvals

In conducting the trial, the Sponsor, UCL CTC and sites shall comply with all laws and statutes, as amended from time to time, applicable to the performance of clinical trials including, but not limited to:

- the principles of ICH Harmonised Tripartite Guideline for Good Clinical Practice
- the Human Rights Act 1998
- the Data Protection Act 1998
- the Freedom of Information Act 2000
- the Research Governance Framework for Health and Social Care, issued by the UK Department of Health (Second Edition 2005) or the Scottish Health Department Research Governance Framework for Health and Community Care (Second Edition 2006)

17.1. Ethical Approval

The trial will be conducted in accordance with the World Medical Association Declaration of Helsinki entitled 'Ethical Principles for Medical Research Involving Human Subjects' (1996 version) and in accordance with the terms and conditions of the ethical approval given to the trial.

The trial has received a favourable opinion from the London – Camden & Islington Research Ethics Committee.

UCL CTC will submit Annual Progress Reports to the REC, which will commence one year from the date of ethical approval for the trial.

17.2. Site Approvals

The Lead Comprehensive Local Research Network (CLRN), Central and East London CLRN, has given NHS permission following global governance checks. Local governance checks will be undertaken by local CLRNs associated with individual trial sites.

Evidence of approval from the Trust R&D for a trial site must be provided to UCL CTC. Sites will only be activated when all necessary local approvals for the trial have been obtained.

17.3. Protocol Amendments

UCL CTC will be responsible for gaining ethical approval, for amendments made to the protocol and other trial-related documents. Once approved, UCL CTC will ensure that all amended documents are distributed to sites and CLRNs as appropriate.

Site staff will be responsible for acknowledging receipt of documents and for implementing all amendments.

For peer review only - http://bmjopen.bmj.com/site/about/guidelines.xhtml

17.4. Patient Confidentiality & Data Protection

Patient identifiable data, including full name, address, date of birth, gender and NHS number will be required for the registration process and will be provided to UCL CTC. UCL CTC will preserve patient confidentiality and will not disclose or reproduce any information by which patients could be identified. Data will be stored in a secure manner and UCL CTC trials are registered in accordance with the Data Protection Act 1998 with the Data Protection Officer at UCL. Patient identifiable data will be passed on to Birkbeck, University of London. This is described in the patient information sheet and the patients will consent to it.

For peer review only

For peer review only - http://bmjopen.bmj.com/site/about/guidelines.xhtml

18. Sponsorship and Indemnity

18.1. Sponsor Details

Sponsor Name: University College London

Address: Joint Research Office
Gower Street
London
WC1E 6BT

Contact: Director of Research Support

Tel 020 3447 9995/2178 (unit admin)

Fax 020 3447 9937

18.2. Indemnity

University College London holds insurance against claims from participants for injury caused by their participation in the clinical trial. Participants may be able to claim compensation if they can prove that UCL has been negligent. However, if this clinical trial is being carried out in a hospital, the hospital continues to have a duty of care to the participant of the clinical trial. University College London does not accept liability for any breach in the hospital's duty of care, or any negligence on the part of hospital employees. This applies whether the hospital is an NHS Trust or otherwise.

Hospitals selected to participate in this clinical trial shall provide clinical negligence insurance cover for harm caused by their employees and a copy of the relevant insurance policy or summary shall be provided to University College London, upon request.

For peer review only - http://bmjopen.bmj.com/site/about/guidelines.xhtml

19. Funding

This trial is funded by the National Institute for Health Research Health Technology Assessment (NIHR HTA).

For peer review only

For peer review only - http://bmjopen.bmj.com/site/about/guidelines.xhtml

20. Publication Policy

The TMG will oversee the publication and presentation of the data to peer reviewed journals and scientific meetings. All members of the TMG will approve publications. The writing committee will be led by Professor Stuart Taylor and include all TMG members. All TMG members, Trial Coordinator and Trial Statistician will be authors on the publications and named individually.

For peer review only

For peer review only - http://bmjopen.bmj.com/site/about/guidelines.xhtml

21. References

1. *Cancer Stats from Cancer Research UK Website*. Available from: http://info.cancerresearchuk.org.cancerstats/.
2. *NICE Lung Cancer Guidance 2011.*
3. Gilligan, D., et al., *Preoperative chemotherapy in patients with resectable non-small cell lung cancer: results of the MRC LU22/NVALT 2/EORTC 08012 multicentre randomised trial and update of systematic review.* Lancet, 2007. **369**(9577): p. 1929-37.
4. Floriani, I., et al., *Performance of imaging modalities in diagnosis of liver metastases from colorectal cancer: a systematic review and meta-analysis.* J Magn Reson Imaging, 2010. **31**(1): p. 19-31.
5. Qu, X., et al., *A meta-analysis of (1)(8)FDG-PET-CT, (1)(8)FDG-PET, MRI and bone scintigraphy for diagnosis of bone metastases in patients with lung cancer.* Eur J Radiol, 2012. **81**(5): p. 1007-15.
6. Ohno, Y., et al., *Non-small cell lung cancer: whole-body MR examination for M-stage assessment--utility for whole-body diffusion-weighted imaging compared with integrated FDG PET/CT.* Radiology, 2008. **248**(2): p. 643-54.
7. Pierce, D.A. and D.L. Preston, *Radiation-related cancer risks at low doses among atomic bomb survivors.* Radiat Res, 2000. **154**(2): p. 178-86.
8. Schmidt, G.P., M.F. Reiser, and A. Baur-Melnyk, *Whole-body MRI for the staging and follow-up of patients with metastasis.* Eur J Radiol, 2009. **70**(3): p. 393-400.
9. Squillaci, E., et al., *Staging of colon cancer: whole-body MRI vs. whole-body PET-CT--initial clinical experience.* Abdom Imaging, 2008. **33**(6): p. 676-88.
10. Yi, C.A., et al., *Non-small cell lung cancer staging: efficacy comparison of integrated PET/CT versus 3.0-T whole-body MR imaging.* Radiology, 2008. **248**(2): p. 632-42.
11. Risberg, T., et al., *Diagnostic delay causes more psychological distress in female than in male cancer patients.* Anticancer Res, 1996. **16**(2): p. 995-9.
12. Bastiaannet, E., et al., *Perception of burden experienced during diagnostic tests by melanoma patients with lymph node metastases.* Melanoma Res, 2009. **19**(1): p. 36-41.
13. von Wagner, C., et al., *Choosing between CT colonography and colonoscopy in the diagnostic context: a qualitative study of influences on patient preferences.* Health Expect, 2009. **12**(1): p. 18-26.
14. Harcourt, D., N. Rumsey, and N. Ambler, *Same-day diagnosis of symptomatic breast problems: Psychological impact and coping strategies.* Psychology, Health & Medicine 1999. **4**(1): p. 5.
15. *NICE Guide to the methods of technology appraisal 2008*. Available from: http://www.nice.org.uk/media/B52/A7/TAMethodsGuideUpdatedJune2008.pdf.
16. Bossuyt, P.M., et al., *Towards complete and accurate reporting of studies of diagnostic accuracy: The STARD Initiative.* Radiology, 2003. **226**(1): p. 24-8.
17. Bossuyt, P.M., et al., *Comparative accuracy: assessing new tests against existing diagnostic pathways.* BMJ, 2006. **332**(7549): p. 1089-92.
18. Ryan, M., et al., *Use of discrete choice experiments to elicit preferences.* Qual Health Care, 2001. **10 Suppl 1**: p. i55-60.

For peer review only

For peer review only - http://bmjopen.bmj.com/site/about/guidelines.xhtml

19. Ryan, M. and S. Farrar, *Using conjoint analysis to elicit preferences for health care.* BMJ, 2000. **320**(7248): p. 1530-3.
20. Stephens, R.J. and D.H. Johnson, *Treatment and outcomes for elderly patients with small cell lung cancer.* Drugs Aging, 2000. **17**(3): p. 229-47.
21. Julious, S.A., M.J. Campbell, and D.G. Altman, *Estimating sample sizes for continuous, binary, and ordinal outcomes in paired comparisons: practical hints.* J Biopharm Stat, 1999. **9**(2): p. 241-51.
22. Newcombe, R.G., *Improved confidence intervals for the difference between binomial proportions based on paired data.* Stat Med, 1998. **17**(22): p. 2635-50.
23. Reitsma, J.B., et al., *A review of solutions for diagnostic accuracy studies with an imperfect or missing reference standard.* J Clin Epidemiol, 2009. **62**(8): p. 797-806.
24. White, I.R., P. Royston, and A.M. Wood, *Multiple imputation using chained equations: Issues and guidance for practice.* Stat Med, 2011. **30**(4): p. 377-99.
25. Begg, C.B., R.A. Greenes, and B. Iglewicz, *The influence of uninterpretability on the assessment of diagnostic tests.* J Chronic Dis, 1986. **39**(8): p. 575-84.
26. Briggs, A.H., D.E. Wonderling, and C.Z. Mooney, *Pulling cost-effectiveness analysis up by its bootstraps: a non-parametric approach to confidence interval estimation.* Health Econ, 1997. **6**(4): p. 327-40.
27. Cooper, K.L., et al., *Positron emission tomography (PET) and magnetic resonance imaging (MRI) for the assessment of axillary lymph node metastases in early breast cancer: systematic review and economic evaluation.* Health Technol Assess, 2011. **15**(4): p. iii-iv, 1-134.
28. Briggs, A.H., K. Claxton, and M.J. Sculpher, *Decision modelling for health economic evaluation*. Oxford handbooks in health economic evaluation. 2006, Oxford: Oxford University Press. x, 237 p.
29. Johnson, R., *Conjoint Analysis Software*, I. Sawtooth Software, Editor.: Sequim, WA
30. Azvolinsky, A., *Cancer prognosis: role of BMI and fat tissue.* J Natl Cancer Inst, 2014. **106**(6): p. dju177.
31. Yip, C., et al., *Assessment of sarcopenia and changes in body composition after neoadjuvant chemotherapy and associations with clinical outcomes in oesophageal cancer.* Eur Radiol, 2014. **24**(5): p. 998-1005.
32. Attaran, S., et al., *A propensity-matched comparison of survival after lung resection in patients with a high versus low body mass index.* Eur J Cardiothorac Surg, 2012. **42**(4): p. 653-8.
33. Curvo-Semedo, L., et al., *Diffusion-weighted MRI in rectal cancer: apparent diffusion coefficient as a potential noninvasive marker of tumor aggressiveness.* J Magn Reson Imaging, 2012. **35**(6): p. 1365-71.
34. De Felice, C., et al., *Apparent diffusion coefficient on 3.0 Tesla magnetic resonance imaging and prognostic factors in breast cancer.* Eur J Gynaecol Oncol, 2014. **35**(4): p. 408-14.
35. Ohno, Y., et al., *Diffusion-weighted MRI versus 18F-FDG PET/CT: performance as predictors of tumor treatment response and patient survival in patients with non-small cell lung cancer receiving chemoradiotherapy.* AJR Am J Roentgenol, 2012. **198**(1): p. 75-82.

For peer review only - http://bmjopen.bmj.com/site/about/guidelines.xhtml

36. Zakaria, R., et al., *Diffusion-weighted MRI characteristics of the cerebral metastasis to brain boundary predicts patient outcomes.* BMC Med Imaging, 2014. **14**: p. 26.
37. Parikh, J., et al., *Changes in primary breast cancer heterogeneity may augment midtreatment MR imaging assessment of response to neoadjuvant chemotherapy.* Radiology, 2014. **272**(1): p. 100-12.

For peer review only

For peer review only - http://bmjopen.bmj.com/site/about/guidelines.xhtml

Appendix 1: Abbreviations

**CDR**	Clinical Data Repository
**CI**	Chief Investigator
**CLRN**	Comprehensive Local Research Network
**CRF**	Case Report Form
**CSP**	Coordinated System for gaining NHS Permission
**CT**	Computerised Tomography
**CTSA**	Clinical Trial Site Agreement
**DCE**	Direct Choice Experiment
**DPA**	Data Protection Act
**GCP**	Good Clinical Practice
**GHQ**	General Health Questionnaire
**ICER**	Incremental Cost-Effectiveness Ratio
**ICH GCP**	International Conference of Harmonisation-Good Clinical Practice
**IDMC**	Independent Data Monitoring Committee
**IRAS**	Integrated Research Application System
**ISF**	Investigator Site File
**ISRCTN**	International Standard Randomised Controlled Trial Number
**ITT**	Intention To Treat
**MDT**	Multi-Disciplinary Team
**MRI**	Magnetic Resonance Imaging
**NCRI**	National Cancer Research Institute
**NCRN**	National Cancer Research Network
**NHS**	National Health Service
**NICE**	National Institute for Clinical Excellence
**NIHR**	National Institute for Health Research
**NIHR HTA**	National Institute for Health Research Health Technology Assessment
**NRES**	National Research Ethics Service
**OPD**	Out Patients Department
**PACS**	Picture Archiving and Communications System
**PANAS**	Positive and Negative Affect
**PET**	Positron Emission Tomography
**PET-CT**	Positron Emission Tomography- Computerised Tomography
**PI**	Principal Investigator
**PSS**	Personal Social Services
**QA**	Quality Assurance
**QALY**	Quality Adjusted Life Year
**R&D**	Research & Development
**RCT**	Randomised Controlled Trial
**REC**	Research Ethics Committee
**RIS**	Radiology Information System
**SSI**	Site Specific Information
**STARD**	STAndards for the Reporting of Diagnostic accuracy studies
**T**	Tesla

For peer review only - http://bmjopen.bmj.com/site/about/guidelines.xhtml

Streamline L

**TMF**	Trial Master File
**TMG**	Trial Management Group
**TSC**	Trial Steering Committee
**UCL**	University College London
**UCL CTC**	Cancer Research UK & UCL Cancer Trials Centre
**UCLH**	University College London Hospital
**USS**	Ultrasound Scan
**VATS**	Video-Assisted Thoracoscopic Surgery
**WB-MRI**	Whole Body Magnetic Resonance Imaging
**WCBP**	Woman of Childbearing Potential

For peer review only

For peer review only - http://bmjopen.bmj.com/site/about/guidelines.xhtml

Appendix 2: Consent Process

Patients will be recruited at several hospital sites, and for many, the actual WB-MRI will take place at a different hospital (one of the central imaging hubs). It is vital that recruited patients undergo informed consent, have a minimum of 24 hours to consider participation and are subsequently free to withdraw at any time.
The consent process must be sensitive to the needs of patients which may differ from individual to individual. It is important therefore to provide patients with options as to how they may provide their consent.

The proposed consent process for this trial is based on the experience of other similar trials. It was found that patients and recruitment sites found it more efficient if the designated recruiting individual at the peripheral hospital knows that after fully explaining the trial, the patient in principle is willing to take part. For all recruitment sites, patients are seen by a clinical practitioner after a suspected diagnosis of lung cancer based on an abnormal chest X-Ray and CT scan. At this discussion the diagnosis is explained and the need for a series of staging tests (such as PET CT, bronchoscopy, biopsy etc.) is also explained. Because of the need to stage the patient quickly so treatment may start, these tests are often done within a very short time period. There is no scheduled return by the patient to an OPD during this staging process. Instead patients are next seen face to face after the MDT has made a decision about their treatment.

If consent in principle is given, the recruiter can then ring the central hub and there and then arrange the time of the MRI and thus whenever possible inform the patient immediately of the time and date so they can plan their attendance for the scan. Patients expressing an interest in participating in the trial will therefore be asked if they prefer to sign the consent form when first seen when the trial is explained (and this will be later filed in the medical notes and the conversation documented). It will be made perfectly clear that they are free to withdraw at any time and that the scan will be at least 24 hours later (usually more than this). This will save them having to re-attend the hospital simply to sign a consent form, incurring time and expense at a very busy and difficult time for them. Before they go home, where possible patients will be given the date of the WB-MRI and a copy of their signed consent form. The recruitment site can then register the patient with UCL CTC. The date of the WB-MRI will be at least 24 hours later and in reality likely several days later. The patient will take the full patient information sheet home to review at their leisure. They will be provided with a telephone number at the recruitment site to ring if on reflection, they want to opt out of the trial. If they opt out, this will be recorded in the medical notes and screening log. Patients will also be asked permission to be called by the recruitment site or imaging hub to remind them of the date of scan and confirm their attendance. Patients will retain the right to opt out at any time by cancelling or not attending their WB-MRI appointment.

As noted, a major advantage of this approach is that the patient does not have to make a special trip back to the hospital just to sign the consent form. The signed consent

For peer review only - http://bmjopen.bmj.com/site/about/guidelines.xhtml

form does not leave the recruitment hospital and can be more efficiently filed in the patient notes and a copy filed in the Investigator site file: an important aspect of trial governance and patient clinical care.

Note this method of consent has been previously deemed ethically sound and efficient in the NIHR HTA funded Siggar 1 trial (CT colonography, colonoscopy or barium enema for diagnosis or colorectal cancer in older symptomatic patients), Northern and Yorkshire MREC, MREC/3/3/075, which ran from 2004 to 2009 and recruited over 5000 patients to undergo CT colonography in a similar trial design to the current proposal. The trial team did pilot a scheme where patients returned a copy of their signed consent form after 24 hours if they still wanted to take part, but patients found this very cumbersome and confusing and much preferred the process proposed, using telephone confirmation. The procedure is supported by the patient representatives in the trial and has also worked successfully in the BOOST trial which randomised patients to standard care or endobronchial biopsy at exactly the same point in the patient pathway. The BOOST trial ran at many of the intended recruitment sites for Streamline L.

Patients will be given the option to come back and sign the consent form if they or their recruiter feel this is preferable.

Occasionally it may be more convenient for the patient to obtain written consent when the patient attends for the WB-MRI or before a planned conventional staging test. For example the patient may wish to take part in the trial, not wish to sign the consent form on the same day but also would prefer not to attend a hospital appointment just to sign the consent form. This is acceptable although it is the least desirable option. After the patient has signed consent they will need to be registered before proceeding with the WB-MRI and a copy of the consent form will need to be returned to the initial recruitment site.

For peer review only - http://bmjopen.bmj.com/site/about/guidelines.xhtml

Appendix 3: Minimum WB-MRI protocol dataset

MRI Protocol
Whole body is head to thigh

The aim is to complete the whole protocol in 60 minutes or less. Depending on the MRI technology available, it may be possible to use the 5mm slices for all axial imaging, although up to 7mm is acceptable if time constraints are problematic.

1. These specifications are a set of minimum requirements – where higher field strengths/scanner software permits the resolution/quality of sequences should be optimised (whilst keeping the maximum imaging time limited to 1 hour).

2. Scanning maybe performed at either 1.5T or 3T.

3. Whole-body coverage is defined as head to mid-thigh optimised for detection of metastases.

Imaging Procedure

a) A standard safety questionnaire should be completed.
b) For patients undergoing contrast enhancement: set up IV line in a vein in the antecubital fossa, connected to an automated injector with two syringes (contrast and saline flush).
c) Unless contra-indicated administer 20mg buscopan or 1mg glucagon iv. to be given just before the start of the scan.

1. Whole-body diffusion weighted imaging:
Axial: STIR-EPI (or other fat sat technique) diffusion weighted imaging. Fixed slice thickness of 5mm to 7mm (to match T2 and t1 weighted axials as below) two b-values (b50 and b900). A minimum acquisition matrix of 128 x 128 (or an interpolated equivalent) (rectangular FOV should be used if available and appropriate for the patient), as a reference a minimum SNR of 6 on b50 images (for liver) should be maintained if possible by increasing the number of averages. All imaging should be performed in gentle respiration (recommended as 4 stations of 50 slices beginning from the vertex to mid thighs). Diffusion imaging through the brain is optional.

2. Whole-body T2 weighted imaging:
Axial*:* Axial T2 weighted (without fat-suppression) imaging should be performed from vertex to mid-thigh. A 5 to 7 mm slice thickness should be used for all scanners. *Where possible (within the 60 minute imaging time)* respiratory and ECG triggering should be used for the chest, respiratory triggering alone for the upper abdomen. The head, neck, pelvis and legs should be scanned without any triggering. The number of stacks should be adjusted to cover the imaging volume.

For peer review only - http://bmjopen.bmj.com/site/about/guidelines.xhtml

4. **Pre-contrast T1 weighted imaging:**

DIXON Technique to be applied if available.

a. Axial: Whole-body T1 GRE (e.g. Flash 2D) non-contrast enhanced non fat sat. Image resolution and slice thickness should be ideally matched to T2 weighted imaging.

OR

b. Coronal: T1 fat saturated volume interpolated gradient echo imaging (e.g. 3D) pre contrast.

5. **Post-contrast T1 weighted imaging (if gadolinium not contraindicated or refused):**

Minimum data set
Axial liver (60-70 sec)
Axial lung (equilibrium phase)
SFOV axial head

Optional
Coronal (organ specific or whole body)

a. Axial: post contrast e.g. T1 fat saturated volume interpolated gradient echo imaging (3D) breath hold of the *liver (60-70 seconds delay) and lungs*. Multiple breath-holds employed to provide full volume coverage if required. A minimum of a 256x256 (rectangular FOV acquisition if possible and appropriate for the patient) acquisition matrix should be employed. 5-7 mm slice thickness.
b. Coronal: post contrast whole body; e.g. T1 fat saturated volume interpolated gradient echo imaging (3D) and post contrast. Slice thickness 5mm. Breath Hold.
c. **Axial:** fat saturated T1 weighted imaging of the brain (SFOV). An acquisition matrix of 256 x 256 should be employed.

For peer review only - http://bmjopen.bmj.com/site/about/guidelines.xhtml

Appendix 4: Exploratory Analysis

The collated WB-MRI datasets and 12 months patient follow up data collated as part of the trial protocol provides opportunities to retrospectively mine the data for additional diagnostic indices and prognostic markers. All analysis will be done by members of the research team under supervision of the Chief Investigator.

There is for example, increasing data linking total body fat with prognosis in malignancy [30, 31]. In lung cancer, a higher body mass index has been associated with a better prognosis [32]. From the WB-MRI datasets acquired as part of the trial, it is possible to extract quantitative data on total body fat using simple segmentation algorithms which can be linked with patients' outcomes such as metastatic status and 12 month survival.

In addition, quantification of the MRI signal returned from sites of disease may be able to predict histological characteristics and also may be linked to prognosis. For example diffusion weighted imaging of the primary tumour has been shown to differentiate between grades of dysplasia in rectal cancer [33] and may predict prognosis in lung, brain and breast cancer [34-36]. The heterogeneity of signal within tumours can be measured and has been linked with overall prognosis and treatment response. For example in breast cancer, increased homogeneity of the signal measured using regions of interest placed in metastatic deposits in response to treatment is associated with a better prognosis [37]. The various sequences performed as part of the collected WB-MRI protocol will allow testing of such associations in recruited patients.

The following or similar may be quantified in all or a subset of WB-MRI datasets:

1) Body fat (organ, visceral and whole body).
2) MRI signal from the primary tumour and metastatic sites, notably diffusion weighted signal, apparent diffusion coefficient, T1 (including fat and water components), T2 signal, Contrast enhancement, and textural analysis (for heterogeneity) of the primary and metastatic deposits on the various sequences.

We will look for correlations between MRI variables and clinical data collated as part of the trial (for example basic histology, metastatic status, patient outcome and findings of conventional staging tests) or collected as part of the patients' usual care, for example, more detailed histology of biopsies or excised tumour (including tumours markers if collected as part of usual clinical care). Associations will be sought between the MRI variables and the clinical variables using appropriate conventional parametric and non-parametric statistics, for example:

- Differences in body fat composition between different cancer histological subtypes, patients with and without metastatic disease, and according to 12 month survival.
- Heterogeneity of MRI signal from a region of interest drawn within the primary tumours and metastatic deposits, comparing between lesions in the same

For peer review only - http://bmjopen.bmj.com/site/about/guidelines.xhtml

patient, between different patients and according to histological parameters and 12 month survival.

In addition comparisons will be made between MRI signal within normal body tissues (such as the liver, pancreas and muscle) between patients scanned on the different MRI scanners comparing with local MRI phantom data as appropriate to see how homogenous the MRI data is between different imaging hubs.

In some instances use of external commercial software may be required (e.g. fat quantification). MRI datasets will be anonymised and no personal identifiable information will be shared outside the research team at any time.

For peer review only

For peer review only - http://bmjopen.bmj.com/site/about/guidelines.xhtml

Appendix 5: Protocol Version History

**Protocol:**		**Amendments:**		
**Version no.**	**Date**	**Amendment no.**	**Protocol Section (no./title)**	**Summary of main changes from previous version.**
1.0				
1.1	14/09/12	N/A	Front cover & Section 1.1<br>Section 5<br>8.2.10<br>Section 14.2<br>Appendix 2	ISRCTN number added and new CR UK logo added<br>Flowchart and inserted re consent process<br>Text inserted re QoLs<br>Text inserted re destruction of interview recordings<br>Text inserted re consent process
2.0	15/03/13	1.0	Throughout<br>Section 4<br>Section 5<br>Section 7<br>Section 8<br>Section 9<br>Section 12<br>Section 13<br>Appendix 3	Typos and clarifications<br>Clarification about GCP training and documents required for activation updated<br>Details added about interpreters for informed consent and removal of informed consent log information<br>Removal of text regarding slot availability updates<br>Addition of ad hoc mini MDT, reduce the amount of patient experience questionnaires, change the content of these questionnaires and alter who is sending them to patients. Clarifications of timing of WB-MRI and the process of early release of findings<br>Update data management guidelines in line with changes at UCL CTC<br>Details removed about informed consent log<br>Addition of details re patient withdrawal<br>Clarifications to Whole body MRI protocol
3.0	24/04/14	3.0	Section 1<br>Section 5<br>Section 6<br>Section 7<br>Section 8	Rewording of the inclusion criteria<br>Removal of information about QoL and Patient diaries given out by site at consent<br>Rewording of the MDT log information and inclusion criteria<br>Removal of patient diaries and QoLs from registration packs.<br>Rewording and clarification of Conventional Imaging, WB-MRI reveal and MDT discussion, Final Treatment decision and follow up sections. Updating information to state that the Interview section of the trial is now closed to recruitment. Updating information that UCL CTC is now posting out QoL and patient diaries at baseline.

For peer review only - http://bmjopen.bmj.com/site/about/guidelines.xhtml

Streamline L

4.0	22/12/14	4.0	Section 8.2.12<br><br>Section 10<br><br>Appendix 4<br><br>Appendix 5	Information added regarding exploratory analysis for additional diagnostic indices and prognostic markers.<br>Safety Reporting section updated to provide more information on staging complications and how these are reported and reviewed by the CI.<br>Exploratory Analysis added to include the additional analysis of WB-MRI scans<br>Protocol Version History has become Appendix 5.
5.0	11/05/15	5.0	Section 8<br><br>Section 10<br><br>Section 13<br><br>Section 16	Changing target recruitment for patient experience questionnaires to 50 patients and closing this part of the trial. Updating and clarifying information regarding the Discrete Choice Experiment and confirming the time point these will be sent to patients.<br>Updating the Safety Section to confirm that only staging complications related to the WB-MRI or additional tests performed due to WB-MRI are to be recorded on the Staging Complications CRF.<br>The withdrawal process was updated and clarified to better match the new Change of Status CRF.<br>The type of Interim safety data that will be supplied to IDMC was updated
6.0	06/09/16	6.0	Sections 1.1 & 1.2<br><br>Section 13<br><br>Section 16.1	Recruitment target increased from 250 to 353<br>Anticipated duration of recruitment increased from 3 to 4 years.<br>Deletion of section on early release of WB MRI and patient failure to undergo WB MRI<br>Rationale for revised target accrual added<br>Grammatical and typographical errors corrected throughout

For peer review only

For peer review only - http://bmjopen.bmj.com/site/about/guidelines.xhtml

Streamline C

Streamlining Staging of Colorectal Cancer with Whole Body MRI

Trial Sponsor:	University College London (UCL)
Trial Sponsor reference:	UCL/11/0097
Trial funder:	NIHR HTA
Funder reference:	10/68/01
ISRCTN no:	43958015
Protocol version no:	6.0
Protocol version date:	06/09/2016

This project was funded by the National Institute for Health Research (NIHR) Health Technology Assessment (HTA) programme *(project number 10/68/01)*

For peer review only

Protocol version 6.0, 06/09/2016 Authorisation signatures

Name & Role:	Signature:	Date authorised:
**Chief Investigator:**<br>Professor Stuart Taylor<br>Professor of Medical Imaging, UCL		14/10/16
**For the Sponsor:**<br>Professor Jonathan Ledermann<br>Director, UCL CTC		14 Oct 2016
Sandy Beare<br>Tumour Group Lead, UCL CTC		14/10/2016

Please note: This trial protocol must not be applied to patients treated outside the Streamline C trial. UCL CTC can only ensure that approved trial investigators are provided with amendments to the protocol.

For peer review only - http://bmjopen.bmj.com/site/about/guidelines.xhtml

Coordinating Centre:

For general queries, supply of trial documentation and central data management please contact:

Streamline C Trial Coordinator
Cancer Research UK & UCL Cancer Trials Centre (UCL CTC)
90 Tottenham Court Road
London
W1T 4TJ

Tel: +44 (0) 20 7679 9878
Fax: +44 (0) 20 7679 9871
09:00 to 17:00 Monday to Friday

Email: ctc.streamlineC@ucl.ac.uk

Other trial contacts:

Chief Investigator: Professor Stuart Taylor
Address: Dept Specialist X Ray, Level 2 Podium,
University College London Hospital (UCLH)
235 Euston Road
London
NW1 2BU

Trial Management Group (TMG):

Prof Stuart Taylor	Professor of Medical Imaging	UCLH
Dr Dow-Mu Koh	Consultant Radiologist	The Royal Marsden
Prof Vicky Goh	Professor of Clinical Cancer Imaging	Guys and St Thomas
Prof Anwar Padhani	Clinical Lead in MRI	Mount Vernon
Dr Shonit Punwani	Senior Lecturer in Medical Imaging	UCLH
Prof Andrea Rockall	Professor of Cancer Imaging	Imperial College London
Prof Steve Halligan	Professor of Radiology	UCL
Dr Neal Navani	Consultant in Thoracic Medicine	UCLH
Prof Sam Janes	Wellcome Senior Fellow	UCLH
Dr John Bridgewater	Consultant Oncologist	UCLH
Alison Morton	Patient Representative	
Alf Oliver	Patient Representative	
Dr Anne Miles	Senior Lecturer in Health Psychology	Birkbeck College
Prof Steve Morris	Professor of Health Economics	UCL
Dr Susan Mallett	Senior Medical Statistician	University of Birmingham
Krystyna Reczko	Trial Coordinator	UCL CTC
Katie Prior	Data Manager	UCL CTC
Marian Duggan (maternity leave)	Senior Trial Coordinator	UCL CTC
Abby Sharp (maternity cover)	Senior Trial Coordinator	UCL CTC
Dr Sandy Beare	Tumour Group Lead	UCL CTC

For peer review only - http://bmjopen.bmj.com/site/about/guidelines.xhtml

Table of Contents

1. PROTOCOL SUMMARY 7
1.1. SUMMARY OF TRIAL DESIGN 7
1.2. TRIAL SCHEMA 9
2. INTRODUCTION 10
2.1. BACKGROUND 10
3. TRIAL OVERVIEW 12
3.1. PRIMARY OBJECTIVE 12
3.2. SECONDARY OBJECTIVES 12
3.3. TRIAL ACTIVATION 13
4. SELECTION OF SITES/SITE INVESTIGATORS 14
4.1. SITE SELECTION 14
4.1.1. Selection of Principal Investigator and other investigators at sites 14
4.1.2. Training requirements for site staff 14
4.2. SITE INITIATION AND ACTIVATION 15
4.2.1. Site initiation 15
4.2.2. Required documentation 15
4.2.3. Site activation letter 15
5. INFORMED CONSENT 16
6. SELECTION OF PATIENTS 18
6.1. PRE-REGISTRATION EVALUATION 18
6.2. SCREENING LOG 18
6.3. MULTI DISCIPLINARY TEAM (MDT) MEETING LOG 18
6.4. PATIENT ELIGIBILITY 18
6.4.1. Patient Inclusion Criteria 18
6.4.2. Exclusion Criteria 19
6.4.3. Pregnancy and Birth Control 19
7. REGISTRATION PROCEDURES 20
7.1. REGISTRATION 20
7.2. REGISTRATION PACKS 20
7.3. BOOKING WHOLE BODY MRI 20
8. PATIENT MANAGEMENT AND TRIAL PROCEDURES 22
8.1. SUMMARY 22
8.1.1. Staging and Treatment Decisions 22
8.1.2. Patient experience Interviews and Questionnaires (Optional) 23
8.1.3. Quality of Life Questionnaires and Patient Diaries 23
8.2. DETAILED DESCRIPTION 25
8.2.1. Standard care 25
8.2.2. Trial Imaging 25
8.2.3. Release of Conventional and trial WB-MRI findings 27
8.2.4. Final treatment plan 28
8.2.5. Early release of WB-MRI findings 29
8.2.6. Time to full staging 29
8.2.7. Patient follow up 30
8.2.8. Patient diaries and EQ-5D Quality of Life questionnaires 30
8.2.9. Final reference standard for tumour stage (Consensus Meetings) 30
8.2.10. Health psychology assessment 32

For peer review only - http://bmjopen.bmj.com/site/about/guidelines.xhtml
For peer review only

8.2.11. Cost Effectiveness Assessment 35
8.2.12. Nested substudies 37

9. DATA MANAGEMENT GUIDELINES 39

9.1. COMPLETING CASE REPORT FORMS 39
9.2. MISSING DATA 39
9.3. TIMELINES FOR DATA RETURN 39
9.4. DATA QUERIES 40

10. SAFETY REPORTING 41

11. INCIDENT REPORTING 42

12. TRIAL MONITORING AND OVERSIGHT 43

12.1. CENTRAL MONITORING 43
12.2. 'FOR CAUSE' ON-SITE MONITORING 43
12.3. OVERSIGHT COMMITTEES 44
12.3.1. Trial Management Group (TMG) 44
12.3.2. Trial Steering Committee (TSC) 44
12.3.3. Independent Data Monitoring Committee (IDMC) 44
12.3.4. Role of UCL CTC 44

13. WITHDRAWAL OF PATIENTS 45

14. TRIAL CLOSURE 46

14.1. END OF TRIAL 46
14.2. ARCHIVING OF TRIAL DOCUMENTATION 46
14.3. EARLY DISCONTINUATION OF TRIAL 46
14.4. WITHDRAWAL FROM TRIAL PARTICIPATION BY A SITE 46

15. QUALITY ASSURANCE 47

16. STATISTICS 48

16.1. SAMPLE SIZE CALCULATION 48
16.2. POPULATION FOR ANALYSIS 49
16.3. ANALYSIS OF THE PRIMARY ENDPOINT 49
16.4. ANALYSIS OF SECONDARY ENDPOINTS 50
16.4.1. Efficacy (secondary) 50
16.4.2. Safety 52
16.4.3. Economic evaluation 52
16.4.4. Health psychology assessment 53
16.4.5. Handling missing data 54
16.5. INTERIM ANALYSES 54

17. ETHICAL APPROVALS 55

17.1. ETHICAL APPROVAL 55
17.2. SITE APPROVALS 55
17.3. PROTOCOL AMENDMENTS 55
17.4. PATIENT CONFIDENTIALITY & DATA PROTECTION 56

18. SPONSORSHIP AND INDEMNITY 57

18.1. SPONSOR DETAILS 57
18.2. INDEMNITY 57

19. FUNDING 58

20. PUBLICATION POLICY 59

For peer review only - http://bmjopen.bmj.com/site/about/guidelines.xhtml

Streamline C

21. REFERENCES 60
APPENDIX 1: ABBREVIATIONS 63
APPENDIX 2: CONSENT PROCESS 65
APPENDIX 3: MINIMUM WB-MRI PROTOCOL DATASET 67
APPENDIX 4: EXPLORATORY ANALYSIS 69
APPENDIX 5: PROTOCOL VERSION HISTORY 71

For peer review only

For peer review only - http://bmjopen.bmj.com/site/about/guidelines.xhtml

1. Protocol Summary

1.1. Summary of Trial Design

**Title:**	Streamlining Staging of Colorectal Cancer with Whole Body MRI
**Short Title/acronym:**	Streamline C
**Sponsor name & reference:**	UCL/11/0097
**Funder name & reference:**	NIHR HTA
**ISRCTN no:**	43958015
**Design:**	Multicentre comparison
**Overall aim:**	To evaluate whether early whole body magnetic resonance Imaging (WB-MRI) increases per patient sensitivity for metastasis in colorectal cancer compared to standard NICE-approved diagnostic pathways.
**Primary endpoint:**	Per patient sensitivity for metastasis detection by whole body MRI (WB-MRI) compared to standard staging pathways in newly diagnosed colorectal cancer
**Secondary endpoints:**	1. The time and test number taken to reach, and the nature of, the first major treatment decision based on WB-MRI in comparison to standard staging pathways.<br>2. Diagnostic accuracy of WB-MRI and conventional staging pathways for local tumour staging and detection of metastasis in comparison to an expert derived consensus reference standard.<br>3. Lifetime incremental cost and cost-effectiveness of staging using WB-MRI compared to standard diagnostic pathways.<br>4. Patient experience of staging using WB-MRI in comparison to standard diagnostic pathways and priorities placed by patients on differing attributes related to competing staging pathways.<br>5. Inter-observer variability in WB-MRI analysis and effect of diagnostic confidence on staging accuracy.<br>6. Diagnostic accuracy of limited T1 and diffusion weighted sequences compared to full multi-sequence WB-MRI protocols.
**Target accrual:**	360
**Inclusion & exclusion criteria:**	**Inclusion criteria:**<br>• Adult patients (18 or over) with histologically

For peer review only - http://bmjopen.bmj.com/site/about/guidelines.xhtml

	proven or suspected colorectal cancer referred for staging.<br>• Suspicion of colorectal cancer defined as: Presence of a mass highly suspicious for colorectal cancer on endoscopy, barium enema, CT colonography or other imaging which triggers staging investigations.<br>• Patient must have given written informed consent and be willing to comply with the protocol intervention and follow up.<br>**Exclusion criteria:**<br>• Any psychiatric or other disorder likely to impact on informed consent<br>• Evidence of severe or uncontrolled systemic disease which make it undesirable for the patient to participate in the trial<br>• Pregnancy<br>• Contraindications to MRI (e.g. cardiac pacemaker, severe claustrophobia, inability to lie flat)<br>• Polyp cancer (because metastatic disease in these patients is vanishingly rare)
**Planned number of sites:**	14 (including 7 Imaging hubs)
**Target Country**	UK
**Trial Procedure:**	All patients will undergo a whole body MRI protocol in addition to the standard staging protocol employed at their institution.
**Anticipated duration of recruitment:**	4 years
**Duration of patient follow up:**	12 months
**Definition of end of trial:**	12 months after enrolment of the final patient.
**Other related research:**	Sub-study of WB-MRI generalisability

For peer review only

For peer review only - http://bmjopen.bmj.com/site/about/guidelines.xhtml

1.2. Trial Schema

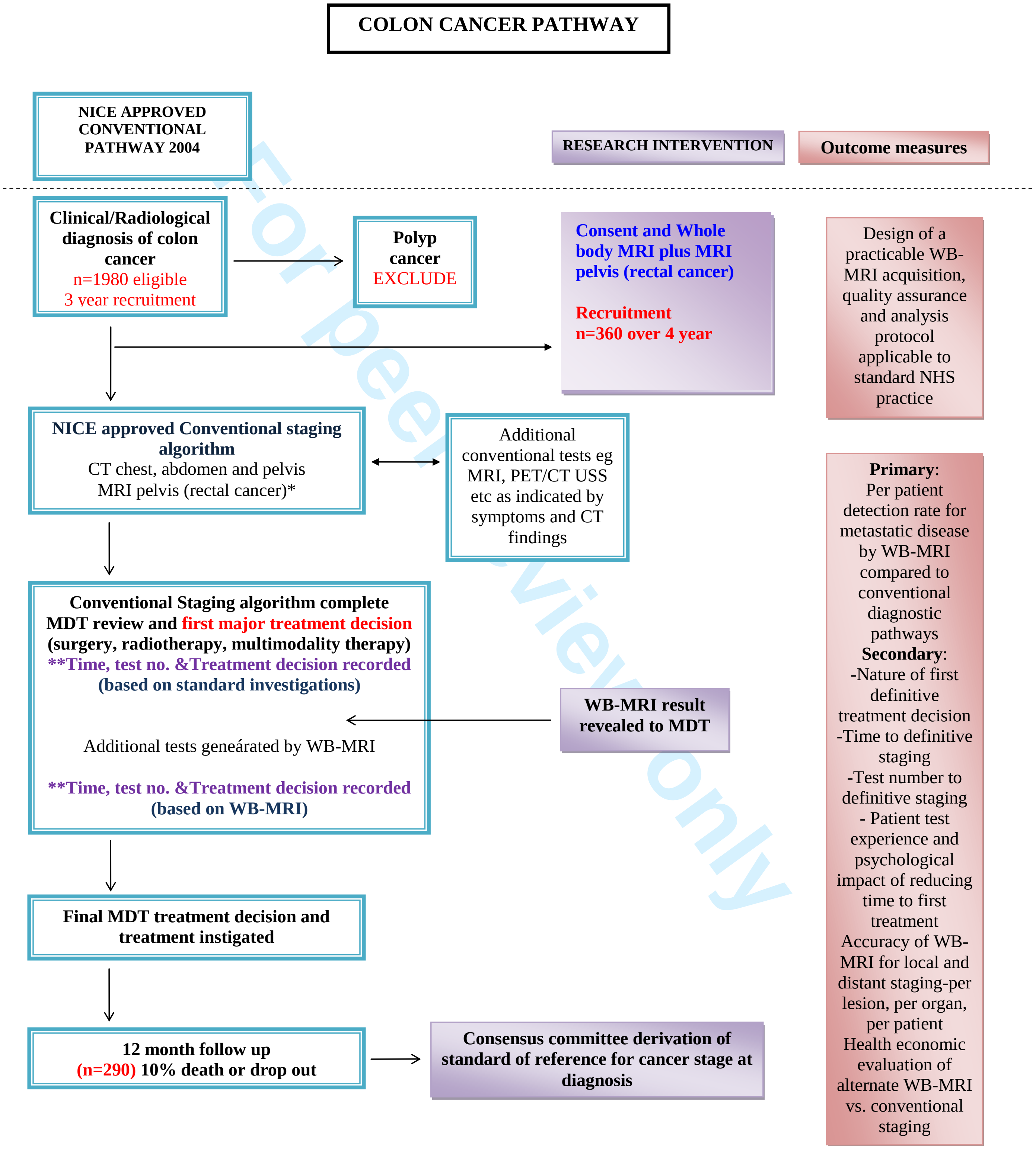

For peer review only

For peer review only - http://bmjopen.bmj.com/site/about/guidelines.xhtml

2. Introduction

2.1. Background

Colorectal cancer is the third most common malignancy in the UK and accounts for 13% of all new cancer diagnosis; approximately 40,000 patients are diagnosed per year [1]. Treatment decisions for colorectal cancer are critically dependent upon rapid and accurate tumour staging at diagnosis. In particular detection of metastatic disease (i.e. disease which has disseminated beyond the primary tumour into distant organ sites such as bone, liver and brain) is vital to optimised therapeutic triage. More aggressive approaches to treating metastatic colorectal cancer using combined chemotherapy and surgical resection regimens are widely adopted in routine National Health Service (NHS) practice [2].

Success rates however depend on the accuracy of initial disease staging so all disease sites are appropriately identified and treated [3]. Currently around 80% of patients with colorectal cancer undergo primary surgery with curative intent, although up to 50% subsequently relapse with metastatic disease, often within 12 months [4], in part reflecting sub-optimal disease staging. Accurate local staging of the tumour and spread to lymph nodes is also of importance. Imaging derived local staging in rectal cancer dictates the use of pre-operative (neo-adjuvant) chemotherapy. Furthermore, trials investigating neoadjuvant chemotherapy for non-rectal colonic tumours are currently recruiting in the NHS (FOXTROT).

Staging of colorectal cancer within the NHS is reliant on high technology imaging platforms such as computerised tomography (CT), positron emission tomography (PET) and magnetic resonance imaging (MRI), together with standard scintigraphy, plain X-rays and ultrasound. Staging pathways are complex (see flowchart, section 1.2), and governed by National Institute for Clinical Excellence (NICE) approved guidelines [5] which delineate an integrated step by step deployment of the various imaging modalities before tumour staging is deemed complete and the first treatment decision is made.

The complexity of current staging algorithms reflects the differing diagnostic attributes of the various imaging modalities across tumour types and body organs. CT for example is superior to ultrasound for detecting liver metastasis [6], but inferior to scintigraphy in diagnosing malignant spread to the bone. PET scanning is in general superior to scintigraphy for detecting bone metastases [7], but not as effective as MRI in identifying spread to the brain [8]. It is not unusual for a patient with colorectal cancer to undergo CT chest abdomen and pelvis, together with pelvic MRI in the case of rectal cancer. However additional tests such as ultrasound, MRI and PET-CT are increasingly used in cases of known or suspected metastatic disease. Such a stepwise multi-modality approach is time and resource intensive and onerous for patients at a very difficult time following their cancer diagnosis. Furthermore patients receive significant doses of diagnostic ionising radiation during the staging process; a single PET-CT imparts 6-10 times normal annual background radiation and even small doses of radiation can increase an individual's risk of subsequent malignancy [9].

Recent technological advances mean an MRI scan of the whole body can be acquired in less than one hour; sensitivity for metastatic cancer spread is high [10]. MRI does

For peer review only - http://bmjopen.bmj.com/site/about/guidelines.xhtml

not impart ionising radiation and is advocated as a safe and effective alternative to the current multi-modality approach. There is no available secondary research evidence concerning the accuracy of whole body MRI (WB-MRI) in cancer staging. Indeed meta-analysis would be challenging because literature is limited to relatively small cohort studies comparing WB-MRI with conventional imaging tests, and in a wide variety of cancers. These do however suggest potential as an efficient and accurate "one stop shop" alternative for cancer staging [8, 11, 12]. For example, in 123 patients with non-small cell cancer, Yi found no difference between PET-CT and WB-MRI, with both achieving 86% accuracy [12]. Notably, the vast majority of available studies are single site, using one MRI platform, with interpretation by one or two experienced radiologists.

The literature is deficient regarding generalisability of WB-MRI. For example, variability amongst interpreting radiologists is unknown and the effect (if any) of different manufacturers' platforms is uncertain. It is also unclear how WB-MRI can be best integrated into existing NHS pathways, which frequently differ between institutions, or how effective it would be in different clinical settings. By undertaking a multicentre trial of colorectal cancer using a variety of MRI platforms and interpreting radiologists, the current proposal will address these unknown variables.

Health psychology assessment

WB-MRI staging of colorectal cancer could reduce the physical and psychological burden associated with current pathways. By reducing time from diagnosis to treatment, WB-MRI may reduce patient anxiety, minimising concerns that treatment delay might adversely affect prognosis. Delay in diagnosis is known to be distressing, particularly for women [13]. In addition, staging scans can be uncomfortable and embarrassing [14]. MRI for example, while avoiding radiation, is noisy and claustrophobic. The potential of WB-MRI to generate additional tests is also an important consideration, although recent data suggest test sensitivity is the key determinant of patient choice and influences preference more than discomfort and complications [15]. While rapid staging means patients without metastasis are in receipt of good news more quickly, it also means those with metastasis will also receive bad news earlier, which may impact adversely on psychological wellbeing: It is known that patients are more depressed following a speedier diagnosis of breast cancer (at a one-stop clinic) when compared to those waiting a week [16].

Cost Effectiveness Analysis

Although WB-MRI is a relatively expensive imaging technology, other standard tests in the staging pathways such as PET-CT are also resource intensive. A detailed analysis of the cost and the cost-effectiveness of WB-MRI versus standard NICE-approved staging algorithms conforming to accepted economic evaluation methods [17] is essential to any trial investigating the role of WB-MRI in cancer staging.

For peer review only - http://bmjopen.bmj.com/site/about/guidelines.xhtml

3. Trial Overview

This trial will be an independent multi-centre prospective cohort trial comparing the staging accuracy of early WB-MRI with standard pathways in newly diagnosed colorectal cancer. We will follow the recommendations of the STAndards for the Reporting of Diagnostic accuracy studies (STARD) initiative [18]. A key element of our design is the investigation of early WB-MRI as a replacement test for standard pathways [19], and data regarding the therapeutic impact of WB-MRI will be collated, as well as addressing the practicalities of general NHS implementation. The trial design, which compares two different staging strategies in the same patients ensures that clean, comparable data are collected for both pathways while simultaneously increasing trial power to meet trial endpoints.

In addition, both qualitative and quantitative assessments will be employed to determine the psychological burden and acceptability of WB-MRI versus standard pathways, and to identify those attributes which most strongly influence patient preference. Health-related quality of life data will be collected which will be used to inform cost-effectiveness modelling, and determine whether patients believe more rapid staging would have helped them cope better with their cancer diagnosis.

3.1. Primary objective

To evaluate whether early whole body magnetic resonance Imaging (WB-MRI) increases per patient sensitivity for metastasis in colorectal cancer compared to standard NICE-approved diagnostic pathways.

3.2. Secondary objectives

1) To determine how WB-MRI influences time to and nature of first major treatment decision following definitive staging compared to standard investigations and to determine whether early WB-MRI could reduce or replace standard investigations.
2) To assess the accuracy of WB-MRI and standard diagnostic pathways for local and distant staging by comparison with an expert derived consensus reference standard using 1-year patient follow up data.
3) To evaluate the lifetime incremental cost and cost-effectiveness of staging using WB-MRI compared to standard diagnostic pathways.
4) To evaluate patients' experiences of staging using WB-MRI and to determine the priorities placed by patients on differing attributes related to competing staging pathways, including impact of reducing time to first treatment.
5) To determine the effect of radiologist confidence on the diagnostic accuracy of WB-MRI for metastatic disease.
6) To evaluate the diagnostic performance of limited WB-MRI protocols based on diffusion and T1 weighted imaging only, and to assess the incremental benefit of intravenous gadolinium contrast enhancement.

Additional objectives which will be met by the trial will be the definition of an efficient WB-MRI acquisition and analysis protocol, and quality assurance (QA) program

For peer review only - http://bmjopen.bmj.com/site/about/guidelines.xhtml

applicable to standard NHS practice and collation of a database of WB-MRI datasets for NHS staff training and service development.

3.3. Trial activation

UCL CTC will ensure that all trial documentation has been reviewed and approved by all relevant bodies and that the following have been obtained prior to activating the trial:

- Research Ethics Committee approval
- 'Adoption' into NIHR portfolio
- NHS permissions
- Adequate funding for central coordination
- Confirmation of sponsorship
- Adequate insurance provision

For peer review only

For peer review only - http://bmjopen.bmj.com/site/about/guidelines.xhtml

4. Selection of Sites/Site Investigators

4.1. Site selection

In this protocol a ‘recruitment site’ refers to a hospital where certain trial-related activities are conducted.

Recruitment sites must be able to comply with:

- Patient recruitment, follow up schedules and all requirements of the trial protocol
- Requirements of the Research Governance Framework
- Data collection requirements
- **No** trial imaging, recruitment sites will refer patients to designated imaging hub (see below)

In this protocol an ‘imaging hub’ refers to a hospital where certain trial-related activities are conducted.

Imaging hubs must be able to comply with:

- Patient recruitment (where applicable), follow up schedules (where applicable) and all requirements of the trial protocol
- Requirements of the Research Governance Framework
- Data collection requirements
- Trial imaging

4.1.1. Selection of Principal Investigator and other investigators at sites

Sites must have an appropriate Principal Investigator (PI), i.e. a health care professional authorised by the site and ethics committee, to lead and coordinate the work of the trial on behalf of the site. Other investigators at site wishing to participate in the trial must be trained and approved by the PI. All investigators must be medical doctors and have experience of diagnosing, staging or treating colorectal cancer.

4.1.2. Training requirements for site staff

All site staff must be appropriately qualified by education, training and experience to perform the trial related duties allocated to them, which must be recorded on the site delegation log.

CVs for all staff must be kept up to date, with signed and dated and copies held in the Investigator Site File (ISF). An up to date, signed copy of the CV for the PI must be forwarded to UCL CTC upon request.

All staff involved in the trial must receive GCP training which is relevant to their role and responsibilities within the trial. The frequency of repeat training may be dictated by the requirements of their employing institution, or 2 yearly where the institution has no policy, and more frequently when there have been updates to the legal or regulatory requirements for the conduct of clinical trials.

For peer review only - http://bmjopen.bmj.com/site/about/guidelines.xhtml
For peer review only

4.2. Site initiation and activation

4.2.1. Site initiation

Before a site is activated, the UCL CTC trial team will arrange a site initiation with the site which the PI and site research team must attend. The site will be trained in the day to day management of the trial, and essential documentation required for the trial will be checked.

Site initiation will be performed for each site by site visit or teleconference.

4.2.2. Required documentation

The following documentation must be submitted by the site to UCL CTC prior to a site being activated by UCL CTC trial team:

- Trial specific Declaration of Participation/Site Registration Form (identifying relevant local staff)
- All relevant institutional approvals (e.g. local NHS permission)
- A completed site delegation log that is initialled and dated by the PI
- A copy of the PI's current CV that is signed and dated

In addition, the following agreements must be in place:

- A signed Clinical Trial Site Agreement (CTSA) between the Sponsor and the relevant institution (usually a NHS Trust)

4.2.3. Site activation letter

Once the UCL CTC trial team has received all required documentation and the site has been initiated, a site activation letter will be issued to the PI. **Sites may not begin to approach patients until the site activation letter has been issued.**

Once the site has been activated by UCL CTC, the PI is responsible for ensuring:

- adherence to the most recent version of the protocol;
- all relevant site staff are trained in the protocol requirements;
- appropriate recruitment and medical care of patients in the trial;
- timely completion and return of CRFs (including assessment of all adverse events).

For peer review only - http://bmjopen.bmj.com/site/about/guidelines.xhtml

5. Informed consent

Sites are responsible for assessing a patient's capacity to give informed consent.

Sites must ensure that all patients have been given the current approved version of the patient information sheet, are fully informed about the trial and have confirmed their willingness to take part in the trial by signing the current approved consent form.

Sites must assess a patient's ability to understand verbal and written information in English and whether or not an interpreter would be required to ensure fully informed consent. If a patient requires an interpreter and none is available, the patient should not be considered for the trial.

The PI, or, where delegated by the PI, other appropriately trained site staff, are required to provide a full explanation of the trial to each patient prior to trial entry. During these discussions, the current approved patient information sheet for the trial should be discussed with the patient. A minimum of twenty four hours before the WB-MRI must be allowed for the patient to consider and discuss participation in the trial.

The consent process has been left deliberately flexible in order to accommodate the needs of individual patients and variations in Site requirements. The flowchart below describes three different options for obtaining written informed consent from patients, each of which ensures that patients have at least 24 hours to consider their participation in the trial before any trial related activities take place.

Streamline Recruitment Pathways/Consent Process

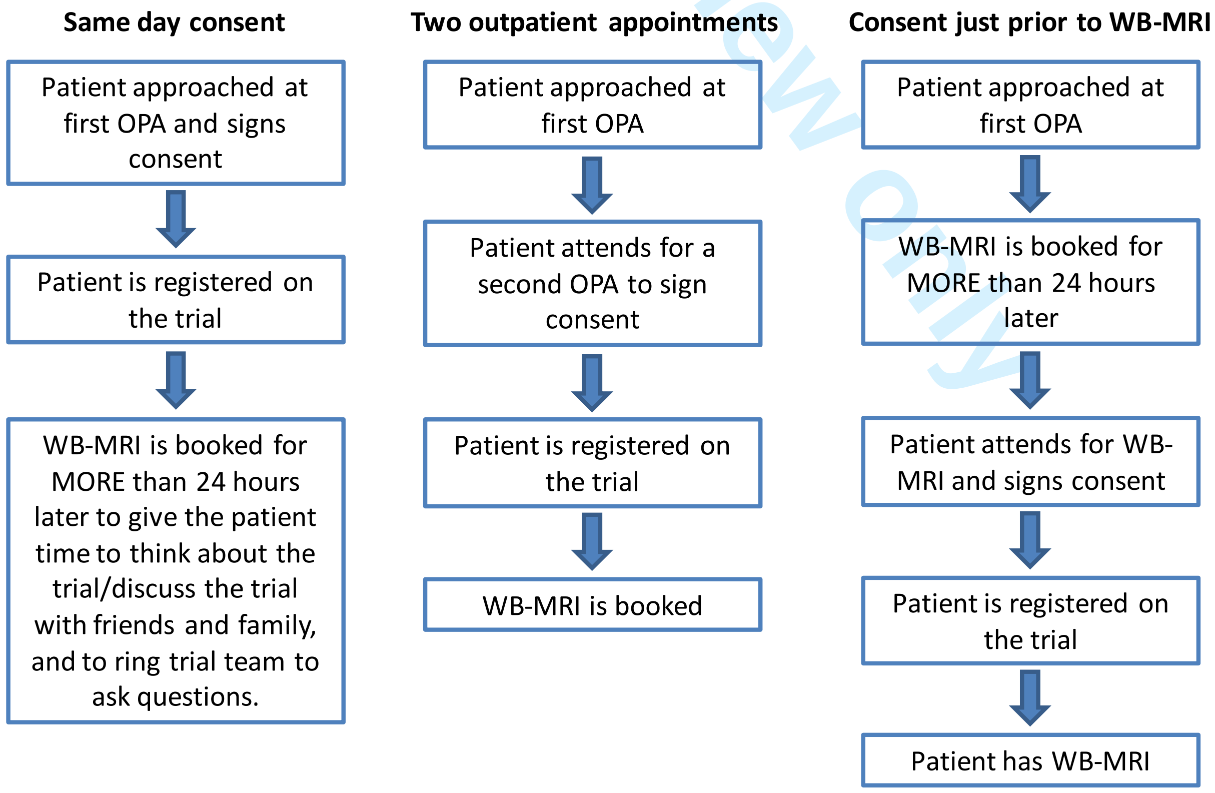

For peer review only - http://bmjopen.bmj.com/site/about/guidelines.xhtml

See Appendix 2 for a more detailed example of the consent process.

Written informed consent on the current approved version of the consent form for the trial must be obtained before any trial-specific procedures are conducted. The discussion and consent process must be documented in the patient notes.

Site staff are responsible for:

- checking that the correct (current approved) version of the patient information sheet and consent form are used;
- checking that information on the consent form is complete and legible;
- checking that the patient has completed/initialled all relevant sections and signed and dated the form;
- checking that an appropriate member of staff has countersigned and dated the consent form to confirm that they provided information to the patient;
- checking that an appropriate member of staff has made dated entries in the patient's medical notes relating to the informed consent process (i.e. information given, consent signed etc.);
- Following registration:
    - adding the patient trial number to all copies of the consent form, which should be filed in the patient's medical notes and investigator site file.
    - giving the patient a copy of their signed consent form and patient information sheet.

The right of the patient to refuse to participate in the trial without giving reasons must be respected. All patients are free to withdraw at any time. Also refer to section 13.0 (Withdrawal of patients).

For peer review only - http://bmjopen.bmj.com/site/about/guidelines.xhtml

6. Selection of Patients

6.1. Pre-registration Evaluation

The following assessments or procedures are required to evaluate the suitability of patients for the trial:

- Suspicion of colorectal cancer defined as: Presence of a mass highly suspicious for colorectal cancer on endoscopy, barium enema, CT colonography or other imaging which triggers staging investigations
- No contraindications to MRI (e.g. cardiac pacemaker, severe claustrophobia, inability to lie flat)

6.2. Screening Log

A screening log must be maintained by the site and kept in the Investigator Site File. This must record all potentially eligible patients approached about the trial and the reasons why they were not registered in the trial if this is the case. The log must be sent to UCL CTC in line with the monitoring plan and as requested, with patient identifiers removed prior to sending.

6.3. Multi Disciplinary Team (MDT) Meeting Log

An anonymous record of the number of patients with a new diagnosis of colorectal cancer discussed in the MDT meeting, with their attributed cancer stage (TNM or Dukes) will be submitted annually to UCL CTC. This will be used to assess whether the group of patients recruited to the trial is representative of the general population of newly diagnosed colorectal cancer patients seen at Sites.

6.4. Patient Eligibility

There will be no exception to the eligibility requirements at the time of registration. Queries in relation to the eligibility criteria must be addressed prior to calling/faxing for registration. Patients are eligible for the trial if all the inclusion criteria are met and none of the exclusion criteria applies.

6.4.1. Patient Inclusion Criteria

- Adult patients (18 or over) with histologically proven or suspected colorectal cancer referred for staging.
- Suspicion of colorectal cancer defined as: Presence of a mass highly suspicious for colorectal cancer on endoscopy, barium enema, CT colonography or other imaging which triggers staging investigations.
- Patient must have given written informed consent and be willing to comply with the protocol intervention and follow up.

For peer review only - http://bmjopen.bmj.com/site/about/guidelines.xhtml

For peer review only

6.4.2. Exclusion Criteria

- Any psychiatric or other disorder likely to impact on informed consent.
- Evidence of severe or uncontrolled systemic disease which make it undesirable for the patient to participate in the trial.
- Pregnancy.
- Contraindications to MRI (e.g. cardiac pacemaker, severe claustrophobia, inability to lie flat).
- Polyp cancer (because metastatic disease in these patients is vanishingly rare).

6.4.3. Pregnancy and Birth Control

MRI poses a theoretical risk to the foetus, particularly in the first trimester due to local acoustic and heating effects. However the risk is generally deemed very small and significantly less than the risk of ionising radiation imparted by PET-CT and CT (standard imaging investigations).

A woman of childbearing potential (WCBP) is a sexually mature woman (i.e. any female who has ever experienced menstrual bleeding) and who has not undergone a hysterectomy or who has not been postmenopausal for 24 consecutive months (i.e. who has had menses at any time in the preceding 24 consecutive months). Patients will be excluded if they are pregnant.

There is no requirement for additional contraceptive advice to patients over and above that routinely given as part of their routine clinical care given their diagnoses of colorectal cancer.

The need to perform a pregnancy test in WCBP will be decided as part of the patient's routine clinical care given the risk to pregnancy from standard staging investigations and subsequent treatment. There is no requirement to perform a pregnancy test purely because of recruitment to the trial if this would not have been performed as part of standard clinical care.

For peer review only - http://bmjopen.bmj.com/site/about/guidelines.xhtml

7. Registration Procedures

7.1. Registration

Patient registration will be undertaken centrally at UCL CTC and this must be performed prior to commencement of any trial intervention. Registration can be performed via telephone or fax.

Following pre-registration evaluations (as detailed in section 6.1), confirmation of eligibility and consent of a patient at a site, the registration form must be fully completed prior to faxing or telephoning UCL CTC. The eligibility criteria will be reviewed during the registration telephone call using the same form at UCL CTC.

A trial number will be assigned for the patient during the call and must be recorded at site by the caller. If desired, the site may fax a copy of the completed registration form to UCL CTC and the faxed registration from will be used it confirm patient eligibility at UCL CTC.

UCL CTC will email confirmation of the patient's inclusion in the trial and their trial number to the main contact, PI and Imaging Hub contact. If requested, a fax confirmation may also be sent. Case report forms will be emailed to the main contact at site.

Registration telephone number:	+44 (0)20 7679 9880
Registration fax number:	+44 (0)20 7679 9871
UCL CTC Office hours:	09:00 to 17:00 Monday to Friday

7.2. Registration Packs

Once a patient has consented to take part in the trial they should be provided with a registration pack to include the following:

- A copy of their signed consent form and patient information sheet
- An expenses claim form (to claim travel expenses to and from WB-MRI appointment)
- Pre-paid return envelope

7.3. Booking Whole Body MRI

Once a patient has been registered it is the responsibility of the recruiting site to contact their designated imaging hub to make arrangements for the WB-MRI. Each recruitment site will be provided with a contact number at their designated imaging hub. Recruitment sites will approach consecutive potentially eligible patients until the number of recruited patients fills the MRI capacity of the imaging hub.

For peer review only - http://bmjopen.bmj.com/site/about/guidelines.xhtml

It is possible recruitment potential at recruitment sites will be greater than the WB-MRI capacity at each central imaging hub. If a patient is approached and registered for the trial but MRI capacity is such that the scan could not take place in a timely fashion, a decision to withdraw the patient will be made by the recruitment site and the patient will then be informed. This possibility is explained to patients in the patient information sheet.

Because of the need to perform WB-MRI in a timely fashion, it is anticipated recruitment sites will be requested to pause recruitment if WB-MRI waiting time exceeds 2 weeks (if WB-MRI is performed after conventional imaging is complete) or 3 weeks (if WB-MRI is to be performed concurrently with standard tests). There will be flexibility in these timings depending on the circumstances of individual patients and their anticipated time to complete standard staging.

For peer review only

For peer review only - http://bmjopen.bmj.com/site/about/guidelines.xhtml

8. Patient Management and Trial Procedures

8.1. Summary

8.1.1. Staging and Treatment Decisions

The following information is required for the trial to document staging and treatment decisions:

- Stage and treatment decision based on conventional imaging only (and the number, timing nature and findings of these investigations)
- Stage and theoretical treatment decision based on WB-MRI only (and any additional tests generated by the WB-MRI) only (and the number, timing nature and findings of additional tests generated)
- A final treatment decision incorporating all available tests.

Conventional Imaging

Trial patients will undergo the standard staging protocol employed at their institution as per usual clinical care pathways. See section 8.2.1 for further detail.

Whole Body MRI Imaging and MDT Reveal

Recruited patients will undergo a WB-MRI at one of the designated imaging hubs. Recruiting sites should contact their designated imaging hub to request a WB-MRI slot (see section 7.3). The WB-MRI should be performed either concurrently with the standard staging investigations, or no later than 3 weeks after the final standard staging investigation. Images will be uploaded at each hub to a secure central imaging server called 3Dnet™. If a language interpreter was required for a patient during the consent process, then an interpreter should be made available to the patient during the WB-MRI. The WB-MRI will be reported by designated radiologist(s) at each imaging hub who are blinded to the conventional imaging tests and other clinical information (other than the cancer diagnosis and location). A copy of the report will also be uploaded to 3Dnet™. See section 8.2.2 for further detail.

Once the MDT has made a treatment decision based on the conventional imaging, the MDT coordinator, research nurse or other designated individual will reveal the WB-MRI images and report on 3Dnet™ via an internet-enabled PC. The MDT will review the WB-MRI results and decide whether or not any additional tests are required or would have theoretically been required (if in fact the test was already performed as part of conventional staging) in addition to the WB-MRI. If any tests are required that have not already been performed as part of the standard staging, they should be performed and the patient reviewed again at the next MDT when the results are available. If no further tests are needed then the stage and theoretical treatment decision based on the WB-MRI (and any additional tests generated from the WB-MRI) must then be made by the MDT. The MDT must then decide a final treatment decision for the patient based on all imaging available. If referral to the liver MDT is indicated, the final treatment decision based on WB-MRI will be that made by the liver MDT after review of the WB-MRI and generated tests. See section 8.2.3 for further detail.

For peer review only - http://bmjopen.bmj.com/site/about/guidelines.xhtml

If a patient is due to start treatment before the next formal MDT meeting, an ad hoc mini MDT meeting may be scheduled outside of the standard MDT meeting. This should include the relevant multidisciplinary clinical team members who are appropriate to make the final treatment decision based on the specific imaging finding, according to usual standards of clinical care. A record will be kept of who is present during the ad hoc mini MDTs on the CRFs.

8.1.2. Patient experience Interviews and Questionnaires (Optional)

Interviews (Study 1)

A total of 25 patients have undergone interviews by a Health Psychologist pertaining to their experience of the cancer staging process. **Target accrual has now been reached for the interview part of the trial and recruitment to this is now closed.**

Questionnaires (Study 2)

Patient experience questionnaires from baseline and post-staging have been received for approximately 50 patients to assess patient experience and acceptability of WB-MRI and standard tests. **Target accrual has now been reached for the questionnaire part of the trial and recruitment to this is now closed.**

Discrete choice experiment (Study 3)

A discrete choice experiment (DCE) questionnaire will be administered to a subset of approximately 50 patients to elicit their preference for various attributes associated with conventional and WB-MRI staging pathways. The DCE questionnaire is optional and patients will be asked to consent to this on trial entry. Once a patient consents to the DCE they will be allocated a DCE questionnaire (out of a possible 2 DCE questionnaires) which will be posted by UCL CTC along with their Quality of Life questionnaire, patient diary and pre-paid envelope immediately after registration (baseline). UCL CTC will supply the details of any recruited patients who consented to complete DCE questionnaire to the Health psychologists who may contact them directly.

8.1.3. Quality of Life Questionnaires and Patient Diaries

Upon consent the recruiting site must inform the patient that they will receive an EQ-5D Quality of Life Questionnaire and Patient Diary by post from UCL CTC and they are to complete these and return them using the pre-paid envelope provided. The patient diary will cover a 3 month period and will collect information regarding the patient's Primary Health Care contacts, other health care contacts and also the medication they are currently taking. Patient diaries and EQ-5D questionnaires will be posted to all patients at consent and then at 3 monthly intervals for 12 months.

For peer review only - http://bmjopen.bmj.com/site/about/guidelines.xhtml

For peer review only

Streamline C

Table 1: Summary of patient management and trial activities

Activity	Protocol section	Timing							Responsible organisation/person
		Prior to registration	Immediately after registration	During staging	After staging (months after registration)				
					1	3	6	9	
Pre-registration evaluation & assessment of eligibility	6.1	X							Recruiting site
Consent	5.0	X							Recruiting site
Register patient	7.1	X							Recruiting site
Give patient registration pack (copy of PIS and consent, expenses claim form and pre-paid return envelope)	7.2		X						Recruiting site
Book WB-MRI scan & inform patient of appointment date	7.3		X						Recruiting site
WB-MRI	8.2.2			X					Imaging Hub
Upload WB-MRI scan & report	8.2.2			X					Imaging Hub
Post EQ-5D questionnaires	8.2.8		X			X	X	X	UCL CTC
Post patient resource diary	8.2.8		X			X	X	X	UCL CTC
Discrete choice experiment Questionnaire	8.2.10		X						UCL CTC

For peer review only

For peer review only - http://bmjopen.bmj.com/site/about/guidelines.xhtml

8.2. Detailed description

8.2.1. Standard care

Trial patients will undergo the conventional staging investigations employed at their recruiting institution according to local protocols and the requirements of their clinical care team.

All conventional investigations will be performed and interpreted by the usual radiologists and clinicians employed at the site of the investigations. Standard clinical reports will be made and all investigations (and their results) will be freely available on hospital Picture Archiving and Communications System (PACS), Radiology Information System (RIS) and Clinical Data Repository (CDR) systems as per usual clinical practice.

The type and date of investigations (e.g. CT scan, PET-CT, organ specific MRI, biopsy etc) will be recorded on CRFs, along with the presence and location of metastatic disease based on the radiological report.

8.2.2. Trial Imaging

Whole Body MRI (WB-MRI) protocol

Recruited patients will undergo a WB-MRI at their designated imaging hub site. The choice of MRI platform (i.e. manufacturer and Tesla (T) strength) will be decided by the local hub radiologist according to scanner availability and their usual practice. It is anticipated most MRIs will be performed at 1.5T. Exact imaging parameters will vary according to MRI platform but a minimum dataset of sequences will be acquired (full details given in appendix 3).

Patients with rectal cancer would normally undergo staging pelvic MRI, and the WB-MRI will be additional to this. Where possible (and according to site and patient preference), the WB-MRI and rectal staging MRI would be performed on the same day to reduce patient travel.

Timing

The WB-MRI will be performed either concurrently with the standard staging investigations, or no later than 3 weeks after their final standard staging investigation.

Blinding

To ensure the integrity of the trial, the WB-MRI must be reported by a radiologist blinded to the conventional imaging tests and other clinical information (other than the cancer diagnosis and site). Conversely the WB-MRI images and reports must not be available to either those radiologists reporting the standard staging investigations, nor those involved in direct patient care before the WB-MRI is revealed in the MDT meeting.

The un-anonymised WB-MRI images will therefore not be immediately sent to the Picture Archive and Communication System (PACS) at either the imaging hub or recruitment sites. Instead images will be uploaded at each hub to a secure central

For peer review only - http://bmjopen.bmj.com/site/about/guidelines.xhtml

imaging server (3Dnet™) provided by Biotronics3D. This solution allows easy upload of MRI datasets via standard internet connection. A PC based internet gateway will be installed in each imaging hub to facilitate automated transfer of WB-MRI from the scanner/workstation to 3Dnet™), and thereafter back to PACS to the appropriate time point after MDT revelation (see below).

Interpretation and reporting

The WB-MRI will be interpreted by designated radiologist(s) at each imaging hub who are expert in interpretation based on previous experience of reporting WB-MRI in cancer staging. As noted above, the radiologist will be blinded to the standard staging investigations performed on recruited patients.

Interpretation can be performed using the 3Dnet™ software, or stand alone workstation according to the preference of the radiologist.

Images will be analysed in the following order:

1) Diffusion and non contrast enhanced T1 images
2) Diffusion, non contrast enhanced T1 and T2 images
3) Diffusion, non contrast enhanced T1, T2 and contrast enhanced T1 images.

After viewing each sequence set (and before reviewing the next set), the radiologist will complete WB-MRI Imaging Booklet CRFs documenting the presence, location and size of metastatic disease, together with their diagnostic confidence on a scale of 1 to 6. Reporting time for each sequence set analysis (defined as the time required to interrogate the sequences to reach a diagnostic conclusion) will be recorded along with the technical quality of the MRI dataset.

The reporting radiologist will then produce a free text clinical report as per their usual clinical practice (using all available sequences and based on the TMN 7 staging guidance) for subsequent release to the clinical team. This report will contain information relating to the local T and N stage of the tumour, together with the presence, location, number and size of metastatic deposits, as well as important “incidental” findings, for example aortic aneurysm. The radiologist may express their level of confidence in reported findings as they would in normal clinical practice but a formal numerical score of confidence will not be provided as this would not mirror how radiological examinations are reported in standard clinical care. If the radiologist would usually recommend additional tests for equivocal findings, this will be included in the report so as to also mirror routine clinical practice. The definitions of T, N M stage will be based on conventional MRI criteria adopted by the radiologist in their usual clinical practice.

The free text report will be uploaded onto the 3Dnet™ software, and a copy stored as part of the patient’s medical record.

For peer review only - http://bmjopen.bmj.com/site/about/guidelines.xhtml

8.2.3. Release of Conventional and trial WB-MRI findings

MDT discussion of the first major treatment decision based on conventional imaging

WB-MRI images and reports will be withheld initially from the clinical care team and radiologists reporting standard imaging (to avoid bias) until patients have completed all conventional investigations and have been definitively staged such that a first major treatment decision has been made by the MDT based on these conventional tests. The first major treatment decision based on conventional imaging will be defined as:

- Referral for surgical excision of either the primary tumour and/or a metastatic site
- Instigation of definitive treatment using chemotherapy, radiotherapy or a combination of the two
- Decision to offer palliative/supportive care only
- Request for a highly invasive surgical staging procedure such as surgical mediastinal lymph node sampling (mediastinoscopy), video-assisted thoracoscopic surgery (VATS), or laparoscopy

In some instances the first major treatment decision will not be made by the colorectal MDT, for example the patient being referred to a liver MDT. In this case the treatment decisions of the liver MDT made according to the protocols described below will be used for the purposes of the trial.

If additional conventional tests are required to complete staging, the WB-MRI result will be withheld until they are completed, and the patient re-discussed with the findings of the additional test(s).

The nature, date and findings of all conventional investigations will be recorded by the MDT, and the TNM 7 stage recorded. The MDT/appropriate MDT personnel will define the nature of the first treatment decision based on conventional investigations in the MDT record and on the CRF.

Release of WB-MRI images and report

Once a stage and treatment decision has been made based on conventional imaging, the WB-MRI images (supplemented where possible with "screen shots" and annotations of relevant findings) and radiologist's report will be revealed to the MDT using the 3Dnet™ software on an internet enabled PC. If technical issues preclude use of 3Dnet™ in the MDT, presentation of a written copy of the WB-MRI report is permissible.

When the WB-MRI images and report are released, the MDT will discuss the images and decide:

1) *If the patient can be adequately staged based on WB-MRI alone:*

   In this case the MDT should state the patient's stage and theoretical treatment decision based on the WB-MRI alone.

Or:

For peer review only - http://bmjopen.bmj.com/site/about/guidelines.xhtml

2) *If any additional tests are required based on the WB-MRI alone for adequate staging* (for example for equivocal WB-MRI findings):
    a. For additional tests that would have theoretically been requested based on the WB-MRI alone but have already been performed as part of the standard imaging, the results can be used by the MDT. The MDT personnel will state the patient's stage and theoretical treatment decision based on the WB-MRI and those additional tests that would have theoretically been generated.
    b. If the additional tests generated by WB-MRI have not already been performed as part of standard care and are deemed necessary in the opinion of the MDT these will be undertaken according to standard clinical care. After completion of the additional test(s), the patient will be re-discussed by the MDT and a final treatment decision made based on the WB-MRI and the additional test(s) undertaken.

If, following revelation of the findings of the WB-MRI (and any additional tests it generates), the colorectal MDT request review by the liver MDT, (which had not been the case based on standard imaging), for the purposes of the trial the final treatment decision based on WB-MRI will be that made by the liver MDT after review of the WB-MRI and generated tests. The fact that following WB-MRI a referral to the liver MDT is made will be recorded.

8.2.4. Final treatment plan

Once a stage and treatment decision has been made based on conventional imaging alone and also a stage and theoretical treatment decision made based on the WB-MRI (and any additional tests it generates) alone a final treatment plan can be made by the MDT. The final treatment plan for the patient will be based on all available information (including the WB-MRI). The MDT radiologist(s) will be at liberty to review the WB-MRI data after the WB-MRI has been revealed in order to review its findings and feedback to the clinical care team.

The completed MDT CRF will thus list:

- Stage and treatment decision based on conventional investigations (and the number, timing nature and findings of these investigations).
- Stage and theoretical treatment decision based on WB-MRI (and the number, timing nature and findings of additional tests generated).
- Final treatment decision incorporating all available tests.

Once the WB-MRI images and report have been revealed to the MDT according to trial protocol, they can be released onto the relevant hospital PACS server using the 3Dnet™ gateway described above or other IT solution (e.g. image exchange portal) and be made freely available to those providing subsequent patient care, as per usual clinical practice.

For peer review only - http://bmjopen.bmj.com/site/about/guidelines.xhtml

8.2.5. Early release of WB-MRI findings

Any requests for early WB-MRI release must be made to the site PI, who will discuss the request with the lead clinician who is responsible for the patient's clinical care (if the request did not originate from the lead clinician). After discussion if it is decided by the PI, that release of the WB-MRI images and report is necessary, the PI (or a designated member of the trial team) will inform UCL CTC. The WB-MRI result should only be released early in the event of an emergency situation in which the patient is unfit to undergo additional investigations (including MRI) which would have normally been performed were the patient not in the Streamline C trial, and the WB-MRI may have a direct effect on immediate patient care. In the event of early release, the patient will be replaced, although the findings of the WB-MRI and conventional staging (performed up to the time of early MRI release) will be collated by UCL CTC on the standard trial CRFs for subsequent reporting in the trial publications.

Early release for important clinical findings

The time between the patient's diagnosis and the WB-MRI reveal is typically less than 4 weeks at the proposed recruitment sites. The majority of findings on WB-MRI will have no direct impact on patient care during this time. However should WB-MRI reveal a serious finding which could have an immediate impact on direct patient care before the MDT, the reporting radiologist will contact the patient's clinician to discuss the finding and a decision will be made as to whether the results should be revealed early to all members of the clinical team (based on review of standard tests already performed which may also have detected the finding). Specific findings which will trigger this review are:

- Impending spinal cord compression
- Deep vein thrombosis or pulmonary embolism
- Brain metastasis with significant mass effect requiring immediate treatment

It is unlikely the patient would be asymptomatic in any of these scenarios, and it is probable additional imaging tests would have already been requested as part of usual clinical care.

Early release for urgent patient management

Although unlikely, it is possible there may be a need to access the WB-MRI report urgently before its release to the MDT, for example if a recruited patient presents to hospital with collapse and knowledge of the WB-MRI findings would potentially change patient management. As noted above, imaging hubs will keep a copy of the free text report of the WB-MRI and this will be made available if requested by clinical teams in this scenario.

8.2.6. Time to full staging

Standard pathway

The total time required to fully stage the patient using standard imaging pathways will be calculated. The start of the staging process will be defined as the date of request of the first staging investigation following a proven or assumed diagnosis of colorectal

For peer review only - http://bmjopen.bmj.com/site/about/guidelines.xhtml

cancer (for example date of requested for CT chest, abdomen and pelvis after colonoscopic diagnosis of likely malignant tumour). The completion of staging will be defined as the date of the MDT/appropriate MDT personnel made the first major treatment decision based on the standard imaging.

WB-MRI

Because revelation of the result of WB-MRI is deferred until after standard staging is complete, actual measurement of the time to full staging using WB-MRI will not be possible. The theoretical time to complete staging using WB-MRI will be thus modelled taking into consideration the time from recruitment date to the date of the WB-MRI, plus the number and type of additional staging investigations generated by WB-MRI.

8.2.7. Patient follow up

Patients will be followed for a period of 12 months from the date of recruitment. Follow up will not require any trial specific patient visits to the recruiting site, and follow up CRFs can be completed using hospital databases or patient notes.

For all recruited patients the final TN tumour stage, according to the 7th classification, will be recorded based on histopathological analysis of surgical specimens (if surgery is performed). The results of any biopsy procedure undertaken over the 12 months will also be recorded. The date, nature and findings of follow up imaging investigations will be recorded for each recruited patient, in particular the presence or absence of metastasis.

The date and cause of patient death and post mortem findings (if performed) will also be recorded.

MDT records and hospital data repositories will be used to collate this data, and data collection will be coordinated by designated individuals at each recruitment site, aided by UCL CTC.

8.2.8. Patient diaries and EQ-5D Quality of Life questionnaires

All patients will receive a Diary and EQ-5D Quality of Life questionnaire every 3 months which will be posted by UCL CTC. Upon consent the recruiting site must inform the patient that they will receive an EQ-5D questionnaire and Diary through the post from UCL CTC and they are to complete these and return them using the pre-paid envelope provided. The patient diary will cover a 3 month period and will collect information regarding the patient's Primary Health Care contacts, other health care contacts and also the medication they are currently taking. Patient diaries and EQ-5D questionnaires will be posted to all patients at consent and then at 3 monthly intervals for 12 months.

8.2.9. Final reference standard for tumour stage (Consensus Meetings)

Multi-disciplinary consensus panel review is standard methodology for diagnostic test accuracy studies where an independent reference standard is impossible because of incorporation bias. Consensus panels will convene annually to derive the reference standard for tumour stage at diagnosis for recruited patients completing the first 12 month follow up (i.e. a panel at the end of year 2 will consider patients recruited in

For peer review only - http://bmjopen.bmj.com/site/about/guidelines.xhtml
For peer review only

year 1, a panel at the end of year 3 will consider patient recruited in year 2 etc.). The panels will consider all available clinical information including the results of all original staging investigations, WB-MRI, histopathology (surgical resection and biopsies), follow up imaging and post-mortem reports (where available) and MDT records. UCL CTC will coordinate collation of these data via submitted CRFs over the preceding 12 months for presentation to the panel. Each imaging hub or recruitment site will host a consensus panel to consider patients undergoing WB-MRI (or recruited) at its site (so all imaging studies are available for review on local PACS systems if required). Each panel will consist of at least an oncologist, and/or a colorectal surgeon, and 3 radiologists, 1 external to the imaging hub and 2 internal: 1 with specific expertise in WB-MRI and 1 with expertise in PET (a single radiologist is acceptable if they have expertise in WB-MRI and PET). The panel will have access to a histopathologist if required. Each panel will adjudicate on the TMN stage of the cancer at diagnosis, including the organ specific sites and burden of metastatic spread.

The definition of the presence or absence of metastasis will be assigned for each organ on a designated CRF. The designation will be made in consensus by all on the panel. Consideration will be made to histology in all biopsied lesions. In the absence of histological proof of metastasis, metastatic disease will be assumed if new lesions appear during the 12 month follow up with imaging characteristics compatible with metastasis and no alternative explanation, or if lesions with characteristics compatible with metastasis which either grow or shrink (on therapy). Lesions identified which remain stable over the 12 month follow up period will assumed not to be metastatic unless there are specific circumstances considered by the panel that indicate malignancy (e.g. change in lesion morphology with treatment).

All “new” sites of metastatic disease diagnosed in the 12 month follow up will be assessed by the panel to see if they were visible in retrospect at diagnosis. Although the primary analysis by the original reporting radiologist will be that used to define test accuracy, this retrospective review will allow definition of the rates of perceptual error.

For patients in whom the primary tumour has been completely removed within 3 months of diagnosis, all new metastatic sites will be assumed to have been present at diagnosis for the purposes of calculating test sensitivity.

If the primary tumour is left in situ for more than 3 months (or there is incomplete removal), any new diagnosed sites of metastatic disease will be assumed to have been present at diagnosis if identified within 6 months of diagnosis of the primary tumour. If they are diagnosed beyond 6 months of diagnosis, and there is no evidence of their presence on retrospective review of all staging investigations, they will be assumed to be new disease not present at diagnosis.

If patients with tumours left in situ do not undergo any imaging capable of detecting metastatic disease within 6 months of diagnosis of the primary tumour (other than initial staging tests) and imaging beyond this identifies metastatic disease not visible in retrospect on any trial imaging, the consensus panel will opine if the disease was likely present at diagnosis, based on its location, size and imaging characteristics.

If a patient dies before the 12 month follow up, the panel will review all available imaging, histopathology and clinical course prior to death and in consensus state if a confident diagnosis of the presence or absence of metastatic disease can be made (for example imaging characteristics compatible with metastasis and no alternative

For peer review only - http://bmjopen.bmj.com/site/about/guidelines.xhtml

explanation, or if lesions with characteristics compatible with metastasis which either grow or shrink (on therapy). If this judgement cannot be made with confidence (for example if the patient has equivocal lesions on staging investigations and no further follow up), these patients will not be excluded but multiple imputation will be used to account for missing data. A sensitivity analysis for the primary outcome, where only patients with complete data are analysed, will exclude these patients.

8.2.10. Health psychology assessment

Study 1: Patient interviews (approximate n=25)

Target accrual has now been reached for the patient experience interview part of the trial and so recruitment to this part is now closed. Individual in-depth interviews were conducted by a health psychologist pertaining to their experience of the cancer staging process. The aim of the interviews is to determine patients' experience of WB-MRI, standard tests and those tests generated by WB-MRI. We collected simple demographic data from participants in relation to gender, age and educational level as such factors may influence experience and preferences, and this data will enable us to determine whether the sample of people who are interviewed are representative of the sample of people who also participate in studies 2 and 3 (see below). The interviews assessed which aspects of testing caused patients physical or psychological stress (e.g. number of tests/hospital visits, test attributes (i.e. physical experiences of the tests such as claustrophobia, need to lie still, scanner noise etc.), total length of time taken, additional tests generated by initial scan findings) and elicited any factors patients felt would have made staging easier for them.

The interviews provided in-depth qualitative data about the experience of cancer staging and in particular under-going WB-MRI, and has informed the modification of a questionnaire (study 2) designed to assess patient acceptability and experience of WB-MRI compared to standard tests, and also has informed the design of a discrete choice experiment (study 3).

Study 2: Patient questionnaires (approximate n=50)

Target accrual has now been reached for the patient experience questionnaire part of the trial and so recruitment to this part is now closed. Follow up questionnaires will continue to be posted until the last patient's month 6 time point occurs. A questionnaire was developed and sent to consenting patients to assess their experience and acceptability of WB-MRI and standard tests (e.g. PET-CT). Final design was informed by analysis of the qualitative data collected in study 1 (See above). Patient Experience Questionnaire content is outlined in Table 3 and includes patients' views about test preparation (e.g. fasting), and experience of the scan itself (e.g. discomfort, claustrophobia, fear etc.). Patients were also asked to rate the importance of various attributes associated with competing staging pathways (e.g. waiting time from diagnosis to treatment, total test number, test accuracy etc.). This rating identified those attributes considered most important by patients, which has been included in the discrete choice experiment (study 3).

Patients were given the opportunity to opt in to completing the questionnaires when they were recruited into the trial. Questionnaires, together with the positive and negative affect schedule (PANAS) and the General Health Questionnaire (GHQ-12) were administered during and after staging pathways, and at 3 and 6 months later to

For peer review only - http://bmjopen.bmj.com/site/about/guidelines.xhtml

examine patient test preferences once they are aware of their cancer stage and knew which of the tests they received were more accurate as well as the burden placed on them by any extra tests they needed as a result of WB-MRI.

Patients were posted the baseline questionnaire along with pre-paid reply envelope at the time they were recruited into the trial. The questionnaire was worded to take into account the fact that some patients had not yet had any staging investigations. Patients were posted the post-staging and 3 and 6 month follow up questionnaires along with pre-paid envelopes at the relevant time points.

Study 3: Discrete choice experiment (approximate n=50)

Discrete choice experiments (DCE) can elicit preferences for different types of health care provision [18] by estimating the relative importance of different attributes, and the trade-offs between them [19]. WB-MRI and conventional staging pathways differ in associated attributes, not only related to physical experience but also rate of adverse events, time to diagnosis, overall accuracy etc. The most important attributes have been identified in studies 1 and 2 and appropriate levels assigned to each based on accumulating data from the trial, together with appropriate literature review. A DCE questionnaire has been developed whereby patients state their preference between two choices, with each choice containing different levels of the identified attributes.

The DCE questionnaire is optional and patients will be asked to consent to complete the questionnaire on trial entry. Once a patient consents to the DCE they will be allocated a DCE questionnaire (out of a possible 2 DCE questionnaires) which will be posted by UCL CTC immediately after registration (baseline) along with their QoL questionnaire, patient diary and pre-paid envelope. UCL CTC will supply the details of any recruited patients which consented to complete the DCE questionnaire to the Health psychologists who may contact them directly.

For peer review only

For peer review only - http://bmjopen.bmj.com/site/about/guidelines.xhtml

Streamline C

Table 3: Questionnaire schedule. Questionnaires will be posted to patients by UCL CTC.

	Baseline (peri-staging)	Months after registration		
		1	3	6
Demographic measures	✓			
Co-morbidities	✓			
Self-rated health	✓			
Symptoms of bowel cancer	✓			
Concern for future consequences	✓			
Which staging tests have had	✓	✓		
Perceived importance of test attributes	✓	✓		
Perceived acceptability of different tests	✓	✓		
Least acceptable aspects of different tests	✓	✓		
Agreement to having tests again (short and long-term)	✓	✓		
Single test choice	✓	✓		
Test recovery time	✓	✓		
Patient experience of WB-MRI and standard tests	✓	✓		
Perceived efficacy of WB-MRI and standard tests		✓		
Difficulties with completing scan (WB-MRI and standard tests)		✓		
Ranked attribute preferences	✓	✓		
Feelings about number of tests			✓	✓
EQ-5D	✓		✓	✓
PANAS	✓	✓	✓	✓
GHQ-12	✓	✓	✓	✓

For peer review only

For peer review only - http://bmjopen.bmj.com/site/about/guidelines.xhtml

8.2.11. Cost Effectiveness Assessment

Overview

We will undertake a detailed analysis of the cost and the cost-effectiveness of WB-MRI versus standard NICE-approved staging algorithms. The analysis will conform to accepted economic evaluation methods [17]. All costs will be assessed from the perspective of the NHS and personal social services (PSS).

Making comparisons between WB-MRI versus standard staging algorithms

The care pathway for colorectal cancer patients can both be divided into two stages, the *treatment decision pathway* and the *subsequent disease pathway*. The former includes the time from diagnosis to treatment decision by the MDT; the latter includes the time period following the treatment decision.

The treatment decision pathway will be different between WB-MRI and standard staging algorithms, yielding different costs and potentially different treatment decisions. In patients for whom the treatment decision with WB-MRI is the same as that with conventional staging algorithms, the subsequent disease pathways will be the same. Where the treatment decision with WB-MRI is different, the disease pathway will be different, yielding potentially different costs and health outcomes.

If in the patients studied the concordance between the treatment decisions associated with WB-MRI and conventional staging algorithms is high, then the economic analysis can focus on the cost of the treatment decision pathways only because the disease pathways will be no different. In this case the cost-effectiveness of WB-MRI versus conventional staging algorithms depends only on the incremental cost (positive or negative) of WB-MRI versus standard staging algorithms in the treatment decision pathway.

Conversely, if the concordance between the treatment decisions is low, then the economic analysis ought to focus on both the treatment decision pathways and the subsequent disease pathways because both of these will vary between WB-MRI and conventional staging algorithms. In this case the cost-effectiveness of WB-MRI depends on the incremental cost of the WB-MRI versus standard staging algorithms in the treatment decision pathway plus the incremental costs and health benefits of the disease pathway.

The precise nature of the economic analysis will therefore depend on the degree of concordance between treatment decisions provoked by WB-MRI versus conventional staging.

Discordance of major treatment decisions

Discordance will be defined when the first major treatment decision differs between WB-MRI and standard investigations in greater than 10% of patients. Concordance will be defined as the absence of discordance. For colorectal patients a 4x4 table will be used to summarise major differences between 4 main treatment decisions (surgery for the primary but no chemotherapy, surgery for the primary and chemotherapy (and/or radiotherapy), chemotherapy (and/or radiotherapy) without surgery, and surgical metastectomy with or without chemotherapy) for colorectal cancer).

For peer review only - http://bmjopen.bmj.com/site/about/guidelines.xhtml

Streamline C

Scenario 1: there is concordance between the treatment decisions associated with WB-MRI and standard staging

In this case, the cost components included in the analysis will be:

- Conventional tests for staging of disease among diagnosed patients (e.g., CT chest, pelvis and abdomen and MRI pelvis for colon cancer; plus additional tests as indicated by the conventional staging algorithm);
- Costs of treating adverse events associated with staging tests;
- WB-MRI, plus additional tests generated by WB-MRI;
- MDT meetings.

The volume of resource use for each cost component will be measured directly in the trial from treatment decisions recorded in MDT reports, based first on conventional staging alone and then based on WB-MRI alone, and on patient records included in the trial. Unit costs will be taken from standard published sources. Since the two algorithms yield the same treatment decisions cost-effectiveness depends on the incremental cost of WB-MRI versus conventional staging algorithms in the treatment decision pathway (i.e., formally this is a cost-minimisation analysis).

Scenario 2: there is discordance between the treatment decisions associated with WB-MRI and standard staging

In this case, cost-effectiveness depends on the incremental cost (positive or negative) of the treatment decision pathway and disease pathway associated with WB-MRI versus conventional staging and the incremental health benefits (positive or negative). We will calculate cost-effectiveness in terms of the incremental cost per quality-adjusted life year (QALY) gained using one year and lifetime time horizons.

For the analysis based on the one-year time horizon, the trial will provide information on the treatment decisions arising from WB-MRI and standard staging and follow up data for the first year. We will collect these data from three sources.

Firstly, for all patients in the trial we will collect the resource use data for the main drivers of hospital costs using a trial specific CRF. This will collect resource use data on the following cost components for each patient:

- Imaging investigations
- Chemotherapy
- Radiotherapy
- Surgery and biopsies
- Outpatient visits
- Inpatient stays
- Day cases

These data will be recorded prospectively based mainly on MDT consensus meeting recommendations and on patient records (for major changes in treatment). Unit costs will be taken from standard published sources and applied to the resource use data, allowing us to cost the care received by each patient.

In addition, from this source we will collect data on whether or not the patient died during the follow up period, and if they died, the date of death.

For peer review only - http://bmjopen.bmj.com/site/about/guidelines.xhtml

Secondly, for all patients in the trial we will prospectively collect resource use data using patient diaries. This will supplement the above data which may not provide a complete picture of hospital resource use, plus it will allow us to collect data on primary and community care contacts. These will record resource use data on the following cost components for each patient:

- Medications taken
- Day cases
- GP contacts
- Practice and community nurse contacts
- A&E Visits
- Any other primary care or community care contacts related to cancer

The diaries will be posted out by UCL CTC at baseline, 3, 6, and 9 months and patients will be asked to complete them for the following 3 month period.

In addition we will also collect data on health-related quality of life score, measured according to the EQ-5D (www.euroqol.org), which will be measured at baseline and at 3, 6, 9 and 12 months for all surviving patients.

UCL CTC will contact sites just prior to sending out any post registration diaries or questionnaires to check the status of the patient to ensure none are sent inappropriately to deceased patients.

Patients who are found not to have cancer or are withdrawn for other reasons (see section 13), will not be sent diaries, quality of life or patient experience questionnaires to complete. Thirdly, the two sources of resource use data described above will be supplemented by retrospective review of resource use (including imaging, outpatient visits, inpatient stays and medication use) for the 12 months post diagnosis of up to 150 patients diagnosed with colorectal cancer at UCLH. This retrospective review will be carried out by a member of the clinical team and according to guidance provided by the UCLH Caldicott guardian. All collated retrospective data will be fully anonymised before sharing with other trial researchers outside the clinical care team.

8.2.12. Nested substudies

1) Diagnostic performance of limited WB-MRI protocols based on diffusion and T1 weighted imaging only, and to assess the incremental benefit of intravenous gadolinium contrast enhancement.

   As noted above, WB-MRI will be analysed using a locked sequential viewing paradigm such that the diagnostic accuracy of Diffusion and non contrast enhanced T1 images, Diffusion, non contrast enhanced T1 and T2 images, and Diffusion, non contrast enhanced T1, T2 and contrast enhanced T1 images can be calculated.

2) Effect of radiologists' diagnostic confidence on the accuracy of WB-MRI.

   Radiologists will indicate their level of confidence for the presence or absence of metastasis on a 1-6 scale on the CRF recording their interpretation of the WB-MRI.

3) Inter-observer variation in WB-MRI interpretation.

For peer review only - http://bmjopen.bmj.com/site/about/guidelines.xhtml

Streamline C

Each hub radiologist (n=6) will interpret a sample of 25 WB-MRI datasets selected at random from the other imaging hubs to define inter-observer variation in the reported presence absence of metastatic disease.

4) The collated WB-MRI datasets and 12 month patient follow up data collated as part of the trial protocol provides opportunities to retrospectively mine the data for additional diagnostic indices and prognostic markers (See appendix 4). The following or similar may be quantified in all or a subset of whole body MRI datasets:

- Body fat (organ, visceral and whole body),
- MRI signal from the primary tumour and metastatic sites, notably diffusion weighted signal, apparent diffusion coefficient, T1 (including fat and water components), T2 signal, Contrast enhancement, and textural analysis (for heterogeneity) of the primary and metastatic deposits on the various sequences.

For peer review only

For peer review only - http://bmjopen.bmj.com/site/about/guidelines.xhtml

9. Data Management Guidelines

Data will be collected from sites on version controlled case report forms (CRFs) designed for the trial and supplied by UCL CTC. Data entered onto CRFs must reflect source data at site. Some data will be recorded directly on the CRFs (i.e. no prior written or electronic record of data), and it will be considered to be source data. This will be made clear in the CRF guidance.

Where supporting documentation (e.g. autopsy reports, pathology reports, CT scan images etc.) is being submitted to UCL CTC, the patient's trial number must be clearly indicated on all material and any patient identifiers removed/blacked out prior to sending to maintain confidentiality.

9.1. Completing Case Report Forms

All CRFs must be completed and signed by staff who are listed on the site staff delegation log and authorised by the PI to perform this duty. The PI is responsible for the accuracy of all data reported in the CRF.

All entries must be clear, legible and written in ball point pen. The use of abbreviations and acronyms must be avoided.

Any corrections made to a CRF at site must be made by drawing a single line through the incorrect item ensuring that the previous entry is not obscured. Each correction must be dated and initialled. Correction fluid must not be used.

The use of abbreviations and acronyms must be avoided.

Once completed the original CRFs must be sent to UCL CTC and a copy kept at site.

9.2. Missing Data

To avoid the need for unnecessary data queries CRFs must be checked at site to ensure there are no blank fields before sending to UCL CTC. When data are unavailable because a measure has not been taken or test not performed, enter "ND" for not done. If an item was not required at the particular time the form relates to, enter "NA" for not applicable. When data are unknown enter the value "NK" (only use if every effort has been made to obtain the data).

9.3. Timelines for data return

CRFs must be completed at site and returned to UCL CTC as soon as possible and within 4 weeks of scheduled time point. For further details, see CRF instructions.

Sites who persistently do not return data within the required timelines may be suspended from recruiting further patients into the trial by UCL CTC and subjected to a 'for cause' monitoring visit. See section 12.2 ('For cause' on-site monitoring) for details.

For peer review only - http://bmjopen.bmj.com/site/about/guidelines.xhtml

9.4. Data Queries

Data arriving at UCL CTC will be checked for legibility, completeness, accuracy and consistency, including checks for missing or unusual values. Query reports will be sent to the data contact at site. Further guidance on how data contacts should respond to Data Queries can be found on the Query Reports.

For peer review only

For peer review only - http://bmjopen.bmj.com/site/about/guidelines.xhtml

10. Safety Reporting

Clinical Review

The MRI Imaging Hub and/or Recruiting site will record any complications attributable to the WB-MRI or any additional test performed as a result of the WB-MRI, notably contrast reactions, biopsy complications (infection, bleeding or hospital admission), and treatment target breaches on the Staging Complications CRF which will be submitted to UCL CTC.

UCL CTC will provide trial data to the CI on a periodic basis for review. If it is declared necessary to revise the conduct of the trial due to safety concerns, UCL CTC will inform the REC as appropriate.

For peer review only

For peer review only - http://bmjopen.bmj.com/site/about/guidelines.xhtml

11. Incident Reporting

Organisations must notify UCL CTC of all deviations from the protocol or GCP immediately. UCL CTC may require a report on the incident(s) and a form will be provided if the organisation does not have an appropriate document (e.g. Trust Incident Form).

If site staff are unsure whether a certain occurrence constitutes a deviation from the protocol or GCP, the UCL CTC trial team can be contacted immediately to discuss.

UCL CTC will use an organisation's history of non-compliance to make decisions on future collaborations.

For peer review only

For peer review only - http://bmjopen.bmj.com/site/about/guidelines.xhtml

12. Trial Monitoring and Oversight

Participating sites and PIs must agree to allow trial-related on site monitoring, Sponsor audits and regulatory inspections by providing direct access to source data/documents as required. Patients are informed of this in the patient information sheet and are asked to consent to their medical notes being reviewed by appropriate individuals on the consent form.

UCL CTC will determine the appropriate level and nature of monitoring required for the trial. Risk will be assessed on an ongoing basis and adjustments made accordingly.

12.1. Central Monitoring

Sites will be requested to submit relevant logs to UCL CTC at the frequency detailed in the trial monitoring plan or on request and these will be checked for consistency and completeness. Also refer to sections 4.2.2 (Required Documentation) and 6.2 (Screening Logs).

Ensuring patient eligibility is the responsibility of the PI or other delegated Investigator(s). Checks of the criteria listed on the registration form will be undertaken by an appropriately trained UCL CTC staff member prior to registration. Also refer to section 7.1 (Registration).

Details relating to the informed consent process will be collected on the registration form and are subject to review by UCL CTC as part of patient eligibility.

Data received at UCL CTC will be subject to review in accordance with section 9.4 (Data Queries).

Where central monitoring of data and/or documentation submitted by sites indicates that a patient may have been placed at risk, the matter will be raised urgently with site staff and escalated as appropriate (refer to section 11 (Incident Reporting) and 12.3 (‘For cause’ on site monitoring) for further details).

12.2. ’For Cause’ On-Site Monitoring

On site monitoring visits may be scheduled at a site where there is evidence or suspicion of non-compliance with important aspect(s) of the trial protocol/GCP requirements. Sites will be sent a letter in advance outlining the reason(s) for the visit. The letter will include a list of the documents that are to be reviewed, interviews that will be conducted, planned inspections of the facilities, who will be performing the visit and when the visit is likely to occur.

Following a monitoring visit, the Trial Monitor/Trial Coordinator will provide a report to the site, which will summarise the documents reviewed and a statement of findings, deviations, deficiencies, conclusions, actions taken and actions required. The PI at each site will be responsible for ensuring that monitoring findings are addressed in a timely manner, and by the deadline specified.

For peer review only - http://bmjopen.bmj.com/site/about/guidelines.xhtml

UCL CTC will assess whether it is appropriate for the site to continue participation in the trial. Refer to section 11 (Incident Reporting) for details.

12.3. Oversight Committees

12.3.1. Trial Management Group (TMG)

The TMG will include the Chief Investigator, clinicians and experts from relevant specialities and Streamline C trial staff from UCL CTC (see page 3). The TMG will be responsible for overseeing the trial. The group will meet regularly at least once a year and will send updates to PIs (via newsletters or at Investigator meetings) and to the NCRI Colorectal Clinical Studies Group.

The TMG will review substantial amendments to the protocol prior to submission to the REC. All PIs will be kept informed of substantial amendments through their nominated responsible individuals.

TMG members must sign a charter confirming acceptance of their responsibilities.

12.3.2. Trial Steering Committee (TSC)

The role of the TSC is to provide overall supervision of the trial. The TSC will review the recommendations of the Independent Data Monitoring Committee and, on consideration of this information, recommend any appropriate amendments/actions for the trial as necessary. The TSC acts on behalf of the funder and Sponsor.

TSC members must sign a charter confirming acceptance of their responsibilities and declaring any potential conflicts of interest.

12.3.3. Independent Data Monitoring Committee (IDMC)

The role of the IDMC is to provide independent advice on data and safety aspects of the trial. Meetings of the Committee will be held at least once a year or as necessary to address any issues. The IDMC is advisory to the TSC and can recommend premature closure of the trial to the TSC.

IDMC members must sign a charter confirming acceptance of their responsibilities and declaring any potential conflicts of interest.

12.3.4. Role of UCL CTC

UCL CTC will be responsible for the day to day coordination and management of the trial and will act as custodian of the data generated in the trial (on behalf of UCL). UCL CTC is responsible for all duties relating to safety reporting which are conducted in accordance with section 10 (Safety Reporting).

For peer review only

For peer review only - http://bmjopen.bmj.com/site/about/guidelines.xhtml

13. Withdrawal of Patients

In consenting to the trial, patients are consenting to the WB-MRI, and also specifically consenting to completing questionnaires and diaries.

Losses to follow up

If a patient moves from the area, the site should make every effort to ensure the patient is followed up at another participating trial site and for this new site to take over the responsibility for the patient, or for follow up via GP. Details of participating trial sites can be obtained from the UCL CTC trial team who must be informed of the transfer of care and follow up arrangements.

Patients should be considered lost to follow up only once documented efforts on the part of the site have failed to produce any response or information from the patient or GP over the course of one year.

If a patient is lost to follow up at a site every effort should be made to contact the patient's GP to obtain information on the patient's status. If the patient cannot be contacted this should be recorded on the Change of Status CRF.

Patient Withdrawing Consent

If a patient expresses their wish to withdraw from the trial, sites should explain the importance of allowing routine follow up data to be used for trial purposes and for allowing existing collected data to be used. If the patient gives a reason for their withdrawal, this should be recorded (see below).

Withdrawal of Consent to Future Data Collection and use of past data

If a patient explicitly states they do not wish to contribute further data to the trial or allow the use of past data, their decision must be respected and recorded on the Change of Status CRF. In this event details should be recorded in the patient's hospital records, no further CRFs must be completed and no further data sent to UCL CTC.

Withdrawal of Consent to QoL, Resource use diary and/or Patient experience questionnaires

If a patient withdraws consent from completing the QoL, Resource use diary and/or the Patient experience questionnaires their decision must be recorded on the Change of Status CRF. UCL CTC will not post any further documents to the patient. However, the patient may continue to participate in the trial, and trial follow up completed as per protocol.

For peer review only - http://bmjopen.bmj.com/site/about/guidelines.xhtml

14. Trial Closure

14.1. End of Trial

For regulatory purposes the end of the trial will be 1 year after recruitment of the final patient at which point the 'declaration of end of trial' form will be submitted to ethical committees, as required.

Following this, UCL CTC will advise sites on the procedure for closing the trial at the site.

14.2. Archiving of Trial Documentation

At the end of the trial, UCL CTC will archive securely all centrally held trial related documentation for a minimum of 5 years. Arrangements for confidential destruction will then be made. It is the responsibility of PIs to ensure data and all essential documents relating to the trial held at site are retained for a minimum of 5 years after the end of the trial, in accordance with national legislation and for the maximum period of time permitted by the site.

Essential documents are those which enable both the conduct of the trial and the quality of the data produced to be evaluated and show whether the site complied with the principles of GCP and all applicable regulatory requirements.

UCL CTC will notify sites when trial documentation held at sites may be archived. All archived documents must continue to be available for inspection by appropriate authorities upon request.

Health Psychology data collected as part of this research will be retained for 10 years in line with Birkbeck, University of London's records management policy. At the end of this time period the documents will be shredded.

14.3. Early discontinuation of trial

The trial may be stopped before completion as an Urgent Safety Measure on the recommendation of the TSC or IDMC (see section 12.3.2 TSC and 12.3.3 IDMC). Sites will be informed in writing by UCL CTC of reasons for early closure and the actions to be taken with regards the treatment and follow up of patients.

14.4. Withdrawal from trial participation by a site

Should a site choose to close to recruitment the PI must inform UCL CTC in writing. Follow up as per protocol must continue for all patients recruited into the trial at that site and other responsibilities continue as per Clinical Trial Site Agreement (CTSA).

For peer review only - http://bmjopen.bmj.com/site/about/guidelines.xhtml

15. Quality Assurance

The technical quality of at least 10% of the WB-MRI from each imaging hub will be assessed by the trial radiographer at UCLH. Using the 3Dnet™ software, the radiographer will access performed scans on a weekly basis and enter an audit score of quality according to the definitions below. A report of the quality will be provided to the CI every 2 weeks and to the TMG at each meeting

Technical quality - general

1 = More than one sequence with substantial degradation of images severely limiting interpretation of those sequences, and not repeated

2 = One sequence with substantial degradation of images severely limiting interpretation of that sequence, and not repeated

3 = More than one sequence has minor artefact, but all remain fully diagnostic and repeat although optimal, not necessary OR all sequences initially technically inadequate (score 1 or 2) correctly repeated

4 = One sequence a has minor artefact, but remains fully diagnostic and repeat, although optimal, not necessary

5 = All sequences technically optimal with no artefact or degradation

Technical quality - anatomical coverage

1 = Wrong examination performed

2 = More than one sequence does not adequately cover the body (skull to mid thigh), or designated organ(s) coverage

3 = One sequence does not optimally cover the body or designated organ(s) coverage but examination remains fully diagnostic

4 = All sequences optimally cover the body and designated organ(s) coverage

For peer review only - http://bmjopen.bmj.com/site/about/guidelines.xhtml

16. Statistics

16.1. Sample size calculation

SAMPLE SIZE: Studies of diagnostic test accuracy are based on a cohort design where all patients receive all main diagnostic tests (CT or WB-MRI), although additional tests ordered in each staging pathway differ between patients, based on clinical appropriateness. Existing pathways and therapeutic decision making is complex for both cancers (see flow charts). This NIHR HTA trial focuses on first definitive staging and we are powered at this point in the patient pathway, informed by a detailed literature review.

Multiple therapeutic options are contingent on diagnosis of metastatic disease.

To power on any one of these (e.g. liver resection rates) would be beyond pragmatic recruitment and available funding.

We therefore power on a change in sensitivity for metastases detection with a WB-MRI pathway as a replacement to the standard NICE staging pathway.

Sample size calculation

- Paired trial design – comparing two diagnostic pathways where both are used in all patients.
- Sample size method for difference in paired proportions [22].
- Power trial to show difference in sensitivity between two diagnostic pathways, as fewer patients are with metastasis than without. Trials powered like this should also be suitable for difference in specificity.
- 80% power type II error, type I error 5% ($p<0.05$)
- Ratio of marginal cells in 2 x 2 table: s/t=7 (table 4)
- Sum of marginal cells in 2 x 2 table: s+t/N = 0.14
- A sample size of 116 patients with metastasis is needed, from a population of 40% prevalence of metastasis. Thus a total sample size of 290 (116 times 2.5) is required.

Table 4: Calculations comparing WB-MRI to PET

		MDT results for standard tests: patients with metastases detected		
	Result	+	-	Total
MDT results for WB-MRI plus additional tests: patients with metastases detected	+	85	14	99
	-	2	15	17
	Total	87	29	116

For peer review only - http://bmjopen.bmj.com/site/about/guidelines.xhtml

290 patients would be required to detect a difference of 10% in sensitivity of WB-MRI for metastatic disease (85%) compared to conventional staging (75%) given the disease prevalence as described above.

The sample size above must be adjusted to account for "withdrawals". Patients are classified as withdrawn if they are recruited but are not evaluable for any of the following reasons (i) they do not have an WB-MRI scan, the main component of the primary outcome (ii) they do not have cancer (iii) they are lost to follow up before one year and do not have sufficient information for the expert panel to evaluate whether metastases were present.

The withdrawal rate was expected to be 10%, giving a target recruitment of 290 patients. However, the actual withdrawal rate has been 19% and the target recruitment has therefore been revised to 360 patients.

16.2. Population for analysis

The analysis population will be all patients, equivalent to the RCT population intention to treat (ITT) population, with multiple imputation used to impute missing data. For the primary outcome, complete case analysis (all available patient data with no imputation) will be reported alongside as a sensitivity analysis to the ITT analysis.

16.3. Analysis of the primary endpoint

Primary Outcome- Per patient sensitivity for metastasis detection by whole body MRI (WB-MRI) compared to standard staging pathways in newly diagnosed colorectal cancer against an expert derived consensus reference standard.

The primary objective is to evaluate whether early whole body magnetic resonance Imaging (WB-MRI) increases per patient sensitivity for metastasis in colorectal cancer compared to standard NICE-approved diagnostic pathways.

- Comparison to detect metastases between early WB-MRI alone and standard NICE diagnostic strategy.
- Difference in per patient sensitivity.
- Paired comparison of proportions of each strategy against the reference standard of full clinical diagnosis and 12 month follow up. [23]
- Imputation will be used to account for missing data and imperfect reference data. [24].

Definition of positive test result from standard tests (conventional imaging)

- Conventional imaging tests include CT and may include PET, USS, organ specific MRI, bone scans, lymph node sampling.
- Report from the MDT will express presence of metastatic disease for each patient categorised as yes, no and equivocal.

For peer review only - http://bmjopen.bmj.com/site/about/guidelines.xhtml

- Equivocal results will be grouped with positive test results as these results require additional follow up investigations compared to negative results.
- The radiology imaging report supplied to the MDT will be used for sensitivity analysis using free text report (possible and probable metastasis counted as positive).

Definition of positive test result from WB-MRI and additional tests requested after WB-MRI (WB-MRI as replacement test)

- WB-MRI imaging CRF report from the WB-MRI radiologist will express presence of metastatic disease for each patient categorised as yes, no and equivocal.
- Equivocal results will be grouped with positive test results as these results require additional follow up investigations compared to negative results.

Definition of positive test result from reference test: 12 month expert consensus panel

Expert panel results will be classified as positive or negative for metastasis by expert consensus. No equivocal category will be allowed.

Multiple imputation will be used for missing data using chained equations in STATA [25].

Sensitivity analysis: A sensitivity analysis will investigate the impact of the equivocal results on the detection rate by WB-MRI. Equivocal results will be grouped with negative test results [26].

A planned subgroup analysis will include: patients with rectal and non-rectal colorectal cancer.

16.4. Analysis of secondary endpoints

16.4.1. Efficacy (secondary)

Secondary outcome- The time and test number taken to reach, and the nature of, the first major treatment decision based on WB-MRI in comparison to standard staging pathways

The objective is to determine how WB-MRI influences time to and nature of first major treatment decision following definitive staging compared to standard investigations and to determine whether early WB-MRI could reduce or replace standard investigations.

In this secondary outcome, the following components will be measured up to the time of the first major treatment decision

- Nature of first major treatment decision and number of patients with changed management decision
- Time taken for diagnostic pathways
- Number of tests: average per patient, number of patients with fewer tests in pathway

These will be compared for WB-MRI pathway in comparison to standard staging pathways.

For peer review only - http://bmjopen.bmj.com/site/about/guidelines.xhtml

This information will be reported and discussed as a basis for whether early WB-MRI could replace or reduce standard investigations.

No absolute pre-specified definition of the combination of these outcomes likely to be considered clinically significant is attempted although it is noted that:

- An average decrease in diagnosis time of 7 days is likely to be considered clinically significant if accuracy were similar.

Secondary outcome- Diagnosis accuracy of WB-MRI and conventional staging pathways for local tumour staging and detection of metastasis in comparison to an expert derived consensus reference standard

The objective is to assess the accuracy of WB-MRI and standard diagnostic pathways for local and distant staging by comparison with an expert derived consensus reference standard using 1 year patient follow up data.

- All comparisons except difference in sensitivity per patient compare WB-MRI as both replacement and additional test compared to standard NICE staging pathway

The following outcomes will be assessed as secondary outcome #2:

- Difference in sensitivity per patient: WB-MRI as additional test compared to standard NICE staging pathway
- Difference in sensitivity and specificity per organ site. Count each site once per patient, regardless of the number of metastases per organ.
- Difference in sensitivity and specificity per metastasis.

Secondary outcome- Inter-observer variability in WB-MRI analysis and effect of diagnostic confidence on staging accuracy.

The objective is to determine the effect of radiologist confidence on the diagnostic accuracy of WB-MRI for metastatic disease.

A substudy will be conducted to look at inter-observer variability between radiologists interpreting WB-MRI images and the effect of diagnostic confidence on staging accuracy.

- Each hub radiologist (n=5) will interpret a sample of 25 WB-MRI datasets selected at random from the other imaging hubs to define inter-observer variation in the reported presence absence of metastatic disease.
- These images will be read after patient management decisions are taken and so will not affect patient diagnosis or treatment.

Secondary outcome – Diagnostic accuracy of limited T1 and diffusion weighted sequences compared to full multi-sequence WB-MRI protocols

The objective is to evaluate the diagnostic performance of limited WB-MRI protocols based on diffusion and T1 weighted imaging only, and to assess the incremental benefit of intravenous gadolinium contrast enhancement.

Comparison of diagnostic accuracy of WB-MRI alone (i) block 1 sequence (ii) block 2 (iii) block 3 sequences.

- Reference standard: WB-MRI plus standard tests with 12 month follow up.

For peer review only - http://bmjopen.bmj.com/site/about/guidelines.xhtml

- Methods and tables: as per primary outcome and secondary outcome #3.
- Analysed for units of analysis as (i) per patient (ii) per organ site (iii) per metastasis.

16.4.2. Safety

We will provide information on the following:

- Additional tests ordered as a result of WB-MRI but not ordered for the patient in the standard NICE staging pathway. Note this might include tests due to abnormalities outside colon.
- Extra days in hospital due to extra tests ordered through WB-MRI.

16.4.3. Economic evaluation

Patient-specific utility profiles will then be constructed assuming a straight line relation between each of the patients EQ-5D scores at each follow up point. The quality-adjusted life-years (QALYs) experienced by each patient up to one year will be calculated as the area underneath this profile.

We will calculate for each individual patient in the trial their costs and outcomes up to one year. We will also have data from the retrospective review of resource use in UCLH patients previously diagnosed with colorectal cancer.

Individual patients will then be grouped according to the specific disease path depending on the treatment decision (e.g., surgery for the primary but no chemotherapy, surgery for the primary and chemotherapy (and/or radiotherapy), chemotherapy (and/or radiotherapy) without surgery, and surgical metastectomy with or without chemotherapy for colorectal cancer) and the accuracy of the staging result. We will calculate the mean costs and QALYs for each group. Mean costs and QALYs for WB-MRI versus standard staging algorithms will then be calculated based on the proportion in each group using each algorithm, which will be different since in this scenario there is discordance in treatment decision.

Cost-effectiveness will be calculated as the mean cost difference between WB-MRI versus standard staging algorithms divided by the mean difference in outcomes (QALYs) to give the incremental cost-effectiveness ratio (ICER). Non-parametric methods for calculating confidence intervals around the ICER based on bootstrapped estimates of the mean cost and QALY differences will be used [27]. The bootstrap replications will also be used to construct a cost-effectiveness acceptability curve, which will show the probability that WB-MRI is cost-effective at one year for different values of the NHS' willingness to pay for an additional QALY. We will also subject the results to extensive deterministic (one-, two- and multi-way) sensitivity analysis.

For the analysis based on the lifetime time horizon we will use the 1 year data described above. To extrapolate beyond the end of the 1 year follow up period we will develop a de novo cost-effectiveness model for the disease pathway, which will be populated via available evidence. These will model patient movements between long-term health states. The models will be similar in design to a recent NIHR HTA-funded trial of PET and MRI for detection of metastasis in breast cancer [28]. Following decisions about model structure, a list of parameter estimates required for the model will be developed. The specific details of the data to be used to populate the model will

For peer review only - http://bmjopen.bmj.com/site/about/guidelines.xhtml

be determined following the development of the structure and the systematic searches of the literature to identify existing models.

The upshot of this analysis is that we will calculate for each individual patient in the trial their lifetime costs and QALYs. Individual patients will then be grouped according to the specific disease path and the accuracy of the staging result, as before, and we will calculate the mean costs and QALYs for each group. Mean costs and QALYs for WB-MRI versus standard staging algorithms will then be calculated based on the proportion in each group using each algorithm, again as before. We will undertake deterministic (one-, two- and multi-way) and probabilistic sensitivity analysis, the latter assuming appropriate distributions and parameter values [29].

16.4.4. Health psychology assessment

Study 1: Patient interviews (approximate n=25)

No formal statistical analysis will be carried out on the information from the patient interviews. The interviews will provide in-depth qualitative data about the experience of cancer staging and in particular under-going WB-MRI, and will inform the modification of a questionnaire (study 2) designed to assess patient acceptability and experience of WB-MRI compared to standard tests, and also inform the design of a discrete choice experiment (study 3).

Study 2: Patient questionnaires (approximate n=50)

Comparative patient experience between WB-MRI and standard staging investigations, identification of important staging pathway attributes, comparative anxiety, expectations and attribute importance before and following the staging process will be analysed using within subjects ANOVA or the Wilcoxon matched-pair sign-test depending on whether the data are normally distributed or not.

Study 3: Discrete choice experiment (approximate n=50)

Sample size should be greater than (500*c)/(t*a) where t = the number of sets of choices, a = the number of scenarios to choose between in each choice, and c = the largest number of levels for any one attribute [30]. Assuming each person will undertake 15 sets of choices, there are two scenarios in each choice, and the largest number of levels for any one attribute is 3 then the required sample size is (500*3)/(15*2) = 50. We will administer this questionnaire to approximately 50 colon patients after staging has been completed, sampling across more than one of the imaging hubs. The questionnaire will be posted to patients with a pre-paid reply envelope. A reminder letter will be sent if no questionnaire has been returned within approximately 4 weeks. Analysis will conform to the checklist for conjoint analysis applications in health developed by the International Society for Pharmaceuticals and Outcome Research (ISPOR). We will use a random effects probit model for statistical analysis, which is appropriate given the data structure. Outcomes: The average relative importance weighting of attributes ascribed to standard versus WB-MRI staging pathways by patients diagnosed with colonic cancer.

For peer review only - http://bmjopen.bmj.com/site/about/guidelines.xhtml

16.4.5. Handling missing data

Multiple imputation will be used for missing data using chained equations in STATA [25].

For the primary outcome, complete case analysis (all available patient data with no imputation) will be reported alongside as a sensitivity analysis to the ITT analysis.

16.5. Interim analyses

Interim safety data will be supplied to the IDMC on a periodic basis from UCL CTC on:

- Accumulating information relating to recruitment and data quality (e.g. data return rates).
- Safety data:
  - Additional tests performed as a result of the WB-MRI that would not have otherwise been performed as part of the patient's standard diagnostic pathway. Note this might include tests due to abnormalities outside colon or lung.
  - Potential harms to patients (e.g. staging complications resulting from MRI).
  - Whole Body MRIs that were not revealed or revealed too early/late.
- Percentage missing data for primary outcome.
- Prevalence of metastasis within recruited patients (key sample size assumption).

There will be no interim analysis based on the trial outcomes of diagnostic accuracy as all diagnostic test results are made available for the MDT meeting which decides first management treatment decision.

For peer review only

For peer review only - http://bmjopen.bmj.com/site/about/guidelines.xhtml

17. Ethical Approvals

In conducting the trial, the Sponsor, UCL CTC and sites shall comply with all laws and statutes, as amended from time to time, applicable to the performance of clinical trials including, but not limited to:

- the principles of ICH Harmonised Tripartite Guideline for Good Clinical Practice
- the Human Rights Act 1998
- the Data Protection Act 1998
- the Freedom of Information Act 2000
- the Research Governance Framework for Health and Social Care, issued by the UK Department of Health (Second Edition 2005) or the Scottish Health Department Research Governance Framework for Health and Community Care (Second Edition 2006)

17.1. Ethical Approval

The trial will be conducted in accordance with the World Medical Association Declaration of Helsinki entitled 'Ethical Principles for Medical Research Involving Human Subjects' (1996 version) and in accordance with the terms and conditions of the ethical approval given to the trial.

The trial has received a favourable opinion from the London – Camden & Islington Research Ethics Committee.

UCL CTC will submit Annual Progress Reports to the REC, which will commence one year from the date of ethical approval for the trial.

17.2. Site Approvals

The Lead Comprehensive Local Research Network (CLRN), Central and East London CLRN, has given NHS permission following global governance checks. Local governance checks will be undertaken by local CLRNs associated with individual trial sites.

Evidence of approval from the Trust R&D for a trial site must be provided to UCL CTC. Sites will only be activated when all necessary local approvals for the trial have been obtained.

17.3. Protocol Amendments

UCL CTC will be responsible for gaining ethical approval, for amendments made to the protocol and other trial-related documents. Once approved, UCL CTC will ensure that all amended documents are distributed to sites and CLRNs as appropriate.

Site staff will be responsible for acknowledging receipt of documents and for implementing all amendments.

For peer review only - http://bmjopen.bmj.com/site/about/guidelines.xhtml

17.4. Patient Confidentiality & Data Protection

Patient identifiable data, including full name, address, date of birth, gender and NHS number will be required for the registration process and will be provided to UCL CTC. UCL CTC will preserve patient confidentiality and will not disclose or reproduce any information by which patients could be identified. Data will be stored in a secure manner and UCL CTC trials are registered in accordance with the Data Protection Act 1998 with the Data Protection Officer at UCL. Patient identifiable data will be passed onto Birkbeck, University of London. This is described in the patient information sheet and the patients will consent to it.

For peer review only

For peer review only - http://bmjopen.bmj.com/site/about/guidelines.xhtml

18. Sponsorship and Indemnity

18.1. Sponsor Details

Sponsor Name: University College London

Address: Joint Research Office
Gower Street
London
WC1E 6BT

Contact: Director of Research Support

Tel: 020 3447 9995/2178 (unit admin)

Fax: 020 3447 9937

18.2. Indemnity

University College London holds insurance against claims from participants for injury caused by their participation in the clinical trial. Participants may be able to claim compensation if they can prove that UCL has been negligent. However, if this clinical trial is being carried out in a hospital, the hospital continues to have a duty of care to the participant of the clinical trial. University College London does not accept liability for any breach in the hospital's duty of care, or any negligence on the part of hospital employees. This applies whether the hospital is an NHS Trust or otherwise.

Hospitals selected to participate in this clinical trial shall provide clinical negligence insurance cover for harm caused by their employees and a copy of the relevant insurance policy or summary shall be provided to University College London, upon request.

For peer review only - http://bmjopen.bmj.com/site/about/guidelines.xhtml

19. Funding

This trial is funded by the National Institute for Health Research Health Technology Assessment (NIHR HTA).

For peer review only

For peer review only - http://bmjopen.bmj.com/site/about/guidelines.xhtml

20. Publication Policy

The TMG will oversee the publication and presentation of the data to peer reviewed journals and scientific meetings. All members of the TMG will approve publications. The writing committee will be led by Professor Stuart Taylor and include all TMG members. All TMG members, Trial Coordinator and Trial Statistician will be authors on the publications and named individually.

For peer review only

For peer review only - http://bmjopen.bmj.com/site/about/guidelines.xhtml

21. References

1. *Cancer Stats from Cancer Research UK Website*. Available from: http://info.cancerresearchuk.org.cancerstats/.
2. Garden, O.J., et al., *Guidelines for resection of colorectal cancer liver metastases.* Gut, 2006. **55 Suppl 3**: p. iii1-8.
3. Abdalla, E.K., et al., *Improving resectability of hepatic colorectal metastases: expert consensus statement.* Ann Surg Oncol, 2006. **13**(10): p. 1271-80.
4. Buyse, M., et al., *Progression-free survival is a surrogate for survival in advanced colorectal cancer.* J Clin Oncol, 2007. **25**(33): p. 5218-24.
5. *NICE Guidance: Improving Outcomes in Colorectal Cancers 2004*. Available from: http://www.nice.org.uk/nicemedia/pdf/CSGCCfullguidance.pdf.
6. Floriani, I., et al., *Performance of imaging modalities in diagnosis of liver metastases from colorectal cancer: a systematic review and meta-analysis.* J Magn Reson Imaging, 2010. **31**(1): p. 19-31.
7. Qu, X., et al., *A meta-analysis of (1)(8)FDG-PET-CT, (1)(8)FDG-PET, MRI and bone scintigraphy for diagnosis of bone metastases in patients with lung cancer.* Eur J Radiol, 2012. **81**(5): p. 1007-15.
8. Ohno, Y., et al., *Non-small cell lung cancer: whole-body MR examination for M-stage assessment--utility for whole-body diffusion-weighted imaging compared with integrated FDG PET/CT.* Radiology, 2008. **248**(2): p. 643-54.
9. Pierce, D.A. and D.L. Preston, *Radiation-related cancer risks at low doses among atomic bomb survivors.* Radiat Res, 2000. **154**(2): p. 178-86.
10. Schmidt, G.P., M.F. Reiser, and A. Baur-Melnyk, *Whole-body MRI for the staging and follow-up of patients with metastasis.* Eur J Radiol, 2009. **70**(3): p. 393-400.
11. Squillaci, E., et al., *Staging of colon cancer: whole-body MRI vs. whole-body PET-CT--initial clinical experience.* Abdom Imaging, 2008. **33**(6): p. 676-88.
12. Yi, C.A., et al., *Non-small cell lung cancer staging: efficacy comparison of integrated PET/CT versus 3.0-T whole-body MR imaging.* Radiology, 2008. **248**(2): p. 632-42.
13. Risberg, T., et al., *Diagnostic delay causes more psychological distress in female than in male cancer patients.* Anticancer Res, 1996. **16**(2): p. 995-9.
14. Bastiaannet, E., et al., *Perception of burden experienced during diagnostic tests by melanoma patients with lymph node metastases.* Melanoma Res, 2009. **19**(1): p. 36-41.
15. von Wagner, C., et al., *Choosing between CT colonography and colonoscopy in the diagnostic context: a qualitative study of influences on patient preferences.* Health Expect, 2009. **12**(1): p. 18-26.
16. Harcourt, D., N. Rumsey, and N. Ambler, *Same-day diagnosis of symptomatic breast problems: Psychological impact and coping strategies.* Psychology, Health & Medicine 1999. **4**(1): p. 5.
17. *NICE Guide to the methods of technology appraisal 2008*. Available from: http://www.nice.org.uk/media/B52/A7/TAMethodsGuideUpdatedJune2008.pdf.
18. Bossuyt, P.M., et al., *Towards complete and accurate reporting of studies of diagnostic accuracy: The STARD Initiative.* Radiology, 2003. **226**(1): p. 24-8.
19. Bossuyt, P.M., et al., *Comparative accuracy: assessing new tests against existing diagnostic pathways.* BMJ, 2006. **332**(7549): p. 1089-92.

For peer review only - http://bmjopen.bmj.com/site/about/guidelines.xhtml

20. Ryan, M., et al., *Use of discrete choice experiments to elicit preferences.* Qual Health Care, 2001. **10 Suppl 1**: p. i55-60.
21. Ryan, M. and S. Farrar, *Using conjoint analysis to elicit preferences for health care.* BMJ, 2000. **320**(7248): p. 1530-3.
22. Julious, S.A., M.J. Campbell, and D.G. Altman, *Estimating sample sizes for continuous, binary, and ordinal outcomes in paired comparisons: practical hints.* J Biopharm Stat, 1999. **9**(2): p. 241-51.
23. Newcombe, R.G., *Improved confidence intervals for the difference between binomial proportions based on paired data.* Stat Med, 1998. **17**(22): p. 2635-50.
24. Reitsma, J.B., et al., *A review of solutions for diagnostic accuracy studies with an imperfect or missing reference standard.* J Clin Epidemiol, 2009. **62**(8): p. 797-806.
25. White, I.R., P. Royston, and A.M. Wood, *Multiple imputation using chained equations: Issues and guidance for practice.* Stat Med, 2011. **30**(4): p. 377-99.
26. Begg, C.B., R.A. Greenes, and B. Iglewicz, *The influence of uninterpretability on the assessment of diagnostic tests.* J Chronic Dis, 1986. **39**(8): p. 575-84.
27. Briggs, A.H., D.E. Wonderling, and C.Z. Mooney, *Pulling cost-effectiveness analysis up by its bootstraps: a non-parametric approach to confidence interval estimation.* Health Econ, 1997. **6**(4): p. 327-40.
28. Cooper, K.L., et al., *Positron emission tomography (PET) and magnetic resonance imaging (MRI) for the assessment of axillary lymph node metastases in early breast cancer: systematic review and economic evaluation.* Health Technol Assess, 2011. **15**(4): p. iii-iv, 1-134.
29. Briggs, A.H., K. Claxton, and M.J. Sculpher, *Decision modelling for health economic evaluation*. Oxford handbooks in health economic evaluation. 2006, Oxford: Oxford University Press. x, 237 p.
30. Johnson, R., *Conjoint Analysis Software*, I. Sawtooth Software, Editor.: Sequim, WA
31. Azvolinsky, A., *Cancer prognosis: role of BMI and fat tissue.* J Natl Cancer Inst, 2014. **106**(6): p. dju177.
32. Yip, C., et al., *Assessment of sarcopenia and changes in body composition after neoadjuvant chemotherapy and associations with clinical outcomes in oesophageal cancer.* Eur Radiol, 2014. **24**(5): p. 998-1005.
33. Attaran, S., et al., *A propensity-matched comparison of survival after lung resection in patients with a high versus low body mass index.* Eur J Cardiothorac Surg, 2012. **42**(4): p. 653-8.
34. Curvo-Semedo, L., et al., *Diffusion-weighted MRI in rectal cancer: apparent diffusion coefficient as a potential noninvasive marker of tumor aggressiveness.* J Magn Reson Imaging, 2012. **35**(6): p. 1365-71.
35. De Felice, C., et al., *Apparent diffusion coefficient on 3.0 Tesla magnetic resonance imaging and prognostic factors in breast cancer.* Eur J Gynaecol Oncol, 2014. **35**(4): p. 408-14.
36. Ohno, Y., et al., *Diffusion-weighted MRI versus 18F-FDG PET/CT: performance as predictors of tumor treatment response and patient survival in patients with non-small cell lung cancer receiving chemoradiotherapy.* AJR Am J Roentgenol, 2012. **198**(1): p. 75-82.

For peer review only - http://bmjopen.bmj.com/site/about/guidelines.xhtml

37. Zakaria, R., et al., *Diffusion-weighted MRI characteristics of the cerebral metastasis to brain boundary predicts patient outcomes.* BMC Med Imaging, 2014. **14**: p. 26.
38. Parikh, J., et al., *Changes in primary breast cancer heterogeneity may augment midtreatment MR imaging assessment of response to neoadjuvant chemotherapy.* Radiology, 2014. **272**(1): p. 100-12.

For peer review only

For peer review only - http://bmjopen.bmj.com/site/about/guidelines.xhtml

Appendix 1: Abbreviations

**CDR**	Clinical Data Repository
**CI**	Chief Investigator
**CLRN**	Comprehensive Local Research Network
**CRF**	Case Report Form
**CSP**	Coordinated System for gaining NHS Permission
**CT**	Computerised Tomography
**CTSA**	Clinical Trial Site Agreement
**DCE**	Direct Choice Experiment
**DPA**	Data Protection Act
**GCP**	Good Clinical Practice
**GHQ**	General Health Questionnaire
**ICER**	Incremental Cost-Effectiveness Ratio
**ICH GCP**	International Conference of Harmonisation-Good Clinical Practice
**IDMC**	Independent Data Monitoring Committee
**IRAS**	Integrated Research Application System
**ISF**	Investigator Site File
**ISRCTN**	International Standard Randomised Controlled Trial Number
**ITT**	Intention To Treat
**MDT**	Multi-Disciplinary Team
**MRI**	Magnetic Resonance Imaging
**NCRI**	National Cancer Research Institute
**NCRN**	National Cancer Research Network
**NHS**	National Health Service
**NICE**	National Institute for Clinical Excellence
**NIHR**	National Institute for Health Research
**NIHR HTA**	National Institute for Health Research Health Technology Assessment
**NRES**	National Research Ethics Service
**OPD**	Out Patients Department
**PACS**	Picture Archiving and Communications System
**PANAS**	Positive and Negative Affect
**PET**	Positron Emission Tomography
**PET-CT**	Positron Emission Tomography- Computerised Tomography
**PI**	Principal Investigator
**PSS**	Personal Social Services
**QA**	Quality Assurance
**QALY**	Quality Adjusted Life Year
**R&D**	Research & Development
**RCT**	Randomised Controlled Trial
**REC**	Research Ethics Committee
**RIS**	Radiology Information System
**SSI**	Site Specific Information
**STARD**	STAndards for the Reporting of Diagnostic accuracy studies
**T**	Tesla
**TMF**	Trial Master File

For peer review only

For peer review only - http://bmjopen.bmj.com/site/about/guidelines.xhtml

Streamline C

**TMG**	Trial Management Group
**TSC**	Trial Steering Committee
**UCL**	University College London
**UCLH**	University College London Hospital
**UCL CTC**	Cancer Research UK & UCL Cancer Trials Centre
**USS**	Ultrasound Scan
**VATS**	Video-Assisted Thoracoscopic Surgery
**WB-MRI**	Whole Body Magnetic Resonance Imaging
**WCBP**	Woman of Childbearing Potential

For peer review only

For peer review only - http://bmjopen.bmj.com/site/about/guidelines.xhtml

Appendix 2: Consent process

Patients will be recruited at several hospital sites, and for many, the actual WB-MRI will take place at a different hospital (one of the central imaging hubs). It is vital that recruited patients undergo informed consent, have a minimum of 24 hours to consider participation and are subsequently free to withdraw at any time.
The consent process must be sensitive to the needs of patients which may differ from individual to individual. It is important therefore to provide patients with options as to how they may provide their consent.

The proposed consent process for this trial is based on the experience of other similar trials. It was found that patients and recruitment sites found it more efficient if the designated recruiting individual at the peripheral hospital knows that after fully explaining the trial, the patient in principle is willing to take part. For all recruitment sites, patients are seen by a clinical practitioner after a suspected diagnosis of colon cancer based on an abnormal endoscopy, CT scan or barium enema. At this discussion the diagnosis is explained and the need for a series of staging tests (such as CT scanning, PET CT, and MRI etc.) is also explained. Because of the need to stage the patient quickly so treatment may start, these tests are often done within a very short time period. There is no scheduled return by the patient to an OPD during this staging process. Instead patients are next seen face to face after the MDT has made a decision about their treatment.

If consent in principle is given, the recruiter can then ring the central hub and there and then arrange the time of the MRI and thus whenever possible inform the patient immediately of the time and date so they can plan their attendance for the scan. Patients expressing an interest in participating in the trial will therefore be asked if they prefer to sign the consent form when first seen when the trial is explained (and this will be later filed in the medical notes and the conversation documented). It will be made perfectly clear that they are free to withdraw at any time and that the scan will be at least 24 hours later (usually more than this). This will save them having to re-attend the hospital simply to sign a consent form, incurring time and expense at a very busy and difficult time for them. Before they go home, where possible patients will be given the date of the WB-MRI and a copy of their signed consent form. The recruitment site can then register the patient with UCL CTC. The date of the WB-MRI will be at least 24 hours later and in reality likely several days later. The patient will take the full patient information sheet home to review at their leisure. They will be provided with a telephone number at the recruitment site to ring if on reflection, they want to opt out of the trial. If they opt out, this will be recorded in the medical notes and screening log. Patients will also be asked permission to be called by the recruitment site or imaging hub to remind them of the date of scan and confirm their attendance. Patients will retain the right to opt out at any time by cancelling or not attending their WB-MRI appointment.

As noted, a major advantage of this approach is that the patient does not have to make a special trip back to the hospital just to sign the consent form. The signed consent

For peer review only - http://bmjopen.bmj.com/site/about/guidelines.xhtml

form does not leave the recruitment hospital and can be more efficiently filed in the patient notes and a copy filed in the Investigator site file: an important aspect of trial governance and patient clinical care.

Note this method of consent has been previously deemed ethically sound and efficient in the NIHR HTA funded Siggar 1 trial (CT colonography, colonoscopy or barium enema for diagnosis or colorectal cancer in older symptomatic patients), Northern and Yorkshire MREC, MREC/3/3/075, which ran from 2004 to 2009 and recruited over 5000 patients to undergo CT colonography in a similar trial design to the current proposal. The trial team did pilot a scheme where patients returned a copy of their signed consent form after 24 hours if they still wanted to take part, but patients found this very cumbersome and confusing and much preferred the process proposed, using telephone confirmation. The procedure is supported by the patient representatives in the trial and has also worked successfully in the BOOST trial which randomised patients to standard care or endobronchial biopsy at exactly the same point in the patient pathway. The BOOST trial ran at many of the intended recruitment sites for Streamline C.

Patients will be given the option to come back and sign the consent form if they or their recruiter feel this is preferable.

Occasionally it may be more convenient for the patient to obtain written consent when the patient attends for the WB-MRI or before a planned conventional staging test. For example the patient may wish to take part in the trial, not wish to sign the consent form on the same day but also would prefer not to attend a hospital appointment just to sign the consent form. This is acceptable although it is the least desirable option. After the patient has signed consent they will need to be registered before proceeding with the WB-MRI and a copy of the consent form will need to be returned to the initial recruitment site.

For peer review only - http://bmjopen.bmj.com/site/about/guidelines.xhtml

Appendix 3: Minimum WB-MRI protocol dataset

MRI Protocol
Whole body is head to thigh

The aim is to complete the whole protocol in 60 minutes or less. Depending on the MRI technology available, it may be possible to use the 5mm slices for all axial imaging, although up to 7mm is acceptable if time constraints are problematic.

1. These specifications are a set of minimum requirements – where higher field strengths/scanner software permits the resolution/quality of sequences should be optimised (whilst keeping the maximum imaging time limited to 1 hour).

2. Scanning maybe performed at either 1.5T or 3T.

3. Whole-body coverage is defined as head to mid-thigh optimised for detection of metastases.

Imaging Procedure

a) A standard safety questionnaire should be completed.
b) For patients undergoing contrast enhancement: set up IV line in a vein in the antecubital fossa, connected to an automated injector with two syringes (contrast and saline flush).
c) Unless contra-indicated administer 20mg buscopan or 1mg glucagon iv. to be given just before the start of the scan.

1. Whole-body diffusion weighted imaging:
Axial: STIR-EPI (or other fat sat technique) diffusion weighted imaging. Fixed slice thickness of 5mm to 7mm (to match T2 and t1 weighted axials as below) two b-values (b50 and b900). A minimum acquisition matrix of 128 x 128 (or an interpolated equivalent) (rectangular FOV should be used if available and appropriate for the patient), as a reference a minimum SNR of 6 on b50 images (for liver) should be maintained if possible by increasing the number of averages. All imaging should be performed in gentle respiration (recommended as 4 stations of 50 slices beginning from the vertex to mid thighs). Diffusion imaging through the brain is optional.

2. Whole-body T2 weighted imaging:
Axial*:* Axial T2 weighted (without fat-suppression) imaging should be performed from vertex to mid-thigh. A 5 to 7 mm slice thickness should be used for all scanners. *Where possible (within the 60 minute imaging time)* respiratory and ECG triggering should be used for the chest, respiratory triggering alone for the upper abdomen. The head, neck, pelvis and legs should be scanned without any triggering. The number of stacks should be adjusted to cover the imaging volume.

For peer review only - http://bmjopen.bmj.com/site/about/guidelines.xhtml

Streamline C

4. **Pre-contrast T1 weighted imaging:**

DIXON Technique to be applied if available.

a. Axial: Whole-body T1 GRE (e.g. Flash 2D) non-contrast enhanced non fat sat. Image resolution and slice thickness should be ideally matched to T2 weighted imaging.

OR

b. Coronal: T1 fat saturated volume interpolated gradient echo imaging (e.g. 3D) pre contrast.

5. **Post-contrast T1 weighted imaging (if gadolinium not contraindicated or refused):**

Minimum data set
Axial liver (60-70 sec)
Axial lung (equilibrium phase)
SFOV axial head

Optional
Coronal (organ specific or whole body)

a. Axial: post contrast e.g. T1 fat saturated volume interpolated gradient echo imaging (3D) breath hold of the *liver (60-70 seconds delay) and lungs*. Multiple breath-holds employed to provide full volume coverage if required. A minimum of a 256x256 (rectangular FOV acquisition if possible and appropriate for the patient) acquisition matrix should be employed. 5-7 mm slice thickness.

b. Coronal: post contrast whole body; e.g.T1 fat saturated volume interpolated gradient echo imaging (3D) and post contrast. Slice thickness 5mm. Breath Hold.

c. **Axial:** fat saturated T1 weighted imaging of the brain (SFOV). An acquisition matrix of 256 x 256 should be employed.

For peer review only

For peer review only - http://bmjopen.bmj.com/site/about/guidelines.xhtml

Appendix 4: Exploratory Analysis

The collated WB-MRI datasets and 12 months patient follow up data collated as part of the trial protocol provides opportunities to retrospectively mine the data for additional diagnostic indices and prognostic markers. All analysis will be done by members of the research team under supervision of the Chief Investigator.

There is for example, increasing data linking total body fat with prognosis in malignancy [31, 32]. In lung cancer, a higher body mass index has been associated with a better prognosis [33]. From the WB-MRI datasets acquired as part of the trial, it is possible to extract quantitative data on total body fat using simple segmentation algorithms which can be linked with patients' outcomes such as metastatic status and 12 month survival.

In addition, quantification of the MRI signal returned from sites of disease may be able to predict histological characteristics and also may be linked to prognosis. For example diffusion weighted imaging of the primary tumour has been shown to differentiate between grades of dysplasia in rectal cancer [34] and may predict prognosis in lung, brain and breast cancer [35-37]. The heterogeneity of signal within tumours can be measured and has been linked with overall prognosis and treatment response. For example in breast cancer, increased homogeneity of the signal measured using regions of interest placed in metastatic deposits in response to treatment is associated with a better prognosis [38]. The various sequences performed as part of the collected WB-MRI protocol will allow testing of such associations in recruited patients.

The following or similar may be quantified in all or a subset of WB-MRI datasets:

1. Body fat (organ, visceral and whole body).
2. MRI signal from the primary tumour and metastatic sites, notably diffusion weighted signal, apparent diffusion coefficient, T1 (including fat and water components), T2 signal, Contrast enhancement, and textural analysis (for heterogeneity) of the primary and metastatic deposits on the various sequences.

We will look for correlations between MRI variables and clinical data collated as part of the trial (for example basic histology, metastatic status, patient outcome and findings of conventional staging tests) or collected as part of the patients' usual care, for example, more detailed histology of biopsies or excised tumour (including tumours markers if collected as part of usual clinical care). Associations will be sought between the MRI variables and the clinical variables using appropriate conventional parametric and non-parametric statistics, for example:

- Differences in body fat composition between different cancer histological subtypes, patients with and without metastatic disease, and according to 12 month survival
- Heterogeneity of MRI signal from a region of interest drawn within the primary tumours and metastatic deposits, comparing between lesions in the same

For peer review only - http://bmjopen.bmj.com/site/about/guidelines.xhtml

patient, between different patients and according to histological parameters and 12 month survival.

In addition comparisons will be made between MRI signal within normal body tissues (such as the liver, pancreas and muscle) between patients scanned on the different MRI scanners comparing with local MRI phantom data as appropriate to see how homogenous the MRI data is between different imaging hubs.

In some instances use of external commercial software may be required (e.g. fat quantification). MRI datasets will be anonymised and no personal identifiable information will be shared outside the research team at any time.

For peer review only

For peer review only - http://bmjopen.bmj.com/site/about/guidelines.xhtml

Appendix 5: Protocol Version History

**Protocol:**		**Amendments:**		
**Version no.**	**Date**	**Amendment no.**	**Protocol Section (no./title)**	**Summary of main changes from previous version.**
1.0				
1.1	14/09/12	N/A	Front cover & Section 1.1<br>Section 5<br>8.2.10<br>Section 14.2<br>Appendix 2	ISRCTN number and new CR UK logo added<br>Flowchart and text re consent process inserted<br>Text inserted re QoLs<br>Text inserted re destruction of interview recordings<br>Text inserted re consent process
2.0	15/03/13	1.0	Throughout<br>Section 4<br>Section 5<br>Section 7<br>Section 8<br>Section 9<br>Section 12<br>Section 13<br>Appendix 3	Typos and clarifications<br>Clarification about GCP training and documents required for activation updated<br>Details added about interpreters for informed consent and removal of informed consent log information<br>Removal of text regarding slot availability updates<br>Addition of ad hoc mini MDT, reduce the amount of patient experience questionnaires, change the content of these questionnaires and alter who is sending them to patients.<br>Clarifications of timing of WB-MRI and the process of early release of findings<br>Update data management guidelines in line with changes at UCL CTC<br>Details about informed consent log removed<br>Addition of details re patient withdrawal<br>Clarifications to Whole body MRI protocol
3.0	24/04/14	3.0	Section 5<br>Section 7<br>Section 8	Removal of information about QoL and Patient diaries given out by site at consent<br>Removal of patient diaries and QoLs from registration packs.<br>Updating information to state that the Interview section of the trial is now closed to recruitment.<br>Updating information that UCL CTC is now posting out QoL and patient diaries at baseline.

For peer review only - http://bmjopen.bmj.com/site/about/guidelines.xhtml

Streamline C

4.0	22/12/14	5.0	Section 8<br><br>8.2.12<br><br>Section 10<br><br>Appendix 4<br><br>Appendix 5	Rewording and clarification of Conventional Imaging, WB-MRI reveal and MDT discussion, Final Treatment decision and follow up sections. Updating information to state that the Interview section of the trial is now closed to recruitment. Updating information that UCL CTC is now posting out QoL and patient diaries at baseline.<br>Information added regarding exploratory analysis for additional diagnostic indices and prognostic markers.<br>Safety Reporting section updated to provide more information on staging complications and how these are reported and reviewed by the CI.<br>Exploratory Analysis added to include the additional analysis of WB-MRI scans<br>Protocol Version History has become Appendix 5.
5.0	27/08/15	5.0	Section 8<br><br>Section 10<br><br>Section 13<br><br>Section 16	Changing the target recruitment for the patient experience questionnaires to 50 patients and closing this part of the trial. Updating and clarifying information regarding the Discrete Choice Experiment and confirming the time point these will be sent to patients.<br>Updating the Safety Section to confirm that only staging complications related to the WB-MRI or additional tests performed due to WB-MRI are to be recorded on the Staging Complications CRF.<br>The withdrawal process was updated and clarified to better match the new Change of Status CRF.<br>The type of Interim safety data that will be supplied to IDMC was updated
6.0	06/09/16	7.0	Section 1<br><br>Section 13<br><br>Section 16	Recruitment target increased from 322 to 360 in sections 1.1 and 1.2.<br>Anticipated duration of recruitment increased from 3 to 4 years in sections 1.1 and 1.2.<br>Deletion of section on early release of WB MRI and patient failure to undergo WB MRI<br>Rationale for revised target accrual added to 16.1.<br>Grammatical and typographical errors corrected throughout

For peer review only

For peer review only - http://bmjopen.bmj.com/site/about/guidelines.xhtml

COREQ (COnsolidated criteria for REporting Qualitative research) Checklist

A checklist of items that should be included in reports of qualitative research. You must report the page number in your manuscript where you consider each of the items listed in this checklist. If you have not included this information, either revise your manuscript accordingly before submitting or note N/A.

Topic	Item No.	Guide Questions/Description	Reported on Page No.
**Domain 1: Research team and reflexivity**			
Personal characteristics			
Interviewer/facilitator	1	Which author/s conducted the interview or focus group?	Page 8
Credentials	2	What were the researcher's credentials? E.g. PhD, MD	Page 8
Occupation	3	What was their occupation at the time of the study?	Page 8
Gender	4	Was the researcher male or female?	Page 8
Experience and training	5	What experience or training did the researcher have?	Page 8
Relationship with participants			
Relationship established	6	Was a relationship established prior to study commencement?	Page 7
Participant knowledge of the interviewer	7	What did the participants know about the researcher? e.g. personal goals, reasons for doing the research	Page 8
Interviewer characteristics	8	What characteristics were reported about the inter viewer/facilitator? e.g. Bias, assumptions, reasons and interests in the research topic	Page 8
**Domain 2: Study design**			
Theoretical framework			
Methodological orientation and Theory	9	What methodological orientation was stated to underpin the study? e.g. grounded theory, discourse analysis, ethnography, phenomenology, content analysis	Page 9
Participant selection			
Sampling	10	How were participants selected? e.g. purposive, convenience, consecutive, snowball	Page 9
Method of approach	11	How were participants approached? e.g. face-to-face, telephone, mail, email	Page 7
Sample size	12	How many participants were in the study?	Page 8
Non-participation	13	How many people refused to participate or dropped out? Reasons?	Page 8
Setting			
Setting of data collection	14	Where was the data collected? e.g. home, clinic, workplace	Page 7
Presence of non-participants	15	Was anyone else present besides the participants and researchers?	Page 7
Description of sample	16	What are the important characteristics of the sample? e.g. demographic data, date	Pages 10& 11
Data collection			
Interview guide	17	Were questions, prompts, guides provided by the authors? Was it pilot tested?	Box 1 and Page 8
Repeat interviews	18	Were repeat inter views carried out? If yes, how many?	Page 7
Audio/visual recording	19	Did the research use audio or visual recording to collect the data?	Page 8
Field notes	20	Were field notes made during and/or after the inter view or focus group?	Page 9
Duration	21	What was the duration of the inter views or focus group?	Page 8
Data saturation	22	Was data saturation discussed?	Page 8
Transcripts returned	23	Were transcripts returned to participants for comment and/or	Page 9

For peer review only

Topic	Item No.	Guide Questions/Description	Reported on Page No.
		correction?	
**Domain 3: analysis and findings** *Data analysis*			
Number of data coders	24	How many data coders coded the data?	Page 9
Description of the coding tree	25	Did authors provide a description of the coding tree?	Page 9
Derivation of themes	26	Were themes identified in advance or derived from the data?	Page 9
Software	27	What software, if applicable, was used to manage the data?	Page 9
Participant checking	28	Did participants provide feedback on the findings?	Page 9
Reporting			
Quotations presented	29	Were participant quotations presented to illustrate the themes/findings? Was each quotation identified? e.g. participant number	Pages 11-18
Data and findings consistent	30	Was there consistency between the data presented and the findings?	Pages 11-18
Clarity of major themes	31	Were major themes clearly presented in the findings?	Pages 11-18
Clarity of minor themes	32	Is there a description of diverse cases or discussion of minor themes?	Pages 11-18

Developed from: Tong A, Sainsbury P, Craig J. Consolidated criteria for reporting qualitative research (COREQ): a 32-item checklist for interviews and focus groups. *International Journal for Quality in Health Care*. 2007. Volume 19, Number 6: pp. 349 – 357

Once you have completed this checklist, please save a copy and upload it as part of your submission. DO NOT include this checklist as part of the main manuscript document. It must be uploaded as a separate file.

For peer review only

BMJ Open

Patient experience and perceived acceptability of whole body magnetic resonance imaging for staging colorectal and lung cancer compared with current staging scans: a qualitative study

Journal:	*BMJ Open*
Manuscript ID	bmjopen-2017-016391.R2
Article Type:	Research
Date Submitted by the Author:	02-Jun-2017
Complete List of Authors:	Evans, Ruth; Birkbeck University of London, Psychological Sciences<br>Taylor, Stuart; University College London Hospitals NHS Foundation Trust, Centre for Medical Imaging<br>Janes, Sam; University College London, Respiratory Medicine<br>Halligan, Steve; University College London, Centre for Medical Imaging<br>Morton, Alison; Patient Representative<br>Navani, Neal; University College London, Respiratory medicine<br>Oliver, Alf; Patient Representative<br>Rockall, Andrea; Royal Marsden NHS Foundation Trust, Radiology<br>Teague, Jonathan; Cancer Research UK and UCL Clinical Trials Centre<br>Miles, Anne; Birkbeck, University of London,
**Primary Subject Heading**:	Radiology and imaging
Secondary Subject Heading:	Oncology, Patient-centred medicine, Qualitative research
Keywords:	Magnetic resonance imaging < RADIOLOGY & IMAGING, Respiratory tract tumours < ONCOLOGY, Gastrointestinal tumours < ONCOLOGY, QUALITATIVE RESEARCH

SCHOLARONE™ Manuscripts

For peer review only

Title: Patient experience and perceived acceptability of whole body magnetic resonance imaging for staging colorectal and lung cancer compared with current staging scans: a qualitative study.

Authors: REC Evans[a], SA Taylor[b], S Janes[c], S Halligan[b], A Morton[d], N Navani[c], A Oliver[d], A Rockall[e], J Teague[f] and A Miles[a]

[a] Psychological Sciences, Birkbeck, University of London, Malet Street, London, WC1E 7HX

[b] Centre for Medical Imaging, Division of Medicine, University College London, Gower Street, London, WC1E 6BT

[c] Lungs for Living Research Centre, Division of Medicine, University College London, Gower Street, London, WC1E 6BT

[d] Patient Representative, no institutional address available

[e] Department of Surgery and Cancer, Imperial College London, Kensington, London SW7 2AZ

[f] CR UK &UCL Clinical Trials Centre, 90 Tottenham Court Road, London, W1T 4TJ

Corresponding author: Dr Anne Miles, Psychological Science, Birkbeck, University of London, Malet Street, London, WC1E 7HX, ae.miles@bbk.ac.uk, Tel: +44 20 7631 6488

Funding statement:

This project was funded by the National Institute of Health Research health technology assessment NIHR HTA programme (project number 10/68/01) and will be published in full in Health Technology Assessment. The project is supported by researchers at the National

For peer review only - http://bmjopen.bmj.com/site/about/guidelines.xhtml

Institute for Health Research University College London Hospitals Biomedical Research Centre.

Department of Health disclaimer:

This report presents independent research commissioned by the National Institute for Health Research (NIHR). The views and opinions expressed by authors in this publication are those of the authors and do not necessarily reflect those of the NHS, the NIHR, NETSCC or the HTA programme or the Department of Health. The views and opinions expressed by the interviewees in this publication are those of the interviewees and do not necessarily reflect those of the authors, those of the NHS, the NIHR, MRC, CCF, NETSCC or the HTA programme or the Department of Health.

Competing interests

We have read and understood BMJ policy on declaration of interests and declare that we have no competing interests.

Authors' contribution

SA Taylor, S Janes, S Halligan, A Morton, N Navani, A Oliver, A Rockall and A Miles designed the clinical trial protocol which includes this study. J Teague assisted in patient recruitment. REC Evans and A Miles interviewed patients, analysed and interpreted the data. REC Evans wrote the first draft of the manuscript. All authors reviewed the manuscript and approved the final draft.

Acknowledgments:

The authors would like to acknowledge the contribution of the following individuals to the clinical trial which provided the data presented in this paper: A Aboagye, L Agoramoorthy,

For peer review only - http://bmjopen.bmj.com/site/about/guidelines.xhtml

S Ahmed, A Amadi, G Anand, G Atkin, A Austria, S Ball, F Bazari, R Beable, S Beare, H Beedham, T Beeston, N Bharwani, G Bhatnagar, A Bhowmik, L Blakeway, D Blunt, P Boavida, D Boisfer, D Breen, J Bridgewater, S Burke, R Butawan, Y Campbell, E Chang, D Chao, S Chukundah, B Collins, C Collins, V Conteh, J Couture, J Crosbie, H Curtis, A Daniel, L Davis, K Desai, M Duggan, S Ellis, C Elton, A Engledow, C Everitt, S Ferdous, A Frow, M Furneaux, N Gibbons, R Glynne-Jones, A Gogbashian, V Goh, S Gourtsoyianni, A Green, Laura Green, Liz Green, A Groves, A Guthrie, E Hadley, A Hameeduddin, G Hanid, S Hans, B Hans, A Higginson, L Honeyfield, H Hughes, J Hughes, L Hurl, E Isaac, M Jackson, A Jalloh, R Jannapureddy, A Jayme, A Johnson, E Johnson, P Julka, J Kalasthry, E Karapanagiotou, S Karp, C Kay, J Kellaway, S Khan, D Koh, T Light, P Limbu, S Lock, I Locke, T Loke, A Lowe, N Lucas, S Maheswaran, S Mallett, E Marwood, J McGowan, F Mckirdy, T Mills-Baldock, T Moon, V Morgan, S Morris, S Nasseri, P Nichols, C Norman, E Ntala, A Nunes, A Obichere, J O'Donohue, I Olaleye, A Onajobi, T O'Shaughnessy, A Padhani, H Pardoe, W Partridge, U Patel, K Perry, W Piga, D Prezzi, K Prior, S Punwani, J Pyers, H Rafiee, F Rahman, I Rajanpandian, S Ramesh, S Raouf, K Reczko, A Reinhardt, D Robinson, P Russell, K Sargus, E Scurr, K Shahabuddin, A Sharp, B Shepherd, H Sidhu, I Simcock, C Simeon, A Smith, D Smith, D Snell, J Spence, R Srirajaskanthan, V Stachini, S Stegner, J Stirling, N Strickland, K Tarver, M Thaha, M Train, S Tulmuntaha, N Tunariu, K van Ree, A Verjee, C Wanstall, S Weir, S Wijeyekoon, J Wilson, S Wilson, T Win, L Woodrow, D Yu.

Data sharing statement: Extra data from anonymised transcripts are available by emailing ae.miles@bbk.ac.uk

For peer review only

ABSTRACT:

Objective: To describe the experience and acceptability of whole body magnetic resonance imaging (WB-MRI) staging compared with standard scans among patients with highly suspected or known colorectal or lung cancer.

Design: Qualitative study using one-to-one interviews with thematic analysis.

Setting: Patients recruited from 10 hospitals in London, East and South East England between March 2013 and July 2014.

Participants:51 patients (31 male, age range 40 to 89 years), with varying levels of social deprivation were recruited consecutively from two parallel clinical trials comparing the diagnostic accuracy and cost-effectiveness of WB-MRI with standard scans for staging colorectal and lung cancer ('Streamline- C' and 'Streamline-L'). WB-MRI was offered as an additional scan as part of the trials.

Results: In general WB-MRI presented a greater challenge than standard scans, although all but 4 patients completed the WB-MRI. Key challenges were: enclosed space, noise and scan duration; reduced patient tolerance was associated with claustrophobia, pulmonary symptoms and existing co-morbidities. Coping strategies facilitated scan tolerance and were grouped into 1) those intended to help with physical and emotional challenges, and 2) those focused on motivation to complete the scan e.g. focusing on health benefit. Our study suggests that good staff communication could reduce anxiety and boost coping strategies.

Conclusions: Although WB-MRI was perceived as more challenging than standard scans, it was sufficiently acceptable and tolerated by most patients to potentially replace them if appropriate.

Trial Registration: ISRCTN43958015 and ISRCTN50436483

Keywords: Cancer Oncology Neoplasms Magnetic Resonance Imaging Patient Satisfaction

For peer review only - http://bmjopen.bmj.com/site/about/guidelines.xhtml

ARTICLE SUMMARY

Strengths and limitations of this study

- This study is the first to describe the experience and acceptability of having a whole body MRI in patients with highly suspected lung or colorectal cancer compared with standard scans.
- Qualitative methodology, and large numbers recruited (with wide age range and deprivation levels), enabled us to observe that there is a range of intensity of difficulties experienced during the whole body MRI.
- Assessment within the context of a research trial, with some patient declining participation due to claustrophobia, might have led to an under-reporting of WB-MRI challenge.

For peer review only

For peer review only - http://bmjopen.bmj.com/site/about/guidelines.xhtml

BACKGROUND

Current NICE recommended staging pathways for lung and colorectal cancer are often complex and time consuming, involving several different imaging tests utilising ionising radiation, and potentially adding to the physical and psychological burden of patients with suspected cancer (1;2). For example it is not unusual for a patient with suspected lung cancer to undergo diagnostic CT, followed by staging PET-CT, dedicated brain imaging and invasive mediastinal nodal sampling prior to the first major treatment decision. More accurate and streamlined staging could improve patient outcomes both by triaging to optimal therapy and decreasing the time between diagnosis and treatment (3). Whole body magnetic resonance imaging (WB-MRI) has been advocated as a safe accurate and efficient "one stop shop" investigation that could potentially replace current complex multi-modality staging strategies (4). A single WB-MRI scan could not only accelerate staging but would simultaneously reduce exposure to ionising radiation, in theory reducing the risk of subsequent radiation induced malignancies, particularly in those diagnosed at a younger age. WB-MRI however can be stressful: its duration, 45 to 90 minutes, is longer than standard tests - CT takes a few seconds. MRI scanners are noisy and require full body and head immersion into a relatively narrow tube, often necessitating coils wrapped around the patient that restrict movement. Somewhere between 5-30% of patients experience distress associated with the anticipated and actual experience of undergoing MRI (5-9). Anxiety relates to the scan experience itself as well as the result (5). Severe claustrophobia can lead to premature scan termination in 1-15% of attempts (10;11), and distress is associated with elevated post scan anxiety (6), which can engender MRI fear or phobia (12-14), especially problematic in patients needing future MRI studies.

For peer review only - http://bmjopen.bmj.com/site/about/guidelines.xhtml

Increased physical and psychological vulnerability of patients with suspected cancer may reduce their ability to cope with a WB-MRI (2;15). Cancer diagnosis and subsequent treatment are still much feared despite recognition of better treatment outcomes (16;17); and patients may be experiencing shock, anxiety and worries about the future (15;18). While two small qualitative studies have investigated general patient experience of MRI (19;20) there have been few descriptions of patients undergoing WB- MRI, particularly in those diagnosed with or highly suspected of having cancer.

Patient acceptability is central to the successful adoption of any new technology. Poor acceptability could reduce adherence to WB-MRI, which in turn considerably blunts the impact of the technology, even if diagnostically superior to existing tests.

Two parallel, multi-centre, prospective cohort studies (21) investigating the diagnostic accuracy and cost-effectiveness of WB-MRI compared to standard pathways for newly diagnosed lung (‘Streamline-L’) and colon (‘Streamline-C’) cancer have recently completed. The aim of this study was to describe patient experience and acceptability of WB-MRI and compare with standard staging tests, in the context of lung and colorectal cancer, using interviews from patients recruited to the Streamline trials. In the trials, the WB-MRI was offered as an additional scan alongside those performed as part of standard care.

METHODS

There is little existing research describing patients’ experiences of undergoing WB-MRI and we adopted a qualitative design as this provides the flexibility to capture rich descriptions of experiences without a priori knowledge of potential responses. One-to-one interviews were completed face to face (at home or at hospital) or via telephone. Individual interviews were

For peer review only - http://bmjopen.bmj.com/site/about/guidelines.xhtml

conducted to facilitate expression of emotions and negative experiences that may be inhibited in a group setting. For two interviews a relative was present, to support translation where English was not the patient's first language. Patients were interviewed only once.

Trial and interview study recruitment

Patients were recruited to the 'Streamline' trials from 10 English NHS hospitals and consented to undergo WB-MRI in addition to standard staging investigations at one of five MRI scan hospital hubs. During trial consent, patients could opt into the interview study and were contacted, by psychology researchers by phone, as soon as possible after all imaging had been completed. Prior to trial consent patients were given information about cancer staging and the WB-MRI; staging was explained to patients as a process where doctors use tests to assess whether cancer has spread around the body to help decide best treatment, the trial was described as an assessment about whether the WB-MRI may be quicker or better at staging newly diagnosed cancer than standard scans. Accelerated treatment pathways meant some interviews (n=13) were undertaken after treatment had commenced. Trial inclusion/ exclusion criteria are published (21).

The a priori total sample size was a minimum of 50, stratified by cancer site (25 lung and 25 colorectal patients). There was flexibility to recruit beyond this number to achieve saturation, i.e. the point beyond which further interviews contribute little new information, although this was not needed. The first 123 trial patients were approached and 91 (74%) agreed to be interviewed. Of the 91 who agreed, 51 patients (56%) participated; we believed we had reached saturation by 50 patients and ceased interviews, however one additional interview was subsequently completed as a patient (from the original 91 consenting patients) expressed a strong wish to share their views. Reasons patients were not interviewed included: consent

For peer review only - http://bmjopen.bmj.com/site/about/guidelines.xhtml

retracted (n=8); participation in a related questionnaire study (n=14); withdrawal from the main trial (n=12); and interview quota reached before completion of all staging imaging tests (n=6).

Conducting the interview

Interviews were conducted from March 2013 to July 2014, by two female psychology researchers (PhD qualified), independent of the clinical trial team, with prior training and experience of conducting in-depth interviews with cancer patients(22;23). The researchers introduced themselves as working with the doctors at the patient's hospital and explained that the interview was an opportunity to provide feedback about the WB-MRI and other tests the patients had had. A topic guide was developed (Box 1) to encourage discussion of key aspects of the staging experience including comparisons between WB-MRI and standard investigations. This guide was reviewed after completion of the first two interviews to assess whether it needed to be modified. Topic ordering and emphasis varied depending on relevance to each patient. Open ended questions were followed by verbal probing to elicit further clarification. Interviews lasted between 12 and 86 minutes (mean 48 minutes), and were recorded digitally. Participating patients were paid £20 plus travel expenses.

Box 1: Key interview topics

- *I understand that you have been having a number of scans to investigate your symptoms, can you tell me what's been happening?*
- *Can you describe what it feels like physically to have the scan?*
- *How did you feel during/after/about the test?*
- *What were the staff like?*

For peer review only - http://bmjopen.bmj.com/site/about/guidelines.xhtml

- *What did you do to cope with [problem identified by patient e.g. noise, keeping still, etc]*
- *Would you have another WB-MRI if the doctor recommended it?*

Additional measures

Age, gender and postcode data were collated. Postcode was used to calculate an area based deprivation score using the 2010 IMD scale (24) which was then categorised into quintiles (quintile 1 highest and quintile 5 lowest deprivation).

Data capture, coding and analysis

Interviews were transcribed verbatim. NVIVO, version 10, was used to manage data (tagging and labelling) while completing thematic analysis (25). A coding structure was developed by (psychology researcher name removed), using transcript review combined with a reflective log maintained during interviews, this was reviewed and agreed by both psychology researchers. This framework identified themes related to scan events, beliefs, attitudes, and emotional responses as well as coping strategies. Themes identified were influenced by prior research as well as emergent from the data. This process was iterative, with constant data comparisons to identify similarities and differences within and across individual interviews. Matrix tables were created with themes as columns and relevant data summarised into separate rows from each transcript. Matrix tables provided an overview of all 51 interviews, and ensured that the full range of experiences were represented in the final description. Patients were not asked to verify the thematic analysis so as not to increase participation burden.

Statistical analysis

For peer review only - http://bmjopen.bmj.com/site/about/guidelines.xhtml

Data were entered into SPSS, version 20. Analysis Of Variance assessed differences in mean ages between lung and colorectal patient groups; Chi Square assessed group differences in gender, deprivation and interview method; and the Mann-Whitney test was used to assess differences in the median time interval from WB-MRI scan to interview date, between patient groups.

Ethical review

The trial protocol was reviewed and ethical permission granted by Camden and Islington NRES committee on 03/10/2012 reference numbers: 12/LO/1176 (Streamline C) and 12/LO/1177 (Streamline L).

RESULTS

Table 1 gives full demographics of the interviewed cohort. All but four patients completed the WB-MRI. There were no significant differences in age, gender, or area-based deprivation between those who volunteered for interviews (n=91), and those who declined (n=32); mean age: 65 vs. 64, $F_{(1,121)}$ <1, p=0.527; % male: 64% vs. 56%, X^2 (df=1, n=123) =0.562, p=0.453; % in deprivation categories (Fisher's Exact Test chi-square: 1.834, p=0.788). Similarly there were no significant demographic differences between patients who were (n=51) or were not (n=40) interviewed; mean age 65 vs. 64, $F_{(1,89)}$ <1, p=0.579; gender % male: 53.4% vs. 46.6%, X^2 (df=1, n=91)=0.437, p=0.508, % in deprivation categories (Fisher's Exact chi-square: 5.247, p=0.254). The majority of patients were interviewed over the phone (76%, n=39) and the median interval between WB-MRI and interview was 15 days (1-63 days).

Table 1: Demographic characteristics of interview participants, by trial stream

For peer review only - http://bmjopen.bmj.com/site/about/guidelines.xhtml

	'Streamline- L'* % (n=25)	'Streamline-C'* %(n=26)	Group differences
Gender			
Male	60 (15)	61.5 (16)	X^2=0.013, df=1,
Female	40 (10)	38.5 (10)	p=0.910
Mean age (years)	65 (Sd=11 years)	64 (sd=9 years)	t=0.261, df=49; p=0.795
Area Deprivation Score (quintile)			
Highest 1	52.0 (13)	30.8 (8)	X^2=5.459
2	32.0 (8)	23.1 (6)	df=2,
Mid to low 3-5	16.0 (4)	46.2 (12)	p=0.065
I/v method			
Face to Face	20.0 (5)	26.9 (7)	X^2=0.339, df=1;
Phone	80.0 (20)	73.1 (19)	p=0.560
Median Interval between interview and WB-MRI	15 days (6-43 days)	20 days (1-63 days)	U=270.00, n=51, p=0.299

*Streamline-L' -lung cancer, 'Streamline-C' –colorectal cancer

Was the WB-MRI scan a challenge? Diversity of experience and comparison with other scans and tests.

For peer review only - http://bmjopen.bmj.com/site/about/guidelines.xhtml

For peer review only

As part of the Streamline trials, all patients had WB-MRI as an additional 'trial' investigation alongside standard staging investigations. For patients recruited to 'Streamline-C', standard investigation included CT of the chest, abdomen and pelvis, with some undergoing an additional abdominal/ pelvic MRI. Patients recruited to 'Streamline-L' underwent at least a CT and PET-CT with a proportion undergoing bronchoscopy for tissue sampling.

WB-MRI was perceived to be more challenging than standard scans by many: *"I would say the most challenging of the MRI scans was the body scan"*s56 (60-69, male, Colorectal -'C'), like *"torture - medieval torture"*s12 (50-59,male,C) while CT was described as *"a walk in the park"*s42 (60-69, male, C), and *"very simple"*s35 (50-59, female, Lung – 'L'). PET-CT was described by some as *"so much easier"*s51 (70-79, female, L) and *"wasn't so unpleasant"* s12 (50-59, male, C). However the CT and PET_CT were not without challenges: for example some commented on the intravenous contrast administered during the CT scan *"you feel a bit, you know, woozy, funny, …and it did make me feel slightly off"* s63 (<50, male, C) and the perceived 'invasiveness' of the radiation exposure associated with CT and PET-CT: "*all that radiation dye in my system and everything…And it just disturbed me. Because I couldn't go near my niece and she's only six"* s54 (<50, female, L). Another patient described the bronchoscopy as more unpleasant *"I'd rather have them scans again than have that tube down my throat again, that was the worst thing, and I hated it"* s73 (60-69, male, L).

What was difficult about the WB-MRI?

Factors identified as difficult were: claustrophobia, physical discomfort, noise, scan duration, as well as the challenge of coping with emotions elicited during the scan such as fear /panic and isolation. There was a range of reaction intensity, with some reporting positive experiences (Table 2).

For peer review only - http://bmjopen.bmj.com/site/about/guidelines.xhtml

Table 2: Problems and the diversity of challenge experienced related to WB-MRI

Problem	No challenge/ enjoyable	Low challenge	Medium/ High	Did not complete
Enclosed space	*"I'm not claustrophobic …that [enclosed space] doesn't frighten me" s36 (50-59, male, L)*	*"Maybe in the back of my mind you're worried about it [enclosed space]" s5(60-69, female, C)*	*"Coffin came to mind…initially, you feel like 'Oh god, it's so close in here'" s26(60-69, female, L)*	**"***I had this sense of being in a sarcophagus" s17(60-69, male, L)*
Noise	*"I thought it like music really…and it relaxed me"* s64(80+, male, C)	*"the noise was a bit…disconcerting because it was quite loud and quite sort of clanking and banging… …and it took some time getting used to it"* s63(<50, male, C)	*"that's one of the worst ones that I've had to go through with the noise… I felt like something was going to fall off and hit me."* s54(<50, female, L)	

For peer review only - http://bmjopen.bmj.com/site/about/guidelines.xhtml

Scan duration/ discomfort	*"It's actually quite comfortable in there"* s87(60-69, male, L)	*"It was not excruciatingly painful. It just ached a bit, that is all."* s18(60-69, male, C)	*"My back was uncomfortable. My, the back of my neck was uncomfortable…" "my brain was saying, "You can't go much longer…* s56(60-69, male, C)	
Negative Emotions (e.g. anxiety, panic, shock, distress)	*"I mean, there wasn't, you know, anything in particular to be frightening [sic]"* s76(<50, male, C)	"*when I first went in there, I was quite shocked …. …I wondered what the hell was going on… after a while I quite enjoyed it"* s18(60-69, male, C)	*"when I first laid down in the scan I was frightened" s79(60-69, female, C)* and *"I'm lying there and all of a sudden, they put all these things on top of me… And I'm thinking, 'Oh, my God! …this is threatening'"*s53 (60-69, male, C)	*I don't know whether the people doing the scan knew I had lung cancer, I didn't feel any empathy or any sort of friendly attitude. I walked out of there feeling quite upset actually"* s30 (50-59,male, L)

For peer review only - http://bmjopen.bmj.com/site/about/guidelines.xhtml

Claustrophobia: patients described feeling trapped or buried in the WB-MRI "*it was the feeling of being sort of trapped because you can feel the machine all around your body*" s12 (50-59, male, C) and "*I had this sense of being in this sarcophagus*" s17 (60-69, male, L). Only one participant asked to terminate the scan early because of claustrophobia (s17). In comparison the CT scan was described as being like "*going through the big polo mint. And, therefore, it's not as much as like entombment*" s80 (60-69, male, C) and the PET-CT was described as *"not as claustrophobic. That one [MRI] is close to your face. And the other one [PET-CT] is a bit higher. So that, you know, that's not a big problem, really"* s73 (60-69, male, L).

Noise: Noise emitted from the MRI during data acquisition was aversive for some "*horrendous. It's horrible!* "s55 (60-69, female, L), but not all, *"it was quite good.… I would say, 'What's coming next? What's the next noise?' So, I enjoyed that"* s18 (60-69, male, C). The absence of noise was a notable feature of other scans e.g. "*PET scan isn't noisy*" s91 (60-69, male, L) and "*there wasn't all the noise. That's the only difference really*" s41 (60-69, male, L).

Scan duration and physical discomfort**:** The scan length differentiated WB-MRI from other tests "*the CT scan is very quick…you're in and out. Whereas, the MRI scan was very long*" s13 (60-69, female, C). However one patient saw the PET-CT scan duration times as equivalent "*if you took the PET scan, and take the waiting time [for radioactive glucose to circulate], and deduct that, timewise, they're much the same*" s49 (>80, male, L). Duration was linked to comfort: "*the back of my neck was uncomfortable, [the WB-MRI] maybe 15 minutes too long to be comfortable*" s56 (60-69, male, C). In contrast a patient without

For peer review only - http://bmjopen.bmj.com/site/about/guidelines.xhtml

discomfort viewed the long duration acceptable, *“I think it’s over one and a half hour or something like that, so it was alright for me… is comfortable”* s19 (60-69, male, C). Emotions elicited by WB-MRI: WB-MRI induced strong emotions in some: *“I was going to have a sort of panic attack and have to be taken out of the machine…this claustrophobia, the feeling of being...trapped, really” s12*(50-59, male, C). Worry and fear were also prompted by the novelty of the experience “*the oppression comes from the unknown”*s45 (70-79, male, C). Dealing with feelings of panic and fear was a challenge in itself and needed effort to overcome “*when I first laid down in the scan I was frightened…I was trying to put it [fear] out of my head and think positively*” s79 (60-69, female, C). In comparison “*they [CT and PET-CT] were slightly more easier to relax in that environment, not that I didn’t relax in the MRI scan too, it’s just that it took a bit more sort of power, more energy if you like, to relax*” s41, (60-69, male, L).

Cancer context: The WB-MRI was perceived to have the potential to either suggest a cancer diagnosis or reveal additional findings associated with a poorer prognosis and treatment implications *“I was lying in there and I was thinking, what if they say it's cancer? Then, what?”* s54 (<50, female, L) and *“whether they’d find … it has gone everywhere and you know nothing you can do, I suppose that was the main worry”* s60 (60-69, female, L). One patient had a dilemma whether to go ahead with the scan because of the possibility that its findings might mean he wasn’t offered treatment “*I was of two minds about the trial scan because I didn’t want the trial scan to say the cancer’s spread and they wouldn’t operate.”* s30 (50-59, male, L). However another patient reported relief when the scan confirmed that initial tumours were localised “*my biggest fear, I know I’ve got the cancer in the bowels. I’ve seen it myself on the colonoscopy. And I was worried that they might, it might just disguise I’ve got other cancers. So when he said straight away, there was nothing else and I feel*

For peer review only - http://bmjopen.bmj.com/site/about/guidelines.xhtml

relieved" s45 (70-79, male, C). There was also appreciation of having the WB-MRI because it did reveal spread "*The MRI actually showed up even more of what's going on with me and I think if I hadn't had it, they would…we still wouldn't know. Because they found traces in five other places I feel a lot better now having the MRI scan, knowing that they've picked up on all of it" and "without that, they would…I would just be having the chemo and not the full treatment that I need*" s25 (<50, female, L). The existential threat posed by a cancer diagnosis may have influenced how physical characteristics of the scan where interpreted, one patient described the enclosed experience as being *"It's like being in a coffin. You know, I did think that. It's like being in a coffin. But I wasn't. You just have to think that… Because if you've got something really, really bad, it would be like being in a coffin. Just like thinking, 'my God. This is going to be me'. You know. And I would have thought that could be quite scary*" s16 (60-69, male, C), at this point the patient, recently diagnosed with colorectal cancer became tearful. Another patient interpreted MRI sounds to indicate the presence of pathology *" And then you'll hear all these noises coming through you, so you wonder…, I thought, if the machine is clicking on, may be something is wrong there with me"* s13 (60-69, female, C).

Explaining differences in experiences (see Figure One)

Cancer type: Physical symptoms reported more frequently by those recruited to 'Streamline-L' were associated with discomfort, "*I found lying flat on my back, yes, very uncomfortable... I was coping okay for about 20, 25 minutes and then that's when this pressure or aching or uncomfortable feeling on my chest began to kick in"*s30 (50-59, male, L) and difficulty with breath holding "*they had to make me do it a few times because I couldn't hold it for the length of time they wanted me to … I've got COPD, whether that made it worse, I don't know"* s58 (60-69, male, L).

For peer review only - http://bmjopen.bmj.com/site/about/guidelines.xhtml

Existing musculoskeletal problems: Sometimes these made the WB-MRI more uncomfortable, but not for all: *"I do suffer a bit from back pain… My back started to sort of be pretty uncomfortable*" s42 (60-69, male, C), and *"I was worried about my neck being uncomfortable … because of arthritis but they managed to sort me out and then I managed to last the hour*"s18 (60-69, male, C).

Mental health co-morbidities: One patient with prior anxiety found the scan difficult, describing their anxiety as causing feelings of claustrophobia "*I'm not claustrophobic. I just felt that way because of the anxiety. When I get anxious, it feels like everything's just closing in on me"* s54 (<50, female, L).

Prior experiences: One patient terminated the scan because he didn't like being constrained and his aversion came as a surprise, *"a sense of being constrained and being strapped down which is a new one for me… I haven't encountered that situation before"*s17 (60-69, male, L). In contrast, another participant (s8: 60-69, male, C) who knew he suffered from severe claustrophobia requested a sedative and completed the scan with relative ease. Work experiences meant some patients were used to confined spaces and/or loud noises, *"it doesn't bother me. I've worked in pipes and tunnels and all sorts of places"*s91 (60-69, male, L) and "*if I wasn't in the trade I suppose that that could really freak you out because it's quite loud…"* s76 (<50, male, C). Vicarious experiences were sources of knowledge for patients without prior personal experience, and influenced expectations, sometimes reassuring "*I have a few friends and relatives… they have already done MRI, so I had something from them. this [is not] painful or anything like that. "* s19 (60-69, male, C); sometimes contrary to their own eventual experience, *"it was not as intimidating as I thought… I had heard people*

For peer review only - http://bmjopen.bmj.com/site/about/guidelines.xhtml

talking about going in to an MRI scanner, and they were telling me how nervous they were, and how they hated it…. And when I saw it, I thought, 'That's okay'" s18 (60-69, male, C).

Staff contact: Staff supported patients with verbal reassurance, information provision, as well as making them physically comfortable. For example, "*they told me all about it so I didn't get anxious. I knew what to expect"* s26 (60-69, female, L), and *"they managed to find something to put under my neck…they did put it in the right place under my neck and I was okay"* s18 (60-69, male, C). Staff communication acted as a distraction *"the person speaking keeps my mind occupied"* s26 (60-69, female, L). Some patients spoke of a sense of isolation when in the scanner and hearing a voice confirmed they were not alone *"just made me feel a bit confident that you wasn't on your own, you know?"* s58 (60-69, female, L). Patients varied in whether they felt they received enough information *"they told me everything I needed to know"* s76 (<50, male, C) versus *"there wasn't any [information]…that was the lacking part*" s35 (50-59, female, L). Whether staff were perceived to be reassuring was also important for some and a change in staff mid-way through the scan was detrimental: "*she was constantly talking to me, I was fine. But then … another lady took over and she didn't talk as much … …when I panicked"* s58 (60-69, female, L) and *"People I've been speaking with beforehand… I've actually built up the initial … trust… Then, suddenly, a complete stranger is now telling me to breathe in and out… then said, ' I should be coming down now to inject you with dye'…* "*In my mind, I thought the absolutely bizarre"* s80 (60-69, male, C). Failure to heed a patient preference influenced their decision to terminate the scan *"…they told me that they were going to inject dye. And once again I said 'don't put that cannula in my left arm' but she did and the vein collapsed. And I said to them 'I can't do any more now… I've had enough'"* s30 (50-59, Male, L).

For peer review only - http://bmjopen.bmj.com/site/about/guidelines.xhtml

Coping: Patients adopted various coping strategies, categorised into two main groups: 1) strategies for coping with distressing thoughts, emotional responses, or physical sensations, e.g. mental distraction and relaxation. *"Because I love Cyprus I was thinking of Cyprus the whole time. And it sort of takes your mind off of what's going"*s55 (60-69, female, L) and *"breathing, breathing…And you know trying to sort of just to calm…Because you can't help but get anxious"*s60 (60-69, female, L); and 2) Strategies related to motivation to complete the scan, such as focusing on benefit beliefs: "*I was so fed up with pain that I would have done…you know, any investigation was better… it was [WB-MRI] something to get me better"*s14 (60-69, female, C). *"I was laying there and I was thinking, 'Well, it's for my own benefit so I can live with that'"*s8 (60-69, male, C) and "*knowing I had cancer, you know, that's probably the most frightening thing that probably anyone could be told… So, this [WB_MRI]was all a culmination of things that's going to help me get better….The bed rocking a little bit, these loud noises really palled into insignificance because in my body now, I've got a nasty little house guest, which has now stayed, not welcome. I'm going to rid. And this is part of the mechanism to get rid…I was totally focused on the cancer. And these are the pictures that would help me get that done*" s80 (60-69, male, C).

Patients undergoing the WB-MRI scan were not only faced with the challenge of a potentially new technology, but were also coping with a possible or recent cancer diagnosis. For some, this influenced the challenge posed by the WB-MRI; "*it's been you know really anxious time. It's not just going to have an MRI. You obviously know something is wrong with and you're hoping that there's only going to be one thing wrong with you and then you get two things [tests prior to WB-MRI found a spine tumour in addition to lung cancer]. And all of a sudden, it's a completely different ballgame you know for me. About that stage, I had realised that I probably wasn't going to be able have the operation and just carry on with my*

For peer review only - http://bmjopen.bmj.com/site/about/guidelines.xhtml

life as normal. .... which is why I was finding it so hard to relax. It wasn't the MRI, it was just me. ...you're stressed out" s60 (60-69, female, L).

Willingness to have another WB-MRI Scan

All patients stated they would undergo another WB-MRI scan if the doctor recommended it, including the four patients who had requested scan termination. However this agreement was offered with varying enthusiasm; *"well if I have to....I don't like it one bit but if it has to be done"* s73 (60-69, male, L) compared with *"yes without hesitation"* s63(<50,male,C).

CONCLUSIONS

We aimed to describe patient experience and acceptability of WB- MRI as a potential replacement for the modalities currently used for staging lung and colorectal cancer. Patients recognised that WB-MRI was different from other scans although the extent of the challenge varied considerably. The majority completed WB-MRI with just four patients requesting premature termination; all were prepared to attempt future WB-MRI. Patients adopted a variety of coping strategies, these centred on the physical and emotional responses experienced during scanning, as well as focusing on beliefs that bolstered motivation to complete the scan.

To our knowledge, there are only two other comparable qualitative studies of patients' experiences of MRI (19;20). In common with our study the enclosed space, noise, physical discomfort and duration were identified as challenging, and the need for staff support was also highlighted. The importance of a "trustful dialogue" between radiographer and patients, to facilitate coping strategies (19), resonate with comments made by patients in our study.

For peer review only - http://bmjopen.bmj.com/site/about/guidelines.xhtml

Noise and confinement are the most frequently cited negative aspects of MRI in quantitative work published over the last 20 years (6;12). Other studies identify additional factors increasing the likelihood of premature scan termination, including acquisition position (head first, and/or prone); age and gender (middle age or female); prior experience (first scan or prior negative experience) and comorbidity (pain, anxiety) (6;13;26;27). Our results concur with some of these findings; we found some evidence that co-morbidity (e.g. anxiety and physical symptoms) increased scan challenge although there were contrary examples where musculoskeletal patients did not experience anticipated discomfort after adjustments were made by staff. Unlike others studies (e.g. (12;14)) prior experience (positive or negative) generally increased coping ability.

Our study adds to the existing literature. Our patient cohort were highly suspected or just diagnosed with cancer, and we were able to elicit influences of this on their experience of MRI. Furthermore the specific scan type under investigation (WB MRI) differs from simpler MRI scans such as knees and spine, based on its longer duration and need for receiver coils to cover the whole body. Patients described how the WB-MRI had the potential to reveal a cancer diagnosis or additional metastatic disease, with treatment implications. This caused anxiety and a dilemma as to whether to have the scan. However some patients also described relief and gratitude for having the scan either when it found no additional cancer or indeed when it diagnosed additional metastatic disease. The perceived length of the scan, together with its title of “whole body” may have added to patients perceptions about its ability to influence their treatment and prognosis. The WB-MRI scanner was likened to a coffin or sarcophagus and it is possible that these observations were more emotive because the patients were contemplating a life threatening diagnosis. Carlsson and Carlsson (19) reported greater fear when MRI was used to confirm serious illness. WB-MRI for cancer staging comes at a

For peer review only - http://bmjopen.bmj.com/site/about/guidelines.xhtml

point when patients are likely to be emotionally vulnerable, stretching coping resources for challenging medical procedures. However, while the implications of scanning patients with co-morbidities and emotional vulnerability should be acknowledged, it is important to note that most completed the WB-MRI.

Our study suggests that good staff communication could reduce anxiety and boost coping strategies by acting as a source of distraction, motivation and emotional reassurance. The varied experiences encountered underline the need for staff interaction to be tailored. Advanced staff training to build rapport can reduce MRI non-completion rates and increase patient satisfaction (28;29). A recent UK survey suggests that whilst strategies are in place to help reduce MRI related anxiety (commonly written leaflets, verbal pre-scan information, music during scanning, dedicated staff support), these are not optimised yet, with resource restrictions a potential barrier to implementation (30).

Study limitations

The generalisability of our results may be limited. WB-MRI was performed within the context of a research trial and some patients declined participation citing claustrophobia. Acceptability of WB-MRI may be lower than our study suggests. However the patients were from a wide age range and deprivation levels, and were interviewed from a number of different hospitals. As a result, a diverse spread of experiences were documented and included some who ultimately were unable to tolerate scanning.

Clinical implications

Our study confirms that WB-MRI can be a challenging experience and that staff support is important in modifying scan related stress. Our study also highlights that patient experience is

For peer review only - http://bmjopen.bmj.com/site/about/guidelines.xhtml

varied and for the most part, the scan was tolerated, suggesting WB-MRI could potentially replace existing modalities, assuming adequate diagnostic accuracy and cost-effectiveness. Experience of WB-MRI reflects the success of coping strategies adopted by patients and the quality of support they receive. Further research would facilitate understanding of the interrelationship between patients' experiences, the effectiveness of their coping strategies, and of the support received from staff such that patients and clinicians can benefit maximally from the diagnostic potential of WB-MRI.

Figure One: A model illustrating the factors that influence a patient's WB-MRI experience.

For peer review only

For peer review only - http://bmjopen.bmj.com/site/about/guidelines.xhtml

(1) Brocken P, Prins JB, Dekhuijzen PN et al.The faster the better?-A systematic review on distress in the diagnostic phase of suspected cancer, and the influence of rapid diagnostic pathways. *Psychooncology* 2012 Jan;21(1):1-10.

(2) Brocken P, van der Heijden EH, Oud KT et al.Distress in suspected lung cancer patients following rapid and standard diagnostic programs: a prospective observational study. *Psychooncology* 2015 Apr;24(4):433-41.

(3) Risberg T, Sorbye SW, Norum J et al.Diagnostic delay causes more psychological distress in female than in male cancer patients. *Anticancer Res* 1996 Mar;16(2):995-9.

(4) Ciliberto M, Maggi F, Treglia G, et al. Comparison between whole-body MRI and Fluorine-18-Fluorodeoxyglucose PET or PET/CT in oncology: a systematic review. *Radiol Oncol* 2013 Jul 30;47(3):206-18.

(5) Katz RC, Wilson L, Frazer N. Anxiety and its determinants in patients undergoing magnetic resonance imaging. *J Behav Ther Exp Psychiatry* 1994 Jun;25(2):131-4.

(6) MacKenzie R, Sims C, Owens RG et al. Patients' perceptions of magnetic resonance imaging. *Clin Radiol* 1995 Mar;50(3):137-43.

(7) McIsaac HK, Thordarson DS, Shafran R et al.Claustrophobia and the magnetic resonance imaging procedure. *J Behav Med* 1998;21(3):255-68.

(8) Melendez JC, McCrank E. Anxiety-related reactions associated with magnetic resonance imaging examinations. *JAMA* 1993 Aug 11;270(6):745-7.

(9) van MD, Klaming L, Weda H. Pinpointing moments of high anxiety during an MRI examination. *Int J Behav Med* 2014 Jun;21(3):487-95.

(10) Dewey M, Schink T, Dewey CF. Claustrophobia during magnetic resonance imaging: cohort study in 55,000 patients. *J Magn Reson Imaging* 2007;38(3):722-5.

(11) Eshed I, Althoff CE, Hamm B et al. Claustrophobia and premature termination of magnetic resonance imaging examinations. *J Magn Reson Imaging* 2007 Aug;26(2):401-4.

(12) Harris LM, Cumming SR, Menzies RG. Predicting anxiety in magnetic resonance imaging scans. *Int J Behav Med* 2004;11(1):1-7.

(13) Kilborn LC, Labbe EE. Magnetic resonance imaging scanning procedures: development of phobic response during scan and at one-month follow-up. *J Behav Med* 1990 Aug;13(4):391-401.

(14) Lukins R, Davan IG, Drummond PD. A cognitive behavioural approach to preventing anxiety during magnetic resonance imaging. *J Behav Ther Exp Psychiatry* 1997 Jun;28(2):97-104.

(15) Wiljer D, Walton T, Gilbert J et al.Understanding the needs of colorectal cancer patients during the pre-diagnosis phase. *J Cancer Educ* 2013;28(3):402-7.

For peer review only - http://bmjopen.bmj.com/site/about/guidelines.xhtml

(16) Lehto RH. Identifying Primary Concerns in Patients Newly Diagnosed With Lung Cancer. *Oncology Nursing Forum* 2011;38(4):440-7.

(17) Robb KA, Simon AE, Miles A et al.Public perceptions of cancer: a qualitative study of the balance of positive and negative beliefs. *BMJ Open* 2014;4(7):e005434.

(18) Martin MY., Fouad MN, Oster RA et al.What do cancer patients worry about when making decisions about treatment? Variation across racial/ethnic groups. *Support Care Cancer* 2014;22(1):233-44.

(19) Carlsson S, Carlsson E. 'The situation and the uncertainty about the coming result scared mebut interaction with the radiographers helped me through': a qualitative study on patients' experiences of magnetic resonance imaging examinations. *Journal of Clinical Nursing* 2013;22:3225-34.

(20) Tornqvist E, Mansson A, Larsson EM et al. It's like being in another world – patients' lived experience of magnetic resonance imaging. *Issues in Clinical Nursing* 2006;954-61.

(21) Taylor SA, Mallett S, Miles A, et al. Streamlining staging of lung and colorectal cancer with whole body MRI; study protocols for two multicentre, non-randomised, single-arm, prospective diagnostic accuracy studies (Streamline C and Streamline L). BMC Cancer 2017 May 2;17(1):299.

(22) Evans RE, Simon AE, Wardle J. Public perceptions of the harms and benefits of testicular cancer education: a qualitative study. Cancer Epidemiol 2010 Apr;34(2):212-9.

(23) Miles A, Wardle J, Atkin W. Receiving a screen-detected diagnosis of cancer: the experience of participants in the UK flexible sigmoidoscopy trial. Psychooncology 2003 Dec;12(8):784-802.

(24) McLennan D, Barnes H, Noble M et al. The English Indices of Deprivation 2010. London: Department for Communities and Local Government; 2011.

(25) Braun V, Clarke V. Using thematic analysis in psychology. Qualitative Research in Psychology 2006;3(2):77-101.

(26) Mohlman J, Eldreth DA, Price RB et al. Predictors of unsuccessful magnetic resonance imaging scanning in older generalized anxiety disorder patients and controls. *J Behav Med* 2012;35:19-26.

(27) Thorpe S, Salkovskis PM, Dittner A. Claustrophobia in MRI: the role of cognitions. *Magnetic Resonance Imaging* 2008;26:1081-8.

(28) Lang E V. A Better Patient Experience Through Better Communication. *J Radiol Nurs* 2012;31((4)):114-9.17(1):18-23.

(29) Lang E V, Ward C, Laser E. Effect of team training on patients' ability to complete MRI examinations. *Acad Radiol* 2010;17(1):18-23.

For peer review only - http://bmjopen.bmj.com/site/about/guidelines.xhtml

(30) Tischler V, Calton T, Williams M et al.Patient anxiety in magnetic resonance imaging centres: Is further intervention needed? *Radiography* 2008;14:265-6.

For peer review only

For peer review only - http://bmjopen.bmj.com/site/about/guidelines.xhtml

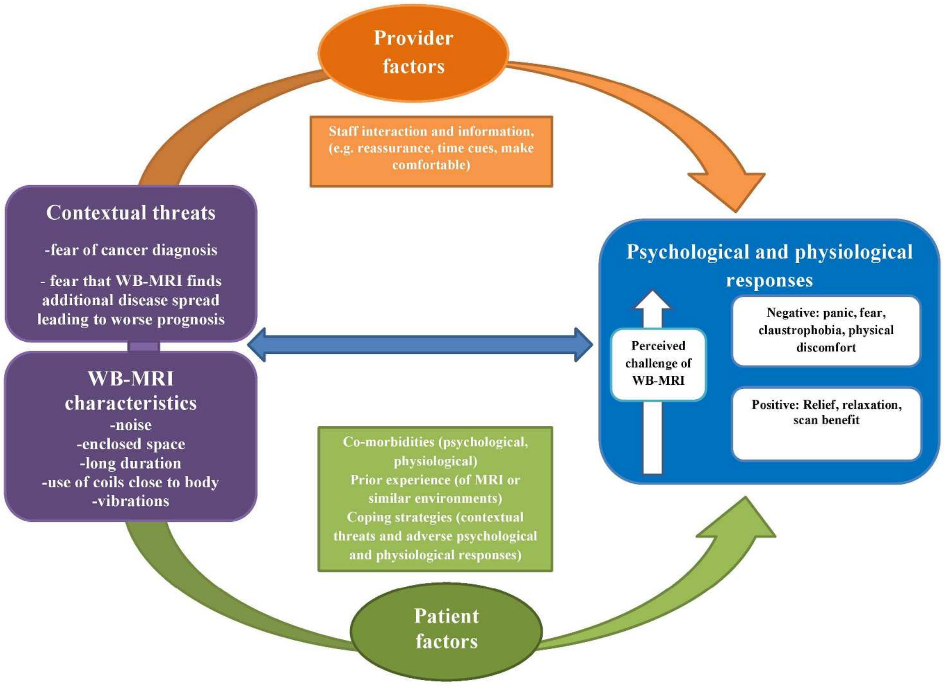

Figure 1: A model illustrating the factors that influence a patient's WB-MRI experience

297x210mm (300 x 300 DPI)

Streamline L

Streamlining Staging of Lung Cancer with Whole Body MRI

Trial Sponsor:	University College London (UCL)
Trial Sponsor reference:	UCL/12/0156
Trial funder:	NIHR HTA
Funder reference:	10/68/01
ISRCTN no:	50436483
Protocol version no:	6.0
Protocol version date:	06/09/2016

This project was funded by the National Institute for Health Research (NIHR) Health Technology Assessment (HTA) programme *(project number 10/68/01)*

For peer review only

Protocol version 6.0, 06/09/2016 Authorisation signatures

Name & Role:	Signature:	Date authorised:
**Chief Investigator:**<br>Professor Stuart Taylor<br>Professor of Medical Imaging, UCL	[illegible]	14/10/16
**For the Sponsor:**<br>Professor Jonathan Ledermann<br>Director, UCL CTC	[illegible]	14 OCT 2016
Sandy Beare<br>Tumour Group Lead, UCL CTC	[illegible]	14/10/2016

Please note: This trial protocol must not be applied to patients treated outside the Streamline L trial. UCL CTC can only ensure that approved trial investigators are provided with amendments to the protocol.

For peer review only - http://bmjopen.bmj.com/site/about/guidelines.xhtml

Coordinating Centre:

For general queries, supply of trial documentation and central data management please contact:

Streamline L Trial Coordinator
Cancer Research UK & UCL Cancer Trials Centre (UCL CTC)
90 Tottenham Court Road
London
W1T 4TJ

Tel: +44 (0) 20 7679 9878
Fax: +44 (0) 20 7679 9871
09:00 to 17:00 Monday to Friday

Email: ctc.streamlineL@ucl.ac.uk

Other trial contacts:

Chief Investigator: Professor Stuart Taylor
Address: Dept Specialist X Ray, Level 2 Podium,
University College London Hospital (UCLH)
235 Euston Road
London
NW1 2BU

Trial Management Group (TMG):

Prof Stuart Taylor	Professor of Medical Imaging	UCLH
Dr Dow-Mu Koh	Consultant Radiologist	The Royal Marsden
Prof Vicky Goh	Professor of Clinical Cancer Imaging	Guy's and St Thomas'
Prof Anwar Padhani	Clinical Lead in MRI	Mount Vernon
Dr Shonit Punwani	Senior Lecturer in Medical Imaging	UCLH
Prof Andrea Rockall	Professor of Cancer Imaging	Imperial College London
Prof Steve Halligan	Professor of Radiology	UCL
Dr Neal Navani	Consultant in Thoracic Medicine	UCLH
Prof Sam Janes	Wellcome Senior Fellow	UCLH
Dr John Bridgewater	Consultant Oncologist	UCLH
Alison Morton	Patient Representative	
Alf Oliver	Patient Representative	
Dr Anne Miles	Senior Lecturer in Health Psychology	Birkbeck College
Prof Steve Morris	Professor of Health Economics	UCL
Dr Susan Mallett	Senior Medical Statistician	University of Birmingham
Krystyna Reczko	Trial Coordinator	UCL CTC
Katie Prior	Data Manager	UCL CTC
Marian Duggan (maternity leave)	Senior Trial Coordinator	UCL CTC
Abby Sharp (maternity cover)	Senior Trial Coordinator	UCL CTC
Dr Sandy Beare	Tumour Group Lead	UCL CTC

For peer review only - http://bmjopen.bmj.com/site/about/guidelines.xhtml

Table of Contents

1. PROTOCOL SUMMARY 7
1.1. SUMMARY OF TRIAL DESIGN 7
1.2. TRIAL SCHEMA 9
2. INTRODUCTION 10
2.1. BACKGROUND 10
3. TRIAL OVERVIEW 12
3.1. PRIMARY OBJECTIVE 12
3.2. SECONDARY OBJECTIVES 12
3.3. TRIAL ACTIVATION 13
4. SELECTION OF SITES/SITE INVESTIGATORS 14
4.1. SITE SELECTION 14
4.1.1. Selection of Principal Investigator and other investigators at sites 14
4.1.2. Training requirements for site staff 14
4.2. SITE INITIATION AND ACTIVATION 15
4.2.1. Site initiation 15
4.2.2. Required documentation 15
4.2.3. Site activation letter 15
5. INFORMED CONSENT 16
6. SELECTION OF PATIENTS 18
6.1. PRE-REGISTRATION EVALUATION 18
6.2. SCREENING LOG 18
6.3. MULTI DISCIPLINARY (MDT) MEETING LOG 18
6.4. PATIENT ELIGIBILITY 18
6.4.1. Patient Inclusion Criteria 18
6.4.2. Exclusion Criteria 19
6.4.3. Pregnancy and Birth Control 19
7. REGISTRATION PROCEDURES 20
7.1. REGISTRATION 20
7.2. REGISTRATION PACKS 20
7.3. BOOKING WHOLE BODY MRI 20
8. PATIENT MANAGEMENT AND TRIAL PROCEDURES 22
8.1. SUMMARY 22
8.1.1. Staging and Treatment Decisions 22
8.1.2. Patient experience Interviews and Questionnaires (Optional) 23
8.1.3. Quality of Life Questionnaires and Patient Diaries 23
8.2. DETAILED DESCRIPTION 25
8.2.1. Standard care 25
8.2.2. Trial Imaging 25
8.2.3. Release of Conventional and trial WB-MRI findings 26
8.2.4. Final treatment plan 28
8.2.5. Early release of WB-MRI findings 28
8.2.6. Time to full staging 29
8.2.7. Patient follow up 29
8.2.8. Patient diaries and EQ-5D Quality of Life questionnaires 30
8.2.9. Final reference standard for tumour stage (Consensus Meetings) 30

For peer review only - http://bmjopen.bmj.com/site/about/guidelines.xhtml

8.2.10. *Health psychology assessment* ... *31*
8.2.11. *Cost Effectiveness Assessment* ... *34*
8.2.12. *Nested substudies* ... *36*
9. DATA MANAGEMENT GUIDELINES ... 38
9.1. COMPLETING CASE REPORT FORMS ... 38
9.2. MISSING DATA ... 38
9.3. TIMELINES FOR DATA RETURN ... 38
9.4. DATA QUERIES ... 39
10. SAFETY REPORTING ... 40
11. INCIDENT REPORTING ... 41
12. TRIAL MONITORING AND OVERSIGHT ... 42
12.1. CENTRAL MONITORING ... 42
12.2. 'FOR CAUSE' ON SITE MONITORING ... 42
12.3. OVERSIGHT COMMITTEES ... 43
12.3.1. *Trial Management Group (TMG)* ... *43*
12.3.2. *Trial Steering Committee (TSC)* ... *43*
12.3.3. *Independent Data Monitoring Committee (IDMC)* ... *43*
12.3.4. *Role of UCL CTC* ... *43*
13. WITHDRAWAL OF PATIENTS ... 44
14. TRIAL CLOSURE ... 45
14.1. END OF TRIAL ... 45
14.2. ARCHIVING OF TRIAL DOCUMENTATION ... 45
14.3. EARLY DISCONTINUATION OF TRIAL ... 45
14.4. WITHDRAWAL FROM TRIAL PARTICIPATION BY A SITE ... 45
15. QUALITY ASSURANCE ... 46
16. STATISTICS ... 47
16.1. SAMPLE SIZE CALCULATION ... 47
16.2. POPULATION FOR ANALYSIS ... 48
16.3. ANALYSIS OF THE PRIMARY ENDPOINT ... 49
16.4. ANALYSIS OF SECONDARY ENDPOINTS ... 50
16.4.1. *Efficacy (secondary)* ... *50*
16.4.2. *Safety* ... *51*
16.4.3. *Economic evaluation* ... *51*
16.4.4. *Health psychology assessment* ... *52*
16.4.5. *Handling missing data* ... *53*
16.5. INTERIM ANALYSES ... 53
17. ETHICAL APPROVALS ... 55
17.1. ETHICAL APPROVAL ... 55
17.2. SITE APPROVALS ... 55
17.3. PROTOCOL AMENDMENTS ... 55
17.4. PATIENT CONFIDENTIALITY & DATA PROTECTION ... 56
18. SPONSORSHIP AND INDEMNITY ... 57
18.1. SPONSOR DETAILS ... 57
18.2. INDEMNITY ... 57
19. FUNDING ... 58
20. PUBLICATION POLICY ... 59

For peer review only - http://bmjopen.bmj.com/site/about/guidelines.xhtml

21. REFERENCES ... 60
APPENDIX 1: ABBREVIATIONS ... 63
APPENDIX 2: CONSENT PROCESS ... 65
APPENDIX 3: MINIMUM WB-MRI PROTOCOL DATASET ... 67
APPENDIX 4: EXPLORATORY ANALYSIS ... 69
APPENDIX 5: PROTOCOL VERSION HISTORY ... 71

For peer review only

For peer review only - http://bmjopen.bmj.com/site/about/guidelines.xhtml

1. Protocol Summary

1.1. Summary of Trial Design

**Title:**	Streamlining Staging of Lung Cancer with Whole Body MRI
**Short Title/acronym:**	Streamline L
**Sponsor name & reference:**	UCL/12/0156
**Funder name & reference:**	NIHR HTA
**ISRCTN no:**	50436483
**Design:**	Multicentre comparison
**Overall aim:**	To evaluate whether early whole body magnetic resonance Imaging (WB-MRI) increases per patient sensitivity for metastasis in non-small cell lung cancer compared to standard NICE-approved diagnostic pathways.
**Primary endpoint:**	Per patient sensitivity for metastasis detection by whole body MRI (WB-MRI) compared to standard staging pathways in newly diagnosed non-small cell lung cancer
**Secondary endpoints:**	1. The time and test number taken to reach, and the nature of, the first major treatment decision based on WB-MRI in comparison to standard staging pathways.<br>2. Diagnostic accuracy of WB-MRI and conventional staging pathways for local tumour staging and detection of metastasis in comparison to an expert derived consensus reference standard.<br>3. Lifetime incremental cost and cost-effectiveness of staging using WB-MRI compared to standard diagnostic pathways.<br>4. Patient experience of staging using WB-MRI in comparison to standard diagnostic pathways and priorities placed by patients on differing attributes related to competing staging pathways.<br>5. Inter-observer variability in WB-MRI analysis and effect of diagnostic confidence on staging accuracy.<br>6. Diagnostic accuracy of limited T1 and diffusion weighted sequences compared to full multi-sequence WB-MRI protocols.
**Target accrual:**	353

For peer review only - http://bmjopen.bmj.com/site/about/guidelines.xhtml

**Inclusion & exclusion criteria:**	**Inclusion criteria:**<br>• Adult patients (18 or over) with suspected primary non-small cell lung cancer on chest CT with sufficient confidence to trigger staging investigations/biopsy OR with already histologically proven primary non-small cell lung cancer<br>• Disease is potentially radically treatable as defined as stage IIIb or less on diagnostic CT (i.e. T1-4, N0-2, M0)<br>• Performance status 0-2 (fit to undergo radical treatment if indicated)<br>• Patient must have given written informed consent and be willing to comply with the protocol intervention and follow up.<br>**Exclusion criteria:**<br>• Any psychiatric or other disorder likely to impact on informed consent<br>• Evidence of severe or uncontrolled systemic disease which makes it undesirable for the patient to participate in the trial<br>• Pregnancy<br>• Contraindications to MRI (e.g. cardiac pacemaker, severe claustrophobia, inability to lie flat)<br>• Unequivocal metastatic or N3 disease on diagnostic CT chest and abdomen (including M1a disease; malignant pleural effusion)<br>• Further staging work up not indicated in the opinion of the MDT due to poor performance status or patient choice<br>• Histologies other than non-small cell lung cancer.
**Planned number of sites:**	17 (including 7 Imaging hubs)
**Target Country**	UK
**Trial Procedure:**	All patients will undergo a whole body MRI protocol in addition to the standard staging protocol employed at their institution.
**Anticipated duration of recruitment:**	4 years
**Duration of patient follow up:**	12 months
**Definition of end of trial:**	12 months after enrolment of the final patient.
**Other related research:**	Sub-study of WB-MRI generalisability

For peer review only - http://bmjopen.bmj.com/site/about/guidelines.xhtml

1.2. Trial Schema

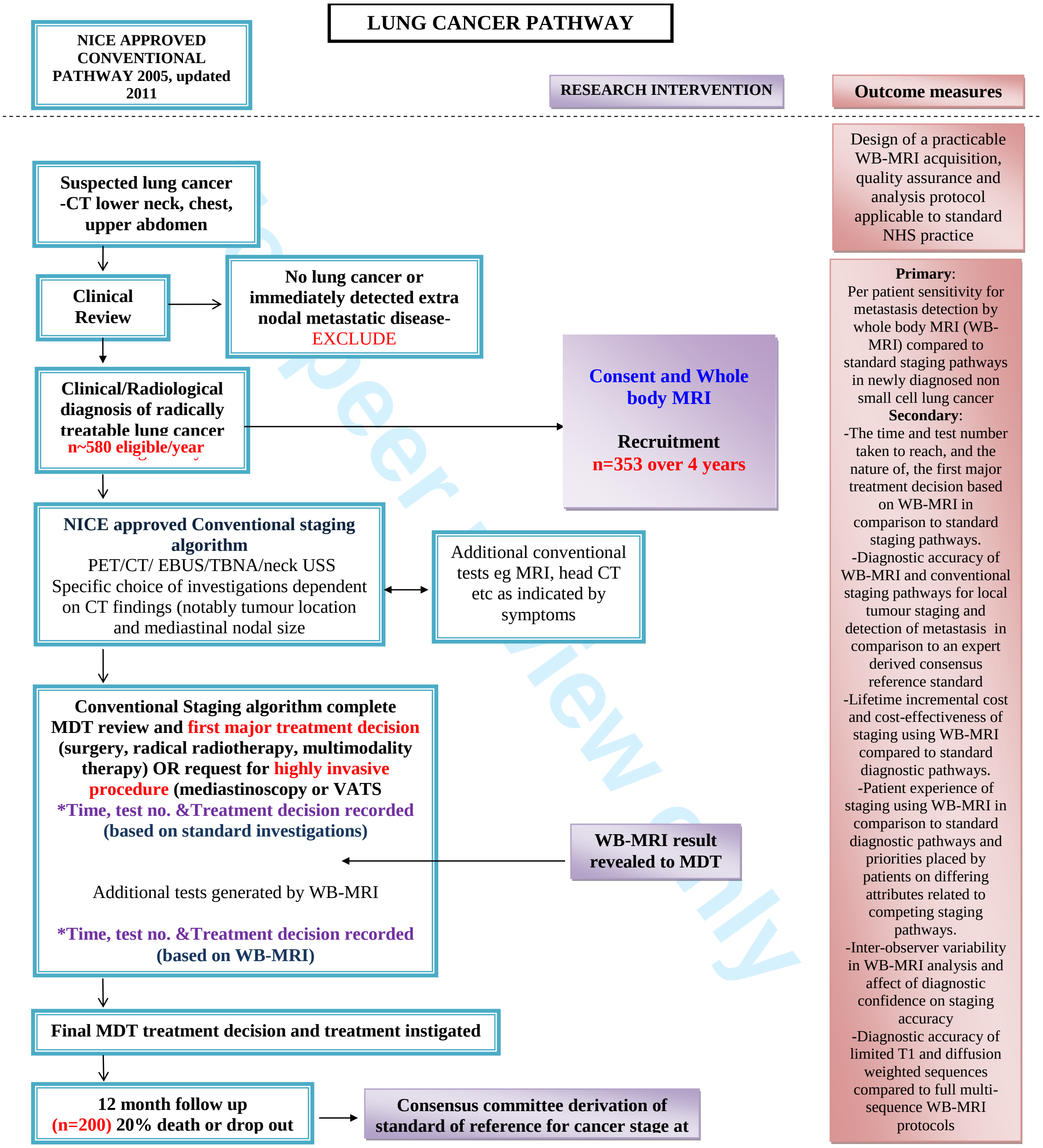

Key: EBUS= Endobronchial Ultrasound. TBNA= transbronchial needle aspiration. USS=ultrasound VATS= video-assisted thoracoscopic surgery

For peer review only - http://bmjopen.bmj.com/site/about/guidelines.xhtml

2. Introduction

2.1. Background

Lung cancer is the second most common malignancy in the UK and accounts for 13% of all new cancer diagnosis; approximately 40,000 patients are diagnosed per year [1]. Treatment decisions for lung cancer are critically dependent upon rapid and accurate tumour staging at diagnosis. In particular detection of metastatic disease (i.e. disease which has disseminated beyond the primary tumour into distant organ sites such as bone, liver and brain) is vital to optimised therapeutic triage. Although 1 year lung cancer survival in the UK is just 33% [2], correctly staged and treated early stage disease (i.e. without metastatic spread) achieves between 54 and 80% 5 year survival. Conversely, currently over 20% of patients undergoing "curative" thoracotomy for presumed early stage disease rapidly relapse with metastatic spread undetected by conventional staging tests - so called "futile thoracotomy" [3]. Accurate local staging of the tumour and spread to lymph nodes is also of importance. For example in patients with non-small cell lung cancer, spread to contra lateral mediastinal lymph nodes precludes surgery with curative intent.

Staging of lung cancer within the NHS is reliant on high technology imaging platforms such as computerised tomography (CT), positron emission tomography (PET) and magnetic resonance imaging (MRI), together with standard scintigraphy, plain X-Rays and ultrasound. Minimally invasive tissue sampling techniques such as endobronchial ultrasound (EBUS) are also increasingly used. Staging pathways are complex (see flowchart, section 1.2), and governed by NICE approved guidelines [2] which delineate an integrated step by step deployment of the various imaging modalities before tumour staging is deemed complete and the first treatment decision is made. The complexity of current staging algorithms reflects the differing diagnostic attributes of the various imaging modalities across tumour types and body organs. CT for example is superior to ultrasound for detecting liver metastasis [4], but inferior to scintigraphy in diagnosing malignant spread to the bone. PET scanning is in general superior to scintigraphy for detecting bone metastases [5], but not as effective as MRI in identifying spread to the brain [6]. It is not unusual for a patient newly diagnosed with lung cancer to undergo standard chest and abdominal CT, whole body PET, brain CT, and invasive mediastinal nodal sampling before the first definitive treatment is decided. Such a stepwise multi-modality approach is time and resource intensive and onerous for patients at a very difficult time following their cancer diagnosis. Furthermore patients receive significant doses of diagnostic ionising radiation during the staging process - a single PET-CT imparts 6-10 times normal annual background radiation and even small doses of radiation can increase an individual's risk of subsequent malignancy [7].

Recent technological advances mean an MRI scan of the whole body can be acquired in less than one hour; sensitivity for metastatic cancer spread is high [8]. MRI does not impart ionising radiation and is advocated as a safe and effective alternative to the current multi-modality approach. There is no available secondary research evidence concerning the accuracy of WB-MRI in cancer staging. Indeed meta-analysis would be challenging because literature is limited to relatively small cohort studies comparing WB-MRI with conventional imaging tests, and in a wide variety of cancers. These do however suggest potential as an efficient and accurate "one stop shop" alternative for

For peer review only - http://bmjopen.bmj.com/site/about/guidelines.xhtml

cancer staging [6, 9, 10]. For example, in 123 patients with non-small cell cancer, Yi found no difference between PET-CT and WB-MRI, both achieving 86% accuracy [10]. Notably, the vast majority of available studies are single site, using one MRI platform, with interpretation by one or two experienced radiologists. The literature is deficient regarding generalisability of WB-MRI. For example, variability amongst interpreting radiologists is unknown and the effect (if any) of different manufacturers' platforms is uncertain. It is also unclear how WB-MRI can be best integrated into existing NHS pathways, which frequently differ between institutions, or how effective it would be in different clinical settings. By undertaking a multi–centre trial of lung cancer using a variety of MRI platforms and interpreting radiologists, the current proposal will address these unknown variables.

Health psychology assessment

WB-MRI staging of lung cancer could reduce the physical and psychological burden associated with current pathways. By reducing time from diagnosis to treatment, WB-MRI may reduce patient anxiety, minimising concerns that treatment delay might adversely affect prognosis. Delay in diagnosis is known to be distressing, particularly for women [11]. In addition, staging scans can be uncomfortable and embarrassing [12]. MRI for example, while avoiding radiation, is noisy and claustrophobic. The potential of WB-MRI to generate additional tests is also an important consideration, although recent data suggest test sensitivity is the key determinant of patient choice and influences preference more than discomfort and complications [13]. While rapid staging means patients without metastasis are in receipt of good news more quickly, it also means those with metastasis will also receive bad news earlier, which may impact adversely on psychological wellbeing: It is known that patients are more depressed following a speedier diagnosis of breast cancer (at a one-stop clinic) when compared to those waiting a week [14].

Cost Effectiveness Analysis

Although WB-MRI is a relatively expensive imaging technology, other standard tests in the staging pathways such as PET-CT are also resource intensive. A detailed analysis of the cost and the cost-effectiveness of WB-MRI versus standard NICE-approved staging algorithms conforming to accepted economic evaluation methods [15] is essential to any trial investigating the role of WB-MRI in cancer staging.

For peer review only - http://bmjopen.bmj.com/site/about/guidelines.xhtml

3. Trial Overview

This trial will be an independent multi-centre prospective cohort trial comparing the staging accuracy of early WB-MRI with standard pathways in newly diagnosed non-small cell lung cancer. We will follow the recommendations of the STAndards for the Reporting of Diagnostic accuracy studies (STARD) initiative [16]. A key element of our design is the investigation of early WB-MRI as a replacement test for standard pathways [17], and data regarding the therapeutic impact of WB-MRI will be collated, as well as addressing the practicalities of general NHS implementation. The trial design, which compares two different staging strategies in the same patients ensures that clean, comparable data are collected for both pathways while simultaneously increasing trial power to meet trial endpoints.

In addition, both qualitative and quantitative assessments will be employed to determine the psychological burden and acceptability of WB-MRI versus standard pathways, and to identify those attributes which most strongly influence patient preference. Health-related quality of life data will be collected which will be used to inform cost-effectiveness modelling, and determine whether patients believe more rapid staging would have helped them cope better with their cancer diagnosis.

3.1. Primary objective

To evaluate whether early whole body magnetic resonance Imaging (WB-MRI) increases per patient sensitivity for metastasis in non-small cell lung cancer compared to standard NICE-approved diagnostic pathways.

3.2. Secondary objectives

1) To determine how WB-MRI influences time to and nature of first major treatment decision following definitive staging compared to standard investigations and to determine whether early WB-MRI could reduce or replace standard investigations.
2) To assess the accuracy of WB-MRI and standard diagnostic pathways for local and distant staging by comparison with an expert derived consensus reference standard using 1-year patient follow up data.
3) To evaluate the lifetime incremental cost and cost-effectiveness of staging using WB-MRI compared to standard diagnostic pathways.
4) To evaluate patients' experiences of staging using WB-MRI and to determine the priorities placed by patients on differing attributes related to competing staging pathways, including impact of reducing time to first treatment.
5) To determine the effect of radiologist confidence on the diagnostic accuracy of WB-MRI for metastatic disease.
6) To evaluate the diagnostic performance of limited WB-MRI protocols based on diffusion and T1 weighted imaging only, and to assess the incremental benefit of intravenous gadolinium contrast enhancement.

For peer review only - http://bmjopen.bmj.com/site/about/guidelines.xhtml

Additional objectives which will be met by the trial will be the definition of an efficient WB-MRI acquisition and analysis protocol, and quality assurance (QA) program applicable to standard NHS practice and collation of a database of WB-MRI datasets for NHS staff training and service development.

3.3. Trial activation

UCL CTC will ensure that all trial documentation has been reviewed and approved by all relevant bodies and that the following have been obtained prior to activating the trial:

- Research Ethics Committee approval
- 'Adoption' into NIHR portfolio
- NHS permissions
- Adequate funding for central coordination
- Confirmation of sponsorship
- Adequate insurance provision

For peer review only

For peer review only - http://bmjopen.bmj.com/site/about/guidelines.xhtml

4. Selection of Sites/Site Investigators

4.1. Site selection

In this protocol a 'recruitment site' refers to a hospital where certain trial-related activities are conducted.

Recruitment sites must be able to comply with:

- Patient recruitment, follow up schedules and all requirements of the trial protocol
- Requirements of the Research Governance Framework
- Data collection requirements
- **No** trial imaging, recruitment sites will refer patients to designated imaging hub (see below)

In this protocol an 'imaging hub' refers to a hospital where certain trial-related activities are conducted.

Imaging hubs must be able to comply with:

- Patient recruitment (where applicable), follow up schedules (where applicable) and all requirements of the trial protocol
- Requirements of the Research Governance Framework
- Data collection requirements
- Trial imaging

4.1.1. Selection of Principal Investigator and other investigators at sites

Sites must have an appropriate Principal Investigator (PI), i.e. a health care professional authorised by the site and ethics committee, to lead and coordinate the work of the trial on behalf of the site. Other investigators at site wishing to participate in the trial must be trained and approved by the PI. All investigators must be medical doctors and have experience of diagnosing, staging or treating lung cancer.

4.1.2. Training requirements for site staff

All site staff must be appropriately qualified by education, training and experience to perform the trial related duties allocated to them, which must be recorded on the site delegation log.

CVs for all staff must be kept up to date, with signed and dated and copies held in the Investigator Site File (ISF). An up to date, signed copy of the CV for the PI must be forwarded to UCL CTC upon request.

All staff involved in the trial must receive GCP training which is relevant to their role and responsibilities within the trial. The frequency of repeat training may be dictated by the requirements of their employing institution, or 2 yearly where the institution has no policy, and more frequently when there have been updates to the legal or regulatory requirements for the conduct of clinical trials.

For peer review only - http://bmjopen.bmj.com/site/about/guidelines.xhtml

4.2. Site initiation and activation

4.2.1. Site initiation

Before a site is activated, the UCL CTC trial team will arrange a site initiation with the site which the PI and site research team must attend. The site will be trained in the day to day management of the trial, and essential documentation required for the trial will be checked.

Site initiation will be performed for each site by site visit or teleconference.

4.2.2. Required documentation

The following documentation must be submitted by the site to UCL CTC prior to a site being activated by UCL CTC trial team:

- Trial specific Declaration of Participation/Site Registration Form (identifying relevant local staff)
- All relevant institutional approvals (e.g. local NHS permission)
- A completed site delegation log that is initialled and dated by the PI
- A copy of the PI's current CV that is signed and dated

In addition, the following agreement must be in place:

- A signed Clinical Trial Site Agreement (CTSA) between the Sponsor and the relevant institution (usually a NHS Trust)

4.2.3. Site activation letter

Once the UCL CTC trial team has received all required documentation and the site has been initiated, a site activation letter will be issued to the PI. **Sites may not begin to approach patients until the site activation letter has been issued.**

Once the site has been activated by UCL CTC, the PI is responsible for ensuring:

- adherence to the most recent version of the protocol;
- all relevant site staff are trained in the protocol requirements;
- appropriate recruitment and medical care of patients in the trial;
- timely completion and return of CRFs (including assessment of all adverse events).

For peer review only - http://bmjopen.bmj.com/site/about/guidelines.xhtml

5. Informed consent

Sites are responsible for assessing a patient's capacity to give informed consent.

Sites must ensure that all patients have been given the current approved version of the patient information sheet, are fully informed about the trial and have confirmed their willingness to take part in the trial by signing the current approved consent form.

Sites must assess a patient's ability to understand verbal and written information in English and whether or not an interpreter would be required to ensure fully informed consent. If a patient requires an interpreter and none is available, the patient should not be considered for the trial.

The PI, or, where delegated by the PI, other appropriately trained site staff, are required to provide a full explanation of the trial to each patient prior to trial entry. During these discussions, the current approved patient information sheet for the trial should be discussed with the patient. A minimum of 24 hours before the WB-MRI must be allowed for the patient to consider and discuss participation in the trial.

The consent process has been left deliberately flexible in order to accommodate the needs of individual patients and variations in Site requirements. The flowchart below describes three different options for obtaining written informed consent from patients, each of which ensures that patients have at least 24 hours to consider their participation in the trial before any trial-related activities take place.

Streamline Recruitment Pathways/Consent Process

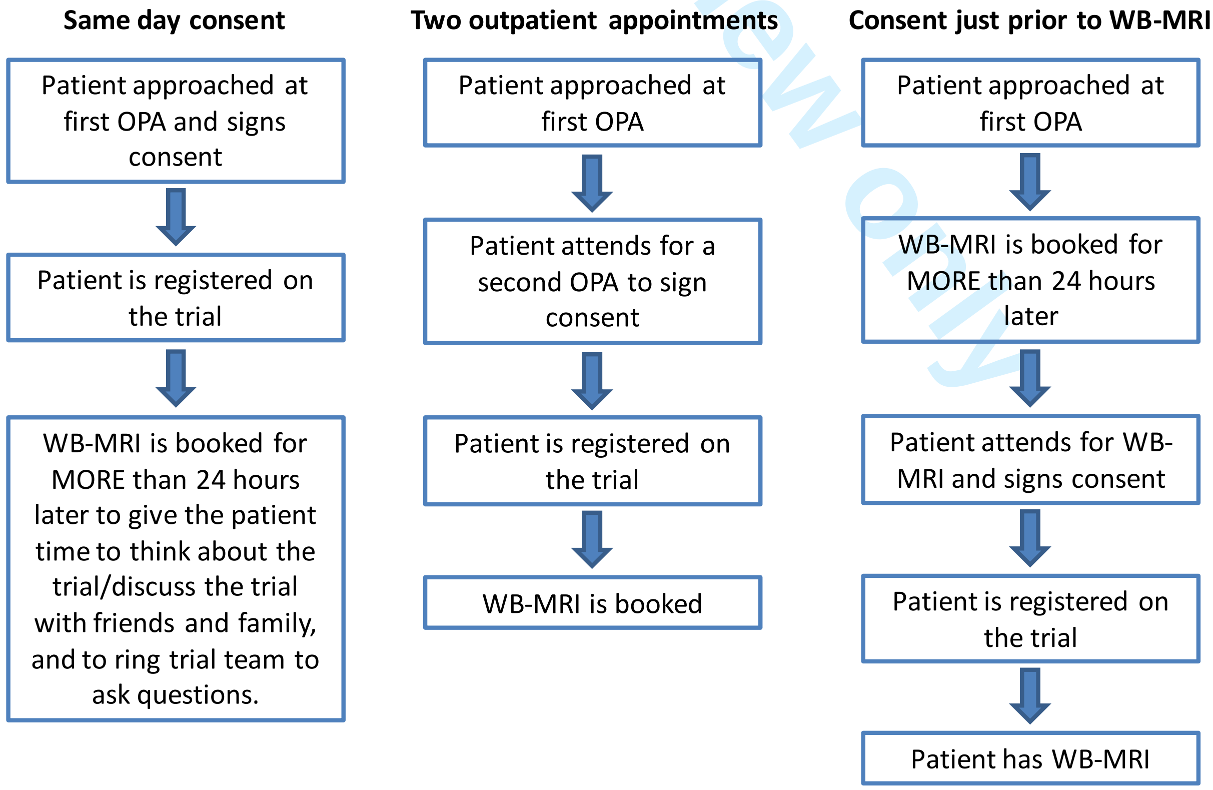

For peer review only - http://bmjopen.bmj.com/site/about/guidelines.xhtml

See Appendix 2 for a more detailed example of the consent process.

Written informed consent on the current approved version of the consent form for the trial must be obtained before any trial-specific procedures are conducted. The discussion and consent process must be documented in the patient notes.

Site staff are responsible for:

- checking that the correct (current approved) version of the patient information sheet and consent form are used;
- checking that information on the consent form is complete and legible;
- checking that the patient has completed/initialled all relevant sections and signed and dated the form;
- checking that an appropriate member of staff has countersigned and dated the consent form to confirm that they provided information to the patient;
- checking that an appropriate member of staff has made dated entries in the patient's medical notes relating to the informed consent process (i.e. information given, consent signed etc.);
- Following registration:
    - adding the patient trial number to all copies of the consent form, which should be filed in the patient's medical notes and investigator site file,
    - giving the patient a copy of their signed consent form and patient information sheet.

The right of the patient to refuse to participate in the trial without giving reasons must be respected. All patients are free to withdraw at any time. Also refer to section 13.0 (Withdrawal of patients).

For peer review only - http://bmjopen.bmj.com/site/about/guidelines.xhtml

6. Selection of Patients

6.1. Pre-registration Evaluation

The following assessments or procedures are required to evaluate the suitability of patients for the trial:

- Histologically proven or clinically diagnosed (defined as radiological diagnosis of lung cancer on chest CT with sufficient confidence to trigger staging investigations) primary non-small cell lung cancer with potentially radically treatable disease
- No contraindications to MRI (e.g. cardiac pacemaker, severe claustrophobia, inability to lie flat)

6.2. Screening Log

A screening log must be maintained by the site and kept in the Investigator Site File. This must record all potentially eligible patients approached about the trial and the reasons why they were not registered in the trial if this is the case. The log must be sent to UCL CTC in line with the monitoring plan and as requested, with patient identifiers removed prior to sending.

6.3. Multi Disciplinary (MDT) Meeting Log

An anonymous record of the number of patients with a new diagnosis of non-small cell lung cancer discussed in the MDT meeting, with their attributed cancer stage will be submitted annually to UCL CTC. This will be used to assess whether the group of patients recruited to the trial is representative of the general population of newly diagnosed lung cancer patients seen at Sites.

6.4. Patient Eligibility

There will be no exception to the eligibility requirements at the time of registration. Queries in relation to the eligibility criteria must be addressed prior to calling/faxing for registration. Patients are eligible for the trial if all the inclusion criteria are met and none of the exclusion criteria applies.

6.4.1. Patient Inclusion Criteria

- Adult patients (18 or over) with suspected primary non-small cell lung cancer on chest CT with sufficient confidence to trigger staging investigations/biopsy OR with already histologically proven primary non-small cell lung cancer
- Disease is potentially radically treatable as defined as stage IIIb or less on diagnostic CT (i.e. T1-4, N0-2, M0)
- Performance status 0-2 (fit to undergo radical treatment if indicated)
- Patient must have given written informed consent and be willing to comply with the protocol intervention and follow up.

For peer review only - http://bmjopen.bmj.com/site/about/guidelines.xhtml

6.4.2. Exclusion Criteria

- Unequivocal metastatic or N3 disease on diagnostic CT chest and abdomen (including M1a disease; malignant pleural effusion)
- Further staging work up not indicated in the opinion of the MDT or clinician due to poor performance status or patient choice.
- Histologies other than non-small cell lung cancer
- Any psychiatric or other disorder likely to impact on informed consent
- Evidence of severe or uncontrolled systemic disease which makes it undesirable for the patient to participate in the trial
- Pregnancy
- Contraindications to MRI (e.g. cardiac pacemaker, severe claustrophobia, inability to lie flat)

6.4.3. Pregnancy and Birth Control

MRI poses a theoretical risk to the foetus, particularly in the first trimester due to local acoustic and heating effects. However the risk is generally deemed very small and significantly less than the risk of ionising radiation imparted by PET-CT and CT (standard imaging investigations).

A woman of childbearing potential (WCBP) is a sexually mature woman (i.e. any female who has ever experienced menstrual bleeding) and who has not undergone a hysterectomy or who has not been postmenopausal for 24 consecutive months (i.e. who has had menses at any time in the preceding 24 consecutive months). Patients will be excluded if they are pregnant.

There is no requirement for additional contraceptive advice to patients over and above that routinely given as part of their routine clinical care given their diagnoses of lung cancer.

The need to perform a pregnancy test in WCBP will be decided as part of the patient's routine clinical care given the risk to pregnancy from standard staging investigations and subsequent treatment. There is no requirement to perform a pregnancy test purely because of recruitment to the trial if this would not have been performed as part of standard clinical care.

For peer review only - http://bmjopen.bmj.com/site/about/guidelines.xhtml

7. Registration Procedures

7.1. Registration

Patient registration will be undertaken centrally at UCL CTC and this must be performed prior to commencement of any trial intervention. Registration can be performed via telephone or fax.

Following pre-registration evaluations (as detailed in section 6.1), confirmation of eligibility and consent of a patient at a site, the registration form must be fully completed prior to faxing or telephoning UCL CTC. The eligibility criteria will be reviewed during the registration telephone call using the same form at UCL CTC.

A trial number will be assigned for the patient during the call and must be recorded at site by the caller. If desired, the site may fax a copy of the completed registration form to UCL CTC and the faxed registration from will be used to confirm patient eligibility at UCL CTC.

UCL CTC will email confirmation of the patient's inclusion in the trial and their trial number to the main contact, PI and Imaging Hub contact. If requested, a fax confirmation may also be sent. Case report forms will be emailed to the main contact at site.

Registration telephone number:	+44 (0)20 7679 9880
Registration fax number:	+44 (0)20 7679 9871
UCL CTC Office hours:	09:00 to 17:00 Monday to Friday

7.2. Registration Packs

Once a patient has consented to take part in the trial they should be provided with a registration pack to include the following:

- A copy of their signed consent form and patient information sheet
- An expenses claim form (to claim travel expenses to and from WB-MRI appointment)
- Pre-paid return envelope

7.3. Booking Whole Body MRI

Once a patient has been registered it is the responsibility of the recruiting site to contact their designated imaging hub to make arrangements for the WB-MRI. Each recruitment site will be provided with a contact number for their designated imaging hub. Recruitment sites will approach consecutive potentially eligible patients until the number of recruited patients fills the MRI capacity of the imaging hub.

For peer review only - http://bmjopen.bmj.com/site/about/guidelines.xhtml

It is possible recruitment potential at recruitment sites will be greater than the WB-MRI capacity at each central imaging hub. If a patient is approached and registered for the trial but MRI capacity is such that the scan could not take place in a timely fashion, a decision to withdraw the patient will be made by the recruitment site and the patient will then be informed. This possibility is explained to patients in the patient information sheet.

Because of the need to perform WB-MRI in a timely fashion, it is anticipated recruitment sites will be requested to pause recruitment if WB-MRI waiting time exceeds 2 weeks (if WB-MRI is performed after conventional imaging is complete) or 3 weeks (if WB-MRI is to be performed concurrently with standard tests). There will be flexibility in these timings depending on the circumstances of individual patients and their anticipated time to complete standard staging.

For peer review only - http://bmjopen.bmj.com/site/about/guidelines.xhtml

8. Patient Management and Trial Procedures

8.1. Summary

8.1.1. Staging and Treatment Decisions

The following information is required for the trial to document staging and treatment decisions:

- Stage and treatment decision based on conventional imaging only (and the number, timing, nature and findings of these investigations)
- Stage and theoretical treatment decision based on WB-MRI (and any additional tests generated by the WB-MRI) only (and the number, timing, nature and findings of additional tests generated)
- A final treatment decision incorporating all available tests.

Conventional Imaging

Trial patients will undergo the conventional staging protocol employed at their institution as per usual clinical care pathways. See section 8.2.1 for further detail.

Whole Body MRI Imaging and MDT Reveal

Recruited patients will undergo a WB-MRI at one of the designated imaging hubs. Recruiting sites should contact their designated imaging hub to request a WB-MRI slot (see section 7.3). The WB-MRI should be performed either concurrently with the standard staging investigations, or no later than 3 weeks after the final standard staging investigation. Images will be uploaded at each hub to a secure central imaging server called 3Dnet™. If a language interpreter was required for a patient during the consent process, then an interpreter should be made available to the patient during the WB-MRI. The WB-MRI will be reported by designated radiologist(s) at each imaging hub who are blinded to the conventional imaging tests and other clinical information (other than the cancer diagnosis and location). A copy of the report will also be uploaded to 3Dnet™. See section 8.2.2 for further detail.

Once the MDT has made a treatment decision based on the conventional imaging the MDT coordinator, research nurse or other designated individual will reveal the WB-MRI images and report on 3Dnet™ via an internet-enabled PC. The MDT will review the WB-MRI results and decide whether or not any additional tests are required or theoretically would have been required (if in fact the test was already performed as part of conventional staging) in addition to the WB-MRI. If any tests are required that have not already been performed as part of the standard staging, they should be requested and the patient reviewed again at the next MDT when the results are available. If no further tests are needed then the stage and theoretical treatment decision based on the WB-MRI (and the results of any actual or theoretical additional tests generated from the WB-MRI) must then be made by the MDT. The MDT must then decide a final treatment decision for the patient based on all imaging available.

If a patient is due to start treatment before the next formal MDT meeting, an ad hoc mini MDT meeting may be scheduled outside of the standard MDT meeting. This should include the relevant multidisciplinary clinical team members who are appropriate

For peer review only - http://bmjopen.bmj.com/site/about/guidelines.xhtml

to make the final treatment decision based on the specific imaging finding, according to usual standards of clinical care. A record will be kept of who is present during the ad hoc mini MDTs on the CRFs.

8.1.2. Patient experience Interviews and Questionnaires (Optional)

Interviews (Study 1)

A total of 25 patients have undergone interviews by the Health Psychologist pertaining to their experience of the cancer staging process. **Target accrual has now been reached for the interview part of the trial and recruitment to this is now closed**.

Questionnaires (Study 2)

Patient experience questionnaires from baseline and post-staging have been received for at approximately 50 patients to assess patient experience and acceptability of WB-MRI and standard tests. **Target accrual has now been reached for the questionnaire part of the trial and recruitment to this is now closed.**

Discrete choice experiment (Study 3)

A discrete choice experiment (DCE) questionnaire will be administered to a subset of approximately 50 patients to elicit their preference for various attributes associated with conventional and WB-MRI staging pathways. The DCE questionnaire is optional and patients will be asked to consent to this on trial entry. Once a patient consents to the DCE they will be allocated a DCE questionnaire (out of a possible 2 DCE questionnaires) which will be posted by UCL CTC along with their Quality of Life questionnaire, patient diary and pre-paid envelope immediately after registration (baseline). UCL CTC will supply the details of any recruited patients who consented to complete DCE questionnaire to the Health psychologists who may contact them directly.

8.1.3. Quality of Life Questionnaires and Patient Diaries

Upon consent the recruiting site must inform the patient that they will receive an EQ-5D Quality of Life Questionnaire and Patient Diary through the post from UCL CTC and they are to complete these and return them using the pre-paid envelope provided. The patient diary will cover a 3 month period and will collect information regarding the patient's Primary Health Care contacts, other health care contacts and also the medication they are currently taking. Patient diaries and EQ-5D questionnaires will be posted to all patients at consent and then at 3 monthly intervals for 12 months.

For peer review only - http://bmjopen.bmj.com/site/about/guidelines.xhtml

Table 1: Summary of patient management and trial activities

Activity	Protocol section	Timing							Responsible organisation/person
		Prior to registration	Immediately after registration	During staging	After staging (months after registration)				
					1	3	6	9	
Pre-registration evaluation & assessment of eligibility	6.1	X							Recruiting site
Consent	5.0	X							Recruiting site
Register patient	7.1	X							Recruiting site
Give patient registration pack (copy of PIS and consent, expenses claim form and pre-paid return envelope)	7.2		X						Recruiting site
Book WB-MRI scan & inform patient of appointment date	7.3		X						Recruiting site
WB-MRI	8.2.2			X					Imaging Hub
Upload WB-MRI scan & report	8.2.2			X					Imaging Hub
Post EQ-5D questionnaires	8.2.7		X			X	X	X	UCL CTC
Post patient resource diary	8.2.7		X			X	X	X	UCL CTC
Discrete choice experiment Questionnaire	8.2.10		X						UCL CTC

For peer review only

For peer review only - http://bmjopen.bmj.com/site/about/guidelines.xhtml

8.2. Detailed description

8.2.1. Standard care

Trial patients will undergo the conventional staging investigations employed at their recruiting institution according to local protocols and the requirements of their clinical care team.

All conventional investigations will be performed and interpreted by the usual radiologists and clinicians employed at the site of the investigations. Standard clinical reports will be made and all investigations (and their results) will be freely available on hospital Picture Archiving and Communications System (PACS), Radiology Information System (RIS) and Clinical Data Repository (CDR) systems as per usual clinical practice.

The type and date of investigations (e.g. CT scan, PET-CT, organ specific MRI, biopsy etc.) will be recorded on CRFs, along with the presence and location of metastatic disease based on the radiological report.

8.2.2. Trial Imaging

Whole Body MRI (WB MRI) protocol

Recruited patients will undergo a WB-MRI at their designated imaging hub site. The choice of MRI platform (i.e. manufacturer and Tesla (T) strength) will be decided by the local hub radiologist according to scanner availability and their usual practice. It is anticipated most MRIs will be performed at 1.5T. Exact imaging parameters will vary according to MRI platform but a minimum dataset of sequences will be acquired (full details given in appendix 3).

Timing

The WB-MRI will be performed either concurrently with the standard staging investigations, or no later than 3 weeks after their final standard staging investigation.

Blinding

To ensure the integrity of the trial, the WB-MRI must be reported by a radiologist blinded to the conventional imaging tests and other clinical information (other than the cancer diagnosis and site). Conversely the WB-MRI images and reports must be blinded to radiologists reporting the standard staging investigations, and those involved in direct patient care before the WB-MRI is revealed in the MDT.

The un-anonymised WB-MRI images will therefore not be immediately sent to the Picture Archive and Communication System (PACS) at either the imaging hub or recruitment sites. Instead images will be uploaded to a secure central imaging server (3Dnet™) provided by Biotronics3D. This solution allows easy upload of MRI datasets via standard internet connection. A PC based internet gateway will be installed in each imaging hub to facilitate automated transfer of WB-MRI from the scanner/workstation to 3Dnet™), and thereafter automatically back to PACS to the appropriate time point after MDT revelation (see below).

For peer review only - http://bmjopen.bmj.com/site/about/guidelines.xhtml

Interpretation and reporting

The WB-MRI will be interpreted by designated radiologist(s) at each imaging hub who are expert in interpretation based on previous experience of reporting WB-MRI in cancer staging. As noted above, the radiologist will be blinded to the standard staging investigations performed on recruited patients.

Interpretation can be performed using the 3Dnet™ software, or stand alone workstation according to the preference of the radiologist.

Images will be analysed in the following order:

1) Diffusion and non contrast enhanced T1 images
2) Diffusion, non contrast enhanced T1 and T2 images
3) Diffusion, non contrast enhanced T1, T2 and contrast enhanced T1 images.

After viewing each sequence set (and before reviewing the next set), the radiologist will complete WB-MRI Imaging Booklet CRFs documenting the presence, location and size of metastatic disease, together with their diagnostic confidence on a scale of 1 to 6. Reporting time for each sequence set analysis (defined as the time required to interrogate the sequences to reach a diagnostic conclusion) will be recorded along with the technical quality of the MRI dataset.

The reporting radiologist will then produce a free text clinical report as per their usual clinical practice (using all available sequences and based on the TMN 7 staging guidance) for subsequent release to the clinical team. This report will contain information relating to the local T and N stage of the tumour, together with the presence, location, number and size of metastatic deposits, as well as important "incidental" findings, for example aortic aneurysm. The radiologist may express their level of confidence in reported findings as they would in normal clinical practice but a formal numerical score of confidence will not be provided as this would not mirror how radiological examinations are reported in standard clinical care. If the radiologist would usually recommend additional tests for equivocal findings, this will be included in the report so as to also mirror routine clinical practice. The definitions of T, N M stage will be based on conventional MRI criteria adopted by the radiologist in their usual clinical practice.

The free text report will be uploaded onto the 3Dnet™ software, and a copy stored as part of the patient's medical record.

8.2.3. Release of Conventional and trial WB-MRI findings

MDT discussion of the first major treatment decision based on conventional imaging

WB-MRI images and reports will be withheld initially from the clinical care team and radiologists reporting standard imaging (to avoid bias) until patients have completed all conventional investigations and have been definitively staged such that a first major treatment decision has been made by the MDT based on these conventional tests. The first major treatment decision based on conventional imaging will be defined as:

- Referral for surgical excision of either the primary tumour and/or a metastatic site

For peer review only - http://bmjopen.bmj.com/site/about/guidelines.xhtml

- Instigation of definitive treatment using chemotherapy, radiotherapy or a combination of the two
- Decision to offer palliative/supportive care only
- Request for a highly invasive surgical staging procedure such as surgical mediastinal lymph node sampling (mediastinoscopy), video-assisted thoracoscopic surgery (VATS), or laparoscopy

If additional conventional tests are required to complete staging, the WB-MRI result will be withheld until they are completed, and the patient re-discussed with the findings of the additional test(s).

The nature, date and findings of all standard investigations will be recorded by the MDT, and the TNM 7 stage recorded. The MDT will define the nature of the first treatment decision based on conventional investigations in the MDT record and on the CRF.

Release of WB-MRI images and report

Once a stage and treatment decision has been made based on conventional imaging, the WB-MRI images (supplemented where possible with "screen shots" and annotations of relevant findings) and radiologist's report will be revealed to the MDT using the 3Dnet™ software on an internet enabled PC. If technical issues preclude use of 3Dnet™ in the MDT, presentation of a written copy of the WB-MRI report is permissible.

When the WB-MRI images and report are released, the MDT will discuss the images and decide:

1) *If the patient can be adequately staged based on WB-MRI alone:*

   In this case the MDT should state the patient's stage and theoretical treatment decision based on the WB-MRI alone.

Or:

2) *If any additional tests are required based on the WB-MRI alone for adequate staging* (for example for equivocal WB-MRI findings):
   a. For additional tests that would have theoretically been requested based on the WB-MRI alone but have already been performed as part of the standard imaging, the results can be used by the MDT. The MDT personnel will state the patient's stage and theoretical treatment decision based on the WB-MRI and those additional tests that would have theoretically been generated.
   b. If the additional tests generated by WB-MRI have not already been performed as part of standard care and are deemed necessary in the opinion of the MDT these will be undertaken according to standard clinical care. After completion of the additional test(s), the patient will be re-discussed by the MDT and a final treatment decision made based on the WB-MRI and the additional test(s) undertaken.

For peer review only - http://bmjopen.bmj.com/site/about/guidelines.xhtml

8.2.4. Final treatment plan

Once a stage and treatment decision has been made based on conventional imaging and also a stage and theoretical treatment decision made based on the WB-MRI alone (and any additional tests) then a final treatment plan can be made by the MDT. The final treatment plan for the patient will be based on all available information including the WB-MRI. The MDT radiologist(s) will be at liberty to review the WB-MRI data after the WB-MRI has been revealed in order to review its findings and feedback to the clinical care team.

The completed MDT CRF will thus list:

- Stage and treatment decision based on conventional investigations (and the number, timing, nature and findings of these investigations).
- Stage and theoretical treatment decision based on WB-MRI (and any additional tests generated by the WB-MRI) alone (and the number, timing, nature and findings of additional tests generated).
- A Final treatment decision incorporating all available tests.

Once the WB-MRI images and report have been revealed to the MDT according to trial protocol, they can be released onto the relevant hospital PACS server using the 3Dnet™ gateway described above or other IT solution (e.g. image exchange portal) and be made freely available to those providing subsequent patient care, as per usual clinical practice.

8.2.5. Early release of WB-MRI findings

Any requests for early WB-MRI release must be made to the site PI, who will discuss the request with the lead clinician who is responsible for the patient’s clinical care (if the request did not originate from the lead clinician). After discussion, if it is decided by the PI that release of the WB-MRI images and report is necessary, the PI (or a designated member of the trial team) will inform UCL CTC. The WB-MRI result should only be released early in the event of an emergency situation in which the patient is unfit to undergo additional investigations (including MRI) which would have normally been performed were the patient not in the Streamline L trial, and the WB-MRI may have a direct effect on immediate patient care. In the event of early release, the patient will be replaced, although the findings of the WB-MRI and conventional staging (performed up to the time of early MRI release) will be collated by UCL CTC on the standard trial CRFs for subsequent reporting in the trial publications.

Early release for important clinical findings

The time between the patient’s diagnosis and the WB-MRI reveal is typically less than 4 weeks at the proposed recruitment sites. The majority of findings on WB-MRI will have no direct impact on patient care during this time. However should WB-MRI reveal a serious finding which could have an immediate impact on direct patient care before the MDT, the reporting radiologist will contact the patient’s clinician to discuss the finding and a decision will be made as to whether the results should be revealed early to all members of the clinical team (based on review of standard tests already performed which may also have detected the finding). Specific findings which will trigger this review are:

- Impending spinal cord compression

For peer review only - http://bmjopen.bmj.com/site/about/guidelines.xhtml
For peer review only

- Deep vein thrombosis or pulmonary embolism
- Brain metastasis with significant mass effect requiring immediate treatment

It is unlikely the patient would be asymptomatic in any of these scenarios, and it is probable additional imaging tests would have already been requested as part of usual clinical care.

Early release for urgent patient management

Although unlikely, it is possible there may be a need to access the WB-MRI report urgently before its release to the MDT, for example if a recruited patient presents to hospital with collapse and knowledge of the WB-MRI findings would potentially change patient management. As noted above, imaging hubs will keep a copy of the free text report of the WB-MRI and this will be made available if requested by clinical teams in this scenario.

8.2.6. Time to full staging

Standard pathway

The total time required to fully stage the patient using standard imaging pathways will be calculated. The start of the staging process will be defined as the date of request of the first staging investigation following a proven or assumed diagnosis of lung cancer (for example date of requested PET/CT after CT or biopsy diagnosis of likely lung tumour). The completion of staging will be defined as the date when the MDT/ appropriate MDT personnel made the first major treatment decision based on the standard imaging.

WB-MRI

Because revelation of the result of WB-MRI is deferred until after standard staging is complete, actual measurement of the time to full staging using WB-MRI will not be possible. The theoretical time to complete staging using WB-MRI will be thus modelled taking into consideration the time from recruitment date to the date of the WB-MRI, plus the number and type of additional staging investigations generated by WB-MRI.

8.2.7. Patient follow up

Patients will be followed for a period of 12 months from the date of recruitment. Follow up will not require any trial specific patient visits to the recruiting site, and follow up CRFs can be completed using hospital databases or patient notes.

For all recruited patients the final TN tumour stage according to the 7th classification will be recorded based on histopathological analysis of surgical specimens (if surgery is performed). The results of any biopsy procedure undertaken over the 12 months will also be recorded. The date, nature and findings of follow up imaging investigations will be recorded for each recruited patient, in particular the presence or absence of metastasis.

The date and cause of patient death and post mortem findings (if performed) will also be recorded.

For peer review only - http://bmjopen.bmj.com/site/about/guidelines.xhtml

Use will be made of MDT records and hospital data repositories to collate this data, and data collection will be coordinated by designated individuals at each recruitment site, aided by UCL CTC.

8.2.8. Patient diaries and EQ-5D Quality of Life questionnaires

All patients will receive a Diary and EQ-5D Quality of Life questionnaire every 3 months which will be posted by UCL CTC. Upon consent the recruiting site must inform the patient that they will receive an EQ-5D questionnaire and Diary through the post from UCL CTC and they are to complete these and return them using the pre-paid envelope provided. The patient diary will cover a 3 month period and will collect information regarding the patient's Primary Health Care contacts, other health care contacts and also the medication they are currently taking. Patient diaries and EQ-5D questionnaires will be posted to all patients at consent and then at 3 monthly intervals for 12 months.

8.2.9. Final reference standard for tumour stage (Consensus Meetings)

Multi-disciplinary consensus panel review is standard methodology for diagnostic test accuracy studies where an independent reference standard is impossible because of incorporation bias. Consensus panels will convene annually to derive the reference standard for tumour stage at diagnosis for recruited patients completing the first 12 month follow up (i.e. a panel at the end of year 2 will consider patients recruited in year 1, a panel at the end of year 3 will consider patient recruited in year 2 etc.). The panels will consider all available clinical information including the results of all original staging investigations, WB-MRI, histopathology (surgical resection and biopsies), follow up imaging and post-mortem reports (where available) and MDT records. UCL CTC will coordinate collation of these data via submitted CRFs over the preceding 12 months for presentation to the panel. Each imaging hub or recruitment site will host a consensus panel to consider patients undergoing WB-MRI (or recruited) at its site (so all imaging studies are available for review on local PACS systems if required). Each panel will consist of at least an oncologist, and/or chest physician, and 3 radiologists, 1 external to the imaging hub and 2 internal: 1 with specific expertise in WB-MRI and 1 with expertise in PET (a single radiologist is acceptable if they have expertise in WB-MRI and PET). The panel will have access to a histopathologist if required. Each panel will adjudicate on the TMN stage of the cancer at diagnosis, including the organ specific sites and burden of metastatic spread.

The definition of the presence or absence of metastasis will be assigned for each organ on a designated CRF. The designation will be made in consensus by all on the panel. Consideration will be made to histology in all biopsied lesions. In the absence of histological proof of metastasis, metastatic disease will be assumed if new lesions appear during the 12 month follow up with imaging characteristics compatible with metastasis and no alternative explanation, or if lesions with characteristics compatible with metastasis which either grow or shrink (on therapy). Lesions identified which remain stable over the 12 month follow up period will assumed not to be metastatic unless there are specific circumstances considered by the panel that indicate malignancy (e.g. change in lesion morphology with treatment).

For peer review only - http://bmjopen.bmj.com/site/about/guidelines.xhtml

All “new” sites of metastatic disease diagnosed in the 12 month follow up will be assessed by the panel to see if they were visible in retrospect at diagnosis. Although the primary analysis by the original reporting radiologist will be that used to define test accuracy, this retrospective review will allow definition of the rates of perceptual error.

For patients in whom the primary tumour has been completely removed within 3 months of diagnosis, all new metastatic sites will be assumed to have been present at diagnosis for the purposes of calculating test sensitivity.

If the primary tumour is left in situ for more than 3 months (or there is incomplete removal), any new diagnosed sites of metastatic disease will be assumed to have been present at diagnosis if identified within 6 months of diagnosis of the primary tumour. If they are diagnosed beyond 6 months of diagnosis, and there is no evidence of their presence on retrospective review of all staging investigations, they will be assumed to be new disease not present at diagnosis.

If patients with tumours left in situ do not undergo any imaging capable of detecting metastatic disease within 6 months of diagnosis of the primary tumour (other than initial staging tests) and imaging beyond this identifies metastatic disease not visible in retrospect on any trial imaging, the consensus panel will opine if the disease was likely present at diagnosis, based on its location, size and imaging characteristics.

If a patient dies before the 12 month follow up, the panel will review all available imaging, histopathology and clinical course prior to death and in consensus state if a confident diagnosis of the presence or absence of metastatic disease can be made (for example imaging characteristics compatible with metastasis and no alternative explanation, or if lesions with characteristics compatible with metastasis which either grow or shrink (on therapy). If this judgement cannot be made with confidence (for example if the patient has equivocal lesions on staging investigations and no further follow up), these patients will not be excluded but multiple imputation will be used to account for missing data. A sensitivity analysis for the primary outcome, where only patients with complete data are analysed, will exclude these patients.

8.2.10. Health psychology assessment

Study 1: Patient interviews (approximate n=25)

Target accrual has now been reached for the patient experience interview part of the trial and so recruitment to this part is now closed. Individual in-depth interviews were conducted by a health psychologist pertaining to their experience of the cancer staging process. The aim of the interviews is to determine patients’ experience of WB-MRI, standard tests and those tests generated by WB-MRI. We collected simple demographic data from participants in relation to gender, age and educational level as such factors may influence experience and preferences, and this data will enable us to determine whether the sample of people who are interviewed are representative of the sample of people who also participate in studies 2 and 3 (see below). The interviews assessed which aspects of testing caused patients physical or psychological stress (e.g. number of tests/hospital visits, test attributes (i.e. physical experiences of the tests such as claustrophobia, need to lie still, scanner noise etc.), total length of time taken, additional tests generated by initial scan findings) and elicited any factors patients felt would have made staging easier for them.

For peer review only - http://bmjopen.bmj.com/site/about/guidelines.xhtml

The interviews provided in-depth qualitative data about the experience of cancer staging and in particular undergoing WB-MRI, and has informed the modification of a questionnaire (study 2) designed to assess patient acceptability and experience of WB-MRI compared to standard tests, and also has informed the design of a discrete choice experiment (study 3).

Study 2: Patient questionnaires (approximate n=50)

Target accrual has now been reached for the patient experience questionnaire part of the trial and so recruitment to this part is now closed. Follow up questionnaires will continue to be posted until the last patient's month 6 time point occurs. A questionnaire was developed and sent to consenting patients to assess their experience and acceptability of WB-MRI and standard tests (e.g. PET-CT). Final design was informed by analysis of the qualitative data collected in study 1 (See above). Patient Experience Questionnaire content is outlined in Table 3 and includes patients' views about test preparation (e.g. fasting), and experience of the scan itself (e.g. discomfort, claustrophobia, fear etc.). Patients were also asked to rate the importance of various attributes associated with competing staging pathways (e.g. waiting time from diagnosis to treatment, total test number, test accuracy etc.). This rating identified those attributes considered most important by patients, which has been included in the discrete choice experiment (study 3).

Patients were given the opportunity to opt in to completing the questionnaires when they were recruited into the trial. Questionnaires, together with the positive and negative affect schedule (PANAS) and the General Health Questionnaire (GHQ-12) were administered during and after staging pathways, and at 3 and 6 months later to examine patient test preferences once they are aware of their cancer stage and knew which of the tests they received were more accurate as well as the burden placed on them by any extra tests they needed as a result of WB-MRI.

Patients were posted the baseline questionnaire along with pre-paid reply envelope at the time they were recruited into the trial. The questionnaire was worded to take into account the fact that some patients had not yet had any staging investigations. Patients were posted the post-staging and 3 and 6 month follow up questionnaires along with pre-paid envelopes at the relevant time points.

Study 3: Discrete choice experiment (approximate n=50)

Discrete choice experiments (DCE) can elicit preferences for different types of health care provision [18] by estimating the relative importance of different attributes, and the trade-offs between them [19]. WB-MRI and conventional staging pathways differ in associated attributes, not only related to physical experience but also rate of adverse events, time to diagnosis, overall accuracy etc. The most important attributes have been identified in studies 1 and 2 and appropriate levels assigned to each based on accumulating data from the trial, together with appropriate literature review. A DCE questionnaire has been developed whereby patients state their preference between two choices, with each choice containing different levels of the identified attributes.

The DCE questionnaire is optional and patients will be asked to consent to complete the questionnaire on trial entry. Once a patient consents to the DCE they will be allocated a DCE questionnaire (out of a possible 2 DCE questionnaires) which will be posted by UCL CTC immediately after registration (baseline) along with their QoL

For peer review only - http://bmjopen.bmj.com/site/about/guidelines.xhtml

questionnaire, patient diary and pre-paid envelope. UCL CTC will supply the details of any recruited patients who consented to complete the DCE questionnaire to the Health psychologists who may contact them directly.

Table 2: Questionnaire schedule.

Questionnaires will be posted to patients by UCL CTC.

	Baseline (peri-staging)	Months after registration		
		1	3	6
Demographic measures	✓			
Co-morbidities	✓			
Self-rated health	✓			
Symptoms of lung cancer	✓			
Concern for future consequences	✓			
Which staging tests have had	✓	✓		
Perceived importance of test attributes	✓	✓		
Perceived acceptability of different tests	✓	✓		
Least acceptable aspects of different tests	✓	✓		
Agreement to having tests again (short and long-term)	✓	✓		
Single test choice	✓	✓		
Test recovery time	✓	✓		
Patient experience of WB-MRI and standard tests	✓	✓		
Perceived efficacy of WB-MRI and standard tests		✓		
Difficulties with completing scan (WB-MRI and standard tests)		✓		
Ranked attribute preferences	✓	✓		
Feelings about number of tests			✓	✓
EQ-5D	✓		✓	✓
PANAS	✓	✓	✓	✓
GHQ-12	✓	✓	✓	✓

For peer review only - http://bmjopen.bmj.com/site/about/guidelines.xhtml

8.2.11. Cost Effectiveness Assessment

Overview

We will undertake a detailed analysis of the cost and the cost-effectiveness of WB-MRI versus standard NICE-approved staging algorithms. The analysis will conform to accepted economic evaluation methods [15]. All costs will be assessed from the perspective of the NHS and personal social services (PSS).

Making comparisons between WB-MRI versus standard staging algorithms

The care pathway for lung cancer patients can both be divided into two stages, the *treatment decision pathway* and the *subsequent disease pathway*. The former includes the time from diagnosis to treatment decision by the MDT; the latter includes the time period following the treatment decision.

The treatment decision pathway will be different between WB-MRI and standard staging algorithms, yielding different costs and potentially different treatment decisions. In patients for whom the treatment decision with WB-MRI is the same as that with conventional staging algorithms, the subsequent disease pathways will be the same. Where the treatment decision with WB-MRI is different, the disease pathway will be different, yielding potentially different costs and health outcomes.

If in the patients studied the concordance between the treatment decisions associated with WB-MRI and conventional staging algorithms is high, then the economic analysis can focus on the cost of the treatment decision pathways only because the disease pathways will be no different. In this case the cost-effectiveness of WB-MRI versus conventional staging algorithms depends only on the incremental cost (positive or negative) of WB-MRI versus standard staging algorithms in the treatment decision pathway.

Conversely, if the concordance between the treatment decisions is low, then the economic analysis ought to focus on both the treatment decision pathways and the subsequent disease pathways because both of these will vary between WB-MRI and conventional staging algorithms. In this case the cost-effectiveness of WB-MRI depends on the incremental cost of the WB-MRI versus standard staging algorithms in the treatment decision pathway plus the incremental costs and health benefits of the disease pathway.

The precise nature of the economic analysis will therefore depend on the degree of concordance between treatment decisions provoked by WB-MRI versus conventional staging.

Discordance of major treatment decisions

Discordance will be defined when the first major treatment decision differs between WB-MRI and standard investigations in greater than 10% of patients. Concordance will be defined as the absence of discordance. For lung cancer we define a major difference in treatment decision as occurring when only one pathway suggests the patient is a surgical candidate or suitable for radical radiotherapy. A 3x2 table will be constructed according to decision to perform surgery, use radical radiotherapy or not based on WB-MRI vs. standard diagnostic pathways.

For peer review only - http://bmjopen.bmj.com/site/about/guidelines.xhtml

Scenario 1: there is concordance between the treatment decisions associated with WB-MRI and standard staging

In this case, the cost components included in the analysis will be:

- Conventional tests for staging of disease among diagnosed patients (e.g., PET/CT/EBUS/TBNA/USS neck for lung cancer, plus additional tests as indicated by the conventional staging algorithm);
- Costs of treating adverse events associated with staging tests;
- WB-MRI, plus additional tests generated by WB-MRI;
- MDT meetings.

The volume of resource use for each cost component will be measured directly in the trial from treatment decisions recorded in MDT reports, based first on conventional staging alone and then based on WB-MRI alone, and on patient records included in the trial. Unit costs will be taken from standard published sources. Since the two algorithms yield the same treatment decisions cost-effectiveness depends on the incremental cost of WB-MRI versus conventional staging algorithms in the treatment decision pathway (i.e., formally this is a cost-minimisation analysis).

Scenario 2: there is discordance between the treatment decisions associated with WB-MRI and standard staging

In this case, cost-effectiveness depends on the incremental cost (positive or negative) of the treatment decision pathway and disease pathway associated with WB-MRI versus conventional staging and the incremental health benefits (positive or negative). We will calculate cost-effectiveness in terms of the incremental cost per quality-adjusted life year (QALY) gained using one year and lifetime time horizons.

For the analysis based on the one-year time horizon, the trial will provide information on the treatment decisions arising from WB-MRI and standard staging and follow up data for the first year. We will collect these data from three sources.

Firstly for all patients in the trial we will collect the resource use data for the main drivers of hospital costs using a trial specific CRF. This will collect resource use data on the following cost components for each patient:

- Imaging investigations
- Chemotherapy
- Radiotherapy
- Surgery and biopsies
- Outpatient visits
- Inpatient stays
- Day cases

These data will be recorded prospectively based mainly on MDT consensus meeting recommendations and on patient records (for major changes in treatment). Unit costs will be taken from standard published sources and applied to the resource use data, allowing us to cost the care received by each patient.

For peer review only - http://bmjopen.bmj.com/site/about/guidelines.xhtml

In addition, from this source we will collect data on whether or not the patient died during the follow up period, and if they died, the date of death.

Secondly for all patients in the trial we will prospectively collect resource use data using patient diaries. This will supplement the above data which may not provide a complete picture of hospital resource use, plus it will allow us to collect data on primary and community care contacts. These will record resource use data on the following cost components for each patient:

- Medications taken
- Day cases
- GP contacts
- Practice and community nurse contacts
- A&E Visits
- Any other primary care or community care contacts related to cancer

The diaries will be posted out by UCL CTC at baseline, 3, 6, and 9 months and patients will be asked to complete them for the following 3 month period.

In addition we will also collect data on health-related quality of life score, measured according to the EQ-5D (www.euroqol.org) which will be measured at baseline and at 3, 6, 9 and 12 months for all surviving patients.

UCL CTC will contact sites just prior to sending out any post registration diaries or questionnaires to check the status of the patient to ensure none are sent inappropriately to deceased patients.

Patients who are found not to have cancer or are withdrawn for other reasons (see section 13), will not be sent diaries, quality of life or patient experience questionnaires to complete.

Thirdly the two sources of resource use data described above will be supplemented with a review of resource data use collected in the recently completed Lung-BOOST trial. This may be supplemented by retrospective review of resource use (including imaging, outpatient visits, inpatients stays and medication use) for the 12 months post diagnosis of up to 150 patients diagnosed with lung cancer at UCLH. This retrospective review will be carried out by a member of the clinical team and according to guidance provided by the UCLH Caldicott guardian. All collated retrospective data will be fully anonymised before sharing with other trial researchers outside the clinical care team.

8.2.12. Nested substudies

1) Diagnostic performance of limited WB-MRI protocols based on diffusion and T1 weighted imaging only, and to assess the incremental benefit of intravenous gadolinium contrast enhancement.

   As noted above, WB-MRI will be analysed using a locked sequential viewing paradigm such that the diagnostic accuracy of Diffusion and non contrast enhanced T1 images, Diffusion, non contrast enhanced T1 and T2 images, and Diffusion, non contrast enhanced T1, T2 and contrast enhanced T1 images can be calculated.

For peer review only - http://bmjopen.bmj.com/site/about/guidelines.xhtml

2) Effect of radiologists' diagnostic confidence on the accuracy of WB-MRI.

Radiologists will indicate their level of confidence for the presence or absence of metastasis on a 1-6 scale on the CRF recording their interpretation of the WB-MRI.

3) Inter-observer variation in WB-MRI interpretation.

Each hub radiologist (n=6) will interpret a sample of 25 WB-MRI datasets selected at random from the other imaging hubs to define inter-observer variation in the reported presence absence of metastatic disease.

4) The collated WB-MRI datasets and 12 month patient follow up data collated as part of the trial protocol provides opportunities to retrospectively mine the data for additional diagnostic indices and prognostic markers (See appendix 4). The following or similar may be quantified in all or a subset of whole body MRI datasets:

- Body fat (organ, visceral and whole body)
- MRI signal from the primary tumour and metastatic sites, notably diffusion weighted signal, apparent diffusion coefficient, T1 (including fat and water components), T2 signal, Contrast enhancement, and textural analysis (for heterogeneity) of the primary and metastatic deposits on the various sequences.

For peer review only - http://bmjopen.bmj.com/site/about/guidelines.xhtml

9. Data Management Guidelines

Data will be collected from sites on version controlled case report forms (CRFs) designed for the trial and supplied by UCL CTC. Data entered onto CRFs must reflect source data at site. Some data will be recorded directly on the CRFs (i.e. no prior written or electronic record of data), and it will be considered to be source data. This will be made clear in the CRF guidance.

Where supporting documentation (e.g. autopsy reports, pathology reports, CT scan images etc.) is being submitted to UCL CTC, the patient's trial number must be clearly indicated on all material and any patient identifiers removed/blacked out prior to sending to maintain confidentiality.

9.1. Completing Case Report Forms

All CRFs must be completed and signed by staff who are listed on the site staff delegation log and authorised by the PI to perform this duty. The PI is responsible for the accuracy of all data reported in the CRF.

All entries must be clear, legible and written in ball point pen. The use of abbreviations and acronyms must be avoided.

Any corrections made to a CRF at site must be made by drawing a single line through the incorrect item ensuring that the previous entry is not obscured. Each correction must be dated and initialled. Correction fluid must not be used.

The use of abbreviations and acronyms must be avoided.

Once completed the original CRFs must be sent to UCL CTC and a copy kept at site.

9.2. Missing Data

To avoid the need for unnecessary data queries CRFs must be checked at site to ensure there are no blank fields before sending to UCL CTC. When data are unavailable because a measure has not been taken or test not performed, enter "ND" for not done. If an item was not required at the particular time the form relates to, enter "NA" for not applicable. When data are unknown enter the value "NK" (only use if every effort has been made to obtain the data).

9.3. Timelines for data return

CRFs must be completed at site and returned to UCL CTC as soon as possible and within 4 weeks of scheduled time point. For further details, see CRF instructions.

Sites who persistently do not return data within the required timelines may be suspended from recruiting further patients into the trial by UCL CTC and subjected to a 'for cause' monitoring visit. See section 12.2 ('For cause' on-site monitoring) for details.

For peer review only - http://bmjopen.bmj.com/site/about/guidelines.xhtml

9.4. Data Queries

Data arriving at UCL CTC will be checked for legibility, completeness, accuracy and consistency, including checks for missing or unusual values. Query reports will be sent to the data contact at site. Further guidance on how data contacts should respond to Data Queries can be found on the Query Reports.

For peer review only

For peer review only - http://bmjopen.bmj.com/site/about/guidelines.xhtml

10. Safety Reporting

Clinical Review

The MRI Imaging Hub and/or Recruiting site will record any complications attributable to the WB-MRI or any additional test performed as a result of the WB-MRI, notably contrast reactions, biopsy complications (infection, bleeding or hospital admission), and treatment target breaches on the Staging Complications CRF which will be submitted to UCL CTC.

UCL CTC will provide trial data to the CI on a periodic basis for review. If it is declared necessary to revise the conduct of the trial due to safety concerns, UCL CTC will inform the REC as appropriate.

For peer review only

For peer review only - http://bmjopen.bmj.com/site/about/guidelines.xhtml

11. Incident Reporting

Organisations must notify UCL CTC of all deviations from the protocol or GCP immediately. UCL CTC may require a report on the incident(s) and a form will be provided if the organisation does not have an appropriate document (e.g. Trust Incident Form).

If site staff are unsure whether a certain occurrence constitutes a deviation from the protocol or GCP, the UCL CTC trial team can be contacted immediately to discuss.

UCL CTC will use an organisation's history of non-compliance to make decisions on future collaborations.

For peer review only

For peer review only - http://bmjopen.bmj.com/site/about/guidelines.xhtml

12. Trial Monitoring and Oversight

Participating sites and PIs must agree to allow trial-related on site monitoring, Sponsor audits and regulatory inspections by providing direct access to source data/documents as required. Patients are informed of this in the patient information sheet and are asked to consent to their medical notes being reviewed by appropriate individuals on the consent form.

UCL CTC will determine the appropriate level and nature of monitoring required for the trial. Risk will be assessed on an ongoing basis and adjustments made accordingly.

12.1. Central Monitoring

Sites will be requested to submit relevant logs to UCL CTC at the frequency detailed in the trial monitoring plan or on request and these will be checked for consistency and completeness. Also refer to sections 4.2.2 (Required Documentation) and 6.2 (Screening Logs).

Ensuring patient eligibility is the responsibility of the PI or other delegated Investigator(s). Checks of the criteria listed on the registration form will be undertaken by an appropriately trained UCL CTC staff member prior to registration. Also refer to section 7.1 (Registration).

Details relating to the informed consent process will be collected on the registration form and are subject to review by UCL CTC as part of patient eligibility.

Data received at UCL CTC will be subject to review in accordance with section 9.4 (Data Queries).

Where central monitoring of data and/or documentation submitted by sites indicates that a patient may have been placed at risk, the matter will be raised urgently with site staff and escalated as appropriate (refer to section 11 (Incident Reporting) and 12.2 ('For cause' on site monitoring) for further details).

12.2. 'For Cause' On Site Monitoring

On site monitoring visits may be scheduled at a site where there is evidence or suspicion of non-compliance with important aspect(s) of the trial protocol/GCP requirements. Sites will be sent a letter in advance outlining the reason(s) for the visit. The letter will include a list of the documents that are to be reviewed, interviews that will be conducted, planned inspections of the facilities, who will be performing the visit and when the visit is likely to occur.

Following a monitoring visit, the Trial Monitor/Trial Coordinator will provide a report to the site, which will summarise the documents reviewed and a statement of findings, deviations, deficiencies, conclusions, actions taken and actions required. The PI at each site will be responsible for ensuring that monitoring findings are addressed in a timely manner, and by the deadline specified.

For peer review only - http://bmjopen.bmj.com/site/about/guidelines.xhtml

UCL CTC will assess whether it is appropriate for the site to continue participation in the trial. Refer to section 11 (Incident Reporting) for details.

12.3. Oversight Committees

12.3.1. Trial Management Group (TMG)

The TMG will include the Chief Investigator, clinicians and experts from relevant specialities and Streamline L trial staff from UCL CTC (see page 3). The TMG will be responsible for overseeing the trial. The group will meet regularly at least once a year and will send updates to PIs (via newsletters or at Investigator meetings) and to the NCRI Lung Clinical Studies Group.

The TMG will review substantial amendments to the protocol prior to submission to the REC. All PIs will be kept informed of substantial amendments through their nominated responsible individuals.

TMG members must sign a charter confirming acceptance of their responsibilities.

12.3.2. Trial Steering Committee (TSC)

The role of the TSC is to provide overall supervision of the trial. The TSC will review the recommendations of the Independent Data Monitoring Committee and, on consideration of this information, recommend any appropriate amendments/actions for the trial as necessary. The TSC acts on behalf of the funder and Sponsor.

TSC members must sign a charter confirming acceptance of their responsibilities and declaring any potential conflicts of interest.

12.3.3. Independent Data Monitoring Committee (IDMC)

The role of the IDMC is to provide independent advice on data and safety aspects of the trial. Meetings of the Committee will be held at least once a year or as necessary to address any issues. The IDMC is advisory to the TSC and can recommend premature closure of the trial to the TSC.

IDMC members must sign a charter confirming acceptance of their responsibilities and declaring any potential conflicts of interest.

12.3.4. Role of UCL CTC

UCL CTC will be responsible for the day to day coordination and management of the trial and will act as custodian of the data generated in the trial (on behalf of UCL). UCL CTC is responsible for all duties relating to safety reporting which are conducted in accordance with section 10 (Safety Reporting).

For peer review only - http://bmjopen.bmj.com/site/about/guidelines.xhtml

13. Withdrawal of Patients

In consenting to the trial, patients are consenting to the WB-MRI, and also specifically consenting to completing questionnaires and diaries.

Losses to follow up

If a patient moves from the area, the site should make every effort to ensure the patient is followed up at another participating trial site and for this new site to take over the responsibility for the patient, or for follow up via GP. Details of participating trial sites can be obtained from the UCL CTC trial team who must be informed of the transfer of care and follow up arrangements.

Patients should be considered lost to follow up only once documented efforts on the part of the site have failed to produce any response or information from the patient or GP over the course of one year.

If a patient is lost to follow up at a site every effort should be made to contact the patient's GP to obtain information on the patient's status. If the patient cannot be contacted this should be recorded on the Change of Status CRF.

Patients Withdrawing Consent

If a patient expresses their wish to withdraw from the trial, sites should explain the importance of allowing routine follow up data to be used for trial purposes and for allowing existing collected data to be used. If the patient gives a reason for their withdrawal, this should be recorded (see below).

Withdrawal of Consent to Future Data Collection and use of past data

If a patient explicitly states they do not wish to contribute further data to the trial or allow the use of past data, their decision must be respected and recorded on the Change of Status CRF. In this event details should be recorded in the patient's hospital records, no further CRFs must be completed and no further data sent to UCL CTC.

Withdrawal of Consent to QoL, Resource use diary and/or Patient experience questionnaires

If a patient withdraws consent from completing the QoL, Resource use diary and/or the Patient experience questionnaires their decision must be recorded on the Change of Status CRF. UCL CTC will not post any further documents to the patient. However, the patient may continue to participate in the trial, and trial follow up completed as per protocol.

For peer review only - http://bmjopen.bmj.com/site/about/guidelines.xhtml

14. Trial Closure

14.1. End of Trial

For regulatory purposes the end of the trial will be 1 year after recruitment of the final patient at which point the 'declaration of end of trial' form will be submitted to ethical committees, as required.

Following this, UCL CTC will advise sites on the procedure for closing the trial at the site.

14.2. Archiving of Trial Documentation

At the end of the trial, UCL CTC will archive securely all centrally held trial related documentation for a minimum of 5 years. Arrangements for confidential destruction will then be made. It is the responsibility of PIs to ensure data and all essential documents relating to the trial held at site are retained for a minimum of 5 years after the end of the trial, in accordance with national legislation and for the maximum period of time permitted by the site.

Essential documents are those which enable both the conduct of the trial and the quality of the data produced to be evaluated and show whether the site complied with the principles of GCP and all applicable regulatory requirements.

UCL CTC will notify sites when trial documentation held at sites may be archived. All archived documents must continue to be available for inspection by appropriate authorities upon request.

Health Psychology data collected as part of this research will be retained for 10 years in line with Birkbeck, University of London's records management policy. At the end of this time period the documents will be shredded. The recordings of the interviews will be destroyed after they have been transcribed and verified.

14.3. Early discontinuation of trial

The trial may be stopped before completion as an Urgent Safety Measure on the recommendation of the TSC or IDMC (see section 12.3.2 TSC and 12.3.3 IDMC). Sites will be informed in writing by UCL CTC of reasons for early closure and the actions to be taken with regards the treatment and follow up of patients.

14.4. Withdrawal from trial participation by a site

Should a site choose to close to recruitment the PI must inform UCL CTC in writing. Follow up as per protocol must continue for all patients recruited into the trial at that site and other responsibilities continue as per Clinical Trial Site Agreement (CTSA).

For peer review only - http://bmjopen.bmj.com/site/about/guidelines.xhtml

15. Quality Assurance

The technical quality of at least 10% the WB-MRIs from each imaging hub will be assessed by the trial radiographer at UCLH. Using the 3Dnet™ software, the radiographer will access performed scans on a weekly basis and enter an audit score of quality according to the definitions below. A report of the quality will be provided to the CI every 2 weeks and to the TMG at each meeting.

Technical quality – general

1 = More than one sequence with substantial degradation of images severely limiting interpretation of those sequences, and not repeated

2 = One sequence with substantial degradation of images severely limiting interpretation of that sequence, and not repeated

3 = More than one sequence has minor artefact, but all remain fully diagnostic and repeat although optimal, not necessary OR all sequences initially technically inadequate (score 1 or 2) correctly repeated

4 = One sequence a has minor artefact, but remains fully diagnostic and repeat, although optimal, not necessary

5 = All sequences technically optimal with no artefact or degradation

Technical quality – anatomical coverage

1 = Wrong examination performed

2 = More than one sequence does not adequately cover the body (skull to mid thigh), or designated organ(s) coverage

3 = One sequence does not optimally cover the body or designated organ(s) coverage but examination remains fully diagnostic

4 = All sequences optimally cover the body and designated organ(s) coverage

For peer review only

For peer review only - http://bmjopen.bmj.com/site/about/guidelines.xhtml

16. Statistics

16.1. Sample size calculation

SAMPLE SIZE: Studies of diagnostic test accuracy are based on a cohort design where all patients receive all main diagnostic tests (PET/CT or WB-MRI), although additional tests ordered in each staging pathway differ between patients, based on clinical appropriateness. Existing pathways and therapeutic decision making is complex for lung cancers (see flow chart). This NIHR HTA trial focuses on first definitive staging and we are powered at this point in the patient pathway, informed by a detailed literature review.

Multiple therapeutic options are contingent on diagnosis of metastatic disease.

We therefore power on a change in sensitivity for metastases detection with a WB-MRI pathway as a replacement to the standard NICE staging pathway.

The premise of the trial power is to detect a higher sensitivity for extra thoracic disease (specifically brain and bone metastasis) with WB-MRI in comparison to standard imaging paradigms. To power on equivalence or non-inferiority would be well beyond pragmatic recruitment and funding.

Based on recent meta-analysis data [10] considering 1874 patients, the overall prevalence of brain metastasis at presentation in NSCLC is 13% (6% to 32%) and bone 20% (8-34%). Around 20% have metastasis at multiple sites [1, 4, 17, 20].

Prevalence assumptions

Conservative estimate of the prevalence of brain metastasis at presentation is 10%.

20% of these patients are already identified as having metastatic disease via detection of co-existing extra cranial disease on pre-diagnosis CT (e.g. liver, adrenal).

A further 10% of patients will have disease at more than one site on CT (and therefore more likely detected by PET CT than if isolated to the brain).

Overall 12% (n=40) of the hypothetical 325 patients with potentially operable disease will have isolated brain metastasis and 5% (n=16) have bone metastasis. The remaining 8% (of the total 25% with undiagnosed extra-thoracic metastatic disease) will have metastasis at other organ sites (e.g. lung, liver adrenal).

Sample size calculation

Comparison of WB-MRI to PET & PET/CT accuracy - both against reference standard

- Paired trial design – comparing two diagnostic pathways where both are used in all patients.
- Sample size method for difference in paired proportions [21].
- Power trial to show difference in sensitivity, as fewer patients are with metastasis than without. Trials powered like this should also be suitable for difference in specificity.
- 80% power type II error, type I error 5% (p<0.05).

For peer review only - http://bmjopen.bmj.com/site/about/guidelines.xhtml

Streamline L

- Assume ratio of marginal cells of 2 x 2 table comparing WB-MRI to PET: s/t = 9 to 11 (tables 3 and 4)
- Assume sum of marginal cells as proportion of total with disease: s+t/N=0.24
- A sample size of 50 patients with occult metastasis is needed, from a population of 25% prevalence of metastasis. Thus a total sample size of 200 (50 times 4) is required.

200 patients with no metastasis at lung cancer diagnosis and potential surgical candidates would be required to detect a difference of 24% in sensitivity of WB-MRI for metastatic disease (79%) compared to conventional staging (55%) given site specific disease prevalence as described above.

The sample size above must be adjusted to account for "withdrawals". Patients are classified as withdrawn if they are recruited but are not evaluable for any of the following reasons (i) they do not have an WB-MRI scan, the main component of the primary outcome (ii) they do not have cancer (iii) they are lost to follow up before 1 year and do not have sufficient information for the expert panel to evaluate whether metastases were present.

The withdrawal rate was expected to be 20%, giving a target recruitment of 250 patients. However, the actual withdrawal rate has been 43% and the target recruitment has therefore been revised to 353 patients.

Table 3: Calculations comparing WB-MRI to PET

		PET & PET/CT		
	Result	+	-	**Total**
**WB-MRI**	+	26	11	37
	-	1	12	13
	**Total**	27	23	**50**

Table 4: Calculations broken down by tumour site

		PET & PET/CT		
	Result	+	-	**Total**
**WB-MRI**	+	brain-7<br>bone-7<br>other 12	brain-10<br>bone-1<br>other 0	37
	-	brain-0<br>bone-0<br>other-1	brain-8<br>bone-1<br>other-3	13
	**Total**	27	23	**50**

16.2. Population for analysis

The analysis population will be all patients, equivalent to the RCT population intention to treat (ITT) population, with multiple imputation used to impute missing data. For the primary outcome, complete case analysis (all available patient data with no imputation) will be reported alongside as a sensitivity analysis to the ITT analysis.

For peer review only - http://bmjopen.bmj.com/site/about/guidelines.xhtml

16.3. Analysis of the primary endpoint

Primary Outcome- Per patient sensitivity for metastasis detection by whole body MRI (WB-MRI) compared to standard staging pathways in newly diagnosed non-small cell lung cancer against an expert derived consensus reference standard.

The primary objective is to evaluate whether early whole body magnetic resonance Imaging (WB-MRI) increases per patient sensitivity for metastasis in non-small cell lung cancer compared to standard NICE-approved diagnostic pathways.

- Comparison to detect metastases between early WB-MRI alone and standard NICE diagnostic strategy.
- Difference in per patient sensitivity.
- Paired comparison of proportions of each strategy against the reference standard of full clinical diagnosis and 12 month follow up. [22]
- Imputation will be used to account for missing data and imperfect reference data. [23]

Definition of positive test result from standard tests (conventional imaging)

- Conventional imaging tests include CT and may include PET, USS, organ specific MRI, bone scans, lymph node sampling.
- Report from the MDT will express presence of metastatic disease for each patient categorised as yes, no and equivocal.
- Equivocal results will be grouped with positive test results as these results require additional follow up investigations compared to negative results.
- The radiology imaging report supplied to the MDT will be used for sensitivity analysis using free text report (possible and probable metastasis counted as positive).

Definition of positive test result from WB-MRI and additional tests requested after WB-MRI (WB-MRI as replacement test)

- WB-MRI imaging CRF report from the WB-MRI radiologist will express presence of metastatic disease for each patient categorised as yes, no and equivocal.
- Equivocal results will be grouped with positive test results as these results require additional follow up investigations compared to negative results.

Definition of positive test result from reference test: 12 month expert consensus panel

Expert panel results will be classified as positive or negative for metastasis by expert consensus. No equivocal category will be allowed.

Multiple imputation will be used for missing data using chained equations in STATA [24].

Sensitivity analysis: A sensitivity analysis will investigate the impact of the equivocal results on the detection rate by WB-MRI. Equivocal results will be grouped with negative test results [25].

For peer review only - http://bmjopen.bmj.com/site/about/guidelines.xhtml

16.4. Analysis of secondary endpoints

16.4.1. Efficacy (secondary)

Secondary outcome- The time and test number taken to reach, and the nature of, the first major treatment decision based on WB-MRI in comparison to standard staging pathways.

The objective is to determine how WB-MRI influences time to and nature of first major treatment decision following definitive staging compared to standard investigations and to determine whether early WB-MRI could reduce or replace standard investigations.

For this secondary outcome, the following components will be measured up to the time of the first major treatment decision

- Nature of first major treatment decision and number of patients with changed management decision
- Time taken for diagnostic pathways
- Number of tests: average per patient, number of patients with fewer tests in pathway

These will be compared for WB-MRI pathway in comparison to standard staging pathways.

This information will be reported and discussed as a basis for whether early WB-MRI could replace or reduce standard investigations.

No absolute pre-specified definition of the combination of these outcomes likely to be considered clinically significant is attempted although it is noted that:

- An average decrease in diagnosis time of 7 days is likely to be considered clinically significant if accuracy were similar.

Secondary outcome- Diagnosis accuracy of WB-MRI and conventional staging pathways for local tumour staging and detection of metastasis in comparison to an expert derived consensus reference standard.

The objective is to assess the accuracy of WB-MRI and standard diagnostic pathways for local and distant staging by comparison with an expert derived consensus reference standard using 1 year patient follow up data.

- All comparisons except difference in sensitivity per patient compare WB-MRI as both replacement and additional test compared to standard NICE staging pathway

The following outcomes will be assessed for this secondary outcome:

- Difference in sensitivity per patient: WB-MRI as additional test compared to standard NICE staging pathway
- Difference in sensitivity and specificity per organ site. Count each site once per patient, regardless of the number of metastases per organ.
- Difference in sensitivity and specificity per metastasis.

For peer review only - http://bmjopen.bmj.com/site/about/guidelines.xhtml

Secondary outcome- Inter-observer variability in WB-MRI analysis and effect of diagnostic confidence on staging accuracy.

The objective is to determine the effect of radiologist confidence on the diagnostic accuracy of WB-MRI for metastatic disease.

A substudy will be conducted to look at inter-observer variability between radiologists interpreting WB-MRI images and the effect of diagnostic confidence on staging accuracy.

- Each hub radiologist (n=6) will interpret a sample of 25 WB-MRI datasets selected at random from the other imaging hubs to define inter-observer variation in the reported presence absence of metastatic disease.
- These images will be read after patient management decisions are taken and so will not affect patient diagnosis or treatment.

Secondary outcome – Diagnostic accuracy of limited T1 and diffusion weighted sequences compared to full multi-sequence WB-MRI protocols.

To evaluate the diagnostic performance of limited WB-MRI protocols based on diffusion and T1 weighted imaging only, and to assess the incremental benefit of intravenous gadolinium contrast enhancement.

Comparison of diagnostic accuracy of WB-MRI alone (i) block 1 sequence (ii) block 2 (iii) block 3 sequences.

- Reference standard: WB-MRI plus standard tests with 12 month follow up.
- Methods and tables: as per primary outcome and secondary outcome #3.
- Analysed for units of analysis as (i) per patient (ii) per organ site (iii) per metastasis.

16.4.2. Safety

We will provide information on the following:

- Additional tests ordered as a result of WB-MRI but not ordered for the patient in the standard NICE staging pathway. Note this might include tests due to abnormalities outside lung.
- Extra days in hospital due to extra tests ordered through WB-MRI.

16.4.3. Economic evaluation

Patient-specific utility profiles will then be constructed assuming a straight line relation between each of the patients EQ-5D scores at each follow up point. The quality-adjusted life-years (QALYs) experienced by each patient up to one year will be calculated as the area underneath this profile.

We will calculate for each individual patient in the trial their costs and outcomes up to one year. We will also have data from the Lung-BOOST trial and retrospective data collection for patients previously diagnosed with lung cancer at UCLH on the cost of similar patients up to one year.

For peer review only - http://bmjopen.bmj.com/site/about/guidelines.xhtml

Individual patients will then be grouped according to the specific disease path depending on the treatment decision (e.g., surgery, radical radiotherapy or chemotherapy/non radical radiotherapy for lung cancer) and the accuracy of the staging result. We will calculate the mean costs and QALYs for each group. Mean costs and QALYs for WB-MRI versus standard staging algorithms will then be calculated based on the proportion in each group using each algorithm, which will be different since in this scenario there is discordance in treatment decision.

Cost-effectiveness will be calculated as the mean cost difference between WB-MRI versus standard staging algorithms divided by the mean difference in outcomes (QALYs) to give the incremental cost-effectiveness ratio (ICER). Non-parametric methods for calculating confidence intervals around the ICER based on bootstrapped estimates of the mean cost and QALY differences will be used [26]. The bootstrap replications will also be used to construct a cost-effectiveness acceptability curve, which will show the probability that WB-MRI is cost-effective at one year for different values of the NHS' willingness to pay for an additional QALY. We will also subject the results to extensive deterministic (one-, two- and multi-way) sensitivity analysis.

For the analysis based on the lifetime time horizon we will use the 1 year data described above. To extrapolate beyond the end of the 1 year follow up period we will develop a de novo cost-effectiveness model for the disease pathway, which will be populated via available evidence. These will model patient movements between long-term health states. The models will be similar in design to a recent NIHR HTA-funded trial of PET and MRI for detection of metastasis in breast cancer [27]. Following decisions about model structure, a list of parameter estimates required for the model will be developed. The specific details of the data to be used to populate the model will be determined following the development of the structure and the systematic searches of the literature to identify existing models.

The upshot of this analysis is that we will calculate for each individual patient in the trial their lifetime costs and QALYs. Individual patients will then be grouped according to the specific disease path and the accuracy of the staging result, as before, and we will calculate the mean costs and QALYs for each group. Mean costs and QALYs for WB-MRI versus standard staging algorithms will then be calculated based on the proportion in each group using each algorithm, again as before. We will undertake deterministic (one-, two- and multi-way) and probabilistic sensitivity analysis, the latter assuming appropriate distributions and parameter values [28].

16.4.4. Health psychology assessment

Study 1: Patient interviews (approximate n=25)

No formal statistical analysis will be carried out on the information from the patient interviews. The interviews will provide in-depth qualitative data about the experience of cancer staging and in particular undergoing WB-MRI, and will inform the modification of a questionnaire (study 2) designed to assess patient acceptability and experience of WB-MRI compared to standard tests, and also inform the design of a discrete choice experiment (study 3).

For peer review only - http://bmjopen.bmj.com/site/about/guidelines.xhtml

Study 2: Patient questionnaires (approximate n=50)

Comparative patient experience between WB-MRI and standard staging investigations, identification of important staging pathway attributes, comparative anxiety, expectations and attribute importance before and following the staging process will be analysed using within subjects ANOVA or the Wilcoxon matched-pair sign-test depending on whether the data are normally distributed or not.

Study 3: Discrete choice experiment (approximate n=50)

Sample size should be greater than (500*c)/(t*a) where t = the number of sets of choices, a = the number of scenarios to choose between in each choice, and c = the largest number of levels for any one attribute [29]. Assuming each person will undertake 15 sets of choices, there are two scenarios in each choice, and the largest number of levels for any one attribute is 3 then the required sample size is (500*3)/(15*2) = 50. We will administer this questionnaire to approximately 50 lung patients after staging has been completed, sampling across more than one of the imaging hubs. The questionnaire will be posted to patients with a pre-paid reply envelope. A reminder letter will be sent if no questionnaire has been returned within approximately 4 weeks. Analysis will conform to the checklist for conjoint analysis applications in health developed by the International Society for Pharmaceuticals and Outcome Research (ISPOR). We will use a random effects probit model for statistical analysis, which is appropriate given the data structure. Outcomes: The average relative importance weighting of attributes ascribed to standard versus WB-MRI staging pathways by patients diagnosed with lung cancer.

16.4.5. Handling missing data

Multiple imputation will be used for missing data using chained equations in STATA [24].

For the primary outcome, complete case analysis (all available patient data with no imputation) will be reported alongside as a sensitivity analysis to the ITT analysis.

16.5. Interim analyses

Interim safety data will be supplied to the IDMC on a periodic basis from UCL CTC on:

- Accumulating information relating to recruitment and data quality (e.g. data return rates).
- Safety data:
    - Additional tests performed as a result of the WB-MRI that would not have otherwise been performed as part of the patient's standard diagnostic pathway. Note this might include tests due to abnormalities outside colon or lung.
    - Potential harms to patients (e.g. staging complications resulting from MRI).
    - Whole Body MRIs that were not revealed or revealed too early/late.
- Percentage missing data for primary outcome.
- Prevalence of metastasis within recruited patients (key sample size assumption).

For peer review only - http://bmjopen.bmj.com/site/about/guidelines.xhtml

Streamline L

There will be no interim analysis based on the trial outcomes of diagnostic accuracy as all diagnostic test results are made available for the MDT meeting which decides first management treatment decision.

For peer review only

For peer review only - http://bmjopen.bmj.com/site/about/guidelines.xhtml

17. Ethical Approvals

In conducting the trial, the Sponsor, UCL CTC and sites shall comply with all laws and statutes, as amended from time to time, applicable to the performance of clinical trials including, but not limited to:

- the principles of ICH Harmonised Tripartite Guideline for Good Clinical Practice
- the Human Rights Act 1998
- the Data Protection Act 1998
- the Freedom of Information Act 2000
- the Research Governance Framework for Health and Social Care, issued by the UK Department of Health (Second Edition 2005) or the Scottish Health Department Research Governance Framework for Health and Community Care (Second Edition 2006)

17.1. Ethical Approval

The trial will be conducted in accordance with the World Medical Association Declaration of Helsinki entitled 'Ethical Principles for Medical Research Involving Human Subjects' (1996 version) and in accordance with the terms and conditions of the ethical approval given to the trial.

The trial has received a favourable opinion from the London – Camden & Islington Research Ethics Committee.

UCL CTC will submit Annual Progress Reports to the REC, which will commence one year from the date of ethical approval for the trial.

17.2. Site Approvals

The Lead Comprehensive Local Research Network (CLRN), Central and East London CLRN, has given NHS permission following global governance checks. Local governance checks will be undertaken by local CLRNs associated with individual trial sites.

Evidence of approval from the Trust R&D for a trial site must be provided to UCL CTC. Sites will only be activated when all necessary local approvals for the trial have been obtained.

17.3. Protocol Amendments

UCL CTC will be responsible for gaining ethical approval, for amendments made to the protocol and other trial-related documents. Once approved, UCL CTC will ensure that all amended documents are distributed to sites and CLRNs as appropriate.

Site staff will be responsible for acknowledging receipt of documents and for implementing all amendments.

For peer review only - http://bmjopen.bmj.com/site/about/guidelines.xhtml

17.4. Patient Confidentiality & Data Protection

Patient identifiable data, including full name, address, date of birth, gender and NHS number will be required for the registration process and will be provided to UCL CTC. UCL CTC will preserve patient confidentiality and will not disclose or reproduce any information by which patients could be identified. Data will be stored in a secure manner and UCL CTC trials are registered in accordance with the Data Protection Act 1998 with the Data Protection Officer at UCL. Patient identifiable data will be passed on to Birkbeck, University of London. This is described in the patient information sheet and the patients will consent to it.

For peer review only

For peer review only - http://bmjopen.bmj.com/site/about/guidelines.xhtml

18. Sponsorship and Indemnity

18.1. Sponsor Details

Sponsor Name: University College London

Address: Joint Research Office
Gower Street
London
WC1E 6BT

Contact: Director of Research Support

Tel 020 3447 9995/2178 (unit admin)

Fax 020 3447 9937

18.2. Indemnity

University College London holds insurance against claims from participants for injury caused by their participation in the clinical trial. Participants may be able to claim compensation if they can prove that UCL has been negligent. However, if this clinical trial is being carried out in a hospital, the hospital continues to have a duty of care to the participant of the clinical trial. University College London does not accept liability for any breach in the hospital's duty of care, or any negligence on the part of hospital employees. This applies whether the hospital is an NHS Trust or otherwise.

Hospitals selected to participate in this clinical trial shall provide clinical negligence insurance cover for harm caused by their employees and a copy of the relevant insurance policy or summary shall be provided to University College London, upon request.

For peer review only - http://bmjopen.bmj.com/site/about/guidelines.xhtml

19. Funding

This trial is funded by the National Institute for Health Research Health Technology Assessment (NIHR HTA).

For peer review only

For peer review only - http://bmjopen.bmj.com/site/about/guidelines.xhtml

20. Publication Policy

The TMG will oversee the publication and presentation of the data to peer reviewed journals and scientific meetings. All members of the TMG will approve publications. The writing committee will be led by Professor Stuart Taylor and include all TMG members. All TMG members, Trial Coordinator and Trial Statistician will be authors on the publications and named individually.

For peer review only

For peer review only - http://bmjopen.bmj.com/site/about/guidelines.xhtml

21. References

1. *Cancer Stats from Cancer Research UK Website*. Available from: http://info.cancerresearchuk.org.cancerstats/.
2. *NICE Lung Cancer Guidance 2011.*
3. Gilligan, D., et al., *Preoperative chemotherapy in patients with resectable non-small cell lung cancer: results of the MRC LU22/NVALT 2/EORTC 08012 multicentre randomised trial and update of systematic review.* Lancet, 2007. **369**(9577): p. 1929-37.
4. Floriani, I., et al., *Performance of imaging modalities in diagnosis of liver metastases from colorectal cancer: a systematic review and meta-analysis.* J Magn Reson Imaging, 2010. **31**(1): p. 19-31.
5. Qu, X., et al., *A meta-analysis of (1)(8)FDG-PET-CT, (1)(8)FDG-PET, MRI and bone scintigraphy for diagnosis of bone metastases in patients with lung cancer.* Eur J Radiol, 2012. **81**(5): p. 1007-15.
6. Ohno, Y., et al., *Non-small cell lung cancer: whole-body MR examination for M-stage assessment--utility for whole-body diffusion-weighted imaging compared with integrated FDG PET/CT.* Radiology, 2008. **248**(2): p. 643-54.
7. Pierce, D.A. and D.L. Preston, *Radiation-related cancer risks at low doses among atomic bomb survivors.* Radiat Res, 2000. **154**(2): p. 178-86.
8. Schmidt, G.P., M.F. Reiser, and A. Baur-Melnyk, *Whole-body MRI for the staging and follow-up of patients with metastasis.* Eur J Radiol, 2009. **70**(3): p. 393-400.
9. Squillaci, E., et al., *Staging of colon cancer: whole-body MRI vs. whole-body PET-CT--initial clinical experience.* Abdom Imaging, 2008. **33**(6): p. 676-88.
10. Yi, C.A., et al., *Non-small cell lung cancer staging: efficacy comparison of integrated PET/CT versus 3.0-T whole-body MR imaging.* Radiology, 2008. **248**(2): p. 632-42.
11. Risberg, T., et al., *Diagnostic delay causes more psychological distress in female than in male cancer patients.* Anticancer Res, 1996. **16**(2): p. 995-9.
12. Bastiaannet, E., et al., *Perception of burden experienced during diagnostic tests by melanoma patients with lymph node metastases.* Melanoma Res, 2009. **19**(1): p. 36-41.
13. von Wagner, C., et al., *Choosing between CT colonography and colonoscopy in the diagnostic context: a qualitative study of influences on patient preferences.* Health Expect, 2009. **12**(1): p. 18-26.
14. Harcourt, D., N. Rumsey, and N. Ambler, *Same-day diagnosis of symptomatic breast problems: Psychological impact and coping strategies.* Psychology, Health & Medicine 1999. **4**(1): p. 5.
15. *NICE Guide to the methods of technology appraisal 2008*. Available from: http://www.nice.org.uk/media/B52/A7/TAMethodsGuideUpdatedJune2008.pdf.
16. Bossuyt, P.M., et al., *Towards complete and accurate reporting of studies of diagnostic accuracy: The STARD Initiative.* Radiology, 2003. **226**(1): p. 24-8.
17. Bossuyt, P.M., et al., *Comparative accuracy: assessing new tests against existing diagnostic pathways.* BMJ, 2006. **332**(7549): p. 1089-92.
18. Ryan, M., et al., *Use of discrete choice experiments to elicit preferences.* Qual Health Care, 2001. **10 Suppl 1**: p. i55-60.

For peer review only - http://bmjopen.bmj.com/site/about/guidelines.xhtml

19. Ryan, M. and S. Farrar, *Using conjoint analysis to elicit preferences for health care.* BMJ, 2000. **320**(7248): p. 1530-3.
20. Stephens, R.J. and D.H. Johnson, *Treatment and outcomes for elderly patients with small cell lung cancer.* Drugs Aging, 2000. **17**(3): p. 229-47.
21. Julious, S.A., M.J. Campbell, and D.G. Altman, *Estimating sample sizes for continuous, binary, and ordinal outcomes in paired comparisons: practical hints.* J Biopharm Stat, 1999. **9**(2): p. 241-51.
22. Newcombe, R.G., *Improved confidence intervals for the difference between binomial proportions based on paired data.* Stat Med, 1998. **17**(22): p. 2635-50.
23. Reitsma, J.B., et al., *A review of solutions for diagnostic accuracy studies with an imperfect or missing reference standard.* J Clin Epidemiol, 2009. **62**(8): p. 797-806.
24. White, I.R., P. Royston, and A.M. Wood, *Multiple imputation using chained equations: Issues and guidance for practice.* Stat Med, 2011. **30**(4): p. 377-99.
25. Begg, C.B., R.A. Greenes, and B. Iglewicz, *The influence of uninterpretability on the assessment of diagnostic tests.* J Chronic Dis, 1986. **39**(8): p. 575-84.
26. Briggs, A.H., D.E. Wonderling, and C.Z. Mooney, *Pulling cost-effectiveness analysis up by its bootstraps: a non-parametric approach to confidence interval estimation.* Health Econ, 1997. **6**(4): p. 327-40.
27. Cooper, K.L., et al., *Positron emission tomography (PET) and magnetic resonance imaging (MRI) for the assessment of axillary lymph node metastases in early breast cancer: systematic review and economic evaluation.* Health Technol Assess, 2011. **15**(4): p. iii-iv, 1-134.
28. Briggs, A.H., K. Claxton, and M.J. Sculpher, *Decision modelling for health economic evaluation*. Oxford handbooks in health economic evaluation. 2006, Oxford: Oxford University Press. x, 237 p.
29. Johnson, R., *Conjoint Analysis Software*, I. Sawtooth Software, Editor.: Sequim, WA
30. Azvolinsky, A., *Cancer prognosis: role of BMI and fat tissue.* J Natl Cancer Inst, 2014. **106**(6): p. dju177.
31. Yip, C., et al., *Assessment of sarcopenia and changes in body composition after neoadjuvant chemotherapy and associations with clinical outcomes in oesophageal cancer.* Eur Radiol, 2014. **24**(5): p. 998-1005.
32. Attaran, S., et al., *A propensity-matched comparison of survival after lung resection in patients with a high versus low body mass index.* Eur J Cardiothorac Surg, 2012. **42**(4): p. 653-8.
33. Curvo-Semedo, L., et al., *Diffusion-weighted MRI in rectal cancer: apparent diffusion coefficient as a potential noninvasive marker of tumor aggressiveness.* J Magn Reson Imaging, 2012. **35**(6): p. 1365-71.
34. De Felice, C., et al., *Apparent diffusion coefficient on 3.0 Tesla magnetic resonance imaging and prognostic factors in breast cancer.* Eur J Gynaecol Oncol, 2014. **35**(4): p. 408-14.
35. Ohno, Y., et al., *Diffusion-weighted MRI versus 18F-FDG PET/CT: performance as predictors of tumor treatment response and patient survival in patients with non-small cell lung cancer receiving chemoradiotherapy.* AJR Am J Roentgenol, 2012. **198**(1): p. 75-82.

For peer review only - http://bmjopen.bmj.com/site/about/guidelines.xhtml

36. Zakaria, R., et al., *Diffusion-weighted MRI characteristics of the cerebral metastasis to brain boundary predicts patient outcomes.* BMC Med Imaging, 2014. **14**: p. 26.
37. Parikh, J., et al., *Changes in primary breast cancer heterogeneity may augment midtreatment MR imaging assessment of response to neoadjuvant chemotherapy.* Radiology, 2014. **272**(1): p. 100-12.

For peer review only

For peer review only - http://bmjopen.bmj.com/site/about/guidelines.xhtml

Appendix 1: Abbreviations

**CDR**	Clinical Data Repository
**CI**	Chief Investigator
**CLRN**	Comprehensive Local Research Network
**CRF**	Case Report Form
**CSP**	Coordinated System for gaining NHS Permission
**CT**	Computerised Tomography
**CTSA**	Clinical Trial Site Agreement
**DCE**	Direct Choice Experiment
**DPA**	Data Protection Act
**GCP**	Good Clinical Practice
**GHQ**	General Health Questionnaire
**ICER**	Incremental Cost-Effectiveness Ratio
**ICH GCP**	International Conference of Harmonisation-Good Clinical Practice
**IDMC**	Independent Data Monitoring Committee
**IRAS**	Integrated Research Application System
**ISF**	Investigator Site File
**ISRCTN**	International Standard Randomised Controlled Trial Number
**ITT**	Intention To Treat
**MDT**	Multi-Disciplinary Team
**MRI**	Magnetic Resonance Imaging
**NCRI**	National Cancer Research Institute
**NCRN**	National Cancer Research Network
**NHS**	National Health Service
**NICE**	National Institute for Clinical Excellence
**NIHR**	National Institute for Health Research
**NIHR HTA**	National Institute for Health Research Health Technology Assessment
**NRES**	National Research Ethics Service
**OPD**	Out Patients Department
**PACS**	Picture Archiving and Communications System
**PANAS**	Positive and Negative Affect
**PET**	Positron Emission Tomography
**PET-CT**	Positron Emission Tomography- Computerised Tomography
**PI**	Principal Investigator
**PSS**	Personal Social Services
**QA**	Quality Assurance
**QALY**	Quality Adjusted Life Year
**R&D**	Research & Development
**RCT**	Randomised Controlled Trial
**REC**	Research Ethics Committee
**RIS**	Radiology Information System
**SSI**	Site Specific Information
**STARD**	STAndards for the Reporting of Diagnostic accuracy studies
**T**	Tesla

For peer review only

For peer review only - http://bmjopen.bmj.com/site/about/guidelines.xhtml

Streamline L

**TMF**	Trial Master File
**TMG**	Trial Management Group
**TSC**	Trial Steering Committee
**UCL**	University College London
**UCL CTC**	Cancer Research UK & UCL Cancer Trials Centre
**UCLH**	University College London Hospital
**USS**	Ultrasound Scan
**VATS**	Video-Assisted Thoracoscopic Surgery
**WB-MRI**	Whole Body Magnetic Resonance Imaging
**WCBP**	Woman of Childbearing Potential

For peer review only

For peer review only - http://bmjopen.bmj.com/site/about/guidelines.xhtml

Appendix 2: Consent Process

Patients will be recruited at several hospital sites, and for many, the actual WB-MRI will take place at a different hospital (one of the central imaging hubs). It is vital that recruited patients undergo informed consent, have a minimum of 24 hours to consider participation and are subsequently free to withdraw at any time.
The consent process must be sensitive to the needs of patients which may differ from individual to individual. It is important therefore to provide patients with options as to how they may provide their consent.

The proposed consent process for this trial is based on the experience of other similar trials. It was found that patients and recruitment sites found it more efficient if the designated recruiting individual at the peripheral hospital knows that after fully explaining the trial, the patient in principle is willing to take part. For all recruitment sites, patients are seen by a clinical practitioner after a suspected diagnosis of lung cancer based on an abnormal chest X-Ray and CT scan. At this discussion the diagnosis is explained and the need for a series of staging tests (such as PET CT, bronchoscopy, biopsy etc.) is also explained. Because of the need to stage the patient quickly so treatment may start, these tests are often done within a very short time period. There is no scheduled return by the patient to an OPD during this staging process. Instead patients are next seen face to face after the MDT has made a decision about their treatment.

If consent in principle is given, the recruiter can then ring the central hub and there and then arrange the time of the MRI and thus whenever possible inform the patient immediately of the time and date so they can plan their attendance for the scan. Patients expressing an interest in participating in the trial will therefore be asked if they prefer to sign the consent form when first seen when the trial is explained (and this will be later filed in the medical notes and the conversation documented). It will be made perfectly clear that they are free to withdraw at any time and that the scan will be at least 24 hours later (usually more than this). This will save them having to re-attend the hospital simply to sign a consent form, incurring time and expense at a very busy and difficult time for them. Before they go home, where possible patients will be given the date of the WB-MRI and a copy of their signed consent form. The recruitment site can then register the patient with UCL CTC. The date of the WB-MRI will be at least 24 hours later and in reality likely several days later. The patient will take the full patient information sheet home to review at their leisure. They will be provided with a telephone number at the recruitment site to ring if on reflection, they want to opt out of the trial. If they opt out, this will be recorded in the medical notes and screening log. Patients will also be asked permission to be called by the recruitment site or imaging hub to remind them of the date of scan and confirm their attendance. Patients will retain the right to opt out at any time by cancelling or not attending their WB-MRI appointment.

As noted, a major advantage of this approach is that the patient does not have to make a special trip back to the hospital just to sign the consent form. The signed consent

For peer review only - http://bmjopen.bmj.com/site/about/guidelines.xhtml

form does not leave the recruitment hospital and can be more efficiently filed in the patient notes and a copy filed in the Investigator site file: an important aspect of trial governance and patient clinical care.

Note this method of consent has been previously deemed ethically sound and efficient in the NIHR HTA funded Siggar 1 trial (CT colonography, colonoscopy or barium enema for diagnosis or colorectal cancer in older symptomatic patients), Northern and Yorkshire MREC, MREC/3/3/075, which ran from 2004 to 2009 and recruited over 5000 patients to undergo CT colonography in a similar trial design to the current proposal. The trial team did pilot a scheme where patients returned a copy of their signed consent form after 24 hours if they still wanted to take part, but patients found this very cumbersome and confusing and much preferred the process proposed, using telephone confirmation. The procedure is supported by the patient representatives in the trial and has also worked successfully in the BOOST trial which randomised patients to standard care or endobronchial biopsy at exactly the same point in the patient pathway. The BOOST trial ran at many of the intended recruitment sites for Streamline L.

Patients will be given the option to come back and sign the consent form if they or their recruiter feel this is preferable.

Occasionally it may be more convenient for the patient to obtain written consent when the patient attends for the WB-MRI or before a planned conventional staging test. For example the patient may wish to take part in the trial, not wish to sign the consent form on the same day but also would prefer not to attend a hospital appointment just to sign the consent form. This is acceptable although it is the least desirable option. After the patient has signed consent they will need to be registered before proceeding with the WB-MRI and a copy of the consent form will need to be returned to the initial recruitment site.

For peer review only - http://bmjopen.bmj.com/site/about/guidelines.xhtml

Appendix 3: Minimum WB-MRI protocol dataset

MRI Protocol
Whole body is head to thigh

The aim is to complete the whole protocol in 60 minutes or less. Depending on the MRI technology available, it may be possible to use the 5mm slices for all axial imaging, although up to 7mm is acceptable if time constraints are problematic.

1. These specifications are a set of minimum requirements – where higher field strengths/scanner software permits the resolution/quality of sequences should be optimised (whilst keeping the maximum imaging time limited to 1 hour).

2. Scanning maybe performed at either 1.5T or 3T.

3. Whole-body coverage is defined as head to mid-thigh optimised for detection of metastases.

Imaging Procedure

a) A standard safety questionnaire should be completed.
b) For patients undergoing contrast enhancement: set up IV line in a vein in the antecubital fossa, connected to an automated injector with two syringes (contrast and saline flush).
c) Unless contra-indicated administer 20mg buscopan or 1mg glucagon iv. to be given just before the start of the scan.

1. Whole-body diffusion weighted imaging:
Axial: STIR-EPI (or other fat sat technique) diffusion weighted imaging. Fixed slice thickness of 5mm to 7mm (to match T2 and t1 weighted axials as below) two b-values (b50 and b900). A minimum acquisition matrix of 128 x 128 (or an interpolated equivalent) (rectangular FOV should be used if available and appropriate for the patient), as a reference a minimum SNR of 6 on b50 images (for liver) should be maintained if possible by increasing the number of averages. All imaging should be performed in gentle respiration (recommended as 4 stations of 50 slices beginning from the vertex to mid thighs). Diffusion imaging through the brain is optional.

2. Whole-body T2 weighted imaging:
Axial*:* Axial T2 weighted (without fat-suppression) imaging should be performed from vertex to mid-thigh. A 5 to 7 mm slice thickness should be used for all scanners. *Where possible (within the 60 minute imaging time)* respiratory and ECG triggering should be used for the chest, respiratory triggering alone for the upper abdomen. The head, neck, pelvis and legs should be scanned without any triggering. The number of stacks should be adjusted to cover the imaging volume.

For peer review only - http://bmjopen.bmj.com/site/about/guidelines.xhtml

4. **Pre-contrast T1 weighted imaging:**

DIXON Technique to be applied if available.

a. Axial: Whole-body T1 GRE (e.g. Flash 2D) non-contrast enhanced non fat sat. Image resolution and slice thickness should be ideally matched to T2 weighted imaging.

OR

b. Coronal: T1 fat saturated volume interpolated gradient echo imaging (e.g. 3D) pre contrast.

5. **Post-contrast T1 weighted imaging (if gadolinium not contraindicated or refused):**

Minimum data set
Axial liver (60-70 sec)
Axial lung (equilibrium phase)
SFOV axial head

Optional
Coronal (organ specific or whole body)

a. Axial: post contrast e.g. T1 fat saturated volume interpolated gradient echo imaging (3D) breath hold of the *liver (60-70 seconds delay) and lungs*. Multiple breath-holds employed to provide full volume coverage if required. A minimum of a 256x256 (rectangular FOV acquisition if possible and appropriate for the patient) acquisition matrix should be employed. 5-7 mm slice thickness.
b. Coronal: post contrast whole body; e.g. T1 fat saturated volume interpolated gradient echo imaging (3D) and post contrast. Slice thickness 5mm. Breath Hold.
c. **Axial:** fat saturated T1 weighted imaging of the brain (SFOV). An acquisition matrix of 256 x 256 should be employed.

For peer review only - http://bmjopen.bmj.com/site/about/guidelines.xhtml

For peer review only

Appendix 4: Exploratory Analysis

The collated WB-MRI datasets and 12 months patient follow up data collated as part of the trial protocol provides opportunities to retrospectively mine the data for additional diagnostic indices and prognostic markers. All analysis will be done by members of the research team under supervision of the Chief Investigator.

There is for example, increasing data linking total body fat with prognosis in malignancy [30, 31]. In lung cancer, a higher body mass index has been associated with a better prognosis [32]. From the WB-MRI datasets acquired as part of the trial, it is possible to extract quantitative data on total body fat using simple segmentation algorithms which can be linked with patients' outcomes such as metastatic status and 12 month survival.

In addition, quantification of the MRI signal returned from sites of disease may be able to predict histological characteristics and also may be linked to prognosis. For example diffusion weighted imaging of the primary tumour has been shown to differentiate between grades of dysplasia in rectal cancer [33] and may predict prognosis in lung, brain and breast cancer [34-36]. The heterogeneity of signal within tumours can be measured and has been linked with overall prognosis and treatment response. For example in breast cancer, increased homogeneity of the signal measured using regions of interest placed in metastatic deposits in response to treatment is associated with a better prognosis [37]. The various sequences performed as part of the collected WB-MRI protocol will allow testing of such associations in recruited patients.

The following or similar may be quantified in all or a subset of WB-MRI datasets:

1) Body fat (organ, visceral and whole body).
2) MRI signal from the primary tumour and metastatic sites, notably diffusion weighted signal, apparent diffusion coefficient, T1 (including fat and water components), T2 signal, Contrast enhancement, and textural analysis (for heterogeneity) of the primary and metastatic deposits on the various sequences.

We will look for correlations between MRI variables and clinical data collated as part of the trial (for example basic histology, metastatic status, patient outcome and findings of conventional staging tests) or collected as part of the patients' usual care, for example, more detailed histology of biopsies or excised tumour (including tumours markers if collected as part of usual clinical care). Associations will be sought between the MRI variables and the clinical variables using appropriate conventional parametric and non-parametric statistics, for example:

- Differences in body fat composition between different cancer histological subtypes, patients with and without metastatic disease, and according to 12 month survival.
- Heterogeneity of MRI signal from a region of interest drawn within the primary tumours and metastatic deposits, comparing between lesions in the same

For peer review only - http://bmjopen.bmj.com/site/about/guidelines.xhtml

patient, between different patients and according to histological parameters and 12 month survival.

In addition comparisons will be made between MRI signal within normal body tissues (such as the liver, pancreas and muscle) between patients scanned on the different MRI scanners comparing with local MRI phantom data as appropriate to see how homogenous the MRI data is between different imaging hubs.

In some instances use of external commercial software may be required (e.g. fat quantification). MRI datasets will be anonymised and no personal identifiable information will be shared outside the research team at any time.

For peer review only

For peer review only - http://bmjopen.bmj.com/site/about/guidelines.xhtml

Appendix 5: Protocol Version History

**Protocol:**		**Amendments:**		
**Version no.**	**Date**	**Amendment no.**	**Protocol Section (no./title)**	**Summary of main changes from previous version.**
1.0				
1.1	14/09/12	N/A	Front cover & Section 1.1<br>Section 5<br>8.2.10<br>Section 14.2<br><br>Appendix 2	ISRCTN number added and new CR UK logo added<br>Flowchart and inserted re consent process<br>Text inserted re QoLs<br>Text inserted re destruction of interview recordings<br>Text inserted re consent process
2.0	15/03/13	1.0	Throughout<br>Section 4<br><br>Section 5<br><br><br>Section 7<br><br>Section 8<br><br><br><br><br><br>Section 9<br><br>Section 12<br>Section 13<br>Appendix 3	Typos and clarifications<br>Clarification about GCP training and documents required for activation updated<br>Details added about interpreters for informed consent and removal of informed consent log information<br>Removal of text regarding slot availability updates<br>Addition of ad hoc mini MDT, reduce the amount of patient experience questionnaires, change the content of these questionnaires and alter who is sending them to patients. Clarifications of timing of WB-MRI and the process of early release of findings<br>Update data management guidelines in line with changes at UCL CTC<br>Details removed about informed consent log<br>Addition of details re patient withdrawal<br>Clarifications to Whole body MRI protocol
3.0	24/04/14	3.0	Section 1<br>Section 5<br><br>Section 6<br><br>Section 7<br><br>Section 8	Rewording of the inclusion criteria<br>Removal of information about QoL and Patient diaries given out by site at consent<br>Rewording of the MDT log information and inclusion criteria<br>Removal of patient diaries and QoLs from registration packs.<br>Rewording and clarification of Conventional Imaging, WB-MRI reveal and MDT discussion, Final Treatment decision and follow up sections. Updating information to state that the Interview section of the trial is now closed to recruitment. Updating information that UCL CTC is now posting out QoL and patient diaries at baseline.

For peer review only - http://bmjopen.bmj.com/site/about/guidelines.xhtml

Streamline L

4.0	22/12/14	4.0	Section 8.2.12<br><br>Section 10<br><br>Appendix 4<br><br>Appendix 5	Information added regarding exploratory analysis for additional diagnostic indices and prognostic markers.<br>Safety Reporting section updated to provide more information on staging complications and how these are reported and reviewed by the CI.<br>Exploratory Analysis added to include the additional analysis of WB-MRI scans<br>Protocol Version History has become Appendix 5.
5.0	11/05/15	5.0	Section 8<br><br>Section 10<br><br>Section 13<br><br>Section 16	Changing target recruitment for patient experience questionnaires to 50 patients and closing this part of the trial. Updating and clarifying information regarding the Discrete Choice Experiment and confirming the time point these will be sent to patients.<br>Updating the Safety Section to confirm that only staging complications related to the WB-MRI or additional tests performed due to WB-MRI are to be recorded on the Staging Complications CRF.<br>The withdrawal process was updated and clarified to better match the new Change of Status CRF.<br>The type of Interim safety data that will be supplied to IDMC was updated
6.0	06/09/16	6.0	Sections 1.1 & 1.2<br><br>Section 13<br><br>Section 16.1	Recruitment target increased from 250 to 353<br>Anticipated duration of recruitment increased from 3 to 4 years.<br>Deletion of section on early release of WB MRI and patient failure to undergo WB MRI<br>Rationale for revised target accrual added<br>Grammatical and typographical errors corrected throughout

For peer review only

For peer review only - http://bmjopen.bmj.com/site/about/guidelines.xhtml

Streamline C

Streamlining Staging of Colorectal Cancer with Whole Body MRI

Trial Sponsor: University College London (UCL)
Trial Sponsor reference: UCL/11/0097

Trial funder: NIHR HTA
Funder reference: 10/68/01
ISRCTN no: 43958015

Protocol version no: 6.0
Protocol version date: 06/09/2016

This project was funded by the National Institute for Health Research (NIHR) Health Technology Assessment (HTA) programme *(project number 10/68/01)*

For peer review only

Protocol version 6.0, 06/09/2016 Authorisation signatures

Name & Role:	Signature:	Date authorised:
**Chief Investigator:**<br>Professor Stuart Taylor<br>Professor of Medical Imaging, UCL		14/10/16
**For the Sponsor:**<br>Professor Jonathan Ledermann<br>Director, UCL CTC		14 Oct 2016
Sandy Beare<br>Tumour Group Lead, UCL CTC		14/10/2016

Please note: This trial protocol must not be applied to patients treated outside the Streamline C trial. UCL CTC can only ensure that approved trial investigators are provided with amendments to the protocol.

For peer review only - http://bmjopen.bmj.com/site/about/guidelines.xhtml

Coordinating Centre:

For general queries, supply of trial documentation and central data management please contact:

Streamline C Trial Coordinator
Cancer Research UK & UCL Cancer Trials Centre (UCL CTC)
90 Tottenham Court Road
London
W1T 4TJ

Tel: +44 (0) 20 7679 9878
Fax: +44 (0) 20 7679 9871
09:00 to 17:00 Monday to Friday

Email: ctc.streamlineC@ucl.ac.uk

Other trial contacts:

Chief Investigator: Professor Stuart Taylor
Address: Dept Specialist X Ray, Level 2 Podium,
University College London Hospital (UCLH)
235 Euston Road
London
NW1 2BU

Trial Management Group (TMG):

Prof Stuart Taylor	Professor of Medical Imaging	UCLH
Dr Dow-Mu Koh	Consultant Radiologist	The Royal Marsden
Prof Vicky Goh	Professor of Clinical Cancer Imaging	Guys and St Thomas
Prof Anwar Padhani	Clinical Lead in MRI	Mount Vernon
Dr Shonit Punwani	Senior Lecturer in Medical Imaging	UCLH
Prof Andrea Rockall	Professor of Cancer Imaging	Imperial College London
Prof Steve Halligan	Professor of Radiology	UCL
Dr Neal Navani	Consultant in Thoracic Medicine	UCLH
Prof Sam Janes	Wellcome Senior Fellow	UCLH
Dr John Bridgewater	Consultant Oncologist	UCLH
Alison Morton	Patient Representative	
Alf Oliver	Patient Representative	
Dr Anne Miles	Senior Lecturer in Health Psychology	Birkbeck College
Prof Steve Morris	Professor of Health Economics	UCL
Dr Susan Mallett	Senior Medical Statistician	University of Birmingham
Krystyna Reczko	Trial Coordinator	UCL CTC
Katie Prior	Data Manager	UCL CTC
Marian Duggan (maternity leave)	Senior Trial Coordinator	UCL CTC
Abby Sharp (maternity cover)	Senior Trial Coordinator	UCL CTC
Dr Sandy Beare	Tumour Group Lead	UCL CTC

For peer review only - http://bmjopen.bmj.com/site/about/guidelines.xhtml

Table of Contents

1. PROTOCOL SUMMARY 7
1.1. SUMMARY OF TRIAL DESIGN 7
1.2. TRIAL SCHEMA 9
2. INTRODUCTION 10
2.1. BACKGROUND 10
3. TRIAL OVERVIEW 12
3.1. PRIMARY OBJECTIVE 12
3.2. SECONDARY OBJECTIVES 12
3.3. TRIAL ACTIVATION 13
4. SELECTION OF SITES/SITE INVESTIGATORS 14
4.1. SITE SELECTION 14
4.1.1. Selection of Principal Investigator and other investigators at sites 14
4.1.2. Training requirements for site staff 14
4.2. SITE INITIATION AND ACTIVATION 15
4.2.1. Site initiation 15
4.2.2. Required documentation 15
4.2.3. Site activation letter 15
5. INFORMED CONSENT 16
6. SELECTION OF PATIENTS 18
6.1. PRE-REGISTRATION EVALUATION 18
6.2. SCREENING LOG 18
6.3. MULTI DISCIPLINARY TEAM (MDT) MEETING LOG 18
6.4. PATIENT ELIGIBILITY 18
6.4.1. Patient Inclusion Criteria 18
6.4.2. Exclusion Criteria 19
6.4.3. Pregnancy and Birth Control 19
7. REGISTRATION PROCEDURES 20
7.1. REGISTRATION 20
7.2. REGISTRATION PACKS 20
7.3. BOOKING WHOLE BODY MRI 20
8. PATIENT MANAGEMENT AND TRIAL PROCEDURES 22
8.1. SUMMARY 22
8.1.1. Staging and Treatment Decisions 22
8.1.2. Patient experience Interviews and Questionnaires (Optional) 23
8.1.3. Quality of Life Questionnaires and Patient Diaries 23
8.2. DETAILED DESCRIPTION 25
8.2.1. Standard care 25
8.2.2. Trial Imaging 25
8.2.3. Release of Conventional and trial WB-MRI findings 27
8.2.4. Final treatment plan 28
8.2.5. Early release of WB-MRI findings 29
8.2.6. Time to full staging 29
8.2.7. Patient follow up 30
8.2.8. Patient diaries and EQ-5D Quality of Life questionnaires 30
8.2.9. Final reference standard for tumour stage (Consensus Meetings) 30
8.2.10. Health psychology assessment 32

For peer review only - http://bmjopen.bmj.com/site/about/guidelines.xhtml

8.2.11. *Cost Effectiveness Assessment* ... *35*
8.2.12. *Nested substudies* ... *37*

9. DATA MANAGEMENT GUIDELINES ... 39

9.1. Completing Case Report Forms ... 39
9.2. Missing Data ... 39
9.3. Timelines for data return ... 39
9.4. Data Queries ... 40

10. SAFETY REPORTING ... 41

11. INCIDENT REPORTING ... 42

12. TRIAL MONITORING AND OVERSIGHT ... 43

12.1. Central Monitoring ... 43
12.2. 'For Cause' On-Site Monitoring ... 43
12.3. Oversight Committees ... 44
12.3.1. *Trial Management Group (TMG)* ... *44*
12.3.2. *Trial Steering Committee (TSC)* ... *44*
12.3.3. *Independent Data Monitoring Committee (IDMC)* ... *44*
12.3.4. *Role of UCL CTC* ... *44*

13. WITHDRAWAL OF PATIENTS ... 45

14. TRIAL CLOSURE ... 46

14.1. End of Trial ... 46
14.2. Archiving of Trial Documentation ... 46
14.3. Early discontinuation of trial ... 46
14.4. Withdrawal from trial participation by a site ... 46

15. QUALITY ASSURANCE ... 47

16. STATISTICS ... 48

16.1. Sample size calculation ... 48
16.2. Population for analysis ... 49
16.3. Analysis of the primary endpoint ... 49
16.4. Analysis of secondary endpoints ... 50
16.4.1. *Efficacy (secondary)* ... *50*
16.4.2. *Safety* ... *52*
16.4.3. *Economic evaluation* ... *52*
16.4.4. *Health psychology assessment* ... *53*
16.4.5. *Handling missing data* ... *54*
16.5. Interim analyses ... 54

17. ETHICAL APPROVALS ... 55

17.1. Ethical Approval ... 55
17.2. Site Approvals ... 55
17.3. Protocol Amendments ... 55
17.4. Patient Confidentiality & Data Protection ... 56

18. SPONSORSHIP AND INDEMNITY ... 57

18.1. Sponsor Details ... 57
18.2. Indemnity ... 57

19. FUNDING ... 58

20. PUBLICATION POLICY ... 59

For peer review only - http://bmjopen.bmj.com/site/about/guidelines.xhtml

Streamline C

21. REFERENCES ... 60
APPENDIX 1: ABBREVIATIONS ... 63
APPENDIX 2: CONSENT PROCESS ... 65
APPENDIX 3: MINIMUM WB-MRI PROTOCOL DATASET ... 67
APPENDIX 4: EXPLORATORY ANALYSIS ... 69
APPENDIX 5: PROTOCOL VERSION HISTORY ... 71

For peer review only

For peer review only - http://bmjopen.bmj.com/site/about/guidelines.xhtml

1. Protocol Summary

1.1. Summary of Trial Design

**Title:**	Streamlining Staging of Colorectal Cancer with Whole Body MRI
**Short Title/acronym:**	Streamline C
**Sponsor name & reference:**	UCL/11/0097
**Funder name & reference:**	NIHR HTA
**ISRCTN no:**	43958015
**Design:**	Multicentre comparison
**Overall aim:**	To evaluate whether early whole body magnetic resonance Imaging (WB-MRI) increases per patient sensitivity for metastasis in colorectal cancer compared to standard NICE-approved diagnostic pathways.
**Primary endpoint:**	Per patient sensitivity for metastasis detection by whole body MRI (WB-MRI) compared to standard staging pathways in newly diagnosed colorectal cancer
**Secondary endpoints:**	1. The time and test number taken to reach, and the nature of, the first major treatment decision based on WB-MRI in comparison to standard staging pathways.<br>2. Diagnostic accuracy of WB-MRI and conventional staging pathways for local tumour staging and detection of metastasis in comparison to an expert derived consensus reference standard.<br>3. Lifetime incremental cost and cost-effectiveness of staging using WB-MRI compared to standard diagnostic pathways.<br>4. Patient experience of staging using WB-MRI in comparison to standard diagnostic pathways and priorities placed by patients on differing attributes related to competing staging pathways.<br>5. Inter-observer variability in WB-MRI analysis and effect of diagnostic confidence on staging accuracy.<br>6. Diagnostic accuracy of limited T1 and diffusion weighted sequences compared to full multi-sequence WB-MRI protocols.
**Target accrual:**	360
**Inclusion & exclusion criteria:**	**Inclusion criteria:**<br>• Adult patients (18 or over) with histologically

For peer review only - http://bmjopen.bmj.com/site/about/guidelines.xhtml

	proven or suspected colorectal cancer referred for staging.<br>• Suspicion of colorectal cancer defined as: Presence of a mass highly suspicious for colorectal cancer on endoscopy, barium enema, CT colonography or other imaging which triggers staging investigations.<br>• Patient must have given written informed consent and be willing to comply with the protocol intervention and follow up.<br>**Exclusion criteria:**<br>• Any psychiatric or other disorder likely to impact on informed consent<br>• Evidence of severe or uncontrolled systemic disease which make it undesirable for the patient to participate in the trial<br>• Pregnancy<br>• Contraindications to MRI (e.g. cardiac pacemaker, severe claustrophobia, inability to lie flat)<br>• Polyp cancer (because metastatic disease in these patients is vanishingly rare)
**Planned number of sites:**	14 (including 7 Imaging hubs)
**Target Country**	UK
**Trial Procedure:**	All patients will undergo a whole body MRI protocol in addition to the standard staging protocol employed at their institution.
**Anticipated duration of recruitment:**	4 years
**Duration of patient follow up:**	12 months
**Definition of end of trial:**	12 months after enrolment of the final patient.
**Other related research:**	Sub-study of WB-MRI generalisability

For peer review only

For peer review only - http://bmjopen.bmj.com/site/about/guidelines.xhtml

1.2. Trial Schema

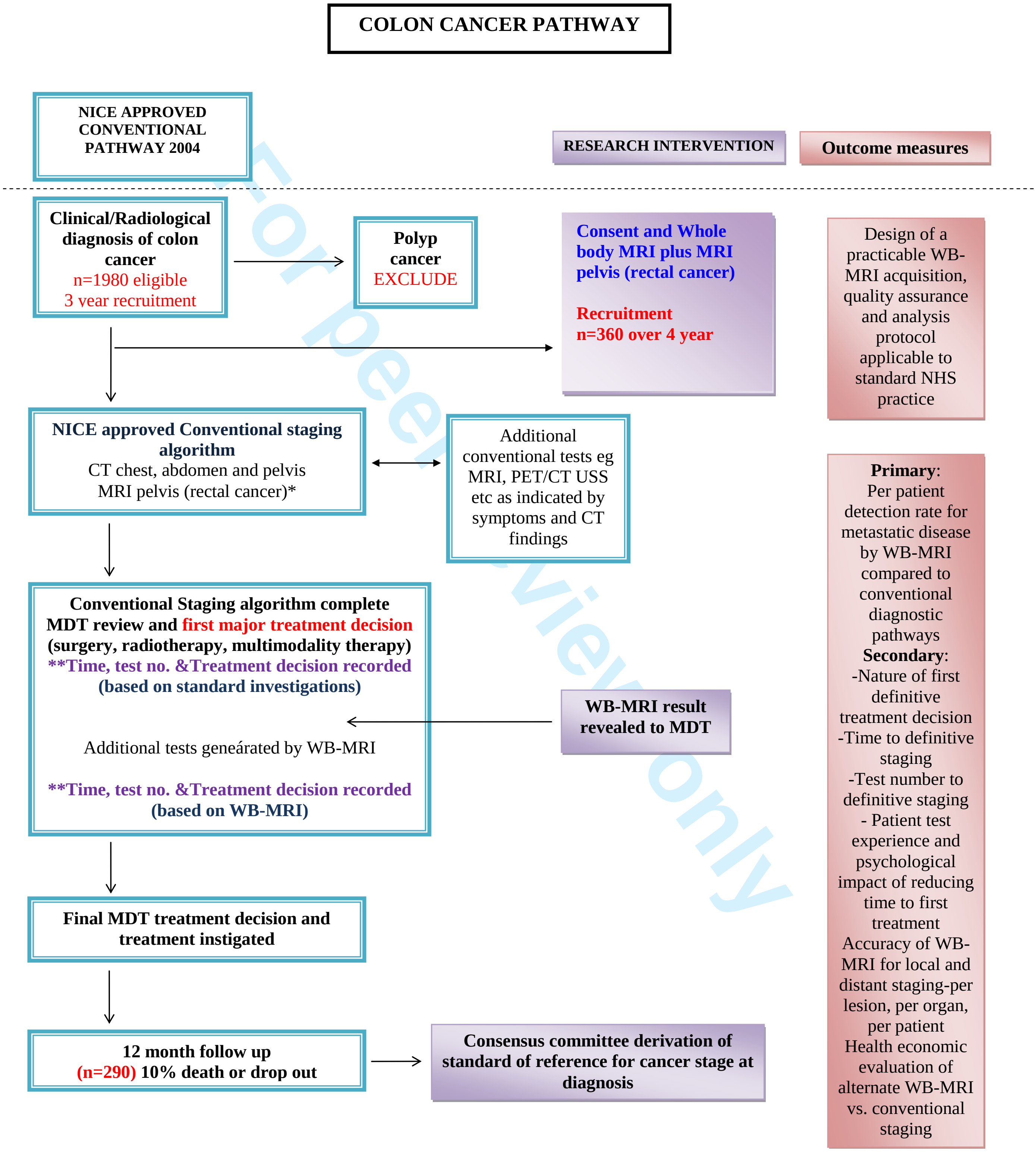

For peer review only

For peer review only - http://bmjopen.bmj.com/site/about/guidelines.xhtml

2. Introduction

2.1. Background

Colorectal cancer is the third most common malignancy in the UK and accounts for 13% of all new cancer diagnosis; approximately 40,000 patients are diagnosed per year [1]. Treatment decisions for colorectal cancer are critically dependent upon rapid and accurate tumour staging at diagnosis. In particular detection of metastatic disease (i.e. disease which has disseminated beyond the primary tumour into distant organ sites such as bone, liver and brain) is vital to optimised therapeutic triage. More aggressive approaches to treating metastatic colorectal cancer using combined chemotherapy and surgical resection regimens are widely adopted in routine National Health Service (NHS) practice [2].

Success rates however depend on the accuracy of initial disease staging so all disease sites are appropriately identified and treated [3]. Currently around 80% of patients with colorectal cancer undergo primary surgery with curative intent, although up to 50% subsequently relapse with metastatic disease, often within 12 months [4], in part reflecting sub-optimal disease staging. Accurate local staging of the tumour and spread to lymph nodes is also of importance. Imaging derived local staging in rectal cancer dictates the use of pre-operative (neo-adjuvant) chemotherapy. Furthermore, trials investigating neoadjuvant chemotherapy for non-rectal colonic tumours are currently recruiting in the NHS (FOXTROT).

Staging of colorectal cancer within the NHS is reliant on high technology imaging platforms such as computerised tomography (CT), positron emission tomography (PET) and magnetic resonance imaging (MRI), together with standard scintigraphy, plain X-rays and ultrasound. Staging pathways are complex (see flowchart, section 1.2), and governed by National Institute for Clinical Excellence (NICE) approved guidelines [5] which delineate an integrated step by step deployment of the various imaging modalities before tumour staging is deemed complete and the first treatment decision is made.

The complexity of current staging algorithms reflects the differing diagnostic attributes of the various imaging modalities across tumour types and body organs. CT for example is superior to ultrasound for detecting liver metastasis [6], but inferior to scintigraphy in diagnosing malignant spread to the bone. PET scanning is in general superior to scintigraphy for detecting bone metastases [7], but not as effective as MRI in identifying spread to the brain [8]. It is not unusual for a patient with colorectal cancer to undergo CT chest abdomen and pelvis, together with pelvic MRI in the case of rectal cancer. However additional tests such as ultrasound, MRI and PET-CT are increasingly used in cases of known or suspected metastatic disease. Such a stepwise multi-modality approach is time and resource intensive and onerous for patients at a very difficult time following their cancer diagnosis. Furthermore patients receive significant doses of diagnostic ionising radiation during the staging process; a single PET-CT imparts 6-10 times normal annual background radiation and even small doses of radiation can increase an individual's risk of subsequent malignancy [9].

Recent technological advances mean an MRI scan of the whole body can be acquired in less than one hour; sensitivity for metastatic cancer spread is high [10]. MRI does

For peer review only - http://bmjopen.bmj.com/site/about/guidelines.xhtml

not impart ionising radiation and is advocated as a safe and effective alternative to the current multi-modality approach. There is no available secondary research evidence concerning the accuracy of whole body MRI (WB-MRI) in cancer staging. Indeed meta-analysis would be challenging because literature is limited to relatively small cohort studies comparing WB-MRI with conventional imaging tests, and in a wide variety of cancers. These do however suggest potential as an efficient and accurate "one stop shop" alternative for cancer staging [8, 11, 12]. For example, in 123 patients with non-small cell cancer, Yi found no difference between PET-CT and WB-MRI, with both achieving 86% accuracy [12]. Notably, the vast majority of available studies are single site, using one MRI platform, with interpretation by one or two experienced radiologists.

The literature is deficient regarding generalisability of WB-MRI. For example, variability amongst interpreting radiologists is unknown and the effect (if any) of different manufacturers' platforms is uncertain. It is also unclear how WB-MRI can be best integrated into existing NHS pathways, which frequently differ between institutions, or how effective it would be in different clinical settings. By undertaking a multicentre trial of colorectal cancer using a variety of MRI platforms and interpreting radiologists, the current proposal will address these unknown variables.

Health psychology assessment

WB-MRI staging of colorectal cancer could reduce the physical and psychological burden associated with current pathways. By reducing time from diagnosis to treatment, WB-MRI may reduce patient anxiety, minimising concerns that treatment delay might adversely affect prognosis. Delay in diagnosis is known to be distressing, particularly for women [13]. In addition, staging scans can be uncomfortable and embarrassing [14]. MRI for example, while avoiding radiation, is noisy and claustrophobic. The potential of WB-MRI to generate additional tests is also an important consideration, although recent data suggest test sensitivity is the key determinant of patient choice and influences preference more than discomfort and complications [15]. While rapid staging means patients without metastasis are in receipt of good news more quickly, it also means those with metastasis will also receive bad news earlier, which may impact adversely on psychological wellbeing: It is known that patients are more depressed following a speedier diagnosis of breast cancer (at a one-stop clinic) when compared to those waiting a week [16].

Cost Effectiveness Analysis

Although WB-MRI is a relatively expensive imaging technology, other standard tests in the staging pathways such as PET-CT are also resource intensive. A detailed analysis of the cost and the cost-effectiveness of WB-MRI versus standard NICE-approved staging algorithms conforming to accepted economic evaluation methods [17] is essential to any trial investigating the role of WB-MRI in cancer staging.

For peer review only - http://bmjopen.bmj.com/site/about/guidelines.xhtml

3. Trial Overview

This trial will be an independent multi-centre prospective cohort trial comparing the staging accuracy of early WB-MRI with standard pathways in newly diagnosed colorectal cancer. We will follow the recommendations of the STAndards for the Reporting of Diagnostic accuracy studies (STARD) initiative [18]. A key element of our design is the investigation of early WB-MRI as a replacement test for standard pathways [19], and data regarding the therapeutic impact of WB-MRI will be collated, as well as addressing the practicalities of general NHS implementation. The trial design, which compares two different staging strategies in the same patients ensures that clean, comparable data are collected for both pathways while simultaneously increasing trial power to meet trial endpoints.

In addition, both qualitative and quantitative assessments will be employed to determine the psychological burden and acceptability of WB-MRI versus standard pathways, and to identify those attributes which most strongly influence patient preference. Health-related quality of life data will be collected which will be used to inform cost-effectiveness modelling, and determine whether patients believe more rapid staging would have helped them cope better with their cancer diagnosis.

3.1. Primary objective

To evaluate whether early whole body magnetic resonance Imaging (WB-MRI) increases per patient sensitivity for metastasis in colorectal cancer compared to standard NICE-approved diagnostic pathways.

3.2. Secondary objectives

1) To determine how WB-MRI influences time to and nature of first major treatment decision following definitive staging compared to standard investigations and to determine whether early WB-MRI could reduce or replace standard investigations.
2) To assess the accuracy of WB-MRI and standard diagnostic pathways for local and distant staging by comparison with an expert derived consensus reference standard using 1-year patient follow up data.
3) To evaluate the lifetime incremental cost and cost-effectiveness of staging using WB-MRI compared to standard diagnostic pathways.
4) To evaluate patients' experiences of staging using WB-MRI and to determine the priorities placed by patients on differing attributes related to competing staging pathways, including impact of reducing time to first treatment.
5) To determine the effect of radiologist confidence on the diagnostic accuracy of WB-MRI for metastatic disease.
6) To evaluate the diagnostic performance of limited WB-MRI protocols based on diffusion and T1 weighted imaging only, and to assess the incremental benefit of intravenous gadolinium contrast enhancement.

Additional objectives which will be met by the trial will be the definition of an efficient WB-MRI acquisition and analysis protocol, and quality assurance (QA) program

For peer review only - http://bmjopen.bmj.com/site/about/guidelines.xhtml

applicable to standard NHS practice and collation of a database of WB-MRI datasets for NHS staff training and service development.

3.3. Trial activation

UCL CTC will ensure that all trial documentation has been reviewed and approved by all relevant bodies and that the following have been obtained prior to activating the trial:

- Research Ethics Committee approval
- 'Adoption' into NIHR portfolio
- NHS permissions
- Adequate funding for central coordination
- Confirmation of sponsorship
- Adequate insurance provision

For peer review only

For peer review only - http://bmjopen.bmj.com/site/about/guidelines.xhtml

4. Selection of Sites/Site Investigators

4.1. Site selection

In this protocol a 'recruitment site' refers to a hospital where certain trial-related activities are conducted.

Recruitment sites must be able to comply with:

- Patient recruitment, follow up schedules and all requirements of the trial protocol
- Requirements of the Research Governance Framework
- Data collection requirements
- **No** trial imaging, recruitment sites will refer patients to designated imaging hub (see below)

In this protocol an 'imaging hub' refers to a hospital where certain trial-related activities are conducted.

Imaging hubs must be able to comply with:

- Patient recruitment (where applicable), follow up schedules (where applicable) and all requirements of the trial protocol
- Requirements of the Research Governance Framework
- Data collection requirements
- Trial imaging

4.1.1. Selection of Principal Investigator and other investigators at sites

Sites must have an appropriate Principal Investigator (PI), i.e. a health care professional authorised by the site and ethics committee, to lead and coordinate the work of the trial on behalf of the site. Other investigators at site wishing to participate in the trial must be trained and approved by the PI. All investigators must be medical doctors and have experience of diagnosing, staging or treating colorectal cancer.

4.1.2. Training requirements for site staff

All site staff must be appropriately qualified by education, training and experience to perform the trial related duties allocated to them, which must be recorded on the site delegation log.

CVs for all staff must be kept up to date, with signed and dated and copies held in the Investigator Site File (ISF). An up to date, signed copy of the CV for the PI must be forwarded to UCL CTC upon request.

All staff involved in the trial must receive GCP training which is relevant to their role and responsibilities within the trial. The frequency of repeat training may be dictated by the requirements of their employing institution, or 2 yearly where the institution has no policy, and more frequently when there have been updates to the legal or regulatory requirements for the conduct of clinical trials.

For peer review only - http://bmjopen.bmj.com/site/about/guidelines.xhtml

4.2. Site initiation and activation

4.2.1. Site initiation

Before a site is activated, the UCL CTC trial team will arrange a site initiation with the site which the PI and site research team must attend. The site will be trained in the day to day management of the trial, and essential documentation required for the trial will be checked.

Site initiation will be performed for each site by site visit or teleconference.

4.2.2. Required documentation

The following documentation must be submitted by the site to UCL CTC prior to a site being activated by UCL CTC trial team:

- Trial specific Declaration of Participation/Site Registration Form (identifying relevant local staff)
- All relevant institutional approvals (e.g. local NHS permission)
- A completed site delegation log that is initialled and dated by the PI
- A copy of the PI's current CV that is signed and dated

In addition, the following agreements must be in place:

- A signed Clinical Trial Site Agreement (CTSA) between the Sponsor and the relevant institution (usually a NHS Trust)

4.2.3. Site activation letter

Once the UCL CTC trial team has received all required documentation and the site has been initiated, a site activation letter will be issued to the PI. **Sites may not begin to approach patients until the site activation letter has been issued.**

Once the site has been activated by UCL CTC, the PI is responsible for ensuring:

- adherence to the most recent version of the protocol;
- all relevant site staff are trained in the protocol requirements;
- appropriate recruitment and medical care of patients in the trial;
- timely completion and return of CRFs (including assessment of all adverse events).

For peer review only - http://bmjopen.bmj.com/site/about/guidelines.xhtml

5. Informed consent

Sites are responsible for assessing a patient's capacity to give informed consent.

Sites must ensure that all patients have been given the current approved version of the patient information sheet, are fully informed about the trial and have confirmed their willingness to take part in the trial by signing the current approved consent form.

Sites must assess a patient's ability to understand verbal and written information in English and whether or not an interpreter would be required to ensure fully informed consent. If a patient requires an interpreter and none is available, the patient should not be considered for the trial.

The PI, or, where delegated by the PI, other appropriately trained site staff, are required to provide a full explanation of the trial to each patient prior to trial entry. During these discussions, the current approved patient information sheet for the trial should be discussed with the patient. A minimum of twenty four hours before the WB-MRI must be allowed for the patient to consider and discuss participation in the trial.

The consent process has been left deliberately flexible in order to accommodate the needs of individual patients and variations in Site requirements. The flowchart below describes three different options for obtaining written informed consent from patients, each of which ensures that patients have at least 24 hours to consider their participation in the trial before any trial related activities take place.

Streamline Recruitment Pathways/Consent Process

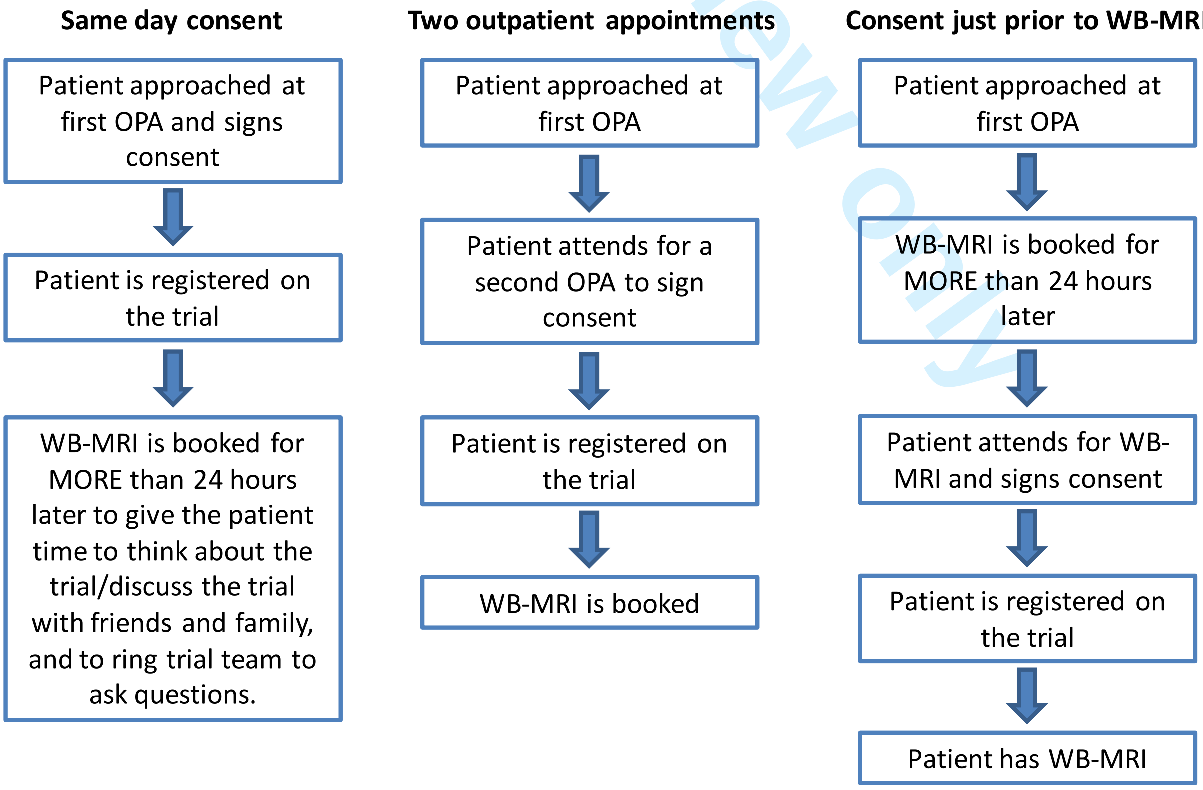

For peer review only - http://bmjopen.bmj.com/site/about/guidelines.xhtml

For peer review only

See Appendix 2 for a more detailed example of the consent process.

Written informed consent on the current approved version of the consent form for the trial must be obtained before any trial-specific procedures are conducted. The discussion and consent process must be documented in the patient notes.

Site staff are responsible for:

- checking that the correct (current approved) version of the patient information sheet and consent form are used;
- checking that information on the consent form is complete and legible;
- checking that the patient has completed/initialled all relevant sections and signed and dated the form;
- checking that an appropriate member of staff has countersigned and dated the consent form to confirm that they provided information to the patient;
- checking that an appropriate member of staff has made dated entries in the patient's medical notes relating to the informed consent process (i.e. information given, consent signed etc.);
- Following registration:
    - adding the patient trial number to all copies of the consent form, which should be filed in the patient's medical notes and investigator site file.
    - giving the patient a copy of their signed consent form and patient information sheet.

The right of the patient to refuse to participate in the trial without giving reasons must be respected. All patients are free to withdraw at any time. Also refer to section 13.0 (Withdrawal of patients).

For peer review only - http://bmjopen.bmj.com/site/about/guidelines.xhtml

6. Selection of Patients

6.1. Pre-registration Evaluation

The following assessments or procedures are required to evaluate the suitability of patients for the trial:

- Suspicion of colorectal cancer defined as: Presence of a mass highly suspicious for colorectal cancer on endoscopy, barium enema, CT colonography or other imaging which triggers staging investigations
- No contraindications to MRI (e.g. cardiac pacemaker, severe claustrophobia, inability to lie flat)

6.2. Screening Log

A screening log must be maintained by the site and kept in the Investigator Site File. This must record all potentially eligible patients approached about the trial and the reasons why they were not registered in the trial if this is the case. The log must be sent to UCL CTC in line with the monitoring plan and as requested, with patient identifiers removed prior to sending.

6.3. Multi Disciplinary Team (MDT) Meeting Log

An anonymous record of the number of patients with a new diagnosis of colorectal cancer discussed in the MDT meeting, with their attributed cancer stage (TNM or Dukes) will be submitted annually to UCL CTC. This will be used to assess whether the group of patients recruited to the trial is representative of the general population of newly diagnosed colorectal cancer patients seen at Sites.

6.4. Patient Eligibility

There will be no exception to the eligibility requirements at the time of registration. Queries in relation to the eligibility criteria must be addressed prior to calling/faxing for registration. Patients are eligible for the trial if all the inclusion criteria are met and none of the exclusion criteria applies.

6.4.1. Patient Inclusion Criteria

- Adult patients (18 or over) with histologically proven or suspected colorectal cancer referred for staging.
- Suspicion of colorectal cancer defined as: Presence of a mass highly suspicious for colorectal cancer on endoscopy, barium enema, CT colonography or other imaging which triggers staging investigations.
- Patient must have given written informed consent and be willing to comply with the protocol intervention and follow up.

For peer review only

For peer review only - http://bmjopen.bmj.com/site/about/guidelines.xhtml

6.4.2. Exclusion Criteria

- Any psychiatric or other disorder likely to impact on informed consent.
- Evidence of severe or uncontrolled systemic disease which make it undesirable for the patient to participate in the trial.
- Pregnancy.
- Contraindications to MRI (e.g. cardiac pacemaker, severe claustrophobia, inability to lie flat).
- Polyp cancer (because metastatic disease in these patients is vanishingly rare).

6.4.3. Pregnancy and Birth Control

MRI poses a theoretical risk to the foetus, particularly in the first trimester due to local acoustic and heating effects. However the risk is generally deemed very small and significantly less than the risk of ionising radiation imparted by PET-CT and CT (standard imaging investigations).

A woman of childbearing potential (WCBP) is a sexually mature woman (i.e. any female who has ever experienced menstrual bleeding) and who has not undergone a hysterectomy or who has not been postmenopausal for 24 consecutive months (i.e. who has had menses at any time in the preceding 24 consecutive months). Patients will be excluded if they are pregnant.

There is no requirement for additional contraceptive advice to patients over and above that routinely given as part of their routine clinical care given their diagnoses of colorectal cancer.

The need to perform a pregnancy test in WCBP will be decided as part of the patient's routine clinical care given the risk to pregnancy from standard staging investigations and subsequent treatment. There is no requirement to perform a pregnancy test purely because of recruitment to the trial if this would not have been performed as part of standard clinical care.

For peer review only

For peer review only - http://bmjopen.bmj.com/site/about/guidelines.xhtml

7. Registration Procedures

7.1. Registration

Patient registration will be undertaken centrally at UCL CTC and this must be performed prior to commencement of any trial intervention. Registration can be performed via telephone or fax.

Following pre-registration evaluations (as detailed in section 6.1), confirmation of eligibility and consent of a patient at a site, the registration form must be fully completed prior to faxing or telephoning UCL CTC. The eligibility criteria will be reviewed during the registration telephone call using the same form at UCL CTC.

A trial number will be assigned for the patient during the call and must be recorded at site by the caller. If desired, the site may fax a copy of the completed registration form to UCL CTC and the faxed registration from will be used it confirm patient eligibility at UCL CTC.

UCL CTC will email confirmation of the patient's inclusion in the trial and their trial number to the main contact, PI and Imaging Hub contact. If requested, a fax confirmation may also be sent. Case report forms will be emailed to the main contact at site.

Registration telephone number:	+44 (0)20 7679 9880
Registration fax number:	+44 (0)20 7679 9871
UCL CTC Office hours:	09:00 to 17:00 Monday to Friday

7.2. Registration Packs

Once a patient has consented to take part in the trial they should be provided with a registration pack to include the following:

- A copy of their signed consent form and patient information sheet
- An expenses claim form (to claim travel expenses to and from WB-MRI appointment)
- Pre-paid return envelope

7.3. Booking Whole Body MRI

Once a patient has been registered it is the responsibility of the recruiting site to contact their designated imaging hub to make arrangements for the WB-MRI. Each recruitment site will be provided with a contact number at their designated imaging hub. Recruitment sites will approach consecutive potentially eligible patients until the number of recruited patients fills the MRI capacity of the imaging hub.

For peer review only - http://bmjopen.bmj.com/site/about/guidelines.xhtml

It is possible recruitment potential at recruitment sites will be greater than the WB-MRI capacity at each central imaging hub. If a patient is approached and registered for the trial but MRI capacity is such that the scan could not take place in a timely fashion, a decision to withdraw the patient will be made by the recruitment site and the patient will then be informed. This possibility is explained to patients in the patient information sheet.

Because of the need to perform WB-MRI in a timely fashion, it is anticipated recruitment sites will be requested to pause recruitment if WB-MRI waiting time exceeds 2 weeks (if WB-MRI is performed after conventional imaging is complete) or 3 weeks (if WB-MRI is to be performed concurrently with standard tests). There will be flexibility in these timings depending on the circumstances of individual patients and their anticipated time to complete standard staging.

For peer review only

For peer review only - http://bmjopen.bmj.com/site/about/guidelines.xhtml

8. Patient Management and Trial Procedures

8.1. Summary

8.1.1. Staging and Treatment Decisions

The following information is required for the trial to document staging and treatment decisions:

- Stage and treatment decision based on conventional imaging only (and the number, timing nature and findings of these investigations)
- Stage and theoretical treatment decision based on WB-MRI only (and any additional tests generated by the WB-MRI) only (and the number, timing nature and findings of additional tests generated)
- A final treatment decision incorporating all available tests.

Conventional Imaging

Trial patients will undergo the standard staging protocol employed at their institution as per usual clinical care pathways. See section 8.2.1 for further detail.

Whole Body MRI Imaging and MDT Reveal

Recruited patients will undergo a WB-MRI at one of the designated imaging hubs. Recruiting sites should contact their designated imaging hub to request a WB-MRI slot (see section 7.3). The WB-MRI should be performed either concurrently with the standard staging investigations, or no later than 3 weeks after the final standard staging investigation. Images will be uploaded at each hub to a secure central imaging server called 3Dnet™. If a language interpreter was required for a patient during the consent process, then an interpreter should be made available to the patient during the WB-MRI. The WB-MRI will be reported by designated radiologist(s) at each imaging hub who are blinded to the conventional imaging tests and other clinical information (other than the cancer diagnosis and location). A copy of the report will also be uploaded to 3Dnet™. See section 8.2.2 for further detail.

Once the MDT has made a treatment decision based on the conventional imaging, the MDT coordinator, research nurse or other designated individual will reveal the WB-MRI images and report on 3Dnet™ via an internet-enabled PC. The MDT will review the WB-MRI results and decide whether or not any additional tests are required or would have theoretically been required (if in fact the test was already performed as part of conventional staging) in addition to the WB-MRI. If any tests are required that have not already been performed as part of the standard staging, they should be performed and the patient reviewed again at the next MDT when the results are available. If no further tests are needed then the stage and theoretical treatment decision based on the WB-MRI (and any additional tests generated from the WB-MRI) must then be made by the MDT. The MDT must then decide a final treatment decision for the patient based on all imaging available. If referral to the liver MDT is indicated, the final treatment decision based on WB-MRI will be that made by the liver MDT after review of the WB-MRI and generated tests. See section 8.2.3 for further detail.

For peer review only - http://bmjopen.bmj.com/site/about/guidelines.xhtml

If a patient is due to start treatment before the next formal MDT meeting, an ad hoc mini MDT meeting may be scheduled outside of the standard MDT meeting. This should include the relevant multidisciplinary clinical team members who are appropriate to make the final treatment decision based on the specific imaging finding, according to usual standards of clinical care. A record will be kept of who is present during the ad hoc mini MDTs on the CRFs.

8.1.2. Patient experience Interviews and Questionnaires (Optional)

Interviews (Study 1)

A total of 25 patients have undergone interviews by a Health Psychologist pertaining to their experience of the cancer staging process. **Target accrual has now been reached for the interview part of the trial and recruitment to this is now closed.**

Questionnaires (Study 2)

Patient experience questionnaires from baseline and post-staging have been received for approximately 50 patients to assess patient experience and acceptability of WB-MRI and standard tests. **Target accrual has now been reached for the questionnaire part of the trial and recruitment to this is now closed.**

Discrete choice experiment (Study 3)

A discrete choice experiment (DCE) questionnaire will be administered to a subset of approximately 50 patients to elicit their preference for various attributes associated with conventional and WB-MRI staging pathways. The DCE questionnaire is optional and patients will be asked to consent to this on trial entry. Once a patient consents to the DCE they will be allocated a DCE questionnaire (out of a possible 2 DCE questionnaires) which will be posted by UCL CTC along with their Quality of Life questionnaire, patient diary and pre-paid envelope immediately after registration (baseline). UCL CTC will supply the details of any recruited patients who consented to complete DCE questionnaire to the Health psychologists who may contact them directly.

For peer review only

8.1.3. Quality of Life Questionnaires and Patient Diaries

Upon consent the recruiting site must inform the patient that they will receive an EQ-5D Quality of Life Questionnaire and Patient Diary by post from UCL CTC and they are to complete these and return them using the pre-paid envelope provided. The patient diary will cover a 3 month period and will collect information regarding the patient's Primary Health Care contacts, other health care contacts and also the medication they are currently taking. Patient diaries and EQ-5D questionnaires will be posted to all patients at consent and then at 3 monthly intervals for 12 months.

For peer review only - http://bmjopen.bmj.com/site/about/guidelines.xhtml

Streamline C

Table 1: Summary of patient management and trial activities

Activity	Protocol section	Timing							Responsible organisation/person
		Prior to registration	Immediately after registration	During staging	After staging (months after registration)				
					1	3	6	9	
Pre-registration evaluation & assessment of eligibility	6.1	X							Recruiting site
Consent	5.0	X							Recruiting site
Register patient	7.1	X							Recruiting site
Give patient registration pack (copy of PIS and consent, expenses claim form and pre-paid return envelope)	7.2		X						Recruiting site
Book WB-MRI scan & inform patient of appointment date	7.3		X						Recruiting site
WB-MRI	8.2.2			X					Imaging Hub
Upload WB-MRI scan & report	8.2.2			X					Imaging Hub
Post EQ-5D questionnaires	8.2.8		X			X	X	X	UCL CTC
Post patient resource diary	8.2.8		X			X	X	X	UCL CTC
Discrete choice experiment Questionnaire	8.2.10		X						UCL CTC

For peer review only

For peer review only - http://bmjopen.bmj.com/site/about/guidelines.xhtml

8.2. Detailed description

8.2.1. Standard care

Trial patients will undergo the conventional staging investigations employed at their recruiting institution according to local protocols and the requirements of their clinical care team.

All conventional investigations will be performed and interpreted by the usual radiologists and clinicians employed at the site of the investigations. Standard clinical reports will be made and all investigations (and their results) will be freely available on hospital Picture Archiving and Communications System (PACS), Radiology Information System (RIS) and Clinical Data Repository (CDR) systems as per usual clinical practice.

The type and date of investigations (e.g. CT scan, PET-CT, organ specific MRI, biopsy etc) will be recorded on CRFs, along with the presence and location of metastatic disease based on the radiological report.

8.2.2. Trial Imaging

Whole Body MRI (WB-MRI) protocol

Recruited patients will undergo a WB-MRI at their designated imaging hub site. The choice of MRI platform (i.e. manufacturer and Tesla (T) strength) will be decided by the local hub radiologist according to scanner availability and their usual practice. It is anticipated most MRIs will be performed at 1.5T. Exact imaging parameters will vary according to MRI platform but a minimum dataset of sequences will be acquired (full details given in appendix 3).

Patients with rectal cancer would normally undergo staging pelvic MRI, and the WB-MRI will be additional to this. Where possible (and according to site and patient preference), the WB-MRI and rectal staging MRI would be performed on the same day to reduce patient travel.

Timing

The WB-MRI will be performed either concurrently with the standard staging investigations, or no later than 3 weeks after their final standard staging investigation.

Blinding

To ensure the integrity of the trial, the WB-MRI must be reported by a radiologist blinded to the conventional imaging tests and other clinical information (other than the cancer diagnosis and site). Conversely the WB-MRI images and reports must not be available to either those radiologists reporting the standard staging investigations, nor those involved in direct patient care before the WB-MRI is revealed in the MDT meeting.

The un-anonymised WB-MRI images will therefore not be immediately sent to the Picture Archive and Communication System (PACS) at either the imaging hub or recruitment sites. Instead images will be uploaded at each hub to a secure central

For peer review only - http://bmjopen.bmj.com/site/about/guidelines.xhtml

imaging server (3Dnet™) provided by Biotronics3D. This solution allows easy upload of MRI datasets via standard internet connection. A PC based internet gateway will be installed in each imaging hub to facilitate automated transfer of WB-MRI from the scanner/workstation to 3Dnet™), and thereafter back to PACS to the appropriate time point after MDT revelation (see below).

Interpretation and reporting

The WB-MRI will be interpreted by designated radiologist(s) at each imaging hub who are expert in interpretation based on previous experience of reporting WB-MRI in cancer staging. As noted above, the radiologist will be blinded to the standard staging investigations performed on recruited patients.

Interpretation can be performed using the 3Dnet™ software, or stand alone workstation according to the preference of the radiologist.

Images will be analysed in the following order:

1) Diffusion and non contrast enhanced T1 images
2) Diffusion, non contrast enhanced T1 and T2 images
3) Diffusion, non contrast enhanced T1, T2 and contrast enhanced T1 images.

After viewing each sequence set (and before reviewing the next set), the radiologist will complete WB-MRI Imaging Booklet CRFs documenting the presence, location and size of metastatic disease, together with their diagnostic confidence on a scale of 1 to 6. Reporting time for each sequence set analysis (defined as the time required to interrogate the sequences to reach a diagnostic conclusion) will be recorded along with the technical quality of the MRI dataset.

The reporting radiologist will then produce a free text clinical report as per their usual clinical practice (using all available sequences and based on the TMN 7 staging guidance) for subsequent release to the clinical team. This report will contain information relating to the local T and N stage of the tumour, together with the presence, location, number and size of metastatic deposits, as well as important “incidental” findings, for example aortic aneurysm. The radiologist may express their level of confidence in reported findings as they would in normal clinical practice but a formal numerical score of confidence will not be provided as this would not mirror how radiological examinations are reported in standard clinical care. If the radiologist would usually recommend additional tests for equivocal findings, this will be included in the report so as to also mirror routine clinical practice. The definitions of T, N M stage will be based on conventional MRI criteria adopted by the radiologist in their usual clinical practice.

The free text report will be uploaded onto the 3Dnet™ software, and a copy stored as part of the patient’s medical record.

For peer review only - http://bmjopen.bmj.com/site/about/guidelines.xhtml

8.2.3. Release of Conventional and trial WB-MRI findings

MDT discussion of the first major treatment decision based on conventional imaging

WB-MRI images and reports will be withheld initially from the clinical care team and radiologists reporting standard imaging (to avoid bias) until patients have completed all conventional investigations and have been definitively staged such that a first major treatment decision has been made by the MDT based on these conventional tests. The first major treatment decision based on conventional imaging will be defined as:

- Referral for surgical excision of either the primary tumour and/or a metastatic site
- Instigation of definitive treatment using chemotherapy, radiotherapy or a combination of the two
- Decision to offer palliative/supportive care only
- Request for a highly invasive surgical staging procedure such as surgical mediastinal lymph node sampling (mediastinoscopy), video-assisted thoracoscopic surgery (VATS), or laparoscopy

In some instances the first major treatment decision will not be made by the colorectal MDT, for example the patient being referred to a liver MDT. In this case the treatment decisions of the liver MDT made according to the protocols described below will be used for the purposes of the trial.

If additional conventional tests are required to complete staging, the WB-MRI result will be withheld until they are completed, and the patient re-discussed with the findings of the additional test(s).

The nature, date and findings of all conventional investigations will be recorded by the MDT, and the TNM 7 stage recorded. The MDT/appropriate MDT personnel will define the nature of the first treatment decision based on conventional investigations in the MDT record and on the CRF.

Release of WB-MRI images and report

Once a stage and treatment decision has been made based on conventional imaging, the WB-MRI images (supplemented where possible with “screen shots” and annotations of relevant findings) and radiologist’s report will be revealed to the MDT using the 3Dnet™ software on an internet enabled PC. If technical issues preclude use of 3Dnet™ in the MDT, presentation of a written copy of the WB-MRI report is permissible.

When the WB-MRI images and report are released, the MDT will discuss the images and decide:

1) *If the patient can be adequately staged based on WB-MRI alone:*

   In this case the MDT should state the patient’s stage and theoretical treatment decision based on the WB-MRI alone.

Or:

For peer review only - http://bmjopen.bmj.com/site/about/guidelines.xhtml

2) *If any additional tests are required based on the WB-MRI alone for adequate staging* (for example for equivocal WB-MRI findings):

   a. For additional tests that would have theoretically been requested based on the WB-MRI alone but have already been performed as part of the standard imaging, the results can be used by the MDT. The MDT personnel will state the patient's stage and theoretical treatment decision based on the WB-MRI and those additional tests that would have theoretically been generated.

   b. If the additional tests generated by WB-MRI have not already been performed as part of standard care and are deemed necessary in the opinion of the MDT these will be undertaken according to standard clinical care. After completion of the additional test(s), the patient will be re-discussed by the MDT and a final treatment decision made based on the WB-MRI and the additional test(s) undertaken.

If, following revelation of the findings of the WB-MRI (and any additional tests it generates), the colorectal MDT request review by the liver MDT, (which had not been the case based on standard imaging), for the purposes of the trial the final treatment decision based on WB-MRI will be that made by the liver MDT after review of the WB-MRI and generated tests. The fact that following WB-MRI a referral to the liver MDT is made will be recorded.

8.2.4. Final treatment plan

Once a stage and treatment decision has been made based on conventional imaging alone and also a stage and theoretical treatment decision made based on the WB-MRI (and any additional tests it generates) alone a final treatment plan can be made by the MDT. The final treatment plan for the patient will be based on all available information (including the WB-MRI). The MDT radiologist(s) will be at liberty to review the WB-MRI data after the WB-MRI has been revealed in order to review its findings and feedback to the clinical care team.

The completed MDT CRF will thus list:

- Stage and treatment decision based on conventional investigations (and the number, timing nature and findings of these investigations).
- Stage and theoretical treatment decision based on WB-MRI (and the number, timing nature and findings of additional tests generated).
- Final treatment decision incorporating all available tests.

Once the WB-MRI images and report have been revealed to the MDT according to trial protocol, they can be released onto the relevant hospital PACS server using the 3Dnet™ gateway described above or other IT solution (e.g. image exchange portal) and be made freely available to those providing subsequent patient care, as per usual clinical practice.

For peer review only

For peer review only - http://bmjopen.bmj.com/site/about/guidelines.xhtml

8.2.5. Early release of WB-MRI findings

Any requests for early WB-MRI release must be made to the site PI, who will discuss the request with the lead clinician who is responsible for the patient's clinical care (if the request did not originate from the lead clinician). After discussion if it is decided by the PI, that release of the WB-MRI images and report is necessary, the PI (or a designated member of the trial team) will inform UCL CTC. The WB-MRI result should only be released early in the event of an emergency situation in which the patient is unfit to undergo additional investigations (including MRI) which would have normally been performed were the patient not in the Streamline C trial, and the WB-MRI may have a direct effect on immediate patient care. In the event of early release, the patient will be replaced, although the findings of the WB-MRI and conventional staging (performed up to the time of early MRI release) will be collated by UCL CTC on the standard trial CRFs for subsequent reporting in the trial publications.

Early release for important clinical findings

The time between the patient's diagnosis and the WB-MRI reveal is typically less than 4 weeks at the proposed recruitment sites. The majority of findings on WB-MRI will have no direct impact on patient care during this time. However should WB-MRI reveal a serious finding which could have an immediate impact on direct patient care before the MDT, the reporting radiologist will contact the patient's clinician to discuss the finding and a decision will be made as to whether the results should be revealed early to all members of the clinical team (based on review of standard tests already performed which may also have detected the finding). Specific findings which will trigger this review are:

- Impending spinal cord compression
- Deep vein thrombosis or pulmonary embolism
- Brain metastasis with significant mass effect requiring immediate treatment

It is unlikely the patient would be asymptomatic in any of these scenarios, and it is probable additional imaging tests would have already been requested as part of usual clinical care.

Early release for urgent patient management

Although unlikely, it is possible there may be a need to access the WB-MRI report urgently before its release to the MDT, for example if a recruited patient presents to hospital with collapse and knowledge of the WB-MRI findings would potentially change patient management. As noted above, imaging hubs will keep a copy of the free text report of the WB-MRI and this will be made available if requested by clinical teams in this scenario.

8.2.6. Time to full staging

Standard pathway

The total time required to fully stage the patient using standard imaging pathways will be calculated. The start of the staging process will be defined as the date of request of the first staging investigation following a proven or assumed diagnosis of colorectal

For peer review only - http://bmjopen.bmj.com/site/about/guidelines.xhtml

cancer (for example date of requested for CT chest, abdomen and pelvis after colonoscopic diagnosis of likely malignant tumour). The completion of staging will be defined as the date of the MDT/appropriate MDT personnel made the first major treatment decision based on the standard imaging.

WB-MRI

Because revelation of the result of WB-MRI is deferred until after standard staging is complete, actual measurement of the time to full staging using WB-MRI will not be possible. The theoretical time to complete staging using WB-MRI will be thus modelled taking into consideration the time from recruitment date to the date of the WB-MRI, plus the number and type of additional staging investigations generated by WB-MRI.

8.2.7. Patient follow up

Patients will be followed for a period of 12 months from the date of recruitment. Follow up will not require any trial specific patient visits to the recruiting site, and follow up CRFs can be completed using hospital databases or patient notes.

For all recruited patients the final TN tumour stage, according to the 7th classification, will be recorded based on histopathological analysis of surgical specimens (if surgery is performed). The results of any biopsy procedure undertaken over the 12 months will also be recorded. The date, nature and findings of follow up imaging investigations will be recorded for each recruited patient, in particular the presence or absence of metastasis.

The date and cause of patient death and post mortem findings (if performed) will also be recorded.

MDT records and hospital data repositories will be used to collate this data, and data collection will be coordinated by designated individuals at each recruitment site, aided by UCL CTC.

8.2.8. Patient diaries and EQ-5D Quality of Life questionnaires

All patients will receive a Diary and EQ-5D Quality of Life questionnaire every 3 months which will be posted by UCL CTC. Upon consent the recruiting site must inform the patient that they will receive an EQ-5D questionnaire and Diary through the post from UCL CTC and they are to complete these and return them using the pre-paid envelope provided. The patient diary will cover a 3 month period and will collect information regarding the patient's Primary Health Care contacts, other health care contacts and also the medication they are currently taking. Patient diaries and EQ-5D questionnaires will be posted to all patients at consent and then at 3 monthly intervals for 12 months.

8.2.9. Final reference standard for tumour stage (Consensus Meetings)

Multi-disciplinary consensus panel review is standard methodology for diagnostic test accuracy studies where an independent reference standard is impossible because of incorporation bias. Consensus panels will convene annually to derive the reference standard for tumour stage at diagnosis for recruited patients completing the first 12 month follow up (i.e. a panel at the end of year 2 will consider patients recruited in

For peer review only - http://bmjopen.bmj.com/site/about/guidelines.xhtml

year 1, a panel at the end of year 3 will consider patient recruited in year 2 etc.). The panels will consider all available clinical information including the results of all original staging investigations, WB-MRI, histopathology (surgical resection and biopsies), follow up imaging and post-mortem reports (where available) and MDT records. UCL CTC will coordinate collation of these data via submitted CRFs over the preceding 12 months for presentation to the panel. Each imaging hub or recruitment site will host a consensus panel to consider patients undergoing WB-MRI (or recruited) at its site (so all imaging studies are available for review on local PACS systems if required). Each panel will consist of at least an oncologist, and/or a colorectal surgeon, and 3 radiologists, 1 external to the imaging hub and 2 internal: 1 with specific expertise in WB-MRI and 1 with expertise in PET (a single radiologist is acceptable if they have expertise in WB-MRI and PET). The panel will have access to a histopathologist if required. Each panel will adjudicate on the TMN stage of the cancer at diagnosis, including the organ specific sites and burden of metastatic spread.

The definition of the presence or absence of metastasis will be assigned for each organ on a designated CRF. The designation will be made in consensus by all on the panel. Consideration will be made to histology in all biopsied lesions. In the absence of histological proof of metastasis, metastatic disease will be assumed if new lesions appear during the 12 month follow up with imaging characteristics compatible with metastasis and no alternative explanation, or if lesions with characteristics compatible with metastasis which either grow or shrink (on therapy). Lesions identified which remain stable over the 12 month follow up period will assumed not to be metastatic unless there are specific circumstances considered by the panel that indicate malignancy (e.g. change in lesion morphology with treatment).

All “new” sites of metastatic disease diagnosed in the 12 month follow up will be assessed by the panel to see if they were visible in retrospect at diagnosis. Although the primary analysis by the original reporting radiologist will be that used to define test accuracy, this retrospective review will allow definition of the rates of perceptual error.

For patients in whom the primary tumour has been completely removed within 3 months of diagnosis, all new metastatic sites will be assumed to have been present at diagnosis for the purposes of calculating test sensitivity.

If the primary tumour is left in situ for more than 3 months (or there is incomplete removal), any new diagnosed sites of metastatic disease will be assumed to have been present at diagnosis if identified within 6 months of diagnosis of the primary tumour. If they are diagnosed beyond 6 months of diagnosis, and there is no evidence of their presence on retrospective review of all staging investigations, they will be assumed to be new disease not present at diagnosis.

If patients with tumours left in situ do not undergo any imaging capable of detecting metastatic disease within 6 months of diagnosis of the primary tumour (other than initial staging tests) and imaging beyond this identifies metastatic disease not visible in retrospect on any trial imaging, the consensus panel will opine if the disease was likely present at diagnosis, based on its location, size and imaging characteristics.

If a patient dies before the 12 month follow up, the panel will review all available imaging, histopathology and clinical course prior to death and in consensus state if a confident diagnosis of the presence or absence of metastatic disease can be made (for example imaging characteristics compatible with metastasis and no alternative

For peer review only - http://bmjopen.bmj.com/site/about/guidelines.xhtml

explanation, or if lesions with characteristics compatible with metastasis which either grow or shrink (on therapy). If this judgement cannot be made with confidence (for example if the patient has equivocal lesions on staging investigations and no further follow up), these patients will not be excluded but multiple imputation will be used to account for missing data. A sensitivity analysis for the primary outcome, where only patients with complete data are analysed, will exclude these patients.

8.2.10. Health psychology assessment

Study 1: Patient interviews (approximate n=25)

Target accrual has now been reached for the patient experience interview part of the trial and so recruitment to this part is now closed. Individual in-depth interviews were conducted by a health psychologist pertaining to their experience of the cancer staging process. The aim of the interviews is to determine patients' experience of WB-MRI, standard tests and those tests generated by WB-MRI. We collected simple demographic data from participants in relation to gender, age and educational level as such factors may influence experience and preferences, and this data will enable us to determine whether the sample of people who are interviewed are representative of the sample of people who also participate in studies 2 and 3 (see below). The interviews assessed which aspects of testing caused patients physical or psychological stress (e.g. number of tests/hospital visits, test attributes (i.e. physical experiences of the tests such as claustrophobia, need to lie still, scanner noise etc.), total length of time taken, additional tests generated by initial scan findings) and elicited any factors patients felt would have made staging easier for them.

The interviews provided in-depth qualitative data about the experience of cancer staging and in particular under-going WB-MRI, and has informed the modification of a questionnaire (study 2) designed to assess patient acceptability and experience of WB-MRI compared to standard tests, and also has informed the design of a discrete choice experiment (study 3).

Study 2: Patient questionnaires (approximate n=50)

Target accrual has now been reached for the patient experience questionnaire part of the trial and so recruitment to this part is now closed. Follow up questionnaires will continue to be posted until the last patient's month 6 time point occurs. A questionnaire was developed and sent to consenting patients to assess their experience and acceptability of WB-MRI and standard tests (e.g. PET-CT). Final design was informed by analysis of the qualitative data collected in study 1 (See above). Patient Experience Questionnaire content is outlined in Table 3 and includes patients' views about test preparation (e.g. fasting), and experience of the scan itself (e.g. discomfort, claustrophobia, fear etc.). Patients were also asked to rate the importance of various attributes associated with competing staging pathways (e.g. waiting time from diagnosis to treatment, total test number, test accuracy etc.). This rating identified those attributes considered most important by patients, which has been included in the discrete choice experiment (study 3).

Patients were given the opportunity to opt in to completing the questionnaires when they were recruited into the trial. Questionnaires, together with the positive and negative affect schedule (PANAS) and the General Health Questionnaire (GHQ-12) were administered during and after staging pathways, and at 3 and 6 months later to

For peer review only - http://bmjopen.bmj.com/site/about/guidelines.xhtml

examine patient test preferences once they are aware of their cancer stage and knew which of the tests they received were more accurate as well as the burden placed on them by any extra tests they needed as a result of WB-MRI.

Patients were posted the baseline questionnaire along with pre-paid reply envelope at the time they were recruited into the trial. The questionnaire was worded to take into account the fact that some patients had not yet had any staging investigations. Patients were posted the post-staging and 3 and 6 month follow up questionnaires along with pre-paid envelopes at the relevant time points.

Study 3: Discrete choice experiment (approximate n=50)

Discrete choice experiments (DCE) can elicit preferences for different types of health care provision [18] by estimating the relative importance of different attributes, and the trade-offs between them [19]. WB-MRI and conventional staging pathways differ in associated attributes, not only related to physical experience but also rate of adverse events, time to diagnosis, overall accuracy etc. The most important attributes have been identified in studies 1 and 2 and appropriate levels assigned to each based on accumulating data from the trial, together with appropriate literature review. A DCE questionnaire has been developed whereby patients state their preference between two choices, with each choice containing different levels of the identified attributes.

The DCE questionnaire is optional and patients will be asked to consent to complete the questionnaire on trial entry. Once a patient consents to the DCE they will be allocated a DCE questionnaire (out of a possible 2 DCE questionnaires) which will be posted by UCL CTC immediately after registration (baseline) along with their QoL questionnaire, patient diary and pre-paid envelope. UCL CTC will supply the details of any recruited patients which consented to complete the DCE questionnaire to the Health psychologists who may contact them directly.

For peer review only - http://bmjopen.bmj.com/site/about/guidelines.xhtml

Streamline C

Table 3: Questionnaire schedule. Questionnaires will be posted to patients by UCL CTC.

	Baseline (peri-staging)	Months after registration		
		1	3	6
Demographic measures	✓			
Co-morbidities	✓			
Self-rated health	✓			
Symptoms of bowel cancer	✓			
Concern for future consequences	✓			
Which staging tests have had	✓	✓		
Perceived importance of test attributes	✓	✓		
Perceived acceptability of different tests	✓	✓		
Least acceptable aspects of different tests	✓	✓		
Agreement to having tests again (short and long-term)	✓	✓		
Single test choice	✓	✓		
Test recovery time	✓	✓		
Patient experience of WB-MRI and standard tests	✓	✓		
Perceived efficacy of WB-MRI and standard tests		✓		
Difficulties with completing scan (WB-MRI and standard tests)		✓		
Ranked attribute preferences	✓	✓		
Feelings about number of tests			✓	✓
EQ-5D	✓		✓	✓
PANAS	✓	✓	✓	✓
GHQ-12	✓	✓	✓	✓

For peer review only

For peer review only - http://bmjopen.bmj.com/site/about/guidelines.xhtml

8.2.11. Cost Effectiveness Assessment

Overview

We will undertake a detailed analysis of the cost and the cost-effectiveness of WB-MRI versus standard NICE-approved staging algorithms. The analysis will conform to accepted economic evaluation methods [17]. All costs will be assessed from the perspective of the NHS and personal social services (PSS).

Making comparisons between WB-MRI versus standard staging algorithms

The care pathway for colorectal cancer patients can both be divided into two stages, the *treatment decision pathway* and the *subsequent disease pathway*. The former includes the time from diagnosis to treatment decision by the MDT; the latter includes the time period following the treatment decision.

The treatment decision pathway will be different between WB-MRI and standard staging algorithms, yielding different costs and potentially different treatment decisions. In patients for whom the treatment decision with WB-MRI is the same as that with conventional staging algorithms, the subsequent disease pathways will be the same. Where the treatment decision with WB-MRI is different, the disease pathway will be different, yielding potentially different costs and health outcomes.

If in the patients studied the concordance between the treatment decisions associated with WB-MRI and conventional staging algorithms is high, then the economic analysis can focus on the cost of the treatment decision pathways only because the disease pathways will be no different. In this case the cost-effectiveness of WB-MRI versus conventional staging algorithms depends only on the incremental cost (positive or negative) of WB-MRI versus standard staging algorithms in the treatment decision pathway.

Conversely, if the concordance between the treatment decisions is low, then the economic analysis ought to focus on both the treatment decision pathways and the subsequent disease pathways because both of these will vary between WB-MRI and conventional staging algorithms. In this case the cost-effectiveness of WB-MRI depends on the incremental cost of the WB-MRI versus standard staging algorithms in the treatment decision pathway plus the incremental costs and health benefits of the disease pathway.

The precise nature of the economic analysis will therefore depend on the degree of concordance between treatment decisions provoked by WB-MRI versus conventional staging.

Discordance of major treatment decisions

Discordance will be defined when the first major treatment decision differs between WB-MRI and standard investigations in greater than 10% of patients. Concordance will be defined as the absence of discordance. For colorectal patients a 4x4 table will be used to summarise major differences between 4 main treatment decisions (surgery for the primary but no chemotherapy, surgery for the primary and chemotherapy (and/or radiotherapy), chemotherapy (and/or radiotherapy) without surgery, and surgical metastectomy with or without chemotherapy) for colorectal cancer).

For peer review only - http://bmjopen.bmj.com/site/about/guidelines.xhtml

Streamline C

Scenario 1: there is concordance between the treatment decisions associated with WB-MRI and standard staging

In this case, the cost components included in the analysis will be:

- Conventional tests for staging of disease among diagnosed patients (e.g., CT chest, pelvis and abdomen and MRI pelvis for colon cancer; plus additional tests as indicated by the conventional staging algorithm);
- Costs of treating adverse events associated with staging tests;
- WB-MRI, plus additional tests generated by WB-MRI;
- MDT meetings.

The volume of resource use for each cost component will be measured directly in the trial from treatment decisions recorded in MDT reports, based first on conventional staging alone and then based on WB-MRI alone, and on patient records included in the trial. Unit costs will be taken from standard published sources. Since the two algorithms yield the same treatment decisions cost-effectiveness depends on the incremental cost of WB-MRI versus conventional staging algorithms in the treatment decision pathway (i.e., formally this is a cost-minimisation analysis).

Scenario 2: there is discordance between the treatment decisions associated with WB-MRI and standard staging

In this case, cost-effectiveness depends on the incremental cost (positive or negative) of the treatment decision pathway and disease pathway associated with WB-MRI versus conventional staging and the incremental health benefits (positive or negative). We will calculate cost-effectiveness in terms of the incremental cost per quality-adjusted life year (QALY) gained using one year and lifetime time horizons.

For the analysis based on the one-year time horizon, the trial will provide information on the treatment decisions arising from WB-MRI and standard staging and follow up data for the first year. We will collect these data from three sources.

Firstly, for all patients in the trial we will collect the resource use data for the main drivers of hospital costs using a trial specific CRF. This will collect resource use data on the following cost components for each patient:

- Imaging investigations
- Chemotherapy
- Radiotherapy
- Surgery and biopsies
- Outpatient visits
- Inpatient stays
- Day cases

These data will be recorded prospectively based mainly on MDT consensus meeting recommendations and on patient records (for major changes in treatment). Unit costs will be taken from standard published sources and applied to the resource use data, allowing us to cost the care received by each patient.

In addition, from this source we will collect data on whether or not the patient died during the follow up period, and if they died, the date of death.

For peer review only - http://bmjopen.bmj.com/site/about/guidelines.xhtml

Secondly, for all patients in the trial we will prospectively collect resource use data using patient diaries. This will supplement the above data which may not provide a complete picture of hospital resource use, plus it will allow us to collect data on primary and community care contacts. These will record resource use data on the following cost components for each patient:

- Medications taken
- Day cases
- GP contacts
- Practice and community nurse contacts
- A&E Visits
- Any other primary care or community care contacts related to cancer

The diaries will be posted out by UCL CTC at baseline, 3, 6, and 9 months and patients will be asked to complete them for the following 3 month period.

In addition we will also collect data on health-related quality of life score, measured according to the EQ-5D (www.euroqol.org), which will be measured at baseline and at 3, 6, 9 and 12 months for all surviving patients.

UCL CTC will contact sites just prior to sending out any post registration diaries or questionnaires to check the status of the patient to ensure none are sent inappropriately to deceased patients.

Patients who are found not to have cancer or are withdrawn for other reasons (see section 13), will not be sent diaries, quality of life or patient experience questionnaires to complete. Thirdly, the two sources of resource use data described above will be supplemented by retrospective review of resource use (including imaging, outpatient visits, inpatient stays and medication use) for the 12 months post diagnosis of up to 150 patients diagnosed with colorectal cancer at UCLH. This retrospective review will be carried out by a member of the clinical team and according to guidance provided by the UCLH Caldicott guardian. All collated retrospective data will be fully anonymised before sharing with other trial researchers outside the clinical care team.

8.2.12. Nested substudies

1) Diagnostic performance of limited WB-MRI protocols based on diffusion and T1 weighted imaging only, and to assess the incremental benefit of intravenous gadolinium contrast enhancement.

   As noted above, WB-MRI will be analysed using a locked sequential viewing paradigm such that the diagnostic accuracy of Diffusion and non contrast enhanced T1 images, Diffusion, non contrast enhanced T1 and T2 images, and Diffusion, non contrast enhanced T1, T2 and contrast enhanced T1 images can be calculated.

2) Effect of radiologists' diagnostic confidence on the accuracy of WB-MRI.

   Radiologists will indicate their level of confidence for the presence or absence of metastasis on a 1-6 scale on the CRF recording their interpretation of the WB-MRI.

3) Inter-observer variation in WB-MRI interpretation.

For peer review only - http://bmjopen.bmj.com/site/about/guidelines.xhtml

Streamline C

Each hub radiologist (n=6) will interpret a sample of 25 WB-MRI datasets selected at random from the other imaging hubs to define inter-observer variation in the reported presence absence of metastatic disease.

4) The collated WB-MRI datasets and 12 month patient follow up data collated as part of the trial protocol provides opportunities to retrospectively mine the data for additional diagnostic indices and prognostic markers (See appendix 4). The following or similar may be quantified in all or a subset of whole body MRI datasets:

- Body fat (organ, visceral and whole body),
- MRI signal from the primary tumour and metastatic sites, notably diffusion weighted signal, apparent diffusion coefficient, T1 (including fat and water components), T2 signal, Contrast enhancement, and textural analysis (for heterogeneity) of the primary and metastatic deposits on the various sequences.

For peer review only

For peer review only - http://bmjopen.bmj.com/site/about/guidelines.xhtml

9. Data Management Guidelines

Data will be collected from sites on version controlled case report forms (CRFs) designed for the trial and supplied by UCL CTC. Data entered onto CRFs must reflect source data at site. Some data will be recorded directly on the CRFs (i.e. no prior written or electronic record of data), and it will be considered to be source data. This will be made clear in the CRF guidance.

Where supporting documentation (e.g. autopsy reports, pathology reports, CT scan images etc.) is being submitted to UCL CTC, the patient's trial number must be clearly indicated on all material and any patient identifiers removed/blacked out prior to sending to maintain confidentiality.

9.1. Completing Case Report Forms

All CRFs must be completed and signed by staff who are listed on the site staff delegation log and authorised by the PI to perform this duty. The PI is responsible for the accuracy of all data reported in the CRF.

All entries must be clear, legible and written in ball point pen. The use of abbreviations and acronyms must be avoided.

Any corrections made to a CRF at site must be made by drawing a single line through the incorrect item ensuring that the previous entry is not obscured. Each correction must be dated and initialled. Correction fluid must not be used.

The use of abbreviations and acronyms must be avoided.

Once completed the original CRFs must be sent to UCL CTC and a copy kept at site.

9.2. Missing Data

To avoid the need for unnecessary data queries CRFs must be checked at site to ensure there are no blank fields before sending to UCL CTC. When data are unavailable because a measure has not been taken or test not performed, enter "ND" for not done. If an item was not required at the particular time the form relates to, enter "NA" for not applicable. When data are unknown enter the value "NK" (only use if every effort has been made to obtain the data).

9.3. Timelines for data return

CRFs must be completed at site and returned to UCL CTC as soon as possible and within 4 weeks of scheduled time point. For further details, see CRF instructions.

Sites who persistently do not return data within the required timelines may be suspended from recruiting further patients into the trial by UCL CTC and subjected to a 'for cause' monitoring visit. See section 12.2 ('For cause' on-site monitoring) for details.

For peer review only - http://bmjopen.bmj.com/site/about/guidelines.xhtml

9.4. Data Queries

Data arriving at UCL CTC will be checked for legibility, completeness, accuracy and consistency, including checks for missing or unusual values. Query reports will be sent to the data contact at site. Further guidance on how data contacts should respond to Data Queries can be found on the Query Reports.

For peer review only

For peer review only - http://bmjopen.bmj.com/site/about/guidelines.xhtml

10. Safety Reporting

Clinical Review

The MRI Imaging Hub and/or Recruiting site will record any complications attributable to the WB-MRI or any additional test performed as a result of the WB-MRI, notably contrast reactions, biopsy complications (infection, bleeding or hospital admission), and treatment target breaches on the Staging Complications CRF which will be submitted to UCL CTC.

UCL CTC will provide trial data to the CI on a periodic basis for review. If it is declared necessary to revise the conduct of the trial due to safety concerns, UCL CTC will inform the REC as appropriate.

For peer review only

For peer review only - http://bmjopen.bmj.com/site/about/guidelines.xhtml

11. Incident Reporting

Organisations must notify UCL CTC of all deviations from the protocol or GCP immediately. UCL CTC may require a report on the incident(s) and a form will be provided if the organisation does not have an appropriate document (e.g. Trust Incident Form).

If site staff are unsure whether a certain occurrence constitutes a deviation from the protocol or GCP, the UCL CTC trial team can be contacted immediately to discuss.

UCL CTC will use an organisation's history of non-compliance to make decisions on future collaborations.

For peer review only

For peer review only - http://bmjopen.bmj.com/site/about/guidelines.xhtml

12. Trial Monitoring and Oversight

Participating sites and PIs must agree to allow trial-related on site monitoring, Sponsor audits and regulatory inspections by providing direct access to source data/documents as required. Patients are informed of this in the patient information sheet and are asked to consent to their medical notes being reviewed by appropriate individuals on the consent form.

UCL CTC will determine the appropriate level and nature of monitoring required for the trial. Risk will be assessed on an ongoing basis and adjustments made accordingly.

12.1. Central Monitoring

Sites will be requested to submit relevant logs to UCL CTC at the frequency detailed in the trial monitoring plan or on request and these will be checked for consistency and completeness. Also refer to sections 4.2.2 (Required Documentation) and 6.2 (Screening Logs).

Ensuring patient eligibility is the responsibility of the PI or other delegated Investigator(s). Checks of the criteria listed on the registration form will be undertaken by an appropriately trained UCL CTC staff member prior to registration. Also refer to section 7.1 (Registration).

Details relating to the informed consent process will be collected on the registration form and are subject to review by UCL CTC as part of patient eligibility.

Data received at UCL CTC will be subject to review in accordance with section 9.4 (Data Queries).

Where central monitoring of data and/or documentation submitted by sites indicates that a patient may have been placed at risk, the matter will be raised urgently with site staff and escalated as appropriate (refer to section 11 (Incident Reporting) and 12.3 (‘For cause’ on site monitoring) for further details).

12.2. ’For Cause’ On-Site Monitoring

On site monitoring visits may be scheduled at a site where there is evidence or suspicion of non-compliance with important aspect(s) of the trial protocol/GCP requirements. Sites will be sent a letter in advance outlining the reason(s) for the visit. The letter will include a list of the documents that are to be reviewed, interviews that will be conducted, planned inspections of the facilities, who will be performing the visit and when the visit is likely to occur.

Following a monitoring visit, the Trial Monitor/Trial Coordinator will provide a report to the site, which will summarise the documents reviewed and a statement of findings, deviations, deficiencies, conclusions, actions taken and actions required. The PI at each site will be responsible for ensuring that monitoring findings are addressed in a timely manner, and by the deadline specified.

For peer review only - http://bmjopen.bmj.com/site/about/guidelines.xhtml

UCL CTC will assess whether it is appropriate for the site to continue participation in the trial. Refer to section 11 (Incident Reporting) for details.

12.3. Oversight Committees

12.3.1. Trial Management Group (TMG)

The TMG will include the Chief Investigator, clinicians and experts from relevant specialities and Streamline C trial staff from UCL CTC (see page 3). The TMG will be responsible for overseeing the trial. The group will meet regularly at least once a year and will send updates to PIs (via newsletters or at Investigator meetings) and to the NCRI Colorectal Clinical Studies Group.

The TMG will review substantial amendments to the protocol prior to submission to the REC. All PIs will be kept informed of substantial amendments through their nominated responsible individuals.

TMG members must sign a charter confirming acceptance of their responsibilities.

12.3.2. Trial Steering Committee (TSC)

The role of the TSC is to provide overall supervision of the trial. The TSC will review the recommendations of the Independent Data Monitoring Committee and, on consideration of this information, recommend any appropriate amendments/actions for the trial as necessary. The TSC acts on behalf of the funder and Sponsor.

TSC members must sign a charter confirming acceptance of their responsibilities and declaring any potential conflicts of interest.

12.3.3. Independent Data Monitoring Committee (IDMC)

The role of the IDMC is to provide independent advice on data and safety aspects of the trial. Meetings of the Committee will be held at least once a year or as necessary to address any issues. The IDMC is advisory to the TSC and can recommend premature closure of the trial to the TSC.

IDMC members must sign a charter confirming acceptance of their responsibilities and declaring any potential conflicts of interest.

12.3.4. Role of UCL CTC

UCL CTC will be responsible for the day to day coordination and management of the trial and will act as custodian of the data generated in the trial (on behalf of UCL). UCL CTC is responsible for all duties relating to safety reporting which are conducted in accordance with section 10 (Safety Reporting).

For peer review only

For peer review only - http://bmjopen.bmj.com/site/about/guidelines.xhtml

13. Withdrawal of Patients

In consenting to the trial, patients are consenting to the WB-MRI, and also specifically consenting to completing questionnaires and diaries.

Losses to follow up

If a patient moves from the area, the site should make every effort to ensure the patient is followed up at another participating trial site and for this new site to take over the responsibility for the patient, or for follow up via GP. Details of participating trial sites can be obtained from the UCL CTC trial team who must be informed of the transfer of care and follow up arrangements.

Patients should be considered lost to follow up only once documented efforts on the part of the site have failed to produce any response or information from the patient or GP over the course of one year.

If a patient is lost to follow up at a site every effort should be made to contact the patient's GP to obtain information on the patient's status. If the patient cannot be contacted this should be recorded on the Change of Status CRF.

Patient Withdrawing Consent

If a patient expresses their wish to withdraw from the trial, sites should explain the importance of allowing routine follow up data to be used for trial purposes and for allowing existing collected data to be used. If the patient gives a reason for their withdrawal, this should be recorded (see below).

Withdrawal of Consent to Future Data Collection and use of past data

If a patient explicitly states they do not wish to contribute further data to the trial or allow the use of past data, their decision must be respected and recorded on the Change of Status CRF. In this event details should be recorded in the patient's hospital records, no further CRFs must be completed and no further data sent to UCL CTC.

Withdrawal of Consent to QoL, Resource use diary and/or Patient experience questionnaires

If a patient withdraws consent from completing the QoL, Resource use diary and/or the Patient experience questionnaires their decision must be recorded on the Change of Status CRF. UCL CTC will not post any further documents to the patient. However, the patient may continue to participate in the trial, and trial follow up completed as per protocol.

For peer review only - http://bmjopen.bmj.com/site/about/guidelines.xhtml

14. Trial Closure

14.1. End of Trial

For regulatory purposes the end of the trial will be 1 year after recruitment of the final patient at which point the 'declaration of end of trial' form will be submitted to ethical committees, as required.

Following this, UCL CTC will advise sites on the procedure for closing the trial at the site.

14.2. Archiving of Trial Documentation

At the end of the trial, UCL CTC will archive securely all centrally held trial related documentation for a minimum of 5 years. Arrangements for confidential destruction will then be made. It is the responsibility of PIs to ensure data and all essential documents relating to the trial held at site are retained for a minimum of 5 years after the end of the trial, in accordance with national legislation and for the maximum period of time permitted by the site.

Essential documents are those which enable both the conduct of the trial and the quality of the data produced to be evaluated and show whether the site complied with the principles of GCP and all applicable regulatory requirements.

UCL CTC will notify sites when trial documentation held at sites may be archived. All archived documents must continue to be available for inspection by appropriate authorities upon request.

Health Psychology data collected as part of this research will be retained for 10 years in line with Birkbeck, University of London's records management policy. At the end of this time period the documents will be shredded.

14.3. Early discontinuation of trial

The trial may be stopped before completion as an Urgent Safety Measure on the recommendation of the TSC or IDMC (see section 12.3.2 TSC and 12.3.3 IDMC). Sites will be informed in writing by UCL CTC of reasons for early closure and the actions to be taken with regards the treatment and follow up of patients.

14.4. Withdrawal from trial participation by a site

Should a site choose to close to recruitment the PI must inform UCL CTC in writing. Follow up as per protocol must continue for all patients recruited into the trial at that site and other responsibilities continue as per Clinical Trial Site Agreement (CTSA).

For peer review only - http://bmjopen.bmj.com/site/about/guidelines.xhtml

15. Quality Assurance

The technical quality of at least 10% of the WB-MRI from each imaging hub will be assessed by the trial radiographer at UCLH. Using the 3Dnet™ software, the radiographer will access performed scans on a weekly basis and enter an audit score of quality according to the definitions below. A report of the quality will be provided to the CI every 2 weeks and to the TMG at each meeting

Technical quality - general

1 = More than one sequence with substantial degradation of images severely limiting interpretation of those sequences, and not repeated

2 = One sequence with substantial degradation of images severely limiting interpretation of that sequence, and not repeated

3 = More than one sequence has minor artefact, but all remain fully diagnostic and repeat although optimal, not necessary OR all sequences initially technically inadequate (score 1 or 2) correctly repeated

4 = One sequence a has minor artefact, but remains fully diagnostic and repeat, although optimal, not necessary

5 = All sequences technically optimal with no artefact or degradation

Technical quality - anatomical coverage

1 = Wrong examination performed

2 = More than one sequence does not adequately cover the body (skull to mid thigh), or designated organ(s) coverage

3 = One sequence does not optimally cover the body or designated organ(s) coverage but examination remains fully diagnostic

4 = All sequences optimally cover the body and designated organ(s) coverage

For peer review only

For peer review only - http://bmjopen.bmj.com/site/about/guidelines.xhtml

Streamline C

16. Statistics

16.1. Sample size calculation

SAMPLE SIZE: Studies of diagnostic test accuracy are based on a cohort design where all patients receive all main diagnostic tests (CT or WB-MRI), although additional tests ordered in each staging pathway differ between patients, based on clinical appropriateness. Existing pathways and therapeutic decision making is complex for both cancers (see flow charts). This NIHR HTA trial focuses on first definitive staging and we are powered at this point in the patient pathway, informed by a detailed literature review.

Multiple therapeutic options are contingent on diagnosis of metastatic disease.

To power on any one of these (e.g. liver resection rates) would be beyond pragmatic recruitment and available funding.

We therefore power on a change in sensitivity for metastases detection with a WB-MRI pathway as a replacement to the standard NICE staging pathway.

Sample size calculation

- Paired trial design – comparing two diagnostic pathways where both are used in all patients.
- Sample size method for difference in paired proportions [22].
- Power trial to show difference in sensitivity between two diagnostic pathways, as fewer patients are with metastasis than without. Trials powered like this should also be suitable for difference in specificity.
- 80% power type II error, type I error 5% ($p<0.05$)
- Ratio of marginal cells in 2 x 2 table: s/t=7 (table 4)
- Sum of marginal cells in 2 x 2 table: s+t/N = 0.14
- A sample size of 116 patients with metastasis is needed, from a population of 40% prevalence of metastasis. Thus a total sample size of 290 (116 times 2.5) is required.

Table 4: Calculations comparing WB-MRI to PET

		MDT results for standard tests: patients with metastases detected		
	Result	+	-	Total
MDT results for WB-MRI plus additional tests: patients with metastases detected	+	85	14	99
	-	2	15	17
	Total	87	29	116

For peer review only - http://bmjopen.bmj.com/site/about/guidelines.xhtml

290 patients would be required to detect a difference of 10% in sensitivity of WB-MRI for metastatic disease (85%) compared to conventional staging (75%) given the disease prevalence as described above.

The sample size above must be adjusted to account for “withdrawals”. Patients are classified as withdrawn if they are recruited but are not evaluable for any of the following reasons (i) they do not have an WB-MRI scan, the main component of the primary outcome (ii) they do not have cancer (iii) they are lost to follow up before one year and do not have sufficient information for the expert panel to evaluate whether metastases were present.

The withdrawal rate was expected to be 10%, giving a target recruitment of 290 patients. However, the actual withdrawal rate has been 19% and the target recruitment has therefore been revised to 360 patients.

16.2. Population for analysis

The analysis population will be all patients, equivalent to the RCT population intention to treat (ITT) population, with multiple imputation used to impute missing data. For the primary outcome, complete case analysis (all available patient data with no imputation) will be reported alongside as a sensitivity analysis to the ITT analysis.

16.3. Analysis of the primary endpoint

Primary Outcome- Per patient sensitivity for metastasis detection by whole body MRI (WB-MRI) compared to standard staging pathways in newly diagnosed colorectal cancer against an expert derived consensus reference standard.

The primary objective is to evaluate whether early whole body magnetic resonance Imaging (WB-MRI) increases per patient sensitivity for metastasis in colorectal cancer compared to standard NICE-approved diagnostic pathways.

- Comparison to detect metastases between early WB-MRI alone and standard NICE diagnostic strategy.
- Difference in per patient sensitivity.
- Paired comparison of proportions of each strategy against the reference standard of full clinical diagnosis and 12 month follow up. [23]
- Imputation will be used to account for missing data and imperfect reference data. [24].

Definition of positive test result from standard tests (conventional imaging)

- Conventional imaging tests include CT and may include PET, USS, organ specific MRI, bone scans, lymph node sampling.
- Report from the MDT will express presence of metastatic disease for each patient categorised as yes, no and equivocal.

For peer review only - http://bmjopen.bmj.com/site/about/guidelines.xhtml

Streamline C

- Equivocal results will be grouped with positive test results as these results require additional follow up investigations compared to negative results.
- The radiology imaging report supplied to the MDT will be used for sensitivity analysis using free text report (possible and probable metastasis counted as positive).

Definition of positive test result from WB-MRI and additional tests requested after WB-MRI (WB-MRI as replacement test)

- WB-MRI imaging CRF report from the WB-MRI radiologist will express presence of metastatic disease for each patient categorised as yes, no and equivocal.
- Equivocal results will be grouped with positive test results as these results require additional follow up investigations compared to negative results.

Definition of positive test result from reference test: 12 month expert consensus panel

Expert panel results will be classified as positive or negative for metastasis by expert consensus. No equivocal category will be allowed.

Multiple imputation will be used for missing data using chained equations in STATA [25].

Sensitivity analysis: A sensitivity analysis will investigate the impact of the equivocal results on the detection rate by WB-MRI. Equivocal results will be grouped with negative test results [26].

A planned subgroup analysis will include: patients with rectal and non-rectal colorectal cancer.

16.4. Analysis of secondary endpoints

16.4.1. Efficacy (secondary)

Secondary outcome- The time and test number taken to reach, and the nature of, the first major treatment decision based on WB-MRI in comparison to standard staging pathways

The objective is to determine how WB-MRI influences time to and nature of first major treatment decision following definitive staging compared to standard investigations and to determine whether early WB-MRI could reduce or replace standard investigations.

In this secondary outcome, the following components will be measured up to the time of the first major treatment decision

- Nature of first major treatment decision and number of patients with changed management decision
- Time taken for diagnostic pathways
- Number of tests: average per patient, number of patients with fewer tests in pathway

These will be compared for WB-MRI pathway in comparison to standard staging pathways.

For peer review only - http://bmjopen.bmj.com/site/about/guidelines.xhtml

This information will be reported and discussed as a basis for whether early WB-MRI could replace or reduce standard investigations.

No absolute pre-specified definition of the combination of these outcomes likely to be considered clinically significant is attempted although it is noted that:

- An average decrease in diagnosis time of 7 days is likely to be considered clinically significant if accuracy were similar.

Secondary outcome- Diagnosis accuracy of WB-MRI and conventional staging pathways for local tumour staging and detection of metastasis in comparison to an expert derived consensus reference standard

The objective is to assess the accuracy of WB-MRI and standard diagnostic pathways for local and distant staging by comparison with an expert derived consensus reference standard using 1 year patient follow up data.

- All comparisons except difference in sensitivity per patient compare WB-MRI as both replacement and additional test compared to standard NICE staging pathway

The following outcomes will be assessed as secondary outcome #2:

- Difference in sensitivity per patient: WB-MRI as additional test compared to standard NICE staging pathway
- Difference in sensitivity and specificity per organ site. Count each site once per patient, regardless of the number of metastases per organ.
- Difference in sensitivity and specificity per metastasis.

Secondary outcome- Inter-observer variability in WB-MRI analysis and effect of diagnostic confidence on staging accuracy.

The objective is to determine the effect of radiologist confidence on the diagnostic accuracy of WB-MRI for metastatic disease.

A substudy will be conducted to look at inter-observer variability between radiologists interpreting WB-MRI images and the effect of diagnostic confidence on staging accuracy.

- Each hub radiologist (n=5) will interpret a sample of 25 WB-MRI datasets selected at random from the other imaging hubs to define inter-observer variation in the reported presence absence of metastatic disease.
- These images will be read after patient management decisions are taken and so will not affect patient diagnosis or treatment.

Secondary outcome – Diagnostic accuracy of limited T1 and diffusion weighted sequences compared to full multi-sequence WB-MRI protocols

The objective is to evaluate the diagnostic performance of limited WB-MRI protocols based on diffusion and T1 weighted imaging only, and to assess the incremental benefit of intravenous gadolinium contrast enhancement.

Comparison of diagnostic accuracy of WB-MRI alone (i) block 1 sequence (ii) block 2 (iii) block 3 sequences.

- Reference standard: WB-MRI plus standard tests with 12 month follow up.

For peer review only - http://bmjopen.bmj.com/site/about/guidelines.xhtml

- Methods and tables: as per primary outcome and secondary outcome #3.
- Analysed for units of analysis as (i) per patient (ii) per organ site (iii) per metastasis.

16.4.2. Safety

We will provide information on the following:

- Additional tests ordered as a result of WB-MRI but not ordered for the patient in the standard NICE staging pathway. Note this might include tests due to abnormalities outside colon.
- Extra days in hospital due to extra tests ordered through WB-MRI.

16.4.3. Economic evaluation

Patient-specific utility profiles will then be constructed assuming a straight line relation between each of the patients EQ-5D scores at each follow up point. The quality-adjusted life-years (QALYs) experienced by each patient up to one year will be calculated as the area underneath this profile.

We will calculate for each individual patient in the trial their costs and outcomes up to one year. We will also have data from the retrospective review of resource use in UCLH patients previously diagnosed with colorectal cancer.

Individual patients will then be grouped according to the specific disease path depending on the treatment decision (e.g., surgery for the primary but no chemotherapy, surgery for the primary and chemotherapy (and/or radiotherapy), chemotherapy (and/or radiotherapy) without surgery, and surgical metastectomy with or without chemotherapy for colorectal cancer) and the accuracy of the staging result. We will calculate the mean costs and QALYs for each group. Mean costs and QALYs for WB-MRI versus standard staging algorithms will then be calculated based on the proportion in each group using each algorithm, which will be different since in this scenario there is discordance in treatment decision.

Cost-effectiveness will be calculated as the mean cost difference between WB-MRI versus standard staging algorithms divided by the mean difference in outcomes (QALYs) to give the incremental cost-effectiveness ratio (ICER). Non-parametric methods for calculating confidence intervals around the ICER based on bootstrapped estimates of the mean cost and QALY differences will be used [27]. The bootstrap replications will also be used to construct a cost-effectiveness acceptability curve, which will show the probability that WB-MRI is cost-effective at one year for different values of the NHS' willingness to pay for an additional QALY. We will also subject the results to extensive deterministic (one-, two- and multi-way) sensitivity analysis.

For the analysis based on the lifetime time horizon we will use the 1 year data described above. To extrapolate beyond the end of the 1 year follow up period we will develop a de novo cost-effectiveness model for the disease pathway, which will be populated via available evidence. These will model patient movements between long-term health states. The models will be similar in design to a recent NIHR HTA-funded trial of PET and MRI for detection of metastasis in breast cancer [28]. Following decisions about model structure, a list of parameter estimates required for the model will be developed. The specific details of the data to be used to populate the model will

For peer review only - http://bmjopen.bmj.com/site/about/guidelines.xhtml
For peer review only

be determined following the development of the structure and the systematic searches of the literature to identify existing models.

The upshot of this analysis is that we will calculate for each individual patient in the trial their lifetime costs and QALYs. Individual patients will then be grouped according to the specific disease path and the accuracy of the staging result, as before, and we will calculate the mean costs and QALYs for each group. Mean costs and QALYs for WB-MRI versus standard staging algorithms will then be calculated based on the proportion in each group using each algorithm, again as before. We will undertake deterministic (one-, two- and multi-way) and probabilistic sensitivity analysis, the latter assuming appropriate distributions and parameter values [29].

16.4.4. Health psychology assessment

Study 1: Patient interviews (approximate n=25)

No formal statistical analysis will be carried out on the information from the patient interviews. The interviews will provide in-depth qualitative data about the experience of cancer staging and in particular under-going WB-MRI, and will inform the modification of a questionnaire (study 2) designed to assess patient acceptability and experience of WB-MRI compared to standard tests, and also inform the design of a discrete choice experiment (study 3).

Study 2: Patient questionnaires (approximate n=50)

Comparative patient experience between WB-MRI and standard staging investigations, identification of important staging pathway attributes, comparative anxiety, expectations and attribute importance before and following the staging process will be analysed using within subjects ANOVA or the Wilcoxon matched-pair sign-test depending on whether the data are normally distributed or not.

Study 3: Discrete choice experiment (approximate n=50)

Sample size should be greater than (500*c)/(t*a) where t = the number of sets of choices, a = the number of scenarios to choose between in each choice, and c = the largest number of levels for any one attribute [30]. Assuming each person will undertake 15 sets of choices, there are two scenarios in each choice, and the largest number of levels for any one attribute is 3 then the required sample size is (500*3)/(15*2) = 50. We will administer this questionnaire to approximately 50 colon patients after staging has been completed, sampling across more than one of the imaging hubs. The questionnaire will be posted to patients with a pre-paid reply envelope. A reminder letter will be sent if no questionnaire has been returned within approximately 4 weeks. Analysis will conform to the checklist for conjoint analysis applications in health developed by the International Society for Pharmaceuticals and Outcome Research (ISPOR). We will use a random effects probit model for statistical analysis, which is appropriate given the data structure. Outcomes: The average relative importance weighting of attributes ascribed to standard versus WB-MRI staging pathways by patients diagnosed with colonic cancer.

For peer review only - http://bmjopen.bmj.com/site/about/guidelines.xhtml

16.4.5. Handling missing data

Multiple imputation will be used for missing data using chained equations in STATA [25].

For the primary outcome, complete case analysis (all available patient data with no imputation) will be reported alongside as a sensitivity analysis to the ITT analysis.

16.5. Interim analyses

Interim safety data will be supplied to the IDMC on a periodic basis from UCL CTC on:

- Accumulating information relating to recruitment and data quality (e.g. data return rates).
- Safety data:
  - Additional tests performed as a result of the WB-MRI that would not have otherwise been performed as part of the patient's standard diagnostic pathway. Note this might include tests due to abnormalities outside colon or lung.
  - Potential harms to patients (e.g. staging complications resulting from MRI).
  - Whole Body MRIs that were not revealed or revealed too early/late.
- Percentage missing data for primary outcome.
- Prevalence of metastasis within recruited patients (key sample size assumption).

There will be no interim analysis based on the trial outcomes of diagnostic accuracy as all diagnostic test results are made available for the MDT meeting which decides first management treatment decision.

For peer review only

For peer review only - http://bmjopen.bmj.com/site/about/guidelines.xhtml

17. Ethical Approvals

In conducting the trial, the Sponsor, UCL CTC and sites shall comply with all laws and statutes, as amended from time to time, applicable to the performance of clinical trials including, but not limited to:

- the principles of ICH Harmonised Tripartite Guideline for Good Clinical Practice
- the Human Rights Act 1998
- the Data Protection Act 1998
- the Freedom of Information Act 2000
- the Research Governance Framework for Health and Social Care, issued by the UK Department of Health (Second Edition 2005) or the Scottish Health Department Research Governance Framework for Health and Community Care (Second Edition 2006)

17.1. Ethical Approval

The trial will be conducted in accordance with the World Medical Association Declaration of Helsinki entitled 'Ethical Principles for Medical Research Involving Human Subjects' (1996 version) and in accordance with the terms and conditions of the ethical approval given to the trial.

The trial has received a favourable opinion from the London – Camden & Islington Research Ethics Committee.

UCL CTC will submit Annual Progress Reports to the REC, which will commence one year from the date of ethical approval for the trial.

17.2. Site Approvals

The Lead Comprehensive Local Research Network (CLRN), Central and East London CLRN, has given NHS permission following global governance checks. Local governance checks will be undertaken by local CLRNs associated with individual trial sites.

Evidence of approval from the Trust R&D for a trial site must be provided to UCL CTC. Sites will only be activated when all necessary local approvals for the trial have been obtained.

17.3. Protocol Amendments

UCL CTC will be responsible for gaining ethical approval, for amendments made to the protocol and other trial-related documents. Once approved, UCL CTC will ensure that all amended documents are distributed to sites and CLRNs as appropriate.

Site staff will be responsible for acknowledging receipt of documents and for implementing all amendments.

For peer review only - http://bmjopen.bmj.com/site/about/guidelines.xhtml

17.4. Patient Confidentiality & Data Protection

Patient identifiable data, including full name, address, date of birth, gender and NHS number will be required for the registration process and will be provided to UCL CTC. UCL CTC will preserve patient confidentiality and will not disclose or reproduce any information by which patients could be identified. Data will be stored in a secure manner and UCL CTC trials are registered in accordance with the Data Protection Act 1998 with the Data Protection Officer at UCL. Patient identifiable data will be passed onto Birkbeck, University of London. This is described in the patient information sheet and the patients will consent to it.

For peer review only

For peer review only - http://bmjopen.bmj.com/site/about/guidelines.xhtml

18. Sponsorship and Indemnity

18.1. Sponsor Details

Sponsor Name: University College London

Address: Joint Research Office
Gower Street
London
WC1E 6BT

Contact: Director of Research Support

Tel: 020 3447 9995/2178 (unit admin)

Fax: 020 3447 9937

18.2. Indemnity

University College London holds insurance against claims from participants for injury caused by their participation in the clinical trial. Participants may be able to claim compensation if they can prove that UCL has been negligent. However, if this clinical trial is being carried out in a hospital, the hospital continues to have a duty of care to the participant of the clinical trial. University College London does not accept liability for any breach in the hospital's duty of care, or any negligence on the part of hospital employees. This applies whether the hospital is an NHS Trust or otherwise.

Hospitals selected to participate in this clinical trial shall provide clinical negligence insurance cover for harm caused by their employees and a copy of the relevant insurance policy or summary shall be provided to University College London, upon request.

For peer review only - http://bmjopen.bmj.com/site/about/guidelines.xhtml

19. Funding

This trial is funded by the National Institute for Health Research Health Technology Assessment (NIHR HTA).

For peer review only

For peer review only - http://bmjopen.bmj.com/site/about/guidelines.xhtml

20. Publication Policy

The TMG will oversee the publication and presentation of the data to peer reviewed journals and scientific meetings. All members of the TMG will approve publications. The writing committee will be led by Professor Stuart Taylor and include all TMG members. All TMG members, Trial Coordinator and Trial Statistician will be authors on the publications and named individually.

For peer review only

For peer review only - http://bmjopen.bmj.com/site/about/guidelines.xhtml

21. References

1. *Cancer Stats from Cancer Research UK Website*. Available from: http://info.cancerresearchuk.org.cancerstats/.
2. Garden, O.J., et al., *Guidelines for resection of colorectal cancer liver metastases.* Gut, 2006. **55 Suppl 3**: p. iii1-8.
3. Abdalla, E.K., et al., *Improving resectability of hepatic colorectal metastases: expert consensus statement.* Ann Surg Oncol, 2006. **13**(10): p. 1271-80.
4. Buyse, M., et al., *Progression-free survival is a surrogate for survival in advanced colorectal cancer.* J Clin Oncol, 2007. **25**(33): p. 5218-24.
5. *NICE Guidance: Improving Outcomes in Colorectal Cancers 2004*. Available from: http://www.nice.org.uk/nicemedia/pdf/CSGCCfullguidance.pdf.
6. Floriani, I., et al., *Performance of imaging modalities in diagnosis of liver metastases from colorectal cancer: a systematic review and meta-analysis.* J Magn Reson Imaging, 2010. **31**(1): p. 19-31.
7. Qu, X., et al., *A meta-analysis of (1)(8)FDG-PET-CT, (1)(8)FDG-PET, MRI and bone scintigraphy for diagnosis of bone metastases in patients with lung cancer.* Eur J Radiol, 2012. **81**(5): p. 1007-15.
8. Ohno, Y., et al., *Non-small cell lung cancer: whole-body MR examination for M-stage assessment--utility for whole-body diffusion-weighted imaging compared with integrated FDG PET/CT.* Radiology, 2008. **248**(2): p. 643-54.
9. Pierce, D.A. and D.L. Preston, *Radiation-related cancer risks at low doses among atomic bomb survivors.* Radiat Res, 2000. **154**(2): p. 178-86.
10. Schmidt, G.P., M.F. Reiser, and A. Baur-Melnyk, *Whole-body MRI for the staging and follow-up of patients with metastasis.* Eur J Radiol, 2009. **70**(3): p. 393-400.
11. Squillaci, E., et al., *Staging of colon cancer: whole-body MRI vs. whole-body PET-CT--initial clinical experience.* Abdom Imaging, 2008. **33**(6): p. 676-88.
12. Yi, C.A., et al., *Non-small cell lung cancer staging: efficacy comparison of integrated PET/CT versus 3.0-T whole-body MR imaging.* Radiology, 2008. **248**(2): p. 632-42.
13. Risberg, T., et al., *Diagnostic delay causes more psychological distress in female than in male cancer patients.* Anticancer Res, 1996. **16**(2): p. 995-9.
14. Bastiaannet, E., et al., *Perception of burden experienced during diagnostic tests by melanoma patients with lymph node metastases.* Melanoma Res, 2009. **19**(1): p. 36-41.
15. von Wagner, C., et al., *Choosing between CT colonography and colonoscopy in the diagnostic context: a qualitative study of influences on patient preferences.* Health Expect, 2009. **12**(1): p. 18-26.
16. Harcourt, D., N. Rumsey, and N. Ambler, *Same-day diagnosis of symptomatic breast problems: Psychological impact and coping strategies.* Psychology, Health & Medicine 1999. **4**(1): p. 5.
17. *NICE Guide to the methods of technology appraisal 2008*. Available from: http://www.nice.org.uk/media/B52/A7/TAMethodsGuideUpdatedJune2008.pdf.
18. Bossuyt, P.M., et al., *Towards complete and accurate reporting of studies of diagnostic accuracy: The STARD Initiative.* Radiology, 2003. **226**(1): p. 24-8.
19. Bossuyt, P.M., et al., *Comparative accuracy: assessing new tests against existing diagnostic pathways.* BMJ, 2006. **332**(7549): p. 1089-92.

For peer review only - http://bmjopen.bmj.com/site/about/guidelines.xhtml

20. Ryan, M., et al., *Use of discrete choice experiments to elicit preferences.* Qual Health Care, 2001. **10 Suppl 1**: p. i55-60.
21. Ryan, M. and S. Farrar, *Using conjoint analysis to elicit preferences for health care.* BMJ, 2000. **320**(7248): p. 1530-3.
22. Julious, S.A., M.J. Campbell, and D.G. Altman, *Estimating sample sizes for continuous, binary, and ordinal outcomes in paired comparisons: practical hints.* J Biopharm Stat, 1999. **9**(2): p. 241-51.
23. Newcombe, R.G., *Improved confidence intervals for the difference between binomial proportions based on paired data.* Stat Med, 1998. **17**(22): p. 2635-50.
24. Reitsma, J.B., et al., *A review of solutions for diagnostic accuracy studies with an imperfect or missing reference standard.* J Clin Epidemiol, 2009. **62**(8): p. 797-806.
25. White, I.R., P. Royston, and A.M. Wood, *Multiple imputation using chained equations: Issues and guidance for practice.* Stat Med, 2011. **30**(4): p. 377-99.
26. Begg, C.B., R.A. Greenes, and B. Iglewicz, *The influence of uninterpretability on the assessment of diagnostic tests.* J Chronic Dis, 1986. **39**(8): p. 575-84.
27. Briggs, A.H., D.E. Wonderling, and C.Z. Mooney, *Pulling cost-effectiveness analysis up by its bootstraps: a non-parametric approach to confidence interval estimation.* Health Econ, 1997. **6**(4): p. 327-40.
28. Cooper, K.L., et al., *Positron emission tomography (PET) and magnetic resonance imaging (MRI) for the assessment of axillary lymph node metastases in early breast cancer: systematic review and economic evaluation.* Health Technol Assess, 2011. **15**(4): p. iii-iv, 1-134.
29. Briggs, A.H., K. Claxton, and M.J. Sculpher, *Decision modelling for health economic evaluation*. Oxford handbooks in health economic evaluation. 2006, Oxford: Oxford University Press. x, 237 p.
30. Johnson, R., *Conjoint Analysis Software*, I. Sawtooth Software, Editor.: Sequim, WA
31. Azvolinsky, A., *Cancer prognosis: role of BMI and fat tissue.* J Natl Cancer Inst, 2014. **106**(6): p. dju177.
32. Yip, C., et al., *Assessment of sarcopenia and changes in body composition after neoadjuvant chemotherapy and associations with clinical outcomes in oesophageal cancer.* Eur Radiol, 2014. **24**(5): p. 998-1005.
33. Attaran, S., et al., *A propensity-matched comparison of survival after lung resection in patients with a high versus low body mass index.* Eur J Cardiothorac Surg, 2012. **42**(4): p. 653-8.
34. Curvo-Semedo, L., et al., *Diffusion-weighted MRI in rectal cancer: apparent diffusion coefficient as a potential noninvasive marker of tumor aggressiveness.* J Magn Reson Imaging, 2012. **35**(6): p. 1365-71.
35. De Felice, C., et al., *Apparent diffusion coefficient on 3.0 Tesla magnetic resonance imaging and prognostic factors in breast cancer.* Eur J Gynaecol Oncol, 2014. **35**(4): p. 408-14.
36. Ohno, Y., et al., *Diffusion-weighted MRI versus 18F-FDG PET/CT: performance as predictors of tumor treatment response and patient survival in patients with non-small cell lung cancer receiving chemoradiotherapy.* AJR Am J Roentgenol, 2012. **198**(1): p. 75-82.

For peer review only - http://bmjopen.bmj.com/site/about/guidelines.xhtml

Streamline C

37. Zakaria, R., et al., *Diffusion-weighted MRI characteristics of the cerebral metastasis to brain boundary predicts patient outcomes.* BMC Med Imaging, 2014. **14**: p. 26.

38. Parikh, J., et al., *Changes in primary breast cancer heterogeneity may augment midtreatment MR imaging assessment of response to neoadjuvant chemotherapy.* Radiology, 2014. **272**(1): p. 100-12.

For peer review only

For peer review only - http://bmjopen.bmj.com/site/about/guidelines.xhtml

Appendix 1: Abbreviations

**CDR**	Clinical Data Repository
**CI**	Chief Investigator
**CLRN**	Comprehensive Local Research Network
**CRF**	Case Report Form
**CSP**	Coordinated System for gaining NHS Permission
**CT**	Computerised Tomography
**CTSA**	Clinical Trial Site Agreement
**DCE**	Direct Choice Experiment
**DPA**	Data Protection Act
**GCP**	Good Clinical Practice
**GHQ**	General Health Questionnaire
**ICER**	Incremental Cost-Effectiveness Ratio
**ICH GCP**	International Conference of Harmonisation-Good Clinical Practice
**IDMC**	Independent Data Monitoring Committee
**IRAS**	Integrated Research Application System
**ISF**	Investigator Site File
**ISRCTN**	International Standard Randomised Controlled Trial Number
**ITT**	Intention To Treat
**MDT**	Multi-Disciplinary Team
**MRI**	Magnetic Resonance Imaging
**NCRI**	National Cancer Research Institute
**NCRN**	National Cancer Research Network
**NHS**	National Health Service
**NICE**	National Institute for Clinical Excellence
**NIHR**	National Institute for Health Research
**NIHR HTA**	National Institute for Health Research Health Technology Assessment
**NRES**	National Research Ethics Service
**OPD**	Out Patients Department
**PACS**	Picture Archiving and Communications System
**PANAS**	Positive and Negative Affect
**PET**	Positron Emission Tomography
**PET-CT**	Positron Emission Tomography- Computerised Tomography
**PI**	Principal Investigator
**PSS**	Personal Social Services
**QA**	Quality Assurance
**QALY**	Quality Adjusted Life Year
**R&D**	Research & Development
**RCT**	Randomised Controlled Trial
**REC**	Research Ethics Committee
**RIS**	Radiology Information System
**SSI**	Site Specific Information
**STARD**	STAndards for the Reporting of Diagnostic accuracy studies
**T**	Tesla
**TMF**	Trial Master File

For peer review only

For peer review only - http://bmjopen.bmj.com/site/about/guidelines.xhtml

Streamline C

**TMG**	Trial Management Group
**TSC**	Trial Steering Committee
**UCL**	University College London
**UCLH**	University College London Hospital
**UCL CTC**	Cancer Research UK & UCL Cancer Trials Centre
**USS**	Ultrasound Scan
**VATS**	Video-Assisted Thoracoscopic Surgery
**WB-MRI**	Whole Body Magnetic Resonance Imaging
**WCBP**	Woman of Childbearing Potential

For peer review only

For peer review only - http://bmjopen.bmj.com/site/about/guidelines.xhtml

Appendix 2: Consent process

Patients will be recruited at several hospital sites, and for many, the actual WB-MRI will take place at a different hospital (one of the central imaging hubs). It is vital that recruited patients undergo informed consent, have a minimum of 24 hours to consider participation and are subsequently free to withdraw at any time.
The consent process must be sensitive to the needs of patients which may differ from individual to individual. It is important therefore to provide patients with options as to how they may provide their consent.

The proposed consent process for this trial is based on the experience of other similar trials. It was found that patients and recruitment sites found it more efficient if the designated recruiting individual at the peripheral hospital knows that after fully explaining the trial, the patient in principle is willing to take part. For all recruitment sites, patients are seen by a clinical practitioner after a suspected diagnosis of colon cancer based on an abnormal endoscopy, CT scan or barium enema. At this discussion the diagnosis is explained and the need for a series of staging tests (such as CT scanning, PET CT, and MRI etc.) is also explained. Because of the need to stage the patient quickly so treatment may start, these tests are often done within a very short time period. There is no scheduled return by the patient to an OPD during this staging process. Instead patients are next seen face to face after the MDT has made a decision about their treatment.

If consent in principle is given, the recruiter can then ring the central hub and there and then arrange the time of the MRI and thus whenever possible inform the patient immediately of the time and date so they can plan their attendance for the scan. Patients expressing an interest in participating in the trial will therefore be asked if they prefer to sign the consent form when first seen when the trial is explained (and this will be later filed in the medical notes and the conversation documented). It will be made perfectly clear that they are free to withdraw at any time and that the scan will be at least 24 hours later (usually more than this). This will save them having to re-attend the hospital simply to sign a consent form, incurring time and expense at a very busy and difficult time for them. Before they go home, where possible patients will be given the date of the WB-MRI and a copy of their signed consent form. The recruitment site can then register the patient with UCL CTC. The date of the WB-MRI will be at least 24 hours later and in reality likely several days later. The patient will take the full patient information sheet home to review at their leisure. They will be provided with a telephone number at the recruitment site to ring if on reflection, they want to opt out of the trial. If they opt out, this will be recorded in the medical notes and screening log. Patients will also be asked permission to be called by the recruitment site or imaging hub to remind them of the date of scan and confirm their attendance. Patients will retain the right to opt out at any time by cancelling or not attending their WB-MRI appointment.

As noted, a major advantage of this approach is that the patient does not have to make a special trip back to the hospital just to sign the consent form. The signed consent

For peer review only - http://bmjopen.bmj.com/site/about/guidelines.xhtml

form does not leave the recruitment hospital and can be more efficiently filed in the patient notes and a copy filed in the Investigator site file: an important aspect of trial governance and patient clinical care.

Note this method of consent has been previously deemed ethically sound and efficient in the NIHR HTA funded Siggar 1 trial (CT colonography, colonoscopy or barium enema for diagnosis or colorectal cancer in older symptomatic patients), Northern and Yorkshire MREC, MREC/3/3/075, which ran from 2004 to 2009 and recruited over 5000 patients to undergo CT colonography in a similar trial design to the current proposal. The trial team did pilot a scheme where patients returned a copy of their signed consent form after 24 hours if they still wanted to take part, but patients found this very cumbersome and confusing and much preferred the process proposed, using telephone confirmation. The procedure is supported by the patient representatives in the trial and has also worked successfully in the BOOST trial which randomised patients to standard care or endobronchial biopsy at exactly the same point in the patient pathway. The BOOST trial ran at many of the intended recruitment sites for Streamline C.

Patients will be given the option to come back and sign the consent form if they or their recruiter feel this is preferable.

Occasionally it may be more convenient for the patient to obtain written consent when the patient attends for the WB-MRI or before a planned conventional staging test. For example the patient may wish to take part in the trial, not wish to sign the consent form on the same day but also would prefer not to attend a hospital appointment just to sign the consent form. This is acceptable although it is the least desirable option. After the patient has signed consent they will need to be registered before proceeding with the WB-MRI and a copy of the consent form will need to be returned to the initial recruitment site.

For peer review only

For peer review only - http://bmjopen.bmj.com/site/about/guidelines.xhtml

Appendix 3: Minimum WB-MRI protocol dataset

MRI Protocol
Whole body is head to thigh

The aim is to complete the whole protocol in 60 minutes or less. Depending on the MRI technology available, it may be possible to use the 5mm slices for all axial imaging, although up to 7mm is acceptable if time constraints are problematic.

1. These specifications are a set of minimum requirements – where higher field strengths/scanner software permits the resolution/quality of sequences should be optimised (whilst keeping the maximum imaging time limited to 1 hour).

2. Scanning maybe performed at either 1.5T or 3T.

3. Whole-body coverage is defined as head to mid-thigh optimised for detection of metastases.

Imaging Procedure

a) A standard safety questionnaire should be completed.
b) For patients undergoing contrast enhancement: set up IV line in a vein in the antecubital fossa, connected to an automated injector with two syringes (contrast and saline flush).
c) Unless contra-indicated administer 20mg buscopan or 1mg glucagon iv. to be given just before the start of the scan.

1. Whole-body diffusion weighted imaging:
Axial: STIR-EPI (or other fat sat technique) diffusion weighted imaging. Fixed slice thickness of 5mm to 7mm (to match T2 and t1 weighted axials as below) two b-values (b50 and b900). A minimum acquisition matrix of 128 x 128 (or an interpolated equivalent) (rectangular FOV should be used if available and appropriate for the patient), as a reference a minimum SNR of 6 on b50 images (for liver) should be maintained if possible by increasing the number of averages. All imaging should be performed in gentle respiration (recommended as 4 stations of 50 slices beginning from the vertex to mid thighs). Diffusion imaging through the brain is optional.

2. Whole-body T2 weighted imaging:
Axial*:* Axial T2 weighted (without fat-suppression) imaging should be performed from vertex to mid-thigh. A 5 to 7 mm slice thickness should be used for all scanners. *Where possible (within the 60 minute imaging time)* respiratory and ECG triggering should be used for the chest, respiratory triggering alone for the upper abdomen. The head, neck, pelvis and legs should be scanned without any triggering. The number of stacks should be adjusted to cover the imaging volume.

For peer review only - http://bmjopen.bmj.com/site/about/guidelines.xhtml

4. **Pre-contrast T1 weighted imaging:**

DIXON Technique to be applied if available.

a. **Axial**: Whole-body T1 GRE (e.g. Flash 2D) non-contrast enhanced non fat sat. Image resolution and slice thickness should be ideally matched to T2 weighted imaging.

OR

b. **Coronal**: T1 fat saturated volume interpolated gradient echo imaging (e.g. 3D) pre contrast.

5. **Post-contrast T1 weighted imaging (if gadolinium not contraindicated or refused):**

Minimum data set
Axial liver (60-70 sec)
Axial lung (equilibrium phase)
SFOV axial head

Optional
Coronal (organ specific or whole body)

a. **Axial:** post contrast e.g. T1 fat saturated volume interpolated gradient echo imaging (3D) breath hold of the *liver (60-70 seconds delay) and lungs*. Multiple breath-holds employed to provide full volume coverage if required. A minimum of a 256x256 (rectangular FOV acquisition if possible and appropriate for the patient) acquisition matrix should be employed. 5-7 mm slice thickness.
b. **Coronal:** post contrast whole body; e.g.T1 fat saturated volume interpolated gradient echo imaging (3D) and post contrast. Slice thickness 5mm. Breath Hold.
c. **Axial:** fat saturated T1 weighted imaging of the brain (SFOV). An acquisition matrix of 256 x 256 should be employed.

For peer review only - http://bmjopen.bmj.com/site/about/guidelines.xhtml

Appendix 4: Exploratory Analysis

The collated WB-MRI datasets and 12 months patient follow up data collated as part of the trial protocol provides opportunities to retrospectively mine the data for additional diagnostic indices and prognostic markers. All analysis will be done by members of the research team under supervision of the Chief Investigator.

There is for example, increasing data linking total body fat with prognosis in malignancy [31, 32]. In lung cancer, a higher body mass index has been associated with a better prognosis [33]. From the WB-MRI datasets acquired as part of the trial, it is possible to extract quantitative data on total body fat using simple segmentation algorithms which can be linked with patients' outcomes such as metastatic status and 12 month survival.

In addition, quantification of the MRI signal returned from sites of disease may be able to predict histological characteristics and also may be linked to prognosis. For example diffusion weighted imaging of the primary tumour has been shown to differentiate between grades of dysplasia in rectal cancer [34] and may predict prognosis in lung, brain and breast cancer [35-37]. The heterogeneity of signal within tumours can be measured and has been linked with overall prognosis and treatment response. For example in breast cancer, increased homogeneity of the signal measured using regions of interest placed in metastatic deposits in response to treatment is associated with a better prognosis [38]. The various sequences performed as part of the collected WB-MRI protocol will allow testing of such associations in recruited patients.

The following or similar may be quantified in all or a subset of WB-MRI datasets:

1. Body fat (organ, visceral and whole body).
2. MRI signal from the primary tumour and metastatic sites, notably diffusion weighted signal, apparent diffusion coefficient, T1 (including fat and water components), T2 signal, Contrast enhancement, and textural analysis (for heterogeneity) of the primary and metastatic deposits on the various sequences.

We will look for correlations between MRI variables and clinical data collated as part of the trial (for example basic histology, metastatic status, patient outcome and findings of conventional staging tests) or collected as part of the patients' usual care, for example, more detailed histology of biopsies or excised tumour (including tumours markers if collected as part of usual clinical care). Associations will be sought between the MRI variables and the clinical variables using appropriate conventional parametric and non-parametric statistics, for example:

- Differences in body fat composition between different cancer histological subtypes, patients with and without metastatic disease, and according to 12 month survival
- Heterogeneity of MRI signal from a region of interest drawn within the primary tumours and metastatic deposits, comparing between lesions in the same

For peer review only - http://bmjopen.bmj.com/site/about/guidelines.xhtml

patient, between different patients and according to histological parameters and 12 month survival.

In addition comparisons will be made between MRI signal within normal body tissues (such as the liver, pancreas and muscle) between patients scanned on the different MRI scanners comparing with local MRI phantom data as appropriate to see how homogenous the MRI data is between different imaging hubs.

In some instances use of external commercial software may be required (e.g. fat quantification). MRI datasets will be anonymised and no personal identifiable information will be shared outside the research team at any time.

For peer review only

For peer review only - http://bmjopen.bmj.com/site/about/guidelines.xhtml

Appendix 5: Protocol Version History

Protocol:		Amendments:		
**Version no.**	**Date**	**Amendment no.**	**Protocol Section (no./title)**	**Summary of main changes from previous version.**
1.0				
1.1	14/09/12	N/A	Front cover & Section 1.1<br>Section 5<br>8.2.10<br>Section 14.2<br><br>Appendix 2	ISRCTN number and new CR UK logo added<br><br>Flowchart and text re consent process inserted<br>Text inserted re QoLs<br>Text inserted re destruction of interview recordings<br>Text inserted re consent process
2.0	15/03/13	1.0	Throughout<br>Section 4<br><br>Section 5<br><br><br>Section 7<br><br>Section 8<br><br><br><br><br><br>Section 9<br><br>Section 12<br>Section 13<br>Appendix 3	Typos and clarifications<br>Clarification about GCP training and documents required for activation updated<br>Details added about interpreters for informed consent and removal of informed consent log information<br>Removal of text regarding slot availability updates<br>Addition of ad hoc mini MDT, reduce the amount of patient experience questionnaires, change the content of these questionnaires and alter who is sending them to patients.<br>Clarifications of timing of WB-MRI and the process of early release of findings<br>Update data management guidelines in line with changes at UCL CTC<br>Details about informed consent log removed<br>Addition of details re patient withdrawal<br>Clarifications to Whole body MRI protocol
3.0	24/04/14	3.0	Section 5<br><br><br><br>Section 7<br><br>Section 8	Removal of information about QoL and Patient diaries given out by site at consent<br>Removal of patient diaries and QoLs from registration packs.<br>Updating information to state that the Interview section of the trial is now closed to recruitment.<br>Updating information that UCL CTC is now posting out QoL and patient diaries at baseline.

For peer review only - http://bmjopen.bmj.com/site/about/guidelines.xhtml

Streamline C

4.0	22/12/14	5.0	Section 8<br><br>8.2.12<br><br>Section 10<br><br>Appendix 4<br><br>Appendix 5	Rewording and clarification of Conventional Imaging, WB-MRI reveal and MDT discussion, Final Treatment decision and follow up sections. Updating information to state that the Interview section of the trial is now closed to recruitment. Updating information that UCL CTC is now posting out QoL and patient diaries at baseline.<br>Information added regarding exploratory analysis for additional diagnostic indices and prognostic markers.<br>Safety Reporting section updated to provide more information on staging complications and how these are reported and reviewed by the CI.<br>Exploratory Analysis added to include the additional analysis of WB-MRI scans<br>Protocol Version History has become Appendix 5.
5.0	27/08/15	5.0	Section 8<br><br>Section 10<br><br>Section 13<br><br>Section 16	Changing the target recruitment for the patient experience questionnaires to 50 patients and closing this part of the trial. Updating and clarifying information regarding the Discrete Choice Experiment and confirming the time point these will be sent to patients.<br>Updating the Safety Section to confirm that only staging complications related to the WB-MRI or additional tests performed due to WB-MRI are to be recorded on the Staging Complications CRF.<br>The withdrawal process was updated and clarified to better match the new Change of Status CRF.<br>The type of Interim safety data that will be supplied to IDMC was updated
6.0	06/09/16	7.0	Section 1<br><br>Section 13<br><br>Section 16	Recruitment target increased from 322 to 360 in sections 1.1 and 1.2.<br>Anticipated duration of recruitment increased from 3 to 4 years in sections 1.1 and 1.2.<br>Deletion of section on early release of WB MRI and patient failure to undergo WB MRI<br>Rationale for revised target accrual added to 16.1.<br>Grammatical and typographical errors corrected throughout

For peer review only - http://bmjopen.bmj.com/site/about/guidelines.xhtml

COREQ (COnsolidated criteria for REporting Qualitative research) Checklist

A checklist of items that should be included in reports of qualitative research. You must report the page number in your manuscript where you consider each of the items listed in this checklist. If you have not included this information, either revise your manuscript accordingly before submitting or note N/A.

Topic	Item No.	Guide Questions/Description	Reported on Page No.
**Domain 1: Research team and reflexivity**			
Personal characteristics			
Interviewer/facilitator	1	Which author/s conducted the interview or focus group?	Page 8
Credentials	2	What were the researcher's credentials? E.g. PhD, MD	Page 8
Occupation	3	What was their occupation at the time of the study?	Page 8
Gender	4	Was the researcher male or female?	Page 8
Experience and training	5	What experience or training did the researcher have?	Page 8
Relationship with participants			
Relationship established	6	Was a relationship established prior to study commencement?	Page 7
Participant knowledge of the interviewer	7	What did the participants know about the researcher? e.g. personal goals, reasons for doing the research	Page 8
Interviewer characteristics	8	What characteristics were reported about the inter viewer/facilitator? e.g. Bias, assumptions, reasons and interests in the research topic	Page 8
**Domain 2: Study design**			
Theoretical framework			
Methodological orientation and Theory	9	What methodological orientation was stated to underpin the study? e.g. grounded theory, discourse analysis, ethnography, phenomenology, content analysis	Page 9
Participant selection			
Sampling	10	How were participants selected? e.g. purposive, convenience, consecutive, snowball	Page 9
Method of approach	11	How were participants approached? e.g. face-to-face, telephone, mail, email	Page 7
Sample size	12	How many participants were in the study?	Page 8
Non-participation	13	How many people refused to participate or dropped out? Reasons?	Page 8
Setting			
Setting of data collection	14	Where was the data collected? e.g. home, clinic, workplace	Page 7
Presence of non-participants	15	Was anyone else present besides the participants and researchers?	Page 7
Description of sample	16	What are the important characteristics of the sample? e.g. demographic data, date	Pages 10& 11
Data collection			
Interview guide	17	Were questions, prompts, guides provided by the authors? Was it pilot tested?	Box 1 and Page 8
Repeat interviews	18	Were repeat inter views carried out? If yes, how many?	Page 7
Audio/visual recording	19	Did the research use audio or visual recording to collect the data?	Page 8
Field notes	20	Were field notes made during and/or after the inter view or focus group?	Page 9
Duration	21	What was the duration of the inter views or focus group?	Page 8
Data saturation	22	Was data saturation discussed?	Page 8
Transcripts returned	23	Were transcripts returned to participants for comment and/or	Page 9

For peer review only

Topic	Item No.	Guide Questions/Description	Reported on Page No.
		correction?	
**Domain 3: analysis and findings**			
Data analysis			
Number of data coders	24	How many data coders coded the data?	Page 9
Description of the coding tree	25	Did authors provide a description of the coding tree?	Page 9
Derivation of themes	26	Were themes identified in advance or derived from the data?	Page 9
Software	27	What software, if applicable, was used to manage the data?	Page 9
Participant checking	28	Did participants provide feedback on the findings?	Page 9
Reporting			
Quotations presented	29	Were participant quotations presented to illustrate the themes/findings? Was each quotation identified? e.g. participant number	Pages 11-18
Data and findings consistent	30	Was there consistency between the data presented and the findings?	Pages 11-18
Clarity of major themes	31	Were major themes clearly presented in the findings?	Pages 11-18
Clarity of minor themes	32	Is there a description of diverse cases or discussion of minor themes?	Pages 11-18

Developed from: Tong A, Sainsbury P, Craig J. Consolidated criteria for reporting qualitative research (COREQ): a 32-item checklist for interviews and focus groups. *International Journal for Quality in Health Care*. 2007. Volume 19, Number 6: pp. 349 – 357

Once you have completed this checklist, please save a copy and upload it as part of your submission. DO NOT include this checklist as part of the main manuscript document. It must be uploaded as a separate file.

For peer review only
